# Evidence-based Integration of Environmental Sustainability into Clinical Guidelines for Operating Rooms: A Systematic Review

**DOI:** 10.1097/SLA.0000000000006756

**Published:** 2025-05-21

**Authors:** Kim E. van Nieuwenhuizen, Charlotte T.J. Michels, Ingena G.I.A. Both, Jeroen B. Guinée, Frank Willem Jansen, on behalf of the Dutch Expert Panel, Nicole D. Bouvy

**Affiliations:** * Department of Obstetrics and Gynaecology, Leiden University Medical Centre, Leiden, The Netherlands; † Knowledge Institute of the Dutch Association of Medical Specialists, Utrecht, The Netherlands; ‡ Department of Industrial Ecology, Institute of Environmental Sciences (CML), Leiden University, Leiden, The Netherlands; § Department of Biomedical Engineering, Technical University (TU) Delft, Delft, The Netherlands

**Keywords:** clinical guidelines, environmental impact, environmental sustainability, GRADE, life cycle assessment, operating room, surgery

## Abstract

**Objective:**

To assess the feasibility of integrating environmental sustainability into clinical guidelines for operating rooms (ORs) and provide evidence-based recommendations.

**Background:**

Surgical practices contribute significantly to health care’s environmental impact. Incorporating sustainability into evidence-based clinical guidelines can support environmentally responsible decision-making. However, guidance on integrating environmental considerations into clinical recommendations remains limited.

**Methods:**

A systematic review was conducted on the environmental impact of ORs, focusing on 5 key topics: (1) surgical techniques, (2) disposables versus reusables, (3) cover materials, (4) anaesthesia, and (5) OR ventilation. The search was conducted in December 2022 in PubMed (through NCBI), Embase (through OVID), Web of Science (through Webofscience), Cochrane (through Cochrane Library), and Emcare (through OVID). The Grading of Recommendations, Assessment, Development, and Evaluation (GRADE) approach was used to assess the quality of evidence and formulate recommendations.

**Results:**

A total of 42 studies were included, of which 28 employed life cycle assessment (LCA) methods. Grading of Recommendations, Assessment, Development, and Evaluation assessments indicated “very low” to “low” quality of evidence. Key contributors to the OR environmental impact included energy-intensive and resource-intensive surgical technologies, reliance on disposables, anaesthetic gas emissions, and energy consumption for OR ventilation.

**Conclusions:**

Despite “very low” to “low” levels of evidence, environmental outcomes consistently point in the same direction. Studies using LCA methods are instrumental in identifying environmental hotspots for targeted mitigation. Integrating LCA findings into clinical guidelines can support sustainability efforts in surgery, helping guideline panels develop evidence-based recommendations that promote environmentally responsible practices.

With the impacts of climate change escalating, prioritizing environmental sustainability in health care has become even more important within the broader framework of planetary health.^1^ While human activities contribute to environmental degradation, the health care sector is a notable contributor.^2^ Although health care aims to improve well-being, it paradoxically causes environmental harm, underscoring the need to embed sustainability into clinical practice through evidence-based guidelines.

The operating room (OR) is a primary contributor to the health care sector’s carbon footprint.^3^ The use of anesthetic gases and high energy consumption are major sources of greenhouse gas emissions, and the extensive use of disposables further exacerbates the environmental impact. As such, the OR serves as an exemplary case of how to integrate environmental impact considerations into clinical guidelines.

Health care professionals are increasingly relying on environmental science research, like life cycle assessments (LCAs) of health care products and services, to integrate sustainability into practice. LCAs evaluate the environmental impact of these products and services across their entire life cycle.^4^ Environmental science, especially LCAs, represents unfamiliar territory for many clinicians, and assessing the quality of these studies adds complexity. The volume of research on environmental impact in health care is growing, as is the number of systematic reviews.^5–8^ Most studies in these systematic reviews are LCAs, however, there is variation in the standards used to assess study quality. For example, the Standardized Technique for Assessing and Reporting Reviews of LCA checklist was developed by Zumsteg et al,^9^ based on the Preferred Reporting Items for Systematic Reviews and Meta-Analyses (PRISMA) statement. Subsequently, Drew et al^10^ developed a critical appraisal tool based on the Standardized Technique for Assessing and Reporting Reviews of LCA checklist for quality assessment of LCAs. Altogether, different approaches to assess the quality of LCAs are currently used.^5,7^

In clinical guideline development, the standard of Grading of Recommendations, Assessment, Development, and Evaluation (GRADE) can be used to assess the quality of the evidence.^11,12^ This internationally recognized approach facilitates decision-making in fields such as medicine, health policy and public health.^11,13^ Its use has expanded to include animal intervention and epidemiological studies.^14,15^ However, its application in planetary health is relatively new and is expected to increase.^16,17^ To our knowledge, LCAs and other environmental impact studies have not yet been incorporated into frameworks for clinical guidelines.^12,18^

This systematic review aims to gain insight into the integration of environmental impact into clinical guidelines and provide recommendations to reduce the environmental impact of the OR. By identifying areas of improvement and providing clinicians with an approach to integrate these into guideline development, environmental sustainability can be incorporated into daily practice, promoting planetary health.

## METHODS

A systematic review was conducted following the Preferred Reporting Items for Systematic Reviews and Meta-Analyses guidelines and Cochrane guidelines.^19^ The protocol is registered with the International Prospective Register of Systematic Reviews (PROSPERO, ID: CRD42022371108).

### Search Strategy and Selection Criteria

An interdisciplinary Dutch expert panel of 20 individuals, including clinicians, guideline developers, and experts in environmental science, epidemiology, and quality of evidence assessment, was formed (Appendix 1, Supplemental Digital Content 1, http://links.lww.com/SLA/F488). Through expert meetings and stakeholder consultations, the panel identified key topics related to the OR and environmental sustainability: (1) surgical techniques, (2) disposables versus reusables, (3) cover materials, (4) anesthesia, and (5) OR ventilation. A systematic review was conducted for each topic. Relevant literature was searched in PubMed (through NCBI), Embase (through OVID), Web of Science (through Webofscience), Cochrane (through Cochrane Library) and Emcare (through OVID). Studies were selected based on the following criteria: systematic reviews, randomized controlled trials (RCTs), (observational) comparative studies, LCAs, carbon footprint analyses (CFAs); full-text English or Dutch language publication; and studies according to the Population, Intervention, Comparator, and Outcome (PICO). Outcomes included only environmental impact outcomes. Details on PICO, inclusion/exclusion criteria, search strategy, and flowchart per topic are available in the Supplementary Materials (Appendix 2 and 3, Supplemental Digital Content 1, http://links.lww.com/SLA/F488).

### Data Analyses

Two reviewers (K.E.v.N. and I.G.I.A.B.) independently screened articles using Rayyan software (http://rayyan.ai, Rayyan Systems Inc.). The screening process involved title and abstract screening, followed by full-text review for eligibility. Discrepancies were resolved through consensus with a third reviewer (C.T.J.M.). One reviewer​​​​​​ (K.E.v.N.) performed data extraction using a Microsoft Excel template (MS Excel 2016; Microsoft Corp.) and organized the data into evidence tables (Appendix 4, Supplemental Digital Content 1, http://links.lww.com/SLA/F488). Studies were categorized into 4 groups: LCA, CFA using LCA methods, CFA without LCA methods, and “other.” LCAs meet the following criteria: (1) it covers the full life cycle, (2) is based on a functional unit (FU), (3) explicitly mentions LCA as a method, (4) uses an inventory database, and (5) includes more impact categories than just climate impacts. CFAs using LCA methods focus exclusively on climate impacts but meet LCA criteria. CFAs without LCA methods focus on climate impacts but do not meet LCA criteria. The “other” category includes studies such as observational studies and RCTs.

First, the study quality was assessed, followed by the quality of the evidence. For RCTs and observational studies, risk of bias was assessed using the tool of the CLARITY Group at Macmaster University.^20,21^ For LCAs and CFAs, the quality of the studies (eg, risk of bias) was assessed using the appraisal from the study by Drew et al,^10^ based on Weidema’s guidelines for critical review of LCA (Appendix 5, Supplemental Digital Content 1, http://links.lww.com/SLA/F488).

To align with existing guideline development standards, the GRADE approach was used to assess the quality of evidence.^11^ This approach determines evidence quality and the strength of recommendations in guideline development. We consulted experts and applied the GRADE approach as transparently and accurately as possible. For RCTs, GRADE suggests starting with a “high” quality rating, whereas observational studies start at “low.”^22^ Since studies using LCA methods are best in assessing environmental impact, the expert panel decided that these start at “high” quality. Following the steps of the GRADE approach, the expert panel formulated recommendations for each topic.

### Role of the Funding Source

The Dutch Association for Quality Funds Medical Specialists funded the development of this report but had no role in the study design, interpretation, writing, or decision to submit the paper.

## RESULTS

**FIGURE 1:**
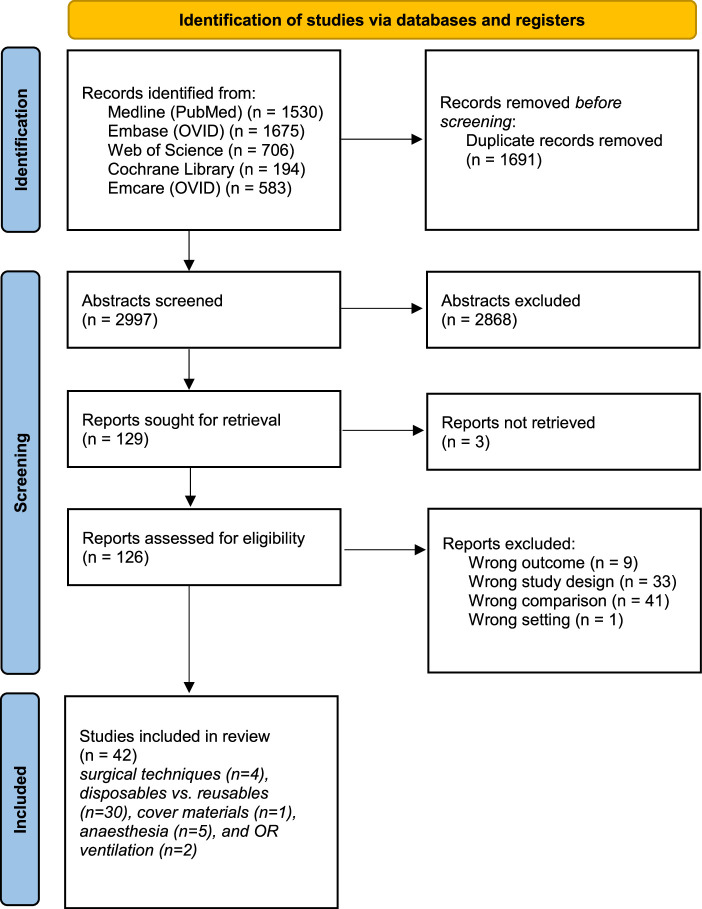
Flow chart of search results (adapted from PRISMA 2009 Flow Diagram).

The search was conducted in December 2022 and identified 4688 articles across the 5 topics. After removing 1691 duplicates, 2997 articles were screened, with 2868 excluded at the title-abstract screening stage. This left 129 full-text articles, of which 3 could not be retrieved. Of the remaining 126 articles, 42 met the inclusion criteria (Fig. 1). These 42 articles assessed the following topics: surgical techniques (n = 4), disposables versus reusables (n = 30), cover materials (n = 1), anesthesia (n = 5), and OR ventilation (n = 2).

The 42 articles contained various designs (n = 20 LCAs, n = 8 CFA using LCA methods, n = 6 CFA without LCA methods, n = 4 systematic reviews, n = 4 other studies). Tables 1, 2, and 3 show the study characteristics, and Appendix 4 (Supplemental Digital Content 1, http://links.lww.com/SLA/F488) provides an overview of the full evidence tables. Detailed information on the assessment of study quality and quality of evidence is provided in Appendix 5 (Supplemental Digital Content 1, http://links.lww.com/SLA/F488). Due to methodological heterogeneity—different methods, data sources, functional units, system boundaries, settings, and assumptions—the results could not be pooled. However, the environmental “hotspots” with the greatest impact and the highest potential for mitigation strategies were identified and embedded in the recommendations. These environmental hotspots are based on LCAs or CFAs using LCA methods, as these encompass all life cycle stages and, therefore, provide a comprehensive perspective. Partial analyses, such as CFAs without LCA methods focusing on specific stages, offer valuable additional insights but are limited in their scope. The recommendations for each topic are presented in Tables 4, 5, and 6.

**TABLE 1:** Study Characteristics—Topic 1 and (Part of) Topic 2

Study	Type of Study	Objective	Method	Setting and Country	Years of Data Collection	Surgical Discipline(s)
Topic 1: Surgical techniques						
Papadopoulou et al (2022)^*^ ^23^	Systematic review	To systematically review the MIS literature with the goal to investigate the carbon footprint of MIS, and other environmental impacts of MIS	Literature search between 1974 and July 2021. In accordance with 2020 PRISMA guidelines and the Cochrane Handbook of Systematic Reviews of Interventions	MIS, UK	—	MIS (robotic and laparoscopic surgery)
Power et al (2012)^24^	CFA without LCA methods	To assess the additional climate impact of MIS as compared with traditional open surgery	LCA-method undetermined	Inpatient and outpatient clinics in the USA	2009	Gastroenterology, obstetrics and gynecology, urology and nephrology
Thiel et al (2015)^25^	LCA	To assess the environmental impacts of 4 different surgical approaches to hysterectomy: vaginal, abdominal, laparoscopic, and robotic	Hybrid LCA (ISO 14040-44)	Hospital in the USA	2011	Obstetrics and gynecology
Woods et al (2015)^26^	CFA without LCA methods	To assess the climate impact, 3 surgical modalities for endometrial cancer staging: laparotomy, laparoscopy, robotic-assisted laparoscopy	LCA-method undetermined	Hospital in the USA	2008–2011	Obstetrics and gynecology; oncology
Topic 2: Disposables vs reusables						
Almutairi et al (2022)^27^	LCA	To assess and compare the environmental impact of different forms of PPE before and after COVID-19	Attributional LCA (ISO 14040–14044)	Dental University Hospital in the UK	2020–2021	Dental care
Boberg et al (2022)^28^	LCA	To assess the environmental impact of single-use, reusable, and mixed trocar systems used for laparoscopic cholecystectomies at 3 hospitals in southern Sweden	Attributional LCA (ISO 14044) (and conventional LCC)	3 hospitals in Sweden	—	Gastroenterology
Boucheron et al (2022)^29^	Other (single-centre retrospective study—waste and water audit)	To assess the environmental impact and costs of cystoscopies with disposable and reusable cystoscopes	—	Hospital in France	2020–2021	Urology
Davis et al (2018)^30^	CFA without LCA methods	To assess the climate impacts of 2 types of flexible ureteroscopes: single-use (LithoVue, Boston Scientific) and reusable (Olympus Flexible Video; typically 16 uses before repair and 180 uses before decommissioning)	LCA-method undetermined	Hospital in Australia	—	Urology and nephrology
Donahue et al (2020)^31^	CFA using LCA methods	To assess the climate impacts of 3 types of vaginal specula that are commonly used in practice (a single-use acrylic model and 2 reusable stainless steel models)	Attributional LCA (ISO 14040)	Hospital in the USA	—	Obstetrics and gynecology; oncology
Drew et al (2021)^*^ ^10^	Systematic review	To summarize the state of LCA practice through review of literature assessing the environmental impact of related services, procedures, equipment and pharmaceuticals	Literature search up to May 2020. The review used STARR-LCA, which is a PRISMA-based framework.	Anesthetic and surgical care, Canada	—	Nonspecific
Eckelman et al (2012)^32^	LCA	To assess the environmental impacts of 2 types of LMAs: single-use (Unique) and reusable (Classic; 40 lifetime uses)	Attributional LCA (ISO 14040)	Hospital in the USA	—	Anesthesiology
Friedericy et al (2022)^33^	LCA	To assess the environmental impact of the reusable RSC compared with blue wrap with closed-loop recycling	Attributional LCA (ISO 14040–14044)	Hospital in the Netherlands	—	Nonspecific
Grimmond et al (2012)^34^	CFA using LCA methods	To assess the climate impacts of 2 different sharps container systems (disposable and reusable) over a 12 mo period	Attributional LCA (PAS 2050)	Hospital in the USA	—	Nonspecific

^*^ Studies in all systematic reviews that met the inclusion criteria were already individually included.

No studies from this systematic review met the inclusion criteria.

COVID-19 indicates Coronavirus Disease 2019; LMA, laryngeal mask airway; MIS, minimally invasive surgery; PPE, ﻿Personal Protective Equipment; PRISMA, Preferred Reporting Items for Systematic Reviews and Meta-Analyses; RSC, ﻿Reusable Steriliz﻿ation Containers; STARR, Standardized Technique for Assessing and Reporting Reviews.

**TABLE 2:** Study Characteristics—Topic 2 (Continued)

Study	Type of Study	Objective	Method	Setting and Country	Years of Data Collection	Surgical Discipline(s)
Topic 2: Disposables vs reusables						
Grimmond et al (2021)^35^	CFA using LCA methods	To assess GWP of hospitals converting from single-use sharps containers to reusable sharps containers (SSC, RSC)	Attributional LCA (PAS 2050)	Acute care hospital trusts in the UK	2018–2019	Nonspecific
Hicks et al (2016)^36^	LCA	To assess environmental impact of reusable patient hospital gowns coated with nAg (nanosilver) product to the use of disposable gowns	Attributional LCA (unspecified)	USA	—	Nonspecific
Hogan et al (2022)^37^	CFA without LCA methods	To assess the carbon footprint of single-use vs reusable flexible cystoscopes based on waste production and estimated carbon emissions	LCA-method undetermined	Hospital in Cork, Ireland	2021	Urology
Ibbotson et al (2013)^38^	LCA	To assess the environmental and financial impacts of 3 types of surgical scissors: disposable plastic reinforced scissors, disposable stainless steel scissors, and reusable stainless steel scissors	Attributional LCA (ISO 14040)	Hospital in Germany	—	Nonspecific
Keil et al (2022)^*^ ^7^	Systematic review	To examine the change in environmental impact when switching from single-use to reusable products in health care	Literature search up to September 2021. The review used the PRISMA framework and used STARR-LCA	Focus on health care products, conducted in Germany	—	Nonspecific
Kemble et al (2022)^39^	CFA without LCA methods	To assess the carbon footprint of single-use and reusable flexible cystoscopes	LCA-method undetermined	USA	—	Urology
Le et al (2022)^40^	LCA	To assess the environmental and human health impacts of reusable and single-use duodenoscopes	Attributional LCA (unspecified)	USA	2020	Gastroenterology
Leiden et al (2020)^41^	LCA	To assess the environmental impacts of 2 types of instrument set for single-level lumbar fusion surgeries: disposable (Neo Pedicle Screw System from Neo Medical SA) and reusable (Viper 2 from DePuy Synthes, 300 uses)	Attributional LCA (ISO 14040)	Hospital in Germany	—	Neurology
McGain et al (2010)^42^	LCA	To assess the environmental and financial impacts of 2 types of commonly used plastic anesthetic drug trays: a single-use polyurethane tray made in China and reusable (300 uses) nylon tray made in Australia. Impacts and financial costs of 2 cotton gauzes and 1 paper towel, which are included with most single-use trays, were separately modeled	Attributional LCA (ISO 14040)	Hospital in Australia	—	Anesthesiology
McGain et al (2012)^43^	LCA	To assess the environmental and financial impacts of 2 types of central venous catheter insertion kits: single-use and reusable	Attributional LCA (ISO 14040)	Hospital in Australia	—	Anesthesiology
McGain et al (2017)^44^	LCA	To assess environmental and financial impacts of reusable and single-use anesthetic equipment	Consequential LCA (ISO 14040)	Hospitals in Australia	—	Anesthesiology
McPherson et al (2019)^45^	CFA using LCA methods	To assess the climate impacts of 2 different sharps container systems (disposable and reusable) over a 12 mo period at Loma Linda University Health in California, USA	Attributional LCA (PAS 2050)	Hospital in the USA	—	Nonspecific
Rodriguez Morris et al (2022)^46^	LCA	To assess the environmental impact of using disposable acrylic speculums vs using reusable stainless-steel speculums	Attributional LCA (ISO 14040)	University Clinic USA	—	Obstetrics and gynecology
Namburar et al (2021)^47^	Other (cross-sectional study—waste audit)	To measure the amount of waste generated during endoscopic procedures and to understand the impact on waste of changing from reusable to single-use endoscopes in the USA	—	2 academic medical centres in the USA	2020	Gastroenterology
Rizan et al (2021)^48^	LCA	To assess environmental and financial impacts of hybrid and single-use instruments in laparoscopic cholecystectomy	Attributional LCA (ISO 14044), consequential LCA and LCC	Hospital in the UK	2020	Gastroenterology

^*^ Studies in all systematic reviews that met the inclusion criteria were already individually included.

No studies from this systematic review met the inclusion criteria.

GWP indicates global warming potential; PRISMA, Preferred Reporting Items for Systematic Reviews and Meta-Analyses; STARR, Standardized Technique for Assessing and Reporting Reviews.

**TABLE 3:** Study Characteristics—Topic 2 (Continued), Topics 3, 4, and 5

Study	Type of Study	Objective	Method	Setting and Country	Years of Data Collection	Surgical Discipline(s)
Topic 2 Disposables versus reusables						
Sanchez et al (2020)^49^	LCA	To assess the environmental and economic impacts of reusable and disposable blood pressure (BP) cuffs	Attributional LCA (ISO 14040)	Outpatient clinic, ambulatory procedure rooms, regular ward and ICU in the US	—	Nonspecific
Sherman et al (2018)^50^	LCA	To assess the environmental and financial impacts of three different types of rigid laryngoscope handle and tongue blade: plastic single-use, metal single-use, and stainless steel reusable (under a range of cleaning options: low-level disinfection, high-level disinfection, sterilisation)	Attributional LCA (ISO 14040)	Hospital in the USA	—	Anaesthesiology
Sorensen et al (2022)^51^	LCA	To assess the environmental impact of either using a single-use double lumen tube (DLT) combined with a reusable bronchoscope or a single-use DLT with an integrated single-use camera	Attributional LCA (ISO 14040)	Hospital in Denmark	—	Thoracic surgery
Unger et al (2014)^52^	LCA	To assess the environmental impact of disposable and reusable dental burs	Attributional LCA (ISO 14040 – 14044)	Dental office in het USA	—	Dental care, oral surgery
Vozzola et al (2018)^53^	LCA	To assess the environmental impacts of two different isolation gowns: reusable and disposable	Attributional LCA (ISO 14040 – 14044)	USA	—	Nonspecific
Vozzola et al (2020)^54^	LCA	To assess the environmental impacts of two types of surgical gown: disposable and reusable	Attributional LCA (ISO 14040 – 14044)	USA	—	Nonspecific
Topic 3 (Cover) materials						
Nowack et al (2012)* ^55^	Systematic review	To identify environmental indicators for procurement decisions of low–value products	Literature search up to 2012. No specific framework used	Germany	—	Nonspecific
Topic 4 Anaesthesia						
Hu et al (2021)^56^	CFA using LCA–methods	To assess the carbon footprint of sevoflurane, isoflurane, desflurane and intravenous propofol and to provide evidence of the potential impact of Vapour Capture Technology	Attributional LCA (ISO 14040 – 14044)	UK	2018	Anaesthesiology
McGain et al (2021)^57^	CFA using LCA–methods	To assess the carbon dioxide equivalent emissions associated with general anaesthesia, spinal anaesthesia and combined (general and spinal) anaesthesia during total knee replacement	Attributional LCA (ISO 14040)	Hospital Australia	2019	Anaesthesiology
Sherman et al (2012)^58^	CFA using LCA–methods	To assess the environmental impact of 5 anaesthetic drugs – sevoflurane, desflurane, isoflurane, N2O and propofol – and to inform clinician drug selection on this basis	Attributional LCA (Unspecified)	Hospital in the USA	—	Anaesthesiology
Thiel (2018)^59^	CFA using LCA–methods	To assess the carbon footprint of various sustainability interventions used for laparoscopic hysterectomy	Hybrid LCA (ISO 14040–44)	Hospital in the USA	2016	Obstetrics & Gynaecology, Anaesthesiology
Wang (2022)^60^	CFA without LCA–methods	To assess the potential savings in carbon emissions for general anaesthesia versus spinal anaesthesia in transforaminal lumbar interbody fusions (TLIFs)	LCA–method undetermined	Hospital in the USA	—	Anaesthesiology & Neurosurgery
Topic 5 Anaesthesia						
Alsved et al (2018)^61^	Other (Comparative study)	To evaluate three types of ventilation systems for ORs with respect to air cleanliness, energy consumption and comfort of working environment as reported by surgical team members	—	Three different ORs in a hospital in Sweden	2015–2016	Orthopaedics
Marsault et al (2021)^62^	Other (Comparative study)	To compare how large, high volume, laminar airflow (LAF) and turbulent airflow (TAF) ventilation systems perform during standardized simulated total hip arthroplasty (THA)	—	Operating room, Denmark	2014–2015	Orthopaedics

* studies in all systematic reviews that met the inclusion criteria were already individually included

† no studies from this systematic review met the inclusion criteria.

**TABLE 4:** Recommendations and GRADE for Topics 1 and 2

PICO	Recommendations Per Topic	GRADE Per Outcome
Topic 1 ‘Surgical techniques’ Question: *What is the role of environmental sustainability of robot-assisted laparoscopic surgery compared with conventional laparoscopic surgery or open surgery?* P: patients who underwent surgery I: robot-assisted surgery C: conventional laparoscopic surgery or open surgery O: climate change, waste, acidification, eutrophication, human toxicity, ecotoxicity, ozone depletion	Be aware that robot-assisted surgery has a greater intra-operative environmental impact compared to other surgical techniques. This is primarily due to the high energy consumption and the use of disposables in robot-assisted laparoscopic surgery. The environmental impact should be considered when choosing a surgical technique. If there is no clear preference based on the literature conclusions and considerations regarding patient outcomes, then use the most sustainable surgical technique. If surgery is performed: • Take sustainability into account when choosing the surgical technique (R1-Refuse). • Pay attention to reducing the use of disposables (R2-Reduce). • Optimise the use of sustainable energy and energy-efficient equipment (R2-Reduce). • Incorporate sustainability into the (re)design of medical technologies (R3-Redesign) and alert the industry to this.	Climate change and waste (critical outcomes) Low GRADE Acidification, eutrophication, human toxicity, ecotoxicity, and ozone depletion (important outcomes) Very low GRADE Sources: *Power, 2012; Thiel, 2015; Woods, 2015*
Topic 2 ‘Disposables versus reusables’ Question 2.1: *What is the difference in sustainability of reusables compared to disposables in the operating room for patients who undergo surgery?* P=patients who undergo a surgical procedure I=reusables, such as: surgical gowns, scrub caps, gloves, glasses, perioperative textiles (i.e. blue drapes, band aids), packing materials, or laryngeal masks C=disposables, such as: surgical gowns, scrub caps, gloves, glasses, perioperative textiles (blue drapes, band aids), packing materials or laryngeal masks O=climate change, waste, acidification, eutrophication, human toxicity, ecotoxicity, ozone depletion Question 2.2: *What is the difference in sustainability of specific reusable medical instruments compared to disposable medical instruments in the operating room for patients who undergo surgery?* P=patients who undergo a surgical procedure I =reusable medical instruments, such as: specula, instruments, scopes (e.g. reusable instruments in a surgical tool kit: scissor, Kocher, tweezer, scalpel, needle driver, ligasure, harmonic, stapler, surgical drill; reusable scopes: duodenoscope, ureterorenoscope, bronchoscope, cystoscope, laryngeal scope; reusable meniscal sutures; reusable suture anchors). C=disposable medical instruments, such as: specula, instruments, scopes (e.g. disposable instruments in a surgical tool kit: scissor, Kocher, tweezer, scalpel, needle driver, vessel sealer, stapler, surgical drill; disposable scopes: duodenoscope, ureterorenoscope, bronchoscope, cystoscope, laryngeal scope; disposable meniscal sutures; disposable suture anchors) O=climate change, waste, acidification, eutrophication, human toxicity, ecotoxicity, ozone depletion	Preferentially use reusables, as disposables have a greater environmental impact (R4-Reuse). • Critically assess whether the use of a product is truly necessary (R1-Refuse) • If disposables are necessary, try to minimise their use (R2-Reduce). To reduce the environmental impact of reusables: • Optimise the cleaning, disinfection, and sterilisation process (e.g., by using sustainable energy, energy-efficient equipment). • Evaluate whether sterilisation is necessary in addition to cleaning and disinfection. • Optimise transportation (e.g., by using a more sustainable mode of transport, shortening transport distances). • Prefer reusables with the longest lifespan, as this reduces the environmental impact. • Incorporate sustainability into the (re)design of products, instruments, and equipment (R3-Redesign). • Highlight to the industry the importance of providing sustainable medical devices. • Incorporate waste management into redesign (e.g., by using fewer types of materials, by including clear waste separation indications, by promoting circularity).	Climate change, waste (critical outcomes) Very low GRADE* Acidification, eutrophication, human toxicity, ecotoxicity, and ozone depletion (important outcomes) Very low GRADE* Sources: *Almutairi, 2022; Boberg, 2022; Boucheron, 2022; Davis, 2019; Donahue, 2020; Eckelman, 2012; Friedericy, 2022; Grimmond, 2012; Grimmond, 2021; Hicks, 2016; Hogan, 2022; Ibbotson, 2013; Kemble, 2022; Le, 2022; Leiden, 2020; McGain, 2010; McGain, 2012; McGain, 2017; McPherson, 2019; Morris, 2022; Namburar, 2021; Rizan, 2021; Sanchez, 2020; Sherman, 2018; Sorensen, 2022; Unger, 2014; Vozzola, 2018; Vozzola, 2020*

* Given that all comparisons involve the assessment of reusable versus disposable medical devices, conclusions regarding the level of evidence of literature from sub question 2.1 and sub question 2.2 are presented in one overview.

**TABLE 5:** Recommendations and GRADE for Topics 3 and 4

PICO	Recommendations Per Topic	GRADE Per Outcome
Topic 3 ‘(Cover) materials’ Question: *What is the effect on environmental sustainability of disposable materials (i.e. heat blankets, surgical drapes, disposable duvets, cellulose pads) in comparison with alternative reusable or sustainable (e.g. biobased) materials that are in contact with patients on the operating table?* P: Patients on the operating table I: Reusable or sustainable (e.g. bio-based) alternative for heat blanket (bair hugger), surgical drapes, disposable duvet and cellulose pads C: Use of disposable heat blanket (bair hugger), disposable surgical drapes, disposable duvet and disposable cellulose pads O: Climate change, waste, water use, land use, energy use	Evaluate whether the use of materials that come into contact with the patient (such as warming blankets, absorbent mats, drapes, disposable duvets) is truly necessary (R1-Refuse, R2-Reduce). • Choose reusable materials whenever possible. Reusable covering materials are generally more sustainable in use. For example, reusable alternatives for drapes, absorbent mats, and duvets are available on the market. • Optimise existing protocols and incorporate sustainability. Assess what is necessary for the patient on a case-by-case basis. • Intensify collaboration with infection prevention to make a risk assessment where the risks of infection/contamination are balanced against sustainability measures. Consult infection prevention guidelines. Optimise the circularity of materials by promoting redesign (R3-Redesign) and implementing circularity in the design. Extend the lifespan of materials and promote reuse whenever possible (R4-Reuse) and inform the industry about this.	Climate change, waste (critical outcomes) Water use, energy use, land use (important outcomes) Sources: No studies included. Recommendations were based on expert opinion, grey literature, and using the R-ladder of circularity.
Topic 4 ‘Anaesthesia’ Question 4.1: *What is the effect on environmental sustainability of inhalation anaesthetics compared with the use of intravenous anaesthesia in patients undergoing surgery?* P=patients undergoing a surgical procedure under general anaesthesia I=inhalation anaesthetics C=intravenous anaesthetics O=climate change, waste, medicine residue in water, human toxicity, ozone depletion Question 4.2: *What is the effect on environmental sustainability of inhalation anaesthetics while using Vapour Capture Technology compared with the use of inhalation anaesthetics while not using Vapour Capture Technology in patients undergoing surgery?* P=patients undergoing a surgical procedure under anaesthesia I =inhalation anaesthetics with use of Vapour Capture Technology C=inhalation anaesthetics without use of Vapour Capture Technology O=climate change, waste, medicine residue in water, human toxicity, ozone depletion Question 4.3: *What is the effect on environmental sustainability of (loco)regional anaesthesia and local anaesthesia compared with the use of general anaesthesia in patients undergoing surgery?* P=patients undergoing a surgical procedure under anaesthesia I =(loco)regional anaesthesia and local anaesthesia C=general anaesthesia O=climate change, waste, medicine residue in water, human toxicity, ozone depletion	Be aware that inhalation anaesthetics have a greater environmental impact compared to intravenous anaesthesia. If there is no clear preference based on the literature conclusions and other considerations, use intravenous anaesthesia instead of inhalation anaesthetics (R1-Refuse). • Minimise the waste of propofol by drawing up the medication precisely (R2-Reduce). If the use of inhalation anaesthetics is preferred, minimise the amount. Consider: • Using low-flow anaesthesia (0.3-0.5 L/min) and a ventilator with End-tidal function (R2-Reduce). • Using sevoflurane or isoflurane (R1-Refuse). Avoid the use of desflurane and nitrous oxide. • VCT requires further research to evaluate its effectiveness and environmental sustainability before widespread implementation.	Question 4.1: Climate change (critical outcome) Very low GRADE Waste (critical outcome), medicine residue in water, human toxicity, ozone depletion (important outcomes) Outcomes not reported Sources: *Sherman, 2012; Thiel, 2018* Question 4.2: Climate change (critical outcome) Very low GRADE Waste (critical outcome), medicine residue in water, human toxicity, ozone depletion (important outcomes) Outcomes not reported Sources: *Hu, 2021* Question 4.3: Climate change, waste (critical outcomes) Very low GRADE Medicine residue in water, human toxicity, ozone depletion (important outcomes) Outcomes not reported Sources: *McGain, 2021; Wang, 2022*

VCT indicates vapour capture technology.

**TABLE 6:** Recommendations and GRADE for Topic 5

PICO	Recommendations Per Topic	GRADE Per Outcome
Topic 5: “OR ventilation” Question 5.1: What is the role of environmental sustainability outcomes regarding the different OR air ventilation criteria (low (class 2), medium (class 1) or high (class 1+) during surgical procedures?^*^ P = surgical procedures I = high, medium air ventilation criteria C = low air ventilation criteria O = climate change, energy use Question 5.2: What is the role of environmental sustainability outcomes across the entire life cycle of the a mixed air handling system in comparison to suppressing semi-suppressing air handling system in surgical procedures? P = surgical procedures I = mixed air handling system (eg, inlet grilles) C = suppressing or semi-suppressing air handling system (eg, unidirectional laminar downflow/plenum, Opragon, Halton) O = climate change, energy use	Be aware that vertical LAF has the highest energy usage, followed by TMA and TAF Assess which OR air ventilation criteria are needed for each surgical indication Perform surgeries in an OR equipped with the appropriate air treatment criteria suitable for the type of surgery (R1-Refuse, R2-Reduce). Consider this in the scheduling of operations If the surgical indication allows, consider using the outpatient treatment room. At an institutional level, ensure that air treatment systems are properly configured. Minimize the use of air treatment (R1-Refuse, R2-Reduce) Implement sequential usage, turning on where necessary and off where possible. This includes considering the number of air changes per hour, air humidification, temperature, and relative humidity (R2-Reduce)	Question 5.1: No studies included. Question 5.2: Energy use (critical outcome) Very low GRADE Climate change (critical outcome) Outcome not reported Sources: Alsved, 2018; Marsault, 2021

* See appendix 2 in the supplementary material for more details on the air ventilation criteria.

The expert panel embedded the R-ladder of circularity in the recommendations.^63^ The R-ladder outlines strategies for achieving a circular economy, applied here to promote circular health care. Strategies positioned at the higher levels of the R-ladder are associated with reduced resource consumption and are thus anticipated to be more sustainable. In topics where LCAs are insufficient or unavailable, the expert panel employed the R-ladder of circularity as a guiding framework when formulating recommendations on health care-related issues. LCAs provide critical evidence to evaluate whether the top levels of the R-ladder for a particular product indeed offer greater sustainability compared with alternative options.

### Surgical Techniques

For “surgical techniques,” 4 studies were identified: 1 systematic review,^23^ 1 LCA,^25^ and 2 CFAs without LCA methods.^24,26^ No new studies emerged from the systematic review. The 3 studies^24–26^ consistently show robot-assisted laparoscopic surgery has the greatest environmental impact compared with conventional laparoscopic or open surgery. Open surgery has the lowest environmental impact. Note that the postoperative period (eg, length of stay, complications, and readmissions) was not considered, which could influence the environmental impact. This should be factored in when evaluating a surgical technique’s environmental impact. The main environmental hotspots for the critical outcome (climate change) in laparoscopic and robot-assisted surgical techniques are the production of disposables and use of anesthetic gases, and for open and vaginal approaches the use of anesthetic gases.^25^ For important outcomes (acidification, eutrophication, human toxicity, ecotoxicity, and ozone depletion), the main hotspots for surgical techniques are the production and disposal of disposables.^25^

The critical appraisal of the CFAs without LCA methods^24,26^ yielded quality scores of 54% and 57%, whereas the LCA^25^ resulted in a score of 80% (Appendix 5, Supplemental Digital Content 1, http://links.lww.com/SLA/F488). The outcomes considered critical were “climate change” and “waste”, the other outcomes were considered important. “Climate change” and “waste” have been assessed as “low” GRADE, and other outcomes as “very low” GRADE. Therefore, the overall level of evidence is “low.”^12^ Despite this, results of the studies point in the same direction regarding surgical techniques. Given this consistent trend and the urgency to reduce environmental impact, it is considered sufficient support for the expert panel to formulate strong recommendations.

### Disposables versus Reusables

For “disposables versus reusables” 30 studies were identified, consisting of 2 systematic reviews,^7,10^ 19 LCAs,^27,28,32,33,36,38,40–44,46,48–54^ 4 CFAs using LCA methods,^31,34,35,45^ 3 CFAs without LCA methods,^30,37,39^ and 2 “other” studies.^29,47^ No additional studies emerged through the 2 systematic reviews. This resulted in a total of 28 included studies.

Of 28 included studies, 22 reported reusables to have a lower environmental impact than disposables across most PICO outcomes.^27,28,31–36,38–40,42,45–50,52–54^ Where disposables performed equal or better, studies used a different energy mix (than in Europe, which is used as a reference)^30,37^ or there is a significant difference in weight.^41,43^ Boucheron et al^29^ show disposable cystoscopes have lower impacts based on waste and water use, but lacked a full life cycle or CFA. Almutairi et al^27^ describe marine eutrophication as having a higher impact for reusable gowns compared with disposables, but all other outcomes favor reusables. Sorensen et al^51^ compare single-use devices in combination with a reusable device. The details, assumptions, and settings of the studies should be carefully considered when extrapolating results. For disposables, the production process is identified as the primary environmental hotspot.^27,28,31–36,38,40–46,48–54^ See for more details Appendix 4 (Supplemental Digital Content 1, http://links.lww.com/SLA/F488).

The cleaning and sterilization process of reusables poses the highest environmental burden, with hotspots being energy use and steam for autoclaves, transportation, and water consumption.^31,32,34,35,45^ Evaluating whether disinfection alone suffices in certain cases is important, as it requires less energy and is, therefore, more sustainable.

Various modes of transportation influence the environmental impact of disposables and reusables in different ways. Eckelman et al^32^ compared railway transport with alternatives like road and air transport, finding that while alternative transport methods have a relatively minor effect on the environmental impact of reusables, disposables experience a substantial increase, especially when transported by air travel.

The critical appraisal of the LCAs and CFAs using LCA methods^27,28,31–36,38,40–46,48–54^ yielded quality scores ranging from 66% to 100%, the CFAs without LCA methods and other studies^29,30,37,39,47^ ranged from 34% to 66%. Critical outcomes were “climate change” and “waste,” which were assessed as “very low” GRADE (Appendix 5, Supplemental Digital Content 1, http://links.lww.com/SLA/F488). Despite this, the majority of LCAs and other studies show results pointing in the same direction,^27,28,31–36,38–40,42,45–50,52–54^ and in cases where they did not,^29,30,37,41,43,51^ reasons for divergent outcomes were identified. Therefore, this collective evidence is deemed sufficient by the expert panel to formulate strong recommendations.

### Cover Materials

The environmental impact of cover materials has been examined, which included, for example, surgical drapes, heat blankets, and absorbency mats. One systematic review was identified, which included studies investigating the environmental impact of sterile surgical drapes.^55^ As these studies were not reported in English or Dutch, they were excluded.

As no studies met the inclusion criteria, it was not possible to conduct a critical appraisal, and no GRADE could be assessed. Therefore, the expert panel formulated recommendations based on expert opinion, grey literature, and the R-ladder of circularity.^63^ One Dutch LCA report^64^ compared reusable and disposable surgical drapes. Reusable surgical drapes were available from 2 suppliers. Results showed that among 6 different types of drapes, only the reusable side drapes from one supplier had a greater climate impact compared with the disposable alternatives. Hotspots differed for both types of surgical drapes (disposable drapes: production and end-of-life phases; reusable drapes: materials used in the production process, the weight and amount of material used, the self-adhesive tape). Reusable surgical drapes can potentially be a sustainable alternative to disposable surgical drapes, however, results should be carefully interpreted and related to the specific setting.

### Anesthesia

Three different PICOs were developed for anesthesia (Appendix 2, Supplemental Digital Content 1, http://links.lww.com/SLA/F488). Five studies were included, consisting of 4 CFAs using LCA methods^56–59^ and 1 CFA without LCA methods^60^ (details in Appendix 4, Supplemental Digital Content 1, http://links.lww.com/SLA/F488).

The first comparison is between inhalation versus intravenous anesthetics. Three studies suggest that general anesthesia using inhalation anesthetics has a greater environmental impact than intravenous anesthesia.^56,58,59^ Replacing inhalation with intravenous anesthetics could yield environmental benefits, but careful consideration is needed per patient.

The second comparison is the use of inhalation anesthetics with or without vapour capture technology (VCT). Hu et al^56^ find that the use of VCT results in similar environmental impacts for inhalation anesthetics (sevoflurane and isoflurane) compared with intravenous anesthesia, but only if nitrous oxide is avoided and low flow (0.5 L/min) is maintained. Despite VCT use, or avoidance of nitrous oxide, desflurane still has a higher impact compared with sevoflurane or isoflurane.^56^

The comparison between (loco)regional or local anesthesia and general anesthesia requires cautious interpretation due to methodological differences. McGain et al^57^ found spinal anesthesia to have a higher environmental impact than general or combined anesthesia, driven by higher oxygen flow rates (6–10 vs 0.5–3 L/min), longer surgery durations (200 vs 161–189 minutes), and disposable use. The study was conducted in Australia and reflects a coal-heavy energy mix. In contrast, Wang et al,^60^ conducted in the USA, found spinal anesthesia to have a lower CO_2_ footprint than general anesthesia, but their analysis excludes materials, energy use, production, the use phase, and disposal.

The critical appraisal of CFAs using LCA methods yielded quality scores ranging from 77% to 91%,^56–59^ whereas the CFA without LCA methods resulted in a score of 31%.^60^ The critical outcomes “climate change” and “waste” were assessed as “very low” GRADE. The 3 CFAs using LCA methods consistently indicate the major areas of impact. Given this consistency and the urgency to reduce environmental impact, the expert panel formulated strong recommendations.

### Operating Room Ventilation

Regarding OR ventilation, 2 PICOs were developed. No studies were identified for PICO 1 (low, medium or high OR air ventilation criteria^65^—see Appendix 2 (Supplemental Digital Content 1, http://links.lww.com/SLA/F488), while 2 comparative studies were found for PICO 2 (mixed air handling systems vs suppressing or semisuppressing air handling systems).^61,62^ No LCAs or CFAs using LCA methods were available, but both studies highlight high energy consumption as a key improvement area.

Alsved et al^61^ quantified energy consumption for ventilation power across airflow types, comparing vertical laminar airflow (LAF), turbulent mixed airflow, and temperature-controlled airflow turbulent airflow (TAF). Laminar airflow (LAF) consumed the most energy, followed by TAF and turbulent mixed airflow. TAF is more energy-efficient than LAF while maintaining high air cleanliness. Marsault et al^62^ show that when the amount of fresh air supplied is halved, the energy consumption is also nearly halved. If an OR is not in use, it is unnecessary to run the air treatment system with the same intensity as when a patient is being operated on. Hospitals should assess the number of acute ORs requiring full operation.

The risk of bias assessment resulted in “some concerns” for Alsved et al^61^ and “high concerns” for Marsault et al.^62^ The critical outcome measure “climate change” was unreported, and the critical outcome measure “energy use” was assessed as “very low” GRADE.

## DISCUSSION

Our systematic review identifies key environmental hotspots in the OR. The quality of evidence was assessed using the GRADE approach,^12^ and recommendations were made for each identified topic. Despite the overall evidence being rated as “low” to “very low,” the consistent identification of which aspects—such as the choice of surgical technique or type of anesthesia—have a greater impact, and similar hotspots across studies, provides clear guidance for targeting interventions to reduce the OR’s environmental impact. Differences in setting (eg, energy mixes per country) must be carefully considered, as these can lead to different recommendations.

Regarding surgical techniques, robot-assisted surgery has the greatest intraoperative environmental impact compared with laparoscopic and open surgery. When multiple techniques yield comparable patient outcomes, sustainability should be a guiding factor in decision-making. Robot-assisted surgery should be carefully weighed against conventional laparoscopic surgery. The expert panel recommends prioritizing the lowest-impact techniques,^25^ and integrating sustainability into medical technology development. Identifying hotspots, such as energy use and the use of disposables, helps mitigate their impact, requiring interdisciplinary collaboration among engineers, environmental scientists, and clinicians. Evaluating the necessity of surgery is essential, aligning with the R-ladder’s highest level (Refuse)^63^ and benefiting both public and environmental health.

For disposables versus reusables, the increased use of disposables in health care has greatly impacted the environment. In nearly all cases, switching to reusables is the preferred solution. However, for some advanced devices, equivalent reusable alternatives are not yet available. To address this, integrating sustainability into product design is crucial, requiring close collaboration with industry.

Regarding cover materials, current practices should be critically assessed, thereby optimizing the use of cover materials, and prioritizing reduction and reuse. In health care, decisions are evidence-based, yet in the absence of evidence, we default to maximum sterility, which is not always necessary and often results in excessive material use. While patient safety is crucial, sustainability can coexist with safety. Safety is perceived as a barrier to sustainability,^66^ but unnecessary fear of infection and nonevidence-based practices like disposable overuse are increasingly recognized.^67^ Sterility requirements should balance safety and sustainability, for example, by reducing absorbent mats and surgical drapes, or replacing them with reusables. Surgeries like curettage or manual placental removal may not require full sterile draping, and sterile gowns and full draping in minimally invasive surgery should be reconsidered. Furthermore, reusable draping is a sustainable alternative, as evidence of low quality shows no clear difference in the risk of postoperative wound infections between disposable and reusable surgical drapes and gowns.^68^ Therefore, increasing knowledge of sustainable alternatives among health care professionals is essential.^67^ Medical associations should consult infection prevention experts to update sterility guidelines by procedure, while health care professionals should be aware of existing OR infection prevention guidelines.^69^

Regarding anesthesia, intravenous anesthesia has a lower environmental impact than inhalational anesthesia and should be preferred when there are no clinical contraindications.^58,59^ The use of VCT to reduce the impact of inhalational anesthetics is still an emerging field. Hu et al^56^ assume a recapture rate of 70%, while Hinterberg et al^70^ measure an in vivo recapture rate of only 25%. Differences in recapture rate affect the environmental impact. Further research is needed before implementing VCT without solid evidence on environmental impact and effectiveness.

Regarding OR air ventilation, saving energy in air ventilation systems can help reduce environmental impact. Turning off unidirectional systems during nights and weekends can save up to 70% of total energy use.^71^ The OR can be safely used again within 30 minutes, maintaining air quality. In addition, not all surgeries may require the highest air ventilation criteria.^65^ The majority of procedures can be performed in ORs with “medium” air ventilation criteria,^65,72^ while minimally invasive surgery could potentially be safely performed in ORs with “low” air ventilation criteria, offering energy reduction.^62^

Our systematic review primarily involves studies using LCA methods, which, to our knowledge, have not been incorporated into clinical guideline development, necessitating a novel approach from guideline developers. A challenge is ensuring that LCAs, evaluated through the GRADE approach, can support strong recommendations.

LCA is a method used to evaluate the environmental impact of a product or process, from raw material extraction to disposal.^4^ In health care, LCA can help identify the most sustainable choices for surgical instruments, energy use, or disposable versus reusable materials. However, a major challenge is the lack of standardization in LCA methodologies—due to variations in functional units (what exactly is being measured), system boundaries (what is included in the analysis), and underlying assumptions. This makes it difficult to compare results across studies and draw straightforward conclusions. For example, one study might assess the carbon footprint of a surgical procedure by including only direct emissions (eg, energy use in the OR), while another study might also consider emissions from manufacturing and waste disposal. A recent publication by Keil et al^73^ highlights the use of various methods to calculate the health care sector’s carbon footprint, underlining the need for standardization. Health care professionals should be aware of these differences in methodologies and interpret results with caution. When using LCA findings to inform decisions, they may need to consult experts in the field to ensure a proper evaluation of the results. Without a consistent approach, it remains challenging for health care professionals to make informed, evidence-based choices about sustainability in their daily practice.

While many LCAs are conducted and environmental hotspots identified, emphasis should be placed on implementing findings in clinical practice. Action is needed to achieve zero emissions. High-income countries have a relatively large footprint, and beyond reducing our own impact, it is crucial to prevent low- and middle-income countries from adopting unsustainable practices—such as the excessive use of disposables. It is crucial to develop health care systems with sustainability as a core consideration.

The systematic review has several limitations. First, only peer-reviewed studies published in Dutch and English were included. Second, the GRADE approach has not previously been applied to LCAs, making this a first exploration. Consequently, methods may require further application for robust validation and potential refinement.

ORs are designed to improve patient health, but also contribute to environmental impact, including climate change, which in turn harms human health. Adopting sustainable practices in ORs can help mitigate these effects while benefiting patients. The expert panel anticipates that sustainability efforts will often lower costs, particularly by following the R-ladder principles (eg, Refuse, Reduce). While transitioning to reusable alternatives may require initial investments, these can be offset by long-term savings. To successfully implement sustainable practices, hospital leadership must prioritize these initiatives and allocate resources effectively.

## CONCLUSIONS

This study represents a first step in integrating environmental sustainability into clinical guidelines, using the OR as an example. Our findings highlight key areas for intervention, including surgical techniques, anesthesia choices, disposable versus reusable materials, and OR energy use. Standardization of LCA methodologies is essential for generating robust evidence to guide decision-making. Health care systems must embed sustainability into routine practice, ensuring that future medical advancements do not come at an unnecessary environmental cost. By taking action now, surgeons and health care professionals can help build a more sustainable and resilient health care system.

## Supplementary Material

## References

[1] Romanello M Walawender M Hsu S-C . The 2024 report of the”Lancet” countdown on health and climate change: facing record-breaking threats from delayed action. Lancet. 2024;404:1847–1896. 39488222 10.1016/S0140-6736(24)01822-1 PMC7616816

[2] Karliner J Slotterback S Boyd R . Health care’s climate footprint: how the health sector contributes to the global climate crisis and opportunities for action [Internet]. Health Care Without Harm; 2019. Accessed May 24, 2024. https://noharm-global.org/sites/default/files/documents-files/5961/HealthCaresClimateFootprint_092319

[3] MacNeill AJ Lillywhite R Brown CJ . The impact of surgery on global climate: a carbon footprinting study of operating theatres in three health systems. The Lancet Planetary Health. 2017;1:e381–e388. 29851650 10.1016/S2542-5196(17)30162-6

[4] Guinée JB Heijungs R Huppes G . Life cycle assessment: past, present, and future. Environ Sci Technol. 2011;45:90–96. 20812726 10.1021/es101316v

[5] Rizan C Steinbach I Nicholson R . The carbon footprint of surgical operations: a systematic review. Ann Surg. 2020;272:986–995. 32516230 10.1097/SLA.0000000000003951

[6] Rodríguez-Jiménez L Romero-Martín M Spruell T . The carbon footprint of healthcare settings: a systematic review. J Adv Nurs. 2023;79:2830–2844. 37198974 10.1111/jan.15671

[7] Keil M Viere T Helms K . The impact of switching from single-use to reusable healthcare products: a transparency checklist and systematic review of life-cycle assessments. Eur J Public Health. 2022;33:56–63. 10.1093/eurpub/ckac174 PMC9898010 36433787

[8] McGain F Naylor C . Environmental sustainability in hospitals–a systematic review and research agenda. J Health Serv Res Policy. 2014;19:245–252. 24813186 10.1177/1355819614534836

[9] Zumsteg JM Cooper JS Noon MS . Systematic review checklist: a standardized technique for assessing and reporting reviews of life cycle assessment data. J Ind Ecol. 2012;16:S12–s21. 26069437 10.1111/j.1530-9290.2012.00476.x PMC4461004

[10] Drew J Christie SD Tyedmers P . Operating in a climate crisis: a state-of-the-science review of life cycle assessment within surgical and anesthetic care. Environ Health Perspect. 2021;129:76001. 34251875 10.1289/EHP8666 PMC8274692

[11] Guyatt GH Oxman AD Vist GE . GRADE: an emerging consensus on rating quality of evidence and strength of recommendations. Brit Med J. 2008;336:924–926. 18436948 10.1136/bmj.39489.470347.AD PMC2335261

[12] Schünemann H Brożek J Guyatt G . GRADE handbook for grading quality of evidence and strength of recommendations. The GRADE Working Group; 2013. Accessed June 10, 2024. http://guidelinedevelopment.org/handbook

[13] Guyatt GH Oxman AD Schünemann HJ . GRADE guidelines: a new series of articles in the Journal of Clinical Epidemiology. J Clin Epidemiol. 2011;64:380–382. 21185693 10.1016/j.jclinepi.2010.09.011

[14] Johnson PI Sutton P Atchley DS . The Navigation Guide - evidence-based medicine meets environmental health: systematic review of human evidence for PFOA effects on fetal growth. Environ Health Perspect. 2014;122:1028–1039. 24968388 10.1289/ehp.1307893 PMC4181929

[15] Koustas E Lam J Sutton P . The Navigation Guide—evidence-based medicine meets environmental health: systematic review of nonhuman evidence for PFOA effects on fetal growth. Environ Health Perspect. 2014;122:1015–1027. 24968374 10.1289/ehp.1307177 PMC4181920

[16] Morgan RL Thayer KA Bero L . GRADE: assessing the quality of evidence in environmental and occupational health. Environ Int. 2016;92-93:611–616. 26827182 10.1016/j.envint.2016.01.004 PMC4902742

[17] Piggott T Leontiadis GI Herrmann A . We’re living through a planetary health crisis: health guidelines must consider planetary health. Lancet Planetary Health. 2024;8:e979–e980. 39674202 10.1016/S2542-5196(24)00300-0

[18] Brouwers MC Kho ME Browman GP . AGREE II: advancing guideline development, reporting and evaluation in health care. Cmaj. 2010;182:E839–E842. 20603348 10.1503/cmaj.090449 PMC3001530

[19] Higgins JPTTJ Chandler J Cumpston M . Cochrane Handbook for Systematic Reviews of Interventions version 64. Cochrane; 2023. Accessed June 10, 2024. www.training.cochrane.org/handbook

[20] CLARITY Group at McMaster University . Tool to Assess Risk of Bias in Cohort Studies. Accessed July 26, 2024. https://www.distillersr.com/resources/methodological-resources/tool-to-assess-risk-of-bias-in-cohort-studies-distillersr

[21] CLARITY Group at McMaster University . Tool to Assess Risk of Bias in Randomized Controlled Trials. Accessed July 26, 2024. https://www.distillersr.com/resources/methodological-resources/tool-to-assess-risk-of-bias-in-randomized-controlled-trials-distillersr

[22] Balshem H Helfand M Schünemann HJ . GRADE guidelines: 3. Rating the quality of evidence. J Clin Epidemiol. 2011;64:401–406. 21208779 10.1016/j.jclinepi.2010.07.015

[23] Papadopoulou A Kumar NS Vanhoestenberghe A . Environmental sustainability in robotic and laparoscopic surgery: systematic review. Br J Surg. 2022;109:921–932. 35726503 10.1093/bjs/znac191

[24] Power NE Silberstein JL Ghoneim TP . Environmental impact of minimally invasive surgery in the United States: an estimate of the carbon dioxide footprint. J Endourol. 2012;26:1639–1644. 22845049 10.1089/end.2012.0298 PMC3521130

[25] Thiel CL Eckelman M Guido R . Environmental impacts of surgical procedures: life cycle assessment of hysterectomy in the United States. Environ Sci Technol. 2015;49:1779–1786. 25517602 10.1021/es504719g PMC4319686

[26] Woods DL McAndrew T Nevadunsky N . Carbon footprint of robotically-assisted laparoscopy, laparoscopy and laparotomy: a comparison. Int J Med Robot. 2015;11:406–412. 25708320 10.1002/rcs.1640

[27] Almutairi W Saget S Mc Donnell J . The planetary health effects of COVID-19 in dental care: a life cycle assessment approach. Br Dent J. 2022;233:309–316. 36028696 10.1038/s41415-022-4906-2 PMC9412817

[28] Boberg L Singh J Montgomery A . Environmental impact of single-use, reusable, and mixed trocar systems used for laparoscopic cholecystectomies. PLoS One. 2022;17:e0271601. 35839237 10.1371/journal.pone.0271601 PMC9286249

[29] Boucheron T Lechevallier E Gondran-Tellier B . Cost and environmental impact of disposable flexible cystoscopes compared to reusable devices. J Endourol. 2022;36:1317–1321. 35703325 10.1089/end.2022.0201

[30] Davis NF McGrath S Quinlan M . Carbon footprint in flexible ureteroscopy: a comparative study on the environmental impact of reusable and single-use ureteroscopes. J Endourol. 2018;32:214–217. 29373918 10.1089/end.2018.0001

[31] Donahue LM Hilton S Bell SG . A comparative carbon footprint analysis of disposable and reusable vaginal specula. Am J Obstet Gynecol. 2020;223:225 e1–e7. 10.1016/j.ajog.2020.02.007 32067971

[32] Eckelman M Mosher M Gonzalez A . Comparative life cycle assessment of disposable and reusable laryngeal mask airways. Anesth Analg. 2012;114:1067–1072. 22492190 10.1213/ANE.0b013e31824f6959

[33] Friedericy HJ van Egmond CW Vogtländer JG . Reducing the environmental impact of sterilization packaging for surgical instruments in the operating room: a comparative life cycle assessment of disposable versus reusable systems. Sustainability. 2022;14:430.

[34] Grimmond T Reiner S . Impact on carbon footprint: a life cycle assessment of disposable versus reusable sharps containers in a large US hospital. Waste Manag Res. 2012;30:639–642. 22627643 10.1177/0734242X12450602

[35] Grimmond TR Bright A Cadman J . Before/after intervention study to determine impact on life-cycle carbon footprint of converting from single-use to reusable sharps containers in 40 UK NHS trusts. BMJ Open. 2021;11:e046200. 10.1136/bmjopen-2020-046200 PMC8477330 34580089

[36] Hicks AL Reed RB Theis TL . Environmental impacts of reusable nanoscale silver-coated hospital gowns compared to single-use, disposable gowns. Environ Sci Nano. 2016;3:1124–1132.

[37] Hogan D Rauf H Kinnear N . The carbon footprint of single-use flexible cystoscopes compared with reusable cystoscopes. J Endourol. 2022;36:1460–1464. 35607858 10.1089/end.2021.0891

[38] Ibbotson S Dettmer T Kara S . Eco-efficiency of disposable and reusable surgical instruments—a scissors case. Int J Life Cycle Assess. 2013;18:1137–1148.

[39] Kemble JP Winoker JS Patel SH . Environmental impact of single-use and reusable flexible cystoscopes. BJU Int. 2023;131:617–622. 36515438 10.1111/bju.15949

[40] Le NNT Hernandez LV Vakil N . Environmental and health outcomes of single-use versus reusable duodenoscopes. Gastrointest Endosc. 2022;96:1002–1008. 35718068 10.1016/j.gie.2022.06.014

[41] Leiden A Cerdas F Noriega D . Life cycle assessment of a disposable and a reusable surgery instrument set for spinal fusion surgeries. Resour Conserv Recy. 2020;156:104704.

[42] McGain F McAlister S McGavin A . The financial and environmental costs of reusable and single-use plastic anaesthetic drug trays. Anaesth Intensive Care. 2010;38:538–544. 20514965 10.1177/0310057X1003800320

[43] McGain F McAlister S McGavin A . A life cycle assessment of reusable and single-use central venous catheter insertion kits. Anesth Analg. 2012;114:1073–1080. 22492185 10.1213/ANE.0b013e31824e9b69

[44] McGain F Story D Lim T . Financial and environmental costs of reusable and single-use anaesthetic equipment. Br J Anaesth. 2017;118:862–869. 28505289 10.1093/bja/aex098

[45] McPherson B Sharip M Grimmond T . The impact on life cycle carbon footprint of converting from disposable to reusable sharps containers in a large US hospital geographically distant from manufacturing and processing facilities. PeerJ. 2019;7:e6204. 30809428 10.7717/peerj.6204 PMC6388662

[46] Rodriguez Morris MI Hicks A . Life cycle assessment of stainless-steel reusable speculums versus disposable acrylic speculums in a university clinic setting: a case study. Environ Res Commun. 2022;4:025002.

[47] Namburar S von Renteln D Damianos J . Estimating the environmental impact of disposable endoscopic equipment and endoscopes. Gut. 2022;71:1326–1331. 34853058 10.1136/gutjnl-2021-324729

[48] Rizan C Bhutta MF . Environmental impact and life cycle financial cost of hybrid (reusable/single-use) instruments versus single-use equivalents in laparoscopic cholecystectomy. Surg Endosc. 2022;36:4067–4078. 34559257 10.1007/s00464-021-08728-z PMC9085686

[49] Sanchez SA Eckelman MJ Sherman JD . Environmental and economic comparison of reusable and disposable blood pressure cuffs in multiple clinical settings. Resour Conserv Recy. 2020;155:104643.

[50] Sherman JD Raibley LAT Eckelman MJ . Life cycle assessment and costing methods for device procurement: comparing reusable and single-use disposable laryngoscopes. Anesth Analg. 2018;127:434–443. 29324492 10.1213/ANE.0000000000002683

[51] Sørensen BL Larsen S Andersen C . A review of environmental and economic aspects of medical devices, illustrated with a comparative study of double-lumen tubes used for one-lung ventilation. Environ Develop Sustain. 2023;25:13219–13252.

[52] Unger SR Landis AE . Comparative life cycle assessment of reused versus disposable dental burs. Int J Life Cycle Assess. 2014;19:1623–1631.

[53] Vozzola E Overcash M Griffing E . Environmental considerations in the selection of isolation gowns: a life cycle assessment of reusable and disposable alternatives. Am J Infect Control. 2018;46:881–886. 29655666 10.1016/j.ajic.2018.02.002

[54] Vozzola E Overcash M Griffing E . An environmental analysis of reusable and disposable surgical gowns. AORN J. 2020;111:315–325. 32128776 10.1002/aorn.12885

[55] Nowack M Hoppe H Guenther E . Review and downscaling of life cycle decision support tools for the procurement of low-value products. Int J Life Cycle Assess. 2012;17:655–665.

[56] Hu X Pierce JMT Taylor T . The carbon footprint of general anaesthetics: a case study in the UK. Resour Conserv Recy. 2021;167:105411.

[57] McGain F Sheridan N Wickramarachchi K . Carbon footprint of general, regional, and combined anesthesia for total knee replacements. Anesthesiology. 2021;135:976–991. 34529033 10.1097/ALN.0000000000003967

[58] Sherman J Le C Lamers V . Life cycle greenhouse gas emissions of anesthetic drugs. Anesth Analg. 2012;114:1086–1090. 22492186 10.1213/ANE.0b013e31824f6940

[59] Thiel CL Woods NC Bilec MM . Strategies to reduce greenhouse gas emissions from laparoscopic surgery. Am J Public Health. 2018;108:S158–s64. 29698098 10.2105/AJPH.2018.304397 PMC5922216

[60] Wang AY Ahsan T Kosarchuk JJ . Assessing the environmental carbon footprint of spinal versus general anesthesia in single-level transforaminal lumbar interbody fusions. World Neurosurg. 2022;163:e199–e206. 35342029 10.1016/j.wneu.2022.03.095

[61] Alsved M Civilis A Ekolind P . Temperature-controlled airflow ventilation in operating rooms compared with laminar airflow and turbulent mixed airflow. J Hosp Infect. 2018;98:181–190. 29074054 10.1016/j.jhin.2017.10.013

[62] Marsault LV Ravn C Overgaard A . Laminar airflow versus turbulent airflow in simulated total hip arthroplasty: measurements of colony-forming units, particles, and energy consumption. J Hosp Infect. 2021;115:117–123. 34182062 10.1016/j.jhin.2021.06.009

[63] Cramer J . Milieu: Elementaire Deeltjes. 16 ed. Singel Uitgeverijen; 2014.

[64] Bijleveld M Uijttewaal M LCA herbruikbare en eenmalige ok-jassen en afdekmateriaal. *CE Delft* 2022. Accessed August 18, 2024. https://ce.nl/wp-content/uploads/2022/05/CE_Delft_210440_LCA_herbruikbare_en_eenmalige_ok-jassen_en_afdekmateriaal_def.pdf

[65] Dutch Society for Medical Microbiology (NVMM) . Guideline ‘Air treatment in operating rooms and treatment rooms (Luchtbehandeling in operatiekamers en behandelkamers)’. *Richtlijnendatabase* 2022. Accessed August 25, 2024. https://richtlijnendatabase.nl/richtlijn/luchtbehandeling_in_operatiekamers_en_behandelkamers/startpagina_-_luchtbehandeling_in_operatiekamers_en_behandelkamers.html

[66] Harris H Bhutta MF Rizan C . A survey of UK and Irish surgeons’ attitudes, behaviours and barriers to change for environmental sustainability. Ann R Coll Surg Engl. 2021;103:725–729. 34719956 10.1308/rcsann.2021.0271 PMC10335270

[67] van Nieuwenhuizen KE Both I Porte PJ . Environmental sustainability and gynaecological surgery: which factors influence behaviour? An interview study. BJOG. 2024;131:716–724. 37973607 10.1111/1471-0528.17709

[68] Dutch Collaborative Partnership for Infection Prevention Guidelines (Samenwerkingsverband Richtlijnen Infectiepreventie [SRI])/ Dutch Society for Surgery (Nederlandse Vereniging voor Heelkunde [NVvH]). Guideline ‘Prevention of postoperative wound infections (Preventie van postoperatieve wondinfecties)’ [Internet]. *Richtlijnendatabase 2024*. Accessed March 10, 2025. https://richtlijnendatabase.nl/richtlijn/preventie_van_postoperatieve_wondinfecties/startpagina_-_preventie_van_postoperatieve_wondinfecties.html

[69] Dutch Collaborative Partnership for Infection Prevention Guidelines (Samenwerkingsverband Richtlijnen Infectiepreventie [SRI]). Guideline ‘Infection prevention in the OR (Infectiepreventie op het OK complex)’ *2024*. Accessed March 10, 2025. https://www.sri-richtlijnen.nl/infectiepreventie-ok/

[70] Hinterberg J Beffart T Gabriel A . Efficiency of inhaled anaesthetic recapture in clinical practice. Br J Anaesth. 2022;129:e79–e81. 35589427 10.1016/j.bja.2022.04.009

[71] Traversari AA Bottenheft C van Heumen SP . Effect of switching off unidirectional downflow systems of operating theaters during prolonged inactivity on the period before the operating theater can safely be used. Am J Infect Control. 2017;45:139–144. 27742147 10.1016/j.ajic.2016.07.019

[72] Lans JLA Mathijssen NMC Bode A . What is the effect of reducing the air change rate on the ventilation effectiveness in ultra-clean operating rooms? J Hosp Infect. 2024;147:115–122. 38423130 10.1016/j.jhin.2024.02.007

[73] Keil M Frehse L Hagemeister M . Carbon footprint of healthcare systems: a systematic review of evidence and methods. BMJ Open. 2024;14:e078464. 10.1136/bmjopen-2023-078464 PMC11086491 38688670

