# Supplementary material for: Evidence-based Integration of Environmental Sustainability into Clinical Guidelines for Operating Rooms: A Systematic Review
Source: Ann Surg. 2025 May 21;283(4):593–607. doi: 10.1097/SLA.0000000000006756 (PMC12978700; doi:10.1097/SLA.0000000000006756)
Supplement: Supplementary file 1 [file sla-283-593-s001.docx]

*Supplementary materials*

Evidence-based integration of environmental sustainability into clinical guidelines for operating rooms: a systematic review

**Authors**Kim E. van Nieuwenhuizen, MD^1^; Charlotte T.J. Michels, PhD^2^; Ingena G.I.A. Both^1^, Prof. Jeroen B. Guinée^3^, Prof. Frank Willem Jansen, MD^1, 4^; on behalf of the Dutch Expert Panel

**Affiliations**
^1^Department of Obstetrics and Gynaecology, Leiden University Medical Centre, Leiden, The Netherlands
^2^Knowledge Institute Of The Dutch Association Of Medical Specialists, Utrecht, The Netherlands
^3^Institute of Environmental Sciences (CML), Department of Industrial Ecology, Leiden University, Leiden, The Netherlands
^4^Department of Biomedical Engineering, Technical University (TU) Delft, Delft, The Netherlands

Contents

[Appendix 1. Dutch Expert panel 3](#_Toc194997700)

[Appendix 2. Detailed methods 4](#_Toc194997701)

[Topic 1: Surgical techniques 4](#_Toc194997702)

[Topic 2: Disposable vs. reusable medical devices 5](#_Toc194997703)

[Topic 3: (Cover) materials in contact with the patient and operating table 6](#_Toc194997704)

[Topic 4: Anaesthesia 8](#_Toc194997705)

[Topic 5: Operating room air ventilation and air handling systems 9](#_Toc194997706)

[Appendix 3. Search strategy 11](#_Toc194997707)

[Topic 1: Surgical techniques 11](#_Toc194997708)

[Topic 2: Disposable vs. reusable medical devices (PICO1) 16](#_Toc194997709)

[Topic 2: Disposables vs. reusables (PICO2) 26](#_Toc194997710)

[Topic 3: (Cover) materials 32](#_Toc194997711)

[Topic 4: Anaesthesia 37](#_Toc194997712)

[Topic 5: Operating room air ventilation 58](#_Toc194997713)

[Appendix 4. Evidence tables 66](#_Toc194997714)

[Table 1a: Study characteristics LCA studies 66](#_Toc194997715)

[Table 1b: Methods LCA studies 76](#_Toc194997716)

[Table 1c: Methods LCA studies 82](#_Toc194997717)

[Table 1d: Data LCA studies 86](#_Toc194997718)

[Table 1e: Results LCA studies 92](#_Toc194997719)

[Table 1f: Comments LCA studies 119](#_Toc194997720)

[Table 2a: Systematic reviews evidence table 123](#_Toc194997721)

[Table 2b: Systematic reviews evidence table 124](#_Toc194997722)

[Table 2c: Systematic reviews evidence table 125](#_Toc194997723)

[Appendix 5. Critical appraisal and GRADE 129](#_Toc194997724)

[Table 3: Risk of bias table for intervention studies 129](#_Toc194997725)

[Table 4: Table of quality assessment for systematic reviews of RCTs/LCAs and observational studies 130](#_Toc194997726)

[Table 5a: Critical appraisal all studies (Drew et al. 2021) 132](#_Toc194997727)

[Table 5b: Critical appraisal all studies (Drew et al. 2021) 133](#_Toc194997728)

[Table 5c: Critical appraisal all studies (Drew et al. 2021) 135](#_Toc194997729)

[Table 5d: Critical appraisal all studies (Drew et al. 2021) 137](#_Toc194997730)

[Table 6: GRADE assessment for LCA studies 139](#_Toc194997731)

[Table 7: Topic 1. Critical appraisal points per study 140](#_Toc194997732)

[Table 8: Topic 1. GRADE assessment per included outcome 142](#_Toc194997733)

[Table 9: Topic 2. Critical appraisal points per study 146](#_Toc194997734)

[Table 10: Topic 2. GRADE assessment per included outcome 171](#_Toc194997735)

[Table 11: Topic 4. Critical appraisal points per study 175](#_Toc194997736)

[Table 12: Topic 4. GRADE assessment per included outcome 180](#_Toc194997737)

[Table 13: Topic 5. Critical appraisal 185](#_Toc194997738)

[Table 14: Topic 5. GRADE assessment per included outcome 186](#_Toc194997739)

## Appendix 1. Dutch Expert panel

| Prof. F.W. Jansen | Gynaecologist | Dutch Society of Obstetrics and Gynaecology |
| --- | --- | --- |
| Prof. N.D. Bouvy | Surgeon | Dutch Society for Surgery |
| I.R. van den Berg | Urologist | Dutch Society for Urology |
| P.W. van Egmond | Orthopedic Surgeon | Dutch Orthopaedic Society |
| R.J.H. Ensink, PhD | Ear, Nose and Throat (ENT) Surgeon | Dutch Society of ENT Surgery |
| N. de Haas (from January 2022) | Plastic Surgeon | Dutch Society of Plastic Surgery |
| A. Kwee, PhD | Gynaecologist | Dutch Society of Obstetrics and Gynaecology |
| N.C. Naus-Postema, PhD | Ophthalmologist | Ophthalmologic Society of the Netherlands |
| K.E. van Nieuwenhuizen | Medical Doctor, PhD candidate | Leiden University Medical Centre, Leiden, The Netherlands |
| N.A. Noordzij (until December 2021) | Plastic Surgeon | Dutch Society of Plastic Surgery |
| C.S. Sie | Anaesthesiologist | Dutch Society of Anaesthesiologists |
| E.S. Smits | Plastic Surgeon | Dutch Society of Plastic Surgery |
| K.E. Veldkamp, PhD | Medical Microbiologist | Dutch Society for Medical Microbiology |
| F.J.M. Westerlaken | Infection Prevention Specialist | Dutch Society of Infection Prevention in Healthcare |
| Prof. J.B. Guinée | Personal title | Institute of Environmental Sciences (CML), Leiden, Netherlands |
| P. de Heer | Advisor | National Healthcare Institute Netherlands |
| S.N. Hofstede | Senior Advisor | Knowledge Institute Of The Dutch Association Of Medical Specialists, Utrecht, The Netherlands |
| M.W. Langendam | Personal Title, Epidemiologist | Amsterdam University Medical Centre, Amsterdam, The Netherlands |
| J.H. van der Lee | Senior Advisor | Knowledge Institute Of The Dutch Association Of Medical Specialists, Utrecht, The Netherlands |
| C.T.J. Michels, PhD | Advisor | Knowledge Institute Of The Dutch Association Of Medical Specialists, Utrecht, The Netherlands |

## Appendix 2. Detailed methods

### Topic 1: Surgical techniques

**Identification of studies via databases and registers**

Records removed *before screening*:

Duplicate records removed (n = 162)

Records identified from:

Medline (PubMed) (n = 129)

Embase (OVID) (n = 130)

Web of Science (n = 85)

Cochrane Library (n = 19)

Emcare (OVID) (n = 48)

**Identification**

Abstracts screened

(n = 249)

Abstracts excluded

(n = 244)

Reports sought for retrieval

(n = 5)

Reports not retrieved

(n = 0)

**Screening**

Reports assessed for eligibility

(n = 4)

Reports excluded:

(n=0)

Studies included in review

(n =4)

**Included**

**Search and select**

A systematic review of the literature was performed to answer the following question: *What is the role of environmental sustainability of robot-assisted laparoscopic surgery compared with conventional laparoscopic surgery or open surgery?*

P: patients who underwent surgery

I: robot-assisted surgery

C: conventional laparoscopic surgery or open surgery

O: climate change, waste, acidification, eutrophication, human toxicity, ecotoxicity, ozone depletion

Search and select (Methods)

The databases Pubmed (via NCBI), Embase (via OVID), Web of Science (via Webofscience), Cochrane (via Cochrane library) and Emcare (via OVID) were searched with relevant search terms from 2000 until December 2022. Studies for this module were selected based on the following criteria:

- Systematic reviews in which searches were performed in at least two databases, with a detailed search strategy, risk of bias assessment and results of individual studies available, randomised controlled trials, (observational) comparative studies, Life Cycle Assessments;
- Full-text English or Dutch language publication; and
- Studies according to the PICO. This included studies that compared robot-assisted surgery with conventional laparoscopic or open surgery and included at least one of the outcomes conform the PICO.

### Topic 2: Disposable vs. reusable medical devices

**Identification of studies via databases and registers**

Records removed *before screening*:

Duplicate records removed (n = 525)

Records identified from*:

Medline (PubMed) (n = 404)

Embase (OVID) (n = 592)

Web of Science (n = 279)

Cochrane Library (n = 9)

Emcare (OVID) (n = 130)

**Identification**

Abstracts screened

(n = 889)

Abstracts excluded**

(n = 811)

Reports sought for retrieval

(n = 78)

Reports not retrieved

(n = 2)

**Screening**

Reports assessed for eligibility

(n = 76)

Reports excluded:

Wrong outcome (n = 4)

Wrong study design (n = 17)

Wrong comparison (n = 24)

Wrong setting (n = 1)

Studies included in review

(n = 30)

**Included**

* Pubmed (via NCBI), Embase (via OVID), Web of Science (via Webofscience), Cochrane (via Cochrane library) and Emcare (via OVID)

**Search and select**

A systematic review of the literature was performed to answer the following questions:

**PICO1:** *What is the difference in sustainability of reusables compared to disposables in the operating room for patients who undergo surgery?*

P = patients who undergo a surgical procedure

I = reusables, such as: surgical gowns, scrub caps, gloves, glasses, perioperative textiles (i.e. blue drapes, band aids), packing materials, or laryngeal masks

C = disposables, such as: surgical gowns, scrub caps, gloves, glasses, perioperative textiles (blue drapes, band aids), packing materials or laryngeal masks

O = climate change, waste, acidification, eutrophication, human toxicity, ecotoxicity, ozone depletion

**PICO2:** *What is the difference in sustainability of specific reusable medical instruments compared to disposable medical instruments in the operating room for patients who undergo surgery?*

P = patients who undergo a surgical procedure

I = reusable medical instruments, such as: specula, instruments, scopes

(e.g reusable instruments in a surgical tool kit: scissor, Kocher, tweezer, scalpel, needle driver, ligasure, harmonic, stapler, surgical drill; reusable scopes: duodenoscope, ureterorenoscope, bronchoscope, cystoscope, laryngeal scope; reusable meniscal sutures; reusable suture anchors).

C = disposable medical instruments, such as: specula, instruments, scopes

(e.g disposable instruments in a surgical tool kit: scissor, Kocher, tweezer, scalpel, needle driver, vessel sealer, stapler, surgical drill; disposable scopes: duodenoscope, ureterorenoscope, bronchoscope, cystoscope, laryngeal scope; disposable meniscal sutures; disposable suture anchors).

O = climate change (CO_2_ footprint/Global Warming Potential (GWP)), waste, acidification, eutrophication, human toxicity, ecotoxicity, ozone depletion

Search and select (Methods)

The databases Pubmed (via NCBI), Embase (via OVID), Web of Science (via Webofscience), Cochrane (via Cochrane library) and Emcare (via OVID) were searched with relevant search terms from 2000 until December 2022. Studies for this module were selected based on the following criteria:

- Systematic reviews (searched in at least two databases, with a detailed search strategy, risk of bias assessment and results of individual studies available), randomized controlled trials, (observational) comparative studies, life cycle assessments, CO_2_ footprint studies and environmental impact studies;
- Full-text English language publication; and
- Studies according to the PICO. Studies that compared disposables with reusables related to the OR and included at least one of the following outcomes: climate change, waste, acidification, eutrophication, human toxicity, ecotoxicity, ozone depletion.

### Topic 3: (Cover) materials in contact with the patient and operating table

**Identification of studies via databases and registers**

Records removed *before screening*:

Duplicate records removed

(n = 289)

Records identified from*:

Medline (PubMed) (n = 279)

Embase (OVID) (n = 303)

Web of Science (n = 128)

Cochrane Library (n = 36)

Emcare (OVID) (n = 94)

**Identification**

Abstracts screened

(n = 551)

Abstracts excluded**

(n = 540)

Reports sought for retrieval

(n = 11)

Reports not retrieved

(n = 0)

**Screening**

Reports assessed for eligibility

(n = 11)

Reports excluded:

Wrong outcome (n = 3)

Wrong study design (n = 3)

Wrong comparison (n = 4)

Studies included in review

(n = 1)

**Included**

* Pubmed (via NCBI), Embase (via OVID), Web of Science (via Webofscience), Cochrane (via Cochrane library) and Emcare (via OVID)

**Search and select**

A systematic review of the literature was performed to answer the following question: *What is the effect on environmental sustainability of disposable materials (i.e. heat blankets, surgical drapes, disposable duvets, cellulose pads) in comparison with alternative reusable or sustainable (e.g. biobased) materials that are in contact with patients on the operating table?*

P: Patients on the operating table

I: Reusable or sustainable (e.g. bio-based) alternative for heat blanket (bair hugger), surgical drapes, disposable duvet and cellulose pads

C: Use of disposable heat blanket (bair hugger), disposable surgical drapes, disposable duvet and disposable cellulose pads

O: Climate change (CO_2_ footprint/Global Warming Potential), waste, water use, land use, energy use

Search and select (Methods)

The databases Pubmed (via NCBI), Embase (via OVID), Web of Science (via Webofscience), Cochrane (via Cochrane library) and Emcare (via OVID) were searched with relevant search terms from 1980 until December 2022. Studies for this module were selected based on the following criteria:

- Systematic reviews in which searches were performed in at least two databases, with a detailed search strategy, risk of bias assessment and results of individual studies available, randomized controlled trials, (observational) comparative studies, Life Cycle Assessments;
- Full-text English or Dutch language publication; and
- Studies according to the PICO. This included studies that compared sustainable alternatives for heat blanket (bair hugger), surgical drapes, disposable duvet and cellulose pads compared with the use of standard heat blanket (bair hugger), surgical drapes, disposable duvet and cellulose pads, and included at least one of the outcomes conform the PICO.

.

### Topic 4: Anaesthesia

**Identification of studies via databases and registers**

Records removed *before screening*:

Duplicate records removed (n = 431)

Records identified from*:

Medline (PubMed) (n = 521)

Embase (OVID) (n = 407)

Web of Science (n = 120)

Cochrane Library (n = 88)

Emcare (OVID) (n = 209)

**Identification**

Abstracts screened

(n = 914)

Abstracts excluded**

(n = 886)

Reports sought for retrieval

(n = 28)

Reports not retrieved

(n = 0)

**Screening**

Reports assessed for eligibility

(n = 28)

Reports excluded:

Wrong outcome (n = 2)

Wrong study design (n = 11)

Wrong comparison (n = 10)

Studies included in review

(n = 5)

**Included**

* Pubmed (via NCBI), Embase (via OVID), Web of Science (via Webofscience), Cochrane (via Cochrane library) and Emcare (via OVID)

**Search and select**

A systematic review of the literature was performed to answer the following questions:

**PICO1:** *What is the effect on environmental sustainability of inhalation anaesthetics compared with the use of intravenous anaesthesia in patients undergoing surgery?*

P = patients undergoing a surgical procedure under general anaesthesia

I = inhalation anaesthetics

C = intravenous anaesthetics

O = climate change (CO_2_ footprint/GWP), waste, medicine residue in water, human toxicity, ozone depletion

**PICO2:** *What is the effect on environmental sustainability of inhalation anaesthetics while using Vapour Capture Technology compared with the use of inhalation anaesthetics while not using Vapour Capture Technology in patients undergoing surgery?*

P = patients undergoing a surgical procedure under anaesthesia

I = inhalation anaesthetics with use of Vapour Capture Technology

C = inhalation anaesthetics without use of Vapour Capture Technology

O = climate change (CO_2_ footprint/GWP), waste, medicine residue in water, human toxicity, ozone depletion

**PICO3:** *What is the effect on environmental sustainability of (loco)regional anaesthesia and local anaesthesia compared with the use of general anaesthesia in patients undergoing surgery?*

P = patients undergoing a surgical procedure under anaesthesia

I = (loco)regional anaesthesia and local anaesthesia

C = general anaesthesia

O = climate change (CO_2_ footprint/GWP), waste, medicine residue in water, human toxicity, ozone depletion

Search and select (Methods)

The databases Pubmed (via NCBI), Embase (via OVID), Web of Science (via Webofscience), Cochrane (via Cochrane library) and Emcare (via OVID) were searched with relevant search terms from 2000 until December 2022. Studies for this module were selected based on the following criteria:

- Systematic reviews (searched in at least two databases, with a detailed search strategy, risk of bias assessment and results of individual studies available), randomized controlled trials, (observational) comparative studies, life cycle assessments, CO_2_ footprint studies and environmental impact studies;
- Full-text English language publication; and
- Studies according to the PICO. Studies that compared different types of anaesthesia related and included at least one of the following outcomes conform the PICO.

### Topic 5: Operating room air ventilation and air handling systems

**Identification of studies via databases and registers**

Records removed *before screening*:

Duplicate records removed (n = 284)

Records identified from*:

Medline (PubMed) (n = 197)

Embase (OVID) (n = 243)

Web of Science (n = 94)

Cochrane Library (n = 42)

Emcare (OVID) (n = 102)

**Identification**

Abstracts screened

(n = 394)

Abstracts excluded**

(n = 387)

Reports sought for retrieval

(n = 7)

Reports not retrieved

(n = 1)

**Screening**

Reports assessed for eligibility

(n = 6)

Reports excluded

Wrong study design (n = 2)

Wrong comparison (n = 2)

Studies included in review

(n = 2)

**Included**

* Pubmed (via NCBI), Embase (via OVID), Web of Science (via Webofscience), Cochrane (via Cochrane library) and Emcare (via OVID)

**Search and select**

A systematic review of the literature was performed to answer the following questions:

**PICO1:** *What is the role of environmental sustainability outcomes regarding the different operating room air ventilation criteria (low (class 2), medium (class 1) or high (class 1+))^70^ during surgical procedures?*

P = surgical procedures

I = high, medium air ventilation criteria

C = low air ventilation criteria

O = climate change (CO_2_ footprint/Global Warming Potential), energy use

**Table S1. Operating room air ventilation criteria^70^**

| **Operating room** | **Class 1+ operating room** | **Class 1 operating room** | **Class 2 operating room** |
| --- | --- | --- | --- |
| **Air exchanges/hour** | At least 20x | At least 20x | At least 6x |
| **Air quality (ISO classes)^$^** | ISO 5 (NEN EN ISO 14644-1) | ISO 7 (NEN EN ISO 14644-1) | ISO 7 (NEN EN ISO 14644-1) |
| **Recovery time (1:100)** | ≤3 min | ≤20 min (NEN EN ISO 14644-3) | Not applicable |
| **Air filtration** | At least HEPA filter H13 (EN 1822) | At least HEPA filter H13 (EN 1822) | At least HEPA filter H13 (EN 1822) |
| **Temperature*** | 18°C – 23°C | 18°C – 23°C | 18°C – 23°C |
| **Relative humidity*** | < 65% | < 65% | < 65% |
| **Pressure hierarchy/airflow** | 3 zones with decreasing cleanliness (clearly indicated on construction plans) relative to the rest of the building | 3 zones with decreasing cleanliness (clearly indicated on construction plans) relative to the rest of the building | 2 zones with decreasing cleanliness (clearly indicated on construction plans) relative to the rest of the building |

^$^ ISO 7 applies to the entire operating room.

*The recommended temperatures and relative humidity levels are indicative.

**PICO2:** *What is the role of environmental sustainability outcomes across the entire life cycle of the a mixed air handling system in comparison to suppressing semi-suppressing air handling system in surgical procedures?*

P = surgical procedures

I = mixed air handling system (e.g. inlet grilles)

C = suppressing or semi-suppressing air handling system (e.g. unidirectional laminar downflow/plenum, opragon, halton)

O = climate change (CO_2_ footprint/Global Warming Potential), energy use

Search and select (Methods)

The databases Pubmed (via NCBI), Embase (via OVID), Web of Science (via Webofscience), Cochrane (via Cochrane library) and Emcare (via OVID) were searched with relevant search terms from 1980 until December 2022. Studies for this module were selected based on the following criteria:

- Systematic reviews in which searches were performed in at least two databases, with a detailed search strategy, risk of bias assessment and results of individual studies available, randomised controlled trials, (observational) comparative studies, Life Cycle Assessments;
- Full-text English or Dutch language publication; and
- Studies according to the PICO. The included studies that compared different air treatment systems and included at least one of the following outcomes: Climate change (CO_2_ footprint/GWP) or energy use.

## Appendix 3. Search strategy

### Topic 1: Surgical techniques

**MEDLINE (PubMed)**

(("Robotic Surgical Procedures"[Mesh] OR "Robotic Surgical Procedures"[tw] OR "Robotic Surgical Procedure"[tw] OR "Robotic Surgical"[tw] OR "Robotic Surgical*"[tw] OR "Robotic Surgery"[tw] OR "Robotic Surg*"[tw] OR "Robot Surgical Procedures"[tw] OR "Robot Surgical Procedure"[tw] OR "Robot Surgical"[tw] OR "Robot Surgical*"[tw] OR "Robot Surgery"[tw] OR "Robot Surg*"[tw] OR "Robot Assisted Surg*"[tw] OR "Robot Assisted Surgery"[tw] OR "Robot Enhanced Procedure"[tw] OR "Robot Enhanced Procedures"[tw] OR "Robot Enhanced Surg*"[tw] OR "Robot Enhanced Surgery"[tw] OR "Robotic Assisted Surg*"[tw] OR "Robotic Assisted Surgery"[tw] OR "Robot Assisted Procedure"[tw] OR "Robot Assisted Procedures"[tw] OR "Robotic Assisted Procedure"[tw] OR "Robotic Assisted Procedures"[tw] OR "Da Vinci robot"[tw] OR "Da Vinci robots"[tw] OR "Da Vinci robotic"[tw] OR "Da Vinci robotics"[tw] OR "Da Vinci robot*"[tw] OR "Surgery robot"[tw] OR "Surgery robots"[tw] OR "Surgery robotic"[tw] OR "Surgery robotics"[tw] OR "Surgery robot*"[tw] OR "Surgical robot"[tw] OR "Surgical robots"[tw] OR "Surgical robotic"[tw] OR "Surgical robotics"[tw] OR "Surgical robot*"[tw] OR "Da Vinci"[tw] OR (("Surgical Procedures Operative"[Mesh] OR "surgery"[Subheading] OR "surgery"[tw] OR "surgical"[tw] OR "surgical*"[tw] OR "Surgeons"[mesh] OR "surgeon"[tw] OR "surgeons"[tw] OR "surgeon*"[tw] OR "neurosurgery"[tw] OR "neurosurgical"[tw] OR "neurosurgical*"[tw] OR "neurosurgeon"[tw] OR "neurosurgeons"[tw] OR "neurosurgeon*"[tw] OR "radiosurgery"[tw] OR "radiosurgical"[tw] OR "radiosurgical*"[tw] OR "radiosurgeon"[tw] OR "radiosurgeons"[tw] OR "radiosurgeon*"[tw]) AND ("Robotics"[Mesh] OR "Robotics"[tw] OR "Robot"[tw] OR "Robots"[tw] OR "Robotic"[tw] OR "Robot*"[tw] OR "Telerobotics"[tw] OR "Telerobot*"[tw]))) AND ("Laparoscopy"[Mesh] OR "Laparoscopy"[tw] OR "Laparoscop*"[tw] OR "Laparotomy"[Mesh] OR "Laparotomy"[tw] OR "Laparotom*"[tw] OR "Minimally Invasive Surgical Procedures"[Mesh] OR "Minimally Invasive Surgery"[tw] OR "Minimally Invasive"[tw] OR "Minimal Surgical"[tw] OR "Minimal Access"[tw] OR "open surgery"[tw] OR ("Surgical Procedures Operative"[Mesh] NOT "Robotic Surgical Procedures"[Mesh]) OR "surgery"[Subheading] OR "conventional surgery"[tw] OR "Endoscopy"[Mesh] OR "Endoscopy"[tw] OR "endoscop*"[tw]) AND ("Acidification potential"[tw] OR "acidification"[tw] OR "air pollution control"[tw] OR "AP in kg SO2 equivalents"[tw] OR "Biodiversity"[Mesh] OR "Biodiversity"[tw] OR "Carbon Footprint"[mesh] OR "carbon footprint"[tw] OR "carbon footprint*"[tw] OR "CFC-11 equiv*"[tw] OR "Climate Change"[Mesh] OR "climate change"[tw] OR "Climatic change"[tw] OR "CO2 emission"[tw] OR "CO2 emissions"[tw] OR "CO2 equiva*"[tw] OR "CO2 footprint"[tw] OR "CO2 footprint*"[tw] OR "conservation of natural resources"[mesh] OR "conservation of natural resources"[tw] OR "Disposable Equipment"[Mesh] OR "Disposable"[tw] OR "Disposables"[tw] OR "eco toxic*"[tw] OR "eco toxicity"[tw] OR "ecoefficien*"[tw] OR "eco-efficien*"[tw] OR "ecoefficiency"[tw] OR "eco-efficiency"[tw] OR "ecological footprint"[tw] OR "ecological footprint*"[tw] OR "ecological sustainability"[tw] OR "ecotoxic*"[tw] OR "ecotoxicity"[tw] OR "Emission reduction strategy"[tw] OR "Emission reduction"[tw] OR **"Environment"[Mesh:noexp]** OR "environmental impact"[tw] OR "environmental impact*"[tw] OR "environmental impacts"[tw] OR "environmental pollut*"[tw] OR "Environmental Pollution"[Mesh] OR "environmental pollution"[tw] OR "environmental protection"[tw] OR "environmental sustainab*"[tw] OR "environmental sustainability"[tw] OR **"Environmental*"[ti]** OR "EP in kg PO4 equivalent"[tw] OR "Equipment reuse"[mesh] OR "Equipment reuse"[tw] OR "Eutrophication potential"[tw] OR "Eutrophication"[Mesh] OR "eutrophication"[tw] OR "FAETP in kg DCB equivalent"[tw] OR "Freshwater Aquatic Ecotoxicity Potential"[tw] OR "Global Warming"[mesh] OR "Global Warming"[tw] OR "Green deal"[tw] OR "Green surgery"[tw] OR "Greenhouse Effect"[mesh] OR "greenhouse effect*"[tw] OR "greenhouse effects"[tw] OR "greenhouse gas emission"[tw] OR "greenhouse gas emissions"[tw] OR "Greenhouse Gas"[tw] OR "Greenhouse Gases"[mesh] OR "Greenhouse Gases"[tw] OR "greening"[tw] OR "GWP in kg CO2 equivalents"[tw] OR "H+ moles equivalents"[tw] OR "hospital waste"[tw] OR "HTTP in kg Dichlorobenzene equivalent"[tw] OR "Human Toxicity Potential"[tw] OR "kg 2.4-D equivalents"[tw] OR "kg CFC-11 equivalent"[tw] OR "kg N equivalents"[tw] OR "kg NOx equivalents"[tw] OR "LCA"[tw] OR "LCAs"[tw] OR "life cycle analysis"[tw] OR "life cycle assess*"[tw] OR "life cycle assessment"[tw] OR "life cycle inventories"[tw] OR "life cycle inventory"[tw] OR "Medical Waste Disposal"[mesh] OR "Medical Waste"[mesh] OR "medical waste"[tw] OR "N equiv*"[tw] OR "Ozone Depletion"[Mesh] OR "ozone depletion"[tw] OR "Photochemical Ozone Depletion Potential"[tw] OR "Plastic overuse"[tw] OR "POCP in kg ethane equivalent"[tw] OR "preservation of natural resources"[tw] OR "recycle*"[tw] OR "Recycling"[mesh] OR "recycling"[tw] OR "Refuse Disposal"[Mesh] OR "Refuse Disposal"[tw] OR "reusable"[tw] OR "Reusables"[tw] OR "reuse"[tw] OR "reused"[tw] OR "reusing"[tw] OR "Rising Sea Level"[tw] OR "Rising Sea Levels"[tw] OR "Sea Level Rise"[mesh] OR "Sea Level Rise"[tw] OR "Smog"[mesh] OR "smog"[tw] OR "SO2 equiv*"[tw] OR "sustainability"**[ti]** OR "Sustainable Development"[Mesh] OR "Sustainable Development"[tw] OR "Waste Disposal"[tw] OR "Waste Disposal, Fluid"[mesh] OR "Waste Management"[mesh] OR "Waste"[tw] OR "waste"[tw] OR "Waste Water"[Mesh] OR "Waste Water"[tw] OR "wastes"[tw] OR "Wastewater"[tw] OR "Water Purification"[Mesh] OR "Water Purification"[tw] OR (("plastic*"[tw] OR "microplastic*"[tw]) AND ("soop"[tw] OR "soup"[tw] OR "pollution"[tw] OR "overuse"[tw] OR "contamination"[tw])) OR (("Plastic"[tw] OR "plastics"[tw]) AND "overuse"[tw]) OR ("hydrogen*"[tw] AND "moles"[tw] AND "equiv*"[tw]) OR ("Dichlorobenzen*"[tw] AND "equiv*"[tw]) OR ("2,4-D"[tw] AND "equiv*"[tw]) OR ("NOx"[tw] AND "equiv*"[tw]) OR ("ethane"[tw] AND "equiv*"[tw]) OR ("PO4"[tw] AND "equiv*"[tw]) OR ("DCB"[tw] AND "equiv*"[tw]) OR ("sustainability"[tw] AND ("environment*"[tw] OR "carbon"[tw])) OR (("Carbon Dioxide"[mesh] OR "Carbon Dioxide"[tw] OR "CO2"[tw]) AND ("pollution"[tw] OR "emission"[tw] OR "emissions"[tw] OR "waste"[tw] OR "environment"[tw] OR "environmental*"[tw] OR "footprint"[tw] OR "footprint*"[tw] OR "sustainable"[tw] OR "hazard"[tw] OR "hazard*"[tw]))) AND ("2000/01/01"[PDAT] : "3000/12/31"[PDAT])) AND **(***("Meta-Analysis"[Publication Type] OR "Meta-Analysis as Topic"[Mesh] OR metaanaly*[tiab] OR meta-analy*[tiab] or metanaly*[tiab] OR "Systematic Review"[Publication Type] OR systematic[sb] OR "Cochrane Database Syst Rev"[Journal] or prisma[tiab] OR preferred reporting items[tiab] OR prospero[tiab] OR ((systemati*[ti] OR scoping[ti] OR umbrella[ti] OR structured literature[ti]) AND (review*[ti] OR overview*[ti])) OR systematic review*[tiab] OR scoping review*[tiab] OR umbrella review*[tiab] OR structured literature review*[tiab] OR systematic qualitative review*[tiab] OR systematic quantitative review*[tiab] OR systematic search and review[tiab] OR systematized review[tiab] OR systematised review[tiab] OR systemic review[tiab] OR systematic literature review*[tiab] OR systematic integrative literature review*[tiab] OR systematically review*[tiab] OR scoping literature review*[tiab] OR systematic critical review[tiab] OR systematic integrative review*[tiab] OR systematic evidence review[tiab] OR Systematic integrative literature review*[tiab] OR Systematic mixed studies review*[tiab] OR Systematized literature review*[tiab] OR Systematic overview*[tiab] OR Systematic narrative review*[tiab] OR ((systemati*[tiab] OR literature[tiab] OR database*[tiab] OR data-base*[tiab] OR structured[tiab] OR comprehensive*[tiab] OR systemic*[tiab]) AND search*[tiab]) OR (Literature[ti] AND review[ti] AND (database*[tiab] OR data-base*[tiab] OR search*[tiab])) OR ((data extraction[tiab] OR data source*[tiab]) AND study selection[tiab]) OR (search strategy[tiab] AND selection criteria[tiab]) OR (data source*[tiab] AND data synthesis[tiab]) OR medline[tiab] OR pubmed[tiab] OR embase[tiab] OR Cochrane[tiab] OR ((critical[ti] OR rapid[ti]) AND (review*[ti] OR overview*[ti] OR synthes*[ti])) OR (((critical*[tiab] OR rapid*[tiab]) AND (review*[tiab] OR overview*[tiab] OR synthes*[tiab]) AND (search*[tiab] OR database*[tiab] OR data-base*[tiab]))) OR metasynthes*[tiab] OR meta-synthes*[tiab])* **OR** ("Randomized Controlled Trial"[Publication Type] OR random*[tiab] OR pragmatic clinical trial*[tiab] OR practical clinical trial*[tiab] OR non-inferiority trial*[tiab] OR noninferiority trial*[tiab] OR superiority trial*[tiab] OR equivalence clinical trial*[tiab]) NOT (("Animals"[Mesh]) OR "Models, Animal"[Mesh] NOT humans[mh]) NOT (letter[pt] OR comment[pt] OR editorial[pt]) **OR ("Comparative Study"[Publication Type] OR "comparison"[tiab] OR "comparative"[tiab] OR "compar*"[tiab] OR "Epidemiologic studies"[mesh:noexp] OR "case control studies"[mesh] OR "cohort studies"[mesh] OR "Controlled Before-After Studies"[mesh] OR "Case control"[tw] OR cohort*[tw] OR "Cohort analy*"[tw] OR "Follow up stud*"[tw] OR "observational stud*"[tw] OR Longitudinal[tw] OR Retrospective*[tw] OR prospective*[tw] OR consecutive*[tw] OR Cross sectional[tw] OR "Cross-sectional studies"[mesh] OR "historically controlled study"[mesh] OR "interrupted time series analysis"[mesh])** OR ("life cycle assess*"[tw] OR "life cycle assessment"[tw] OR "life cycle inventory"[tw] OR "LCA"[tw] OR "LCAs"[tw] OR "life cycle inventory"[tw] OR "life cycle inventories"[tw])**)**

**Embase (OVID)**

((exp "Robot Assisted Surgery"/ OR exp "robotic surgical device"/ OR "Robotic Surgical Procedures".mp OR "Robotic Surgical Procedure".mp OR "Robotic Surgical".mp OR "Robotic Surgical*".mp OR "Robotic Surgery".mp OR "Robotic Surg*".mp OR "Robot Surgical Procedures".mp OR "Robot Surgical Procedure".mp OR "Robot Surgical".mp OR "Robot Surgical*".mp OR "Robot Surgery".mp OR "Robot Surg*".mp OR "Robot Assisted Surg*".mp OR "Robot Assisted Surgery".mp OR "Robot Enhanced Procedure".mp OR "Robot Enhanced Procedures".mp OR "Robot Enhanced Surg*".mp OR "Robot Enhanced Surgery".mp OR "Robotic Assisted Surg*".mp OR "Robotic Assisted Surgery".mp OR "Robot Assisted Procedure".mp OR "Robot Assisted Procedures".mp OR "Robotic Assisted Procedure".mp OR "Robotic Assisted Procedures".mp OR "Da Vinci robot".mp OR "Da Vinci robots".mp OR "Da Vinci robotic".mp OR "Da Vinci robotics".mp OR "Da Vinci robot*".mp OR "Surgery robot".mp OR "Surgery robots".mp OR "Surgery robotic".mp OR "Surgery robotics".mp OR "Surgery robot*".mp OR "Surgical robot".mp OR "Surgical robots".mp OR "Surgical robotic".mp OR "Surgical robotics".mp OR "Surgical robot*".mp OR "Da Vinci".mp OR ((exp "Surgery"/ OR "surgery".fs OR "surgery".mp OR "surgical".mp OR "surgical*".mp OR exp "Surgeon"/ OR "surgeon".mp OR "surgeons".mp OR "surgeon*".mp OR "neurosurgery".mp OR "neurosurgical".mp OR "neurosurgical*".mp OR "neurosurgeon".mp OR "neurosurgeons".mp OR "neurosurgeon*".mp OR "radiosurgery".mp OR "radiosurgical".mp OR "radiosurgical*".mp OR "radiosurgeon".mp OR "radiosurgeons".mp OR "radiosurgeon*".mp) AND ("Robotics"/ OR "Robotics".mp OR exp "Robot"/ OR exp "Medical Robot"/ OR "Robot".mp OR "Robots".mp OR "Robotic".mp OR "Robot*".mp OR "Telerobotics".mp OR "Telerobot*".mp))) AND (exp "Laparoscopy"/ OR "Laparoscopy".mp OR "Laparoscop*".mp OR "Laparotomy"/ OR "Laparotomy".mp OR "Laparotom*".mp OR "Minimally Invasive Surgery"/ OR "Minimally Invasive Surgery".mp OR "Minimally Invasive".mp OR "Minimal Surgical".mp OR "Minimal Access".mp OR "open surgery"/ OR "open surgery".mp OR (exp "Surgery"/ NOT (exp "Robot Assisted Surgery"/ OR exp "robotic surgical device"/)) OR "su".fs OR "conventional surgery".mp OR exp "Endoscopy"/ OR "Endoscopy".mp OR "endoscop*".mp) AND ("Carbon Footprint"/ OR "carbon footprint".mp OR "carbon footprint*".mp OR exp "Climate Change"/ OR "climate change".mp OR "CO2 emission".mp OR "CO2 emissions".mp OR "CO2 footprint".mp OR "CO2 footprint*".mp OR exp "environmental protection"/ OR "conservation of natural resources".mp OR "environmental protection".mp OR "Disposable Equipment"/ OR "Disposables".mp OR "Disposable".mp OR "ecological footprint".mp OR "ecological footprint*".mp OR "ecological sustainability".mp OR exp "environmental impact"/ OR "environmental impact".mp OR "environmental impact*".mp OR "environmental impacts".mp OR "environmental pollut*".mp OR exp "pollution"/ OR "environmental pollution".mp OR "environmental sustainab*".mp OR "environmental sustainability"/ OR "environmental sustainability".mp OR "Global Warming"/ OR "Global Warming".mp OR "Greenhouse Effect"/ OR "greenhouse effect*".mp OR "greenhouse effects".mp OR "greenhouse gas emission".mp OR "greenhouse gas emissions".mp OR "Greenhouse Gas"/ OR "greening".mp OR "hospital waste".mp OR "life cycle assessment"/ OR "environmental impact assessment"/ OR "life cycle assess*".mp OR "life cycle assessment".mp OR "LCA".mp OR "LCAs".mp OR "life cycle inventory".mp OR "life cycle inventories".mp OR exp "Waste Disposal"/ OR exp "Hospital Waste"/ OR "medical waste".mp OR "Rising Sea Level".mp OR "Rising Sea Levels".mp OR "Sea Level Rise"/ OR "Sea Level Rise".mp OR "sustainability"**.ti** OR "Waste Disposal".mp OR "waste water recycling"/ OR "Recycling"/ OR "recycling".mp OR "recycle*".mp OR "Equipment reuse".mp OR "Reusables".mp OR "reusable".mp OR "reuse".mp OR "reused".mp OR "reusing".mp OR exp "Waste Disposal"/ OR exp "Waste Management"/ OR "Plastic overuse".mp OR "Green surgery".mp OR "Emission reduction".mp OR "Emission reduction strategy".mp OR "air pollution control"/ OR "air pollution control".mp **OR "Environment"/ OR "Environmental*".ti** OR "acidification"/ OR "soil acidification"/ OR "ocean acidification"/ OR "acidification".mp OR "Acidification potential".mp OR "AP in kg SO2 equivalents".mp OR "eco-efficiency".mp OR "ecoefficiency".mp OR "eco-efficien*".mp OR "ecoefficien*".mp OR "ecotoxicity"/ OR "ecotoxicity".mp OR "ecotoxic*".mp OR "eco toxicity".mp OR "eco toxic*".mp OR "EP in kg PO4 equivalent".mp OR exp "Eutrophication"/ OR "eutrophication".mp OR "Eutrophication potential".mp OR "FAETP in kg DCB equivalent".mp OR "Freshwater Aquatic Ecotoxicity Potential".mp OR "GWP in kg CO2 equivalents".mp OR "H+ moles equivalents".mp OR "HTTP in kg Dichlorobenzene equivalent".mp OR "Human Toxicity Potential".mp OR "kg 2.4-D equivalents".mp OR "kg CFC-11 equivalent".mp OR "kg N equivalents".mp OR "kg NOx equivalents".mp OR "life cycle analysis".mp OR "ozone depletion".mp OR "Photochemical Ozone Depletion Potential".mp OR "POCP in kg ethane equivalent".mp OR "smog".mp OR exp "Waste"/ OR "waste".mp OR "wastes".mp OR "Ozone Depletion"/ OR "Smog"/ OR "Equipment reuse".mp OR "Greenhouse Gases".mp OR "Greenhouse Gas".mp OR "SO2 equiv*".mp OR "CO2 equiva*".mp OR "CFC-11 equiv*".mp OR "N equiv*".mp OR exp "Biodiversity"/ OR "Biodiversity".mp OR "Climatic change".mp OR "Green deal".mp OR "preservation of natural resources".mp OR "Refuse Disposal".mp OR exp "Wastewater"/ OR "Waste Water".mp OR "Wastewater".mp OR exp "Water Management"/ OR "Water Purification".mp OR (("plastic*".mp OR "microplastic*".mp) AND ("soop".mp OR "soup".mp OR "pollution".mp OR "overuse".mp OR "contamination".mp)) OR "Sustainable Development"/ OR "Sustainable Development".mp OR (("Plastic".mp OR "plastics".mp) AND "overuse".mp) OR ("hydrogen*".mp AND "moles".mp AND "equiv*".mp) OR ("Dichlorobenzen*".mp AND "equiv*".mp) OR ("2,4-D".mp AND "equiv*".mp) OR ("NOx".mp AND "equiv*".mp) OR ("ethane".mp AND "equiv*".mp) OR ("PO4".mp AND "equiv*".mp) OR ("DCB".mp AND "equiv*".mp) OR ("sustainability".mp AND ("environment*".mp OR "carbon".mp)) OR (("Carbon Dioxide"/ OR "Carbon Dioxide".mp OR "CO2".mp) AND ("pollution".mp OR "emission".mp OR "emissions".mp OR "waste".mp OR "environment".mp OR "environmental*".mp OR "footprint".mp OR "footprint*".mp OR "sustainable".mp OR "hazard".mp OR "hazard*".mp))) NOT (conference review or conference abstract).pt AND (2000 OR 2001 OR 2002 OR 2003 OR 2004 OR 2005 OR 2006 OR 2007 OR 2008 OR 2009 OR 2010 OR 2011 OR 2012 OR 2013 OR 2014 OR 2015 OR 2016 OR 2017 OR 2018 OR 2019 OR 2020 OR 2021 OR 2022).yr) AND **(***(exp "meta analysis"/ OR exp "meta analysis (topic)"/ OR metaanaly*.ti,ab OR "meta analy*".ti,ab OR metanaly*.ti,ab OR "systematic review"/ OR "cochrane database of systematic reviews".jn OR prisma.ti,ab OR prospero.ti,ab OR (((systemati* OR scoping OR umbrella OR "structured literature") ADJ3 (review* OR overview*)).ti,ab) OR ((systemic* ADJ1 review*).ti,ab) OR (((systemati* OR literature OR database* OR "data base*") ADJ10 search*).ti,ab) OR (((structured OR comprehensive* OR systemic*) ADJ3 search*).ti,ab) OR (((literature ADJ3 review*).ti,ab) AND (search*.ti,ab OR database*.ti,ab OR "data base*".ti,ab)) OR (("data extraction".ti,ab OR "data source*".ti,ab) AND "study selection".ti,ab) OR ("search strategy".ti,ab AND "selection criteria".ti,ab) OR ("data source*".ti,ab AND "data synthesis".ti,ab) OR medline.ab OR pubmed.ab OR embase.ab OR cochrane.ab OR (((critical OR rapid) ADJ2 (review* OR overview* OR synthes*)).ti) OR ((((critical* OR rapid*) ADJ3 (review* OR overview* OR synthes*)).ab) AND (search*.ab OR database*.ab OR "data base*".ab)) OR metasynthes*.ti,ab OR "meta synthes*".ti,ab)* OR (exp "clinical trial"/ OR exp "randomization"/ OR exp "single blind procedure"/ OR exp "double blind procedure"/ OR exp "crossover procedure"/ OR exp "placebo"/ OR exp "prospective study"/ OR rct.ti,ab OR random*.ti,ab OR "single blind".ti,ab OR "randomised controlled trial".ti,ab OR exp "randomized controlled trial"/ OR placebo*.ti,ab) **OR (exp "Comparative Study"/ OR "comparison".ti,ab OR "comparative".ti,ab OR "compar*".ti,ab OR "major clinical study"/ OR "clinical study"/ OR "case control study"/ OR "family study"/ OR "longitudinal study"/ OR "retrospective study"/ OR "prospective study"/ OR "cohort analysis"/ OR cohort*.ti,ab OR (("case control" ADJ1 (study OR studies)).ti,ab) OR (("follow up" ADJ1 (study OR studies)).ti,ab) OR (observational ADJ1 (study OR studies)) OR ((epidemiologic ADJ1 (study OR studies)).ti,ab) OR (("cross sectional" ADJ1 (study OR studies)).ti,ab)) OR** ("life cycle assessment"/ OR "environmental impact assessment"/ OR "life cycle assess*".mp OR "life cycle assessment".mp OR "life cycle inventory".mp OR "LCA".mp OR "LCAs".mp OR "life cycle inventory".mp OR "life cycle inventories".mp)**)**

**Web of Science**

(TS=("Robot Assisted Surgery" OR "robotic surgical device" OR "Robotic Surgical Procedures" OR "Robotic Surgical Procedure" OR "Robotic Surgical" OR "Robotic Surgical*" OR "Robotic Surgery" OR "Robotic Surg*" OR "Robot Surgical Procedures" OR "Robot Surgical Procedure" OR "Robot Surgical" OR "Robot Surgical*" OR "Robot Surgery" OR "Robot Surg*" OR "Robot Assisted Surg*" OR "Robot Assisted Surgery" OR "Robot Enhanced Procedure" OR "Robot Enhanced Procedures" OR "Robot Enhanced Surg*" OR "Robot Enhanced Surgery" OR "Robotic Assisted Surg*" OR "Robotic Assisted Surgery" OR "Robot Assisted Procedure" OR "Robot Assisted Procedures" OR "Robotic Assisted Procedure" OR "Robotic Assisted Procedures" OR "Da Vinci robot" OR "Da Vinci robots" OR "Da Vinci robotic" OR "Da Vinci robotics" OR "Da Vinci robot*" OR "Surgery robot" OR "Surgery robots" OR "Surgery robotic" OR "Surgery robotics" OR "Surgery robot*" OR "Surgical robot" OR "Surgical robots" OR "Surgical robotic" OR "Surgical robotics" OR "Surgical robot*" OR "Da Vinci" OR (("Surgery" OR "surgery" OR "surgical" OR "surgical*" OR "Surgeon" OR "surgeon" OR "surgeons" OR "surgeon*" OR "neurosurgery" OR "neurosurgical" OR "neurosurgical*" OR "neurosurgeon" OR "neurosurgeons" OR "neurosurgeon*" OR "radiosurgery" OR "radiosurgical" OR "radiosurgical*" OR "radiosurgeon" OR "radiosurgeons" OR "radiosurgeon*") AND ("Robotics" OR "Robotics" OR "Robot" OR "Medical Robot" OR "Robot" OR "Robots" OR "Robotic" OR "Robot*" OR "Telerobotics" OR "Telerobot*"))) AND TS=("Laparoscopy" OR "Laparoscopy" OR "Laparoscop*" OR "Laparotomy" OR "Laparotomy" OR "Laparotom*" OR "Minimally Invasive Surgery" OR "Minimally Invasive Surgery" OR "Minimally Invasive" OR "Minimal Surgical" OR "Minimal Access" OR "open surgery" OR "open surgery" OR ("Surgery" NOT ("Robot Assisted Surgery" OR "robotic surgical device")) OR "conventional surgery" OR "Endoscopy" OR "Endoscopy" OR "endoscop*") AND (TS=("Carbon Footprint" OR "carbon footprint" OR "carbon footprint*" OR "Climate Change" OR "climate change" OR "CO2 emission" OR "CO2 emissions" OR "CO2 footprint" OR "CO2 footprint*" OR "environmental protection" OR "conservation of natural resources" OR "environmental protection" OR "Disposable Equipment" OR "Disposables" OR "Disposable" OR "ecological footprint" OR "ecological footprint*" OR "ecological sustainability" OR "environmental impact" OR "environmental impact" OR "environmental impact*" OR "environmental impacts" OR "environmental pollut*" OR "pollution" OR "environmental pollution" OR "environmental sustainab*" OR "environmental sustainability" OR "environmental sustainability" OR "Global Warming" OR "Global Warming" OR "Greenhouse Effect" OR "greenhouse effect*" OR "greenhouse effects" OR "greenhouse gas emission" OR "greenhouse gas emissions" OR "Greenhouse Gas" OR "greening" OR "hospital waste" OR "life cycle assessment" OR "environmental impact assessment" OR "life cycle assess*" OR "life cycle assessment" OR "LCA" OR "LCAs" OR "life cycle inventory" OR "life cycle inventories" OR "Waste Disposal" OR "Hospital Waste" OR "medical waste" OR "Rising Sea Level" OR "Rising Sea Levels" OR "Sea Level Rise" OR "Sea Level Rise" OR "Waste Disposal" OR "waste water recycling" OR "Recycling" OR "recycling" OR "recycle*" OR "Equipment reuse" OR "Reusables" OR "reusable" OR "reuse" OR "reused" OR "reusing" OR "Waste Disposal" OR "Waste Management" OR "Plastic overuse" OR "Green surgery" OR "Emission reduction" OR "Emission reduction strategy" OR "air pollution control" OR "air pollution control" OR "acidification" OR "soil acidification" OR "ocean acidification" OR "acidification" OR "Acidification potential" OR "AP in kg SO2 equivalents" OR "eco-efficiency" OR "ecoefficiency" OR "eco-efficien*" OR "ecoefficien*" OR "ecotoxicity" OR "ecotoxicity" OR "ecotoxic*" OR "eco toxicity" OR "eco toxic*" OR "EP in kg PO4 equivalent" OR "Eutrophication" OR "eutrophication" OR "Eutrophication potential" OR "FAETP in kg DCB equivalent" OR "Freshwater Aquatic Ecotoxicity Potential" OR "GWP in kg CO2 equivalents" OR "H+ moles equivalents" OR "HTTP in kg Dichlorobenzene equivalent" OR "Human Toxicity Potential" OR "kg 2.4-D equivalents" OR "kg CFC-11 equivalent" OR "kg N equivalents" OR "kg NOx equivalents" OR "life cycle analysis" OR "ozone depletion" OR "Photochemical Ozone Depletion Potential" OR "POCP in kg ethane equivalent" OR "smog" OR "Waste" OR "waste" OR "wastes" OR "Ozone Depletion" OR "Smog" OR "Equipment reuse" OR "Greenhouse Gases" OR "Greenhouse Gas" OR "SO2 equiv*" OR "CO2 equiva*" OR "CFC-11 equiv*" OR "N equiv*" OR "Sustainable Development" OR "Sustainable Development" OR "Biodiversity" OR "Climatic change" OR "Green deal" OR "preservation of natural resources" OR "Refuse Disposal" OR "Waste Water" OR "Wastewater" OR "Water Purification" OR (("plastic*" OR "microplastic*") AND ("soop" OR "soup" OR "pollution" OR "overuse" OR "contamination")) OR (("Plastic" OR "plastics") AND "overuse") OR ("hydrogen*" AND "moles" AND "equiv*") OR ("Dichlorobenzen*" AND "equiv*") OR ("2,4-D" AND "equiv*") OR ("NOx" AND "equiv*") OR ("ethane" AND "equiv*") OR ("PO4" AND "equiv*") OR ("DCB" AND "equiv*") OR ("sustainability" AND ("environment*" OR "carbon")) OR (("Carbon Dioxide" OR "Carbon Dioxide" OR "CO2") AND ("pollution" OR "emission" OR "emissions" OR "waste" OR "environment" OR "environmental*" OR "footprint" OR "footprint*" OR "sustainable" OR "hazard" OR "hazard*"))) OR **TI=("environmental*" OR "sustainab*")**) NOT DT=(meeting abstract) AND PY=(2000 OR 2001 OR 2002 OR 2003 OR 2004 OR 2005 OR 2006 OR 2007 OR 2008 OR 2009 OR 2010 OR 2011 OR 2012 OR 2013 OR 2014 OR 2015 OR 2016 OR 2017 OR 2018 OR 2019 OR 2020 OR 2021 OR 2022)) AND **(**TI=**(***("Meta-Analysis" OR metaanaly* OR "meta-analy*" or metanaly* OR "Systematic Review" OR "Cochrane Database Syst Rev" OR "prisma" OR "preferred reporting items" OR "prospero" OR ((systemati* OR scoping OR umbrella OR "structured literature") NEAR/4 (review* OR overview*)) OR "systematic review*" OR "scoping review*" OR "umbrella review*" OR "structured literature review*" OR "systematic qualitative review*" OR "systematic quantitative review*" OR "systematic search and review" OR "systematized review" OR "systematised review" OR "systemic review" OR "systematic literature review*" OR "systematic integrative literature review*" OR "systematically review*" OR "scoping literature review*" OR "systematic critical review" OR "systematic integrative review*" OR "systematic evidence review" OR "Systematic integrative literature review*" OR "Systematic mixed studies review*" OR "Systematized literature review*" OR "Systematic overview*" OR "Systematic narrative review*" OR (("systemati*" OR "literature" OR "database*" OR "data-base*" OR "structured" OR "comprehensive*" OR "systemic*") NEAR/4 "search*") OR ("Literature" AND "review" AND ("database*" OR "data-base*" OR "search*")) OR (("data extraction" OR "data source*") AND "study selection") OR ("search strategy" AND "selection criteria") OR ("data source*" AND "data synthesis") OR "medline" OR "pubmed" OR "embase" OR "Cochrane" OR (("critical" OR "rapid") NEAR/4 ("review*" OR "overview*" OR "synthes*")) OR ((("critical*" OR "rapid*") NEAR/4 ("review*" OR "overview*" OR "synthes*") NEAR/4 ("search*" OR "database*" OR "data-base*"))) OR metasynthes* OR "meta-synthes*")* **OR** ("Randomized Controlled Trial" OR random* OR "RCT"OR "RCTs" OR "pragmatic clinical trial*" OR "practical clinical trial*" OR "non-inferiority trial*" OR "noninferiority trial*" OR "superiority trial*" OR "equivalence clinical trial*") **OR ("Comparative Study" OR "comparison" OR "comparative" OR "compar*" OR "major clinical study" OR "clinical study" OR "case control study" OR "family study" OR "longitudinal study" OR "retrospective study" OR "prospective study" OR "cohort analysis" OR cohort* OR (("case control" NEAR/1 (study OR studies))) OR (("follow up" NEAR/1 (study OR studies))) OR (observational NEAR/1 (study OR studies)) OR ((epidemiologic NEAR/1 (study OR studies))) OR (("cross sectional" NEAR/1 (study OR studies)))) OR** ("life cycle assess*" OR "life cycle assessment" OR "life cycle inventory" OR "LCA" OR "LCAs" OR "life cycle inventory" OR "life cycle inventories")**)** OR AB=**(***("Meta-Analysis" OR metaanaly* OR "meta-analy*" or metanaly* OR "Systematic Review" OR "Cochrane Database Syst Rev" OR "prisma" OR "preferred reporting items" OR "prospero" OR ((systemati* OR scoping OR umbrella OR "structured literature") NEAR/4 (review* OR overview*)) OR "systematic review*" OR "scoping review*" OR "umbrella review*" OR "structured literature review*" OR "systematic qualitative review*" OR "systematic quantitative review*" OR "systematic search and review" OR "systematized review" OR "systematised review" OR "systemic review" OR "systematic literature review*" OR "systematic integrative literature review*" OR "systematically review*" OR "scoping literature review*" OR "systematic critical review" OR "systematic integrative review*" OR "systematic evidence review" OR "Systematic integrative literature review*" OR "Systematic mixed studies review*" OR "Systematized literature review*" OR "Systematic overview*" OR "Systematic narrative review*" OR (("systemati*" OR "literature" OR "database*" OR "data-base*" OR "structured" OR "comprehensive*" OR "systemic*") NEAR/4 "search*") OR ("Literature" AND "review" AND ("database*" OR "data-base*" OR "search*")) OR (("data extraction" OR "data source*") AND "study selection") OR ("search strategy" AND "selection criteria") OR ("data source*" AND "data synthesis") OR "medline" OR "pubmed" OR "embase" OR "Cochrane" OR (("critical" OR "rapid") NEAR/4 ("review*" OR "overview*" OR "synthes*")) OR ((("critical*" OR "rapid*") NEAR/4 ("review*" OR "overview*" OR "synthes*") NEAR/4 ("search*" OR "database*" OR "data-base*"))) OR metasynthes* OR "meta-synthes*")* **OR** ("Randomized Controlled Trial" OR random* OR "RCT"OR "RCTs" OR "pragmatic clinical trial*" OR "practical clinical trial*" OR "non-inferiority trial*" OR "noninferiority trial*" OR "superiority trial*" OR "equivalence clinical trial*") **OR ("Comparative Study" OR "comparison" OR "comparative" OR "compar*" OR "major clinical study" OR "clinical study" OR "case control study" OR "family study" OR "longitudinal study" OR "retrospective study" OR "prospective study" OR "cohort analysis" OR cohort* OR (("case control" NEAR/1 (study OR studies))) OR (("follow up" NEAR/1 (study OR studies))) OR (observational NEAR/1 (study OR studies)) OR ((epidemiologic NEAR/1 (study OR studies))) OR (("cross sectional" NEAR/1 (study OR studies)))) OR** ("life cycle assess*" OR "life cycle assessment" OR "life cycle inventory" OR "LCA" OR "LCAs" OR "life cycle inventory" OR "life cycle inventories")**))**

**Cochrane Library**

("Robot Assisted Surgery" OR "robotic surgical device" OR "Robotic Surgical Procedures" OR "Robotic Surgical Procedure" OR "Robotic Surgical" OR "Robotic Surgical*" OR "Robotic Surgery" OR "Robotic Surg*" OR "Robot Surgical Procedures" OR "Robot Surgical Procedure" OR "Robot Surgical" OR "Robot Surgical*" OR "Robot Surgery" OR "Robot Surg*" OR "Robot Assisted Surg*" OR "Robot Assisted Surgery" OR "Robot Enhanced Procedure" OR "Robot Enhanced Procedures" OR "Robot Enhanced Surg*" OR "Robot Enhanced Surgery" OR "Robotic Assisted Surg*" OR "Robotic Assisted Surgery" OR "Robot Assisted Procedure" OR "Robot Assisted Procedures" OR "Robotic Assisted Procedure" OR "Robotic Assisted Procedures" OR "Da Vinci robot" OR "Da Vinci robots" OR "Da Vinci robotic" OR "Da Vinci robotics" OR "Da Vinci robot*" OR "Surgery robot" OR "Surgery robots" OR "Surgery robotic" OR "Surgery robotics" OR "Surgery robot*" OR "Surgical robot" OR "Surgical robots" OR "Surgical robotic" OR "Surgical robotics" OR "Surgical robot*" OR "Da Vinci" OR (("Surgery" OR "surgery" OR "surgical" OR "surgical*" OR "Surgeon" OR "surgeon" OR "surgeons" OR "surgeon*" OR "neurosurgery" OR "neurosurgical" OR "neurosurgical*" OR "neurosurgeon" OR "neurosurgeons" OR "neurosurgeon*" OR "radiosurgery" OR "radiosurgical" OR "radiosurgical*" OR "radiosurgeon" OR "radiosurgeons" OR "radiosurgeon*") AND ("Robotics" OR "Robotics" OR "Robot" OR "Medical Robot" OR "Robot" OR "Robots" OR "Robotic" OR "Robot*" OR "Telerobotics" OR "Telerobot*"))):ti,ab,kw AND ("Laparoscopy" OR "Laparoscopy" OR "Laparoscop*" OR "Laparotomy" OR "Laparotomy" OR "Laparotom*" OR "Minimally Invasive Surgery" OR "Minimally Invasive Surgery" OR "Minimally Invasive" OR "Minimal Surgical" OR "Minimal Access" OR "open surgery" OR "open surgery" OR ("Surgery" NOT ("Robot Assisted Surgery" OR "robotic surgical device")) OR "conventional surgery" OR "Endoscopy" OR "Endoscopy" OR "endoscop*"):ti,ab,kw AND (("Carbon Footprint" OR "carbon footprint" OR "carbon footprint*" OR "Climate Change" OR "climate change" OR "CO2 emission" OR "CO2 emissions" OR "CO2 footprint" OR "CO2 footprint*" OR "environmental protection" OR "conservation of natural resources" OR "environmental protection" OR "Disposable Equipment" OR "Disposables" OR "Disposable" OR "ecological footprint" OR "ecological footprint*" OR "ecological sustainability" OR "environmental impact" OR "environmental impact" OR "environmental impact*" OR "environmental impacts" OR "environmental pollut*" OR "pollution" OR "environmental pollution" OR "environmental sustainab*" OR "environmental sustainability" OR "environmental sustainability" OR "Global Warming" OR "Global Warming" OR "Greenhouse Effect" OR "greenhouse effect*" OR "greenhouse effects" OR "greenhouse gas emission" OR "greenhouse gas emissions" OR "Greenhouse Gas" OR "greening" OR "hospital waste" OR "life cycle assessment" OR "environmental impact assessment" OR "life cycle assessment" OR "environmental impact assessment" OR "life cycle assess*" OR "life cycle assessment" OR "LCA" OR "LCAs" OR "life cycle inventory" OR "life cycle inventories" OR "Waste Disposal" OR "Hospital Waste" OR "medical waste" OR "Rising Sea Level" OR "Rising Sea Levels" OR "Sea Level Rise" OR "Sea Level Rise" OR "Waste Disposal" OR "waste water recycling" OR "Recycling" OR "recycling" OR "recycle*" OR "Equipment reuse" OR "Reusables" OR "reusable" OR "reuse" OR "reused" OR "reusing" OR "Waste Disposal" OR "Waste Management" OR "Plastic overuse" OR "Green surgery" OR "Emission reduction" OR "Emission reduction strategy" OR "air pollution control" OR "air pollution control" OR "acidification" OR "soil acidification" OR "ocean acidification" OR "acidification" OR "Acidification potential" OR "AP in kg SO2 equivalents" OR "eco-efficiency" OR "ecoefficiency" OR "eco-efficien*" OR "ecoefficien*" OR "ecotoxicity" OR "ecotoxicity" OR "ecotoxic*" OR "eco toxicity" OR "eco toxic*" OR "EP in kg PO4 equivalent" OR "Eutrophication" OR "eutrophication" OR "Eutrophication potential" OR "FAETP in kg DCB equivalent" OR "Freshwater Aquatic Ecotoxicity Potential" OR "GWP in kg CO2 equivalents" OR "H+ moles equivalents" OR "HTTP in kg Dichlorobenzene equivalent" OR "Human Toxicity Potential" OR "kg 2.4-D equivalents" OR "kg CFC-11 equivalent" OR "kg N equivalents" OR "kg NOx equivalents" OR "life cycle analysis" OR "ozone depletion" OR "Photochemical Ozone Depletion Potential" OR "POCP in kg ethane equivalent" OR "smog" OR "Waste" OR "waste" OR "wastes" OR "Ozone Depletion" OR "Smog" OR "Equipment reuse" OR "Greenhouse Gases" OR "Greenhouse Gas" OR "SO2 equiv*" OR "CO2 equiva*" OR "CFC-11 equiv*" OR "N equiv*" OR "Biodiversity" OR "Climatic change" OR "Green deal" OR "preservation of natural resources" OR "Refuse Disposal" OR "Waste Water" OR "Wastewater" OR "Water Purification" OR (("plastic*" OR "microplastic*") AND ("soop" OR "soup" OR "pollution" OR "overuse" OR "contamination")) OR "Sustainable Development" OR "Sustainable Development" OR (("Plastic" OR "plastics") AND "overuse") OR ("hydrogen*" AND "moles" AND "equiv*") OR ("Dichlorobenzen*" AND "equiv*") OR ("2,4-D" AND "equiv*") OR ("NOx" AND "equiv*") OR ("ethane" AND "equiv*") OR ("PO4" AND "equiv*") OR ("DCB" AND "equiv*") OR ("sustainability" AND ("environment*" OR "carbon")) OR (("Carbon Dioxide" OR "Carbon Dioxide" OR "CO2") AND ("pollution" OR "emission" OR "emissions" OR "waste" OR "environment" OR "environmental*" OR "footprint" OR "footprint*" OR "sustainable" OR "hazard" OR "hazard*"))):ti,ab,kw OR **("environmental" OR "sustainability"):ti**)

**Emcare (OVID)**

((exp "Robot Assisted Surgery"/ OR exp "robotic surgical device"/ OR "Robotic Surgical Procedures".mp OR "Robotic Surgical Procedure".mp OR "Robotic Surgical".mp OR "Robotic Surgical*".mp OR "Robotic Surgery".mp OR "Robotic Surg*".mp OR "Robot Surgical Procedures".mp OR "Robot Surgical Procedure".mp OR "Robot Surgical".mp OR "Robot Surgical*".mp OR "Robot Surgery".mp OR "Robot Surg*".mp OR "Robot Assisted Surg*".mp OR "Robot Assisted Surgery".mp OR "Robot Enhanced Procedure".mp OR "Robot Enhanced Procedures".mp OR "Robot Enhanced Surg*".mp OR "Robot Enhanced Surgery".mp OR "Robotic Assisted Surg*".mp OR "Robotic Assisted Surgery".mp OR "Robot Assisted Procedure".mp OR "Robot Assisted Procedures".mp OR "Robotic Assisted Procedure".mp OR "Robotic Assisted Procedures".mp OR "Da Vinci robot".mp OR "Da Vinci robots".mp OR "Da Vinci robotic".mp OR "Da Vinci robotics".mp OR "Da Vinci robot*".mp OR "Surgery robot".mp OR "Surgery robots".mp OR "Surgery robotic".mp OR "Surgery robotics".mp OR "Surgery robot*".mp OR "Surgical robot".mp OR "Surgical robots".mp OR "Surgical robotic".mp OR "Surgical robotics".mp OR "Surgical robot*".mp OR "Da Vinci".mp OR ((exp "Surgery"/ OR "surgery".mp OR "surgical".mp OR "surgical*".mp OR exp "Surgeon"/ OR "surgeon".mp OR "surgeons".mp OR "surgeon*".mp OR "neurosurgery".mp OR "neurosurgical".mp OR "neurosurgical*".mp OR "neurosurgeon".mp OR "neurosurgeons".mp OR "neurosurgeon*".mp OR "radiosurgery".mp OR "radiosurgical".mp OR "radiosurgical*".mp OR "radiosurgeon".mp OR "radiosurgeons".mp OR "radiosurgeon*".mp) AND ("Robotics"/ OR "Robotics".mp OR exp "Robot"/ OR exp "Medical Robot"/ OR "Robot".mp OR "Robots".mp OR "Robotic".mp OR "Robot*".mp OR "Telerobotics".mp OR "Telerobot*".mp))) AND (exp "Laparoscopy"/ OR "Laparoscopy".mp OR "Laparoscop*".mp OR "Laparotomy"/ OR "Laparotomy".mp OR "Laparotom*".mp OR "Minimally Invasive Surgery"/ OR "Minimally Invasive Surgery".mp OR "Minimally Invasive".mp OR "Minimal Surgical".mp OR "Minimal Access".mp OR "open surgery"/ OR "open surgery".mp OR (exp "Surgery"/ NOT (exp "Robot Assisted Surgery"/ OR exp "robotic surgical device"/)) OR "conventional surgery".mp OR exp "Endoscopy"/ OR "Endoscopy".mp OR "endoscop*".mp) AND ("Carbon Footprint"/ OR "carbon footprint".mp OR "carbon footprint*".mp OR exp "Climate Change"/ OR "climate change".mp OR "CO2 emission".mp OR "CO2 emissions".mp OR "CO2 footprint".mp OR "CO2 footprint*".mp OR exp "environmental protection"/ OR "conservation of natural resources".mp OR "environmental protection".mp OR "Disposable Equipment"/ OR "Disposables".mp OR "Disposable".mp OR "ecological footprint".mp OR "ecological footprint*".mp OR "ecological sustainability".mp OR exp "environmental impact"/ OR "environmental impact".mp OR "environmental impact*".mp OR "environmental impacts".mp OR "environmental pollut*".mp OR exp "pollution"/ OR "environmental pollution".mp OR "environmental sustainab*".mp OR "environmental sustainability"/ OR "environmental sustainability".mp OR "Global Warming"/ OR "Global Warming".mp OR "Greenhouse Effect"/ OR "greenhouse effect*".mp OR "greenhouse effects".mp OR "greenhouse gas emission".mp OR "greenhouse gas emissions".mp OR "Greenhouse Gas"/ OR "greening".mp OR "hospital waste".mp OR "life cycle assessment"/ OR "environmental impact assessment"/ OR "life cycle assess*".mp OR "life cycle assessment".mp OR "LCA".mp OR "LCAs".mp OR "life cycle inventory".mp OR "life cycle inventories".mp OR exp "Waste Disposal"/ OR exp "Hospital Waste"/ OR "medical waste".mp OR "Rising Sea Level".mp OR "Rising Sea Levels".mp OR "Sea Level Rise"/ OR "Sea Level Rise".mp OR "sustainability"**.ti** OR "Waste Disposal".mp OR "waste water recycling"/ OR "Recycling"/ OR "recycling".mp OR "recycle*".mp OR "Equipment reuse".mp OR "Reusables".mp OR "reusable".mp OR "reuse".mp OR "reused".mp OR "reusing".mp OR exp "Waste Disposal"/ OR exp "Waste Management"/ OR "Plastic overuse".mp OR "Green surgery".mp OR "Emission reduction".mp OR "Emission reduction strategy".mp OR "air pollution control"/ OR "air pollution control".mp **OR "Environment"/ OR "Environmental*".ti** OR "acidification"/ OR "soil acidification"/ OR "ocean acidification"/ OR "acidification".mp OR "Acidification potential".mp OR "AP in kg SO2 equivalents".mp OR "eco-efficiency".mp OR "ecoefficiency".mp OR "eco-efficien*".mp OR "ecoefficien*".mp OR "ecotoxicity"/ OR "ecotoxicity".mp OR "ecotoxic*".mp OR "eco toxicity".mp OR "eco toxic*".mp OR "EP in kg PO4 equivalent".mp OR exp "Eutrophication"/ OR "eutrophication".mp OR "Eutrophication potential".mp OR "FAETP in kg DCB equivalent".mp OR "Freshwater Aquatic Ecotoxicity Potential".mp OR "GWP in kg CO2 equivalents".mp OR "H+ moles equivalents".mp OR "HTTP in kg Dichlorobenzene equivalent".mp OR "Human Toxicity Potential".mp OR "kg 2.4-D equivalents".mp OR "kg CFC-11 equivalent".mp OR "kg N equivalents".mp OR "kg NOx equivalents".mp OR "life cycle analysis".mp OR "ozone depletion".mp OR "Photochemical Ozone Depletion Potential".mp OR "POCP in kg ethane equivalent".mp OR "smog".mp OR exp "Waste"/ OR "waste".mp OR "wastes".mp OR "Ozone Depletion"/ OR "Smog"/ OR "Equipment reuse".mp OR "Greenhouse Gases".mp OR "Greenhouse Gas".mp OR "SO2 equiv*".mp OR "CO2 equiva*".mp OR "CFC-11 equiv*".mp OR "N equiv*".mp OR exp "Biodiversity"/ OR "Biodiversity".mp OR "Climatic change".mp OR "Green deal".mp OR "preservation of natural resources".mp OR "Refuse Disposal".mp OR exp "Wastewater"/ OR "Waste Water".mp OR "Wastewater".mp OR exp "Water Management"/ OR "Water Purification".mp OR (("plastic*".mp OR "microplastic*".mp) AND ("soop".mp OR "soup".mp OR "pollution".mp OR "overuse".mp OR "contamination".mp)) OR "Sustainable Development"/ OR "Sustainable Development".mp OR (("Plastic".mp OR "plastics".mp) AND "overuse".mp) OR ("hydrogen*".mp AND "moles".mp AND "equiv*".mp) OR ("Dichlorobenzen*".mp AND "equiv*".mp) OR ("2,4-D".mp AND "equiv*".mp) OR ("NOx".mp AND "equiv*".mp) OR ("ethane".mp AND "equiv*".mp) OR ("PO4".mp AND "equiv*".mp) OR ("DCB".mp AND "equiv*".mp) OR ("sustainability".mp AND ("environment*".mp OR "carbon".mp)) OR (("Carbon Dioxide"/ OR "Carbon Dioxide".mp OR "CO2".mp) AND ("pollution".mp OR "emission".mp OR "emissions".mp OR "waste".mp OR "environment".mp OR "environmental*".mp OR "footprint".mp OR "footprint*".mp OR "sustainable".mp OR "hazard".mp OR "hazard*".mp))) NOT (conference review or conference abstract).pt AND (2000 OR 2001 OR 2002 OR 2003 OR 2004 OR 2005 OR 2006 OR 2007 OR 2008 OR 2009 OR 2010 OR 2011 OR 2012 OR 2013 OR 2014 OR 2015 OR 2016 OR 2017 OR 2018 OR 2019 OR 2020 OR 2021 OR 2022).yr) AND **(***(exp "meta analysis"/ OR exp "meta analysis (topic)"/ OR metaanaly*.ti,ab OR "meta analy*".ti,ab OR metanaly*.ti,ab OR "systematic review"/ OR "cochrane database of systematic reviews".jn OR prisma.ti,ab OR prospero.ti,ab OR (((systemati* OR scoping OR umbrella OR "structured literature") ADJ3 (review* OR overview*)).ti,ab) OR ((systemic* ADJ1 review*).ti,ab) OR (((systemati* OR literature OR database* OR "data base*") ADJ10 search*).ti,ab) OR (((structured OR comprehensive* OR systemic*) ADJ3 search*).ti,ab) OR (((literature ADJ3 review*).ti,ab) AND (search*.ti,ab OR database*.ti,ab OR "data base*".ti,ab)) OR (("data extraction".ti,ab OR "data source*".ti,ab) AND "study selection".ti,ab) OR ("search strategy".ti,ab AND "selection criteria".ti,ab) OR ("data source*".ti,ab AND "data synthesis".ti,ab) OR medline.ab OR pubmed.ab OR embase.ab OR cochrane.ab OR (((critical OR rapid) ADJ2 (review* OR overview* OR synthes*)).ti) OR ((((critical* OR rapid*) ADJ3 (review* OR overview* OR synthes*)).ab) AND (search*.ab OR database*.ab OR "data base*".ab)) OR metasynthes*.ti,ab OR "meta synthes*".ti,ab)* OR (exp "clinical trial"/ OR exp "randomization"/ OR exp "single blind procedure"/ OR exp "double blind procedure"/ OR exp "crossover procedure"/ OR exp "placebo"/ OR exp "prospective study"/ OR rct.ti,ab OR random*.ti,ab OR "single blind".ti,ab OR "randomised controlled trial".ti,ab OR exp "randomized controlled trial"/ OR placebo*.ti,ab) **OR (exp "Comparative Study"/ OR "comparison".ti,ab OR "comparative".ti,ab OR "compar*".ti,ab OR "major clinical study"/ OR "clinical study"/ OR "case control study"/ OR "family study"/ OR "longitudinal study"/ OR "retrospective study"/ OR "prospective study"/ OR "cohort analysis"/ OR cohort*.ti,ab OR (("case control" ADJ1 (study OR studies)).ti,ab) OR (("follow up" ADJ1 (study OR studies)).ti,ab) OR (observational ADJ1 (study OR studies)) OR ((epidemiologic ADJ1 (study OR studies)).ti,ab) OR (("cross sectional" ADJ1 (study OR studies)).ti,ab)) OR** ("life cycle assessment"/ OR "environmental impact assessment"/ OR "life cycle assess*".mp OR "life cycle assessment".mp OR "life cycle inventory".mp OR "LCA".mp OR "LCAs".mp OR "life cycle inventory".mp OR "life cycle inventories".mp)**)**

### Topic 2: Disposable vs. reusable medical devices (PICO1)

**MEDLINE (PubMed)**

(("Equipment Reuse"[mesh] OR "reusable biopsy forceps"[tw] OR "reusable drape"[tw] OR "reusable drapes"[tw] OR "reusable equipment"[tw] OR "reusable flexible optical scope"[tw] OR "reusable flexible optical scopes"[tw] OR "reusable forceps"[tw] OR "reusable laryngeal mask"[tw] OR "reusable laryngeal masks"[tw] OR "reusable medical equipment"[tw] OR "reusable medical equipment packaging"[tw] OR "reusable scissor"[tw] OR "reusable scissors"[tw] OR "reusable scope"[tw] OR "reusable scopes"[tw] OR "reusable sharps container"[tw] OR "reusable sharps containers"[tw] OR "reusable specula"[tw] OR "reusable speculum"[tw] OR "reusable surgical attire"[tw] OR "reusable surgical attires"[tw] OR "reusable surgical clamp"[tw] OR "reusable surgical clamps"[tw] OR "reusable surgical clip"[tw] OR "reusable surgical clips"[tw] OR "reusable surgical kit"[tw] OR "reusable surgical kits"[tw] OR "reusable suture anchor"[tw] OR "reusable suture anchors"[tw] OR "reusable trocar"[tw] OR "reusable trocars"[tw] OR "reusable ureteroscope"[tw] OR "reusable ureteroscopes"[tw] OR "recyclable biopsy forceps"[tw] OR "recyclable drape"[tw] OR "recyclable drapes"[tw] OR "recyclable equipment"[tw] OR "recyclable flexible optical scope"[tw] OR "recyclable flexible optical scopes"[tw] OR "recyclable forceps"[tw] OR "recyclable laryngeal mask"[tw] OR "recyclable laryngeal masks"[tw] OR "recyclable medical equipment"[tw] OR "recyclable medical equipment packaging"[tw] OR "recyclable scissor"[tw] OR "recyclable scissors"[tw] OR "recyclable scope"[tw] OR "recyclable scopes"[tw] OR "recyclable sharps container"[tw] OR "recyclable sharps containers"[tw] OR "recyclable specula"[tw] OR "recyclable speculum"[tw] OR "recyclable surgical attire"[tw] OR "recyclable surgical attires"[tw] OR "recyclable surgical clamp"[tw] OR "recyclable surgical clamps"[tw] OR "recyclable surgical clip"[tw] OR "recyclable surgical clips"[tw] OR "recyclable surgical kit"[tw] OR "recyclable surgical kits"[tw] OR "recyclable suture anchor"[tw] OR "recyclable suture anchors"[tw] OR "recyclable trocar"[tw] OR "recyclable trocars"[tw] OR "recyclable ureteroscope"[tw] OR "recyclable ureteroscopes"[tw] OR (("Equipment Reuse"[mesh] OR "reusables"[tw] OR "reusable"[tw] OR "reusabl*"[tw] OR "reuse"[tw] OR "re usables"[tw] OR "re usable"[tw] OR "re usabl*"[tw] OR "re use"[tw] OR "recyclable"[tw] OR "recyclables"[tw] OR "recycl*"[tw] OR "re cyclable"[tw] OR "re cycl*"[tw] OR "Recycling"[Mesh] OR "reprocess"[tw] OR "reprocessing"[tw] OR "reprocess*"[tw]) AND ("biopsy forceps"[tw] OR "Surgical Drapes"[Mesh] OR "drape"[tw] OR "drapes"[tw] OR "Surgical Equipment"[Mesh] OR "equipment"[tw] OR "flexible optical scope"[tw] OR "flexible optical scopes"[tw] OR "forceps"[tw] OR "Laryngeal Masks"[Mesh] OR "laryngeal mask"[tw] OR "laryngeal masks"[tw] OR "medical equipment"[tw] OR "medical equipment packaging"[tw] OR "scissor"[tw] OR "scissors"[tw] OR "Endoscopes"[Mesh] OR "scopes"[tw] OR "Endoscopes"[tw] OR "Endoscope"[tw] OR "sharps container"[tw] OR "sharps containers"[tw] OR "sharps bin"[tw] OR "sharps bins"[tw] OR "specula"[tw] OR "speculum"[tw] OR "Surgical Attire"[Mesh] OR "surgical attire"[tw] OR "surgical attires"[tw] OR "Surgical Shoe Covers"[tw] OR "Surgical Shoe Cover"[tw] OR "Surgical Gowns"[tw] OR "Surgical Gown"[tw] OR "Masks"[Mesh] OR "Mask"[tw] OR "Masks"[tw] OR "Respirators"[tw] OR "Respirator"[tw] OR "Surgical Instruments"[Mesh] OR "surgical clamp"[tw] OR "surgical clamps"[tw] OR "surgical clip"[tw] OR "surgical clips"[tw] OR "Forcep"[tw] OR "Forceps"[tw] OR "Surgical Clamp"[tw] OR "Surgical Clamps"[tw] OR "Surgical Hook"[tw] OR "Surgical Hooks"[tw] OR "Surgical Instrument"[tw] OR "Surgical Instruments"[tw] OR "Surgical Plug"[tw] OR "Surgical Plugs"[tw] OR "Surgical Scissor"[tw] OR "Surgical Scissors"[tw] OR "Surgical Valve"[tw] OR "Surgical Valves"[tw] OR "Tantalum Clip"[tw] OR "Tantalum Clips"[tw] OR "Surgical Staplers"[tw] OR "Surgical Stapler"[tw] OR "surgical kit"[tw] OR "surgical kits"[tw] OR "Suture Anchors"[Mesh] OR "suture anchor"[tw] OR "suture anchors"[tw] OR "Bone Anchor"[tw] OR "Bone Anchors"[tw] OR "trocar"[tw] OR "trocars"[tw] OR "Ureteroscopes"[Mesh] OR "ureteroscope"[tw] OR "ureteroscopes"[tw] OR "Eyeglasses"[Mesh] OR "band aid"[tw] OR "band aids"[tw] OR "bandaid"[tw] OR "bandaids"[tw] OR "cap"[tw] OR "caps"[tw] OR "cover material"[tw] OR "cover materials"[tw] OR "covering material"[tw] OR "covering materials"[tw] OR "Eyeglasses"[tw] OR "glasses"[tw] OR "glove"[tw] OR "gloves"[tw] OR "gown"[tw] OR "gowns"[tw] OR "packing material"[tw] OR "packing materials"[tw] OR "patch"[tw] OR "patches"[tw] OR "spectacles"[tw] OR "surgery instrument"[tw] OR "surgery instruments"[tw] OR "surgical instrument"[tw] OR "surgical instruments"[tw] OR "medical device"[tw] OR "medical devices"[tw] OR "surgical device"[tw] OR "surgical devices"[tw]))) AND ("Disposable Equipment"[Mesh] OR "disposable biopsy forceps"[tw] OR "disposable drape"[tw] OR "disposable drapes"[tw] OR "disposable equipment"[tw] OR "disposable flexible optical scope"[tw] OR "disposable flexible optical scopes"[tw] OR "disposable laryngeal mask"[tw] OR "disposable laryngeal masks"[tw] OR "disposable medical equipment packaging"[tw] OR "disposable scissor"[tw] OR "disposable scissors"[tw] OR "disposable scope"[tw] OR "disposable scopes"[tw] OR "disposable sharps container"[tw] OR "disposable sharps containers"[tw] OR "disposable specula"[tw] OR "disposable speculum"[tw] OR "disposable surgical attire"[tw] OR "disposable surgical clamp"[tw] OR "disposable surgical clamps"[tw] OR "disposable surgical clip"[tw] OR "disposable surgical clips"[tw] OR "disposable surgical kit"[tw] OR "disposable surgical kits"[tw] OR "disposable suture anchor"[tw] OR "disposable suture anchors"[tw] OR "disposable trocar"[tw] OR "disposable trocars"[tw] OR "disposable ureteroscope"[tw] OR "disposable ureteroscopes"[tw] OR "single use biopsy forceps"[tw] OR "single use drape"[tw] OR "single use drapes"[tw] OR "single use equipment"[tw] OR "single use flexible optical scope"[tw] OR "single use flexible optical scopes"[tw] OR "single use laryngeal mask"[tw] OR "single use laryngeal masks"[tw] OR "single use medical equipment packaging"[tw] OR "single use scissor"[tw] OR "single use scissors"[tw] OR "single use scope"[tw] OR "single use scopes"[tw] OR "single use sharps container"[tw] OR "single use sharps containers"[tw] OR "single use specula"[tw] OR "single use speculum"[tw] OR "single use surgical attire"[tw] OR "single use surgical clamp"[tw] OR "single use surgical clamps"[tw] OR "single use surgical clip"[tw] OR "single use surgical clips"[tw] OR "single use surgical kit"[tw] OR "single use surgical kits"[tw] OR "single use suture anchor"[tw] OR "single use suture anchors"[tw] OR "single use trocar"[tw] OR "single use trocars"[tw] OR "single use ureteroscope"[tw] OR "single use ureteroscopes"[tw] OR (("Disposable Equipment"[Mesh] OR "disposable"[tw] OR "disposables"[tw] OR "dispos*"[tw] OR "single use"[tw] OR "single use*"[tw] OR "singleuse"[tw] OR "singleuse*"[tw] OR "single usage"[tw] OR "single usage*"[tw]) AND ("biopsy forceps"[tw] OR "Surgical Drapes"[Mesh] OR "drape"[tw] OR "drapes"[tw] OR "Surgical Equipment"[Mesh] OR "equipment"[tw] OR "flexible optical scope"[tw] OR "flexible optical scopes"[tw] OR "forceps"[tw] OR "Laryngeal Masks"[Mesh] OR "laryngeal mask"[tw] OR "laryngeal masks"[tw] OR "medical equipment"[tw] OR "medical equipment packaging"[tw] OR "scissor"[tw] OR "scissors"[tw] OR "Endoscopes"[Mesh] OR "scopes"[tw] OR "Endoscopes"[tw] OR "Endoscope"[tw] OR "sharps container"[tw] OR "sharps containers"[tw] OR "sharps bin"[tw] OR "sharps bins"[tw] OR "specula"[tw] OR "speculum"[tw] OR "Surgical Attire"[Mesh] OR "surgical attire"[tw] OR "surgical attires"[tw] OR "Surgical Shoe Covers"[tw] OR "Surgical Shoe Cover"[tw] OR "Surgical Gowns"[tw] OR "Surgical Gown"[tw] OR "Masks"[Mesh] OR "Mask"[tw] OR "Masks"[tw] OR "Respirators"[tw] OR "Respirator"[tw] OR "Surgical Instruments"[Mesh] OR "surgical clamp"[tw] OR "surgical clamps"[tw] OR "surgical clip"[tw] OR "surgical clips"[tw] OR "Forcep"[tw] OR "Forceps"[tw] OR "Surgical Clamp"[tw] OR "Surgical Clamps"[tw] OR "Surgical Hook"[tw] OR "Surgical Hooks"[tw] OR "Surgical Instrument"[tw] OR "Surgical Instruments"[tw] OR "Surgical Plug"[tw] OR "Surgical Plugs"[tw] OR "Surgical Scissor"[tw] OR "Surgical Scissors"[tw] OR "Surgical Valve"[tw] OR "Surgical Valves"[tw] OR "Tantalum Clip"[tw] OR "Tantalum Clips"[tw] OR "Surgical Staplers"[tw] OR "Surgical Stapler"[tw] OR "surgical kit"[tw] OR "surgical kits"[tw] OR "Suture Anchors"[Mesh] OR "suture anchor"[tw] OR "suture anchors"[tw] OR "Bone Anchor"[tw] OR "Bone Anchors"[tw] OR "trocar"[tw] OR "trocars"[tw] OR "Ureteroscopes"[Mesh] OR "ureteroscope"[tw] OR "ureteroscopes"[tw] OR "Eyeglasses"[Mesh] OR "band aid"[tw] OR "band aids"[tw] OR "bandaid"[tw] OR "bandaids"[tw] OR "cap"[tw] OR "caps"[tw] OR "cover material"[tw] OR "cover materials"[tw] OR "covering material"[tw] OR "covering materials"[tw] OR "Eyeglasses"[tw] OR "glasses"[tw] OR "glove"[tw] OR "gloves"[tw] OR "gown"[tw] OR "gowns"[tw] OR "packing material"[tw] OR "packing materials"[tw] OR "patch"[tw] OR "patches"[tw] OR "spectacles"[tw] OR "surgery instrument"[tw] OR "surgery instruments"[tw] OR "surgical instrument"[tw] OR "surgical instruments"[tw] OR "medical device"[tw] OR "medical devices"[tw] OR "surgical device"[tw] OR "surgical devices"[tw]))) AND ("Acidification potential"[tw] OR "acidification"[tw] OR "air pollution control"[tw] OR "AP in kg SO2 equivalents"[tw] OR "Biodiversity"[Mesh] OR "Biodiversity"[tw] OR "Carbon Footprint"[mesh] OR "carbon footprint"[tw] OR "carbon footprint*"[tw] OR "CFC-11 equiv*"[tw] OR "Climate Change"[Mesh] OR "climate change"[tw] OR "Climatic change"[tw] OR "CO2 emission"[tw] OR "CO2 emissions"[tw] OR "CO2 equiva*"[tw] OR "CO2 footprint"[tw] OR "CO2 footprint*"[tw] OR "conservation of natural resources"[mesh] OR "conservation of natural resources"[tw] OR "eco toxic*"[tw] OR "eco toxicity"[tw] OR "ecoefficien*"[tw] OR "eco-efficien*"[tw] OR "ecoefficiency"[tw] OR "eco-efficiency"[tw] OR "ecological footprint"[tw] OR "ecological footprint*"[tw] OR "ecological sustainability"[tw] OR "ecotoxic*"[tw] OR "ecotoxicity"[tw] OR "Emission reduction strategy"[tw] OR "Emission reduction"[tw] OR **"Environment"[Mesh:noexp]** OR "environmental impact"[tw] OR "environmental impact*"[tw] OR "environmental impacts"[tw] OR "environmental pollut*"[tw] OR "Environmental Pollution"[Mesh] OR "environmental pollution"[tw] OR "environmental protection"[tw] OR "environmental sustainab*"[tw] OR "environmental sustainability"[tw] OR **"Environmental*"[tw]** OR "EP in kg PO4 equivalent"[tw] OR "Eutrophication potential"[tw] OR "Eutrophication"[Mesh] OR "eutrophication"[tw] OR "FAETP in kg DCB equivalent"[tw] OR "Freshwater Aquatic Ecotoxicity Potential"[tw] OR "Global Warming"[mesh] OR "Global Warming"[tw] OR "Green deal"[tw] OR "Green surgery"[tw] OR "Greenhouse Effect"[mesh] OR "greenhouse effect*"[tw] OR "greenhouse effects"[tw] OR "greenhouse gas emission"[tw] OR "greenhouse gas emissions"[tw] OR "Greenhouse Gas"[tw] OR "Greenhouse Gases"[mesh] OR "Greenhouse Gases"[tw] OR "greening"[tw] OR "GWP in kg CO2 equivalents"[tw] OR "H+ moles equivalents"[tw] OR "hospital waste"[tw] OR "HTTP in kg Dichlorobenzene equivalent"[tw] OR "Human Toxicity Potential"[tw] OR "kg 2.4-D equivalents"[tw] OR "kg CFC-11 equivalent"[tw] OR "kg N equivalents"[tw] OR "kg NOx equivalents"[tw] OR "LCA"[tw] OR "LCAs"[tw] OR "life cycle analysis"[tw] OR "life cycle assess*"[tw] OR "life cycle assessment"[tw] OR "life cycle inventories"[tw] OR "life cycle inventory"[tw] OR "Medical Waste Disposal"[mesh] OR "Medical Waste"[mesh] OR "medical waste"[tw] OR "N equiv*"[tw] OR "Ozone Depletion"[Mesh] OR "ozone depletion"[tw] OR "Photochemical Ozone Depletion Potential"[tw] OR "Plastic overuse"[tw] OR "POCP in kg ethane equivalent"[tw] OR "preservation of natural resources"[tw] OR "Refuse Disposal"[Mesh] OR "Refuse Disposal"[tw] OR "Rising Sea Level"[tw] OR "Rising Sea Levels"[tw] OR "Sea Level Rise"[mesh] OR "Sea Level Rise"[tw] OR "Smog"[mesh] OR "smog"[tw] OR "SO2 equiv*"[tw] OR "sustainability"**[tw]** OR "Sustainable Development"[Mesh] OR "Sustainable Development"[tw] OR "Waste Disposal"[tw] OR "Waste Disposal, Fluid"[mesh] OR "Waste Management"[mesh] OR "Waste"[tw] OR "waste"[tw] OR "Waste Water"[Mesh] OR "Waste Water"[tw] OR "wastes"[tw] OR "Wastewater"[tw] OR "Water Purification"[Mesh] OR "Water Purification"[tw] OR (("plastic*"[tw] OR "microplastic*") AND ("soop"[tw] OR "soup"[tw] OR "pollution"[tw] OR "overuse"[tw] OR "contamination"[tw])) OR (("Plastic"[tw] OR "plastics"[tw]) AND "overuse"[tw]) OR ("hydrogen*"[tw] AND "moles"[tw] AND "equiv*"[tw]) OR ("Dichlorobenzen*"[tw] AND "equiv*"[tw]) OR ("2,4-D"[tw] AND "equiv*"[tw]) OR ("NOx"[tw] AND "equiv*"[tw]) OR ("ethane"[tw] AND "equiv*"[tw]) OR ("PO4"[tw] AND "equiv*"[tw]) OR ("DCB"[tw] AND "equiv*"[tw]) OR ("sustainability"[tw] AND ("environment*"[tw] OR "carbon"[tw])) OR (("Carbon Dioxide"[mesh] OR "Carbon Dioxide"[tw] OR "CO2"[tw]) AND ("pollution"[tw] OR "emission"[tw] OR "emissions"[tw] OR "waste"[tw] OR "environment"[tw] OR "environmental*"[tw] OR "footprint"[tw] OR "footprint*"[tw] OR "sustainable"[tw] OR "hazard"[tw] OR "hazard*"[tw]))) AND ("2000/01/01"[PDAT] : "3000/12/31"[PDAT])) AND **(***("Meta-Analysis"[Publication Type] OR "Meta-Analysis as Topic"[Mesh] OR metaanaly*[tiab] OR meta-analy*[tiab] or metanaly*[tiab] OR "Systematic Review"[Publication Type] OR systematic[sb] OR "Cochrane Database Syst Rev"[Journal] or prisma[tiab] OR preferred reporting items[tiab] OR prospero[tiab] OR ((systemati*[ti] OR scoping[ti] OR umbrella[ti] OR structured literature[ti]) AND (review*[ti] OR overview*[ti])) OR systematic review*[tiab] OR scoping review*[tiab] OR umbrella review*[tiab] OR structured literature review*[tiab] OR systematic qualitative review*[tiab] OR systematic quantitative review*[tiab] OR systematic search and review[tiab] OR systematized review[tiab] OR systematised review[tiab] OR systemic review[tiab] OR systematic literature review*[tiab] OR systematic integrative literature review*[tiab] OR systematically review*[tiab] OR scoping literature review*[tiab] OR systematic critical review[tiab] OR systematic integrative review*[tiab] OR systematic evidence review[tiab] OR Systematic integrative literature review*[tiab] OR Systematic mixed studies review*[tiab] OR Systematized literature review*[tiab] OR Systematic overview*[tiab] OR Systematic narrative review*[tiab] OR ((systemati*[tiab] OR literature[tiab] OR database*[tiab] OR data-base*[tiab] OR structured[tiab] OR comprehensive*[tiab] OR systemic*[tiab]) AND search*[tiab]) OR (Literature[ti] AND review[ti] AND (database*[tiab] OR data-base*[tiab] OR search*[tiab])) OR ((data extraction[tiab] OR data source*[tiab]) AND study selection[tiab]) OR (search strategy[tiab] AND selection criteria[tiab]) OR (data source*[tiab] AND data synthesis[tiab]) OR medline[tiab] OR pubmed[tiab] OR embase[tiab] OR Cochrane[tiab] OR ((critical[ti] OR rapid[ti]) AND (review*[ti] OR overview*[ti] OR synthes*[ti])) OR (((critical*[tiab] OR rapid*[tiab]) AND (review*[tiab] OR overview*[tiab] OR synthes*[tiab]) AND (search*[tiab] OR database*[tiab] OR data-base*[tiab]))) OR metasynthes*[tiab] OR meta-synthes*[tiab])* **OR** ("Randomized Controlled Trial"[Publication Type] OR random*[tiab] OR pragmatic clinical trial*[tiab] OR practical clinical trial*[tiab] OR non-inferiority trial*[tiab] OR noninferiority trial*[tiab] OR superiority trial*[tiab] OR equivalence clinical trial*[tiab]) NOT (("Animals"[Mesh]) OR "Models, Animal"[Mesh] NOT humans[mh]) NOT (letter[pt] OR comment[pt] OR editorial[pt]) **OR ("Comparative Study"[Publication Type] OR "comparison"[tiab] OR "comparative"[tiab] OR "compar*"[tiab] OR "Epidemiologic studies"[mesh:noexp] OR "case control studies"[mesh] OR "cohort studies"[mesh] OR "Controlled Before-After Studies"[mesh] OR "Case control"[tw] OR cohort*[tw] OR "Cohort analy*"[tw] OR "Follow up stud*"[tw] OR "observational stud*"[tw] OR Longitudinal[tw] OR Retrospective*[tw] OR prospective*[tw] OR consecutive*[tw] OR Cross sectional[tw] OR "Cross-sectional studies"[mesh] OR "historically controlled study"[mesh] OR "interrupted time series analysis"[mesh]) OR** ("life cycle assess*"[tw] OR "life cycle assessment"[tw] OR "life cycle inventory"[tw] OR "LCA"[tw] OR "LCAs"[tw] OR "life cycle inventory"[tw] OR "life cycle inventories"[tw])**)**

**Embase (OVID)**

**(**(exp "Recycling"/ OR "reusable biopsy forceps".mp OR "reusable drape".mp OR "reusable drapes".mp OR "reusable equipment".mp OR "reusable flexible optical scope".mp OR "reusable flexible optical scopes".mp OR "reusable forceps".mp OR "reusable laryngeal mask".mp OR "reusable laryngeal masks".mp OR "reusable medical equipment".mp OR "reusable medical equipment packaging".mp OR "reusable scissor".mp OR "reusable scissors".mp OR "reusable scope".mp OR "reusable scopes".mp OR "reusable sharps container".mp OR "reusable sharps containers".mp OR "reusable specula".mp OR "reusable speculum".mp OR "reusable surgical attire".mp OR "reusable surgical attires".mp OR "reusable surgical clamp".mp OR "reusable surgical clamps".mp OR "reusable surgical clip".mp OR "reusable surgical clips".mp OR "reusable surgical kit".mp OR "reusable surgical kits".mp OR "reusable suture anchor".mp OR "reusable suture anchors".mp OR "reusable trocar".mp OR "reusable trocars".mp OR "reusable ureteroscope".mp OR "reusable ureteroscopes".mp OR "recyclable biopsy forceps".mp OR "recyclable drape".mp OR "recyclable drapes".mp OR "recyclable equipment".mp OR "recyclable flexible optical scope".mp OR "recyclable flexible optical scopes".mp OR "recyclable forceps".mp OR "recyclable laryngeal mask".mp OR "recyclable laryngeal masks".mp OR "recyclable medical equipment".mp OR "recyclable medical equipment packaging".mp OR "recyclable scissor".mp OR "recyclable scissors".mp OR "recyclable scope".mp OR "recyclable scopes".mp OR "recyclable sharps container".mp OR "recyclable sharps containers".mp OR "recyclable specula".mp OR "recyclable speculum".mp OR "recyclable surgical attire".mp OR "recyclable surgical attires".mp OR "recyclable surgical clamp".mp OR "recyclable surgical clamps".mp OR "recyclable surgical clip".mp OR "recyclable surgical clips".mp OR "recyclable surgical kit".mp OR "recyclable surgical kits".mp OR "recyclable suture anchor".mp OR "recyclable suture anchors".mp OR "recyclable trocar".mp OR "recyclable trocars".mp OR "recyclable ureteroscope".mp OR "recyclable ureteroscopes".mp OR ((exp "Recycling"/ OR "reusables".mp OR "reusable".mp OR "reusabl*".mp OR "reuse".mp OR "re usables".mp OR "re usable".mp OR "re usabl*".mp OR "re use".mp OR "recyclable".mp OR "recyclables".mp OR "recycl*".mp OR "re cyclable".mp OR "re cycl*".mp OR "reprocess".mp OR "reprocessing".mp OR "reprocess*".mp) AND (exp "biopsy forceps"/ OR "biopsy forceps".mp OR exp "Surgical Drape"/ OR "drape".mp OR "drapes".mp OR exp "Surgical Equipment"/ OR "equipment".mp OR "flexible optical scope".mp OR "flexible optical scopes".mp OR exp "Forceps"/ OR "forceps".mp OR exp "Laryngeal Mask"/ OR "laryngeal mask".mp OR "laryngeal masks".mp OR exp "medical Device"/ OR "medical equipment".mp OR "medical equipment packaging".mp OR exp "Scissors"/ OR "scissor".mp OR "scissors".mp OR exp "Endoscope"/ OR "scopes".mp OR "Endoscopes".mp OR "Endoscope".mp OR "sharps container"/ OR "sharps container".mp OR "sharps containers".mp OR "sharps bin".mp OR "sharps bins".mp OR "specula".mp OR "speculum".mp OR exp "Surgical Attire"/ OR "surgical attire".mp OR "surgical attires".mp OR "Surgical Shoe Covers".mp OR "Surgical Shoe Cover".mp OR "Surgical Gowns".mp OR "Surgical Gown".mp OR exp "Mask"/ OR "Mask".mp OR "Masks".mp OR "Respirators".mp OR "Respirator".mp OR exp "clamp"/ OR "surgical clamp".mp OR "surgical clamps".mp OR exp "Clip"/ OR "surgical clip".mp OR "surgical clips".mp OR "Forcep".mp OR "Forceps".mp OR "Surgical Clamp".mp OR "Surgical Clamps".mp OR exp "Surgical Hook"/ OR "Surgical Hook".mp OR "Surgical Hooks".mp OR "Surgical Instrument".mp OR "Surgical Instruments".mp OR "Surgical Plug".mp OR "Surgical Plugs".mp OR "Surgical Scissor".mp OR "Surgical Scissors".mp OR "Surgical Valve".mp OR "Surgical Valves".mp OR "Tantalum Clip".mp OR "Tantalum Clips".mp OR exp "Stapler"/ OR "Surgical Staplers".mp OR "Surgical Stapler".mp OR "surgical kit".mp OR "surgical kits".mp OR exp "Suture Anchor"/ OR "suture anchor".mp OR "suture anchors".mp OR "Bone Anchor".mp OR "Bone Anchors".mp OR exp "Trocar"/ OR "trocar".mp OR "trocars".mp OR exp "Ureteroscope"/ OR "ureteroscope".mp OR "ureteroscopes".mp OR exp "Spectacles"/ OR "band aid".mp OR "band aids".mp OR "bandaid".mp OR "bandaids".mp OR "cap".mp OR "caps".mp OR "cover material".mp OR "cover materials".mp OR "covering material".mp OR "covering materials".mp OR "Eyeglasses".mp OR "glasses".mp OR ex "Glove"/ OR "glove".mp OR "gloves".mp OR exp "Surgical Gown"/ OR exp "Patient Gown"/ OR "gown".mp OR "gowns".mp OR "packing material".mp OR "packing materials".mp OR "patch".mp OR "patches".mp OR "spectacles".mp OR "surgery instrument".mp OR "surgery instruments".mp OR "surgical instrument".mp OR "surgical instruments".mp OR "medical device".mp OR "medical devices".mp OR "surgical device".mp OR "surgical devices".mp))) AND (exp "Disposable Equipment"/ OR "disposable biopsy forceps".mp OR "disposable drape".mp OR "disposable drapes".mp OR "disposable equipment".mp OR "disposable flexible optical scope".mp OR "disposable flexible optical scopes".mp OR "disposable laryngeal mask".mp OR "disposable laryngeal masks".mp OR "disposable medical equipment packaging".mp OR "disposable scissor".mp OR "disposable scissors".mp OR "disposable scope".mp OR "disposable scopes".mp OR "disposable sharps container".mp OR "disposable sharps containers".mp OR "disposable specula".mp OR "disposable speculum".mp OR "disposable surgical attire".mp OR "disposable surgical clamp".mp OR "disposable surgical clamps".mp OR "disposable surgical clip".mp OR "disposable surgical clips".mp OR "disposable surgical kit".mp OR "disposable surgical kits".mp OR "disposable suture anchor".mp OR "disposable suture anchors".mp OR "disposable trocar".mp OR "disposable trocars".mp OR "disposable ureteroscope".mp OR "disposable ureteroscopes".mp OR "single use biopsy forceps".mp OR "single use drape".mp OR "single use drapes".mp OR "single use equipment".mp OR "single use flexible optical scope".mp OR "single use flexible optical scopes".mp OR "single use laryngeal mask".mp OR "single use laryngeal masks".mp OR "single use medical equipment packaging".mp OR "single use scissor".mp OR "single use scissors".mp OR "single use scope".mp OR "single use scopes".mp OR "single use sharps container".mp OR "single use sharps containers".mp OR "single use specula".mp OR "single use speculum".mp OR "single use surgical attire".mp OR "single use surgical clamp".mp OR "single use surgical clamps".mp OR "single use surgical clip".mp OR "single use surgical clips".mp OR "single use surgical kit".mp OR "single use surgical kits".mp OR "single use suture anchor".mp OR "single use suture anchors".mp OR "single use trocar".mp OR "single use trocars".mp OR "single use ureteroscope".mp OR "single use ureteroscopes".mp OR ((exp "Disposable Equipment"/ OR "disposable".mp OR "disposables".mp OR "dispos*".mp OR "single use".mp OR "single use*".mp OR "singleuse".mp OR "singleuse*".mp OR "single usage".mp OR "single usage*".mp) AND (exp "biopsy forceps"/ OR "biopsy forceps".mp OR exp "Surgical Drape"/ OR "drape".mp OR "drapes".mp OR exp "Surgical Equipment"/ OR "equipment".mp OR "flexible optical scope".mp OR "flexible optical scopes".mp OR exp "Forceps"/ OR "forceps".mp OR exp "Laryngeal Mask"/ OR "laryngeal mask".mp OR "laryngeal masks".mp OR exp "medical Device"/ OR "medical equipment".mp OR "medical equipment packaging".mp OR exp "Scissors"/ OR "scissor".mp OR "scissors".mp OR exp "Endoscope"/ OR "scopes".mp OR "Endoscopes".mp OR "Endoscope".mp OR "sharps container"/ OR "sharps container".mp OR "sharps containers".mp OR "sharps bin".mp OR "sharps bins".mp OR "specula".mp OR "speculum".mp OR exp "Surgical Attire"/ OR "surgical attire".mp OR "surgical attires".mp OR "Surgical Shoe Covers".mp OR "Surgical Shoe Cover".mp OR "Surgical Gowns".mp OR "Surgical Gown".mp OR exp "Mask"/ OR "Mask".mp OR "Masks".mp OR "Respirators".mp OR "Respirator".mp OR exp "clamp"/ OR "surgical clamp".mp OR "surgical clamps".mp OR exp "Clip"/ OR "surgical clip".mp OR "surgical clips".mp OR "Forcep".mp OR "Forceps".mp OR "Surgical Clamp".mp OR "Surgical Clamps".mp OR exp "Surgical Hook"/ OR "Surgical Hook".mp OR "Surgical Hooks".mp OR "Surgical Instrument".mp OR "Surgical Instruments".mp OR "Surgical Plug".mp OR "Surgical Plugs".mp OR "Surgical Scissor".mp OR "Surgical Scissors".mp OR "Surgical Valve".mp OR "Surgical Valves".mp OR "Tantalum Clip".mp OR "Tantalum Clips".mp OR exp "Stapler"/ OR "Surgical Staplers".mp OR "Surgical Stapler".mp OR "surgical kit".mp OR "surgical kits".mp OR exp "Suture Anchor"/ OR "suture anchor".mp OR "suture anchors".mp OR "Bone Anchor".mp OR "Bone Anchors".mp OR exp "Trocar"/ OR "trocar".mp OR "trocars".mp OR exp "Ureteroscope"/ OR "ureteroscope".mp OR "ureteroscopes".mp OR exp "Spectacles"/ OR "band aid".mp OR "band aids".mp OR "bandaid".mp OR "bandaids".mp OR "cap".mp OR "caps".mp OR "cover material".mp OR "cover materials".mp OR "covering material".mp OR "covering materials".mp OR "Eyeglasses".mp OR "glasses".mp OR ex "Glove"/ OR "glove".mp OR "gloves".mp OR exp "Surgical Gown"/ OR exp "Patient Gown"/ OR "gown".mp OR "gowns".mp OR "packing material".mp OR "packing materials".mp OR "patch".mp OR "patches".mp OR "spectacles".mp OR "surgery instrument".mp OR "surgery instruments".mp OR "surgical instrument".mp OR "surgical instruments".mp OR "medical device".mp OR "medical devices".mp OR "surgical device".mp OR "surgical devices".mp))) AND ("Carbon Footprint"/ OR "carbon footprint".mp OR "carbon footprint*".mp OR exp "Climate Change"/ OR "climate change".mp OR "CO2 emission".mp OR "CO2 emissions".mp OR "CO2 footprint".mp OR "CO2 footprint*".mp OR exp "environmental protection"/ OR "conservation of natural resources".mp OR "environmental protection".mp OR "ecological footprint".mp OR "ecological footprint*".mp OR "ecological sustainability".mp OR exp "environmental impact"/ OR "environmental impact".mp OR "environmental impact*".mp OR "environmental impacts".mp OR "environmental pollut*".mp OR exp "pollution"/ OR "environmental pollution".mp OR "environmental sustainab*".mp OR "environmental sustainability"/ OR "environmental sustainability".mp OR "Global Warming"/ OR "Global Warming".mp OR "Greenhouse Effect"/ OR "greenhouse effect*".mp OR "greenhouse effects".mp OR "greenhouse gas emission".mp OR "greenhouse gas emissions".mp OR "Greenhouse Gas"/ OR "greening".mp OR "hospital waste".mp OR "life cycle assessment"/ OR "environmental impact assessment"/ OR "life cycle assess*".mp OR "life cycle assessment".mp OR "LCA".mp OR "LCAs".mp OR "life cycle inventory".mp OR "life cycle inventories".mp OR exp "Waste Disposal"/ OR exp "Hospital Waste"/ OR "medical waste".mp OR "Rising Sea Level".mp OR "Rising Sea Levels".mp OR "Sea Level Rise"/ OR "Sea Level Rise".mp OR "sustainability"**.mp** OR "Waste Disposal".mp OR "waste water recycling"/ OR exp "Waste Disposal"/ OR exp "Waste Management"/ OR "Plastic overuse".mp OR "Green surgery".mp OR "Emission reduction".mp OR "Emission reduction strategy".mp OR "air pollution control"/ OR "air pollution control".mp **OR "Environment"/ OR "Environmental*".mp** OR "acidification"/ OR "soil acidification"/ OR "ocean acidification"/ OR "acidification".mp OR "Acidification potential".mp OR "AP in kg SO2 equivalents".mp OR "eco-efficiency".mp OR "ecoefficiency".mp OR "eco-efficien*".mp OR "ecoefficien*".mp OR "ecotoxicity"/ OR "ecotoxicity".mp OR "ecotoxic*".mp OR "eco toxicity".mp OR "eco toxic*".mp OR "EP in kg PO4 equivalent".mp OR exp "Eutrophication"/ OR "eutrophication".mp OR "Eutrophication potential".mp OR "FAETP in kg DCB equivalent".mp OR "Freshwater Aquatic Ecotoxicity Potential".mp OR "GWP in kg CO2 equivalents".mp OR "H+ moles equivalents".mp OR "HTTP in kg Dichlorobenzene equivalent".mp OR "Human Toxicity Potential".mp OR "kg 2.4-D equivalents".mp OR "kg CFC-11 equivalent".mp OR "kg N equivalents".mp OR "kg NOx equivalents".mp OR "life cycle analysis".mp OR "ozone depletion".mp OR "Photochemical Ozone Depletion Potential".mp OR "POCP in kg ethane equivalent".mp OR "smog".mp OR exp "Waste"/ OR "waste".mp OR "wastes".mp OR "Ozone Depletion"/ OR "Smog"/ OR "Greenhouse Gases".mp OR "Greenhouse Gas".mp OR "SO2 equiv*".mp OR "CO2 equiva*".mp OR "CFC-11 equiv*".mp OR "N equiv*".mp OR exp "Biodiversity"/ OR "Biodiversity".mp OR "Climatic change".mp OR "Green deal".mp OR "preservation of natural resources".mp OR "Refuse Disposal".mp OR exp "Wastewater"/ OR "Waste Water".mp OR "Wastewater".mp OR exp "Water Management"/ OR "Water Purification".mp OR (("plastic*".mp OR "microplastic*") AND ("soop".mp OR "soup".mp OR "pollution".mp OR "overuse".mp OR "contamination".mp)) OR "Sustainable Development"/ OR "Sustainable Development".mp OR (("Plastic".mp OR "plastics".mp) AND "overuse".mp) OR ("hydrogen*".mp AND "moles".mp AND "equiv*".mp) OR ("Dichlorobenzen*".mp AND "equiv*".mp) OR ("2,4-D".mp AND "equiv*".mp) OR ("NOx".mp AND "equiv*".mp) OR ("ethane".mp AND "equiv*".mp) OR ("PO4".mp AND "equiv*".mp) OR ("DCB".mp AND "equiv*".mp) OR ("sustainability".mp AND ("environment*".mp OR "carbon".mp)) OR (("Carbon Dioxide"/ OR "Carbon Dioxide".mp OR "CO2".mp) AND ("pollution".mp OR "emission".mp OR "emissions".mp OR "waste".mp OR "environment".mp OR "environmental*".mp OR "footprint".mp OR "footprint*".mp OR "sustainable".mp OR "hazard".mp OR "hazard*".mp))) NOT (conference review or conference abstract).pt AND (2000 OR 2001 OR 2002 OR 2003 OR 2004 OR 2005 OR 2006 OR 2007 OR 2008 OR 2009 OR 2010 OR 2011 OR 2012 OR 2013 OR 2014 OR 2015 OR 2016 OR 2017 OR 2018 OR 2019 OR 2020 OR 2021 OR 2022).yr) AND **(***(exp "meta analysis"/ OR exp "meta analysis (topic)"/ OR metaanaly*.ti,ab OR "meta analy*".ti,ab OR metanaly*.ti,ab OR "systematic review"/ OR "cochrane database of systematic reviews".jn OR prisma.ti,ab OR prospero.ti,ab OR (((systemati* OR scoping OR umbrella OR "structured literature") ADJ3 (review* OR overview*)).ti,ab) OR ((systemic* ADJ1 review*).ti,ab) OR (((systemati* OR literature OR database* OR "data base*") ADJ10 search*).ti,ab) OR (((structured OR comprehensive* OR systemic*) ADJ3 search*).ti,ab) OR (((literature ADJ3 review*).ti,ab) AND (search*.ti,ab OR database*.ti,ab OR "data base*".ti,ab)) OR (("data extraction".ti,ab OR "data source*".ti,ab) AND "study selection".ti,ab) OR ("search strategy".ti,ab AND "selection criteria".ti,ab) OR ("data source*".ti,ab AND "data synthesis".ti,ab) OR medline.ab OR pubmed.ab OR embase.ab OR cochrane.ab OR (((critical OR rapid) ADJ2 (review* OR overview* OR synthes*)).ti) OR ((((critical* OR rapid*) ADJ3 (review* OR overview* OR synthes*)).ab) AND (search*.ab OR database*.ab OR "data base*".ab)) OR metasynthes*.ti,ab OR "meta synthes*".ti,ab)* OR (exp "clinical trial"/ OR exp "randomization"/ OR exp "single blind procedure"/ OR exp "double blind procedure"/ OR exp "crossover procedure"/ OR exp "placebo"/ OR exp "prospective study"/ OR rct.ti,ab OR random*.ti,ab OR "single blind".ti,ab OR "randomised controlled trial".ti,ab OR exp "randomized controlled trial"/ OR placebo*.ti,ab) **OR (exp "Comparative Study"/ OR "comparison".ti,ab OR "comparative".ti,ab OR "compar*".ti,ab OR "major clinical study"/ OR "clinical study"/ OR "case control study"/ OR "family study"/ OR "longitudinal study"/ OR "retrospective study"/ OR "prospective study"/ OR "cohort analysis"/ OR cohort*.ti,ab OR (("case control" ADJ1 (study OR studies)).ti,ab) OR (("follow up" ADJ1 (study OR studies)).ti,ab) OR (observational ADJ1 (study OR studies)) OR ((epidemiologic ADJ1 (study OR studies)).ti,ab) OR (("cross sectional" ADJ1 (study OR studies)).ti,ab)) OR** ("life cycle assessment"/ OR "environmental impact assessment"/ OR "life cycle assess*".mp OR "life cycle assessment".mp OR "life cycle inventory".mp OR "LCA".mp OR "LCAs".mp OR "life cycle inventory".mp OR "life cycle inventories".mp)**)**

**Web of Science**

((ti=("Equipment Reuse" OR "reusable biopsy forceps" OR "reusable drape" OR "reusable drapes" OR "reusable equipment" OR "reusable flexible optical scope" OR "reusable flexible optical scopes" OR "reusable forceps" OR "reusable laryngeal mask" OR "reusable laryngeal masks" OR "reusable medical equipment" OR "reusable medical equipment packaging" OR "reusable scissor" OR "reusable scissors" OR "reusable scope" OR "reusable scopes" OR "reusable sharps container" OR "reusable sharps containers" OR "reusable specula" OR "reusable speculum" OR "reusable surgical attire" OR "reusable surgical attires" OR "reusable surgical clamp" OR "reusable surgical clamps" OR "reusable surgical clip" OR "reusable surgical clips" OR "reusable surgical kit" OR "reusable surgical kits" OR "reusable suture anchor" OR "reusable suture anchors" OR "reusable trocar" OR "reusable trocars" OR "reusable ureteroscope" OR "reusable ureteroscopes" OR "recyclable biopsy forceps" OR "recyclable drape" OR "recyclable drapes" OR "recyclable equipment" OR "recyclable flexible optical scope" OR "recyclable flexible optical scopes" OR "recyclable forceps" OR "recyclable laryngeal mask" OR "recyclable laryngeal masks" OR "recyclable medical equipment" OR "recyclable medical equipment packaging" OR "recyclable scissor" OR "recyclable scissors" OR "recyclable scope" OR "recyclable scopes" OR "recyclable sharps container" OR "recyclable sharps containers" OR "recyclable specula" OR "recyclable speculum" OR "recyclable surgical attire" OR "recyclable surgical attires" OR "recyclable surgical clamp" OR "recyclable surgical clamps" OR "recyclable surgical clip" OR "recyclable surgical clips" OR "recyclable surgical kit" OR "recyclable surgical kits" OR "recyclable suture anchor" OR "recyclable suture anchors" OR "recyclable trocar" OR "recyclable trocars" OR "recyclable ureteroscope" OR "recyclable ureteroscopes" OR (("Equipment Reuse" OR "reusables" OR "reusable" OR "reusabl*" OR "reuse" OR "re usables" OR "re usable" OR "re usabl*" OR "re use" OR "recyclable" OR "recyclables" OR "recycl*" OR "re cyclable" OR "re cycl*" OR "Recycling" OR "reprocess" OR "reprocessing" OR "reprocess*") AND ("biopsy forceps" OR "Surgical Drapes" OR "drape" OR "drapes" OR "Surgical Equipment" OR "equipment" OR "flexible optical scope" OR "flexible optical scopes" OR "forceps" OR "Laryngeal Masks" OR "laryngeal mask" OR "laryngeal masks" OR "medical equipment" OR "medical equipment packaging" OR "scissor" OR "scissors" OR "Endoscopes" OR "scopes" OR "Endoscopes" OR "Endoscope" OR "sharps container" OR "sharps containers" OR "sharps bin" OR "sharps bins" OR "specula" OR "speculum" OR "Surgical Attire" OR "surgical attire" OR "surgical attires" OR "Surgical Shoe Covers" OR "Surgical Shoe Cover" OR "Surgical Gowns" OR "Surgical Gown" OR "Masks" OR "Mask" OR "Masks" OR "Respirators" OR "Respirator" OR "Surgical Instruments" OR "surgical clamp" OR "surgical clamps" OR "surgical clip" OR "surgical clips" OR "Forcep" OR "Forceps" OR "Surgical Clamp" OR "Surgical Clamps" OR "Surgical Hook" OR "Surgical Hooks" OR "Surgical Instrument" OR "Surgical Instruments" OR "Surgical Plug" OR "Surgical Plugs" OR "Surgical Scissor" OR "Surgical Scissors" OR "Surgical Valve" OR "Surgical Valves" OR "Tantalum Clip" OR "Tantalum Clips" OR "Surgical Staplers" OR "Surgical Stapler" OR "surgical kit" OR "surgical kits" OR "Suture Anchors" OR "suture anchor" OR "suture anchors" OR "Bone Anchor" OR "Bone Anchors" OR "trocar" OR "trocars" OR "Ureteroscopes" OR "ureteroscope" OR "ureteroscopes" OR "Eyeglasses" OR "band aid" OR "band aids" OR "bandaid" OR "bandaids" OR "cap" OR "caps" OR "cover material" OR "cover materials" OR "covering material" OR "covering materials" OR "Eyeglasses" OR "glasses" OR "glove" OR "gloves" OR "gown" OR "gowns" OR "packing material" OR "packing materials" OR "patch" OR "patches" OR "spectacles" OR "surgery instrument" OR "surgery instruments" OR "surgical instrument" OR "surgical instruments" OR "surgery instrument" OR "surgery instruments" OR "surgical instrument" OR "surgical instruments" OR "medical device" OR "medical devices" OR "surgical device" OR "surgical devices"))) OR ab=("Equipment Reuse" OR "reusable biopsy forceps" OR "reusable drape" OR "reusable drapes" OR "reusable equipment" OR "reusable flexible optical scope" OR "reusable flexible optical scopes" OR "reusable forceps" OR "reusable laryngeal mask" OR "reusable laryngeal masks" OR "reusable medical equipment" OR "reusable medical equipment packaging" OR "reusable scissor" OR "reusable scissors" OR "reusable scope" OR "reusable scopes" OR "reusable sharps container" OR "reusable sharps containers" OR "reusable specula" OR "reusable speculum" OR "reusable surgical attire" OR "reusable surgical attires" OR "reusable surgical clamp" OR "reusable surgical clamps" OR "reusable surgical clip" OR "reusable surgical clips" OR "reusable surgical kit" OR "reusable surgical kits" OR "reusable suture anchor" OR "reusable suture anchors" OR "reusable trocar" OR "reusable trocars" OR "reusable ureteroscope" OR "reusable ureteroscopes" OR "recyclable biopsy forceps" OR "recyclable drape" OR "recyclable drapes" OR "recyclable equipment" OR "recyclable flexible optical scope" OR "recyclable flexible optical scopes" OR "recyclable forceps" OR "recyclable laryngeal mask" OR "recyclable laryngeal masks" OR "recyclable medical equipment" OR "recyclable medical equipment packaging" OR "recyclable scissor" OR "recyclable scissors" OR "recyclable scope" OR "recyclable scopes" OR "recyclable sharps container" OR "recyclable sharps containers" OR "recyclable specula" OR "recyclable speculum" OR "recyclable surgical attire" OR "recyclable surgical attires" OR "recyclable surgical clamp" OR "recyclable surgical clamps" OR "recyclable surgical clip" OR "recyclable surgical clips" OR "recyclable surgical kit" OR "recyclable surgical kits" OR "recyclable suture anchor" OR "recyclable suture anchors" OR "recyclable trocar" OR "recyclable trocars" OR "recyclable ureteroscope" OR "recyclable ureteroscopes" OR (("Equipment Reuse" OR "reusables" OR "reusable" OR "reusabl*" OR "reuse" OR "re usables" OR "re usable" OR "re usabl*" OR "re use" OR "recyclable" OR "recyclables" OR "recycl*" OR "re cyclable" OR "re cycl*" OR "Recycling" OR "reprocess" OR "reprocessing" OR "reprocess*") AND ("biopsy forceps" OR "Surgical Drapes" OR "drape" OR "drapes" OR "Surgical Equipment" OR "equipment" OR "flexible optical scope" OR "flexible optical scopes" OR "forceps" OR "Laryngeal Masks" OR "laryngeal mask" OR "laryngeal masks" OR "medical equipment" OR "medical equipment packaging" OR "scissor" OR "scissors" OR "Endoscopes" OR "scopes" OR "Endoscopes" OR "Endoscope" OR "sharps container" OR "sharps containers" OR "sharps bin" OR "sharps bins" OR "specula" OR "speculum" OR "Surgical Attire" OR "surgical attire" OR "surgical attires" OR "Surgical Shoe Covers" OR "Surgical Shoe Cover" OR "Surgical Gowns" OR "Surgical Gown" OR "Masks" OR "Mask" OR "Masks" OR "Respirators" OR "Respirator" OR "Surgical Instruments" OR "surgical clamp" OR "surgical clamps" OR "surgical clip" OR "surgical clips" OR "Forcep" OR "Forceps" OR "Surgical Clamp" OR "Surgical Clamps" OR "Surgical Hook" OR "Surgical Hooks" OR "Surgical Instrument" OR "Surgical Instruments" OR "Surgical Plug" OR "Surgical Plugs" OR "Surgical Scissor" OR "Surgical Scissors" OR "Surgical Valve" OR "Surgical Valves" OR "Tantalum Clip" OR "Tantalum Clips" OR "Surgical Staplers" OR "Surgical Stapler" OR "surgical kit" OR "surgical kits" OR "Suture Anchors" OR "suture anchor" OR "suture anchors" OR "Bone Anchor" OR "Bone Anchors" OR "trocar" OR "trocars" OR "Ureteroscopes" OR "ureteroscope" OR "ureteroscopes" OR "Eyeglasses" OR "band aid" OR "band aids" OR "bandaid" OR "bandaids" OR "cap" OR "caps" OR "cover material" OR "cover materials" OR "covering material" OR "covering materials" OR "Eyeglasses" OR "glasses" OR "glove" OR "gloves" OR "gown" OR "gowns" OR "packing material" OR "packing materials" OR "patch" OR "patches" OR "spectacles" OR "surgery instrument" OR "surgery instruments" OR "surgical instrument" OR "surgical instruments" OR "surgery instrument" OR "surgery instruments" OR "surgical instrument" OR "surgical instruments" OR "medical device" OR "medical devices" OR "surgical device" OR "surgical devices")))) AND (ti=("Disposable Equipment" OR "disposable biopsy forceps" OR "disposable drape" OR "disposable drapes" OR "disposable equipment" OR "disposable flexible optical scope" OR "disposable flexible optical scopes" OR "disposable laryngeal mask" OR "disposable laryngeal masks" OR "disposable medical equipment packaging" OR "disposable scissor" OR "disposable scissors" OR "disposable scope" OR "disposable scopes" OR "disposable sharps container" OR "disposable sharps containers" OR "disposable specula" OR "disposable speculum" OR "disposable surgical attire" OR "disposable surgical clamp" OR "disposable surgical clamps" OR "disposable surgical clip" OR "disposable surgical clips" OR "disposable surgical kit" OR "disposable surgical kits" OR "disposable suture anchor" OR "disposable suture anchors" OR "disposable trocar" OR "disposable trocars" OR "disposable ureteroscope" OR "disposable ureteroscopes" OR "single use biopsy forceps" OR "single use drape" OR "single use drapes" OR "single use equipment" OR "single use flexible optical scope" OR "single use flexible optical scopes" OR "single use laryngeal mask" OR "single use laryngeal masks" OR "single use medical equipment packaging" OR "single use scissor" OR "single use scissors" OR "single use scope" OR "single use scopes" OR "single use sharps container" OR "single use sharps containers" OR "single use specula" OR "single use speculum" OR "single use surgical attire" OR "single use surgical clamp" OR "single use surgical clamps" OR "single use surgical clip" OR "single use surgical clips" OR "single use surgical kit" OR "single use surgical kits" OR "single use suture anchor" OR "single use suture anchors" OR "single use trocar" OR "single use trocars" OR "single use ureteroscope" OR "single use ureteroscopes" OR (("Disposable Equipment" OR "disposable" OR "disposables" OR "dispos*" OR "single use" OR "single use*" OR "singleuse" OR "singleuse*" OR "single usage" OR "single usage*") AND ("biopsy forceps" OR "Surgical Drapes" OR "drape" OR "drapes" OR "Surgical Equipment" OR "equipment" OR "flexible optical scope" OR "flexible optical scopes" OR "forceps" OR "Laryngeal Masks" OR "laryngeal mask" OR "laryngeal masks" OR "medical equipment" OR "medical equipment packaging" OR "scissor" OR "scissors" OR "Endoscopes" OR "scopes" OR "Endoscopes" OR "Endoscope" OR "sharps container" OR "sharps containers" OR "sharps bin" OR "sharps bins" OR "specula" OR "speculum" OR "Surgical Attire" OR "surgical attire" OR "surgical attires" OR "Surgical Shoe Covers" OR "Surgical Shoe Cover" OR "Surgical Gowns" OR "Surgical Gown" OR "Masks" OR "Mask" OR "Masks" OR "Respirators" OR "Respirator" OR "Surgical Instruments" OR "surgical clamp" OR "surgical clamps" OR "surgical clip" OR "surgical clips" OR "Forcep" OR "Forceps" OR "Surgical Clamp" OR "Surgical Clamps" OR "Surgical Hook" OR "Surgical Hooks" OR "Surgical Instrument" OR "Surgical Instruments" OR "Surgical Plug" OR "Surgical Plugs" OR "Surgical Scissor" OR "Surgical Scissors" OR "Surgical Valve" OR "Surgical Valves" OR "Tantalum Clip" OR "Tantalum Clips" OR "Surgical Staplers" OR "Surgical Stapler" OR "surgical kit" OR "surgical kits" OR "Suture Anchors" OR "suture anchor" OR "suture anchors" OR "Bone Anchor" OR "Bone Anchors" OR "trocar" OR "trocars" OR "Ureteroscopes" OR "ureteroscope" OR "ureteroscopes" OR "Eyeglasses" OR "band aid" OR "band aids" OR "bandaid" OR "bandaids" OR "cap" OR "caps" OR "cover material" OR "cover materials" OR "covering material" OR "covering materials" OR "Eyeglasses" OR "glasses" OR "glove" OR "gloves" OR "gown" OR "gowns" OR "packing material" OR "packing materials" OR "patch" OR "patches" OR "spectacles" OR "surgery instrument" OR "surgery instruments" OR "surgical instrument" OR "surgical instruments" OR "surgery instrument" OR "surgery instruments" OR "surgical instrument" OR "surgical instruments" OR "medical device" OR "medical devices" OR "surgical device" OR "surgical devices"))) OR ab=("Disposable Equipment" OR "disposable biopsy forceps" OR "disposable drape" OR "disposable drapes" OR "disposable equipment" OR "disposable flexible optical scope" OR "disposable flexible optical scopes" OR "disposable laryngeal mask" OR "disposable laryngeal masks" OR "disposable medical equipment packaging" OR "disposable scissor" OR "disposable scissors" OR "disposable scope" OR "disposable scopes" OR "disposable sharps container" OR "disposable sharps containers" OR "disposable specula" OR "disposable speculum" OR "disposable surgical attire" OR "disposable surgical clamp" OR "disposable surgical clamps" OR "disposable surgical clip" OR "disposable surgical clips" OR "disposable surgical kit" OR "disposable surgical kits" OR "disposable suture anchor" OR "disposable suture anchors" OR "disposable trocar" OR "disposable trocars" OR "disposable ureteroscope" OR "disposable ureteroscopes" OR "single use biopsy forceps" OR "single use drape" OR "single use drapes" OR "single use equipment" OR "single use flexible optical scope" OR "single use flexible optical scopes" OR "single use laryngeal mask" OR "single use laryngeal masks" OR "single use medical equipment packaging" OR "single use scissor" OR "single use scissors" OR "single use scope" OR "single use scopes" OR "single use sharps container" OR "single use sharps containers" OR "single use specula" OR "single use speculum" OR "single use surgical attire" OR "single use surgical clamp" OR "single use surgical clamps" OR "single use surgical clip" OR "single use surgical clips" OR "single use surgical kit" OR "single use surgical kits" OR "single use suture anchor" OR "single use suture anchors" OR "single use trocar" OR "single use trocars" OR "single use ureteroscope" OR "single use ureteroscopes" OR (("Disposable Equipment" OR "disposable" OR "disposables" OR "dispos*" OR "single use" OR "single use*" OR "singleuse" OR "singleuse*" OR "single usage" OR "single usage*") AND ("biopsy forceps" OR "Surgical Drapes" OR "drape" OR "drapes" OR "Surgical Equipment" OR "equipment" OR "flexible optical scope" OR "flexible optical scopes" OR "forceps" OR "Laryngeal Masks" OR "laryngeal mask" OR "laryngeal masks" OR "medical equipment" OR "medical equipment packaging" OR "scissor" OR "scissors" OR "Endoscopes" OR "scopes" OR "Endoscopes" OR "Endoscope" OR "sharps container" OR "sharps containers" OR "sharps bin" OR "sharps bins" OR "specula" OR "speculum" OR "Surgical Attire" OR "surgical attire" OR "surgical attires" OR "Surgical Shoe Covers" OR "Surgical Shoe Cover" OR "Surgical Gowns" OR "Surgical Gown" OR "Masks" OR "Mask" OR "Masks" OR "Respirators" OR "Respirator" OR "Surgical Instruments" OR "surgical clamp" OR "surgical clamps" OR "surgical clip" OR "surgical clips" OR "Forcep" OR "Forceps" OR "Surgical Clamp" OR "Surgical Clamps" OR "Surgical Hook" OR "Surgical Hooks" OR "Surgical Instrument" OR "Surgical Instruments" OR "Surgical Plug" OR "Surgical Plugs" OR "Surgical Scissor" OR "Surgical Scissors" OR "Surgical Valve" OR "Surgical Valves" OR "Tantalum Clip" OR "Tantalum Clips" OR "Surgical Staplers" OR "Surgical Stapler" OR "surgical kit" OR "surgical kits" OR "Suture Anchors" OR "suture anchor" OR "suture anchors" OR "Bone Anchor" OR "Bone Anchors" OR "trocar" OR "trocars" OR "Ureteroscopes" OR "ureteroscope" OR "ureteroscopes" OR "Eyeglasses" OR "band aid" OR "band aids" OR "bandaid" OR "bandaids" OR "cap" OR "caps" OR "cover material" OR "cover materials" OR "covering material" OR "covering materials" OR "Eyeglasses" OR "glasses" OR "glove" OR "gloves" OR "gown" OR "gowns" OR "packing material" OR "packing materials" OR "patch" OR "patches" OR "spectacles" OR "surgery instrument" OR "surgery instruments" OR "surgical instrument" OR "surgical instruments" OR "surgery instrument" OR "surgery instruments" OR "surgical instrument" OR "surgical instruments" OR "medical device" OR "medical devices" OR "surgical device" OR "surgical devices")))) AND (TS=("Carbon Footprint" OR "carbon footprint" OR "carbon footprint*" OR "Climate Change" OR "climate change" OR "CO2 emission" OR "CO2 emissions" OR "CO2 footprint" OR "CO2 footprint*" OR "environmental protection" OR "conservation of natural resources" OR "environmental protection" OR "ecological footprint" OR "ecological footprint*" OR "ecological sustainability" OR "environmental impact" OR "environmental impact" OR "environmental impact*" OR "environmental impacts" OR "environmental pollut*" OR "pollution" OR "environmental pollution" OR "environmental sustainab*" OR "environmental sustainability" OR "environmental sustainability" OR "Global Warming" OR "Global Warming" OR "Greenhouse Effect" OR "greenhouse effect*" OR "greenhouse effects" OR "greenhouse gas emission" OR "greenhouse gas emissions" OR "Greenhouse Gas" OR "greening" OR "hospital waste" OR "life cycle assessment" OR "environmental impact assessment" OR "life cycle assess*" OR "life cycle assessment" OR "LCA" OR "LCAs" OR "life cycle inventory" OR "life cycle inventories" OR "Waste Disposal" OR "Hospital Waste" OR "medical waste" OR "Rising Sea Level" OR "Rising Sea Levels" OR "Sea Level Rise" OR "Sea Level Rise" OR "Waste Disposal" OR "waste water recycling" OR "Waste Disposal" OR "Waste Management" OR "Plastic overuse" OR "Green surgery" OR "Emission reduction" OR "Emission reduction strategy" OR "air pollution control" OR "air pollution control" OR "acidification" OR "soil acidification" OR "ocean acidification" OR "acidification" OR "Acidification potential" OR "AP in kg SO2 equivalents" OR "eco-efficiency" OR "ecoefficiency" OR "eco-efficien*" OR "ecoefficien*" OR "ecotoxicity" OR "ecotoxicity" OR "ecotoxic*" OR "eco toxicity" OR "eco toxic*" OR "EP in kg PO4 equivalent" OR "Eutrophication" OR "eutrophication" OR "Eutrophication potential" OR "FAETP in kg DCB equivalent" OR "Freshwater Aquatic Ecotoxicity Potential" OR "GWP in kg CO2 equivalents" OR "H+ moles equivalents" OR "HTTP in kg Dichlorobenzene equivalent" OR "Human Toxicity Potential" OR "kg 2.4-D equivalents" OR "kg CFC-11 equivalent" OR "kg N equivalents" OR "kg NOx equivalents" OR "life cycle analysis" OR "ozone depletion" OR "Photochemical Ozone Depletion Potential" OR "POCP in kg ethane equivalent" OR "smog" OR "Waste" OR "waste" OR "wastes" OR "Ozone Depletion" OR "Smog" OR "Greenhouse Gases" OR "Greenhouse Gas" OR "SO2 equiv*" OR "CO2 equiva*" OR "CFC-11 equiv*" OR "N equiv*" OR "Sustainable Development" OR "Sustainable Development" OR "Biodiversity" OR "Climatic change" OR "Green deal" OR "preservation of natural resources" OR "Refuse Disposal" OR "Waste Water" OR "Wastewater" OR "Water Purification" OR (("plastic*" OR "microplastic*") AND ("soop" OR "soup" OR "pollution" OR "overuse" OR "contamination")) OR (("Plastic" OR "plastics") AND "overuse") OR ("hydrogen*" AND "moles" AND "equiv*") OR ("Dichlorobenzen*" AND "equiv*") OR ("2,4-D" AND "equiv*") OR ("NOx" AND "equiv*") OR ("ethane" AND "equiv*") OR ("PO4" AND "equiv*") OR ("DCB" AND "equiv*") OR ("sustainability" AND ("environment*" OR "carbon")) OR (("Carbon Dioxide" OR "Carbon Dioxide" OR "CO2") AND ("pollution" OR "emission" OR "emissions" OR "waste" OR "environment" OR "environmental*" OR "footprint" OR "footprint*" OR "sustainable" OR "hazard" OR "hazard*"))) OR **TI=("environmental*" OR "sustainab*")** OR **AB=("environmental*" OR "sustainab*")** OR **AK=("environmental*" OR "sustainab*")**) NOT DT=(meeting abstract) AND PY=(2000 OR 2001 OR 2002 OR 2003 OR 2004 OR 2005 OR 2006 OR 2007 OR 2008 OR 2009 OR 2010 OR 2011 OR 2012 OR 2013 OR 2014 OR 2015 OR 2016 OR 2017 OR 2018 OR 2019 OR 2020 OR 2021 OR 2022)) AND **(**TI=**(***("Meta-Analysis" OR metaanaly* OR "meta-analy*" or metanaly* OR "Systematic Review" OR "Cochrane Database Syst Rev" OR "prisma" OR "preferred reporting items" OR "prospero" OR ((systemati* OR scoping OR umbrella OR "structured literature") NEAR/4 (review* OR overview*)) OR "systematic review*" OR "scoping review*" OR "umbrella review*" OR "structured literature review*" OR "systematic qualitative review*" OR "systematic quantitative review*" OR "systematic search and review" OR "systematized review" OR "systematised review" OR "systemic review" OR "systematic literature review*" OR "systematic integrative literature review*" OR "systematically review*" OR "scoping literature review*" OR "systematic critical review" OR "systematic integrative review*" OR "systematic evidence review" OR "Systematic integrative literature review*" OR "Systematic mixed studies review*" OR "Systematized literature review*" OR "Systematic overview*" OR "Systematic narrative review*" OR (("systemati*" OR "literature" OR "database*" OR "data-base*" OR "structured" OR "comprehensive*" OR "systemic*") NEAR/4 "search*") OR ("Literature" AND "review" AND ("database*" OR "data-base*" OR "search*")) OR (("data extraction" OR "data source*") AND "study selection") OR ("search strategy" AND "selection criteria") OR ("data source*" AND "data synthesis") OR "medline" OR "pubmed" OR "embase" OR "Cochrane" OR (("critical" OR "rapid") NEAR/4 ("review*" OR "overview*" OR "synthes*")) OR ((("critical*" OR "rapid*") NEAR/4 ("review*" OR "overview*" OR "synthes*") NEAR/4 ("search*" OR "database*" OR "data-base*"))) OR metasynthes* OR "meta-synthes*")* **OR** ("Randomized Controlled Trial" OR random* OR "RCT"OR "RCTs" OR "pragmatic clinical trial*" OR "practical clinical trial*" OR "non-inferiority trial*" OR "noninferiority trial*" OR "superiority trial*" OR "equivalence clinical trial*") **OR ("Comparative Study" OR "comparison" OR "comparative" OR "compar*" OR "major clinical study" OR "clinical study" OR "case control study" OR "family study" OR "longitudinal study" OR "retrospective study" OR "prospective study" OR "cohort analysis" OR cohort* OR (("case control" NEAR/1 (study OR studies))) OR (("follow up" NEAR/1 (study OR studies))) OR (observational NEAR/1 (study OR studies)) OR ((epidemiologic NEAR/1 (study OR studies))) OR (("cross sectional" NEAR/1 (study OR studies)))) OR** ("life cycle assess*" OR "life cycle assessment" OR "life cycle inventory" OR "LCA" OR "LCAs" OR "life cycle inventory" OR "life cycle inventories")**)** OR AB=**(***("Meta-Analysis" OR metaanaly* OR "meta-analy*" or metanaly* OR "Systematic Review" OR "Cochrane Database Syst Rev" OR "prisma" OR "preferred reporting items" OR "prospero" OR ((systemati* OR scoping OR umbrella OR "structured literature") NEAR/4 (review* OR overview*)) OR "systematic review*" OR "scoping review*" OR "umbrella review*" OR "structured literature review*" OR "systematic qualitative review*" OR "systematic quantitative review*" OR "systematic search and review" OR "systematized review" OR "systematised review" OR "systemic review" OR "systematic literature review*" OR "systematic integrative literature review*" OR "systematically review*" OR "scoping literature review*" OR "systematic critical review" OR "systematic integrative review*" OR "systematic evidence review" OR "Systematic integrative literature review*" OR "Systematic mixed studies review*" OR "Systematized literature review*" OR "Systematic overview*" OR "Systematic narrative review*" OR (("systemati*" OR "literature" OR "database*" OR "data-base*" OR "structured" OR "comprehensive*" OR "systemic*") NEAR/4 "search*") OR ("Literature" AND "review" AND ("database*" OR "data-base*" OR "search*")) OR (("data extraction" OR "data source*") AND "study selection") OR ("search strategy" AND "selection criteria") OR ("data source*" AND "data synthesis") OR "medline" OR "pubmed" OR "embase" OR "Cochrane" OR (("critical" OR "rapid") NEAR/4 ("review*" OR "overview*" OR "synthes*")) OR ((("critical*" OR "rapid*") NEAR/4 ("review*" OR "overview*" OR "synthes*") NEAR/4 ("search*" OR "database*" OR "data-base*"))) OR metasynthes* OR "meta-synthes*")* **OR** ("Randomized Controlled Trial" OR random* OR "RCT"OR "RCTs" OR "pragmatic clinical trial*" OR "practical clinical trial*" OR "non-inferiority trial*" OR "noninferiority trial*" OR "superiority trial*" OR "equivalence clinical trial*") **OR ("Comparative Study" OR "comparison" OR "comparative" OR "compar*" OR "major clinical study" OR "clinical study" OR "case control study" OR "family study" OR "longitudinal study" OR "retrospective study" OR "prospective study" OR "cohort analysis" OR cohort* OR (("case control" NEAR/1 (study OR studies))) OR (("follow up" NEAR/1 (study OR studies))) OR (observational NEAR/1 (study OR studies)) OR ((epidemiologic NEAR/1 (study OR studies))) OR (("cross sectional" NEAR/1 (study OR studies)))) OR** ("life cycle assess*" OR "life cycle assessment" OR "life cycle inventory" OR "LCA" OR "LCAs" OR "life cycle inventory" OR "life cycle inventories")**))**

**Cochrane Library**

("Equipment Reuse" OR "reusable biopsy forceps" OR "reusable drape" OR "reusable drapes" OR "reusable equipment" OR "reusable flexible optical scope" OR "reusable flexible optical scopes" OR "reusable forceps" OR "reusable laryngeal mask" OR "reusable laryngeal masks" OR "reusable medical equipment" OR "reusable medical equipment packaging" OR "reusable scissor" OR "reusable scissors" OR "reusable scope" OR "reusable scopes" OR "reusable sharps container" OR "reusable sharps containers" OR "reusable specula" OR "reusable speculum" OR "reusable surgical attire" OR "reusable surgical attires" OR "reusable surgical clamp" OR "reusable surgical clamps" OR "reusable surgical clip" OR "reusable surgical clips" OR "reusable surgical kit" OR "reusable surgical kits" OR "reusable suture anchor" OR "reusable suture anchors" OR "reusable trocar" OR "reusable trocars" OR "reusable ureteroscope" OR "reusable ureteroscopes" OR "recyclable biopsy forceps" OR "recyclable drape" OR "recyclable drapes" OR "recyclable equipment" OR "recyclable flexible optical scope" OR "recyclable flexible optical scopes" OR "recyclable forceps" OR "recyclable laryngeal mask" OR "recyclable laryngeal masks" OR "recyclable medical equipment" OR "recyclable medical equipment packaging" OR "recyclable scissor" OR "recyclable scissors" OR "recyclable scope" OR "recyclable scopes" OR "recyclable sharps container" OR "recyclable sharps containers" OR "recyclable specula" OR "recyclable speculum" OR "recyclable surgical attire" OR "recyclable surgical attires" OR "recyclable surgical clamp" OR "recyclable surgical clamps" OR "recyclable surgical clip" OR "recyclable surgical clips" OR "recyclable surgical kit" OR "recyclable surgical kits" OR "recyclable suture anchor" OR "recyclable suture anchors" OR "recyclable trocar" OR "recyclable trocars" OR "recyclable ureteroscope" OR "recyclable ureteroscopes" OR (("Equipment Reuse" OR "reusables" OR "reusable" OR "reusabl*" OR "reuse" OR "re usables" OR "re usable" OR "re usabl*" OR "re use" OR "recyclable" OR "recyclables" OR "recycl*" OR "re cyclable" OR "re cycl*" OR "Recycling" OR "reprocess" OR "reprocessing" OR "reprocess*") AND ("biopsy forceps" OR "Surgical Drapes" OR "drape" OR "drapes" OR "Surgical Equipment" OR "equipment" OR "flexible optical scope" OR "flexible optical scopes" OR "forceps" OR "Laryngeal Masks" OR "laryngeal mask" OR "laryngeal masks" OR "medical equipment" OR "medical equipment packaging" OR "scissor" OR "scissors" OR "Endoscopes" OR "scopes" OR "Endoscopes" OR "Endoscope" OR "sharps container" OR "sharps containers" OR "sharps bin" OR "sharps bins" OR "specula" OR "speculum" OR "Surgical Attire" OR "surgical attire" OR "surgical attires" OR "Surgical Shoe Covers" OR "Surgical Shoe Cover" OR "Surgical Gowns" OR "Surgical Gown" OR "Masks" OR "Mask" OR "Masks" OR "Respirators" OR "Respirator" OR "Surgical Instruments" OR "surgical clamp" OR "surgical clamps" OR "surgical clip" OR "surgical clips" OR "Forcep" OR "Forceps" OR "Surgical Clamp" OR "Surgical Clamps" OR "Surgical Hook" OR "Surgical Hooks" OR "Surgical Instrument" OR "Surgical Instruments" OR "Surgical Plug" OR "Surgical Plugs" OR "Surgical Scissor" OR "Surgical Scissors" OR "Surgical Valve" OR "Surgical Valves" OR "Tantalum Clip" OR "Tantalum Clips" OR "Surgical Staplers" OR "Surgical Stapler" OR "surgical kit" OR "surgical kits" OR "Suture Anchors" OR "suture anchor" OR "suture anchors" OR "Bone Anchor" OR "Bone Anchors" OR "trocar" OR "trocars" OR "Ureteroscopes" OR "ureteroscope" OR "ureteroscopes" OR "Eyeglasses" OR "band aid" OR "band aids" OR "bandaid" OR "bandaids" OR "cap" OR "caps" OR "cover material" OR "cover materials" OR "covering material" OR "covering materials" OR "Eyeglasses" OR "glasses" OR "glove" OR "gloves" OR "gown" OR "gowns" OR "packing material" OR "packing materials" OR "patch" OR "patches" OR "spectacles" OR "surgery instrument" OR "surgery instruments" OR "surgical instrument" OR "surgical instruments" OR "surgery instrument" OR "surgery instruments" OR "surgical instrument" OR "surgical instruments" OR "medical device" OR "medical devices" OR "surgical device" OR "surgical devices"))):ti,ab,kw AND ("Disposable Equipment" OR "disposable biopsy forceps" OR "disposable drape" OR "disposable drapes" OR "disposable equipment" OR "disposable flexible optical scope" OR "disposable flexible optical scopes" OR "disposable laryngeal mask" OR "disposable laryngeal masks" OR "disposable medical equipment packaging" OR "disposable scissor" OR "disposable scissors" OR "disposable scope" OR "disposable scopes" OR "disposable sharps container" OR "disposable sharps containers" OR "disposable specula" OR "disposable speculum" OR "disposable surgical attire" OR "disposable surgical clamp" OR "disposable surgical clamps" OR "disposable surgical clip" OR "disposable surgical clips" OR "disposable surgical kit" OR "disposable surgical kits" OR "disposable suture anchor" OR "disposable suture anchors" OR "disposable trocar" OR "disposable trocars" OR "disposable ureteroscope" OR "disposable ureteroscopes" OR "single use biopsy forceps" OR "single use drape" OR "single use drapes" OR "single use equipment" OR "single use flexible optical scope" OR "single use flexible optical scopes" OR "single use laryngeal mask" OR "single use laryngeal masks" OR "single use medical equipment packaging" OR "single use scissor" OR "single use scissors" OR "single use scope" OR "single use scopes" OR "single use sharps container" OR "single use sharps containers" OR "single use specula" OR "single use speculum" OR "single use surgical attire" OR "single use surgical clamp" OR "single use surgical clamps" OR "single use surgical clip" OR "single use surgical clips" OR "single use surgical kit" OR "single use surgical kits" OR "single use suture anchor" OR "single use suture anchors" OR "single use trocar" OR "single use trocars" OR "single use ureteroscope" OR "single use ureteroscopes" OR (("Disposable Equipment" OR "disposable" OR "disposables" OR "dispos*" OR "single use" OR "single use*" OR "singleuse" OR "singleuse*" OR "single usage" OR "single usage*") AND ("biopsy forceps" OR "Surgical Drapes" OR "drape" OR "drapes" OR "Surgical Equipment" OR "equipment" OR "flexible optical scope" OR "flexible optical scopes" OR "forceps" OR "Laryngeal Masks" OR "laryngeal mask" OR "laryngeal masks" OR "medical equipment" OR "medical equipment packaging" OR "scissor" OR "scissors" OR "Endoscopes" OR "scopes" OR "Endoscopes" OR "Endoscope" OR "sharps container" OR "sharps containers" OR "sharps bin" OR "sharps bins" OR "specula" OR "speculum" OR "Surgical Attire" OR "surgical attire" OR "surgical attires" OR "Surgical Shoe Covers" OR "Surgical Shoe Cover" OR "Surgical Gowns" OR "Surgical Gown" OR "Masks" OR "Mask" OR "Masks" OR "Respirators" OR "Respirator" OR "Surgical Instruments" OR "surgical clamp" OR "surgical clamps" OR "surgical clip" OR "surgical clips" OR "Forcep" OR "Forceps" OR "Surgical Clamp" OR "Surgical Clamps" OR "Surgical Hook" OR "Surgical Hooks" OR "Surgical Instrument" OR "Surgical Instruments" OR "Surgical Plug" OR "Surgical Plugs" OR "Surgical Scissor" OR "Surgical Scissors" OR "Surgical Valve" OR "Surgical Valves" OR "Tantalum Clip" OR "Tantalum Clips" OR "Surgical Staplers" OR "Surgical Stapler" OR "surgical kit" OR "surgical kits" OR "Suture Anchors" OR "suture anchor" OR "suture anchors" OR "Bone Anchor" OR "Bone Anchors" OR "trocar" OR "trocars" OR "Ureteroscopes" OR "ureteroscope" OR "ureteroscopes" OR "Eyeglasses" OR "band aid" OR "band aids" OR "bandaid" OR "bandaids" OR "cap" OR "caps" OR "cover material" OR "cover materials" OR "covering material" OR "covering materials" OR "Eyeglasses" OR "glasses" OR "glove" OR "gloves" OR "gown" OR "gowns" OR "packing material" OR "packing materials" OR "patch" OR "patches" OR "spectacles" OR "surgery instrument" OR "surgery instruments" OR "surgical instrument" OR "surgical instruments" OR "surgery instrument" OR "surgery instruments" OR "surgical instrument" OR "surgical instruments" OR "medical device" OR "medical devices" OR "surgical device" OR "surgical devices"))):ti,ab,kw AND

(("Carbon Footprint" OR "carbon footprint" OR "carbon footprint*" OR "Climate Change" OR "climate change" OR "CO2 emission" OR "CO2 emissions" OR "CO2 footprint" OR "CO2 footprint*" OR "environmental protection" OR "conservation of natural resources" OR "environmental protection" OR "ecological footprint" OR "ecological footprint*" OR "ecological sustainability" OR "environmental impact" OR "environmental impact" OR "environmental impact*" OR "environmental impacts" OR "environmental pollut*" OR "pollution" OR "environmental pollution" OR "environmental sustainab*" OR "environmental sustainability" OR "environmental sustainability" OR "Global Warming" OR "Global Warming" OR "Greenhouse Effect" OR "greenhouse effect*" OR "greenhouse effects" OR "greenhouse gas emission" OR "greenhouse gas emissions" OR "Greenhouse Gas" OR "greening" OR "hospital waste" OR "life cycle assessment" OR "environmental impact assessment" OR "life cycle assessment" OR "environmental impact assessment" OR "life cycle assess*" OR "life cycle assessment" OR "LCA" OR "LCAs" OR "life cycle inventory" OR "life cycle inventories" OR "Waste Disposal" OR "Hospital Waste" OR "medical waste" OR "Rising Sea Level" OR "Rising Sea Levels" OR "Sea Level Rise" OR "Sea Level Rise" OR "Waste Disposal" OR "waste water recycling" OR "Waste Disposal" OR "Waste Management" OR "Plastic overuse" OR "Green surgery" OR "Emission reduction" OR "Emission reduction strategy" OR "air pollution control" OR "air pollution control" OR "acidification" OR "soil acidification" OR "ocean acidification" OR "acidification" OR "Acidification potential" OR "AP in kg SO2 equivalents" OR "eco-efficiency" OR "ecoefficiency" OR "eco-efficien*" OR "ecoefficien*" OR "ecotoxicity" OR "ecotoxicity" OR "ecotoxic*" OR "eco toxicity" OR "eco toxic*" OR "EP in kg PO4 equivalent" OR "Eutrophication" OR "eutrophication" OR "Eutrophication potential" OR "FAETP in kg DCB equivalent" OR "Freshwater Aquatic Ecotoxicity Potential" OR "GWP in kg CO2 equivalents" OR "H+ moles equivalents" OR "HTTP in kg Dichlorobenzene equivalent" OR "Human Toxicity Potential" OR "kg 2.4-D equivalents" OR "kg CFC-11 equivalent" OR "kg N equivalents" OR "kg NOx equivalents" OR "life cycle analysis" OR "ozone depletion" OR "Photochemical Ozone Depletion Potential" OR "POCP in kg ethane equivalent" OR "smog" OR "Waste" OR "waste" OR "wastes" OR "Ozone Depletion" OR "Smog" OR "Greenhouse Gases" OR "Greenhouse Gas" OR "SO2 equiv*" OR "CO2 equiva*" OR "CFC-11 equiv*" OR "N equiv*" OR "Biodiversity" OR "Climatic change" OR "Green deal" OR "preservation of natural resources" OR "Refuse Disposal" OR "Waste Water" OR "Wastewater" OR "Water Purification" OR (("plastic*" OR "microplastic*") AND ("soop" OR "soup" OR "pollution" OR "overuse" OR "contamination")) OR "Sustainable Development" OR "Sustainable Development" OR (("Plastic" OR "plastics") AND "overuse") OR ("hydrogen*" AND "moles" AND "equiv*") OR ("Dichlorobenzen*" AND "equiv*") OR ("2,4-D" AND "equiv*") OR ("NOx" AND "equiv*") OR ("ethane" AND "equiv*") OR ("PO4" AND "equiv*") OR ("DCB" AND "equiv*") OR ("sustainability" AND ("environment*" OR "carbon")) OR (("Carbon Dioxide" OR "Carbon Dioxide" OR "CO2") AND ("pollution" OR "emission" OR "emissions" OR "waste" OR "environment" OR "environmental*" OR "footprint" OR "footprint*" OR "sustainable" OR "hazard" OR "hazard*"))):ti,ab,kw OR **("environmental" OR "sustainability"):ti,ab,kw**)

**Emcare (OVID)**

**(**(exp "Recycling"/ OR "reusable biopsy forceps".mp OR "reusable drape".mp OR "reusable drapes".mp OR "reusable equipment".mp OR "reusable flexible optical scope".mp OR "reusable flexible optical scopes".mp OR "reusable forceps".mp OR "reusable laryngeal mask".mp OR "reusable laryngeal masks".mp OR "reusable medical equipment".mp OR "reusable medical equipment packaging".mp OR "reusable scissor".mp OR "reusable scissors".mp OR "reusable scope".mp OR "reusable scopes".mp OR "reusable sharps container".mp OR "reusable sharps containers".mp OR "reusable specula".mp OR "reusable speculum".mp OR "reusable surgical attire".mp OR "reusable surgical attires".mp OR "reusable surgical clamp".mp OR "reusable surgical clamps".mp OR "reusable surgical clip".mp OR "reusable surgical clips".mp OR "reusable surgical kit".mp OR "reusable surgical kits".mp OR "reusable suture anchor".mp OR "reusable suture anchors".mp OR "reusable trocar".mp OR "reusable trocars".mp OR "reusable ureteroscope".mp OR "reusable ureteroscopes".mp OR "recyclable biopsy forceps".mp OR "recyclable drape".mp OR "recyclable drapes".mp OR "recyclable equipment".mp OR "recyclable flexible optical scope".mp OR "recyclable flexible optical scopes".mp OR "recyclable forceps".mp OR "recyclable laryngeal mask".mp OR "recyclable laryngeal masks".mp OR "recyclable medical equipment".mp OR "recyclable medical equipment packaging".mp OR "recyclable scissor".mp OR "recyclable scissors".mp OR "recyclable scope".mp OR "recyclable scopes".mp OR "recyclable sharps container".mp OR "recyclable sharps containers".mp OR "recyclable specula".mp OR "recyclable speculum".mp OR "recyclable surgical attire".mp OR "recyclable surgical attires".mp OR "recyclable surgical clamp".mp OR "recyclable surgical clamps".mp OR "recyclable surgical clip".mp OR "recyclable surgical clips".mp OR "recyclable surgical kit".mp OR "recyclable surgical kits".mp OR "recyclable suture anchor".mp OR "recyclable suture anchors".mp OR "recyclable trocar".mp OR "recyclable trocars".mp OR "recyclable ureteroscope".mp OR "recyclable ureteroscopes".mp OR ((exp "Recycling"/ OR "reusables".mp OR "reusable".mp OR "reusabl*".mp OR "reuse".mp OR "re usables".mp OR "re usable".mp OR "re usabl*".mp OR "re use".mp OR "recyclable".mp OR "recyclables".mp OR "recycl*".mp OR "re cyclable".mp OR "re cycl*".mp OR "reprocess".mp OR "reprocessing".mp OR "reprocess*".mp) AND (exp "biopsy forceps"/ OR "biopsy forceps".mp OR exp "Surgical Drape"/ OR "drape".mp OR "drapes".mp OR exp "Surgical Equipment"/ OR "equipment".mp OR "flexible optical scope".mp OR "flexible optical scopes".mp OR exp "Forceps"/ OR "forceps".mp OR exp "Laryngeal Mask"/ OR "laryngeal mask".mp OR "laryngeal masks".mp OR exp "medical Device"/ OR "medical equipment".mp OR "medical equipment packaging".mp OR exp "Scissors"/ OR "scissor".mp OR "scissors".mp OR exp "Endoscope"/ OR "scopes".mp OR "Endoscopes".mp OR "Endoscope".mp OR "sharps container"/ OR "sharps container".mp OR "sharps containers".mp OR "sharps bin".mp OR "sharps bins".mp OR "specula".mp OR "speculum".mp OR exp "Surgical Attire"/ OR "surgical attire".mp OR "surgical attires".mp OR "Surgical Shoe Covers".mp OR "Surgical Shoe Cover".mp OR "Surgical Gowns".mp OR "Surgical Gown".mp OR exp "Mask"/ OR "Mask".mp OR "Masks".mp OR "Respirators".mp OR "Respirator".mp OR exp "clamp"/ OR "surgical clamp".mp OR "surgical clamps".mp OR exp "Clip"/ OR "surgical clip".mp OR "surgical clips".mp OR "Forcep".mp OR "Forceps".mp OR "Surgical Clamp".mp OR "Surgical Clamps".mp OR exp "Surgical Hook"/ OR "Surgical Hook".mp OR "Surgical Hooks".mp OR "Surgical Instrument".mp OR "Surgical Instruments".mp OR "Surgical Plug".mp OR "Surgical Plugs".mp OR "Surgical Scissor".mp OR "Surgical Scissors".mp OR "Surgical Valve".mp OR "Surgical Valves".mp OR "Tantalum Clip".mp OR "Tantalum Clips".mp OR exp "Stapler"/ OR "Surgical Staplers".mp OR "Surgical Stapler".mp OR "surgical kit".mp OR "surgical kits".mp OR exp "Suture Anchor"/ OR "suture anchor".mp OR "suture anchors".mp OR "Bone Anchor".mp OR "Bone Anchors".mp OR exp "Trocar"/ OR "trocar".mp OR "trocars".mp OR exp "Ureteroscope"/ OR "ureteroscope".mp OR "ureteroscopes".mp OR exp "Spectacles"/ OR "band aid".mp OR "band aids".mp OR "bandaid".mp OR "bandaids".mp OR "cap".mp OR "caps".mp OR "cover material".mp OR "cover materials".mp OR "covering material".mp OR "covering materials".mp OR "Eyeglasses".mp OR "glasses".mp OR ex "Glove"/ OR "glove".mp OR "gloves".mp OR exp "Surgical Gown"/ OR exp "Patient Gown"/ OR "gown".mp OR "gowns".mp OR "packing material".mp OR "packing materials".mp OR "patch".mp OR "patches".mp OR "spectacles".mp OR "surgery instrument".mp OR "surgery instruments".mp OR "surgical instrument".mp OR "surgical instruments".mp OR "medical device".mp OR "medical devices".mp OR "surgical device".mp OR "surgical devices".mp))) AND (exp "Disposable Equipment"/ OR "disposable biopsy forceps".mp OR "disposable drape".mp OR "disposable drapes".mp OR "disposable equipment".mp OR "disposable flexible optical scope".mp OR "disposable flexible optical scopes".mp OR "disposable laryngeal mask".mp OR "disposable laryngeal masks".mp OR "disposable medical equipment packaging".mp OR "disposable scissor".mp OR "disposable scissors".mp OR "disposable scope".mp OR "disposable scopes".mp OR "disposable sharps container".mp OR "disposable sharps containers".mp OR "disposable specula".mp OR "disposable speculum".mp OR "disposable surgical attire".mp OR "disposable surgical clamp".mp OR "disposable surgical clamps".mp OR "disposable surgical clip".mp OR "disposable surgical clips".mp OR "disposable surgical kit".mp OR "disposable surgical kits".mp OR "disposable suture anchor".mp OR "disposable suture anchors".mp OR "disposable trocar".mp OR "disposable trocars".mp OR "disposable ureteroscope".mp OR "disposable ureteroscopes".mp OR "single use biopsy forceps".mp OR "single use drape".mp OR "single use drapes".mp OR "single use equipment".mp OR "single use flexible optical scope".mp OR "single use flexible optical scopes".mp OR "single use laryngeal mask".mp OR "single use laryngeal masks".mp OR "single use medical equipment packaging".mp OR "single use scissor".mp OR "single use scissors".mp OR "single use scope".mp OR "single use scopes".mp OR "single use sharps container".mp OR "single use sharps containers".mp OR "single use specula".mp OR "single use speculum".mp OR "single use surgical attire".mp OR "single use surgical clamp".mp OR "single use surgical clamps".mp OR "single use surgical clip".mp OR "single use surgical clips".mp OR "single use surgical kit".mp OR "single use surgical kits".mp OR "single use suture anchor".mp OR "single use suture anchors".mp OR "single use trocar".mp OR "single use trocars".mp OR "single use ureteroscope".mp OR "single use ureteroscopes".mp OR ((exp "Disposable Equipment"/ OR "disposable".mp OR "disposables".mp OR "dispos*".mp OR "single use".mp OR "single use*".mp OR "singleuse".mp OR "singleuse*".mp OR "single usage".mp OR "single usage*".mp) AND (exp "biopsy forceps"/ OR "biopsy forceps".mp OR exp "Surgical Drape"/ OR "drape".mp OR "drapes".mp OR exp "Surgical Equipment"/ OR "equipment".mp OR "flexible optical scope".mp OR "flexible optical scopes".mp OR exp "Forceps"/ OR "forceps".mp OR exp "Laryngeal Mask"/ OR "laryngeal mask".mp OR "laryngeal masks".mp OR exp "medical Device"/ OR "medical equipment".mp OR "medical equipment packaging".mp OR exp "Scissors"/ OR "scissor".mp OR "scissors".mp OR exp "Endoscope"/ OR "scopes".mp OR "Endoscopes".mp OR "Endoscope".mp OR "sharps container"/ OR "sharps container".mp OR "sharps containers".mp OR "sharps bin".mp OR "sharps bins".mp OR "specula".mp OR "speculum".mp OR exp "Surgical Attire"/ OR "surgical attire".mp OR "surgical attires".mp OR "Surgical Shoe Covers".mp OR "Surgical Shoe Cover".mp OR "Surgical Gowns".mp OR "Surgical Gown".mp OR exp "Mask"/ OR "Mask".mp OR "Masks".mp OR "Respirators".mp OR "Respirator".mp OR exp "clamp"/ OR "surgical clamp".mp OR "surgical clamps".mp OR exp "Clip"/ OR "surgical clip".mp OR "surgical clips".mp OR "Forcep".mp OR "Forceps".mp OR "Surgical Clamp".mp OR "Surgical Clamps".mp OR exp "Surgical Hook"/ OR "Surgical Hook".mp OR "Surgical Hooks".mp OR "Surgical Instrument".mp OR "Surgical Instruments".mp OR "Surgical Plug".mp OR "Surgical Plugs".mp OR "Surgical Scissor".mp OR "Surgical Scissors".mp OR "Surgical Valve".mp OR "Surgical Valves".mp OR "Tantalum Clip".mp OR "Tantalum Clips".mp OR exp "Stapler"/ OR "Surgical Staplers".mp OR "Surgical Stapler".mp OR "surgical kit".mp OR "surgical kits".mp OR exp "Suture Anchor"/ OR "suture anchor".mp OR "suture anchors".mp OR "Bone Anchor".mp OR "Bone Anchors".mp OR exp "Trocar"/ OR "trocar".mp OR "trocars".mp OR exp "Ureteroscope"/ OR "ureteroscope".mp OR "ureteroscopes".mp OR exp "Spectacles"/ OR "band aid".mp OR "band aids".mp OR "bandaid".mp OR "bandaids".mp OR "cap".mp OR "caps".mp OR "cover material".mp OR "cover materials".mp OR "covering material".mp OR "covering materials".mp OR "Eyeglasses".mp OR "glasses".mp OR ex "Glove"/ OR "glove".mp OR "gloves".mp OR exp "Surgical Gown"/ OR exp "Patient Gown"/ OR "gown".mp OR "gowns".mp OR "packing material".mp OR "packing materials".mp OR "patch".mp OR "patches".mp OR "spectacles".mp OR "surgery instrument".mp OR "surgery instruments".mp OR "surgical instrument".mp OR "surgical instruments".mp OR "medical device".mp OR "medical devices".mp OR "surgical device".mp OR "surgical devices".mp))) AND ("Carbon Footprint"/ OR "carbon footprint".mp OR "carbon footprint*".mp OR exp "Climate Change"/ OR "climate change".mp OR "CO2 emission".mp OR "CO2 emissions".mp OR "CO2 footprint".mp OR "CO2 footprint*".mp OR exp "environmental protection"/ OR "conservation of natural resources".mp OR "environmental protection".mp OR "ecological footprint".mp OR "ecological footprint*".mp OR "ecological sustainability".mp OR exp "environmental impact"/ OR "environmental impact".mp OR "environmental impact*".mp OR "environmental impacts".mp OR "environmental pollut*".mp OR exp "pollution"/ OR "environmental pollution".mp OR "environmental sustainab*".mp OR "environmental sustainability"/ OR "environmental sustainability".mp OR "Global Warming"/ OR "Global Warming".mp OR "Greenhouse Effect"/ OR "greenhouse effect*".mp OR "greenhouse effects".mp OR "greenhouse gas emission".mp OR "greenhouse gas emissions".mp OR "Greenhouse Gas"/ OR "greening".mp OR "hospital waste".mp OR "life cycle assessment"/ OR "environmental impact assessment"/ OR "life cycle assess*".mp OR "life cycle assessment".mp OR "LCA".mp OR "LCAs".mp OR "life cycle inventory".mp OR "life cycle inventories".mp OR exp "Waste Disposal"/ OR exp "Hospital Waste"/ OR "medical waste".mp OR "Rising Sea Level".mp OR "Rising Sea Levels".mp OR "Sea Level Rise"/ OR "Sea Level Rise".mp OR "sustainability"**.mp** OR "Waste Disposal".mp OR "waste water recycling"/ OR exp "Waste Disposal"/ OR exp "Waste Management"/ OR "Plastic overuse".mp OR "Green surgery".mp OR "Emission reduction".mp OR "Emission reduction strategy".mp OR "air pollution control"/ OR "air pollution control".mp **OR "Environment"/ OR "Environmental*".mp** OR "acidification"/ OR "soil acidification"/ OR "ocean acidification"/ OR "acidification".mp OR "Acidification potential".mp OR "AP in kg SO2 equivalents".mp OR "eco-efficiency".mp OR "ecoefficiency".mp OR "eco-efficien*".mp OR "ecoefficien*".mp OR "ecotoxicity"/ OR "ecotoxicity".mp OR "ecotoxic*".mp OR "eco toxicity".mp OR "eco toxic*".mp OR "EP in kg PO4 equivalent".mp OR exp "Eutrophication"/ OR "eutrophication".mp OR "Eutrophication potential".mp OR "FAETP in kg DCB equivalent".mp OR "Freshwater Aquatic Ecotoxicity Potential".mp OR "GWP in kg CO2 equivalents".mp OR "H+ moles equivalents".mp OR "HTTP in kg Dichlorobenzene equivalent".mp OR "Human Toxicity Potential".mp OR "kg 2.4-D equivalents".mp OR "kg CFC-11 equivalent".mp OR "kg N equivalents".mp OR "kg NOx equivalents".mp OR "life cycle analysis".mp OR "ozone depletion".mp OR "Photochemical Ozone Depletion Potential".mp OR "POCP in kg ethane equivalent".mp OR "smog".mp OR exp "Waste"/ OR "waste".mp OR "wastes".mp OR "Ozone Depletion"/ OR "Smog"/ OR "Greenhouse Gases".mp OR "Greenhouse Gas".mp OR "SO2 equiv*".mp OR "CO2 equiva*".mp OR "CFC-11 equiv*".mp OR "N equiv*".mp OR exp "Biodiversity"/ OR "Biodiversity".mp OR "Climatic change".mp OR "Green deal".mp OR "preservation of natural resources".mp OR "Refuse Disposal".mp OR exp "Wastewater"/ OR "Waste Water".mp OR "Wastewater".mp OR exp "Water Management"/ OR "Water Purification".mp OR (("plastic*".mp OR "microplastic*") AND ("soop".mp OR "soup".mp OR "pollution".mp OR "overuse".mp OR "contamination".mp)) OR "Sustainable Development"/ OR "Sustainable Development".mp OR (("Plastic".mp OR "plastics".mp) AND "overuse".mp) OR ("hydrogen*".mp AND "moles".mp AND "equiv*".mp) OR ("Dichlorobenzen*".mp AND "equiv*".mp) OR ("2,4-D".mp AND "equiv*".mp) OR ("NOx".mp AND "equiv*".mp) OR ("ethane".mp AND "equiv*".mp) OR ("PO4".mp AND "equiv*".mp) OR ("DCB".mp AND "equiv*".mp) OR ("sustainability".mp AND ("environment*".mp OR "carbon".mp)) OR (("Carbon Dioxide"/ OR "Carbon Dioxide".mp OR "CO2".mp) AND ("pollution".mp OR "emission".mp OR "emissions".mp OR "waste".mp OR "environment".mp OR "environmental*".mp OR "footprint".mp OR "footprint*".mp OR "sustainable".mp OR "hazard".mp OR "hazard*".mp))) NOT (conference review or conference abstract).pt AND (2000 OR 2001 OR 2002 OR 2003 OR 2004 OR 2005 OR 2006 OR 2007 OR 2008 OR 2009 OR 2010 OR 2011 OR 2012 OR 2013 OR 2014 OR 2015 OR 2016 OR 2017 OR 2018 OR 2019 OR 2020 OR 2021 OR 2022).yr) AND **(***(exp "meta analysis"/ OR exp "meta analysis (topic)"/ OR metaanaly*.ti,ab OR "meta analy*".ti,ab OR metanaly*.ti,ab OR "systematic review"/ OR "cochrane database of systematic reviews".jn OR prisma.ti,ab OR prospero.ti,ab OR (((systemati* OR scoping OR umbrella OR "structured literature") ADJ3 (review* OR overview*)).ti,ab) OR ((systemic* ADJ1 review*).ti,ab) OR (((systemati* OR literature OR database* OR "data base*") ADJ10 search*).ti,ab) OR (((structured OR comprehensive* OR systemic*) ADJ3 search*).ti,ab) OR (((literature ADJ3 review*).ti,ab) AND (search*.ti,ab OR database*.ti,ab OR "data base*".ti,ab)) OR (("data extraction".ti,ab OR "data source*".ti,ab) AND "study selection".ti,ab) OR ("search strategy".ti,ab AND "selection criteria".ti,ab) OR ("data source*".ti,ab AND "data synthesis".ti,ab) OR medline.ab OR pubmed.ab OR embase.ab OR cochrane.ab OR (((critical OR rapid) ADJ2 (review* OR overview* OR synthes*)).ti) OR ((((critical* OR rapid*) ADJ3 (review* OR overview* OR synthes*)).ab) AND (search*.ab OR database*.ab OR "data base*".ab)) OR metasynthes*.ti,ab OR "meta synthes*".ti,ab)* OR (exp "clinical trial"/ OR exp "randomization"/ OR exp "single blind procedure"/ OR exp "double blind procedure"/ OR exp "crossover procedure"/ OR exp "placebo"/ OR exp "prospective study"/ OR rct.ti,ab OR random*.ti,ab OR "single blind".ti,ab OR "randomised controlled trial".ti,ab OR exp "randomized controlled trial"/ OR placebo*.ti,ab) **OR (exp "Comparative Study"/ OR "comparison".ti,ab OR "comparative".ti,ab OR "compar*".ti,ab OR "major clinical study"/ OR "clinical study"/ OR "case control study"/ OR "family study"/ OR "longitudinal study"/ OR "retrospective study"/ OR "prospective study"/ OR "cohort analysis"/ OR cohort*.ti,ab OR (("case control" ADJ1 (study OR studies)).ti,ab) OR (("follow up" ADJ1 (study OR studies)).ti,ab) OR (observational ADJ1 (study OR studies)) OR ((epidemiologic ADJ1 (study OR studies)).ti,ab) OR (("cross sectional" ADJ1 (study OR studies)).ti,ab)) OR** ("life cycle assessment"/ OR "environmental impact assessment"/ OR "life cycle assess*".mp OR "life cycle assessment".mp OR "life cycle inventory".mp OR "LCA".mp OR "LCAs".mp OR "life cycle inventory".mp OR "life cycle inventories".mp)**)**

### Topic 2: Disposables vs. reusables (PICO2)

(("reusable scissor"[tw] OR "reusable scissors"[tw] OR "reusable scope"[tw] OR "reusable scopes"[tw] OR "reusable specula"[tw] OR "reusable speculum"[tw] OR "reusable surgical kit"[tw] OR "reusable surgical kits"[tw] OR "reusable ureteroscope"[tw] OR "reusable ureteroscopes"[tw] OR "recyclable flexible optical scope"[tw] OR "recyclable flexible optical scopes"[tw] OR "recyclable scissor"[tw] OR "recyclable scissors"[tw] OR "recyclable scope"[tw] OR "recyclable scopes"[tw] OR "recyclable specula"[tw] OR "recyclable speculum"[tw] OR "recyclable surgical kit"[tw] OR "recyclable surgical kits"[tw] OR "recyclable ureteroscope"[tw] OR "recyclable ureteroscopes"[tw] OR (("Equipment Reuse"[mesh] OR "reusables"[tw] OR "reusable"[tw] OR "reusabl*"[tw] OR "reuse"[tw] OR "re usables"[tw] OR "re usable"[tw] OR "re usabl*"[tw] OR "re use"[tw] OR "recyclable"[tw] OR "recyclables"[tw] OR "recycl*"[tw] OR "re cyclable"[tw] OR "re cycl*"[tw] OR "Recycling"[Mesh] OR "reprocess"[tw] OR "reprocessing"[tw] OR "reprocess*"[tw]) AND ("flexible optical scope"[tw] OR "flexible optical scopes"[tw] OR "scissor"[tw] OR "scissors"[tw] OR "Endoscopes"[Mesh] OR "scopes"[tw] OR "Endoscopes"[tw] OR "Endoscope"[tw] OR "specula"[tw] OR "speculum"[tw] OR "Surgical Scissor"[tw] OR "Surgical Scissors"[tw] OR "Surgical Staplers"[tw] OR "Surgical Stapler"[tw] OR "Staplers"[tw] OR "Stapler"[tw] OR "Staples"[tw] OR "Staple"[tw] OR "Stapling"[tw] OR "Surgical Drill"[tw] OR "Surgical Drills"[tw] OR "Surgical Drilling"[tw] OR "Drill"[tw] OR "Drills"[tw] OR "Drilling"[tw] OR "surgical kit"[tw] OR "surgical kits"[tw] OR "surgery kit"[tw] OR "surgery kits"[tw] OR "Suture Anchors"[Mesh] OR "suture anchor"[tw] OR "suture anchors"[tw] OR "Bone Anchor"[tw] OR "Bone Anchors"[tw] OR "Ureteroscopes"[Mesh] OR "ureteroscope"[tw] OR "ureteroscopes"[tw] OR "kocher"[tw] OR "pincer"[tw] OR "pincers"[tw] OR "tweezer"[tw] OR "tweezers"[tw] OR "scalpel"[tw] OR "scalpels"[tw] OR "needle holder"[tw] OR "needle holders"[tw] OR "ligasure"[tw] OR "ligasur*"[tw] OR "harmonic"[tw] OR "harmonic*"[tw] OR "Angioscope"[tw] OR "Angioscopes"[tw] OR "Arthroscope"[tw] OR "Arthroscopes"[tw] OR "Bronchoscope"[tw] OR "Bronchoscopes"[tw] OR "Colonoscope"[tw] OR "Colonoscopes"[tw] OR "Colposcope"[tw] OR "Colposcopes"[tw] OR "Culdoscope"[tw] OR "Culdoscopes"[tw] OR "Cystoscope"[tw] OR "Cystoscopes"[tw] OR "Duodenoscope"[tw] OR "Duodenoscopes"[tw] OR "Esophagoscope"[tw] OR "Esophagoscopes"[tw] OR "Fetoscope"[tw] OR "Fetoscopes"[tw] OR "Gastroscope"[tw] OR "Gastroscopes"[tw] OR "Hysteroscope"[tw] OR "Hysteroscopes"[tw] OR "Laparoscope"[tw] OR "Laparoscopes"[tw] OR "Laryngoscope"[tw] OR "Laryngoscopes"[tw] OR "Mediastinoscope"[tw] OR "Mediastinoscopes"[tw] OR "Neuroendoscope"[tw] OR "Neuroendoscopes"[tw] OR "Proctoscope"[tw] OR "Proctoscopes"[tw] OR "Sigmoidoscope"[tw] OR "Sigmoidoscopes"[tw] OR "Thoracoscope"[tw] OR "Thoracoscopes"[tw] OR (("Menisci, Tibial"[mesh] OR "menisc*"[tw]) AND ("Suture Techniques"[tw] OR "sutur*"[tw]))))) AND ("disposable scissor"[tw] OR "disposable scissors"[tw] OR "disposable scope"[tw] OR "disposable scopes"[tw] OR "disposable specula"[tw] OR "disposable speculum"[tw] OR "single use scissor"[tw] OR "single use scissors"[tw] OR "single use scope"[tw] OR "single use scopes"[tw] OR (("Disposable Equipment"[Mesh] OR "disposable"[tw] OR "disposables"[tw] OR "dispos*"[tw] OR "single use"[tw] OR "single use*"[tw] OR "singleuse"[tw] OR "singleuse*"[tw] OR "single usage"[tw] OR "single usage*"[tw]) AND ("scissor"[tw] OR "scissors"[tw] OR "Endoscopes"[Mesh] OR "scopes"[tw] OR "Endoscopes"[tw] OR "Endoscope"[tw] OR "Surgical Scissor"[tw] OR "Surgical Scissors"[tw] OR "Surgical Staplers"[tw] OR "Surgical Stapler"[tw] OR "Surgery Staplers"[tw] OR "Surgery Stapler"[tw] OR "Staplers"[tw] OR "Stapler"[tw] OR "Stapling"[tw] OR "Surgical Drill"[tw] OR "Surgical Drills"[tw] OR "Surgical Drilling"[tw] OR "Drill"[tw] OR "Drills"[tw] OR "Drilling"[tw] OR "kocher"[tw] OR "pincer"[tw] OR "pincers"[tw] OR "tweezer"[tw] OR "tweezers"[tw] OR "scalpel"[tw] OR "scalpels"[tw] OR "needle holder"[tw] OR "needle holders"[tw] OR "ligasure"[tw] OR "ligasur*"[tw] OR "harmonic"[tw] OR "harmonic*"[tw] OR "Angioscope"[tw] OR "Angioscopes"[tw] OR "Arthroscope"[tw] OR "Arthroscopes"[tw] OR "Bronchoscope"[tw] OR "Bronchoscopes"[tw] OR "Colonoscope"[tw] OR "Colonoscopes"[tw] OR "Colposcope"[tw] OR "Colposcopes"[tw] OR "Culdoscope"[tw] OR "Culdoscopes"[tw] OR "Cystoscope"[tw] OR "Cystoscopes"[tw] OR "Duodenoscope"[tw] OR "Duodenoscopes"[tw] OR "Esophagoscope"[tw] OR "Esophagoscopes"[tw] OR "Fetoscope"[tw] OR "Fetoscopes"[tw] OR "Gastroscope"[tw] OR "Gastroscopes"[tw] OR "Hysteroscope"[tw] OR "Hysteroscopes"[tw] OR "Laparoscope"[tw] OR "Laparoscopes"[tw] OR "Laryngoscope"[tw] OR "Laryngoscopes"[tw] OR "Mediastinoscope"[tw] OR "Mediastinoscopes"[tw] OR "Neuroendoscope"[tw] OR "Neuroendoscopes"[tw] OR "Proctoscope"[tw] OR "Proctoscopes"[tw] OR "Sigmoidoscope"[tw] OR "Sigmoidoscopes"[tw] OR "Thoracoscope"[tw] OR "Thoracoscopes"[tw]))) AND ("Acidification potential"[tw] OR "acidification"[tw] OR "air pollution control"[tw] OR "AP in kg SO2 equivalents"[tw] OR "Biodiversity"[Mesh] OR "Biodiversity"[tw] OR "Carbon Footprint"[mesh] OR "carbon footprint"[tw] OR "carbon footprint*"[tw] OR "CFC-11 equiv*"[tw] OR "Climate Change"[Mesh] OR "climate change"[tw] OR "Climatic change"[tw] OR "CO2 emission"[tw] OR "CO2 emissions"[tw] OR "CO2 equiva*"[tw] OR "CO2 footprint"[tw] OR "CO2 footprint*"[tw] OR "conservation of natural resources"[mesh] OR "conservation of natural resources"[tw] OR "eco toxic*"[tw] OR "eco toxicity"[tw] OR "ecoefficien*"[tw] OR "eco-efficien*"[tw] OR "ecoefficiency"[tw] OR "eco-efficiency"[tw] OR "ecological footprint"[tw] OR "ecological footprint*"[tw] OR "ecological sustainability"[tw] OR "ecotoxic*"[tw] OR "ecotoxicity"[tw] OR "Emission reduction strategy"[tw] OR "Emission reduction"[tw] OR **"Environment"[Mesh:noexp]** OR "environmental impact"[tw] OR "environmental impact*"[tw] OR "environmental impacts"[tw] OR "environmental pollut*"[tw] OR "Environmental Pollution"[Mesh] OR "environmental pollution"[tw] OR "environmental protection"[tw] OR "environmental sustainab*"[tw] OR "environmental sustainability"[tw] OR **"Environmental*"[ti]** OR "EP in kg PO4 equivalent"[tw] OR "Eutrophication potential"[tw] OR "Eutrophication"[Mesh] OR "eutrophication"[tw] OR "FAETP in kg DCB equivalent"[tw] OR "Freshwater Aquatic Ecotoxicity Potential"[tw] OR "Global Warming"[mesh] OR "Global Warming"[tw] OR "Green deal"[tw] OR "Green surgery"[tw] OR "Greenhouse Effect"[mesh] OR "greenhouse effect*"[tw] OR "greenhouse effects"[tw] OR "greenhouse gas emission"[tw] OR "greenhouse gas emissions"[tw] OR "Greenhouse Gas"[tw] OR "Greenhouse Gases"[mesh] OR "Greenhouse Gases"[tw] OR "greening"[tw] OR "GWP in kg CO2 equivalents"[tw] OR "H+ moles equivalents"[tw] OR "hospital waste"[tw] OR "HTTP in kg Dichlorobenzene equivalent"[tw] OR "Human Toxicity Potential"[tw] OR "kg 2.4-D equivalents"[tw] OR "kg CFC-11 equivalent"[tw] OR "kg N equivalents"[tw] OR "kg NOx equivalents"[tw] OR "LCA"[tw] OR "LCAs"[tw] OR "life cycle analysis"[tw] OR "life cycle assess*"[tw] OR "life cycle assessment"[tw] OR "life cycle inventories"[tw] OR "life cycle inventory"[tw] OR "Medical Waste Disposal"[mesh] OR "Medical Waste"[mesh] OR "medical waste"[tw] OR "N equiv*"[tw] OR "Ozone Depletion"[Mesh] OR "ozone depletion"[tw] OR "Photochemical Ozone Depletion Potential"[tw] OR "Plastic overuse"[tw] OR "POCP in kg ethane equivalent"[tw] OR "preservation of natural resources"[tw] OR "Refuse Disposal"[Mesh] OR "Refuse Disposal"[tw] OR "Rising Sea Level"[tw] OR "Rising Sea Levels"[tw] OR "Sea Level Rise"[mesh] OR "Sea Level Rise"[tw] OR "Smog"[mesh] OR "smog"[tw] OR "SO2 equiv*"[tw] OR "sustainability"**[ti]** OR "Sustainable Development"[Mesh] OR "Sustainable Development"[tw] OR "Waste Disposal"[tw] OR "Waste Disposal, Fluid"[mesh] OR "Waste Management"[mesh] OR "Waste"[tw] OR "waste"[tw] OR "Waste Water"[Mesh] OR "Waste Water"[tw] OR "wastes"[tw] OR "Wastewater"[tw] OR "Water Purification"[Mesh] OR "Water Purification"[tw] OR (("plastic*"[tw] OR "microplastic*") AND ("soop"[tw] OR "soup"[tw] OR "pollution"[tw] OR "overuse"[tw] OR "contamination"[tw])) OR (("Plastic"[tw] OR "plastics"[tw]) AND "overuse"[tw]) OR ("hydrogen*"[tw] AND "moles"[tw] AND "equiv*"[tw]) OR ("Dichlorobenzen*"[tw] AND "equiv*"[tw]) OR ("2,4-D"[tw] AND "equiv*"[tw]) OR ("NOx"[tw] AND "equiv*"[tw]) OR ("ethane"[tw] AND "equiv*"[tw]) OR ("PO4"[tw] AND "equiv*"[tw]) OR ("DCB"[tw] AND "equiv*"[tw]) OR ("sustainability"[tw] AND ("environment*"[tw] OR "carbon"[tw])) OR (("Carbon Dioxide"[mesh] OR "Carbon Dioxide"[tw] OR "CO2"[tw]) AND ("pollution"[tw] OR "emission"[tw] OR "emissions"[tw] OR "waste"[tw] OR "environment"[tw] OR "environmental*"[tw] OR "footprint"[tw] OR "footprint*"[tw] OR "sustainable"[tw] OR "hazard"[tw] OR "hazard*"[tw]))) AND ("2000/01/01"[PDAT] : "3000/12/31"[PDAT])) AND **(***("Meta-Analysis"[Publication Type] OR "Meta-Analysis as Topic"[Mesh] OR metaanaly*[tiab] OR meta-analy*[tiab] or metanaly*[tiab] OR "Systematic Review"[Publication Type] OR systematic[sb] OR "Cochrane Database Syst Rev"[Journal] or prisma[tiab] OR preferred reporting items[tiab] OR prospero[tiab] OR ((systemati*[ti] OR scoping[ti] OR umbrella[ti] OR structured literature[ti]) AND (review*[ti] OR overview*[ti])) OR systematic review*[tiab] OR scoping review*[tiab] OR umbrella review*[tiab] OR structured literature review*[tiab] OR systematic qualitative review*[tiab] OR systematic quantitative review*[tiab] OR systematic search and review[tiab] OR systematized review[tiab] OR systematised review[tiab] OR systemic review[tiab] OR systematic literature review*[tiab] OR systematic integrative literature review*[tiab] OR systematically review*[tiab] OR scoping literature review*[tiab] OR systematic critical review[tiab] OR systematic integrative review*[tiab] OR systematic evidence review[tiab] OR Systematic integrative literature review*[tiab] OR Systematic mixed studies review*[tiab] OR Systematized literature review*[tiab] OR Systematic overview*[tiab] OR Systematic narrative review*[tiab] OR ((systemati*[tiab] OR literature[tiab] OR database*[tiab] OR data-base*[tiab] OR structured[tiab] OR comprehensive*[tiab] OR systemic*[tiab]) AND search*[tiab]) OR (Literature[ti] AND review[ti] AND (database*[tiab] OR data-base*[tiab] OR search*[tiab])) OR ((data extraction[tiab] OR data source*[tiab]) AND study selection[tiab]) OR (search strategy[tiab] AND selection criteria[tiab]) OR (data source*[tiab] AND data synthesis[tiab]) OR medline[tiab] OR pubmed[tiab] OR embase[tiab] OR Cochrane[tiab] OR ((critical[ti] OR rapid[ti]) AND (review*[ti] OR overview*[ti] OR synthes*[ti])) OR (((critical*[tiab] OR rapid*[tiab]) AND (review*[tiab] OR overview*[tiab] OR synthes*[tiab]) AND (search*[tiab] OR database*[tiab] OR data-base*[tiab]))) OR metasynthes*[tiab] OR meta-synthes*[tiab])* **OR** ("Randomized Controlled Trial"[Publication Type] OR random*[tiab] OR pragmatic clinical trial*[tiab] OR practical clinical trial*[tiab] OR non-inferiority trial*[tiab] OR noninferiority trial*[tiab] OR superiority trial*[tiab] OR equivalence clinical trial*[tiab]) NOT (("Animals"[Mesh]) OR "Models, Animal"[Mesh] NOT humans[mh]) NOT (letter[pt] OR comment[pt] OR editorial[pt]) **OR ("Comparative Study"[Publication Type] OR "comparison"[tiab] OR "comparative"[tiab] OR "compar*"[tiab] OR "Epidemiologic studies"[mesh:noexp] OR "case control studies"[mesh] OR "cohort studies"[mesh] OR "Controlled Before-After Studies"[mesh] OR "Case control"[tw] OR cohort*[tw] OR "Cohort analy*"[tw] OR "Follow up stud*"[tw] OR "observational stud*"[tw] OR Longitudinal[tw] OR Retrospective*[tw] OR prospective*[tw] OR consecutive*[tw] OR Cross sectional[tw] OR "Cross-sectional studies"[mesh] OR "historically controlled study"[mesh] OR "interrupted time series analysis"[mesh]) OR** ("life cycle assess*"[tw] OR "life cycle assessment"[tw] OR "life cycle inventory"[tw] OR "LCA"[tw] OR "LCAs"[tw] OR "life cycle inventory"[tw] OR "life cycle inventories"[tw])**)**

**Embase (OVID)**

(("reusable scissor".mp OR "reusable scissors".mp OR "reusable scope".mp OR "reusable scopes".mp OR "reusable specula".mp OR "reusable speculum".mp OR "reusable surgical kit".mp OR "reusable surgical kits".mp OR "reusable ureteroscope".mp OR "reusable ureteroscopes".mp OR "recyclable flexible optical scope".mp OR "recyclable flexible optical scopes".mp OR "recyclable scissor".mp OR "recyclable scissors".mp OR "recyclable scope".mp OR "recyclable scopes".mp OR "recyclable specula".mp OR "recyclable speculum".mp OR "recyclable surgical kit".mp OR "recyclable surgical kits".mp OR "recyclable ureteroscope".mp OR "recyclable ureteroscopes".mp OR ((exp "Recycling"/ OR "reusables".mp OR "reusable".mp OR "reusabl*".mp OR "reuse".mp OR "re usables".mp OR "re usable".mp OR "re usabl*".mp OR "re use".mp OR "recyclable".mp OR "recyclables".mp OR "recycl*".mp OR "re cyclable".mp OR "re cycl*".mp OR "reprocess".mp OR "reprocessing".mp OR "reprocess*".mp) AND ("flexible optical scope".mp OR "flexible optical scopes".mp OR exp "Scissors"/ OR "scissor".mp OR "scissors".mp OR exp "Endoscope"/ OR "scopes".mp OR "Endoscopes".mp OR "Endoscope".mp OR "ophthalmic speculum"/ OR "nose speculum"/ OR "vaginal speculum"/ OR "rectal speculum"/ OR "gynecological and obstetric surgical equipment"/ OR "specula".mp OR "speculum".mp OR "Surgical Scissor".mp OR "Surgical Scissors".mp OR exp "Stapler"/ OR "Surgical Staplers".mp OR "Surgical Stapler".mp OR "Staplers".mp OR "Stapler".mp OR "Staples".mp OR "Staple".mp OR "Stapling".mp OR exp "Surgical Drill"/ OR "Surgical Drill".mp OR "Surgical Drills".mp OR "Surgical Drilling".mp OR exp "Drill"/ OR "Drill".mp OR "Drills".mp OR "Drilling".mp OR "surgical kit".mp OR "surgical kits".mp OR "surgery kit".mp OR "surgery kits".mp OR exp "Suture Anchor"/ OR "suture anchor".mp OR "suture anchors".mp OR "Bone Anchor".mp OR "Bone Anchors".mp OR exp "Ureteroscope"/ OR "ureteroscope".mp OR "ureteroscopes".mp OR "kocher".mp OR "pincer".mp OR "pincers".mp OR "tweezer".mp OR "tweezers".mp OR exp "Scalpel"/ OR "scalpel".mp OR "scalpels".mp OR exp "needle holder"/ OR "needle holder".mp OR "needle holders".mp OR exp "vessel sealing system "/ OR "ligasure".mp OR "ligasur*".mp OR "harmonic".mp OR "harmonic*".mp OR exp "Angioscope"/ OR "Angioscope".mp OR "Angioscopes".mp OR exp "Arthroscope"/ OR "Arthroscope".mp OR "Arthroscopes".mp OR exp "Bronchoscope"/ OR "Bronchoscope".mp OR "Bronchoscopes".mp OR exp "Colonoscope"/ OR "Colonoscope".mp OR "Colonoscopes".mp OR exp "Colposcope"/ OR "Colposcope".mp OR "Colposcopes".mp OR exp "Culdoscope"/ OR "Culdoscope".mp OR "Culdoscopes".mp OR exp "Cystoscope"/ OR "Cystoscope".mp OR "Cystoscopes".mp OR exp "Duodenoscope"/ OR "Duodenoscope".mp OR "Duodenoscopes".mp OR exp "Esophagoscope"/ OR "Esophagoscope".mp OR "Esophagoscopes".mp OR exp "Fetoscope"/ OR "Fetoscope".mp OR "Fetoscopes".mp OR exp "Gastroscope"/ OR "Gastroscope".mp OR "Gastroscopes".mp OR exp "Hysteroscope"/ OR "Hysteroscope".mp OR "Hysteroscopes".mp OR exp "Laparoscope"/ OR "Laparoscope".mp OR "Laparoscopes".mp OR exp "Laryngoscope"/ OR "Laryngoscope".mp OR "Laryngoscopes".mp OR exp "Mediastinoscope"/ OR "Mediastinoscope".mp OR "Mediastinoscopes".mp OR exp "Neuroendoscope"/ OR "Neuroendoscope".mp OR "Neuroendoscopes".mp OR exp "Proctoscope"/ OR "Proctoscope".mp OR "Proctoscopes".mp OR exp "Sigmoidoscope"/ OR "Sigmoidoscope".mp OR "Sigmoidoscopes".mp OR exp "Thoracoscope"/ OR "Thoracoscope".mp OR "Thoracoscopes".mp OR ((exp "Knee Meniscus"/ OR exp "Knee Meniscus Rupture"/ OR "menisc*".mp) AND (exp "Suturing Method"/ OR exp "Suture"/ OR "sutur*".mp))))) AND ("disposable scissor".mp OR "disposable scissors".mp OR "disposable scope".mp OR "disposable scopes".mp OR "disposable specula".mp OR "disposable speculum".mp OR "single use scissor".mp OR "single use scissors".mp OR "single use scope".mp OR "single use scopes".mp OR ((exp "Disposable Equipment"/ OR "disposable".mp OR "disposables".mp OR "dispos*".mp OR "single use".mp OR "single use*".mp OR "singleuse".mp OR "singleuse*".mp OR "single usage".mp OR "single usage*".mp) AND (exp "Scissors"/ OR "scissor".mp OR "scissors".mp OR exp "Endoscope"/ OR "scopes".mp OR "Endoscopes".mp OR "Endoscope".mp OR "Surgical Scissor".mp OR "Surgical Scissors".mp OR exp "Stapler"/ OR "Surgical Staplers".mp OR "Surgical Stapler".mp OR "Surgery Staplers".mp OR "Surgery Stapler".mp OR "Staplers".mp OR "Stapler".mp OR "Stapling".mp OR exp "Surgical Drill"/ OR exp "Drill"/ OR "Surgical Drill".mp OR "Surgical Drills".mp OR "Surgical Drilling".mp OR "Drill".mp OR "Drills".mp OR "Drilling".mp OR "kocher".mp OR "pincer".mp OR "pincers".mp OR "tweezer".mp OR "tweezers".mp OR exp "Scalpel"/ OR "scalpel".mp OR "scalpels".mp OR exp"Needle Holder"/ OR "needle holder".mp OR "needle holders".mp OR "ligasure".mp OR "ligasur*".mp OR "harmonic".mp OR "harmonic*".mp OR exp "Angioscope"/ OR "Angioscope".mp OR "Angioscopes".mp OR exp "Arthroscope"/ OR "Arthroscope".mp OR "Arthroscopes".mp OR exp "Bronchoscope"/ OR "Bronchoscope".mp OR "Bronchoscopes".mp OR exp "Colonoscope"/ OR "Colonoscope".mp OR "Colonoscopes".mp OR exp "Colposcope"/ OR "Colposcope".mp OR "Colposcopes".mp OR exp "Culdoscope"/ OR "Culdoscope".mp OR "Culdoscopes".mp OR exp "Cystoscope"/ OR "Cystoscope".mp OR "Cystoscopes".mp OR exp "Duodenoscope"/ OR "Duodenoscope".mp OR "Duodenoscopes".mp OR exp "Esophagoscope"/ OR "Esophagoscope".mp OR "Esophagoscopes".mp OR exp "Fetoscope"/ OR "Fetoscope".mp OR "Fetoscopes".mp OR exp "Gastroscope"/ OR "Gastroscope".mp OR "Gastroscopes".mp OR exp "Hysteroscope"/ OR "Hysteroscope".mp OR "Hysteroscopes".mp OR exp "Laparoscope"/ OR "Laparoscope".mp OR "Laparoscopes".mp OR exp "Laryngoscope"/ OR "Laryngoscope".mp OR "Laryngoscopes".mp OR exp "Mediastinoscope"/ OR "Mediastinoscope".mp OR "Mediastinoscopes".mp OR exp "Neuroendoscope"/ OR "Neuroendoscope".mp OR "Neuroendoscopes".mp OR exp "Proctoscope"/ OR "Proctoscope".mp OR "Proctoscopes".mp OR exp "Sigmoidoscope"/ OR "Sigmoidoscope".mp OR "Sigmoidoscopes".mp OR exp "Thoracoscope"/ OR "Thoracoscope".mp OR "Thoracoscopes".mp))) AND ("Carbon Footprint"/ OR "carbon footprint".mp OR "carbon footprint*".mp OR exp "Climate Change"/ OR "climate change".mp OR "CO2 emission".mp OR "CO2 emissions".mp OR "CO2 footprint".mp OR "CO2 footprint*".mp OR exp "environmental protection"/ OR "conservation of natural resources".mp OR "environmental protection".mp OR "ecological footprint".mp OR "ecological footprint*".mp OR "ecological sustainability".mp OR exp "environmental impact"/ OR "environmental impact".mp OR "environmental impact*".mp OR "environmental impacts".mp OR "environmental pollut*".mp OR exp "pollution"/ OR "environmental pollution".mp OR "environmental sustainab*".mp OR "environmental sustainability"/ OR "environmental sustainability".mp OR "Global Warming"/ OR "Global Warming".mp OR "Greenhouse Effect"/ OR "greenhouse effect*".mp OR "greenhouse effects".mp OR "greenhouse gas emission".mp OR "greenhouse gas emissions".mp OR "Greenhouse Gas"/ OR "greening".mp OR "hospital waste".mp OR "life cycle assessment"/ OR "environmental impact assessment"/ OR "life cycle assess*".mp OR "life cycle assessment".mp OR "LCA".mp OR "LCAs".mp OR "life cycle inventory".mp OR "life cycle inventories".mp OR exp "Waste Disposal"/ OR exp "Hospital Waste"/ OR "medical waste".mp OR "Rising Sea Level".mp OR "Rising Sea Levels".mp OR "Sea Level Rise"/ OR "Sea Level Rise".mp OR "sustainability"**.mp** OR "Waste Disposal".mp OR "waste water recycling"/ OR exp "Waste Disposal"/ OR exp "Waste Management"/ OR "Plastic overuse".mp OR "Green surgery".mp OR "Emission reduction".mp OR "Emission reduction strategy".mp OR "air pollution control"/ OR "air pollution control".mp **OR "Environment"/ OR "Environmental*".mp** OR "acidification"/ OR "soil acidification"/ OR "ocean acidification"/ OR "acidification".mp OR "Acidification potential".mp OR "AP in kg SO2 equivalents".mp OR "eco-efficiency".mp OR "ecoefficiency".mp OR "eco-efficien*".mp OR "ecoefficien*".mp OR "ecotoxicity"/ OR "ecotoxicity".mp OR "ecotoxic*".mp OR "eco toxicity".mp OR "eco toxic*".mp OR "EP in kg PO4 equivalent".mp OR exp "Eutrophication"/ OR "eutrophication".mp OR "Eutrophication potential".mp OR "FAETP in kg DCB equivalent".mp OR "Freshwater Aquatic Ecotoxicity Potential".mp OR "GWP in kg CO2 equivalents".mp OR "H+ moles equivalents".mp OR "HTTP in kg Dichlorobenzene equivalent".mp OR "Human Toxicity Potential".mp OR "kg 2.4-D equivalents".mp OR "kg CFC-11 equivalent".mp OR "kg N equivalents".mp OR "kg NOx equivalents".mp OR "life cycle analysis".mp OR "ozone depletion".mp OR "Photochemical Ozone Depletion Potential".mp OR "POCP in kg ethane equivalent".mp OR "smog".mp OR exp "Waste"/ OR "waste".mp OR "wastes".mp OR "Ozone Depletion"/ OR "Smog"/ OR "Greenhouse Gases".mp OR "Greenhouse Gas".mp OR "SO2 equiv*".mp OR "CO2 equiva*".mp OR "CFC-11 equiv*".mp OR "N equiv*".mp OR exp "Biodiversity"/ OR "Biodiversity".mp OR "Climatic change".mp OR "Green deal".mp OR "preservation of natural resources".mp OR "Refuse Disposal".mp OR exp "Wastewater"/ OR "Waste Water".mp OR "Wastewater".mp OR exp "Water Management"/ OR "Water Purification".mp OR (("plastic*".mp OR "microplastic*") AND ("soop".mp OR "soup".mp OR "pollution".mp OR "overuse".mp OR "contamination".mp)) OR "Sustainable Development"/ OR "Sustainable Development".mp OR (("Plastic".mp OR "plastics".mp) AND "overuse".mp) OR ("hydrogen*".mp AND "moles".mp AND "equiv*".mp) OR ("Dichlorobenzen*".mp AND "equiv*".mp) OR ("2,4-D".mp AND "equiv*".mp) OR ("NOx".mp AND "equiv*".mp) OR ("ethane".mp AND "equiv*".mp) OR ("PO4".mp AND "equiv*".mp) OR ("DCB".mp AND "equiv*".mp) OR ("sustainability".mp AND ("environment*".mp OR "carbon".mp)) OR (("Carbon Dioxide"/ OR "Carbon Dioxide".mp OR "CO2".mp) AND ("pollution".mp OR "emission".mp OR "emissions".mp OR "waste".mp OR "environment".mp OR "environmental*".mp OR "footprint".mp OR "footprint*".mp OR "sustainable".mp OR "hazard".mp OR "hazard*".mp))) NOT (conference review or conference abstract).pt AND (2000 OR 2001 OR 2002 OR 2003 OR 2004 OR 2005 OR 2006 OR 2007 OR 2008 OR 2009 OR 2010 OR 2011 OR 2012 OR 2013 OR 2014 OR 2015 OR 2016 OR 2017 OR 2018 OR 2019 OR 2020 OR 2021 OR 2022).yr) AND **(***(exp "meta analysis"/ OR exp "meta analysis (topic)"/ OR metaanaly*.ti,ab OR "meta analy*".ti,ab OR metanaly*.ti,ab OR "systematic review"/ OR "cochrane database of systematic reviews".jn OR prisma.ti,ab OR prospero.ti,ab OR (((systemati* OR scoping OR umbrella OR "structured literature") ADJ3 (review* OR overview*)).ti,ab) OR ((systemic* ADJ1 review*).ti,ab) OR (((systemati* OR literature OR database* OR "data base*") ADJ10 search*).ti,ab) OR (((structured OR comprehensive* OR systemic*) ADJ3 search*).ti,ab) OR (((literature ADJ3 review*).ti,ab) AND (search*.ti,ab OR database*.ti,ab OR "data base*".ti,ab)) OR (("data extraction".ti,ab OR "data source*".ti,ab) AND "study selection".ti,ab) OR ("search strategy".ti,ab AND "selection criteria".ti,ab) OR ("data source*".ti,ab AND "data synthesis".ti,ab) OR medline.ab OR pubmed.ab OR embase.ab OR cochrane.ab OR (((critical OR rapid) ADJ2 (review* OR overview* OR synthes*)).ti) OR ((((critical* OR rapid*) ADJ3 (review* OR overview* OR synthes*)).ab) AND (search*.ab OR database*.ab OR "data base*".ab)) OR metasynthes*.ti,ab OR "meta synthes*".ti,ab)* OR (exp "clinical trial"/ OR exp "randomization"/ OR exp "single blind procedure"/ OR exp "double blind procedure"/ OR exp "crossover procedure"/ OR exp "placebo"/ OR exp "prospective study"/ OR rct.ti,ab OR random*.ti,ab OR "single blind".ti,ab OR "randomised controlled trial".ti,ab OR exp "randomized controlled trial"/ OR placebo*.ti,ab) **OR (exp "Comparative Study"/ OR "comparison".ti,ab OR "comparative".ti,ab OR "compar*".ti,ab OR "major clinical study"/ OR "clinical study"/ OR "case control study"/ OR "family study"/ OR "longitudinal study"/ OR "retrospective study"/ OR "prospective study"/ OR "cohort analysis"/ OR cohort*.ti,ab OR (("case control" ADJ1 (study OR studies)).ti,ab) OR (("follow up" ADJ1 (study OR studies)).ti,ab) OR (observational ADJ1 (study OR studies)) OR ((epidemiologic ADJ1 (study OR studies)).ti,ab) OR (("cross sectional" ADJ1 (study OR studies)).ti,ab)) OR** ("life cycle assessment"/ OR "environmental impact assessment"/ OR "life cycle assess*".mp OR "life cycle assessment".mp OR "life cycle inventory".mp OR "LCA".mp OR "LCAs".mp OR "life cycle inventory".mp OR "life cycle inventories".mp)**)**

**Web of Science**

(TS=("reusable scissor" OR "reusable scissors" OR "reusable scope" OR "reusable scopes" OR "reusable specula" OR "reusable speculum" OR "reusable surgical kit" OR "reusable surgical kits" OR "reusable ureteroscope" OR "reusable ureteroscopes" OR "recyclable flexible optical scope" OR "recyclable flexible optical scopes" OR "recyclable scissor" OR "recyclable scissors" OR "recyclable scope" OR "recyclable scopes" OR "recyclable specula" OR "recyclable speculum" OR "recyclable surgical kit" OR "recyclable surgical kits" OR "recyclable ureteroscope" OR "recyclable ureteroscopes" OR (("Equipment Reuse" OR "reusables" OR "reusable" OR "reusabl*" OR "reuse" OR "re usables" OR "re usable" OR "re usabl*" OR "re use" OR "recyclable" OR "recyclables" OR "recycl*" OR "re cyclable" OR "re cycl*" OR "Recycling" OR "reprocess" OR "reprocessing" OR "reprocess*") AND ("flexible optical scope" OR "flexible optical scopes" OR "scissor" OR "scissors" OR "Endoscopes" OR "Endoscopes" OR "Endoscope" OR "specula" OR "speculum" OR "scopes" OR "Surgical Scissor" OR "Surgical Scissors" OR "Surgical Staplers" OR "Surgical Stapler" OR "Staplers" OR "Stapler" OR "Staples" OR "Staple" OR "Stapling" OR "Surgical Drill" OR "Surgical Drills" OR "Surgical Drilling" OR "surgical kit" OR "surgical kits" OR "surgery kit" OR "surgery kits" OR "Suture Anchors" OR "suture anchor" OR "suture anchors" OR "Bone Anchor" OR "Bone Anchors" OR "Ureteroscopes" OR "ureteroscope" OR "ureteroscopes" OR "kocher" OR "pincer" OR "pincers" OR "tweezer" OR "tweezers" OR "scalpel" OR "scalpels" OR "needle holder" OR "needle holders" OR "ligasure" OR "ligasur*" OR "harmonic" OR "harmonic*" OR "Angioscope" OR "Angioscopes" OR "Arthroscope" OR "Arthroscopes" OR "Bronchoscope" OR "Bronchoscopes" OR "Colonoscope" OR "Colonoscopes" OR "Colposcope" OR "Colposcopes" OR "Culdoscope" OR "Culdoscopes" OR "Cystoscope" OR "Cystoscopes" OR "Duodenoscope" OR "Duodenoscopes" OR "Esophagoscope" OR "Esophagoscopes" OR "Fetoscope" OR "Fetoscopes" OR "Gastroscope" OR "Gastroscopes" OR "Hysteroscope" OR "Hysteroscopes" OR "Laparoscope" OR "Laparoscopes" OR "Laryngoscope" OR "Laryngoscopes" OR "Mediastinoscope" OR "Mediastinoscopes" OR "Neuroendoscope" OR "Neuroendoscopes" OR "Proctoscope" OR "Proctoscopes" OR "Sigmoidoscope" OR "Sigmoidoscopes" OR "Thoracoscope" OR "Thoracoscopes" OR (("Menisci, Tibial" OR "menisc*") AND ("Suture Techniques" OR "sutur*"))))) AND TS=("disposable scissor" OR "disposable scissors" OR "disposable scope" OR "disposable scopes" OR "disposable specula" OR "disposable speculum" OR "single use scissor" OR "single use scissors" OR "single use scope" OR "single use scopes" OR (("Disposable Equipment" OR "disposable" OR "disposables" OR "dispos*" OR "single use" OR "single use*" OR "singleuse" OR "singleuse*" OR "single usage" OR "single usage*") AND ("scissor" OR "scissors" OR "Endoscopes" OR "Endoscopes" OR "Endoscope" OR "Surgical Scissor" OR "Surgical Scissors" OR "scopes" OR "Surgical Staplers" OR "Surgical Stapler" OR "Surgery Staplers" OR "Surgery Stapler" OR "Staplers" OR "Stapler" OR "Stapling" OR "Surgical Drill" OR "Surgical Drills" OR "Surgical Drilling" OR "kocher" OR "pincer" OR "pincers" OR "tweezer" OR "tweezers" OR "scalpel" OR "scalpels" OR "needle holder" OR "needle holders" OR "ligasure" OR "ligasur*" OR "harmonic" OR "harmonic*" OR "Angioscope" OR "Angioscopes" OR "Arthroscope" OR "Arthroscopes" OR "Bronchoscope" OR "Bronchoscopes" OR "Colonoscope" OR "Colonoscopes" OR "Colposcope" OR "Colposcopes" OR "Culdoscope" OR "Culdoscopes" OR "Cystoscope" OR "Cystoscopes" OR "Duodenoscope" OR "Duodenoscopes" OR "Esophagoscope" OR "Esophagoscopes" OR "Fetoscope" OR "Fetoscopes" OR "Gastroscope" OR "Gastroscopes" OR "Hysteroscope" OR "Hysteroscopes" OR "Laparoscope" OR "Laparoscopes" OR "Laryngoscope" OR "Laryngoscopes" OR "Mediastinoscope" OR "Mediastinoscopes" OR "Neuroendoscope" OR "Neuroendoscopes" OR "Proctoscope" OR "Proctoscopes" OR "Sigmoidoscope" OR "Sigmoidoscopes" OR "Thoracoscope" OR "Thoracoscopes"))) AND (TS=("Carbon Footprint" OR "carbon footprint" OR "carbon footprint*" OR "Climate Change" OR "climate change" OR "CO2 emission" OR "CO2 emissions" OR "CO2 footprint" OR "CO2 footprint*" OR "environmental protection" OR "conservation of natural resources" OR "environmental protection" OR "ecological footprint" OR "ecological footprint*" OR "ecological sustainability" OR "environmental impact" OR "environmental impact" OR "environmental impact*" OR "environmental impacts" OR "environmental pollut*" OR "pollution" OR "environmental pollution" OR "environmental sustainab*" OR "environmental sustainability" OR "environmental sustainability" OR "Global Warming" OR "Global Warming" OR "Greenhouse Effect" OR "greenhouse effect*" OR "greenhouse effects" OR "greenhouse gas emission" OR "greenhouse gas emissions" OR "Greenhouse Gas" OR "greening" OR "hospital waste" OR "life cycle assessment" OR "environmental impact assessment" OR "life cycle assess*" OR "life cycle assessment" OR "LCA" OR "LCAs" OR "life cycle inventory" OR "life cycle inventories" OR "Waste Disposal" OR "Hospital Waste" OR "medical waste" OR "Rising Sea Level" OR "Rising Sea Levels" OR "Sea Level Rise" OR "Sea Level Rise" OR "Waste Disposal" OR "waste water recycling" OR "Waste Disposal" OR "Waste Management" OR "Plastic overuse" OR "Green surgery" OR "Emission reduction" OR "Emission reduction strategy" OR "air pollution control" OR "air pollution control" OR "acidification" OR "soil acidification" OR "ocean acidification" OR "acidification" OR "Acidification potential" OR "AP in kg SO2 equivalents" OR "eco-efficiency" OR "ecoefficiency" OR "eco-efficien*" OR "ecoefficien*" OR "ecotoxicity" OR "ecotoxicity" OR "ecotoxic*" OR "eco toxicity" OR "eco toxic*" OR "EP in kg PO4 equivalent" OR "Eutrophication" OR "eutrophication" OR "Eutrophication potential" OR "FAETP in kg DCB equivalent" OR "Freshwater Aquatic Ecotoxicity Potential" OR "GWP in kg CO2 equivalents" OR "H+ moles equivalents" OR "HTTP in kg Dichlorobenzene equivalent" OR "Human Toxicity Potential" OR "kg 2.4-D equivalents" OR "kg CFC-11 equivalent" OR "kg N equivalents" OR "kg NOx equivalents" OR "life cycle analysis" OR "ozone depletion" OR "Photochemical Ozone Depletion Potential" OR "POCP in kg ethane equivalent" OR "smog" OR "Waste" OR "waste" OR "wastes" OR "Ozone Depletion" OR "Smog" OR "Greenhouse Gases" OR "Greenhouse Gas" OR "SO2 equiv*" OR "CO2 equiva*" OR "CFC-11 equiv*" OR "N equiv*" OR "Sustainable Development" OR "Sustainable Development" OR "Biodiversity" OR "Climatic change" OR "Green deal" OR "preservation of natural resources" OR "Refuse Disposal" OR "Waste Water" OR "Wastewater" OR "Water Purification" OR (("plastic*" OR "microplastic*") AND ("soop" OR "soup" OR "pollution" OR "overuse" OR "contamination")) OR (("Plastic" OR "plastics") AND "overuse") OR ("hydrogen*" AND "moles" AND "equiv*") OR ("Dichlorobenzen*" AND "equiv*") OR ("2,4-D" AND "equiv*") OR ("NOx" AND "equiv*") OR ("ethane" AND "equiv*") OR ("PO4" AND "equiv*") OR ("DCB" AND "equiv*") OR ("sustainability" AND ("environment*" OR "carbon")) OR (("Carbon Dioxide" OR "Carbon Dioxide" OR "CO2") AND ("pollution" OR "emission" OR "emissions" OR "waste" OR "environment" OR "environmental*" OR "footprint" OR "footprint*" OR "sustainable" OR "hazard" OR "hazard*"))) OR **TI=("environmental*" OR "sustainab*")** OR **AB=("environmental*" OR "sustainab*")** OR **AK=("environmental*" OR "sustainab*")**) NOT DT=(meeting abstract) AND PY=(2000 OR 2001 OR 2002 OR 2003 OR 2004 OR 2005 OR 2006 OR 2007 OR 2008 OR 2009 OR 2010 OR 2011 OR 2012 OR 2013 OR 2014 OR 2015 OR 2016 OR 2017 OR 2018 OR 2019 OR 2020 OR 2021 OR 2022)) AND **(**TI=**(***("Meta-Analysis" OR metaanaly* OR "meta-analy*" or metanaly* OR "Systematic Review" OR "Cochrane Database Syst Rev" OR "prisma" OR "preferred reporting items" OR "prospero" OR ((systemati* OR scoping OR umbrella OR "structured literature") NEAR/4 (review* OR overview*)) OR "systematic review*" OR "scoping review*" OR "umbrella review*" OR "structured literature review*" OR "systematic qualitative review*" OR "systematic quantitative review*" OR "systematic search and review" OR "systematized review" OR "systematised review" OR "systemic review" OR "systematic literature review*" OR "systematic integrative literature review*" OR "systematically review*" OR "scoping literature review*" OR "systematic critical review" OR "systematic integrative review*" OR "systematic evidence review" OR "Systematic integrative literature review*" OR "Systematic mixed studies review*" OR "Systematized literature review*" OR "Systematic overview*" OR "Systematic narrative review*" OR (("systemati*" OR "literature" OR "database*" OR "data-base*" OR "structured" OR "comprehensive*" OR "systemic*") NEAR/4 "search*") OR ("Literature" AND "review" AND ("database*" OR "data-base*" OR "search*")) OR (("data extraction" OR "data source*") AND "study selection") OR ("search strategy" AND "selection criteria") OR ("data source*" AND "data synthesis") OR "medline" OR "pubmed" OR "embase" OR "Cochrane" OR (("critical" OR "rapid") NEAR/4 ("review*" OR "overview*" OR "synthes*")) OR ((("critical*" OR "rapid*") NEAR/4 ("review*" OR "overview*" OR "synthes*") NEAR/4 ("search*" OR "database*" OR "data-base*"))) OR metasynthes* OR "meta-synthes*")* **OR** ("Randomized Controlled Trial" OR random* OR "RCT"OR "RCTs" OR "pragmatic clinical trial*" OR "practical clinical trial*" OR "non-inferiority trial*" OR "noninferiority trial*" OR "superiority trial*" OR "equivalence clinical trial*") **OR ("Comparative Study" OR "comparison" OR "comparative" OR "compar*" OR "major clinical study" OR "clinical study" OR "case control study" OR "family study" OR "longitudinal study" OR "retrospective study" OR "prospective study" OR "cohort analysis" OR cohort* OR (("case control" NEAR/1 (study OR studies))) OR (("follow up" NEAR/1 (study OR studies))) OR (observational NEAR/1 (study OR studies)) OR ((epidemiologic NEAR/1 (study OR studies))) OR (("cross sectional" NEAR/1 (study OR studies)))) OR** ("life cycle assess*" OR "life cycle assessment" OR "life cycle inventory" OR "LCA" OR "LCAs" OR "life cycle inventory" OR "life cycle inventories")**)** OR AB=**(***("Meta-Analysis" OR metaanaly* OR "meta-analy*" or metanaly* OR "Systematic Review" OR "Cochrane Database Syst Rev" OR "prisma" OR "preferred reporting items" OR "prospero" OR ((systemati* OR scoping OR umbrella OR "structured literature") NEAR/4 (review* OR overview*)) OR "systematic review*" OR "scoping review*" OR "umbrella review*" OR "structured literature review*" OR "systematic qualitative review*" OR "systematic quantitative review*" OR "systematic search and review" OR "systematized review" OR "systematised review" OR "systemic review" OR "systematic literature review*" OR "systematic integrative literature review*" OR "systematically review*" OR "scoping literature review*" OR "systematic critical review" OR "systematic integrative review*" OR "systematic evidence review" OR "Systematic integrative literature review*" OR "Systematic mixed studies review*" OR "Systematized literature review*" OR "Systematic overview*" OR "Systematic narrative review*" OR (("systemati*" OR "literature" OR "database*" OR "data-base*" OR "structured" OR "comprehensive*" OR "systemic*") NEAR/4 "search*") OR ("Literature" AND "review" AND ("database*" OR "data-base*" OR "search*")) OR (("data extraction" OR "data source*") AND "study selection") OR ("search strategy" AND "selection criteria") OR ("data source*" AND "data synthesis") OR "medline" OR "pubmed" OR "embase" OR "Cochrane" OR (("critical" OR "rapid") NEAR/4 ("review*" OR "overview*" OR "synthes*")) OR ((("critical*" OR "rapid*") NEAR/4 ("review*" OR "overview*" OR "synthes*") NEAR/4 ("search*" OR "database*" OR "data-base*"))) OR metasynthes* OR "meta-synthes*")* **OR** ("Randomized Controlled Trial" OR random* OR "RCT"OR "RCTs" OR "pragmatic clinical trial*" OR "practical clinical trial*" OR "non-inferiority trial*" OR "noninferiority trial*" OR "superiority trial*" OR "equivalence clinical trial*") **OR ("Comparative Study" OR "comparison" OR "comparative" OR "compar*" OR "major clinical study" OR "clinical study" OR "case control study" OR "family study" OR "longitudinal study" OR "retrospective study" OR "prospective study" OR "cohort analysis" OR cohort* OR (("case control" NEAR/1 (study OR studies))) OR (("follow up" NEAR/1 (study OR studies))) OR (observational NEAR/1 (study OR studies)) OR ((epidemiologic NEAR/1 (study OR studies))) OR (("cross sectional" NEAR/1 (study OR studies)))) OR** ("life cycle assess*" OR "life cycle assessment" OR "life cycle inventory" OR "LCA" OR "LCAs" OR "life cycle inventory" OR "life cycle inventories")**))**

**Cochrane Library**

("reusable scissor" OR "reusable scissors" OR "reusable scope" OR "reusable scopes" OR "reusable specula" OR "reusable speculum" OR "reusable surgical kit" OR "reusable surgical kits" OR "reusable ureteroscope" OR "reusable ureteroscopes" OR "recyclable flexible optical scope" OR "recyclable flexible optical scopes" OR "recyclable scissor" OR "recyclable scissors" OR "recyclable scope" OR "recyclable scopes" OR "recyclable specula" OR "recyclable speculum" OR "recyclable surgical kit" OR "recyclable surgical kits" OR "recyclable ureteroscope" OR "recyclable ureteroscopes" OR (("Equipment Reuse" OR "reusables" OR "reusable" OR "reusabl*" OR "reuse" OR "re usables" OR "re usable" OR "re usabl*" OR "re use" OR "recyclable" OR "recyclables" OR "recycl*" OR "re cyclable" OR "re cycl*" OR "Recycling" OR "reprocess" OR "reprocessing" OR "reprocess*") AND ("flexible optical scope" OR "flexible optical scopes" OR "scissor" OR "scissors" OR "Endoscopes" OR "scopes" OR "Endoscopes" OR "Endoscope" OR "specula" OR "speculum" OR "Surgical Scissor" OR "Surgical Scissors" OR "Surgical Staplers" OR "Surgical Stapler" OR "Staplers" OR "Stapler" OR "Staples" OR "Staple" OR "Stapling" OR "Surgical Drill" OR "Surgical Drills" OR "Surgical Drilling" OR "Drill" OR "Drills" OR "Drilling" OR "surgical kit" OR "surgical kits" OR "surgery kit" OR "surgery kits" OR "Suture Anchors" OR "suture anchor" OR "suture anchors" OR "Bone Anchor" OR "Bone Anchors" OR "Ureteroscopes" OR "ureteroscope" OR "ureteroscopes" OR "kocher" OR "pincer" OR "pincers" OR "tweezer" OR "tweezers" OR "scalpel" OR "scalpels" OR "needle holder" OR "needle holders" OR "ligasure" OR "ligasur*" OR "harmonic" OR "harmonic*" OR "Angioscope" OR "Angioscopes" OR "Arthroscope" OR "Arthroscopes" OR "Bronchoscope" OR "Bronchoscopes" OR "Colonoscope" OR "Colonoscopes" OR "Colposcope" OR "Colposcopes" OR "Culdoscope" OR "Culdoscopes" OR "Cystoscope" OR "Cystoscopes" OR "Duodenoscope" OR "Duodenoscopes" OR "Esophagoscope" OR "Esophagoscopes" OR "Fetoscope" OR "Fetoscopes" OR "Gastroscope" OR "Gastroscopes" OR "Hysteroscope" OR "Hysteroscopes" OR "Laparoscope" OR "Laparoscopes" OR "Laryngoscope" OR "Laryngoscopes" OR "Mediastinoscope" OR "Mediastinoscopes" OR "Neuroendoscope" OR "Neuroendoscopes" OR "Proctoscope" OR "Proctoscopes" OR "Sigmoidoscope" OR "Sigmoidoscopes" OR "Thoracoscope" OR "Thoracoscopes" OR (("Menisci, Tibial" OR "menisc*") AND ("Suture Techniques" OR "sutur*"))))):ti,ab,kw AND

("disposable scissor" OR "disposable scissors" OR "disposable scope" OR "disposable scopes" OR "disposable specula" OR "disposable speculum" OR "single use scissor" OR "single use scissors" OR "single use scope" OR "single use scopes" OR (("Disposable Equipment" OR "disposable" OR "disposables" OR "dispos*" OR "single use" OR "single use*" OR "singleuse" OR "singleuse*" OR "single usage" OR "single usage*") AND ("scissor" OR "scissors" OR "Endoscopes" OR "scopes" OR "Endoscopes" OR "Endoscope" OR "Surgical Scissor" OR "Surgical Scissors" OR "Surgical Staplers" OR "Surgical Stapler" OR "Surgery Staplers" OR "Surgery Stapler" OR "Staplers" OR "Stapler" OR "Stapling" OR "Surgical Drill" OR "Surgical Drills" OR "Surgical Drilling" OR "Drill" OR "Drills" OR "Drilling" OR "kocher" OR "pincer" OR "pincers" OR "tweezer" OR "tweezers" OR "scalpel" OR "scalpels" OR "needle holder" OR "needle holders" OR "ligasure" OR "ligasur*" OR "harmonic" OR "harmonic*" OR "Angioscope" OR "Angioscopes" OR "Arthroscope" OR "Arthroscopes" OR "Bronchoscope" OR "Bronchoscopes" OR "Colonoscope" OR "Colonoscopes" OR "Colposcope" OR "Colposcopes" OR "Culdoscope" OR "Culdoscopes" OR "Cystoscope" OR "Cystoscopes" OR "Duodenoscope" OR "Duodenoscopes" OR "Esophagoscope" OR "Esophagoscopes" OR "Fetoscope" OR "Fetoscopes" OR "Gastroscope" OR "Gastroscopes" OR "Hysteroscope" OR "Hysteroscopes" OR "Laparoscope" OR "Laparoscopes" OR "Laryngoscope" OR "Laryngoscopes" OR "Mediastinoscope" OR "Mediastinoscopes" OR "Neuroendoscope" OR "Neuroendoscopes" OR "Proctoscope" OR "Proctoscopes" OR "Sigmoidoscope" OR "Sigmoidoscopes" OR "Thoracoscope" OR "Thoracoscopes"))):ti,ab,kw AND

(("Carbon Footprint" OR "carbon footprint" OR "carbon footprint*" OR "Climate Change" OR "climate change" OR "CO2 emission" OR "CO2 emissions" OR "CO2 footprint" OR "CO2 footprint*" OR "environmental protection" OR "conservation of natural resources" OR "environmental protection" OR "ecological footprint" OR "ecological footprint*" OR "ecological sustainability" OR "environmental impact" OR "environmental impact" OR "environmental impact*" OR "environmental impacts" OR "environmental pollut*" OR "pollution" OR "environmental pollution" OR "environmental sustainab*" OR "environmental sustainability" OR "environmental sustainability" OR "Global Warming" OR "Global Warming" OR "Greenhouse Effect" OR "greenhouse effect*" OR "greenhouse effects" OR "greenhouse gas emission" OR "greenhouse gas emissions" OR "Greenhouse Gas" OR "greening" OR "hospital waste" OR "life cycle assessment" OR "environmental impact assessment" OR "life cycle assessment" OR "environmental impact assessment" OR "life cycle assess*" OR "life cycle assessment" OR "LCA" OR "LCAs" OR "life cycle inventory" OR "life cycle inventories" OR "Waste Disposal" OR "Hospital Waste" OR "medical waste" OR "Rising Sea Level" OR "Rising Sea Levels" OR "Sea Level Rise" OR "Sea Level Rise" OR "Waste Disposal" OR "waste water recycling" OR "Equipment reuse" OR "Reusables" OR "reusable" OR "reuse" OR "reused" OR "reusing" OR "Waste Disposal" OR "Waste Management" OR "Plastic overuse" OR "Green surgery" OR "Emission reduction" OR "Emission reduction strategy" OR "air pollution control" OR "air pollution control" OR "acidification" OR "soil acidification" OR "ocean acidification" OR "acidification" OR "Acidification potential" OR "AP in kg SO2 equivalents" OR "eco-efficiency" OR "ecoefficiency" OR "eco-efficien*" OR "ecoefficien*" OR "ecotoxicity" OR "ecotoxicity" OR "ecotoxic*" OR "eco toxicity" OR "eco toxic*" OR "EP in kg PO4 equivalent" OR "Eutrophication" OR "eutrophication" OR "Eutrophication potential" OR "FAETP in kg DCB equivalent" OR "Freshwater Aquatic Ecotoxicity Potential" OR "GWP in kg CO2 equivalents" OR "H+ moles equivalents" OR "HTTP in kg Dichlorobenzene equivalent" OR "Human Toxicity Potential" OR "kg 2.4-D equivalents" OR "kg CFC-11 equivalent" OR "kg N equivalents" OR "kg NOx equivalents" OR "life cycle analysis" OR "ozone depletion" OR "Photochemical Ozone Depletion Potential" OR "POCP in kg ethane equivalent" OR "smog" OR "Waste" OR "waste" OR "wastes" OR "Ozone Depletion" OR "Smog" OR "Equipment reuse" OR "Greenhouse Gases" OR "Greenhouse Gas" OR "SO2 equiv*" OR "CO2 equiva*" OR "CFC-11 equiv*" OR "N equiv*" OR "Biodiversity" OR "Climatic change" OR "Green deal" OR "preservation of natural resources" OR "Refuse Disposal" OR "Waste Water" OR "Wastewater" OR "Water Purification" OR (("plastic*" OR "microplastic*") AND ("soop" OR "soup" OR "pollution" OR "overuse" OR "contamination")) OR "Sustainable Development" OR "Sustainable Development" OR (("Plastic" OR "plastics") AND "overuse") OR ("hydrogen*" AND "moles" AND "equiv*") OR ("Dichlorobenzen*" AND "equiv*") OR ("2,4-D" AND "equiv*") OR ("NOx" AND "equiv*") OR ("ethane" AND "equiv*") OR ("PO4" AND "equiv*") OR ("DCB" AND "equiv*") OR ("sustainability" AND ("environment*" OR "carbon")) OR (("Carbon Dioxide" OR "Carbon Dioxide" OR "CO2") AND ("pollution" OR "emission" OR "emissions" OR "waste" OR "environment" OR "environmental*" OR "footprint" OR "footprint*" OR "sustainable" OR "hazard" OR "hazard*"))):ti,ab,kw OR **("environmental" OR "sustainability")**:ti,ab,kw)

**Emcare (OVID)**

(("reusable scissor".mp OR "reusable scissors".mp OR "reusable scope".mp OR "reusable scopes".mp OR "reusable specula".mp OR "reusable speculum".mp OR "reusable surgical kit".mp OR "reusable surgical kits".mp OR "reusable ureteroscope".mp OR "reusable ureteroscopes".mp OR "recyclable flexible optical scope".mp OR "recyclable flexible optical scopes".mp OR "recyclable scissor".mp OR "recyclable scissors".mp OR "recyclable scope".mp OR "recyclable scopes".mp OR "recyclable specula".mp OR "recyclable speculum".mp OR "recyclable surgical kit".mp OR "recyclable surgical kits".mp OR "recyclable ureteroscope".mp OR "recyclable ureteroscopes".mp OR ((exp "Recycling"/ OR "reusables".mp OR "reusable".mp OR "reusabl*".mp OR "reuse".mp OR "re usables".mp OR "re usable".mp OR "re usabl*".mp OR "re use".mp OR "recyclable".mp OR "recyclables".mp OR "recycl*".mp OR "re cyclable".mp OR "re cycl*".mp OR "reprocess".mp OR "reprocessing".mp OR "reprocess*".mp) AND ("flexible optical scope".mp OR "flexible optical scopes".mp OR exp "Scissors"/ OR "scissor".mp OR "scissors".mp OR exp "Endoscope"/ OR "scopes".mp OR "Endoscopes".mp OR "Endoscope".mp OR "ophthalmic speculum"/ OR "nose speculum"/ OR "vaginal speculum"/ OR "rectal speculum"/ OR "gynecological and obstetric surgical equipment"/ OR "specula".mp OR "speculum".mp OR "Surgical Scissor".mp OR "Surgical Scissors".mp OR exp "Stapler"/ OR "Surgical Staplers".mp OR "Surgical Stapler".mp OR "Staplers".mp OR "Stapler".mp OR "Staples".mp OR "Staple".mp OR "Stapling".mp OR exp "Surgical Drill"/ OR "Surgical Drill".mp OR "Surgical Drills".mp OR "Surgical Drilling".mp OR exp "Drill"/ OR "Drill".mp OR "Drills".mp OR "Drilling".mp OR "surgical kit".mp OR "surgical kits".mp OR "surgery kit".mp OR "surgery kits".mp OR exp "Suture Anchor"/ OR "suture anchor".mp OR "suture anchors".mp OR "Bone Anchor".mp OR "Bone Anchors".mp OR exp "Ureteroscope"/ OR "ureteroscope".mp OR "ureteroscopes".mp OR "kocher".mp OR "pincer".mp OR "pincers".mp OR "tweezer".mp OR "tweezers".mp OR exp "Scalpel"/ OR "scalpel".mp OR "scalpels".mp OR exp "needle holder"/ OR "needle holder".mp OR "needle holders".mp OR exp "vessel sealing system "/ OR "ligasure".mp OR "ligasur*".mp OR "harmonic".mp OR "harmonic*".mp OR exp "Angioscope"/ OR "Angioscope".mp OR "Angioscopes".mp OR exp "Arthroscope"/ OR "Arthroscope".mp OR "Arthroscopes".mp OR exp "Bronchoscope"/ OR "Bronchoscope".mp OR "Bronchoscopes".mp OR exp "Colonoscope"/ OR "Colonoscope".mp OR "Colonoscopes".mp OR exp "Colposcope"/ OR "Colposcope".mp OR "Colposcopes".mp OR exp "Culdoscope"/ OR "Culdoscope".mp OR "Culdoscopes".mp OR exp "Cystoscope"/ OR "Cystoscope".mp OR "Cystoscopes".mp OR exp "Duodenoscope"/ OR "Duodenoscope".mp OR "Duodenoscopes".mp OR exp "Esophagoscope"/ OR "Esophagoscope".mp OR "Esophagoscopes".mp OR exp "Fetoscope"/ OR "Fetoscope".mp OR "Fetoscopes".mp OR exp "Gastroscope"/ OR "Gastroscope".mp OR "Gastroscopes".mp OR exp "Hysteroscope"/ OR "Hysteroscope".mp OR "Hysteroscopes".mp OR exp "Laparoscope"/ OR "Laparoscope".mp OR "Laparoscopes".mp OR exp "Laryngoscope"/ OR "Laryngoscope".mp OR "Laryngoscopes".mp OR exp "Mediastinoscope"/ OR "Mediastinoscope".mp OR "Mediastinoscopes".mp OR exp "Neuroendoscope"/ OR "Neuroendoscope".mp OR "Neuroendoscopes".mp OR exp "Proctoscope"/ OR "Proctoscope".mp OR "Proctoscopes".mp OR exp "Sigmoidoscope"/ OR "Sigmoidoscope".mp OR "Sigmoidoscopes".mp OR exp "Thoracoscope"/ OR "Thoracoscope".mp OR "Thoracoscopes".mp OR ((exp "Knee Meniscus"/ OR exp "Knee Meniscus Rupture"/ OR "menisc*".mp) AND (exp "Suturing Method"/ OR exp "Suture"/ OR "sutur*".mp))))) AND ("disposable scissor".mp OR "disposable scissors".mp OR "disposable scope".mp OR "disposable scopes".mp OR "disposable specula".mp OR "disposable speculum".mp OR "single use scissor".mp OR "single use scissors".mp OR "single use scope".mp OR "single use scopes".mp OR ((exp "Disposable Equipment"/ OR "disposable".mp OR "disposables".mp OR "dispos*".mp OR "single use".mp OR "single use*".mp OR "singleuse".mp OR "singleuse*".mp OR "single usage".mp OR "single usage*".mp) AND (exp "Scissors"/ OR "scissor".mp OR "scissors".mp OR exp "Endoscope"/ OR "scopes".mp OR "Endoscopes".mp OR "Endoscope".mp OR "Surgical Scissor".mp OR "Surgical Scissors".mp OR exp "Stapler"/ OR "Surgical Staplers".mp OR "Surgical Stapler".mp OR "Surgery Staplers".mp OR "Surgery Stapler".mp OR "Staplers".mp OR "Stapler".mp OR "Stapling".mp OR exp "Surgical Drill"/ OR exp "Drill"/ OR "Surgical Drill".mp OR "Surgical Drills".mp OR "Surgical Drilling".mp OR "Drill".mp OR "Drills".mp OR "Drilling".mp OR "kocher".mp OR "pincer".mp OR "pincers".mp OR "tweezer".mp OR "tweezers".mp OR exp "Scalpel"/ OR "scalpel".mp OR "scalpels".mp OR exp"Needle Holder"/ OR "needle holder".mp OR "needle holders".mp OR "ligasure".mp OR "ligasur*".mp OR "harmonic".mp OR "harmonic*".mp OR exp "Angioscope"/ OR "Angioscope".mp OR "Angioscopes".mp OR exp "Arthroscope"/ OR "Arthroscope".mp OR "Arthroscopes".mp OR exp "Bronchoscope"/ OR "Bronchoscope".mp OR "Bronchoscopes".mp OR exp "Colonoscope"/ OR "Colonoscope".mp OR "Colonoscopes".mp OR exp "Colposcope"/ OR "Colposcope".mp OR "Colposcopes".mp OR exp "Culdoscope"/ OR "Culdoscope".mp OR "Culdoscopes".mp OR exp "Cystoscope"/ OR "Cystoscope".mp OR "Cystoscopes".mp OR exp "Duodenoscope"/ OR "Duodenoscope".mp OR "Duodenoscopes".mp OR exp "Esophagoscope"/ OR "Esophagoscope".mp OR "Esophagoscopes".mp OR exp "Fetoscope"/ OR "Fetoscope".mp OR "Fetoscopes".mp OR exp "Gastroscope"/ OR "Gastroscope".mp OR "Gastroscopes".mp OR exp "Hysteroscope"/ OR "Hysteroscope".mp OR "Hysteroscopes".mp OR exp "Laparoscope"/ OR "Laparoscope".mp OR "Laparoscopes".mp OR exp "Laryngoscope"/ OR "Laryngoscope".mp OR "Laryngoscopes".mp OR exp "Mediastinoscope"/ OR "Mediastinoscope".mp OR "Mediastinoscopes".mp OR exp "Neuroendoscope"/ OR "Neuroendoscope".mp OR "Neuroendoscopes".mp OR exp "Proctoscope"/ OR "Proctoscope".mp OR "Proctoscopes".mp OR exp "Sigmoidoscope"/ OR "Sigmoidoscope".mp OR "Sigmoidoscopes".mp OR exp "Thoracoscope"/ OR "Thoracoscope".mp OR "Thoracoscopes".mp))) AND ("Carbon Footprint"/ OR "carbon footprint".mp OR "carbon footprint*".mp OR exp "Climate Change"/ OR "climate change".mp OR "CO2 emission".mp OR "CO2 emissions".mp OR "CO2 footprint".mp OR "CO2 footprint*".mp OR exp "environmental protection"/ OR "conservation of natural resources".mp OR "environmental protection".mp OR "ecological footprint".mp OR "ecological footprint*".mp OR "ecological sustainability".mp OR exp "environmental impact"/ OR "environmental impact".mp OR "environmental impact*".mp OR "environmental impacts".mp OR "environmental pollut*".mp OR exp "pollution"/ OR "environmental pollution".mp OR "environmental sustainab*".mp OR "environmental sustainability"/ OR "environmental sustainability".mp OR "Global Warming"/ OR "Global Warming".mp OR "Greenhouse Effect"/ OR "greenhouse effect*".mp OR "greenhouse effects".mp OR "greenhouse gas emission".mp OR "greenhouse gas emissions".mp OR "Greenhouse Gas"/ OR "greening".mp OR "hospital waste".mp OR "life cycle assessment"/ OR "environmental impact assessment"/ OR "life cycle assess*".mp OR "life cycle assessment".mp OR "LCA".mp OR "LCAs".mp OR "life cycle inventory".mp OR "life cycle inventories".mp OR exp "Waste Disposal"/ OR exp "Hospital Waste"/ OR "medical waste".mp OR "Rising Sea Level".mp OR "Rising Sea Levels".mp OR "Sea Level Rise"/ OR "Sea Level Rise".mp OR "sustainability"**.mp** OR "Waste Disposal".mp OR "waste water recycling"/ OR exp "Waste Disposal"/ OR exp "Waste Management"/ OR "Plastic overuse".mp OR "Green surgery".mp OR "Emission reduction".mp OR "Emission reduction strategy".mp OR "air pollution control"/ OR "air pollution control".mp **OR "Environment"/ OR "Environmental*".mp** OR "acidification"/ OR "soil acidification"/ OR "ocean acidification"/ OR "acidification".mp OR "Acidification potential".mp OR "AP in kg SO2 equivalents".mp OR "eco-efficiency".mp OR "ecoefficiency".mp OR "eco-efficien*".mp OR "ecoefficien*".mp OR "ecotoxicity"/ OR "ecotoxicity".mp OR "ecotoxic*".mp OR "eco toxicity".mp OR "eco toxic*".mp OR "EP in kg PO4 equivalent".mp OR exp "Eutrophication"/ OR "eutrophication".mp OR "Eutrophication potential".mp OR "FAETP in kg DCB equivalent".mp OR "Freshwater Aquatic Ecotoxicity Potential".mp OR "GWP in kg CO2 equivalents".mp OR "H+ moles equivalents".mp OR "HTTP in kg Dichlorobenzene equivalent".mp OR "Human Toxicity Potential".mp OR "kg 2.4-D equivalents".mp OR "kg CFC-11 equivalent".mp OR "kg N equivalents".mp OR "kg NOx equivalents".mp OR "life cycle analysis".mp OR "ozone depletion".mp OR "Photochemical Ozone Depletion Potential".mp OR "POCP in kg ethane equivalent".mp OR "smog".mp OR exp "Waste"/ OR "waste".mp OR "wastes".mp OR "Ozone Depletion"/ OR "Smog"/ OR "Greenhouse Gases".mp OR "Greenhouse Gas".mp OR "SO2 equiv*".mp OR "CO2 equiva*".mp OR "CFC-11 equiv*".mp OR "N equiv*".mp OR exp "Biodiversity"/ OR "Biodiversity".mp OR "Climatic change".mp OR "Green deal".mp OR "preservation of natural resources".mp OR "Refuse Disposal".mp OR exp "Wastewater"/ OR "Waste Water".mp OR "Wastewater".mp OR exp "Water Management"/ OR "Water Purification".mp OR (("plastic*".mp OR "microplastic*") AND ("soop".mp OR "soup".mp OR "pollution".mp OR "overuse".mp OR "contamination".mp)) OR "Sustainable Development"/ OR "Sustainable Development".mp OR (("Plastic".mp OR "plastics".mp) AND "overuse".mp) OR ("hydrogen*".mp AND "moles".mp AND "equiv*".mp) OR ("Dichlorobenzen*".mp AND "equiv*".mp) OR ("2,4-D".mp AND "equiv*".mp) OR ("NOx".mp AND "equiv*".mp) OR ("ethane".mp AND "equiv*".mp) OR ("PO4".mp AND "equiv*".mp) OR ("DCB".mp AND "equiv*".mp) OR ("sustainability".mp AND ("environment*".mp OR "carbon".mp)) OR (("Carbon Dioxide"/ OR "Carbon Dioxide".mp OR "CO2".mp) AND ("pollution".mp OR "emission".mp OR "emissions".mp OR "waste".mp OR "environment".mp OR "environmental*".mp OR "footprint".mp OR "footprint*".mp OR "sustainable".mp OR "hazard".mp OR "hazard*".mp))) NOT (conference review or conference abstract).pt AND (2000 OR 2001 OR 2002 OR 2003 OR 2004 OR 2005 OR 2006 OR 2007 OR 2008 OR 2009 OR 2010 OR 2011 OR 2012 OR 2013 OR 2014 OR 2015 OR 2016 OR 2017 OR 2018 OR 2019 OR 2020 OR 2021 OR 2022).yr) AND **(***(exp "meta analysis"/ OR exp "meta analysis (topic)"/ OR metaanaly*.ti,ab OR "meta analy*".ti,ab OR metanaly*.ti,ab OR "systematic review"/ OR "cochrane database of systematic reviews".jn OR prisma.ti,ab OR prospero.ti,ab OR (((systemati* OR scoping OR umbrella OR "structured literature") ADJ3 (review* OR overview*)).ti,ab) OR ((systemic* ADJ1 review*).ti,ab) OR (((systemati* OR literature OR database* OR "data base*") ADJ10 search*).ti,ab) OR (((structured OR comprehensive* OR systemic*) ADJ3 search*).ti,ab) OR (((literature ADJ3 review*).ti,ab) AND (search*.ti,ab OR database*.ti,ab OR "data base*".ti,ab)) OR (("data extraction".ti,ab OR "data source*".ti,ab) AND "study selection".ti,ab) OR ("search strategy".ti,ab AND "selection criteria".ti,ab) OR ("data source*".ti,ab AND "data synthesis".ti,ab) OR medline.ab OR pubmed.ab OR embase.ab OR cochrane.ab OR (((critical OR rapid) ADJ2 (review* OR overview* OR synthes*)).ti) OR ((((critical* OR rapid*) ADJ3 (review* OR overview* OR synthes*)).ab) AND (search*.ab OR database*.ab OR "data base*".ab)) OR metasynthes*.ti,ab OR "meta synthes*".ti,ab)* OR (exp "clinical trial"/ OR exp "randomization"/ OR exp "single blind procedure"/ OR exp "double blind procedure"/ OR exp "crossover procedure"/ OR exp "placebo"/ OR exp "prospective study"/ OR rct.ti,ab OR random*.ti,ab OR "single blind".ti,ab OR "randomised controlled trial".ti,ab OR exp "randomized controlled trial"/ OR placebo*.ti,ab) **OR (exp "Comparative Study"/ OR "comparison".ti,ab OR "comparative".ti,ab OR "compar*".ti,ab OR "major clinical study"/ OR "clinical study"/ OR "case control study"/ OR "family study"/ OR "longitudinal study"/ OR "retrospective study"/ OR "prospective study"/ OR "cohort analysis"/ OR cohort*.ti,ab OR (("case control" ADJ1 (study OR studies)).ti,ab) OR (("follow up" ADJ1 (study OR studies)).ti,ab) OR (observational ADJ1 (study OR studies)) OR ((epidemiologic ADJ1 (study OR studies)).ti,ab) OR (("cross sectional" ADJ1 (study OR studies)).ti,ab)) OR** ("life cycle assessment"/ OR "environmental impact assessment"/ OR "life cycle assess*".mp OR "life cycle assessment".mp OR "life cycle inventory".mp OR "LCA".mp OR "LCAs".mp OR "life cycle inventory".mp OR "life cycle inventories".mp)**)**

### Topic 3: (Cover) materials

**MEDLINE (PubMed)**

(("Operating Tables"[Mesh] OR "Operating Tables"[tw] OR "Operating Table"[tw] OR "Operating Room Tables"[tw] OR "Operating Room Table"[tw] OR "Operation Tables"[tw] OR "Operation Table"[tw] OR "Operation Room Tables"[tw] OR "Operation Room Table"[tw] OR "Operating Rooms"[Mesh] OR "Operating Rooms"[tw] OR "Operating Room"[tw] OR "Operation Rooms"[tw] OR "Operation Room"[tw] OR "Operating Theatres"[tw] OR "Operating Theatre"[tw] OR "Operation Theatres"[tw] OR "Operation Theatre"[tw] OR "Operating Theaters"[tw] OR "Operating Theater"[tw] OR "Operation Theaters"[tw] OR "Operation Theater"[tw] OR "Perioperative Period"[Mesh] OR "Perioperative Care"[Mesh] OR "Perioperative Nursing"[Mesh] OR "Perioperative Medicine"[Mesh] OR "Perioperative"[tw] OR "Perioperativ*"[tw] OR "Peri operative"[tw] OR "Peri operativ*"[tw] OR "Intraoperative"[tw] OR "Operative"[tw] OR "Postoperative"[tw] OR "Preoperative"[tw] OR "Intra operative"[tw] OR "Post operative"[tw] OR "Pre operative"[tw] OR "Intraoperativ*"[tw] OR "Operativ*"[tw] OR "Postoperativ*"[tw] OR "Preoperativ*"[tw] OR "Intra operativ*"[tw] OR "Post operativ*"[tw] OR "Pre operativ*"[tw] OR "Surgical Equipment"[Mesh] OR "surgery"[subheading] OR "surgery"[tw] OR "Surgical Procedures, Operative"[mesh] OR "surgical*"[tw] OR "Surgeons"[mesh] OR "surgeon"[tw] OR "surgeons"[tw] OR "Specialties, Surgical"[Mesh]) AND ("bair hugger"[tw] OR "bair hugger*"[tw] OR "Textiles"[Mesh] OR "Textiles"[tw] OR "Textile"[tw] OR "Bedding and Linens"[Mesh] OR "Bedding"[tw] OR "Beddings"[tw] OR "Linens"[tw] OR "Linen"[tw] OR "blanket"[tw] OR "blankets"[tw] OR "Surgical Drapes"[Mesh] OR "Surgical Drapes"[tw] OR "Surgical Drape"[tw] OR "Drapes"[tw] OR "Drape"[tw] OR "cloth"[tw] OR "cloths"[tw] OR "tablecloths"[tw] OR "tablecloth"[tw] OR "bedsheets"[tw] OR "bedsheet"[tw] OR "bed sheets"[tw] OR "bed sheet"[tw] OR "fiber mats"[tw] OR "fiber mat"[tw] OR "fibre mats"[tw] OR "fibre mat"[tw] OR "fiber pads"[tw] OR "fiber pad"[tw] OR "fibre pads"[tw] OR "fibre pad"[tw] OR "fiberpads"[tw] OR "fiberpad"[tw] OR "fibrepads"[tw] OR "fibrepad"[tw] OR "fibermats"[tw] OR "fibermat"[tw] OR "fibremats"[tw] OR "fibremat"[tw] OR "coverlets"[tw] OR "coverlet"[tw] OR "cellulose pads"[tw] OR "cellulose pad"[tw] OR "cellulose mats"[tw] OR "cellulose mat"[tw] OR (("cellulose"[tw] OR "fibre"[tw] OR "fibres"[tw] OR "fiber"[tw] OR "fibers"[tw]) AND ("pad"[tw] OR "pads"[tw] OR "mat"[tw] OR "mats"[tw]))) AND ("Acidification potential"[tw] OR "acidification"[tw] OR "air pollution control"[tw] OR "AP in kg SO2 equivalents"[tw] OR "Biodiversity"[Mesh] OR "Biodiversity"[tw] OR "Carbon Footprint"[mesh] OR "carbon footprint"[tw] OR "carbon footprint*"[tw] OR "CFC-11 equiv*"[tw] OR "Climate Change"[Mesh] OR "climate change"[tw] OR "Climatic change"[tw] OR "CO2 emission"[tw] OR "CO2 emissions"[tw] OR "CO2 equiva*"[tw] OR "CO2 footprint"[tw] OR "CO2 footprint*"[tw] OR "conservation of natural resources"[mesh] OR "conservation of natural resources"[tw] OR "Disposable Equipment"[Mesh] OR "Disposable"[tw] OR "Disposables"[tw] OR "eco toxic*"[tw] OR "eco toxicity"[tw] OR "ecoefficien*"[tw] OR "eco-efficien*"[tw] OR "ecoefficiency"[tw] OR "eco-efficiency"[tw] OR "ecological footprint"[tw] OR "ecological footprint*"[tw] OR "ecological sustainability"[tw] OR "ecotoxic*"[tw] OR "ecotoxicity"[tw] OR "Emission reduction strategy"[tw] OR "Emission reduction"[tw] OR **"Environment"[Mesh:noexp]** OR "environmental impact"[tw] OR "environmental impact*"[tw] OR "environmental impacts"[tw] OR "environmental pollut*"[tw] OR "Environmental Pollution"[Mesh] OR "environmental pollution"[tw] OR "environmental protection"[tw] OR "environmental sustainab*"[tw] OR "environmental sustainability"[tw] OR **"Environmental*"[ti]** OR "EP in kg PO4 equivalent"[tw] OR "Equipment reuse"[mesh] OR "Equipment reuse"[tw] OR "Eutrophication potential"[tw] OR "Eutrophication"[Mesh] OR "eutrophication"[tw] OR "FAETP in kg DCB equivalent"[tw] OR "Freshwater Aquatic Ecotoxicity Potential"[tw] OR "Global Warming"[mesh] OR "Global Warming"[tw] OR "Green deal"[tw] OR "Green surgery"[tw] OR "Greenhouse Effect"[mesh] OR "greenhouse effect*"[tw] OR "greenhouse effects"[tw] OR "greenhouse gas emission"[tw] OR "greenhouse gas emissions"[tw] OR "Greenhouse Gas"[tw] OR "Greenhouse Gases"[mesh] OR "Greenhouse Gases"[tw] OR "greening"[tw] OR "GWP in kg CO2 equivalents"[tw] OR "H+ moles equivalents"[tw] OR "hospital waste"[tw] OR "HTTP in kg Dichlorobenzene equivalent"[tw] OR "Human Toxicity Potential"[tw] OR "kg 2.4-D equivalents"[tw] OR "kg CFC-11 equivalent"[tw] OR "kg N equivalents"[tw] OR "kg NOx equivalents"[tw] OR "LCA"[tw] OR "LCAs"[tw] OR "life cycle analysis"[tw] OR "life cycle assess*"[tw] OR "life cycle assessment"[tw] OR "life cycle inventories"[tw] OR "life cycle inventory"[tw] OR "Medical Waste Disposal"[mesh] OR "Medical Waste"[mesh] OR "medical waste"[tw] OR "N equiv*"[tw] OR "Ozone Depletion"[Mesh] OR "ozone depletion"[tw] OR "Photochemical Ozone Depletion Potential"[tw] OR "Plastic overuse"[tw] OR "POCP in kg ethane equivalent"[tw] OR "preservation of natural resources"[tw] OR "recycle*"[tw] OR "Recycling"[mesh] OR "recycling"[tw] OR "Refuse Disposal"[Mesh] OR "Refuse Disposal"[tw] OR "reusable"[tw] OR "Reusables"[tw] OR "reuse"[tw] OR "reused"[tw] OR "reusing"[tw] OR "Rising Sea Level"[tw] OR "Rising Sea Levels"[tw] OR "Sea Level Rise"[mesh] OR "Sea Level Rise"[tw] OR "Smog"[mesh] OR "smog"[tw] OR "SO2 equiv*"[tw] OR "sustainability"**[ti]** OR "Sustainable Development"[Mesh] OR "Sustainable Development"[tw] OR "Waste Disposal"[tw] OR "Waste Disposal, Fluid"[mesh] OR "Waste Management"[mesh] OR "Waste"[tw] OR "waste"[tw] OR "Waste Water"[Mesh] OR "Waste Water"[tw] OR "wastes"[tw] OR "Wastewater"[tw] OR "Water Purification"[Mesh] OR "Water Purification"[tw] OR (("plastic*"[tw] OR "microplastic*") AND ("soop"[tw] OR "soup"[tw] OR "pollution"[tw] OR "overuse"[tw] OR "contamination"[tw])) OR (("Plastic"[tw] OR "plastics"[tw]) AND "overuse"[tw]) OR ("hydrogen*"[tw] AND "moles"[tw] AND "equiv*"[tw]) OR ("Dichlorobenzen*"[tw] AND "equiv*"[tw]) OR ("2,4-D"[tw] AND "equiv*"[tw]) OR ("NOx"[tw] AND "equiv*"[tw]) OR ("ethane"[tw] AND "equiv*"[tw]) OR ("PO4"[tw] AND "equiv*"[tw]) OR ("DCB"[tw] AND "equiv*"[tw]) OR ("sustainability"[tw] AND ("environment*"[tw] OR "carbon"[tw])) OR (("Carbon Dioxide"[mesh] OR "Carbon Dioxide"[tw] OR "CO2"[tw]) AND ("pollution"[tw] OR "emission"[tw] OR "emissions"[tw] OR "waste"[tw] OR "environment"[tw] OR "environmental*"[tw] OR "footprint"[tw] OR "footprint*"[tw] OR "sustainable"[tw] OR "hazard"[tw] OR "hazard*"[tw]))) AND ("1980/01/01"[PDAT] : "3000/12/31"[PDAT])) AND **(***("Meta-Analysis"[Publication Type] OR "Meta-Analysis as Topic"[Mesh] OR metaanaly*[tiab] OR meta-analy*[tiab] or metanaly*[tiab] OR "Systematic Review"[Publication Type] OR systematic[sb] OR "Cochrane Database Syst Rev"[Journal] or prisma[tiab] OR preferred reporting items[tiab] OR prospero[tiab] OR ((systemati*[ti] OR scoping[ti] OR umbrella[ti] OR structured literature[ti]) AND (review*[ti] OR overview*[ti])) OR systematic review*[tiab] OR scoping review*[tiab] OR umbrella review*[tiab] OR structured literature review*[tiab] OR systematic qualitative review*[tiab] OR systematic quantitative review*[tiab] OR systematic search and review[tiab] OR systematized review[tiab] OR systematised review[tiab] OR systemic review[tiab] OR systematic literature review*[tiab] OR systematic integrative literature review*[tiab] OR systematically review*[tiab] OR scoping literature review*[tiab] OR systematic critical review[tiab] OR systematic integrative review*[tiab] OR systematic evidence review[tiab] OR Systematic integrative literature review*[tiab] OR Systematic mixed studies review*[tiab] OR Systematized literature review*[tiab] OR Systematic overview*[tiab] OR Systematic narrative review*[tiab] OR ((systemati*[tiab] OR literature[tiab] OR database*[tiab] OR data-base*[tiab] OR structured[tiab] OR comprehensive*[tiab] OR systemic*[tiab]) AND search*[tiab]) OR (Literature[ti] AND review[ti] AND (database*[tiab] OR data-base*[tiab] OR search*[tiab])) OR ((data extraction[tiab] OR data source*[tiab]) AND study selection[tiab]) OR (search strategy[tiab] AND selection criteria[tiab]) OR (data source*[tiab] AND data synthesis[tiab]) OR medline[tiab] OR pubmed[tiab] OR embase[tiab] OR Cochrane[tiab] OR ((critical[ti] OR rapid[ti]) AND (review*[ti] OR overview*[ti] OR synthes*[ti])) OR (((critical*[tiab] OR rapid*[tiab]) AND (review*[tiab] OR overview*[tiab] OR synthes*[tiab]) AND (search*[tiab] OR database*[tiab] OR data-base*[tiab]))) OR metasynthes*[tiab] OR meta-synthes*[tiab])* **OR** ("Randomized Controlled Trial"[Publication Type] OR random*[tiab] OR pragmatic clinical trial*[tiab] OR practical clinical trial*[tiab] OR non-inferiority trial*[tiab] OR noninferiority trial*[tiab] OR superiority trial*[tiab] OR equivalence clinical trial*[tiab]) NOT (("Animals"[Mesh]) OR "Models, Animal"[Mesh] NOT humans[mh]) NOT (letter[pt] OR comment[pt] OR editorial[pt]) **OR ("Comparative Study"[Publication Type] OR "comparison"[tiab] OR "comparative"[tiab] OR "compar*"[tiab] OR "Epidemiologic studies"[mesh:noexp] OR "case control studies"[mesh] OR "cohort studies"[mesh] OR "Controlled Before-After Studies"[mesh] OR "Case control"[tw] OR cohort*[tw] OR "Cohort analy*"[tw] OR "Follow up stud*"[tw] OR "observational stud*"[tw] OR Longitudinal[tw] OR Retrospective*[tw] OR prospective*[tw] OR consecutive*[tw] OR Cross sectional[tw] OR "Cross-sectional studies"[mesh] OR "historically controlled study"[mesh] OR "interrupted time series analysis"[mesh]) OR** ("life cycle assess*"[tw] OR "life cycle assessment"[tw] OR "life cycle inventory"[tw] OR "LCA"[tw] OR "LCAs"[tw] OR "life cycle inventory"[tw] OR "life cycle inventories"[tw])**)**

**Embase (OVID)**

((exp "Operating Table"/ OR "Operating Tables".mp OR "Operating Table".mp OR "Operating Room Tables".mp OR "Operating Room Table".mp OR "Operation Tables".mp OR "Operation Table".mp OR "Operation Room Tables".mp OR "Operation Room Table".mp OR exp "Operating Room"/ OR "Operating Rooms".mp OR "Operating Room".mp OR "Operation Rooms".mp OR "Operation Room".mp OR "Operating Theatres".mp OR "Operating Theatre".mp OR "Operation Theatres".mp OR "Operation Theatre".mp OR "Operating Theaters".mp OR "Operating Theater".mp OR "Operation Theaters".mp OR "Operation Theater".mp OR exp "Perioperative Period"/ OR exp "Perioperative Nursing"/ OR exp "Perioperative Medicine"/ OR "Perioperative".mp OR "Perioperativ*".mp OR "Peri operative".mp OR "Peri operativ*".mp OR exp "intraoperative period"/ OR "Intraoperative".mp OR "Operative".mp OR exp "postoperative period"/ OR exp "postoperative care"/ OR "Postoperative".mp OR exp "preoperative period"/ OR exp "preoperative care"/ OR "Preoperative".mp OR "Intra operative".mp OR "Post operative".mp OR "Pre operative".mp OR "Intraoperativ*".mp OR "Operativ*".mp OR "Postoperativ*".mp OR "Preoperativ*".mp OR "Intra operativ*".mp OR "Post operativ*".mp OR "Pre operativ*".mp OR exp "Surgical Equipment"/ OR "surgery".fs OR "surgery".mp OR exp "Surgery"/ OR "surgical*".mp OR exp "Surgeon"/ OR "surgeon".mp OR "surgeons".mp) AND ("bair hugger".mp OR "bair hugger*".mp OR "forced-air warming system"/ OR exp "Textile"/ OR "Textiles".mp OR "Textile".mp OR "Bedlinen"/ OR "Bedding".mp OR "Beddings".mp OR "Linens".mp OR "Linen".mp OR "blanket".mp OR "blankets".mp OR exp "Surgical Drape"/ OR "Surgical Drapes".mp OR "Surgical Drape".mp OR "Drapes".mp OR "Drape".mp OR "cloth".mp OR "cloths".mp OR "tablecloth".mp OR "tablecloths".mp OR "bedsheets".mp OR "bedsheet".mp OR "bed sheets".mp OR "bed sheet".mp OR "fiber mats".mp OR "fiber mat".mp OR "fibre mats".mp OR "fibre mat".mp OR "fiber pads".mp OR "fiber pad".mp OR "fibre pads".mp OR "fibre pad".mp OR "fiberpads".mp OR "fiberpad".mp OR "fibrepads".mp OR "fibrepad".mp OR "fibermats".mp OR "fibermat".mp OR "fibremats".mp OR "fibremat".mp OR "coverlets".mp OR "coverlet".mp OR "cellulose pads".mp OR "cellulose pad".mp OR "cellulose mats".mp OR "cellulose mat".mp OR (("cellulose".mp OR "fibre".mp OR "fibres".mp OR "fiber".mp OR "fibers".mp) AND ("pad".mp OR "pads".mp OR "mat".mp OR "mats".mp))) AND ("Carbon Footprint"/ OR "carbon footprint".mp OR "carbon footprint*".mp OR exp "Climate Change"/ OR "climate change".mp OR "CO2 emission".mp OR "CO2 emissions".mp OR "CO2 footprint".mp OR "CO2 footprint*".mp OR exp "environmental protection"/ OR "conservation of natural resources".mp OR "environmental protection".mp OR "Disposable Equipment"/ OR "Disposables".mp OR "Disposable".mp OR "ecological footprint".mp OR "ecological footprint*".mp OR "ecological sustainability".mp OR exp "environmental impact"/ OR "environmental impact".mp OR "environmental impact*".mp OR "environmental impacts".mp OR "environmental pollut*".mp OR exp "pollution"/ OR "environmental pollution".mp OR "environmental sustainab*".mp OR "environmental sustainability"/ OR "environmental sustainability".mp OR "Global Warming"/ OR "Global Warming".mp OR "Greenhouse Effect"/ OR "greenhouse effect*".mp OR "greenhouse effects".mp OR "greenhouse gas emission".mp OR "greenhouse gas emissions".mp OR "Greenhouse Gas"/ OR "greening".mp OR "hospital waste".mp OR "life cycle assessment"/ OR "environmental impact assessment"/ OR "life cycle assess*".mp OR "life cycle assessment".mp OR "LCA".mp OR "LCAs".mp OR "life cycle inventory".mp OR "life cycle inventories".mp OR exp "Waste Disposal"/ OR exp "Hospital Waste"/ OR "medical waste".mp OR "Rising Sea Level".mp OR "Rising Sea Levels".mp OR "Sea Level Rise"/ OR "Sea Level Rise".mp OR "sustainability"**.ti** OR "Waste Disposal".mp OR "waste water recycling"/ OR "Recycling"/ OR "recycling".mp OR "recycle*".mp OR "Equipment reuse".mp OR "Reusables".mp OR "reusable".mp OR "reuse".mp OR "reused".mp OR "reusing".mp OR exp "Waste Disposal"/ OR exp "Waste Management"/ OR "Plastic overuse".mp OR "Green surgery".mp OR "Emission reduction".mp OR "Emission reduction strategy".mp OR "air pollution control"/ OR "air pollution control".mp **OR "Environment"/ OR "Environmental*".ti** OR "acidification"/ OR "soil acidification"/ OR "ocean acidification"/ OR "acidification".mp OR "Acidification potential".mp OR "AP in kg SO2 equivalents".mp OR "eco-efficiency".mp OR "ecoefficiency".mp OR "eco-efficien*".mp OR "ecoefficien*".mp OR "ecotoxicity"/ OR "ecotoxicity".mp OR "ecotoxic*".mp OR "eco toxicity".mp OR "eco toxic*".mp OR "EP in kg PO4 equivalent".mp OR exp "Eutrophication"/ OR "eutrophication".mp OR "Eutrophication potential".mp OR "FAETP in kg DCB equivalent".mp OR "Freshwater Aquatic Ecotoxicity Potential".mp OR "GWP in kg CO2 equivalents".mp OR "H+ moles equivalents".mp OR "HTTP in kg Dichlorobenzene equivalent".mp OR "Human Toxicity Potential".mp OR "kg 2.4-D equivalents".mp OR "kg CFC-11 equivalent".mp OR "kg N equivalents".mp OR "kg NOx equivalents".mp OR "life cycle analysis".mp OR "ozone depletion".mp OR "Photochemical Ozone Depletion Potential".mp OR "POCP in kg ethane equivalent".mp OR "smog".mp OR exp "Waste"/ OR "waste".mp OR "wastes".mp OR "Ozone Depletion"/ OR "Smog"/ OR "Equipment reuse".mp OR "Greenhouse Gases".mp OR "Greenhouse Gas".mp OR "SO2 equiv*".mp OR "CO2 equiva*".mp OR "CFC-11 equiv*".mp OR "N equiv*".mp OR exp "Biodiversity"/ OR "Biodiversity".mp OR "Climatic change".mp OR "Green deal".mp OR "preservation of natural resources".mp OR "Refuse Disposal".mp OR exp "Wastewater"/ OR "Waste Water".mp OR "Wastewater".mp OR exp "Water Management"/ OR "Water Purification".mp OR (("plastic*".mp OR "microplastic*") AND ("soop".mp OR "soup".mp OR "pollution".mp OR "overuse".mp OR "contamination".mp)) OR "Sustainable Development"/ OR "Sustainable Development".mp OR (("Plastic".mp OR "plastics".mp) AND "overuse".mp) OR ("hydrogen*".mp AND "moles".mp AND "equiv*".mp) OR ("Dichlorobenzen*".mp AND "equiv*".mp) OR ("2,4-D".mp AND "equiv*".mp) OR ("NOx".mp AND "equiv*".mp) OR ("ethane".mp AND "equiv*".mp) OR ("PO4".mp AND "equiv*".mp) OR ("DCB".mp AND "equiv*".mp) OR ("sustainability".mp AND ("environment*".mp OR "carbon".mp)) OR (("Carbon Dioxide"/ OR "Carbon Dioxide".mp OR "CO2".mp) AND ("pollution".mp OR "emission".mp OR "emissions".mp OR "waste".mp OR "environment".mp OR "environmental*".mp OR "footprint".mp OR "footprint*".mp OR "sustainable".mp OR "hazard".mp OR "hazard*".mp))) NOT (conference review or conference abstract).pt AND (1980 OR 1981 OR 1982 OR 1983 OR 1984 OR 1985 OR 1986 OR 1987 OR 1988 OR 1989 OR 1990 OR 1991 OR 1992 OR 1993 OR 1994 OR 1995 OR 1996 OR 1997 OR 1998 OR 1999 OR 2000 OR 2001 OR 2002 OR 2003 OR 2004 OR 2005 OR 2006 OR 2007 OR 2008 OR 2009 OR 2010 OR 2011 OR 2012 OR 2013 OR 2014 OR 2015 OR 2016 OR 2017 OR 2018 OR 2019 OR 2020 OR 2021 OR 2022).yr) AND **(***(exp "meta analysis"/ OR exp "meta analysis (topic)"/ OR metaanaly*.ti,ab OR "meta analy*".ti,ab OR metanaly*.ti,ab OR "systematic review"/ OR "cochrane database of systematic reviews".jn OR prisma.ti,ab OR prospero.ti,ab OR (((systemati* OR scoping OR umbrella OR "structured literature") ADJ3 (review* OR overview*)).ti,ab) OR ((systemic* ADJ1 review*).ti,ab) OR (((systemati* OR literature OR database* OR "data base*") ADJ10 search*).ti,ab) OR (((structured OR comprehensive* OR systemic*) ADJ3 search*).ti,ab) OR (((literature ADJ3 review*).ti,ab) AND (search*.ti,ab OR database*.ti,ab OR "data base*".ti,ab)) OR (("data extraction".ti,ab OR "data source*".ti,ab) AND "study selection".ti,ab) OR ("search strategy".ti,ab AND "selection criteria".ti,ab) OR ("data source*".ti,ab AND "data synthesis".ti,ab) OR medline.ab OR pubmed.ab OR embase.ab OR cochrane.ab OR (((critical OR rapid) ADJ2 (review* OR overview* OR synthes*)).ti) OR ((((critical* OR rapid*) ADJ3 (review* OR overview* OR synthes*)).ab) AND (search*.ab OR database*.ab OR "data base*".ab)) OR metasynthes*.ti,ab OR "meta synthes*".ti,ab)* OR (exp "clinical trial"/ OR exp "randomization"/ OR exp "single blind procedure"/ OR exp "double blind procedure"/ OR exp "crossover procedure"/ OR exp "placebo"/ OR exp "prospective study"/ OR rct.ti,ab OR random*.ti,ab OR "single blind".ti,ab OR "randomised controlled trial".ti,ab OR exp "randomized controlled trial"/ OR placebo*.ti,ab) **OR (exp "Comparative Study"/ OR "comparison".ti,ab OR "comparative".ti,ab OR "compar*".ti,ab OR "major clinical study"/ OR "clinical study"/ OR "case control study"/ OR "family study"/ OR "longitudinal study"/ OR "retrospective study"/ OR "prospective study"/ OR "cohort analysis"/ OR cohort*.ti,ab OR (("case control" ADJ1 (study OR studies)).ti,ab) OR (("follow up" ADJ1 (study OR studies)).ti,ab) OR (observational ADJ1 (study OR studies)) OR ((epidemiologic ADJ1 (study OR studies)).ti,ab) OR (("cross sectional" ADJ1 (study OR studies)).ti,ab)) OR** ("life cycle assessment"/ OR "environmental impact assessment"/ OR "life cycle assess*".mp OR "life cycle assessment".mp OR "life cycle inventory".mp OR "LCA".mp OR "LCAs".mp OR "life cycle inventory".mp OR "life cycle inventories".mp)**)**

**Web of Science**

(TS=("Operating Tables" OR "Operating Tables" OR "Operating Table" OR "Operating Room Tables" OR "Operating Room Table" OR "Operation Tables" OR "Operation Table" OR "Operation Room Tables" OR "Operation Room Table" OR "Operating Rooms" OR "Operating Rooms" OR "Operating Room" OR "Operation Rooms" OR "Operation Room" OR "Operating Theatres" OR "Operating Theatre" OR "Operation Theatres" OR "Operation Theatre" OR "Operating Theaters" OR "Operating Theater" OR "Operation Theaters" OR "Operation Theater" OR "Perioperative Period" OR "Perioperative Care" OR "Perioperative Nursing" OR "Perioperative Medicine" OR "Perioperative" OR "Perioperativ*" OR "Peri operative" OR "Peri operativ*" OR "Intraoperative" OR "Operative" OR "Postoperative" OR "Preoperative" OR "Intra operative" OR "Post operative" OR "Pre operative" OR "Intraoperativ*" OR "Operativ*" OR "Postoperativ*" OR "Preoperativ*" OR "Intra operativ*" OR "Post operativ*" OR "Pre operativ*" OR "Surgical Equipment" OR "surgery" OR "surgery" OR "Surgical Procedures, Operative" OR "surgical*" OR "Surgeon" OR "surgeon" OR "surgeons" OR "Specialties, Surgical") AND TS=("bair hugger" OR "bair hugger*" OR "Textiles" OR "Textiles" OR "Textile" OR "Bedding and Linens" OR "Bedding" OR "Beddings" OR "Linens" OR "Linen" OR "blanket" OR "blankets" OR "Surgical Drapes" OR "Surgical Drapes" OR "Surgical Drape" OR "Drapes" OR "Drape" OR "cloths" OR "cloth" OR "tablecloths" OR "tablecloth" OR "bedsheets" OR "bedsheet" OR "bed sheets" OR "bed sheet" OR "fiber mats" OR "fiber mat" OR "fibre mats" OR "fibre mat" OR "fibermats" OR "fibermat" OR "fibremats" OR "fibremat" OR "coverlets" OR "coverlet" OR "fiber pads" OR "fiber pad" OR "fibre pads" OR "fibre pad" OR "fiberpads" OR "fiberpad" OR "fibrepads" OR "fibrepad" OR "cellulose pads" OR "cellulose pad" OR "cellulose mats" OR "cellulose mat" OR (("cellulose" OR "fibre" OR "fibres" OR "fiber" OR "fibers") AND ("pad" OR "pads" OR "mat" OR "mats"))) AND (TS=("Carbon Footprint" OR "carbon footprint" OR "carbon footprint*" OR "Climate Change" OR "climate change" OR "CO2 emission" OR "CO2 emissions" OR "CO2 footprint" OR "CO2 footprint*" OR "environmental protection" OR "conservation of natural resources" OR "environmental protection" OR "Disposable Equipment" OR "Disposables" OR "Disposable" OR "ecological footprint" OR "ecological footprint*" OR "ecological sustainability" OR "environmental impact" OR "environmental impact" OR "environmental impact*" OR "environmental impacts" OR "environmental pollut*" OR "pollution" OR "environmental pollution" OR "environmental sustainab*" OR "environmental sustainability" OR "environmental sustainability" OR "Global Warming" OR "Global Warming" OR "Greenhouse Effect" OR "greenhouse effect*" OR "greenhouse effects" OR "greenhouse gas emission" OR "greenhouse gas emissions" OR "Greenhouse Gas" OR "greening" OR "hospital waste" OR "life cycle assessment" OR "environmental impact assessment" OR "life cycle assess*" OR "life cycle assessment" OR "LCA" OR "LCAs" OR "life cycle inventory" OR "life cycle inventories" OR "Waste Disposal" OR "Hospital Waste" OR "medical waste" OR "Rising Sea Level" OR "Rising Sea Levels" OR "Sea Level Rise" OR "Sea Level Rise" OR "Waste Disposal" OR "waste water recycling" OR "Recycling" OR "recycling" OR "recycle*" OR "Equipment reuse" OR "Reusables" OR "reusable" OR "reuse" OR "reused" OR "reusing" OR "Waste Disposal" OR "Waste Management" OR "Plastic overuse" OR "Green surgery" OR "Emission reduction" OR "Emission reduction strategy" OR "air pollution control" OR "air pollution control" OR "acidification" OR "soil acidification" OR "ocean acidification" OR "acidification" OR "Acidification potential" OR "AP in kg SO2 equivalents" OR "eco-efficiency" OR "ecoefficiency" OR "eco-efficien*" OR "ecoefficien*" OR "ecotoxicity" OR "ecotoxicity" OR "ecotoxic*" OR "eco toxicity" OR "eco toxic*" OR "EP in kg PO4 equivalent" OR "Eutrophication" OR "eutrophication" OR "Eutrophication potential" OR "FAETP in kg DCB equivalent" OR "Freshwater Aquatic Ecotoxicity Potential" OR "GWP in kg CO2 equivalents" OR "H+ moles equivalents" OR "HTTP in kg Dichlorobenzene equivalent" OR "Human Toxicity Potential" OR "kg 2.4-D equivalents" OR "kg CFC-11 equivalent" OR "kg N equivalents" OR "kg NOx equivalents" OR "life cycle analysis" OR "ozone depletion" OR "Photochemical Ozone Depletion Potential" OR "POCP in kg ethane equivalent" OR "smog" OR "Waste" OR "waste" OR "wastes" OR "Ozone Depletion" OR "Smog" OR "Equipment reuse" OR "Greenhouse Gases" OR "Greenhouse Gas" OR "SO2 equiv*" OR "CO2 equiva*" OR "CFC-11 equiv*" OR "N equiv*" OR "Sustainable Development" OR "Sustainable Development" OR "Biodiversity" OR "Climatic change" OR "Green deal" OR "preservation of natural resources" OR "Refuse Disposal" OR "Waste Water" OR "Wastewater" OR "Water Purification" OR (("plastic*" OR "microplastic*") AND ("soop" OR "soup" OR "pollution" OR "overuse" OR "contamination")) OR (("Plastic" OR "plastics") AND "overuse") OR ("hydrogen*" AND "moles" AND "equiv*") OR ("Dichlorobenzen*" AND "equiv*") OR ("2,4-D" AND "equiv*") OR ("NOx" AND "equiv*") OR ("ethane" AND "equiv*") OR ("PO4" AND "equiv*") OR ("DCB" AND "equiv*") OR ("sustainability" AND ("environment*" OR "carbon")) OR (("Carbon Dioxide" OR "Carbon Dioxide" OR "CO2") AND ("pollution" OR "emission" OR "emissions" OR "waste" OR "environment" OR "environmental*" OR "footprint" OR "footprint*" OR "sustainable" OR "hazard" OR "hazard*"))) OR **TI=("environmental*" OR "sustainab*")**) NOT DT=(meeting abstract) AND PY=(1980 OR 1981 OR 1982 OR 1983 OR 1984 OR 1985 OR 1986 OR 1987 OR 1988 OR 1989 OR 1990 OR 1991 OR 1992 OR 1993 OR 1994 OR 1995 OR 1996 OR 1997 OR 1998 OR 1999 OR 2000 OR 2001 OR 2002 OR 2003 OR 2004 OR 2005 OR 2006 OR 2007 OR 2008 OR 2009 OR 2010 OR 2011 OR 2012 OR 2013 OR 2014 OR 2015 OR 2016 OR 2017 OR 2018 OR 2019 OR 2020 OR 2021 OR 2022)) AND **(**TI=**(***("Meta-Analysis" OR metaanaly* OR "meta-analy*" or metanaly* OR "Systematic Review" OR "Cochrane Database Syst Rev" OR "prisma" OR "preferred reporting items" OR "prospero" OR ((systemati* OR scoping OR umbrella OR "structured literature") NEAR/4 (review* OR overview*)) OR "systematic review*" OR "scoping review*" OR "umbrella review*" OR "structured literature review*" OR "systematic qualitative review*" OR "systematic quantitative review*" OR "systematic search and review" OR "systematized review" OR "systematised review" OR "systemic review" OR "systematic literature review*" OR "systematic integrative literature review*" OR "systematically review*" OR "scoping literature review*" OR "systematic critical review" OR "systematic integrative review*" OR "systematic evidence review" OR "Systematic integrative literature review*" OR "Systematic mixed studies review*" OR "Systematized literature review*" OR "Systematic overview*" OR "Systematic narrative review*" OR (("systemati*" OR "literature" OR "database*" OR "data-base*" OR "structured" OR "comprehensive*" OR "systemic*") NEAR/4 "search*") OR ("Literature" AND "review" AND ("database*" OR "data-base*" OR "search*")) OR (("data extraction" OR "data source*") AND "study selection") OR ("search strategy" AND "selection criteria") OR ("data source*" AND "data synthesis") OR "medline" OR "pubmed" OR "embase" OR "Cochrane" OR (("critical" OR "rapid") NEAR/4 ("review*" OR "overview*" OR "synthes*")) OR ((("critical*" OR "rapid*") NEAR/4 ("review*" OR "overview*" OR "synthes*") NEAR/4 ("search*" OR "database*" OR "data-base*"))) OR metasynthes* OR "meta-synthes*")* **OR** ("Randomized Controlled Trial" OR random* OR "RCT"OR "RCTs" OR "pragmatic clinical trial*" OR "practical clinical trial*" OR "non-inferiority trial*" OR "noninferiority trial*" OR "superiority trial*" OR "equivalence clinical trial*") **OR ("Comparative Study" OR "comparison" OR "comparative" OR "compar*" OR "major clinical study" OR "clinical study" OR "case control study" OR "family study" OR "longitudinal study" OR "retrospective study" OR "prospective study" OR "cohort analysis" OR cohort* OR (("case control" NEAR/1 (study OR studies))) OR (("follow up" NEAR/1 (study OR studies))) OR (observational NEAR/1 (study OR studies)) OR ((epidemiologic NEAR/1 (study OR studies))) OR (("cross sectional" NEAR/1 (study OR studies)))) OR** ("life cycle assess*" OR "life cycle assessment" OR "life cycle inventory" OR "LCA" OR "LCAs" OR "life cycle inventory" OR "life cycle inventories")**)** OR AB=**(***("Meta-Analysis" OR metaanaly* OR "meta-analy*" or metanaly* OR "Systematic Review" OR "Cochrane Database Syst Rev" OR "prisma" OR "preferred reporting items" OR "prospero" OR ((systemati* OR scoping OR umbrella OR "structured literature") NEAR/4 (review* OR overview*)) OR "systematic review*" OR "scoping review*" OR "umbrella review*" OR "structured literature review*" OR "systematic qualitative review*" OR "systematic quantitative review*" OR "systematic search and review" OR "systematized review" OR "systematised review" OR "systemic review" OR "systematic literature review*" OR "systematic integrative literature review*" OR "systematically review*" OR "scoping literature review*" OR "systematic critical review" OR "systematic integrative review*" OR "systematic evidence review" OR "Systematic integrative literature review*" OR "Systematic mixed studies review*" OR "Systematized literature review*" OR "Systematic overview*" OR "Systematic narrative review*" OR (("systemati*" OR "literature" OR "database*" OR "data-base*" OR "structured" OR "comprehensive*" OR "systemic*") NEAR/4 "search*") OR ("Literature" AND "review" AND ("database*" OR "data-base*" OR "search*")) OR (("data extraction" OR "data source*") AND "study selection") OR ("search strategy" AND "selection criteria") OR ("data source*" AND "data synthesis") OR "medline" OR "pubmed" OR "embase" OR "Cochrane" OR (("critical" OR "rapid") NEAR/4 ("review*" OR "overview*" OR "synthes*")) OR ((("critical*" OR "rapid*") NEAR/4 ("review*" OR "overview*" OR "synthes*") NEAR/4 ("search*" OR "database*" OR "data-base*"))) OR metasynthes* OR "meta-synthes*")* **OR** ("Randomized Controlled Trial" OR random* OR "RCT"OR "RCTs" OR "pragmatic clinical trial*" OR "practical clinical trial*" OR "non-inferiority trial*" OR "noninferiority trial*" OR "superiority trial*" OR "equivalence clinical trial*") **OR ("Comparative Study" OR "comparison" OR "comparative" OR "compar*" OR "major clinical study" OR "clinical study" OR "case control study" OR "family study" OR "longitudinal study" OR "retrospective study" OR "prospective study" OR "cohort analysis" OR cohort* OR (("case control" NEAR/1 (study OR studies))) OR (("follow up" NEAR/1 (study OR studies))) OR (observational NEAR/1 (study OR studies)) OR ((epidemiologic NEAR/1 (study OR studies))) OR (("cross sectional" NEAR/1 (study OR studies)))) OR** ("life cycle assess*" OR "life cycle assessment" OR "life cycle inventory" OR "LCA" OR "LCAs" OR "life cycle inventory" OR "life cycle inventories")**))**

**Cochrane Library**

("Operating Tables" OR "Operating Tables" OR "Operating Table" OR "Operating Room Tables" OR "Operating Room Table" OR "Operation Tables" OR "Operation Table" OR "Operation Room Tables" OR "Operation Room Table" OR "Operating Rooms" OR "Operating Rooms" OR "Operating Room" OR "Operation Rooms" OR "Operation Room" OR "Operating Theatres" OR "Operating Theatre" OR "Operation Theatres" OR "Operation Theatre" OR "Operating Theaters" OR "Operating Theater" OR "Operation Theaters" OR "Operation Theater" OR "Perioperative Period" OR "Perioperative Care" OR "Perioperative Nursing" OR "Perioperative Medicine" OR "Perioperative" OR "Perioperativ*" OR "Peri operative" OR "Peri operativ*" OR "Intraoperative" OR "Operative" OR "Postoperative" OR "Preoperative" OR "Intra operative" OR "Post operative" OR "Pre operative" OR "Intraoperativ*" OR "Operativ*" OR "Postoperativ*" OR "Preoperativ*" OR "Intra operativ*" OR "Post operativ*" OR "Pre operativ*" OR "Surgical Equipment" OR "surgery" OR "surgery" OR "Surgical Procedures, Operative" OR "surgical*" OR "Surgeon" OR "surgeon" OR "surgeons" OR "Specialties, Surgical"):ti,ab,kw

AND

("bair hugger" OR "bair hugger*" OR "Textiles" OR "Textiles" OR "Textile" OR "Bedding and Linens" OR "Bedding" OR "Beddings" OR "Linens" OR "Linen" OR "blanket" OR "blankets" OR "Surgical Drapes" OR "Surgical Drapes" OR "Surgical Drape" OR "Drapes" OR "Drape" OR "cloths" OR "cloth" OR "tablecloths" OR "tablecloth" OR "bedsheets" OR "bedsheet" OR "bed sheets" OR "bed sheet" OR "fiber mats" OR "fiber mat" OR "fibre mats" OR "fibre mat" OR "fibermats" OR "fibermat" OR "fibremats" OR "fibremat" OR "coverlets" OR "coverlet" OR "fiber pads" OR "fiber pad" OR "fibre pads" OR "fibre pad" OR "fiberpads" OR "fiberpad" OR "fibrepads" OR "fibrepad" OR "cellulose pads" OR "cellulose pad" OR "cellulose mats" OR "cellulose mat" OR (("cellulose" OR "fibre" OR "fibres" OR "fiber" OR "fibers") AND ("pad" OR "pads" OR "mat" OR "mats"))):ti,ab,kw

AND

(("Carbon Footprint" OR "carbon footprint" OR "carbon footprint*" OR "Climate Change" OR "climate change" OR "CO2 emission" OR "CO2 emissions" OR "CO2 footprint" OR "CO2 footprint*" OR "environmental protection" OR "conservation of natural resources" OR "environmental protection" OR "Disposable Equipment" OR "Disposables" OR "Disposable" OR "ecological footprint" OR "ecological footprint*" OR "ecological sustainability" OR "environmental impact" OR "environmental impact" OR "environmental impact*" OR "environmental impacts" OR "environmental pollut*" OR "pollution" OR "environmental pollution" OR "environmental sustainab*" OR "environmental sustainability" OR "environmental sustainability" OR "Global Warming" OR "Global Warming" OR "Greenhouse Effect" OR "greenhouse effect*" OR "greenhouse effects" OR "greenhouse gas emission" OR "greenhouse gas emissions" OR "Greenhouse Gas" OR "greening" OR "hospital waste" OR "life cycle assessment" OR "environmental impact assessment" OR "life cycle assessment" OR "environmental impact assessment" OR "life cycle assess*" OR "life cycle assessment" OR "LCA" OR "LCAs" OR "life cycle inventory" OR "life cycle inventories" OR "Waste Disposal" OR "Hospital Waste" OR "medical waste" OR "Rising Sea Level" OR "Rising Sea Levels" OR "Sea Level Rise" OR "Sea Level Rise" OR "Waste Disposal" OR "waste water recycling" OR "Recycling" OR "recycling" OR "recycle*" OR "Equipment reuse" OR "Reusables" OR "reusable" OR "reuse" OR "reused" OR "reusing" OR "Waste Disposal" OR "Waste Management" OR "Plastic overuse" OR "Green surgery" OR "Emission reduction" OR "Emission reduction strategy" OR "air pollution control" OR "air pollution control" OR "acidification" OR "soil acidification" OR "ocean acidification" OR "acidification" OR "Acidification potential" OR "AP in kg SO2 equivalents" OR "eco-efficiency" OR "ecoefficiency" OR "eco-efficien*" OR "ecoefficien*" OR "ecotoxicity" OR "ecotoxicity" OR "ecotoxic*" OR "eco toxicity" OR "eco toxic*" OR "EP in kg PO4 equivalent" OR "Eutrophication" OR "eutrophication" OR "Eutrophication potential" OR "FAETP in kg DCB equivalent" OR "Freshwater Aquatic Ecotoxicity Potential" OR "GWP in kg CO2 equivalents" OR "H+ moles equivalents" OR "HTTP in kg Dichlorobenzene equivalent" OR "Human Toxicity Potential" OR "kg 2.4-D equivalents" OR "kg CFC-11 equivalent" OR "kg N equivalents" OR "kg NOx equivalents" OR "life cycle analysis" OR "ozone depletion" OR "Photochemical Ozone Depletion Potential" OR "POCP in kg ethane equivalent" OR "smog" OR "Waste" OR "waste" OR "wastes" OR "Ozone Depletion" OR "Smog" OR "Equipment reuse" OR "Greenhouse Gases" OR "Greenhouse Gas" OR "SO2 equiv*" OR "CO2 equiva*" OR "CFC-11 equiv*" OR "N equiv*" OR "Biodiversity" OR "Climatic change" OR "Green deal" OR "preservation of natural resources" OR "Refuse Disposal" OR "Waste Water" OR "Wastewater" OR "Water Purification" OR (("plastic*" OR "microplastic*") AND ("soop" OR "soup" OR "pollution" OR "overuse" OR "contamination")) OR "Sustainable Development" OR "Sustainable Development" OR (("Plastic" OR "plastics") AND "overuse") OR ("hydrogen*" AND "moles" AND "equiv*") OR ("Dichlorobenzen*" AND "equiv*") OR ("2,4-D" AND "equiv*") OR ("NOx" AND "equiv*") OR ("ethane" AND "equiv*") OR ("PO4" AND "equiv*") OR ("DCB" AND "equiv*") OR ("sustainability" AND ("environment*" OR "carbon")) OR (("Carbon Dioxide" OR "Carbon Dioxide" OR "CO2") AND ("pollution" OR "emission" OR "emissions" OR "waste" OR "environment" OR "environmental*" OR "footprint" OR "footprint*" OR "sustainable" OR "hazard" OR "hazard*"))):ti,ab,kw OR **("environmental" OR "sustainability"):ti**)

**Emcare (OVID)**

((exp "Operating Table"/ OR "Operating Tables".mp OR "Operating Table".mp OR "Operating Room Tables".mp OR "Operating Room Table".mp OR "Operation Tables".mp OR "Operation Table".mp OR "Operation Room Tables".mp OR "Operation Room Table".mp OR exp "Operating Room"/ OR "Operating Rooms".mp OR "Operating Room".mp OR "Operation Rooms".mp OR "Operation Room".mp OR "Operating Theatres".mp OR "Operating Theatre".mp OR "Operation Theatres".mp OR "Operation Theatre".mp OR "Operating Theaters".mp OR "Operating Theater".mp OR "Operation Theaters".mp OR "Operation Theater".mp OR exp "Perioperative Period"/ OR exp "Perioperative Nursing"/ OR exp "Perioperative Medicine"/ OR "Perioperative".mp OR "Perioperativ*".mp OR "Peri operative".mp OR "Peri operativ*".mp OR exp "intraoperative period"/ OR "Intraoperative".mp OR "Operative".mp OR exp "postoperative period"/ OR exp "postoperative care"/ OR "Postoperative".mp OR exp "preoperative period"/ OR exp "preoperative care"/ OR "Preoperative".mp OR "Intra operative".mp OR "Post operative".mp OR "Pre operative".mp OR "Intraoperativ*".mp OR "Operativ*".mp OR "Postoperativ*".mp OR "Preoperativ*".mp OR "Intra operativ*".mp OR "Post operativ*".mp OR "Pre operativ*".mp OR exp "Surgical Equipment"/ OR "surgery".mp OR exp "Surgery"/ OR "surgical*".mp OR exp "Surgeon"/ OR "surgeon".mp OR "surgeons".mp) AND ("bair hugger".mp OR "bair hugger*".mp OR "forced-air warming system"/ OR exp "Textile"/ OR "Textiles".mp OR "Textile".mp OR "Bedlinen"/ OR "Bedding".mp OR "Beddings".mp OR "Linens".mp OR "Linen".mp OR "blanket".mp OR "blankets".mp OR exp "Surgical Drape"/ OR "Surgical Drapes".mp OR "Surgical Drape".mp OR "Drapes".mp OR "Drape".mp OR "cloth".mp OR "cloths".mp OR "tablecloth".mp OR "tablecloths".mp OR "bedsheets".mp OR "bedsheet".mp OR "bed sheets".mp OR "bed sheet".mp OR "fiber mats".mp OR "fiber mat".mp OR "fibre mats".mp OR "fibre mat".mp OR "fiber pads".mp OR "fiber pad".mp OR "fibre pads".mp OR "fibre pad".mp OR "fiberpads".mp OR "fiberpad".mp OR "fibrepads".mp OR "fibrepad".mp OR "fibermats".mp OR "fibermat".mp OR "fibremats".mp OR "fibremat".mp OR "coverlets".mp OR "coverlet".mp OR "cellulose pads".mp OR "cellulose pad".mp OR "cellulose mats".mp OR "cellulose mat".mp OR (("cellulose".mp OR "fibre".mp OR "fibres".mp OR "fiber".mp OR "fibers".mp) AND ("pad".mp OR "pads".mp OR "mat".mp OR "mats".mp))) AND ("Carbon Footprint"/ OR "carbon footprint".mp OR "carbon footprint*".mp OR exp "Climate Change"/ OR "climate change".mp OR "CO2 emission".mp OR "CO2 emissions".mp OR "CO2 footprint".mp OR "CO2 footprint*".mp OR exp "environmental protection"/ OR "conservation of natural resources".mp OR "environmental protection".mp OR "Disposable Equipment"/ OR "Disposables".mp OR "Disposable".mp OR "ecological footprint".mp OR "ecological footprint*".mp OR "ecological sustainability".mp OR exp "environmental impact"/ OR "environmental impact".mp OR "environmental impact*".mp OR "environmental impacts".mp OR "environmental pollut*".mp OR exp "pollution"/ OR "environmental pollution".mp OR "environmental sustainab*".mp OR "environmental sustainability"/ OR "environmental sustainability".mp OR "Global Warming"/ OR "Global Warming".mp OR "Greenhouse Effect"/ OR "greenhouse effect*".mp OR "greenhouse effects".mp OR "greenhouse gas emission".mp OR "greenhouse gas emissions".mp OR "Greenhouse Gas"/ OR "greening".mp OR "hospital waste".mp OR "life cycle assessment"/ OR "environmental impact assessment"/ OR "life cycle assess*".mp OR "life cycle assessment".mp OR "LCA".mp OR "LCAs".mp OR "life cycle inventory".mp OR "life cycle inventories".mp OR exp "Waste Disposal"/ OR exp "Hospital Waste"/ OR "medical waste".mp OR "Rising Sea Level".mp OR "Rising Sea Levels".mp OR "Sea Level Rise"/ OR "Sea Level Rise".mp OR "sustainability"**.ti** OR "Waste Disposal".mp OR "waste water recycling"/ OR "Recycling"/ OR "recycling".mp OR "recycle*".mp OR "Equipment reuse".mp OR "Reusables".mp OR "reusable".mp OR "reuse".mp OR "reused".mp OR "reusing".mp OR exp "Waste Disposal"/ OR exp "Waste Management"/ OR "Plastic overuse".mp OR "Green surgery".mp OR "Emission reduction".mp OR "Emission reduction strategy".mp OR "air pollution control"/ OR "air pollution control".mp **OR "Environment"/ OR "Environmental*".ti** OR "acidification"/ OR "soil acidification"/ OR "ocean acidification"/ OR "acidification".mp OR "Acidification potential".mp OR "AP in kg SO2 equivalents".mp OR "eco-efficiency".mp OR "ecoefficiency".mp OR "eco-efficien*".mp OR "ecoefficien*".mp OR "ecotoxicity"/ OR "ecotoxicity".mp OR "ecotoxic*".mp OR "eco toxicity".mp OR "eco toxic*".mp OR "EP in kg PO4 equivalent".mp OR exp "Eutrophication"/ OR "eutrophication".mp OR "Eutrophication potential".mp OR "FAETP in kg DCB equivalent".mp OR "Freshwater Aquatic Ecotoxicity Potential".mp OR "GWP in kg CO2 equivalents".mp OR "H+ moles equivalents".mp OR "HTTP in kg Dichlorobenzene equivalent".mp OR "Human Toxicity Potential".mp OR "kg 2.4-D equivalents".mp OR "kg CFC-11 equivalent".mp OR "kg N equivalents".mp OR "kg NOx equivalents".mp OR "life cycle analysis".mp OR "ozone depletion".mp OR "Photochemical Ozone Depletion Potential".mp OR "POCP in kg ethane equivalent".mp OR "smog".mp OR exp "Waste"/ OR "waste".mp OR "wastes".mp OR "Ozone Depletion"/ OR "Smog"/ OR "Equipment reuse".mp OR "Greenhouse Gases".mp OR "Greenhouse Gas".mp OR "SO2 equiv*".mp OR "CO2 equiva*".mp OR "CFC-11 equiv*".mp OR "N equiv*".mp OR exp "Biodiversity"/ OR "Biodiversity".mp OR "Climatic change".mp OR "Green deal".mp OR "preservation of natural resources".mp OR "Refuse Disposal".mp OR exp "Wastewater"/ OR "Waste Water".mp OR "Wastewater".mp OR exp "Water Management"/ OR "Water Purification".mp OR (("plastic*".mp OR "microplastic*") AND ("soop".mp OR "soup".mp OR "pollution".mp OR "overuse".mp OR "contamination".mp)) OR "Sustainable Development"/ OR "Sustainable Development".mp OR (("Plastic".mp OR "plastics".mp) AND "overuse".mp) OR ("hydrogen*".mp AND "moles".mp AND "equiv*".mp) OR ("Dichlorobenzen*".mp AND "equiv*".mp) OR ("2,4-D".mp AND "equiv*".mp) OR ("NOx".mp AND "equiv*".mp) OR ("ethane".mp AND "equiv*".mp) OR ("PO4".mp AND "equiv*".mp) OR ("DCB".mp AND "equiv*".mp) OR ("sustainability".mp AND ("environment*".mp OR "carbon".mp)) OR (("Carbon Dioxide"/ OR "Carbon Dioxide".mp OR "CO2".mp) AND ("pollution".mp OR "emission".mp OR "emissions".mp OR "waste".mp OR "environment".mp OR "environmental*".mp OR "footprint".mp OR "footprint*".mp OR "sustainable".mp OR "hazard".mp OR "hazard*".mp))) NOT (conference review or conference abstract).pt AND (1980 OR 1981 OR 1982 OR 1983 OR 1984 OR 1985 OR 1986 OR 1987 OR 1988 OR 1989 OR 1990 OR 1991 OR 1992 OR 1993 OR 1994 OR 1995 OR 1996 OR 1997 OR 1998 OR 1999 OR 2000 OR 2001 OR 2002 OR 2003 OR 2004 OR 2005 OR 2006 OR 2007 OR 2008 OR 2009 OR 2010 OR 2011 OR 2012 OR 2013 OR 2014 OR 2015 OR 2016 OR 2017 OR 2018 OR 2019 OR 2020 OR 2021 OR 2022).yr) AND **(***(exp "meta analysis"/ OR exp "meta analysis (topic)"/ OR metaanaly*.ti,ab OR "meta analy*".ti,ab OR metanaly*.ti,ab OR "systematic review"/ OR "cochrane database of systematic reviews".jn OR prisma.ti,ab OR prospero.ti,ab OR (((systemati* OR scoping OR umbrella OR "structured literature") ADJ3 (review* OR overview*)).ti,ab) OR ((systemic* ADJ1 review*).ti,ab) OR (((systemati* OR literature OR database* OR "data base*") ADJ10 search*).ti,ab) OR (((structured OR comprehensive* OR systemic*) ADJ3 search*).ti,ab) OR (((literature ADJ3 review*).ti,ab) AND (search*.ti,ab OR database*.ti,ab OR "data base*".ti,ab)) OR (("data extraction".ti,ab OR "data source*".ti,ab) AND "study selection".ti,ab) OR ("search strategy".ti,ab AND "selection criteria".ti,ab) OR ("data source*".ti,ab AND "data synthesis".ti,ab) OR medline.ab OR pubmed.ab OR embase.ab OR cochrane.ab OR (((critical OR rapid) ADJ2 (review* OR overview* OR synthes*)).ti) OR ((((critical* OR rapid*) ADJ3 (review* OR overview* OR synthes*)).ab) AND (search*.ab OR database*.ab OR "data base*".ab)) OR metasynthes*.ti,ab OR "meta synthes*".ti,ab)* OR (exp "clinical trial"/ OR exp "randomization"/ OR exp "single blind procedure"/ OR exp "double blind procedure"/ OR exp "crossover procedure"/ OR exp "placebo"/ OR exp "prospective study"/ OR rct.ti,ab OR random*.ti,ab OR "single blind".ti,ab OR "randomised controlled trial".ti,ab OR exp "randomized controlled trial"/ OR placebo*.ti,ab) **OR (exp "Comparative Study"/ OR "comparison".ti,ab OR "comparative".ti,ab OR "compar*".ti,ab OR "major clinical study"/ OR "clinical study"/ OR "case control study"/ OR "family study"/ OR "longitudinal study"/ OR "retrospective study"/ OR "prospective study"/ OR "cohort analysis"/ OR cohort*.ti,ab OR (("case control" ADJ1 (study OR studies)).ti,ab) OR (("follow up" ADJ1 (study OR studies)).ti,ab) OR (observational ADJ1 (study OR studies)) OR ((epidemiologic ADJ1 (study OR studies)).ti,ab) OR (("cross sectional" ADJ1 (study OR studies)).ti,ab)) OR** ("life cycle assessment"/ OR "environmental impact assessment"/ OR "life cycle assess*".mp OR "life cycle assessment".mp OR "life cycle inventory".mp OR "LCA".mp OR "LCAs".mp OR "life cycle inventory".mp OR "life cycle inventories".mp)**)**

### Topic 4: Anaesthesia

**MEDLINE (PubMed)**

**UV4.1**

(((("Anesthetics, Inhalation"[Mesh] OR "Anesthetics, Inhalation"[pharmacological action] OR "Anesthesia, Inhalation"[Mesh] OR "Closed-Circuit Anesthesia"[tw] OR "Endotracheal Anesthesia"[tw] OR "Closed-Circuit Anaesthesia"[tw] OR "Endotracheal Anaesthesia"[tw] OR "Halothane"[mesh] OR "Isoflurane"[mesh] OR "Methoxyflurane"[mesh] OR "Sevoflurane"[mesh] OR "Anaesthetic gas"[tw] OR "Anaesthetic gases"[tw] OR "Anesthetic gas"[tw] OR "Anesthetic gases"[tw] OR "Desflurane"[tw] OR "Enflurane"[tw] OR "Flurane"[tw] OR "Fluranes"[tw] OR "Gaseous anaesthesia"[tw] OR "Gaseous anaesthetic"[tw] OR "Gaseous anaesthetics"[tw] OR "Gaseous anesthesia"[tw] OR "Gaseous anesthetic"[tw] OR "Gaseous anesthetics"[tw] OR "Halothane"[tw] OR "Inhalation anaesthesia"[tw] OR "inhalation anaesthetic"[tw] OR "inhalation anaesthetics"[tw] OR "Inhalation anesthesia"[tw] OR "inhalation anesthetic"[tw] OR "inhalation anesthetics"[tw] OR "Inhalational agents"[tw] OR "Inhalational anaesthesia"[tw] OR "Inhalational anaesthetic"[tw] OR "Inhalational anaesthetic agent"[tw] OR "Inhalational anaesthetic agents"[tw] OR "Inhalational anaesthetics"[tw] OR "Inhalational anesthesia"[tw] OR "Inhalational anesthetic"[tw] OR "Inhalational anesthetic agent"[tw] OR "Inhalational anesthetic agents"[tw] OR "Inhalational anesthetics"[tw] OR "Inhaled anaesthetic"[tw] OR "Inhaled anaesthetic agent"[tw] OR "Inhaled anaesthetic agents"[tw] OR "Inhaled anaesthetics"[tw] OR "Inhaled anesthetic"[tw] OR "Inhaled anesthetic agents"[tw] OR "Inhaled anesthetics"[tw] OR "insufflation anaesthesia"[tw] OR "insufflation anaesthetic"[tw] OR "insufflation anaesthetics"[tw] OR "insufflation anesthesia"[tw] OR "insufflation anesthetic"[tw] OR "insufflation anesthetics"[tw] OR "Isoflurane"[tw] OR "Methoxyflurane"[tw] OR "Sevoflurane"[tw] OR "Vapor anaesthesia"[tw] OR "Vapor anaesthetic"[tw] OR "Vapor anaesthetics"[tw] OR "Vapor anesthesia"[tw] OR "Vapor anesthetic"[tw] OR "Vapor anesthetics"[tw] OR "Volatile agent"[tw] OR "Volatile agents"[tw] OR "Volatile Anaesthetic"[tw] OR "Volatile Anaesthetics"[tw] OR "Volatile anesthetic"[tw] OR "Volatile anesthetics"[tw] OR "Volatile drug"[tw] OR "Volatile drugs"[tw] OR "Volatile fluorinated liquid"[tw] OR "Volatile fluorinated liquids"[tw] OR "Volatile gas"[tw] OR "Volatile gases"[tw] OR "Volatile liquid agent"[tw] OR "Volatile liquid agents"[tw] OR "Anesthetic Gas"[tw] OR "Anesthetic Gases"[tw] OR "Inhalation Anaesthetic"[tw] OR "Inhalation Anaesthetics"[tw] OR "Inhalation Anesthetic"[tw] OR "Inhalation Anesthetics"[tw]) AND ("Anesthesia, Intravenous"[Mesh] OR "Anesthetics, Intravenous"[Mesh] OR "Anesthetics, Intravenous"[Pharmacological Action] OR "Intravenous Anesthetics"[tw] OR "Intravenous Anaesthetics"[tw] OR "Intravenous Anesthetic"[tw] OR "Intravenous Anaesthetic"[tw] OR "Intravenous Anesthesia"[tw] OR "Intravenous Anaesthesia"[tw] OR "Dissociative Anesthetics"[tw] OR "Dissociative Anaesthetics"[tw] OR "Dissociative Anesthetic"[tw] OR "Dissociative Anaesthetic"[tw] OR "Dissociative Anesthesia"[tw] OR "Dissociative Anaesthesia"[tw] OR "2-(3-methoxyphenyl)-2-(ethylamino)cyclohexanone"[Supplementary Concept] OR "2-Oxo-PCE"[Supplementary Concept] OR "Alfentanil"[mesh] OR "Chloralose"[mesh] OR "Diazepam"[mesh] OR "Etomidate"[mesh] OR "Fentanyl"[mesh] OR "Ketamine"[mesh] OR "Methohexital"[mesh] OR "Midazolam"[mesh] OR "Propanidid"[mesh] OR "Propofol"[mesh] OR "Sodium Oxybate"[mesh] OR "Sufentanil"[mesh] OR "Thiamylal"[mesh] OR "Thiopental"[mesh] OR "Tiletamine"[mesh] OR "Urethane"[mesh] OR "2-(3-methoxyphenyl)-2-(ethylamino)cyclohexanone"[tw] OR "2-Oxo-PCE"[tw] OR "Alfentanil"[tw] OR "Chloralose"[tw] OR "Diazepam"[tw] OR "Etomidate"[tw] OR "Fentanyl"[tw] OR "intravenous anaesthesia"[tw] OR "intravenous anaesthesias"[tw] OR "Intravenous anaesthetic"[tw] OR "Intravenous anaesthetic agent"[tw] OR "Intravenous anaesthetic agents"[tw] OR "Intravenous anaesthetic drug"[tw] OR "Intravenous anaesthetic drugs"[tw] OR "Intravenous anaesthetics"[tw] OR "intravenous anesthesia"[tw] OR "intravenous anesthesias"[tw] OR "intravenous anesthetic"[tw] OR "Intravenous anesthetic agents"[tw] OR "Intravenous anesthetic agents"[tw] OR "Intravenous anesthetic drug"[tw] OR "Intravenous anesthetic drugs"[tw] OR "intravenous anesthetics"[tw] OR "IV anaesthesia"[tw] OR "IV anaesthetics"[tw] OR "IV anaesthetics"[tw] OR "IV anesthesia"[tw] OR "IV anesthetic"[tw] OR "IV anesthetics"[tw] OR "Ketamine"[tw] OR "Methohexital"[tw] OR "Midazolam"[tw] OR "Propanidid"[tw] OR "Propofol"[tw] OR "Sodium Oxybate"[tw] OR "Sufentanil"[tw] OR "Thiamylal"[tw] OR "Thiopental"[tw] OR "Tiletamine"[tw] OR "TIVA"[tw] OR "Total intravenous anaesthesia"[tw] OR "Total Intravenous anesthesia"[tw] OR "Urethane"[tw]) AND ("Acidification potential"[tw] OR "acidification"[tw] OR "air pollution control"[tw] OR "AP in kg SO2 equivalents"[tw] OR "Biodiversity"[Mesh] OR "Biodiversity"[tw] OR "Carbon Footprint"[mesh] OR "carbon footprint"[tw] OR "carbon footprint*"[tw] OR "CFC-11 equi"[tw] OR "Climate Change"[Mesh] OR "climate change"[tw] OR "Climatic change"[tw] OR "CO2 emission"[tw] OR "CO2 emissions"[tw] OR "CO2 equiva*"[tw] OR "CO2 footprint"[tw] OR "CO2 footprint*"[tw] OR "conservation of natural resources"[mesh] OR "conservation of natural resources"[tw] OR "Disposable Equipment"[Mesh] OR "Disposable"[tw] OR "Disposables"[tw] OR "eco toxic*"[tw] OR "eco toxicity"[tw] OR "ecoefficien*"[tw] OR "eco-efficien*"[tw] OR "ecoefficiency"[tw] OR "eco-efficiency"[tw] OR "ecological footprint"[tw] OR "ecological footprint*"[tw] OR "ecological sustainability"[tw] OR "ecotoxic*"[tw] OR "ecotoxicity"[tw] OR "Emission reduction strategy"[tw] OR "Emission reduction"[tw] OR "Environment"[Mesh:noexp] OR "environmental impact"[tw] OR "environmental impact*"[tw] OR "environmental impacts"[tw] OR "environmental pollut*"[tw] OR "Environmental Pollution"[Mesh] OR "environmental pollution"[tw] OR "environmental protection"[tw] OR "environmental sustainab*"[tw] OR "environmental sustainability"[tw] OR "Environmental*"[ti] OR "EP in kg PO4 equivalent"[tw] OR "Equipment reuse"[mesh] OR "Equipment reuse"[tw] OR "Eutrophication potential"[tw] OR "Eutrophication"[Mesh] OR "eutrophication"[tw] OR "FAETP in kg DCB equivalent"[tw] OR "Freshwater Aquatic Ecotoxicity Potential"[tw] OR "Global Warming"[mesh] OR "Global Warming"[tw] OR "Green deal"[tw] OR "Green surgery"[tw] OR "Greenhouse Effect"[mesh] OR "greenhouse effect*"[tw] OR "greenhouse effects"[tw] OR "greenhouse gas emission"[tw] OR "greenhouse gas emissions"[tw] OR "Greenhouse Gas"[tw] OR "Greenhouse Gases"[mesh] OR "Greenhouse Gases"[tw] OR "greening"[tw] OR "GWP in kg CO2 equivalents"[tw] OR "H+ moles equivalents"[tw] OR "hospital waste"[tw] OR "HTTP in kg Dichlorobenzene equivalent"[tw] OR "Human Toxicity Potential"[tw] OR "kg 2.4-D equivalents"[tw] OR "kg CFC-11 equivalent"[tw] OR "kg N equivalents"[tw] OR "kg NOx equivalents"[tw] OR "LCA"[tw] OR "LCAs"[tw] OR "life cycle analysis"[tw] OR "life cycle assess*"[tw] OR "life cycle assessment"[tw] OR "life cycle inventories"[tw] OR "life cycle inventory"[tw] OR "Medical Waste Disposal"[mesh] OR "Medical Waste"[mesh] OR "medical waste"[tw] OR "N equiv*"[tw] OR "Ozone Depletion"[Mesh] OR "ozone depletion"[tw] OR "Photochemical Ozone Depletion Potential"[tw] OR "Plastic overuse"[tw] OR "POCP in kg ethane equivalent"[tw] OR "preservation of natural resources"[tw] OR "recycle*"[tw] OR "Recycling"[mesh] OR "recycling"[tw] OR "Refuse Disposal"[Mesh] OR "Refuse Disposal"[tw] OR "reusable"[tw] OR "Reusables"[tw] OR "reuse"[tw] OR "reused"[tw] OR "reusing"[tw] OR "Rising Sea Level"[tw] OR "Rising Sea Levels"[tw] OR "Sea Level Rise"[mesh] OR "Sea Level Rise"[tw] OR "Smog"[mesh] OR "smog"[tw] OR "SO2 equiv*"[tw] OR "sustainability"[ti] OR "Sustainable Development"[Mesh] OR "Sustainable Development"[tw] OR "Waste Disposal"[tw] OR "Waste Disposal, Fluid"[mesh] OR "Waste Management"[mesh] OR "Waste"[tw] OR "waste"[tw] OR "Waste Water"[Mesh] OR "Waste Water"[tw] OR "wastes"[tw] OR "Wastewater"[tw] OR "Water Purification"[Mesh] OR "Water Purification"[tw] OR (("plastic*"[tw] OR "microplastic*") AND ("soup"[tw] OR "pollution"[tw] OR "overuse"[tw] OR "contamination"[tw])) OR (("Plastic"[tw] OR "plastics"[tw]) AND "overuse"[tw]) OR ("hydrogen*"[tw] AND "moles"[tw] AND "equiv*"[tw]) OR ("Dichlorobenzen*"[tw] AND "equiv*"[tw]) OR ("2,4-D"[tw] AND "equiv*"[tw]) OR ("NOx"[tw] AND "equiv*"[tw]) OR ("ethane"[tw] AND "equiv*"[tw]) OR ("PO4"[tw] AND "equiv*"[tw]) OR ("DCB"[tw] AND "equiv*"[tw]) OR ("sustainability"[tw] AND ("environment*"[tw] OR "carbon"[tw])) OR (("Carbon Dioxide"[mesh] OR "Carbon Dioxide"[tw] OR "CO2"[tw]) AND ("pollution"[tw] OR "emission"[tw] OR "emissions"[tw] OR "waste"[tw] OR "environment"[tw] OR "environmental*"[tw] OR "footprint"[tw] OR "footprint*"[tw] OR "sustainable"[tw] OR "hazard"[tw] OR "hazard*"[tw]))) AND ("2000/01/01"[PDAT] : "3000/12/31"[PDAT])) AND (*("Meta-Analysis"[Publication Type] OR "Meta-Analysis as Topic"[Mesh] OR metaanaly*[tiab] OR meta-analy*[tiab] or metanaly*[tiab] OR "Systematic Review"[Publication Type] OR systematic[sb] OR "Cochrane Database Syst Rev"[Journal] or prisma[tiab] OR preferred reporting items[tiab] OR prospero[tiab] OR ((systemati*[ti] OR scoping[ti] OR umbrella[ti] OR structured literature[ti]) AND (review*[ti] OR overview*[ti])) OR systematic review*[tiab] OR scoping review*[tiab] OR umbrella review*[tiab] OR structured literature review*[tiab] OR systematic qualitative review*[tiab] OR systematic quantitative review*[tiab] OR systematic search and review[tiab] OR systematized review[tiab] OR systematised review[tiab] OR systemic review[tiab] OR systematic literature review*[tiab] OR systematic integrative literature review*[tiab] OR systematically review*[tiab] OR scoping literature review*[tiab] OR systematic critical review[tiab] OR systematic integrative review*[tiab] OR systematic evidence review[tiab] OR Systematic integrative literature review*[tiab] OR Systematic mixed studies review*[tiab] OR Systematized literature review*[tiab] OR Systematic overview*[tiab] OR Systematic narrative review*[tiab] OR ((systemati*[tiab] OR literature[tiab] OR database*[tiab] OR data-base*[tiab] OR structured[tiab] OR comprehensive*[tiab] OR systemic*[tiab]) AND search*[tiab]) OR (Literature[ti] AND review[ti] AND (database*[tiab] OR data-base*[tiab] OR search*[tiab])) OR ((data extraction[tiab] OR data source*[tiab]) AND study selection[tiab]) OR (search strategy[tiab] AND selection criteria[tiab]) OR (data source*[tiab] AND data synthesis[tiab]) OR medline[tiab] OR pubmed[tiab] OR embase[tiab] OR Cochrane[tiab] OR ((critical[ti] OR rapid[ti]) AND (review*[ti] OR overview*[ti] OR synthes*[ti])) OR (((critical*[tiab] OR rapid*[tiab]) AND (review*[tiab] OR overview*[tiab] OR synthes*[tiab]) AND (search*[tiab] OR database*[tiab] OR data-base*[tiab]))) OR metasynthes*[tiab] OR meta-synthes*[tiab])* OR ("Randomized Controlled Trial"[Publication Type] OR random*[tiab] OR pragmatic clinical trial*[tiab] OR practical clinical trial*[tiab] OR non-inferiority trial*[tiab] OR noninferiority trial*[tiab] OR superiority trial*[tiab] OR equivalence clinical trial*[tiab]) NOT (("Animals"[Mesh]) OR "Models, Animal"[Mesh] NOT humans[mh]) NOT (letter[pt] OR comment[pt] OR editorial[pt]) OR ("Comparative Study"[Publication Type] OR "comparison"[tiab] OR "comparative"[tiab] OR "compar*"[tiab] OR "Epidemiologic studies"[mesh:noexp] OR "case control studies"[mesh] OR "cohort studies"[mesh] OR "Controlled Before-After Studies"[mesh] OR "Case control"[tw] OR cohort*[tw] OR "Cohort analy*"[tw] OR "Follow up stud*"[tw] OR "observational stud*"[tw] OR Longitudinal[tw] OR Retrospective*[tw] OR prospective*[tw] OR consecutive*[tw] OR Cross sectional[tw] OR "Cross-sectional studies"[mesh] OR "historically controlled study"[mesh] OR "interrupted time series analysis"[mesh]) OR ("life cycle assess*"[tw] OR "life cycle assessment"[tw] OR "life cycle inventory"[tw] OR "LCA"[tw] OR "LCAs"[tw] OR "life cycle inventory"[tw] OR "life cycle inventories"[tw]))) OR ((("Anesthetics"[majr] OR "Anesthetic"[ti] OR "Anesthetics"[ti] OR "Anaesthetic"[ti] OR "Anaesthetics"[ti] OR "Anesthesia"[ti] OR "Anaesthesia"[ti] OR "Anesthesiology"[ti] OR "Anaesthesiology"[ti] OR "Anesthetics, Inhalation"[majr] OR "Anesthesia, Inhalation"[majr] OR "Closed-Circuit Anesthesia"[ti] OR "Endotracheal Anesthesia"[ti] OR "Closed-Circuit Anaesthesia"[ti] OR "Endotracheal Anaesthesia"[ti] OR "Halothane"[majr] OR "Isoflurane"[majr] OR "Methoxyflurane"[majr] OR "Sevoflurane"[majr] OR "Anaesthetic gas"[ti] OR "Anaesthetic gases"[ti] OR "Anesthetic gas"[ti] OR "Anesthetic gases"[ti] OR "Desflurane"[ti] OR "Enflurane"[ti] OR "Flurane"[ti] OR "Fluranes"[ti] OR "Gaseous anaesthesia"[ti] OR "Gaseous anaesthetic"[ti] OR "Gaseous anaesthetics"[ti] OR "Gaseous anesthesia"[ti] OR "Gaseous anesthetic"[ti] OR "Gaseous anesthetics"[ti] OR "Halothane"[ti] OR "Inhalation anaesthesia"[ti] OR "inhalation anaesthetic"[ti] OR "inhalation anaesthetics"[ti] OR "Inhalation anesthesia"[ti] OR "inhalation anesthetic"[ti] OR "inhalation anesthetics"[ti] OR "Inhalational agents"[ti] OR "Inhalational anaesthesia"[ti] OR "Inhalational anaesthetic"[ti] OR "Inhalational anaesthetic agent"[ti] OR "Inhalational anaesthetic agents"[ti] OR "Inhalational anaesthetics"[ti] OR "Inhalational anesthesia"[ti] OR "Inhalational anesthetic"[ti] OR "Inhalational anesthetic agent"[ti] OR "Inhalational anesthetic agents"[ti] OR "Inhalational anesthetics"[ti] OR "Inhaled anaesthetic"[ti] OR "Inhaled anaesthetic agent"[ti] OR "Inhaled anaesthetic agents"[ti] OR "Inhaled anaesthetics"[ti] OR "Inhaled anesthetic"[ti] OR "Inhaled anesthetic agents"[ti] OR "Inhaled anesthetics"[ti] OR "insufflation anaesthesia"[ti] OR "insufflation anaesthetic"[ti] OR "insufflation anaesthetics"[ti] OR "insufflation anesthesia"[ti] OR "insufflation anesthetic"[ti] OR "insufflation anesthetics"[ti] OR "Isoflurane"[ti] OR "Methoxyflurane"[ti] OR "Sevoflurane"[ti] OR "Vapor anaesthesia"[ti] OR "Vapor anaesthetic"[ti] OR "Vapor anaesthetics"[ti] OR "Vapor anesthesia"[ti] OR "Vapor anesthetic"[ti] OR "Vapor anesthetics"[ti] OR "Volatile agent"[ti] OR "Volatile agents"[ti] OR "Volatile Anaesthetic"[ti] OR "Volatile Anaesthetics"[ti] OR "Volatile anesthetic"[ti] OR "Volatile anesthetics"[ti] OR "Volatile drug"[ti] OR "Volatile drugs"[ti] OR "Volatile fluorinated liquid"[ti] OR "Volatile fluorinated liquids"[ti] OR "Volatile gas"[ti] OR "Volatile gases"[ti] OR "Volatile liquid agent"[ti] OR "Volatile liquid agents"[ti] OR "Anesthetic Gas"[ti] OR "Anesthetic Gases"[ti] OR "Inhalation Anaesthetic"[ti] OR "Inhalation Anaesthetics"[ti] OR "Inhalation Anesthetic"[ti] OR "Inhalation Anesthetics"[ti] OR "Anesthesia, Intravenous"[majr] OR "Anesthetics, Intravenous"[majr] OR "Intravenous Anesthetics"[ti] OR "Intravenous Anaesthetics"[ti] OR "Intravenous Anesthetic"[ti] OR "Intravenous Anaesthetic"[ti] OR "Intravenous Anesthesia"[ti] OR "Intravenous Anaesthesia"[ti] OR "Dissociative Anesthetics"[ti] OR "Dissociative Anaesthetics"[ti] OR "Dissociative Anesthetic"[ti] OR "Dissociative Anaesthetic"[ti] OR "Dissociative Anesthesia"[ti] OR "Dissociative Anaesthesia"[ti] OR "2-(3-methoxyphenyl)-2-(ethylamino)cyclohexanone"[Supplementary Concept] OR "2-Oxo-PCE"[Supplementary Concept] OR "Alfentanil"[majr] OR "Chloralose"[majr] OR "Diazepam"[majr] OR "Etomidate"[majr] OR "Fentanyl"[majr] OR "Ketamine"[majr] OR "Methohexital"[majr] OR "Midazolam"[majr] OR "Propanidid"[majr] OR "Propofol"[majr] OR "Sodium Oxybate"[majr] OR "Sufentanil"[majr] OR "Thiamylal"[majr] OR "Thiopental"[majr] OR "Tiletamine"[majr] OR "Urethane"[majr] OR "2-(3-methoxyphenyl)-2-(ethylamino)cyclohexanone"[ti] OR "2-Oxo-PCE"[ti] OR "Alfentanil"[ti] OR "Chloralose"[ti] OR "Diazepam"[ti] OR "Etomidate"[ti] OR "Fentanyl"[ti] OR "intravenous anaesthesia"[ti] OR "intravenous anaesthesias"[ti] OR "Intravenous anaesthetic"[ti] OR "Intravenous anaesthetic agent"[ti] OR "Intravenous anaesthetic agents"[ti] OR "Intravenous anaesthetic drug"[ti] OR "Intravenous anaesthetic drugs"[ti] OR "Intravenous anaesthetics"[ti] OR "intravenous anesthesia"[ti] OR "intravenous anesthesias"[ti] OR "intravenous anesthetic"[ti] OR "Intravenous anesthetic agents"[ti] OR "Intravenous anesthetic agents"[ti] OR "Intravenous anesthetic drug"[ti] OR "Intravenous anesthetic drugs"[ti] OR "intravenous anesthetics"[ti] OR "IV anaesthesia"[ti] OR "IV anaesthetics"[ti] OR "IV anaesthetics"[ti] OR "IV anesthesia"[ti] OR "IV anesthetic"[ti] OR "IV anesthetics"[ti] OR "Ketamine"[ti] OR "Methohexital"[ti] OR "Midazolam"[ti] OR "Propanidid"[ti] OR "Propofol"[ti] OR "Sodium Oxybate"[ti] OR "Sufentanil"[ti] OR "Thiamylal"[ti] OR "Thiopental"[ti] OR "Tiletamine"[ti] OR "TIVA"[ti] OR "Total intravenous anaesthesia"[ti] OR "Total Intravenous anesthesia"[ti] OR "Urethane"[ti]) AND ("Acidification potential"[ti] OR "acidification"[ti] OR "air pollution control"[ti] OR "AP in kg SO2 equivalents"[ti] OR "Biodiversity"[majr] OR "Biodiversity"[ti] OR "Carbon Footprint"[majr] OR "carbon footprint"[ti] OR "carbon footprint*"[ti] OR "CFC-11 equi"[ti] OR "Climate Change"[majr] OR "climate change"[ti] OR "Climatic change"[ti] OR "CO2 emission"[ti] OR "CO2 emissions"[ti] OR "CO2 equiva*"[ti] OR "CO2 footprint"[ti] OR "CO2 footprint*"[ti] OR "conservation of natural resources"[majr] OR "conservation of natural resources"[ti] OR "Disposable Equipment"[majr] OR "Disposable"[ti] OR "Disposables"[ti] OR "eco toxic*"[ti] OR "eco toxicity"[ti] OR "ecoefficien*"[ti] OR "eco-efficien*"[ti] OR "ecoefficiency"[ti] OR "eco-efficiency"[ti] OR "ecological footprint"[ti] OR "ecological footprint*"[ti] OR "ecological sustainability"[ti] OR "ecotoxic*"[ti] OR "ecotoxicity"[ti] OR "Emission reduction strategy"[ti] OR "Emission reduction"[ti] OR "Environment"[majr:noexp] OR "environmental impact"[ti] OR "environmental impact*"[ti] OR "environmental impacts"[ti] OR "environmental pollut*"[ti] OR "Environmental Pollution"[majr] OR "environmental pollution"[ti] OR "environmental protection"[ti] OR "environmental sustainab*"[ti] OR "environmental sustainability"[ti] OR "Environmental*"[ti] OR "EP in kg PO4 equivalent"[ti] OR "Equipment reuse"[majr] OR "Equipment reuse"[ti] OR "Eutrophication potential"[ti] OR "Eutrophication"[majr] OR "eutrophication"[ti] OR "FAETP in kg DCB equivalent"[ti] OR "Freshwater Aquatic Ecotoxicity Potential"[ti] OR "Global Warming"[majr] OR "Global Warming"[ti] OR "Green deal"[ti] OR "Green surgery"[ti] OR "Greenhouse Effect"[majr] OR "greenhouse effect*"[ti] OR "greenhouse effects"[ti] OR "greenhouse gas emission"[ti] OR "greenhouse gas emissions"[ti] OR "Greenhouse Gas"[ti] OR "Greenhouse Gases"[majr] OR "Greenhouse Gases"[ti] OR "greening"[ti] OR "GWP in kg CO2 equivalents"[ti] OR "H+ moles equivalents"[ti] OR "hospital waste"[ti] OR "HTTP in kg Dichlorobenzene equivalent"[ti] OR "Human Toxicity Potential"[ti] OR "kg 2.4-D equivalents"[ti] OR "kg CFC-11 equivalent"[ti] OR "kg N equivalents"[ti] OR "kg NOx equivalents"[ti] OR "LCA"[ti] OR "LCAs"[ti] OR "life cycle analysis"[ti] OR "life cycle assess*"[ti] OR "life cycle assessment"[ti] OR "life cycle inventories"[ti] OR "life cycle inventory"[ti] OR "Medical Waste Disposal"[majr] OR "Medical Waste"[majr] OR "medical waste"[ti] OR "N equiv*"[ti] OR "Ozone Depletion"[majr] OR "ozone depletion"[ti] OR "Photochemical Ozone Depletion Potential"[ti] OR "Plastic overuse"[ti] OR "POCP in kg ethane equivalent"[ti] OR "preservation of natural resources"[ti] OR "recycle*"[ti] OR "Recycling"[majr] OR "recycling"[ti] OR "Refuse Disposal"[majr] OR "Refuse Disposal"[ti] OR "reusable"[ti] OR "Reusables"[ti] OR "reuse"[ti] OR "reused"[ti] OR "reusing"[ti] OR "Rising Sea Level"[ti] OR "Rising Sea Levels"[ti] OR "Sea Level Rise"[majr] OR "Sea Level Rise"[ti] OR "Smog"[majr] OR "smog"[ti] OR "SO2 equiv*"[ti] OR "sustainability"[ti] OR "Sustainable Development"[majr] OR "Sustainable Development"[ti] OR "Waste Disposal"[ti] OR "Waste Disposal, Fluid"[majr] OR "Waste Management"[majr] OR "Waste"[ti] OR "waste"[ti] OR "Waste Water"[majr] OR "Waste Water"[ti] OR "wastes"[ti] OR "Wastewater"[ti] OR "Water Purification"[majr] OR "Water Purification"[ti] OR (("plastic*"[ti] OR "microplastic*") AND ("soup"[ti] OR "pollution"[ti] OR "overuse"[ti] OR "contamination"[ti])) OR (("Plastic"[ti] OR "plastics"[ti]) AND "overuse"[ti]) OR ("hydrogen*"[ti] AND "moles"[ti] AND "equiv*"[ti]) OR ("Dichlorobenzen*"[ti] AND "equiv*"[ti]) OR ("2,4-D"[ti] AND "equiv*"[ti]) OR ("NOx"[ti] AND "equiv*"[ti]) OR ("ethane"[ti] AND "equiv*"[ti]) OR ("PO4"[ti] AND "equiv*"[ti]) OR ("DCB"[ti] AND "equiv*"[ti]) OR ("sustainability"[ti] AND ("environment*"[ti] OR "carbon"[ti])) OR (("Carbon Dioxide"[majr] OR "Carbon Dioxide"[ti] OR "CO2"[ti]) AND ("pollution"[ti] OR "emission"[ti] OR "emissions"[ti] OR "waste"[ti] OR "environment"[ti] OR "environmental*"[ti] OR "footprint"[ti] OR "footprint*"[ti] OR "sustainable"[ti] OR "hazard"[ti] OR "hazard*"[ti]))) AND ("2000/01/01"[PDAT] : "3000/12/31"[PDAT])) AND (*("Meta-Analysis"[Publication Type] OR "Meta-Analysis as Topic"[Mesh] OR metaanaly*[tiab] OR meta-analy*[tiab] or metanaly*[tiab] OR "Systematic Review"[Publication Type] OR systematic[sb] OR "Cochrane Database Syst Rev"[Journal] or prisma[tiab] OR preferred reporting items[tiab] OR prospero[tiab] OR ((systemati*[ti] OR scoping[ti] OR umbrella[ti] OR structured literature[ti]) AND (review*[ti] OR overview*[ti])) OR systematic review*[tiab] OR scoping review*[tiab] OR umbrella review*[tiab] OR structured literature review*[tiab] OR systematic qualitative review*[tiab] OR systematic quantitative review*[tiab] OR systematic search and review[tiab] OR systematized review[tiab] OR systematised review[tiab] OR systemic review[tiab] OR systematic literature review*[tiab] OR systematic integrative literature review*[tiab] OR systematically review*[tiab] OR scoping literature review*[tiab] OR systematic critical review[tiab] OR systematic integrative review*[tiab] OR systematic evidence review[tiab] OR Systematic integrative literature review*[tiab] OR Systematic mixed studies review*[tiab] OR Systematized literature review*[tiab] OR Systematic overview*[tiab] OR Systematic narrative review*[tiab] OR ((systemati*[tiab] OR literature[tiab] OR database*[tiab] OR data-base*[tiab] OR structured[tiab] OR comprehensive*[tiab] OR systemic*[tiab]) AND search*[tiab]) OR (Literature[ti] AND review[ti] AND (database*[tiab] OR data-base*[tiab] OR search*[tiab])) OR ((data extraction[tiab] OR data source*[tiab]) AND study selection[tiab]) OR (search strategy[tiab] AND selection criteria[tiab]) OR (data source*[tiab] AND data synthesis[tiab]) OR medline[tiab] OR pubmed[tiab] OR embase[tiab] OR Cochrane[tiab] OR ((critical[ti] OR rapid[ti]) AND (review*[ti] OR overview*[ti] OR synthes*[ti])) OR (((critical*[tiab] OR rapid*[tiab]) AND (review*[tiab] OR overview*[tiab] OR synthes*[tiab]) AND (search*[tiab] OR database*[tiab] OR data-base*[tiab]))) OR metasynthes*[tiab] OR meta-synthes*[tiab])* OR ("Randomized Controlled Trial"[Publication Type] OR random*[tiab] OR pragmatic clinical trial*[tiab] OR practical clinical trial*[tiab] OR non-inferiority trial*[tiab] OR noninferiority trial*[tiab] OR superiority trial*[tiab] OR equivalence clinical trial*[tiab]) NOT (("Animals"[Mesh]) OR "Models, Animal"[Mesh] NOT humans[mh]) NOT (letter[pt] OR comment[pt] OR editorial[pt]) OR ("Comparative Study"[Publication Type] OR "comparison"[tiab] OR "comparative"[tiab] OR "compar*"[tiab] OR "Epidemiologic studies"[mesh:noexp] OR "case control studies"[mesh] OR "cohort studies"[mesh] OR "Controlled Before-After Studies"[mesh] OR "Case control"[tw] OR cohort*[tw] OR "Cohort analy*"[tw] OR "Follow up stud*"[tw] OR "observational stud*"[tw] OR Longitudinal[tw] OR Retrospective*[tw] OR prospective*[tw] OR consecutive*[tw] OR Cross sectional[tw] OR "Cross-sectional studies"[mesh] OR "historically controlled study"[mesh] OR "interrupted time series analysis"[mesh]) OR ("life cycle assess*"[tw] OR "life cycle assessment"[tw] OR "life cycle inventory"[tw] OR "LCA"[tw] OR "LCAs"[tw] OR "life cycle inventory"[tw] OR "life cycle inventories"[tw]))))

**UV4.2**

(("Anesthesia, Inhalation"[Mesh] OR "Anesthetics, Inhalation"[Mesh] OR "Anesthetics, Inhalation"[pharmacological action] OR "Closed-Circuit Anesthesia"[tw] OR "Endotracheal Anesthesia"[tw] OR "Closed-Circuit Anaesthesia"[tw] OR "Endotracheal Anaesthesia"[tw] OR "Halothane"[mesh] OR "Isoflurane"[mesh] OR "Methoxyflurane"[mesh] OR "Sevoflurane"[mesh] OR "Anaesthetic gas"[tw] OR "Anaesthetic gases"[tw] OR "Anesthetic gas"[tw] OR "Anesthetic gases"[tw] OR "Desflurane"[tw] OR "Enflurane"[tw] OR "Flurane"[tw] OR "Fluranes"[tw] OR "Gaseous anaesthesia"[tw] OR "Gaseous anaesthetic"[tw] OR "Gaseous anaesthetics"[tw] OR "Gaseous anesthesia"[tw] OR "Gaseous anesthetic"[tw] OR "Gaseous anesthetics"[tw] OR "Halothane"[tw] OR "Inhalation anaesthesia"[tw] OR "inhalation anaesthetic"[tw] OR "inhalation anaesthetics"[tw] OR "Inhalation anesthesia"[tw] OR "inhalation anesthetic"[tw] OR "inhalation anesthetics"[tw] OR "Inhalational agents"[tw] OR "Inhalational anaesthesia"[tw] OR "Inhalational anaesthetic"[tw] OR "Inhalational anaesthetic agent"[tw] OR "Inhalational anaesthetic agents"[tw] OR "Inhalational anaesthetics"[tw] OR "Inhalational anesthesia"[tw] OR "Inhalational anesthetic"[tw] OR "Inhalational anesthetic agent"[tw] OR "Inhalational anesthetic agents"[tw] OR "Inhalational anesthetics"[tw] OR "Inhaled anaesthetic"[tw] OR "Inhaled anaesthetic agent"[tw] OR "Inhaled anaesthetic agents"[tw] OR "Inhaled anaesthetics"[tw] OR "Inhaled anesthetic"[tw] OR "Inhaled anesthetic agents"[tw] OR "Inhaled anesthetics"[tw] OR "insufflation anaesthesia"[tw] OR "insufflation anaesthetic"[tw] OR "insufflation anaesthetics"[tw] OR "insufflation anesthesia"[tw] OR "insufflation anesthetic"[tw] OR "insufflation anesthetics"[tw] OR "Isoflurane"[tw] OR "Methoxyflurane"[tw] OR "Sevoflurane"[tw] OR "Vapor anaesthesia"[tw] OR "Vapor anaesthetic"[tw] OR "Vapor anaesthetics"[tw] OR "Vapor anesthesia"[tw] OR "Vapor anesthetic"[tw] OR "Vapor anesthetics"[tw] OR "Volatile agent"[tw] OR "Volatile agents"[tw] OR "Volatile Anaesthetic"[tw] OR "Volatile Anaesthetics"[tw] OR "Volatile anesthetic"[tw] OR "Volatile anesthetics"[tw] OR "Volatile drug"[tw] OR "Volatile drugs"[tw] OR "Volatile fluorinated liquid"[tw] OR "Volatile fluorinated liquids"[tw] OR "Volatile gas"[tw] OR "Volatile gases"[tw] OR "Volatile liquid agent"[tw] OR "Volatile liquid agents"[tw] OR "Anesthetic Gas"[tw] OR "Anesthetic Gases"[tw] OR "Inhalation Anaesthetic"[tw] OR "Inhalation Anaesthetics"[tw] OR "Inhalation Anesthetic"[tw] OR "Inhalation Anesthetics"[tw]) AND ("anaesthetic gas scavenging system"[tw] OR "anesthetic gas scavenging system"[tw] OR "Contrafluran"[tw] OR "gas extract*"[tw] OR "gas extraction"[tw] OR "gas extraction system"[tw] OR "gas extraction systems"[tw] OR "gas scaveng*"[tw] OR "gas scavenger"[tw] OR "gas scavengers"[tw] OR "gas scavenging "[tw] OR "scavenging device"[tw] OR "scavenging device"[tw] OR "scavenging devices"[tw] OR "scavenging system"[tw] OR "scavenging systems"[tw] OR "vapour captur*"[tw] OR "vapour capture"[tw] OR "Vapour Capture Technology"[tw] OR "vapour recycl*"[tw] OR "vapour recycling technology"[tw] OR "vapour recycling"[tw]) AND ("Acidification potential"[tw] OR "acidification"[tw] OR "air pollution control"[tw] OR "AP in kg SO2 equivalents"[tw] OR "Biodiversity"[Mesh] OR "Biodiversity"[tw] OR "Carbon Footprint"[mesh] OR "carbon footprint"[tw] OR "carbon footprint*"[tw] OR "CFC-11 equi"[tw] OR "Climate Change"[Mesh] OR "climate change"[tw] OR "Climatic change"[tw] OR "CO2 emission"[tw] OR "CO2 emissions"[tw] OR "CO2 equiva*"[tw] OR "CO2 footprint"[tw] OR "CO2 footprint*"[tw] OR "conservation of natural resources"[mesh] OR "conservation of natural resources"[tw] OR "Disposable Equipment"[Mesh] OR "Disposable"[tw] OR "Disposables"[tw] OR "eco toxic*"[tw] OR "eco toxicity"[tw] OR "ecoefficien*"[tw] OR "eco-efficien*"[tw] OR "ecoefficiency"[tw] OR "eco-efficiency"[tw] OR "ecological footprint"[tw] OR "ecological footprint*"[tw] OR "ecological sustainability"[tw] OR "ecotoxic*"[tw] OR "ecotoxicity"[tw] OR "Emission reduction strategy"[tw] OR "Emission reduction"[tw] OR "Environment"[Mesh:noexp] OR "environmental impact"[tw] OR "environmental impact*"[tw] OR "environmental impacts"[tw] OR "environmental pollut*"[tw] OR "Environmental Pollution"[Mesh] OR "environmental pollution"[tw] OR "environmental protection"[tw] OR "environmental sustainab*"[tw] OR "environmental sustainability"[tw] OR "Environmental*"[ti] OR "EP in kg PO4 equivalent"[tw] OR "Equipment reuse"[mesh] OR "Equipment reuse"[tw] OR "Eutrophication potential"[tw] OR "Eutrophication"[Mesh] OR "eutrophication"[tw] OR "FAETP in kg DCB equivalent"[tw] OR "Freshwater Aquatic Ecotoxicity Potential"[tw] OR "Global Warming"[mesh] OR "Global Warming"[tw] OR "Green deal"[tw] OR "Green surgery"[tw] OR "Greenhouse Effect"[mesh] OR "greenhouse effect*"[tw] OR "greenhouse effects"[tw] OR "greenhouse gas emission"[tw] OR "greenhouse gas emissions"[tw] OR "Greenhouse Gas"[tw] OR "Greenhouse Gases"[mesh] OR "Greenhouse Gases"[tw] OR "greening"[tw] OR "GWP in kg CO2 equivalents"[tw] OR "H+ moles equivalents"[tw] OR "hospital waste"[tw] OR "HTTP in kg Dichlorobenzene equivalent"[tw] OR "Human Toxicity Potential"[tw] OR "kg 2.4-D equivalents"[tw] OR "kg CFC-11 equivalent"[tw] OR "kg N equivalents"[tw] OR "kg NOx equivalents"[tw] OR "LCA"[tw] OR "LCAs"[tw] OR "life cycle analysis"[tw] OR "life cycle assess*"[tw] OR "life cycle assessment"[tw] OR "life cycle inventories"[tw] OR "life cycle inventory"[tw] OR "Medical Waste Disposal"[mesh] OR "Medical Waste"[mesh] OR "medical waste"[tw] OR "N equiv*"[tw] OR "Ozone Depletion"[Mesh] OR "ozone depletion"[tw] OR "Photochemical Ozone Depletion Potential"[tw] OR "Plastic overuse"[tw] OR "POCP in kg ethane equivalent"[tw] OR "preservation of natural resources"[tw] OR "recycle*"[tw] OR "Recycling"[mesh] OR "recycling"[tw] OR "Refuse Disposal"[Mesh] OR "Refuse Disposal"[tw] OR "reusable"[tw] OR "Reusables"[tw] OR "reuse"[tw] OR "reused"[tw] OR "reusing"[tw] OR "Rising Sea Level"[tw] OR "Rising Sea Levels"[tw] OR "Sea Level Rise"[mesh] OR "Sea Level Rise"[tw] OR "Smog"[mesh] OR "smog"[tw] OR "SO2 equiv*"[tw] OR "sustainability"[ti] OR "Sustainable Development"[Mesh] OR "Sustainable Development"[tw] OR "Waste Disposal"[tw] OR "Waste Disposal, Fluid"[mesh] OR "Waste Management"[mesh] OR "Waste"[tw] OR "waste"[tw] OR "Waste Water"[Mesh] OR "Waste Water"[tw] OR "wastes"[tw] OR "Wastewater"[tw] OR "Water Purification"[Mesh] OR "Water Purification"[tw] OR (("plastic*"[tw] OR "microplastic*") AND ("soup"[tw] OR "pollution"[tw] OR "overuse"[tw] OR "contamination"[tw])) OR (("Plastic"[tw] OR "plastics"[tw]) AND "overuse"[tw]) OR ("hydrogen*"[tw] AND "moles"[tw] AND "equiv*"[tw]) OR ("Dichlorobenzen*"[tw] AND "equiv*"[tw]) OR ("2,4-D"[tw] AND "equiv*"[tw]) OR ("NOx"[tw] AND "equiv*"[tw]) OR ("ethane"[tw] AND "equiv*"[tw]) OR ("PO4"[tw] AND "equiv*"[tw]) OR ("DCB"[tw] AND "equiv*"[tw]) OR ("sustainability"[tw] AND ("environment*"[tw] OR "carbon"[tw])) OR (("Carbon Dioxide"[mesh] OR "Carbon Dioxide"[tw] OR "CO2"[tw]) AND ("pollution"[tw] OR "emission"[tw] OR "emissions"[tw] OR "waste"[tw] OR "environment"[tw] OR "environmental*"[tw] OR "footprint"[tw] OR "footprint*"[tw] OR "sustainable"[tw] OR "hazard"[tw] OR "hazard*"[tw]))) AND ("2000/01/01"[PDAT] : "3000/12/31"[PDAT])) AND (*("Meta-Analysis"[Publication Type] OR "Meta-Analysis as Topic"[Mesh] OR metaanaly*[tiab] OR meta-analy*[tiab] or metanaly*[tiab] OR "Systematic Review"[Publication Type] OR systematic[sb] OR "Cochrane Database Syst Rev"[Journal] or prisma[tiab] OR preferred reporting items[tiab] OR prospero[tiab] OR ((systemati*[ti] OR scoping[ti] OR umbrella[ti] OR structured literature[ti]) AND (review*[ti] OR overview*[ti])) OR systematic review*[tiab] OR scoping review*[tiab] OR umbrella review*[tiab] OR structured literature review*[tiab] OR systematic qualitative review*[tiab] OR systematic quantitative review*[tiab] OR systematic search and review[tiab] OR systematized review[tiab] OR systematised review[tiab] OR systemic review[tiab] OR systematic literature review*[tiab] OR systematic integrative literature review*[tiab] OR systematically review*[tiab] OR scoping literature review*[tiab] OR systematic critical review[tiab] OR systematic integrative review*[tiab] OR systematic evidence review[tiab] OR Systematic integrative literature review*[tiab] OR Systematic mixed studies review*[tiab] OR Systematized literature review*[tiab] OR Systematic overview*[tiab] OR Systematic narrative review*[tiab] OR ((systemati*[tiab] OR literature[tiab] OR database*[tiab] OR data-base*[tiab] OR structured[tiab] OR comprehensive*[tiab] OR systemic*[tiab]) AND search*[tiab]) OR (Literature[ti] AND review[ti] AND (database*[tiab] OR data-base*[tiab] OR search*[tiab])) OR ((data extraction[tiab] OR data source*[tiab]) AND study selection[tiab]) OR (search strategy[tiab] AND selection criteria[tiab]) OR (data source*[tiab] AND data synthesis[tiab]) OR medline[tiab] OR pubmed[tiab] OR embase[tiab] OR Cochrane[tiab] OR ((critical[ti] OR rapid[ti]) AND (review*[ti] OR overview*[ti] OR synthes*[ti])) OR (((critical*[tiab] OR rapid*[tiab]) AND (review*[tiab] OR overview*[tiab] OR synthes*[tiab]) AND (search*[tiab] OR database*[tiab] OR data-base*[tiab]))) OR metasynthes*[tiab] OR meta-synthes*[tiab])* OR ("Randomized Controlled Trial"[Publication Type] OR random*[tiab] OR pragmatic clinical trial*[tiab] OR practical clinical trial*[tiab] OR non-inferiority trial*[tiab] OR noninferiority trial*[tiab] OR superiority trial*[tiab] OR equivalence clinical trial*[tiab]) NOT (("Animals"[Mesh]) OR "Models, Animal"[Mesh] NOT humans[mh]) NOT (letter[pt] OR comment[pt] OR editorial[pt]) OR ("Comparative Study"[Publication Type] OR "comparison"[tiab] OR "comparative"[tiab] OR "compar*"[tiab] OR "Epidemiologic studies"[mesh:noexp] OR "case control studies"[mesh] OR "cohort studies"[mesh] OR "Controlled Before-After Studies"[mesh] OR "Case control"[tw] OR cohort*[tw] OR "Cohort analy*"[tw] OR "Follow up stud*"[tw] OR "observational stud*"[tw] OR Longitudinal[tw] OR Retrospective*[tw] OR prospective*[tw] OR consecutive*[tw] OR Cross sectional[tw] OR "Cross-sectional studies"[mesh] OR "historically controlled study"[mesh] OR "interrupted time series analysis"[mesh]) OR ("life cycle assess*"[tw] OR "life cycle assessment"[tw] OR "life cycle inventory"[tw] OR "LCA"[tw] OR "LCAs"[tw] OR "life cycle inventory"[tw] OR "life cycle inventories"[tw]))

**UV4.3**

(("Anesthesia, Local"[Mesh] OR "Local Anesthesia"[tw] OR "Local Anaesthesia"[tw] OR "Locoregional Anesthesia"[tw] OR "Locoregional Anaesthesia"[tw] OR "Local Anesthetics"[tw] OR "Local Anaesthetics"[tw] OR "Locoregional Anesthetics"[tw] OR "Locoregional Anaesthetics"[tw] OR "Local Anesthetic"[tw] OR "Local Anaesthetic"[tw] OR "Locoregional Anesthetic"[tw] OR "Locoregional Anaesthetic"[tw] OR "axillary block"[tw] OR "axillary block*"[tw] OR "Bier block"[tw] OR "Bier block*"[tw] OR "Bier's block"[tw] OR "Bier's block*"[tw] OR "brachial plexus block"[tw] OR "brachial plexus block*"[tw] OR "combined spinal epidural"[tw] OR "combined spinal epidural block"[tw] OR "combined spinal epidural block*"[tw] OR "epidural anaesthesia"[tw] OR "epidural anesthesia"[tw] OR "intravenous regional anesthaesia"[tw] OR "intravenous regional anesthesia"[tw] OR "ischiadic block"[tw] OR "ischiadic block*"[tw] OR "local infiltration"[tw] OR "lower extremity block"[tw] OR "lower extremity block*"[tw] OR "nerve block"[tw] OR "nerve block"[tw] OR "nerve block*"[tw] OR "nerve block*"[tw] OR "neuraxial anaesthesia"[tw] OR "neuraxial anesthesia"[tw] OR "neuraxial block"[tw] OR "neuraxial block*"[tw] OR "neuraxial technique"[tw] OR "neuraxial techniques"[tw] OR "peripheral nerve block"[tw] OR "peripheral nerve block*"[tw] OR "plexus block"[tw] OR "plexus block*"[tw] OR "plexus nerve block"[tw] OR "plexus nerve block*"[tw] OR "popliteal block"[tw] OR "popliteal block*"[tw] OR "regional anaesthesia"[tw] OR "regional anesthesia"[tw] OR "spinal anaesthesia"[tw] OR "spinal anaesthetic block"[tw] OR "spinal anaesthetic block*"[tw] OR "spinal anesthesia"[tw] OR "spinal anesthetic block"[tw] OR "spinal anesthetic block*"[tw] OR "supraclavicular block"[tw] OR "supraclavicular block*"[tw] OR "upper extremity block"[tw] OR "upper extremity block*"[tw]) AND ("Anesthesia, General"[Mesh] OR "general anesthesia"[tw] OR "general anaesthesia"[tw] OR "general anesthe*"[tw] OR "general anaesthe*"[tw] OR "Anesthesia, Inhalation"[Mesh] OR "Closed-Circuit Anesthesia"[tw] OR "Endotracheal Anesthesia"[tw] OR "Closed-Circuit Anaesthesia"[tw] OR "Endotracheal Anaesthesia"[tw] OR "Halothane"[mesh] OR "Isoflurane"[mesh] OR "Methoxyflurane"[mesh] OR "Sevoflurane"[mesh] OR "Anaesthetic gas"[tw] OR "Anaesthetic gases"[tw] OR "Anesthetic gas"[tw] OR "Anesthetic gases"[tw] OR "Desflurane"[tw] OR "Enflurane"[tw] OR "Flurane"[tw] OR "Fluranes"[tw] OR "Gaseous anaesthesia"[tw] OR "Gaseous anaesthetic"[tw] OR "Gaseous anaesthetics"[tw] OR "Gaseous anesthesia"[tw] OR "Gaseous anesthetic"[tw] OR "Gaseous anesthetics"[tw] OR "Halothane"[tw] OR "Inhalation anaesthesia"[tw] OR "inhalation anaesthetic"[tw] OR "inhalation anaesthetics"[tw] OR "Inhalation anesthesia"[tw] OR "inhalation anesthetic"[tw] OR "inhalation anesthetics"[tw] OR "Inhalational agents"[tw] OR "Inhalational anaesthesia"[tw] OR "Inhalational anaesthetic"[tw] OR "Inhalational anaesthetic agent"[tw] OR "Inhalational anaesthetic agents"[tw] OR "Inhalational anaesthetics"[tw] OR "Inhalational anesthesia"[tw] OR "Inhalational anesthetic"[tw] OR "Inhalational anesthetic agent"[tw] OR "Inhalational anesthetic agents"[tw] OR "Inhalational anesthetics"[tw] OR "Inhaled anaesthetic"[tw] OR "Inhaled anaesthetic agent"[tw] OR "Inhaled anaesthetic agents"[tw] OR "Inhaled anaesthetics"[tw] OR "Inhaled anesthetic"[tw] OR "Inhaled anesthetic agents"[tw] OR "Inhaled anesthetics"[tw] OR "insufflation anaesthesia"[tw] OR "insufflation anaesthetic"[tw] OR "insufflation anaesthetics"[tw] OR "insufflation anesthesia"[tw] OR "insufflation anesthetic"[tw] OR "insufflation anesthetics"[tw] OR "Isoflurane"[tw] OR "Methoxyflurane"[tw] OR "Sevoflurane"[tw] OR "Vapor anaesthesia"[tw] OR "Vapor anaesthetic"[tw] OR "Vapor anaesthetics"[tw] OR "Vapor anesthesia"[tw] OR "Vapor anesthetic"[tw] OR "Vapor anesthetics"[tw] OR "Volatile agent"[tw] OR "Volatile agents"[tw] OR "Volatile Anaesthetic"[tw] OR "Volatile Anaesthetics"[tw] OR "Volatile anesthetic"[tw] OR "Volatile anesthetics"[tw] OR "Volatile drug"[tw] OR "Volatile drugs"[tw] OR "Volatile fluorinated liquid"[tw] OR "Volatile fluorinated liquids"[tw] OR "Volatile gas"[tw] OR "Volatile gases"[tw] OR "Volatile liquid agent"[tw] OR "Volatile liquid agents"[tw] OR "Anesthetics, Inhalation"[Mesh] OR "Anesthetic Gas"[tw] OR "Anesthetic Gases"[tw] OR "Inhalation Anaesthetic"[tw] OR "Inhalation Anaesthetics"[tw] OR "Inhalation Anesthetic"[tw] OR "Inhalation Anesthetics"[tw] OR "Anesthesia, Intravenous"[Mesh] OR "Anesthetics, Intravenous"[Mesh] OR "Anesthetics, Intravenous"[Pharmacological Action] OR "Intravenous Anesthetics"[tw] OR "Intravenous Anaesthetics"[tw] OR "Intravenous Anesthetic"[tw] OR "Intravenous Anaesthetic"[tw] OR "Intravenous Anesthesia"[tw] OR "Intravenous Anaesthesia"[tw] OR "Dissociative Anesthetics"[tw] OR "Dissociative Anaesthetics"[tw] OR "Dissociative Anesthetic"[tw] OR "Dissociative Anaesthetic"[tw] OR "Dissociative Anesthesia"[tw] OR "Dissociative Anaesthesia"[tw] OR "2-(3-methoxyphenyl)-2-(ethylamino)cyclohexanone"[Supplementary Concept] OR "2-Oxo-PCE"[Supplementary Concept] OR "Alfentanil"[mesh] OR "Chloralose"[mesh] OR "Diazepam"[mesh] OR "Etomidate"[mesh] OR "Fentanyl"[mesh] OR "Ketamine"[mesh] OR "Methohexital"[mesh] OR "Midazolam"[mesh] OR "Propanidid"[mesh] OR "Propofol"[mesh] OR "Sodium Oxybate"[mesh] OR "Sufentanil"[mesh] OR "Thiamylal"[mesh] OR "Thiopental"[mesh] OR "Tiletamine"[mesh] OR "Urethane"[mesh] OR "2-(3-methoxyphenyl)-2-(ethylamino)cyclohexanone"[tw] OR "2-Oxo-PCE"[tw] OR "Alfentanil"[tw] OR "Chloralose"[tw] OR "Diazepam"[tw] OR "Etomidate"[tw] OR "Fentanyl"[tw] OR "intravenous anaesthesia"[tw] OR "intravenous anaesthesias"[tw] OR "Intravenous anaesthetic"[tw] OR "Intravenous anaesthetic agent"[tw] OR "Intravenous anaesthetic agents"[tw] OR "Intravenous anaesthetic drug"[tw] OR "Intravenous anaesthetic drugs"[tw] OR "Intravenous anaesthetics"[tw] OR "intravenous anesthesia"[tw] OR "intravenous anesthesias"[tw] OR "intravenous anesthetic"[tw] OR "Intravenous anesthetic agents"[tw] OR "Intravenous anesthetic agents"[tw] OR "Intravenous anesthetic drug"[tw] OR "Intravenous anesthetic drugs"[tw] OR "intravenous anesthetics"[tw] OR "IV anaesthesia"[tw] OR "IV anaesthetics"[tw] OR "IV anaesthetics"[tw] OR "IV anesthesia"[tw] OR "IV anesthetic"[tw] OR "IV anesthetics"[tw] OR "Ketamine"[tw] OR "Methohexital"[tw] OR "Midazolam"[tw] OR "Propanidid"[tw] OR "Propofol"[tw] OR "Sodium Oxybate"[tw] OR "Sufentanil"[tw] OR "Thiamylal"[tw] OR "Thiopental"[tw] OR "Tiletamine"[tw] OR "TIVA"[tw] OR "Total intravenous anaesthesia"[tw] OR "Total Intravenous anesthesia"[tw] OR "Urethane"[tw]) AND ("Acidification potential"[tw] OR "acidification"[tw] OR "air pollution control"[tw] OR "AP in kg SO2 equivalents"[tw] OR "Biodiversity"[Mesh] OR "Biodiversity"[tw] OR "Carbon Footprint"[mesh] OR "carbon footprint"[tw] OR "carbon footprint*"[tw] OR "CFC-11 equi"[tw] OR "Climate Change"[Mesh] OR "climate change"[tw] OR "Climatic change"[tw] OR "CO2 emission"[tw] OR "CO2 emissions"[tw] OR "CO2 equiva*"[tw] OR "CO2 footprint"[tw] OR "CO2 footprint*"[tw] OR "conservation of natural resources"[mesh] OR "conservation of natural resources"[tw] OR "Disposable Equipment"[Mesh] OR "Disposable"[tw] OR "Disposables"[tw] OR "eco toxic*"[tw] OR "eco toxicity"[tw] OR "ecoefficien*"[tw] OR "eco-efficien*"[tw] OR "ecoefficiency"[tw] OR "eco-efficiency"[tw] OR "ecological footprint"[tw] OR "ecological footprint*"[tw] OR "ecological sustainability"[tw] OR "ecotoxic*"[tw] OR "ecotoxicity"[tw] OR "Emission reduction strategy"[tw] OR "Emission reduction"[tw] OR "Environment"[Mesh:noexp] OR "environmental impact"[tw] OR "environmental impact*"[tw] OR "environmental impacts"[tw] OR "environmental pollut*"[tw] OR "Environmental Pollution"[Mesh] OR "environmental pollution"[tw] OR "environmental protection"[tw] OR "environmental sustainab*"[tw] OR "environmental sustainability"[tw] OR "Environmental*"[ti] OR "EP in kg PO4 equivalent"[tw] OR "Equipment reuse"[mesh] OR "Equipment reuse"[tw] OR "Eutrophication potential"[tw] OR "Eutrophication"[Mesh] OR "eutrophication"[tw] OR "FAETP in kg DCB equivalent"[tw] OR "Freshwater Aquatic Ecotoxicity Potential"[tw] OR "Global Warming"[mesh] OR "Global Warming"[tw] OR "Green deal"[tw] OR "Green surgery"[tw] OR "Greenhouse Effect"[mesh] OR "greenhouse effect*"[tw] OR "greenhouse effects"[tw] OR "greenhouse gas emission"[tw] OR "greenhouse gas emissions"[tw] OR "Greenhouse Gas"[tw] OR "Greenhouse Gases"[mesh] OR "Greenhouse Gases"[tw] OR "greening"[tw] OR "GWP in kg CO2 equivalents"[tw] OR "H+ moles equivalents"[tw] OR "hospital waste"[tw] OR "HTTP in kg Dichlorobenzene equivalent"[tw] OR "Human Toxicity Potential"[tw] OR "kg 2.4-D equivalents"[tw] OR "kg CFC-11 equivalent"[tw] OR "kg N equivalents"[tw] OR "kg NOx equivalents"[tw] OR "LCA"[tw] OR "LCAs"[tw] OR "life cycle analysis"[tw] OR "life cycle assess*"[tw] OR "life cycle assessment"[tw] OR "life cycle inventories"[tw] OR "life cycle inventory"[tw] OR "Medical Waste Disposal"[mesh] OR "Medical Waste"[mesh] OR "medical waste"[tw] OR "N equiv*"[tw] OR "Ozone Depletion"[Mesh] OR "ozone depletion"[tw] OR "Photochemical Ozone Depletion Potential"[tw] OR "Plastic overuse"[tw] OR "POCP in kg ethane equivalent"[tw] OR "preservation of natural resources"[tw] OR "recycle*"[tw] OR "Recycling"[mesh] OR "recycling"[tw] OR "Refuse Disposal"[Mesh] OR "Refuse Disposal"[tw] OR "reusable"[tw] OR "Reusables"[tw] OR "reuse"[tw] OR "reused"[tw] OR "reusing"[tw] OR "Rising Sea Level"[tw] OR "Rising Sea Levels"[tw] OR "Sea Level Rise"[mesh] OR "Sea Level Rise"[tw] OR "Smog"[mesh] OR "smog"[tw] OR "SO2 equiv*"[tw] OR "sustainability"[ti] OR "Sustainable Development"[Mesh] OR "Sustainable Development"[tw] OR "Waste Disposal"[tw] OR "Waste Disposal, Fluid"[mesh] OR "Waste Management"[mesh] OR "Waste"[tw] OR "waste"[tw] OR "Waste Water"[Mesh] OR "Waste Water"[tw] OR "wastes"[tw] OR "Wastewater"[tw] OR "Water Purification"[Mesh] OR "Water Purification"[tw] OR (("plastic*"[tw] OR "microplastic*") AND ("soup"[tw] OR "pollution"[tw] OR "overuse"[tw] OR "contamination"[tw])) OR (("Plastic"[tw] OR "plastics"[tw]) AND "overuse"[tw]) OR ("hydrogen*"[tw] AND "moles"[tw] AND "equiv*"[tw]) OR ("Dichlorobenzen*"[tw] AND "equiv*"[tw]) OR ("2,4-D"[tw] AND "equiv*"[tw]) OR ("NOx"[tw] AND "equiv*"[tw]) OR ("ethane"[tw] AND "equiv*"[tw]) OR ("PO4"[tw] AND "equiv*"[tw]) OR ("DCB"[tw] AND "equiv*"[tw]) OR ("sustainability"[tw] AND ("environment*"[tw] OR "carbon"[tw])) OR (("Carbon Dioxide"[mesh] OR "Carbon Dioxide"[tw] OR "CO2"[tw]) AND ("pollution"[tw] OR "emission"[tw] OR "emissions"[tw] OR "waste"[tw] OR "environment"[tw] OR "environmental*"[tw] OR "footprint"[tw] OR "footprint*"[tw] OR "sustainable"[tw] OR "hazard"[tw] OR "hazard*"[tw]))) AND ("2000/01/01"[PDAT] : "3000/12/31"[PDAT])) AND (*("Meta-Analysis"[Publication Type] OR "Meta-Analysis as Topic"[Mesh] OR metaanaly*[tiab] OR meta-analy*[tiab] or metanaly*[tiab] OR "Systematic Review"[Publication Type] OR systematic[sb] OR "Cochrane Database Syst Rev"[Journal] or prisma[tiab] OR preferred reporting items[tiab] OR prospero[tiab] OR ((systemati*[ti] OR scoping[ti] OR umbrella[ti] OR structured literature[ti]) AND (review*[ti] OR overview*[ti])) OR systematic review*[tiab] OR scoping review*[tiab] OR umbrella review*[tiab] OR structured literature review*[tiab] OR systematic qualitative review*[tiab] OR systematic quantitative review*[tiab] OR systematic search and review[tiab] OR systematized review[tiab] OR systematised review[tiab] OR systemic review[tiab] OR systematic literature review*[tiab] OR systematic integrative literature review*[tiab] OR systematically review*[tiab] OR scoping literature review*[tiab] OR systematic critical review[tiab] OR systematic integrative review*[tiab] OR systematic evidence review[tiab] OR Systematic integrative literature review*[tiab] OR Systematic mixed studies review*[tiab] OR Systematized literature review*[tiab] OR Systematic overview*[tiab] OR Systematic narrative review*[tiab] OR ((systemati*[tiab] OR literature[tiab] OR database*[tiab] OR data-base*[tiab] OR structured[tiab] OR comprehensive*[tiab] OR systemic*[tiab]) AND search*[tiab]) OR (Literature[ti] AND review[ti] AND (database*[tiab] OR data-base*[tiab] OR search*[tiab])) OR ((data extraction[tiab] OR data source*[tiab]) AND study selection[tiab]) OR (search strategy[tiab] AND selection criteria[tiab]) OR (data source*[tiab] AND data synthesis[tiab]) OR medline[tiab] OR pubmed[tiab] OR embase[tiab] OR Cochrane[tiab] OR ((critical[ti] OR rapid[ti]) AND (review*[ti] OR overview*[ti] OR synthes*[ti])) OR (((critical*[tiab] OR rapid*[tiab]) AND (review*[tiab] OR overview*[tiab] OR synthes*[tiab]) AND (search*[tiab] OR database*[tiab] OR data-base*[tiab]))) OR metasynthes*[tiab] OR meta-synthes*[tiab])* OR ("Randomized Controlled Trial"[Publication Type] OR random*[tiab] OR pragmatic clinical trial*[tiab] OR practical clinical trial*[tiab] OR non-inferiority trial*[tiab] OR noninferiority trial*[tiab] OR superiority trial*[tiab] OR equivalence clinical trial*[tiab]) NOT (("Animals"[Mesh]) OR "Models, Animal"[Mesh] NOT humans[mh]) NOT (letter[pt] OR comment[pt] OR editorial[pt]) OR ("Comparative Study"[Publication Type] OR "comparison"[tiab] OR "comparative"[tiab] OR "compar*"[tiab] OR "Epidemiologic studies"[mesh:noexp] OR "case control studies"[mesh] OR "cohort studies"[mesh] OR "Controlled Before-After Studies"[mesh] OR "Case control"[tw] OR cohort*[tw] OR "Cohort analy*"[tw] OR "Follow up stud*"[tw] OR "observational stud*"[tw] OR Longitudinal[tw] OR Retrospective*[tw] OR prospective*[tw] OR consecutive*[tw] OR Cross sectional[tw] OR "Cross-sectional studies"[mesh] OR "historically controlled study"[mesh] OR "interrupted time series analysis"[mesh]) OR ("life cycle assess*"[tw] OR "life cycle assessment"[tw] OR "life cycle inventory"[tw] OR "LCA"[tw] OR "LCAs"[tw] OR "life cycle inventory"[tw] OR "life cycle inventories"[tw]))

**Embase (OVID)**

**UV4.1**

((exp "inhalation anesthetic agent"/ OR exp "inhalation anesthesia"/ OR "Closed-Circuit Anesthesia".mp OR "Endotracheal Anesthesia".mp OR "Closed-Circuit Anaesthesia".mp OR "Endotracheal Anaesthesia".mp OR "Halothane"/ OR "Isoflurane"/ OR "Methoxyflurane"/ OR "Sevoflurane"/ OR "Anaesthetic gas".mp OR "Anaesthetic gases".mp OR "Anesthetic gas".mp OR "Anesthetic gases".mp OR "Desflurane".mp OR "Enflurane".mp OR "Flurane".mp OR "Fluranes".mp OR "Gaseous anaesthesia".mp OR "Gaseous anaesthetic".mp OR "Gaseous anaesthetics".mp OR "Gaseous anesthesia".mp OR "Gaseous anesthetic".mp OR "Gaseous anesthetics".mp OR "Halothane".mp OR "Inhalation anaesthesia".mp OR "inhalation anaesthetic".mp OR "inhalation anaesthetics".mp OR "Inhalation anesthesia".mp OR "inhalation anesthetic".mp OR "inhalation anesthetics".mp OR "Inhalational agents".mp OR "Inhalational anaesthesia".mp OR "Inhalational anaesthetic".mp OR "Inhalational anaesthetic agent".mp OR "Inhalational anaesthetic agents".mp OR "Inhalational anaesthetics".mp OR "Inhalational anesthesia".mp OR "Inhalational anesthetic".mp OR "Inhalational anesthetic agent".mp OR "Inhalational anesthetic agents".mp OR "Inhalational anesthetics".mp OR "Inhaled anaesthetic".mp OR "Inhaled anaesthetic agent".mp OR "Inhaled anaesthetic agents".mp OR "Inhaled anaesthetics".mp OR "Inhaled anesthetic".mp OR "Inhaled anesthetic agents".mp OR "Inhaled anesthetics".mp OR "insufflation anaesthesia".mp OR "insufflation anaesthetic".mp OR "insufflation anaesthetics".mp OR "insufflation anesthesia".mp OR "insufflation anesthetic".mp OR "insufflation anesthetics".mp OR "Isoflurane".mp OR "Methoxyflurane".mp OR "Sevoflurane".mp OR "Vapor anaesthesia".mp OR "Vapor anaesthetic".mp OR "Vapor anaesthetics".mp OR "Vapor anesthesia".mp OR "Vapor anesthetic".mp OR "Vapor anesthetics".mp OR "Volatile agent".mp OR "Volatile agents".mp OR "Volatile Anaesthetic".mp OR "Volatile Anaesthetics".mp OR "Volatile anesthetic".mp OR "Volatile anesthetics".mp OR "Volatile drug".mp OR "Volatile drugs".mp OR "Volatile fluorinated liquid".mp OR "Volatile fluorinated liquids".mp OR "Volatile gas".mp OR "Volatile gases".mp OR "Volatile liquid agent".mp OR "Volatile liquid agents".mp OR "Anesthetic Gas".mp OR "Anesthetic Gases".mp OR "Inhalation Anaesthetic".mp OR "Inhalation Anaesthetics".mp OR "Inhalation Anesthetic".mp OR "Inhalation Anesthetics".mp) AND (exp "intravenous anesthetic agent"/ OR exp "Intravenous Anesthesia"/ OR "Intravenous Anesthetics".mp OR "Intravenous Anaesthetics".mp OR "Intravenous Anesthetic".mp OR "Intravenous Anaesthetic".mp OR "Intravenous Anesthesia".mp OR "Intravenous Anaesthesia".mp OR "Dissociative Anesthetics".mp OR "Dissociative Anaesthetics".mp OR "Dissociative Anesthetic".mp OR "Dissociative Anaesthetic".mp OR "Dissociative Anesthesia".mp OR "Dissociative Anaesthesia".mp OR "2-(3-methoxyphenyl)-2-(ethylamino)cyclohexanone"/ OR "2-Oxo-PCE"/ OR "Alfentanil"/ OR "Chloralose"/ OR "Diazepam"/ OR "Etomidate"/ OR "Fentanyl"/ OR "Ketamine"/ OR "Methohexital"/ OR "Midazolam"/ OR "Propanidid"/ OR "Propofol"/ OR "Sodium Oxybate"/ OR "Sufentanil"/ OR "Thiamylal"/ OR "Thiopental"/ OR "Tiletamine"/ OR "Urethane"/ OR "2-(3-methoxyphenyl)-2-(ethylamino)cyclohexanone".mp OR "2-Oxo-PCE".mp OR "Alfentanil".mp OR "Chloralose".mp OR "Diazepam".mp OR "Etomidate".mp OR "Fentanyl".mp OR "intravenous anaesthesia".mp OR "intravenous anaesthesias".mp OR "Intravenous anaesthetic".mp OR "Intravenous anaesthetic agent".mp OR "Intravenous anaesthetic agents".mp OR "Intravenous anaesthetic drug".mp OR "Intravenous anaesthetic drugs".mp OR "Intravenous anaesthetics".mp OR "intravenous anesthesia".mp OR "intravenous anesthesias".mp OR "intravenous anesthetic".mp OR "Intravenous anesthetic agents".mp OR "Intravenous anesthetic agents".mp OR "Intravenous anesthetic drug".mp OR "Intravenous anesthetic drugs".mp OR "intravenous anesthetics".mp OR "IV anaesthesia".mp OR "IV anaesthetics".mp OR "IV anaesthetics".mp OR "IV anesthesia".mp OR "IV anesthetic".mp OR "IV anesthetics".mp OR "Ketamine".mp OR "Methohexital".mp OR "Midazolam".mp OR "Propanidid".mp OR "Propofol".mp OR "Sodium Oxybate".mp OR "Sufentanil".mp OR "Thiamylal".mp OR "Thiopental".mp OR "Tiletamine".mp OR "TIVA".mp OR "Total intravenous anaesthesia".mp OR "Total Intravenous anesthesia".mp OR "Urethane".mp)) AND ("Carbon Footprint"/ OR "carbon footprint".mp OR "carbon footprint*".mp OR exp "Climate Change"/ OR "climate change".mp OR "CO2 emission".mp OR "CO2 emissions".mp OR "CO2 footprint".mp OR "CO2 footprint*".mp OR exp "environmental protection"/ OR "conservation of natural resources".mp OR "environmental protection".mp OR "Disposable Equipment"/ OR "Disposables".mp OR "Disposable".mp OR "ecological footprint".mp OR "ecological footprint*".mp OR "ecological sustainability".mp OR exp "environmental impact"/ OR "environmental impact".mp OR "environmental impact*".mp OR "environmental impacts".mp OR "environmental pollut*".mp OR exp "pollution"/ OR "environmental pollution".mp OR "environmental sustainab*".mp OR "environmental sustainability"/ OR "environmental sustainability".mp OR "Global Warming"/ OR "Global Warming".mp OR "Greenhouse Effect"/ OR "greenhouse effect*".mp OR "greenhouse effects".mp OR "greenhouse gas emission".mp OR "greenhouse gas emissions".mp OR "Greenhouse Gas"/ OR "greening".mp OR "hospital waste".mp OR "life cycle assessment"/ OR "environmental impact assessment"/ OR "life cycle assess*".mp OR "life cycle assessment".mp OR "LCA".mp OR "LCAs".mp OR "life cycle inventory".mp OR "life cycle inventories".mp OR exp "Waste Disposal"/ OR exp "Hospital Waste"/ OR "medical waste".mp OR "Rising Sea Level".mp OR "Rising Sea Levels".mp OR "Sea Level Rise"/ OR "Sea Level Rise".mp OR "sustainability".ti OR "Waste Disposal".mp OR "waste water recycling"/ OR "Recycling"/ OR "recycling".mp OR "recycle*".mp OR "Equipment reuse".mp OR "Reusables".mp OR "reusable".mp OR "reuse".mp OR "reused".mp OR "reusing".mp OR exp "Waste Disposal"/ OR exp "Waste Management"/ OR "Plastic overuse".mp OR "Green surgery".mp OR "Emission reduction".mp OR "Emission reduction strategy".mp OR "air pollution control"/ OR "air pollution control".mp OR "Environment"/ OR "Environmental*".ti OR "acidification"/ OR "soil acidification"/ OR "ocean acidification"/ OR "acidification".mp OR "Acidification potential".mp OR "AP in kg SO2 equivalents".mp OR "eco-efficiency".mp OR "ecoefficiency".mp OR "eco-efficien*".mp OR "ecoefficien*".mp OR "ecotoxicity"/ OR "ecotoxicity".mp OR "ecotoxic*".mp OR "eco toxicity".mp OR "eco toxic*".mp OR "EP in kg PO4 equivalent".mp OR exp "Eutrophication"/ OR "eutrophication".mp OR "Eutrophication potential".mp OR "FAETP in kg DCB equivalent".mp OR "Freshwater Aquatic Ecotoxicity Potential".mp OR "GWP in kg CO2 equivalents".mp OR "H+ moles equivalents".mp OR "HTTP in kg Dichlorobenzene equivalent".mp OR "Human Toxicity Potential".mp OR "kg 2.4-D equivalents".mp OR "kg CFC-11 equivalent".mp OR "kg N equivalents".mp OR "kg NOx equivalents".mp OR "life cycle analysis".mp OR "ozone depletion".mp OR "Photochemical Ozone Depletion Potential".mp OR "POCP in kg ethane equivalent".mp OR "smog".mp OR exp "Waste"/ OR "waste".mp OR "wastes".mp OR "Ozone Depletion"/ OR "Smog"/ OR "Equipment reuse".mp OR "Greenhouse Gases".mp OR "Greenhouse Gas".mp OR "SO2 equiv*".mp OR "CO2 equiva*".mp OR "CFC-11 equiv*".mp OR "N equiv*".mp OR exp "Biodiversity"/ OR "Biodiversity".mp OR "Climatic change".mp OR "Green deal".mp OR "preservation of natural resources".mp OR "Refuse Disposal".mp OR exp "Wastewater"/ OR "Waste Water".mp OR "Wastewater".mp OR exp "Water Management"/ OR "Water Purification".mp OR (("plastic*".mp OR "microplastic*".mp) AND ("soup".mp OR "pollution".mp OR "overuse".mp OR "contamination".mp)) OR "Sustainable Development"/ OR "Sustainable Development".mp OR (("Plastic".mp OR "plastics".mp) AND "overuse".mp) OR ("hydrogen*".mp AND "moles".mp AND "equiv*".mp) OR ("Dichlorobenzen*".mp AND "equiv*".mp) OR ("2,4-D".mp AND "equiv*".mp) OR ("NOx".mp AND "equiv*".mp) OR ("ethane".mp AND "equiv*".mp) OR ("PO4".mp AND "equiv*".mp) OR ("DCB".mp AND "equiv*".mp) OR ("sustainability".mp AND ("environment*".mp OR "carbon".mp)) OR (("Carbon Dioxide"/ OR "Carbon Dioxide".mp OR "CO2".mp) AND ("pollution".mp OR "emission".mp OR "emissions".mp OR "waste".mp OR "environment".mp OR "environmental*".mp OR "footprint".mp OR "footprint*".mp OR "sustainable".mp OR "hazard".mp OR "hazard*".mp))) NOT (conference review or conference abstract).pt AND (2000 OR 2001 OR 2002 OR 2003 OR 2004 OR 2005 OR 2006 OR 2007 OR 2008 OR 2009 OR 2010 OR 2011 OR 2012 OR 2013 OR 2014 OR 2015 OR 2016 OR 2017 OR 2018 OR 2019 OR 2020 OR 2021 OR 2022).yr AND (*(exp "meta analysis"/ OR exp "meta analysis (topic)"/ OR metaanaly*.ti,ab OR "meta analy*".ti,ab OR metanaly*.ti,ab OR "systematic review"/ OR "cochrane database of systematic reviews".jn OR prisma.ti,ab OR prospero.ti,ab OR (((systemati* OR scoping OR umbrella OR "structured literature") ADJ3 (review* OR overview*)).ti,ab) OR ((systemic* ADJ1 review*).ti,ab) OR (((systemati* OR literature OR database* OR "data base*") ADJ10 search*).ti,ab) OR (((structured OR comprehensive* OR systemic*) ADJ3 search*).ti,ab) OR (((literature ADJ3 review*).ti,ab) AND (search*.ti,ab OR database*.ti,ab OR "data base*".ti,ab)) OR (("data extraction".ti,ab OR "data source*".ti,ab) AND "study selection".ti,ab) OR ("search strategy".ti,ab AND "selection criteria".ti,ab) OR ("data source*".ti,ab AND "data synthesis".ti,ab) OR medline.ab OR pubmed.ab OR embase.ab OR cochrane.ab OR (((critical OR rapid) ADJ2 (review* OR overview* OR synthes*)).ti) OR ((((critical* OR rapid*) ADJ3 (review* OR overview* OR synthes*)).ab) AND (search*.ab OR database*.ab OR "data base*".ab)) OR metasynthes*.ti,ab OR "meta synthes*".ti,ab)* OR (exp "clinical trial"/ OR exp "randomization"/ OR exp "single blind procedure"/ OR exp "double blind procedure"/ OR exp "crossover procedure"/ OR exp "placebo"/ OR exp "prospective study"/ OR rct.ti,ab OR random*.ti,ab OR "single blind".ti,ab OR "randomised controlled trial".ti,ab OR exp "randomized controlled trial"/ OR placebo*.ti,ab) OR (exp "Comparative Study"/ OR "comparison".ti,ab OR "comparative".ti,ab OR "compar*".ti,ab OR "major clinical study"/ OR "clinical study"/ OR "case control study"/ OR "family study"/ OR "longitudinal study"/ OR "retrospective study"/ OR "prospective study"/ OR "cohort analysis"/ OR cohort*.ti,ab OR (("case control" ADJ1 (study OR studies)).ti,ab) OR (("follow up" ADJ1 (study OR studies)).ti,ab) OR (observational ADJ1 (study OR studies)).ti,ab OR ((epidemiologic ADJ1 (study OR studies)).ti,ab) OR (("cross sectional" ADJ1 (study OR studies)).ti,ab)) OR ("life cycle assessment"/ OR "environmental impact assessment"/ OR "life cycle assess*".mp OR "life cycle assessment".mp OR "life cycle inventory".mp OR "LCA".mp OR "LCAs".mp OR "life cycle inventory".mp OR "life cycle inventories".mp))

**UV4.2**

((exp "inhalation anesthetic agent"/ OR exp "inhalation anesthesia"/ OR "Closed-Circuit Anesthesia".mp OR "Endotracheal Anesthesia".mp OR "Closed-Circuit Anaesthesia".mp OR "Endotracheal Anaesthesia".mp OR "Halothane"/ OR "Isoflurane"/ OR "Methoxyflurane"/ OR "Sevoflurane"/ OR "Anaesthetic gas".mp OR "Anaesthetic gases".mp OR "Anesthetic gas".mp OR "Anesthetic gases".mp OR "Desflurane".mp OR "Enflurane".mp OR "Flurane".mp OR "Fluranes".mp OR "Gaseous anaesthesia".mp OR "Gaseous anaesthetic".mp OR "Gaseous anaesthetics".mp OR "Gaseous anesthesia".mp OR "Gaseous anesthetic".mp OR "Gaseous anesthetics".mp OR "Halothane".mp OR "Inhalation anaesthesia".mp OR "inhalation anaesthetic".mp OR "inhalation anaesthetics".mp OR "Inhalation anesthesia".mp OR "inhalation anesthetic".mp OR "inhalation anesthetics".mp OR "Inhalational agents".mp OR "Inhalational anaesthesia".mp OR "Inhalational anaesthetic".mp OR "Inhalational anaesthetic agent".mp OR "Inhalational anaesthetic agents".mp OR "Inhalational anaesthetics".mp OR "Inhalational anesthesia".mp OR "Inhalational anesthetic".mp OR "Inhalational anesthetic agent".mp OR "Inhalational anesthetic agents".mp OR "Inhalational anesthetics".mp OR "Inhaled anaesthetic".mp OR "Inhaled anaesthetic agent".mp OR "Inhaled anaesthetic agents".mp OR "Inhaled anaesthetics".mp OR "Inhaled anesthetic".mp OR "Inhaled anesthetic agents".mp OR "Inhaled anesthetics".mp OR "insufflation anaesthesia".mp OR "insufflation anaesthetic".mp OR "insufflation anaesthetics".mp OR "insufflation anesthesia".mp OR "insufflation anesthetic".mp OR "insufflation anesthetics".mp OR "Isoflurane".mp OR "Methoxyflurane".mp OR "Sevoflurane".mp OR "Vapor anaesthesia".mp OR "Vapor anaesthetic".mp OR "Vapor anaesthetics".mp OR "Vapor anesthesia".mp OR "Vapor anesthetic".mp OR "Vapor anesthetics".mp OR "Volatile agent".mp OR "Volatile agents".mp OR "Volatile Anaesthetic".mp OR "Volatile Anaesthetics".mp OR "Volatile anesthetic".mp OR "Volatile anesthetics".mp OR "Volatile drug".mp OR "Volatile drugs".mp OR "Volatile fluorinated liquid".mp OR "Volatile fluorinated liquids".mp OR "Volatile gas".mp OR "Volatile gases".mp OR "Volatile liquid agent".mp OR "Volatile liquid agents".mp OR "Anesthetic Gas".mp OR "Anesthetic Gases".mp OR "Inhalation Anaesthetic".mp OR "Inhalation Anaesthetics".mp OR "Inhalation Anesthetic".mp OR "Inhalation Anesthetics".mp) AND ("scavenging system"/ OR "anaesthetic gas scavenging system".mp OR "anesthetic gas scavenging system".mp OR "Contrafluran".mp OR "gas extract*".mp OR "gas extraction".mp OR "gas extraction system".mp OR "gas extraction systems".mp OR "gas scaveng*".mp OR "gas scavenger".mp OR "gas scavengers".mp OR "gas scavenging ".mp OR "scavenging device".mp OR "scavenging device".mp OR "scavenging devices".mp OR "scavenging system".mp OR "scavenging systems".mp OR "vapour captur*".mp OR "vapour capture".mp OR "Vapour Capture Technology".mp OR "vapour recycl*".mp OR "vapour recycling technology".mp OR "vapour recycling".mp)) AND ("Carbon Footprint"/ OR "carbon footprint".mp OR "carbon footprint*".mp OR exp "Climate Change"/ OR "climate change".mp OR "CO2 emission".mp OR "CO2 emissions".mp OR "CO2 footprint".mp OR "CO2 footprint*".mp OR exp "environmental protection"/ OR "conservation of natural resources".mp OR "environmental protection".mp OR "Disposable Equipment"/ OR "Disposables".mp OR "Disposable".mp OR "ecological footprint".mp OR "ecological footprint*".mp OR "ecological sustainability".mp OR exp "environmental impact"/ OR "environmental impact".mp OR "environmental impact*".mp OR "environmental impacts".mp OR "environmental pollut*".mp OR exp "pollution"/ OR "environmental pollution".mp OR "environmental sustainab*".mp OR "environmental sustainability"/ OR "environmental sustainability".mp OR "Global Warming"/ OR "Global Warming".mp OR "Greenhouse Effect"/ OR "greenhouse effect*".mp OR "greenhouse effects".mp OR "greenhouse gas emission".mp OR "greenhouse gas emissions".mp OR "Greenhouse Gas"/ OR "greening".mp OR "hospital waste".mp OR "life cycle assessment"/ OR "environmental impact assessment"/ OR "life cycle assess*".mp OR "life cycle assessment".mp OR "LCA".mp OR "LCAs".mp OR "life cycle inventory".mp OR "life cycle inventories".mp OR exp "Waste Disposal"/ OR exp "Hospital Waste"/ OR "medical waste".mp OR "Rising Sea Level".mp OR "Rising Sea Levels".mp OR "Sea Level Rise"/ OR "Sea Level Rise".mp OR "sustainability".ti OR "Waste Disposal".mp OR "waste water recycling"/ OR "Recycling"/ OR "recycling".mp OR "recycle*".mp OR "Equipment reuse".mp OR "Reusables".mp OR "reusable".mp OR "reuse".mp OR "reused".mp OR "reusing".mp OR exp "Waste Disposal"/ OR exp "Waste Management"/ OR "Plastic overuse".mp OR "Green surgery".mp OR "Emission reduction".mp OR "Emission reduction strategy".mp OR "air pollution control"/ OR "air pollution control".mp OR "Environment"/ OR "Environmental*".ti OR "acidification"/ OR "soil acidification"/ OR "ocean acidification"/ OR "acidification".mp OR "Acidification potential".mp OR "AP in kg SO2 equivalents".mp OR "eco-efficiency".mp OR "ecoefficiency".mp OR "eco-efficien*".mp OR "ecoefficien*".mp OR "ecotoxicity"/ OR "ecotoxicity".mp OR "ecotoxic*".mp OR "eco toxicity".mp OR "eco toxic*".mp OR "EP in kg PO4 equivalent".mp OR exp "Eutrophication"/ OR "eutrophication".mp OR "Eutrophication potential".mp OR "FAETP in kg DCB equivalent".mp OR "Freshwater Aquatic Ecotoxicity Potential".mp OR "GWP in kg CO2 equivalents".mp OR "H+ moles equivalents".mp OR "HTTP in kg Dichlorobenzene equivalent".mp OR "Human Toxicity Potential".mp OR "kg 2.4-D equivalents".mp OR "kg CFC-11 equivalent".mp OR "kg N equivalents".mp OR "kg NOx equivalents".mp OR "life cycle analysis".mp OR "ozone depletion".mp OR "Photochemical Ozone Depletion Potential".mp OR "POCP in kg ethane equivalent".mp OR "smog".mp OR exp "Waste"/ OR "waste".mp OR "wastes".mp OR "Ozone Depletion"/ OR "Smog"/ OR "Equipment reuse".mp OR "Greenhouse Gases".mp OR "Greenhouse Gas".mp OR "SO2 equiv*".mp OR "CO2 equiva*".mp OR "CFC-11 equiv*".mp OR "N equiv*".mp OR exp "Biodiversity"/ OR "Biodiversity".mp OR "Climatic change".mp OR "Green deal".mp OR "preservation of natural resources".mp OR "Refuse Disposal".mp OR exp "Wastewater"/ OR "Waste Water".mp OR "Wastewater".mp OR exp "Water Management"/ OR "Water Purification".mp OR (("plastic*".mp OR "microplastic*".mp) AND ("soup".mp OR "pollution".mp OR "overuse".mp OR "contamination".mp)) OR "Sustainable Development"/ OR "Sustainable Development".mp OR (("Plastic".mp OR "plastics".mp) AND "overuse".mp) OR ("hydrogen*".mp AND "moles".mp AND "equiv*".mp) OR ("Dichlorobenzen*".mp AND "equiv*".mp) OR ("2,4-D".mp AND "equiv*".mp) OR ("NOx".mp AND "equiv*".mp) OR ("ethane".mp AND "equiv*".mp) OR ("PO4".mp AND "equiv*".mp) OR ("DCB".mp AND "equiv*".mp) OR ("sustainability".mp AND ("environment*".mp OR "carbon".mp)) OR (("Carbon Dioxide"/ OR "Carbon Dioxide".mp OR "CO2".mp) AND ("pollution".mp OR "emission".mp OR "emissions".mp OR "waste".mp OR "environment".mp OR "environmental*".mp OR "footprint".mp OR "footprint*".mp OR "sustainable".mp OR "hazard".mp OR "hazard*".mp))) NOT (conference review or conference abstract).pt AND (2000 OR 2001 OR 2002 OR 2003 OR 2004 OR 2005 OR 2006 OR 2007 OR 2008 OR 2009 OR 2010 OR 2011 OR 2012 OR 2013 OR 2014 OR 2015 OR 2016 OR 2017 OR 2018 OR 2019 OR 2020 OR 2021 OR 2022).yr AND (*(exp "meta analysis"/ OR exp "meta analysis (topic)"/ OR metaanaly*.ti,ab OR "meta analy*".ti,ab OR metanaly*.ti,ab OR "systematic review"/ OR "cochrane database of systematic reviews".jn OR prisma.ti,ab OR prospero.ti,ab OR (((systemati* OR scoping OR umbrella OR "structured literature") ADJ3 (review* OR overview*)).ti,ab) OR ((systemic* ADJ1 review*).ti,ab) OR (((systemati* OR literature OR database* OR "data base*") ADJ10 search*).ti,ab) OR (((structured OR comprehensive* OR systemic*) ADJ3 search*).ti,ab) OR (((literature ADJ3 review*).ti,ab) AND (search*.ti,ab OR database*.ti,ab OR "data base*".ti,ab)) OR (("data extraction".ti,ab OR "data source*".ti,ab) AND "study selection".ti,ab) OR ("search strategy".ti,ab AND "selection criteria".ti,ab) OR ("data source*".ti,ab AND "data synthesis".ti,ab) OR medline.ab OR pubmed.ab OR embase.ab OR cochrane.ab OR (((critical OR rapid) ADJ2 (review* OR overview* OR synthes*)).ti) OR ((((critical* OR rapid*) ADJ3 (review* OR overview* OR synthes*)).ab) AND (search*.ab OR database*.ab OR "data base*".ab)) OR metasynthes*.ti,ab OR "meta synthes*".ti,ab)* OR (exp "clinical trial"/ OR exp "randomization"/ OR exp "single blind procedure"/ OR exp "double blind procedure"/ OR exp "crossover procedure"/ OR exp "placebo"/ OR exp "prospective study"/ OR rct.ti,ab OR random*.ti,ab OR "single blind".ti,ab OR "randomised controlled trial".ti,ab OR exp "randomized controlled trial"/ OR placebo*.ti,ab) OR (exp "Comparative Study"/ OR "comparison".ti,ab OR "comparative".ti,ab OR "compar*".ti,ab OR "major clinical study"/ OR "clinical study"/ OR "case control study"/ OR "family study"/ OR "longitudinal study"/ OR "retrospective study"/ OR "prospective study"/ OR "cohort analysis"/ OR cohort*.ti,ab OR (("case control" ADJ1 (study OR studies)).ti,ab) OR (("follow up" ADJ1 (study OR studies)).ti,ab) OR (observational ADJ1 (study OR studies)).ti,ab OR ((epidemiologic ADJ1 (study OR studies)).ti,ab) OR (("cross sectional" ADJ1 (study OR studies)).ti,ab)) OR ("life cycle assessment"/ OR "environmental impact assessment"/ OR "life cycle assess*".mp OR "life cycle assessment".mp OR "life cycle inventory".mp OR "LCA".mp OR "LCAs".mp OR "life cycle inventory".mp OR "life cycle inventories".mp))

**UV4.3**

((exp "Local Anesthesia"/ OR exp *"local anesthetic agent"/ OR "Local Anesthesia".mp OR "Local Anaesthesia".mp OR "Locoregional Anesthesia".mp OR "Locoregional Anaesthesia".mp OR "Local Anesthetics".mp OR "Local Anaesthetics".mp OR "Locoregional Anesthetics".mp OR "Locoregional Anaesthetics".mp OR "Local Anesthetic".mp OR "Local Anaesthetic".mp OR "Locoregional Anesthetic".mp OR "Locoregional Anaesthetic".mp OR "axillary block".mp OR "axillary block*".mp OR "Bier block".mp OR "Bier block*".mp OR "Bier's block".mp OR "Bier's block*".mp OR "brachial plexus block".mp OR "brachial plexus block*".mp OR "combined spinal epidural".mp OR "combined spinal epidural block".mp OR "combined spinal epidural block*".mp OR "epidural anaesthesia".mp OR "epidural anesthesia".mp OR "intravenous regional anesthaesia".mp OR "intravenous regional anesthesia".mp OR "ischiadic block".mp OR "ischiadic block*".mp OR "local infiltration".mp OR "lower extremity block".mp OR "lower extremity block*".mp OR "nerve block".mp OR "nerve block".mp OR "nerve block*".mp OR "nerve block*".mp OR "neuraxial anaesthesia".mp OR "neuraxial anesthesia".mp OR "neuraxial block".mp OR "neuraxial block*".mp OR "neuraxial technique".mp OR "neuraxial techniques".mp OR "peripheral nerve block".mp OR "peripheral nerve block*".mp OR "plexus block".mp OR "plexus block*".mp OR "plexus nerve block".mp OR "plexus nerve block*".mp OR "popliteal block".mp OR "popliteal block*".mp OR "regional anaesthesia".mp OR "regional anesthesia".mp OR "spinal anaesthesia".mp OR "spinal anaesthetic block".mp OR "spinal anaesthetic block*".mp OR "spinal anesthesia".mp OR "spinal anesthetic block".mp OR "spinal anesthetic block*".mp OR "supraclavicular block".mp OR "supraclavicular block*".mp OR "upper extremity block".mp OR "upper extremity block*".mp) AND (exp "General Anesthesia"/ OR "general anesthesia".mp OR "general anaesthesia".mp OR "general anesthe*".mp OR "general anaesthe*".mp OR exp "inhalation anesthetic agent"/ OR exp "inhalation anesthesia"/ OR "Closed-Circuit Anesthesia".mp OR "Endotracheal Anesthesia".mp OR "Closed-Circuit Anaesthesia".mp OR "Endotracheal Anaesthesia".mp OR "Halothane"/ OR "Isoflurane"/ OR "Methoxyflurane"/ OR "Sevoflurane"/ OR "Anaesthetic gas".mp OR "Anaesthetic gases".mp OR "Anesthetic gas".mp OR "Anesthetic gases".mp OR "Desflurane".mp OR "Enflurane".mp OR "Flurane".mp OR "Fluranes".mp OR "Gaseous anaesthesia".mp OR "Gaseous anaesthetic".mp OR "Gaseous anaesthetics".mp OR "Gaseous anesthesia".mp OR "Gaseous anesthetic".mp OR "Gaseous anesthetics".mp OR "Halothane".mp OR "Inhalation anaesthesia".mp OR "inhalation anaesthetic".mp OR "inhalation anaesthetics".mp OR "Inhalation anesthesia".mp OR "inhalation anesthetic".mp OR "inhalation anesthetics".mp OR "Inhalational agents".mp OR "Inhalational anaesthesia".mp OR "Inhalational anaesthetic".mp OR "Inhalational anaesthetic agent".mp OR "Inhalational anaesthetic agents".mp OR "Inhalational anaesthetics".mp OR "Inhalational anesthesia".mp OR "Inhalational anesthetic".mp OR "Inhalational anesthetic agent".mp OR "Inhalational anesthetic agents".mp OR "Inhalational anesthetics".mp OR "Inhaled anaesthetic".mp OR "Inhaled anaesthetic agent".mp OR "Inhaled anaesthetic agents".mp OR "Inhaled anaesthetics".mp OR "Inhaled anesthetic".mp OR "Inhaled anesthetic agents".mp OR "Inhaled anesthetics".mp OR "insufflation anaesthesia".mp OR "insufflation anaesthetic".mp OR "insufflation anaesthetics".mp OR "insufflation anesthesia".mp OR "insufflation anesthetic".mp OR "insufflation anesthetics".mp OR "Isoflurane".mp OR "Methoxyflurane".mp OR "Sevoflurane".mp OR "Vapor anaesthesia".mp OR "Vapor anaesthetic".mp OR "Vapor anaesthetics".mp OR "Vapor anesthesia".mp OR "Vapor anesthetic".mp OR "Vapor anesthetics".mp OR "Volatile agent".mp OR "Volatile agents".mp OR "Volatile Anaesthetic".mp OR "Volatile Anaesthetics".mp OR "Volatile anesthetic".mp OR "Volatile anesthetics".mp OR "Volatile drug".mp OR "Volatile drugs".mp OR "Volatile fluorinated liquid".mp OR "Volatile fluorinated liquids".mp OR "Volatile gas".mp OR "Volatile gases".mp OR "Volatile liquid agent".mp OR "Volatile liquid agents".mp OR "Anesthetic Gas".mp OR "Anesthetic Gases".mp OR "Inhalation Anaesthetic".mp OR "Inhalation Anaesthetics".mp OR "Inhalation Anesthetic".mp OR "Inhalation Anesthetics".mp OR exp "intravenous anesthetic agent"/ OR exp "Intravenous Anesthesia"/ OR "Intravenous Anesthetics".mp OR "Intravenous Anaesthetics".mp OR "Intravenous Anesthetic".mp OR "Intravenous Anaesthetic".mp OR "Intravenous Anesthesia".mp OR "Intravenous Anaesthesia".mp OR "Dissociative Anesthetics".mp OR "Dissociative Anaesthetics".mp OR "Dissociative Anesthetic".mp OR "Dissociative Anaesthetic".mp OR "Dissociative Anesthesia".mp OR "Dissociative Anaesthesia".mp OR "2-(3-methoxyphenyl)-2-(ethylamino)cyclohexanone"/ OR "2-Oxo-PCE"/ OR "Alfentanil"/ OR "Chloralose"/ OR "Diazepam"/ OR "Etomidate"/ OR "Fentanyl"/ OR "Ketamine"/ OR "Methohexital"/ OR "Midazolam"/ OR "Propanidid"/ OR "Propofol"/ OR "Sodium Oxybate"/ OR "Sufentanil"/ OR "Thiamylal"/ OR "Thiopental"/ OR "Tiletamine"/ OR "Urethane"/ OR "2-(3-methoxyphenyl)-2-(ethylamino)cyclohexanone".mp OR "2-Oxo-PCE".mp OR "Alfentanil".mp OR "Chloralose".mp OR "Diazepam".mp OR "Etomidate".mp OR "Fentanyl".mp OR "intravenous anaesthesia".mp OR "intravenous anaesthesias".mp OR "Intravenous anaesthetic".mp OR "Intravenous anaesthetic agent".mp OR "Intravenous anaesthetic agents".mp OR "Intravenous anaesthetic drug".mp OR "Intravenous anaesthetic drugs".mp OR "Intravenous anaesthetics".mp OR "intravenous anesthesia".mp OR "intravenous anesthesias".mp OR "intravenous anesthetic".mp OR "Intravenous anesthetic agents".mp OR "Intravenous anesthetic agents".mp OR "Intravenous anesthetic drug".mp OR "Intravenous anesthetic drugs".mp OR "intravenous anesthetics".mp OR "IV anaesthesia".mp OR "IV anaesthetics".mp OR "IV anaesthetics".mp OR "IV anesthesia".mp OR "IV anesthetic".mp OR "IV anesthetics".mp OR "Ketamine".mp OR "Methohexital".mp OR "Midazolam".mp OR "Propanidid".mp OR "Propofol".mp OR "Sodium Oxybate".mp OR "Sufentanil".mp OR "Thiamylal".mp OR "Thiopental".mp OR "Tiletamine".mp OR "TIVA".mp OR "Total intravenous anaesthesia".mp OR "Total Intravenous anesthesia".mp OR "Urethane".mp)) AND ("Carbon Footprint"/ OR "carbon footprint".mp OR "carbon footprint*".mp OR exp "Climate Change"/ OR "climate change".mp OR "CO2 emission".mp OR "CO2 emissions".mp OR "CO2 footprint".mp OR "CO2 footprint*".mp OR exp "environmental protection"/ OR "conservation of natural resources".mp OR "environmental protection".mp OR "Disposable Equipment"/ OR "Disposables".mp OR "Disposable".mp OR "ecological footprint".mp OR "ecological footprint*".mp OR "ecological sustainability".mp OR exp "environmental impact"/ OR "environmental impact".mp OR "environmental impact*".mp OR "environmental impacts".mp OR "environmental pollut*".mp OR exp "pollution"/ OR "environmental pollution".mp OR "environmental sustainab*".mp OR "environmental sustainability"/ OR "environmental sustainability".mp OR "Global Warming"/ OR "Global Warming".mp OR "Greenhouse Effect"/ OR "greenhouse effect*".mp OR "greenhouse effects".mp OR "greenhouse gas emission".mp OR "greenhouse gas emissions".mp OR "Greenhouse Gas"/ OR "greening".mp OR "hospital waste".mp OR "life cycle assessment"/ OR "environmental impact assessment"/ OR "life cycle assess*".mp OR "life cycle assessment".mp OR "LCA".mp OR "LCAs".mp OR "life cycle inventory".mp OR "life cycle inventories".mp OR exp "Waste Disposal"/ OR exp "Hospital Waste"/ OR "medical waste".mp OR "Rising Sea Level".mp OR "Rising Sea Levels".mp OR "Sea Level Rise"/ OR "Sea Level Rise".mp OR "sustainability".ti OR "Waste Disposal".mp OR "waste water recycling"/ OR "Recycling"/ OR "recycling".mp OR "recycle*".mp OR "Equipment reuse".mp OR "Reusables".mp OR "reusable".mp OR "reuse".mp OR "reused".mp OR "reusing".mp OR exp "Waste Disposal"/ OR exp "Waste Management"/ OR "Plastic overuse".mp OR "Green surgery".mp OR "Emission reduction".mp OR "Emission reduction strategy".mp OR "air pollution control"/ OR "air pollution control".mp OR "Environment"/ OR "Environmental*".ti OR "acidification"/ OR "soil acidification"/ OR "ocean acidification"/ OR "acidification".mp OR "Acidification potential".mp OR "AP in kg SO2 equivalents".mp OR "eco-efficiency".mp OR "ecoefficiency".mp OR "eco-efficien*".mp OR "ecoefficien*".mp OR "ecotoxicity"/ OR "ecotoxicity".mp OR "ecotoxic*".mp OR "eco toxicity".mp OR "eco toxic*".mp OR "EP in kg PO4 equivalent".mp OR exp "Eutrophication"/ OR "eutrophication".mp OR "Eutrophication potential".mp OR "FAETP in kg DCB equivalent".mp OR "Freshwater Aquatic Ecotoxicity Potential".mp OR "GWP in kg CO2 equivalents".mp OR "H+ moles equivalents".mp OR "HTTP in kg Dichlorobenzene equivalent".mp OR "Human Toxicity Potential".mp OR "kg 2.4-D equivalents".mp OR "kg CFC-11 equivalent".mp OR "kg N equivalents".mp OR "kg NOx equivalents".mp OR "life cycle analysis".mp OR "ozone depletion".mp OR "Photochemical Ozone Depletion Potential".mp OR "POCP in kg ethane equivalent".mp OR "smog".mp OR exp "Waste"/ OR "waste".mp OR "wastes".mp OR "Ozone Depletion"/ OR "Smog"/ OR "Equipment reuse".mp OR "Greenhouse Gases".mp OR "Greenhouse Gas".mp OR "SO2 equiv*".mp OR "CO2 equiva*".mp OR "CFC-11 equiv*".mp OR "N equiv*".mp OR exp "Biodiversity"/ OR "Biodiversity".mp OR "Climatic change".mp OR "Green deal".mp OR "preservation of natural resources".mp OR "Refuse Disposal".mp OR exp "Wastewater"/ OR "Waste Water".mp OR "Wastewater".mp OR exp "Water Management"/ OR "Water Purification".mp OR (("plastic*".mp OR "microplastic*".mp) AND ("soup".mp OR "pollution".mp OR "overuse".mp OR "contamination".mp)) OR "Sustainable Development"/ OR "Sustainable Development".mp OR (("Plastic".mp OR "plastics".mp) AND "overuse".mp) OR ("hydrogen*".mp AND "moles".mp AND "equiv*".mp) OR ("Dichlorobenzen*".mp AND "equiv*".mp) OR ("2,4-D".mp AND "equiv*".mp) OR ("NOx".mp AND "equiv*".mp) OR ("ethane".mp AND "equiv*".mp) OR ("PO4".mp AND "equiv*".mp) OR ("DCB".mp AND "equiv*".mp) OR ("sustainability".mp AND ("environment*".mp OR "carbon".mp)) OR (("Carbon Dioxide"/ OR "Carbon Dioxide".mp OR "CO2".mp) AND ("pollution".mp OR "emission".mp OR "emissions".mp OR "waste".mp OR "environment".mp OR "environmental*".mp OR "footprint".mp OR "footprint*".mp OR "sustainable".mp OR "hazard".mp OR "hazard*".mp))) NOT (conference review or conference abstract).pt AND (2000 OR 2001 OR 2002 OR 2003 OR 2004 OR 2005 OR 2006 OR 2007 OR 2008 OR 2009 OR 2010 OR 2011 OR 2012 OR 2013 OR 2014 OR 2015 OR 2016 OR 2017 OR 2018 OR 2019 OR 2020 OR 2021 OR 2022).yr AND (*(exp "meta analysis"/ OR exp "meta analysis (topic)"/ OR metaanaly*.ti,ab OR "meta analy*".ti,ab OR metanaly*.ti,ab OR "systematic review"/ OR "cochrane database of systematic reviews".jn OR prisma.ti,ab OR prospero.ti,ab OR (((systemati* OR scoping OR umbrella OR "structured literature") ADJ3 (review* OR overview*)).ti,ab) OR ((systemic* ADJ1 review*).ti,ab) OR (((systemati* OR literature OR database* OR "data base*") ADJ10 search*).ti,ab) OR (((structured OR comprehensive* OR systemic*) ADJ3 search*).ti,ab) OR (((literature ADJ3 review*).ti,ab) AND (search*.ti,ab OR database*.ti,ab OR "data base*".ti,ab)) OR (("data extraction".ti,ab OR "data source*".ti,ab) AND "study selection".ti,ab) OR ("search strategy".ti,ab AND "selection criteria".ti,ab) OR ("data source*".ti,ab AND "data synthesis".ti,ab) OR medline.ab OR pubmed.ab OR embase.ab OR cochrane.ab OR (((critical OR rapid) ADJ2 (review* OR overview* OR synthes*)).ti) OR ((((critical* OR rapid*) ADJ3 (review* OR overview* OR synthes*)).ab) AND (search*.ab OR database*.ab OR "data base*".ab)) OR metasynthes*.ti,ab OR "meta synthes*".ti,ab)* OR (exp "clinical trial"/ OR exp "randomization"/ OR exp "single blind procedure"/ OR exp "double blind procedure"/ OR exp "crossover procedure"/ OR exp "placebo"/ OR exp "prospective study"/ OR rct.ti,ab OR random*.ti,ab OR "single blind".ti,ab OR "randomised controlled trial".ti,ab OR exp "randomized controlled trial"/ OR placebo*.ti,ab) OR (exp "Comparative Study"/ OR "comparison".ti,ab OR "comparative".ti,ab OR "compar*".ti,ab OR "major clinical study"/ OR "clinical study"/ OR "case control study"/ OR "family study"/ OR "longitudinal study"/ OR "retrospective study"/ OR "prospective study"/ OR "cohort analysis"/ OR cohort*.ti,ab OR (("case control" ADJ1 (study OR studies)).ti,ab) OR (("follow up" ADJ1 (study OR studies)).ti,ab) OR (observational ADJ1 (study OR studies)).ti,ab OR ((epidemiologic ADJ1 (study OR studies)).ti,ab) OR (("cross sectional" ADJ1 (study OR studies)).ti,ab)) OR ("life cycle assessment"/ OR "environmental impact assessment"/ OR "life cycle assess*".mp OR "life cycle assessment".mp OR "life cycle inventory".mp OR "LCA".mp OR "LCAs".mp OR "life cycle inventory".mp OR "life cycle inventories".mp))

**Web of Science**

**UV4.1**

(TS=("Closed-Circuit Anesthesia" OR "Endotracheal Anesthesia" OR "Closed-Circuit Anaesthesia" OR "Endotracheal Anaesthesia" OR "Halothane" OR "Isoflurane" OR "Methoxyflurane" OR "Sevoflurane" OR "Anaesthetic gas" OR "Anaesthetic gases" OR "Anesthetic gas" OR "Anesthetic gases" OR "Desflurane" OR "Enflurane" OR "Flurane" OR "Fluranes" OR "Gaseous anaesthesia" OR "Gaseous anaesthetic" OR "Gaseous anaesthetics" OR "Gaseous anesthesia" OR "Gaseous anesthetic" OR "Gaseous anesthetics" OR "Halothane" OR "Inhalation anaesthesia" OR "inhalation anaesthetic" OR "inhalation anaesthetics" OR "Inhalation anesthesia" OR "inhalation anesthetic" OR "inhalation anesthetics" OR "Inhalational agents" OR "Inhalational anaesthesia" OR "Inhalational anaesthetic" OR "Inhalational anaesthetic agent" OR "Inhalational anaesthetic agents" OR "Inhalational anaesthetics" OR "Inhalational anesthesia" OR "Inhalational anesthetic" OR "Inhalational anesthetic agent" OR "Inhalational anesthetic agents" OR "Inhalational anesthetics" OR "Inhaled anaesthetic" OR "Inhaled anaesthetic agent" OR "Inhaled anaesthetic agents" OR "Inhaled anaesthetics" OR "Inhaled anesthetic" OR "Inhaled anesthetic agents" OR "Inhaled anesthetics" OR "insufflation anaesthesia" OR "insufflation anaesthetic" OR "insufflation anaesthetics" OR "insufflation anesthesia" OR "insufflation anesthetic" OR "insufflation anesthetics" OR "Isoflurane" OR "Methoxyflurane" OR "Sevoflurane" OR "Vapor anaesthesia" OR "Vapor anaesthetic" OR "Vapor anaesthetics" OR "Vapor anesthesia" OR "Vapor anesthetic" OR "Vapor anesthetics" OR "Volatile agent" OR "Volatile agents" OR "Volatile Anaesthetic" OR "Volatile Anaesthetics" OR "Volatile anesthetic" OR "Volatile anesthetics" OR "Volatile drug" OR "Volatile drugs" OR "Volatile fluorinated liquid" OR "Volatile fluorinated liquids" OR "Volatile gas" OR "Volatile gases" OR "Volatile liquid agent" OR "Volatile liquid agents" OR "Anesthetics, Inhalation" OR "Anesthetic Gas" OR "Anesthetic Gases" OR "Inhalation Anaesthetic" OR "Inhalation Anaesthetics" OR "Inhalation Anesthetic" OR "Inhalation Anesthetics") AND TS=("Anesthesia, Intravenous" OR "Anesthetics, Intravenous" OR "Anesthetics, Intravenous" OR "Intravenous Anesthetics" OR "Intravenous Anaesthetics" OR "Intravenous Anesthetic" OR "Intravenous Anaesthetic" OR "Intravenous Anesthesia" OR "Intravenous Anaesthesia" OR "Dissociative Anesthetics" OR "Dissociative Anaesthetics" OR "Dissociative Anesthetic" OR "Dissociative Anaesthetic" OR "Dissociative Anesthesia" OR "Dissociative Anaesthesia" OR "2-(3-methoxyphenyl)-2-(ethylamino)cyclohexanone"OR "2-Oxo-PCE"OR "Alfentanil" OR "Chloralose" OR "Diazepam" OR "Etomidate" OR "Fentanyl" OR "Ketamine" OR "Methohexital" OR "Midazolam" OR "Propanidid" OR "Propofol" OR "Sodium Oxybate" OR "Sufentanil" OR "Thiamylal" OR "Thiopental" OR "Tiletamine" OR "Urethane" OR "2-(3-methoxyphenyl)-2-(ethylamino)cyclohexanone" OR "2-Oxo-PCE" OR "Alfentanil" OR "Chloralose" OR "Diazepam" OR "Etomidate" OR "Fentanyl" OR "intravenous anaesthesia" OR "intravenous anaesthesias" OR "Intravenous anaesthetic" OR "Intravenous anaesthetic agent" OR "Intravenous anaesthetic agents" OR "Intravenous anaesthetic drug" OR "Intravenous anaesthetic drugs" OR "Intravenous anaesthetics" OR "intravenous anesthesia" OR "intravenous anesthesias" OR "intravenous anesthetic" OR "Intravenous anesthetic agents" OR "Intravenous anesthetic agents" OR "Intravenous anesthetic drug" OR "Intravenous anesthetic drugs" OR "intravenous anesthetics" OR "IV anaesthesia" OR "IV anaesthetics" OR "IV anaesthetics" OR "IV anesthesia" OR "IV anesthetic" OR "IV anesthetics" OR "Ketamine" OR "Methohexital" OR "Midazolam" OR "Propanidid" OR "Propofol" OR "Sodium Oxybate" OR "Sufentanil" OR "Thiamylal" OR "Thiopental" OR "Tiletamine" OR "TIVA" OR "Total intravenous anaesthesia" OR "Total Intravenous anesthesia" OR "Urethane")) AND (TS=("Carbon Footprint" OR "carbon footprint" OR "carbon footprint*" OR "Climate Change" OR "climate change" OR "CO2 emission" OR "CO2 emissions" OR "CO2 footprint" OR "CO2 footprint*" OR "environmental protection" OR "conservation of natural resources" OR "environmental protection" OR "Disposable Equipment" OR "Disposables" OR "Disposable" OR "ecological footprint" OR "ecological footprint*" OR "ecological sustainability" OR "environmental impact" OR "environmental impact" OR "environmental impact*" OR "environmental impacts" OR "environmental pollut*" OR "pollution" OR "environmental pollution" OR "environmental sustainab*" OR "environmental sustainability" OR "environmental sustainability" OR "Global Warming" OR "Global Warming" OR "Greenhouse Effect" OR "greenhouse effect*" OR "greenhouse effects" OR "greenhouse gas emission" OR "greenhouse gas emissions" OR "Greenhouse Gas" OR "greening" OR "hospital waste" OR "life cycle assessment" OR "environmental impact assessment" OR "life cycle assess*" OR "life cycle assessment" OR "LCA" OR "LCAs" OR "life cycle inventory" OR "life cycle inventories" OR "Waste Disposal" OR "Hospital Waste" OR "medical waste" OR "Rising Sea Level" OR "Rising Sea Levels" OR "Sea Level Rise" OR "Sea Level Rise" OR "Waste Disposal" OR "waste water recycling" OR "Recycling" OR "recycling" OR "recycle*" OR "Equipment reuse" OR "Reusables" OR "reusable" OR "reuse" OR "reused" OR "reusing" OR "Waste Disposal" OR "Waste Management" OR "Plastic overuse" OR "Green surgery" OR "Emission reduction" OR "Emission reduction strategy" OR "air pollution control" OR "air pollution control" OR "acidification" OR "soil acidification" OR "ocean acidification" OR "acidification" OR "Acidification potential" OR "AP in kg SO2 equivalents" OR "eco-efficiency" OR "ecoefficiency" OR "eco-efficien*" OR "ecoefficien*" OR "ecotoxicity" OR "ecotoxicity" OR "ecotoxic*" OR "eco toxicity" OR "eco toxic*" OR "EP in kg PO4 equivalent" OR "Eutrophication" OR "eutrophication" OR "Eutrophication potential" OR "FAETP in kg DCB equivalent" OR "Freshwater Aquatic Ecotoxicity Potential" OR "GWP in kg CO2 equivalents" OR "H+ moles equivalents" OR "HTTP in kg Dichlorobenzene equivalent" OR "Human Toxicity Potential" OR "kg 2.4-D equivalents" OR "kg CFC-11 equivalent" OR "kg N equivalents" OR "kg NOx equivalents" OR "life cycle analysis" OR "ozone depletion" OR "Photochemical Ozone Depletion Potential" OR "POCP in kg ethane equivalent" OR "smog" OR "Waste" OR "waste" OR "wastes" OR "Ozone Depletion" OR "Smog" OR "Equipment reuse" OR "Greenhouse Gases" OR "Greenhouse Gas" OR "SO2 equiv*" OR "CO2 equiva*" OR "CFC-11 equiv*" OR "N equiv*" OR "Sustainable Development" OR "Sustainable Development" OR "Biodiversity" OR "Climatic change" OR "Green deal" OR "preservation of natural resources" OR "Refuse Disposal" OR "Waste Water" OR "Wastewater" OR "Water Purification" OR (("plastic*" OR "microplastic*") AND ("soup" OR "pollution" OR "overuse" OR "contamination")) OR (("Plastic" OR "plastics") AND "overuse") OR ("hydrogen*" AND "moles" AND "equiv*") OR ("Dichlorobenzen*" AND "equiv*") OR ("2,4-D" AND "equiv*") OR ("NOx" AND "equiv*") OR ("ethane" AND "equiv*") OR ("PO4" AND "equiv*") OR ("DCB" AND "equiv*") OR ("sustainability" AND ("environment*" OR "carbon")) OR (("Carbon Dioxide" OR "Carbon Dioxide" OR "CO2") AND ("pollution" OR "emission" OR "emissions" OR "waste" OR "environment" OR "environmental*" OR "footprint" OR "footprint*" OR "sustainable" OR "hazard" OR "hazard*"))) OR TI=("environmental*" OR "sustainab*")) NOT DT=(meeting abstract) AND (PY=(2000 OR 2001 OR 2002 OR 2003 OR 2004 OR 2005 OR 2006 OR 2007 OR 2008 OR 2009 OR 2010 OR 2011 OR 2012 OR 2013 OR 2014 OR 2015 OR 2016 OR 2017 OR 2018 OR 2019 OR 2020 OR 2021 OR 2022)) AND (TI=(*("Meta-Analysis" OR metaanaly* OR "meta-analy*" or metanaly* OR "Systematic Review" OR "Cochrane Database Syst Rev" OR "prisma" OR "preferred reporting items" OR "prospero" OR ((systemati* OR scoping OR umbrella OR "structured literature") NEAR/4 (review* OR overview*)) OR "systematic review*" OR "scoping review*" OR "umbrella review*" OR "structured literature review*" OR "systematic qualitative review*" OR "systematic quantitative review*" OR "systematic search and review" OR "systematized review" OR "systematised review" OR "systemic review" OR "systematic literature review*" OR "systematic integrative literature review*" OR "systematically review*" OR "scoping literature review*" OR "systematic critical review" OR "systematic integrative review*" OR "systematic evidence review" OR "Systematic integrative literature review*" OR "Systematic mixed studies review*" OR "Systematized literature review*" OR "Systematic overview*" OR "Systematic narrative review*" OR (("systemati*" OR "literature" OR "database*" OR "data-base*" OR "structured" OR "comprehensive*" OR "systemic*") NEAR/4 "search*") OR ("Literature" AND "review" AND ("database*" OR "data-base*" OR "search*")) OR (("data extraction" OR "data source*") AND "study selection") OR ("search strategy" AND "selection criteria") OR ("data source*" AND "data synthesis") OR "medline" OR "pubmed" OR "embase" OR "Cochrane" OR (("critical" OR "rapid") NEAR/4 ("review*" OR "overview*" OR "synthes*")) OR ((("critical*" OR "rapid*") NEAR/4 ("review*" OR "overview*" OR "synthes*") NEAR/4 ("search*" OR "database*" OR "data-base*"))) OR metasynthes* OR "meta-synthes*")* OR ("Randomized Controlled Trial" OR random* OR "RCT"OR "RCTs" OR "pragmatic clinical trial*" OR "practical clinical trial*" OR "non-inferiority trial*" OR "noninferiority trial*" OR "superiority trial*" OR "equivalence clinical trial*") OR ("Comparative Study" OR "comparison" OR "comparative" OR "compar*" OR "major clinical study" OR "clinical study" OR "case control study" OR "family study" OR "longitudinal study" OR "retrospective study" OR "prospective study" OR "cohort analysis" OR cohort* OR (("case control" NEAR/1 (study OR studies))) OR (("follow up" NEAR/1 (study OR studies))) OR (observational NEAR/1 (study OR studies)) OR ((epidemiologic NEAR/1 (study OR studies))) OR (("cross sectional" NEAR/1 (study OR studies)))) OR ("life cycle assess*" OR "life cycle assessment" OR "life cycle inventory" OR "LCA" OR "LCAs" OR "life cycle inventory" OR "life cycle inventories")) OR AB=(*("Meta-Analysis" OR metaanaly* OR "meta-analy*" or metanaly* OR "Systematic Review" OR "Cochrane Database Syst Rev" OR "prisma" OR "preferred reporting items" OR "prospero" OR ((systemati* OR scoping OR umbrella OR "structured literature") NEAR/4 (review* OR overview*)) OR "systematic review*" OR "scoping review*" OR "umbrella review*" OR "structured literature review*" OR "systematic qualitative review*" OR "systematic quantitative review*" OR "systematic search and review" OR "systematized review" OR "systematised review" OR "systemic review" OR "systematic literature review*" OR "systematic integrative literature review*" OR "systematically review*" OR "scoping literature review*" OR "systematic critical review" OR "systematic integrative review*" OR "systematic evidence review" OR "Systematic integrative literature review*" OR "Systematic mixed studies review*" OR "Systematized literature review*" OR "Systematic overview*" OR "Systematic narrative review*" OR (("systemati*" OR "literature" OR "database*" OR "data-base*" OR "structured" OR "comprehensive*" OR "systemic*") NEAR/4 "search*") OR ("Literature" AND "review" AND ("database*" OR "data-base*" OR "search*")) OR (("data extraction" OR "data source*") AND "study selection") OR ("search strategy" AND "selection criteria") OR ("data source*" AND "data synthesis") OR "medline" OR "pubmed" OR "embase" OR "Cochrane" OR (("critical" OR "rapid") NEAR/4 ("review*" OR "overview*" OR "synthes*")) OR ((("critical*" OR "rapid*") NEAR/4 ("review*" OR "overview*" OR "synthes*") NEAR/4 ("search*" OR "database*" OR "data-base*"))) OR metasynthes* OR "meta-synthes*")* OR ("Randomized Controlled Trial" OR random* OR "RCT"OR "RCTs" OR "pragmatic clinical trial*" OR "practical clinical trial*" OR "non-inferiority trial*" OR "noninferiority trial*" OR "superiority trial*" OR "equivalence clinical trial*") OR ("Comparative Study" OR "comparison" OR "comparative" OR "compar*" OR "major clinical study" OR "clinical study" OR "case control study" OR "family study" OR "longitudinal study" OR "retrospective study" OR "prospective study" OR "cohort analysis" OR cohort* OR (("case control" NEAR/1 (study OR studies))) OR (("follow up" NEAR/1 (study OR studies))) OR (observational NEAR/1 (study OR studies)) OR ((epidemiologic NEAR/1 (study OR studies))) OR (("cross sectional" NEAR/1 (study OR studies)))) OR ("life cycle assess*" OR "life cycle assessment" OR "life cycle inventory" OR "LCA" OR "LCAs" OR "life cycle inventory" OR "life cycle inventories"))**)**

**UV4.2**

(TS=("Closed-Circuit Anesthesia" OR "Endotracheal Anesthesia" OR "Closed-Circuit Anaesthesia" OR "Endotracheal Anaesthesia" OR "Halothane" OR "Isoflurane" OR "Methoxyflurane" OR "Sevoflurane" OR "Anaesthetic gas" OR "Anaesthetic gases" OR "Anesthetic gas" OR "Anesthetic gases" OR "Desflurane" OR "Enflurane" OR "Flurane" OR "Fluranes" OR "Gaseous anaesthesia" OR "Gaseous anaesthetic" OR "Gaseous anaesthetics" OR "Gaseous anesthesia" OR "Gaseous anesthetic" OR "Gaseous anesthetics" OR "Halothane" OR "Inhalation anaesthesia" OR "inhalation anaesthetic" OR "inhalation anaesthetics" OR "Inhalation anesthesia" OR "inhalation anesthetic" OR "inhalation anesthetics" OR "Inhalational agents" OR "Inhalational anaesthesia" OR "Inhalational anaesthetic" OR "Inhalational anaesthetic agent" OR "Inhalational anaesthetic agents" OR "Inhalational anaesthetics" OR "Inhalational anesthesia" OR "Inhalational anesthetic" OR "Inhalational anesthetic agent" OR "Inhalational anesthetic agents" OR "Inhalational anesthetics" OR "Inhaled anaesthetic" OR "Inhaled anaesthetic agent" OR "Inhaled anaesthetic agents" OR "Inhaled anaesthetics" OR "Inhaled anesthetic" OR "Inhaled anesthetic agents" OR "Inhaled anesthetics" OR "insufflation anaesthesia" OR "insufflation anaesthetic" OR "insufflation anaesthetics" OR "insufflation anesthesia" OR "insufflation anesthetic" OR "insufflation anesthetics" OR "Isoflurane" OR "Methoxyflurane" OR "Sevoflurane" OR "Vapor anaesthesia" OR "Vapor anaesthetic" OR "Vapor anaesthetics" OR "Vapor anesthesia" OR "Vapor anesthetic" OR "Vapor anesthetics" OR "Volatile agent" OR "Volatile agents" OR "Volatile Anaesthetic" OR "Volatile Anaesthetics" OR "Volatile anesthetic" OR "Volatile anesthetics" OR "Volatile drug" OR "Volatile drugs" OR "Volatile fluorinated liquid" OR "Volatile fluorinated liquids" OR "Volatile gas" OR "Volatile gases" OR "Volatile liquid agent" OR "Volatile liquid agents" OR "Anesthetics, Inhalation" OR "Anesthetic Gas" OR "Anesthetic Gases" OR "Inhalation Anaesthetic" OR "Inhalation Anaesthetics" OR "Inhalation Anesthetic" OR "Inhalation Anesthetics") AND TS=("scavenging system" OR "anaesthetic gas scavenging system" OR "anesthetic gas scavenging system" OR "Contrafluran" OR "gas extract*" OR "gas extraction" OR "gas extraction system" OR "gas extraction systems" OR "gas scaveng*" OR "gas scavenger" OR "gas scavengers" OR "gas scavenging " OR "scavenging device" OR "scavenging device" OR "scavenging devices" OR "scavenging system" OR "scavenging systems" OR "vapour captur*" OR "vapour capture" OR "Vapour Capture Technology" OR "vapour recycl*" OR "vapour recycling technology" OR "vapour recycling")) AND (TS=("Carbon Footprint" OR "carbon footprint" OR "carbon footprint*" OR "Climate Change" OR "climate change" OR "CO2 emission" OR "CO2 emissions" OR "CO2 footprint" OR "CO2 footprint*" OR "environmental protection" OR "conservation of natural resources" OR "environmental protection" OR "Disposable Equipment" OR "Disposables" OR "Disposable" OR "ecological footprint" OR "ecological footprint*" OR "ecological sustainability" OR "environmental impact" OR "environmental impact" OR "environmental impact*" OR "environmental impacts" OR "environmental pollut*" OR "pollution" OR "environmental pollution" OR "environmental sustainab*" OR "environmental sustainability" OR "environmental sustainability" OR "Global Warming" OR "Global Warming" OR "Greenhouse Effect" OR "greenhouse effect*" OR "greenhouse effects" OR "greenhouse gas emission" OR "greenhouse gas emissions" OR "Greenhouse Gas" OR "greening" OR "hospital waste" OR "life cycle assessment" OR "environmental impact assessment" OR "life cycle assess*" OR "life cycle assessment" OR "LCA" OR "LCAs" OR "life cycle inventory" OR "life cycle inventories" OR "Waste Disposal" OR "Hospital Waste" OR "medical waste" OR "Rising Sea Level" OR "Rising Sea Levels" OR "Sea Level Rise" OR "Sea Level Rise" OR "Waste Disposal" OR "waste water recycling" OR "Recycling" OR "recycling" OR "recycle*" OR "Equipment reuse" OR "Reusables" OR "reusable" OR "reuse" OR "reused" OR "reusing" OR "Waste Disposal" OR "Waste Management" OR "Plastic overuse" OR "Green surgery" OR "Emission reduction" OR "Emission reduction strategy" OR "air pollution control" OR "air pollution control" OR "acidification" OR "soil acidification" OR "ocean acidification" OR "acidification" OR "Acidification potential" OR "AP in kg SO2 equivalents" OR "eco-efficiency" OR "ecoefficiency" OR "eco-efficien*" OR "ecoefficien*" OR "ecotoxicity" OR "ecotoxicity" OR "ecotoxic*" OR "eco toxicity" OR "eco toxic*" OR "EP in kg PO4 equivalent" OR "Eutrophication" OR "eutrophication" OR "Eutrophication potential" OR "FAETP in kg DCB equivalent" OR "Freshwater Aquatic Ecotoxicity Potential" OR "GWP in kg CO2 equivalents" OR "H+ moles equivalents" OR "HTTP in kg Dichlorobenzene equivalent" OR "Human Toxicity Potential" OR "kg 2.4-D equivalents" OR "kg CFC-11 equivalent" OR "kg N equivalents" OR "kg NOx equivalents" OR "life cycle analysis" OR "ozone depletion" OR "Photochemical Ozone Depletion Potential" OR "POCP in kg ethane equivalent" OR "smog" OR "Waste" OR "waste" OR "wastes" OR "Ozone Depletion" OR "Smog" OR "Equipment reuse" OR "Greenhouse Gases" OR "Greenhouse Gas" OR "SO2 equiv*" OR "CO2 equiva*" OR "CFC-11 equiv*" OR "N equiv*" OR "Sustainable Development" OR "Sustainable Development" OR "Biodiversity" OR "Climatic change" OR "Green deal" OR "preservation of natural resources" OR "Refuse Disposal" OR "Waste Water" OR "Wastewater" OR "Water Purification" OR (("plastic*" OR "microplastic*") AND ("soup" OR "pollution" OR "overuse" OR "contamination")) OR (("Plastic" OR "plastics") AND "overuse") OR ("hydrogen*" AND "moles" AND "equiv*") OR ("Dichlorobenzen*" AND "equiv*") OR ("2,4-D" AND "equiv*") OR ("NOx" AND "equiv*") OR ("ethane" AND "equiv*") OR ("PO4" AND "equiv*") OR ("DCB" AND "equiv*") OR ("sustainability" AND ("environment*" OR "carbon")) OR (("Carbon Dioxide" OR "Carbon Dioxide" OR "CO2") AND ("pollution" OR "emission" OR "emissions" OR "waste" OR "environment" OR "environmental*" OR "footprint" OR "footprint*" OR "sustainable" OR "hazard" OR "hazard*"))) OR TI=("environmental*" OR "sustainab*")) NOT DT=(meeting abstract) AND (PY=(2000 OR 2001 OR 2002 OR 2003 OR 2004 OR 2005 OR 2006 OR 2007 OR 2008 OR 2009 OR 2010 OR 2011 OR 2012 OR 2013 OR 2014 OR 2015 OR 2016 OR 2017 OR 2018 OR 2019 OR 2020 OR 2021 OR 2022)) AND (TI=(*("Meta-Analysis" OR metaanaly* OR "meta-analy*" or metanaly* OR "Systematic Review" OR "Cochrane Database Syst Rev" OR "prisma" OR "preferred reporting items" OR "prospero" OR ((systemati* OR scoping OR umbrella OR "structured literature") NEAR4 (review* OR overview*)) OR "systematic review*" OR "scoping review*" OR "umbrella review*" OR "structured literature review*" OR "systematic qualitative review*" OR "systematic quantitative review*" OR "systematic search and review" OR "systematized review" OR "systematised review" OR "systemic review" OR "systematic literature review*" OR "systematic integrative literature review*" OR "systematically review*" OR "scoping literature review*" OR "systematic critical review" OR "systematic integrative review*" OR "systematic evidence review" OR "Systematic integrative literature review*" OR "Systematic mixed studies review*" OR "Systematized literature review*" OR "Systematic overview*" OR "Systematic narrative review*" OR (("systemati*" OR "literature" OR "database*" OR "data-base*" OR "structured" OR "comprehensive*" OR "systemic*") NEAR4 "search*") OR ("Literature" AND "review" AND ("database*" OR "data-base*" OR "search*")) OR (("data extraction" OR "data source*") AND "study selection") OR ("search strategy" AND "selection criteria") OR ("data source*" AND "data synthesis") OR "medline" OR "pubmed" OR "embase" OR "Cochrane" OR (("critical" OR "rapid") NEAR4 ("review*" OR "overview*" OR "synthes*")) OR ((("critical*" OR "rapid*") NEAR4 ("review*" OR "overview*" OR "synthes*") NEAR4 ("search*" OR "database*" OR "data-base*"))) OR metasynthes* OR "meta-synthes*")* OR ("Randomized Controlled Trial" OR random* OR "RCT"OR "RCTs" OR "pragmatic clinical trial*" OR "practical clinical trial*" OR "non-inferiority trial*" OR "noninferiority trial*" OR "superiority trial*" OR "equivalence clinical trial*") OR ("Comparative Study" OR "comparison" OR "comparative" OR "compar*" OR "major clinical study" OR "clinical study" OR "case control study" OR "family study" OR "longitudinal study" OR "retrospective study" OR "prospective study" OR "cohort analysis" OR cohort* OR (("case control" NEAR1 (study OR studies))) OR (("follow up" NEAR1 (study OR studies))) OR (observational NEAR1 (study OR studies)) OR ((epidemiologic NEAR1 (study OR studies))) OR (("cross sectional" NEAR1 (study OR studies)))) OR ("life cycle assess*" OR "life cycle assessment" OR "life cycle inventory" OR "LCA" OR "LCAs" OR "life cycle inventory" OR "life cycle inventories")) OR AB=(*("Meta-Analysis" OR metaanaly* OR "meta-analy*" or metanaly* OR "Systematic Review" OR "Cochrane Database Syst Rev" OR "prisma" OR "preferred reporting items" OR "prospero" OR ((systemati* OR scoping OR umbrella OR "structured literature") NEAR4 (review* OR overview*)) OR "systematic review*" OR "scoping review*" OR "umbrella review*" OR "structured literature review*" OR "systematic qualitative review*" OR "systematic quantitative review*" OR "systematic search and review" OR "systematized review" OR "systematised review" OR "systemic review" OR "systematic literature review*" OR "systematic integrative literature review*" OR "systematically review*" OR "scoping literature review*" OR "systematic critical review" OR "systematic integrative review*" OR "systematic evidence review" OR "Systematic integrative literature review*" OR "Systematic mixed studies review*" OR "Systematized literature review*" OR "Systematic overview*" OR "Systematic narrative review*" OR (("systemati*" OR "literature" OR "database*" OR "data-base*" OR "structured" OR "comprehensive*" OR "systemic*") NEAR4 "search*") OR ("Literature" AND "review" AND ("database*" OR "data-base*" OR "search*")) OR (("data extraction" OR "data source*") AND "study selection") OR ("search strategy" AND "selection criteria") OR ("data source*" AND "data synthesis") OR "medline" OR "pubmed" OR "embase" OR "Cochrane" OR (("critical" OR "rapid") NEAR4 ("review*" OR "overview*" OR "synthes*")) OR ((("critical*" OR "rapid*") NEAR4 ("review*" OR "overview*" OR "synthes*") NEAR4 ("search*" OR "database*" OR "data-base*"))) OR metasynthes* OR "meta-synthes*")* OR ("Randomized Controlled Trial" OR random* OR "RCT"OR "RCTs" OR "pragmatic clinical trial*" OR "practical clinical trial*" OR "non-inferiority trial*" OR "noninferiority trial*" OR "superiority trial*" OR "equivalence clinical trial*") OR ("Comparative Study" OR "comparison" OR "comparative" OR "compar*" OR "major clinical study" OR "clinical study" OR "case control study" OR "family study" OR "longitudinal study" OR "retrospective study" OR "prospective study" OR "cohort analysis" OR cohort* OR (("case control" NEAR1 (study OR studies))) OR (("follow up" NEAR1 (study OR studies))) OR (observational NEAR1 (study OR studies)) OR ((epidemiologic NEAR1 (study OR studies))) OR (("cross sectional" NEAR1 (study OR studies)))) OR ("life cycle assess*" OR "life cycle assessment" OR "life cycle inventory" OR "LCA" OR "LCAs" OR "life cycle inventory" OR "life cycle inventories"))**)**

**UV4.3**

TS=(("Local Anesthesia" OR "local anesthetic agent" OR "Nerve Block" OR "Local Anesthesia" OR "Local Anaesthesia" OR "Locoregional Anesthesia" OR "Locoregional Anaesthesia" OR "Local Anesthetics" OR "Local Anaesthetics" OR "Locoregional Anesthetics" OR "Locoregional Anaesthetics" OR "Local Anesthetic" OR "Local Anaesthetic" OR "Locoregional Anesthetic" OR "Locoregional Anaesthetic" OR "axillary block" OR "axillary block*" OR "Bier block" OR "Bier block*" OR "Bier's block" OR "Bier's block*" OR "brachial plexus block" OR "brachial plexus block*" OR "combined spinal epidural" OR "combined spinal epidural block" OR "combined spinal epidural block*" OR "epidural anaesthesia" OR "epidural anesthesia" OR "intravenous regional anesthaesia" OR "intravenous regional anesthesia" OR "ischiadic block" OR "ischiadic block*" OR "local infiltration" OR "lower extremity block" OR "lower extremity block*" OR "nerve block" OR "nerve block" OR "nerve block*" OR "nerve block*" OR "neuraxial anaesthesia" OR "neuraxial anesthesia" OR "neuraxial block" OR "neuraxial block*" OR "neuraxial technique" OR "neuraxial techniques" OR "peripheral nerve block" OR "peripheral nerve block*" OR "plexus block" OR "plexus block*" OR "plexus nerve block" OR "plexus nerve block*" OR "popliteal block" OR "popliteal block*" OR "regional anaesthesia" OR "regional anesthesia" OR "spinal anaesthesia" OR "spinal anaesthetic block" OR "spinal anaesthetic block*" OR "spinal anesthesia" OR "spinal anesthetic block" OR "spinal anesthetic block*" OR "supraclavicular block" OR "supraclavicular block*" OR "upper extremity block" OR "upper extremity block*") AND ("General Anesthesia" OR "general anesthesia" OR "general anaesthesia" OR "general anesthe*" OR "general anaesthe*" OR "inhalation anesthetic agent" OR "inhalation anesthesia" OR "Closed Circuit Anesthesia" OR "Endotracheal Anesthesia" OR "Closed Circuit Anaesthesia" OR "Endotracheal Anaesthesia" OR "Halothane" OR "Isoflurane" OR "Methoxyflurane" OR "Sevoflurane" OR "Anaesthetic gas" OR "Anaesthetic gases" OR "Anesthetic gas" OR "Anesthetic gases" OR "Desflurane" OR "Enflurane" OR "Flurane" OR "Fluranes" OR "Gaseous anaesthesia" OR "Gaseous anaesthetic" OR "Gaseous anaesthetics" OR "Gaseous anesthesia" OR "Gaseous anesthetic" OR "Gaseous anesthetics" OR "Halothane" OR "Inhalation anaesthesia" OR "inhalation anaesthetic" OR "inhalation anaesthetics" OR "Inhalation anesthesia" OR "inhalation anesthetic" OR "inhalation anesthetics" OR "Inhalational agents" OR "Inhalational anaesthesia" OR "Inhalational anaesthetic" OR "Inhalational anaesthetic agent" OR "Inhalational anaesthetic agents" OR "Inhalational anaesthetics" OR "Inhalational anesthesia" OR "Inhalational anesthetic" OR "Inhalational anesthetic agent" OR "Inhalational anesthetic agents" OR "Inhalational anesthetics" OR "Inhaled anaesthetic" OR "Inhaled anaesthetic agent" OR "Inhaled anaesthetic agents" OR "Inhaled anaesthetics" OR "Inhaled anesthetic" OR "Inhaled anesthetic agents" OR "Inhaled anesthetics" OR "insufflation anaesthesia" OR "insufflation anaesthetic" OR "insufflation anaesthetics" OR "insufflation anesthesia" OR "insufflation anesthetic" OR "insufflation anesthetics" OR "Isoflurane" OR "Methoxyflurane" OR "Sevoflurane" OR "Vapor anaesthesia" OR "Vapor anaesthetic" OR "Vapor anaesthetics" OR "Vapor anesthesia" OR "Vapor anesthetic" OR "Vapor anesthetics" OR "Volatile agent" OR "Volatile agents" OR "Volatile Anaesthetic" OR "Volatile Anaesthetics" OR "Volatile anesthetic" OR "Volatile anesthetics" OR "Volatile drug" OR "Volatile drugs" OR "Volatile fluorinated liquid" OR "Volatile fluorinated liquids" OR "Volatile gas" OR "Volatile gases" OR "Volatile liquid agent" OR "Volatile liquid agents" OR "Anesthetic Gas" OR "Anesthetic Gases" OR "Inhalation Anaesthetic" OR "Inhalation Anaesthetics" OR "Inhalation Anesthetic" OR "Inhalation Anesthetics" OR "intravenous anesthetic agent" OR "Intravenous Anesthesia" OR "Intravenous Anesthetics" OR "Intravenous Anaesthetics" OR "Intravenous Anesthetic" OR "Intravenous Anaesthetic" OR "Intravenous Anesthesia" OR "Intravenous Anaesthesia" OR "Dissociative Anesthetics" OR "Dissociative Anaesthetics" OR "Dissociative Anesthetic" OR "Dissociative Anaesthetic" OR "Dissociative Anesthesia" OR "Dissociative Anaesthesia" OR "2 (3 methoxyphenyl) 2 (ethylamino)cyclohexanone" OR "2 Oxo PCE" OR "Alfentanil" OR "Chloralose" OR "Diazepam" OR "Etomidate" OR "Fentanyl" OR "Ketamine" OR "Methohexital" OR "Midazolam" OR "Propanidid" OR "Propofol" OR "Sodium Oxybate" OR "Sufentanil" OR "Thiamylal" OR "Thiopental" OR "Tiletamine" OR "Urethane" OR "2 (3 methoxyphenyl) 2 (ethylamino)cyclohexanone" OR "2 Oxo PCE" OR "Alfentanil" OR "Chloralose" OR "Diazepam" OR "Etomidate" OR "Fentanyl" OR "intravenous anaesthesia" OR "intravenous anaesthesias" OR "Intravenous anaesthetic" OR "Intravenous anaesthetic agent" OR "Intravenous anaesthetic agents" OR "Intravenous anaesthetic drug" OR "Intravenous anaesthetic drugs" OR "Intravenous anaesthetics" OR "intravenous anesthesia" OR "intravenous anesthesias" OR "intravenous anesthetic" OR "Intravenous anesthetic agents" OR "Intravenous anesthetic agents" OR "Intravenous anesthetic drug" OR "Intravenous anesthetic drugs" OR "intravenous anesthetics" OR "IV anaesthesia" OR "IV anaesthetics" OR "IV anaesthetics" OR "IV anesthesia" OR "IV anesthetic" OR "IV anesthetics" OR "Ketamine" OR "Methohexital" OR "Midazolam" OR "Propanidid" OR "Propofol" OR "Sodium Oxybate" OR "Sufentanil" OR "Thiamylal" OR "Thiopental" OR "Tiletamine" OR "TIVA" OR "Total intravenous anaesthesia" OR "Total Intravenous anesthesia" OR "Urethane")) AND (TS=("Carbon Footprint" OR "carbon footprint" OR "carbon footprint*" OR "Climate Change" OR "climate change" OR "CO2 emission" OR "CO2 emissions" OR "CO2 footprint" OR "CO2 footprint*" OR "environmental protection" OR "conservation of natural resources" OR "environmental protection" OR "Disposable Equipment" OR "Disposables" OR "Disposable" OR "ecological footprint" OR "ecological footprint*" OR "ecological sustainability" OR "environmental impact" OR "environmental impact" OR "environmental impact*" OR "environmental impacts" OR "environmental pollut*" OR "pollution" OR "environmental pollution" OR "environmental sustainab*" OR "environmental sustainability" OR "environmental sustainability" OR "Global Warming" OR "Global Warming" OR "Greenhouse Effect" OR "greenhouse effect*" OR "greenhouse effects" OR "greenhouse gas emission" OR "greenhouse gas emissions" OR "Greenhouse Gas" OR "greening" OR "hospital waste" OR "life cycle assessment" OR "environmental impact assessment" OR "life cycle assess*" OR "life cycle assessment" OR "LCA" OR "LCAs" OR "life cycle inventory" OR "life cycle inventories" OR "Waste Disposal" OR "Hospital Waste" OR "medical waste" OR "Rising Sea Level" OR "Rising Sea Levels" OR "Sea Level Rise" OR "Sea Level Rise" OR "Waste Disposal" OR "waste water recycling" OR "Recycling" OR "recycling" OR "recycle*" OR "Equipment reuse" OR "Reusables" OR "reusable" OR "reuse" OR "reused" OR "reusing" OR "Waste Disposal" OR "Waste Management" OR "Plastic overuse" OR "Green surgery" OR "Emission reduction" OR "Emission reduction strategy" OR "air pollution control" OR "air pollution control" OR "acidification" OR "soil acidification" OR "ocean acidification" OR "acidification" OR "Acidification potential" OR "AP in kg SO2 equivalents" OR "eco-efficiency" OR "ecoefficiency" OR "eco-efficien*" OR "ecoefficien*" OR "ecotoxicity" OR "ecotoxicity" OR "ecotoxic*" OR "eco toxicity" OR "eco toxic*" OR "EP in kg PO4 equivalent" OR "Eutrophication" OR "eutrophication" OR "Eutrophication potential" OR "FAETP in kg DCB equivalent" OR "Freshwater Aquatic Ecotoxicity Potential" OR "GWP in kg CO2 equivalents" OR "H+ moles equivalents" OR "HTTP in kg Dichlorobenzene equivalent" OR "Human Toxicity Potential" OR "kg 2.4-D equivalents" OR "kg CFC-11 equivalent" OR "kg N equivalents" OR "kg NOx equivalents" OR "life cycle analysis" OR "ozone depletion" OR "Photochemical Ozone Depletion Potential" OR "POCP in kg ethane equivalent" OR "smog" OR "Waste" OR "waste" OR "wastes" OR "Ozone Depletion" OR "Smog" OR "Equipment reuse" OR "Greenhouse Gases" OR "Greenhouse Gas" OR "SO2 equiv*" OR "CO2 equiva*" OR "CFC-11 equiv*" OR "N equiv*" OR "Sustainable Development" OR "Sustainable Development" OR "Biodiversity" OR "Climatic change" OR "Green deal" OR "preservation of natural resources" OR "Refuse Disposal" OR "Waste Water" OR "Wastewater" OR "Water Purification" OR (("plastic*" OR "microplastic*") AND ("soup" OR "pollution" OR "overuse" OR "contamination")) OR (("Plastic" OR "plastics") AND "overuse") OR ("hydrogen*" AND "moles" AND "equiv*") OR ("Dichlorobenzen*" AND "equiv*") OR ("2,4-D" AND "equiv*") OR ("NOx" AND "equiv*") OR ("ethane" AND "equiv*") OR ("PO4" AND "equiv*") OR ("DCB" AND "equiv*") OR ("sustainability" AND ("environment*" OR "carbon")) OR (("Carbon Dioxide" OR "Carbon Dioxide" OR "CO2") AND ("pollution" OR "emission" OR "emissions" OR "waste" OR "environment" OR "environmental*" OR "footprint" OR "footprint*" OR "sustainable" OR "hazard" OR "hazard*"))) OR TI=("environmental*" OR "sustainab*")) NOT DT=(meeting abstract) AND (PY=(2000 OR 2001 OR 2002 OR 2003 OR 2004 OR 2005 OR 2006 OR 2007 OR 2008 OR 2009 OR 2010 OR 2011 OR 2012 OR 2013 OR 2014 OR 2015 OR 2016 OR 2017 OR 2018 OR 2019 OR 2020 OR 2021 OR 2022)) AND (TI=(*("Meta-Analysis" OR metaanaly* OR "meta-analy*" or metanaly* OR "Systematic Review" OR "Cochrane Database Syst Rev" OR "prisma" OR "preferred reporting items" OR "prospero" OR ((systemati* OR scoping OR umbrella OR "structured literature") NEAR4 (review* OR overview*)) OR "systematic review*" OR "scoping review*" OR "umbrella review*" OR "structured literature review*" OR "systematic qualitative review*" OR "systematic quantitative review*" OR "systematic search and review" OR "systematized review" OR "systematised review" OR "systemic review" OR "systematic literature review*" OR "systematic integrative literature review*" OR "systematically review*" OR "scoping literature review*" OR "systematic critical review" OR "systematic integrative review*" OR "systematic evidence review" OR "Systematic integrative literature review*" OR "Systematic mixed studies review*" OR "Systematized literature review*" OR "Systematic overview*" OR "Systematic narrative review*" OR (("systemati*" OR "literature" OR "database*" OR "data-base*" OR "structured" OR "comprehensive*" OR "systemic*") NEAR4 "search*") OR ("Literature" AND "review" AND ("database*" OR "data-base*" OR "search*")) OR (("data extraction" OR "data source*") AND "study selection") OR ("search strategy" AND "selection criteria") OR ("data source*" AND "data synthesis") OR "medline" OR "pubmed" OR "embase" OR "Cochrane" OR (("critical" OR "rapid") NEAR4 ("review*" OR "overview*" OR "synthes*")) OR ((("critical*" OR "rapid*") NEAR4 ("review*" OR "overview*" OR "synthes*") NEAR4 ("search*" OR "database*" OR "data-base*"))) OR metasynthes* OR "meta-synthes*")* OR ("Randomized Controlled Trial" OR random* OR "RCT"OR "RCTs" OR "pragmatic clinical trial*" OR "practical clinical trial*" OR "non-inferiority trial*" OR "noninferiority trial*" OR "superiority trial*" OR "equivalence clinical trial*") OR ("Comparative Study" OR "comparison" OR "comparative" OR "compar*" OR "major clinical study" OR "clinical study" OR "case control study" OR "family study" OR "longitudinal study" OR "retrospective study" OR "prospective study" OR "cohort analysis" OR cohort* OR (("case control" NEAR1 (study OR studies))) OR (("follow up" NEAR1 (study OR studies))) OR (observational NEAR1 (study OR studies)) OR ((epidemiologic NEAR1 (study OR studies))) OR (("cross sectional" NEAR1 (study OR studies)))) OR ("life cycle assess*" OR "life cycle assessment" OR "life cycle inventory" OR "LCA" OR "LCAs" OR "life cycle inventory" OR "life cycle inventories")) OR AB=(*("Meta-Analysis" OR metaanaly* OR "meta-analy*" or metanaly* OR "Systematic Review" OR "Cochrane Database Syst Rev" OR "prisma" OR "preferred reporting items" OR "prospero" OR ((systemati* OR scoping OR umbrella OR "structured literature") NEAR4 (review* OR overview*)) OR "systematic review*" OR "scoping review*" OR "umbrella review*" OR "structured literature review*" OR "systematic qualitative review*" OR "systematic quantitative review*" OR "systematic search and review" OR "systematized review" OR "systematised review" OR "systemic review" OR "systematic literature review*" OR "systematic integrative literature review*" OR "systematically review*" OR "scoping literature review*" OR "systematic critical review" OR "systematic integrative review*" OR "systematic evidence review" OR "Systematic integrative literature review*" OR "Systematic mixed studies review*" OR "Systematized literature review*" OR "Systematic overview*" OR "Systematic narrative review*" OR (("systemati*" OR "literature" OR "database*" OR "data-base*" OR "structured" OR "comprehensive*" OR "systemic*") NEAR4 "search*") OR ("Literature" AND "review" AND ("database*" OR "data-base*" OR "search*")) OR (("data extraction" OR "data source*") AND "study selection") OR ("search strategy" AND "selection criteria") OR ("data source*" AND "data synthesis") OR "medline" OR "pubmed" OR "embase" OR "Cochrane" OR (("critical" OR "rapid") NEAR4 ("review*" OR "overview*" OR "synthes*")) OR ((("critical*" OR "rapid*") NEAR4 ("review*" OR "overview*" OR "synthes*") NEAR4 ("search*" OR "database*" OR "data-base*"))) OR metasynthes* OR "meta-synthes*")* OR ("Randomized Controlled Trial" OR random* OR "RCT"OR "RCTs" OR "pragmatic clinical trial*" OR "practical clinical trial*" OR "non-inferiority trial*" OR "noninferiority trial*" OR "superiority trial*" OR "equivalence clinical trial*") OR ("Comparative Study" OR "comparison" OR "comparative" OR "compar*" OR "major clinical study" OR "clinical study" OR "case control study" OR "family study" OR "longitudinal study" OR "retrospective study" OR "prospective study" OR "cohort analysis" OR cohort* OR (("case control" NEAR1 (study OR studies))) OR (("follow up" NEAR1 (study OR studies))) OR (observational NEAR1 (study OR studies)) OR ((epidemiologic NEAR1 (study OR studies))) OR (("cross sectional" NEAR1 (study OR studies)))) OR ("life cycle assess*" OR "life cycle assessment" OR "life cycle inventory" OR "LCA" OR "LCAs" OR "life cycle inventory" OR "life cycle inventories"))**)**

**Cochrane**

**UV4.1**

(("inhalation anesthetic agent" OR "inhalation anesthesia" OR "Closed Circuit Anesthesia" OR "Endotracheal Anesthesia" OR "Closed Circuit Anaesthesia" OR "Endotracheal Anaesthesia" OR "Halothane" OR "Isoflurane" OR "Methoxyflurane" OR "Sevoflurane" OR "Anaesthetic gas" OR "Anaesthetic gases" OR "Anesthetic gas" OR "Anesthetic gases" OR "Desflurane" OR "Enflurane" OR "Flurane" OR "Fluranes" OR "Gaseous anaesthesia" OR "Gaseous anaesthetic" OR "Gaseous anaesthetics" OR "Gaseous anesthesia" OR "Gaseous anesthetic" OR "Gaseous anesthetics" OR "Halothane" OR "Inhalation anaesthesia" OR "inhalation anaesthetic" OR "inhalation anaesthetics" OR "Inhalation anesthesia" OR "inhalation anesthetic" OR "inhalation anesthetics" OR "Inhalational agents" OR "Inhalational anaesthesia" OR "Inhalational anaesthetic" OR "Inhalational anaesthetic agent" OR "Inhalational anaesthetic agents" OR "Inhalational anaesthetics" OR "Inhalational anesthesia" OR "Inhalational anesthetic" OR "Inhalational anesthetic agent" OR "Inhalational anesthetic agents" OR "Inhalational anesthetics" OR "Inhaled anaesthetic" OR "Inhaled anaesthetic agent" OR "Inhaled anaesthetic agents" OR "Inhaled anaesthetics" OR "Inhaled anesthetic" OR "Inhaled anesthetic agents" OR "Inhaled anesthetics" OR "insufflation anaesthesia" OR "insufflation anaesthetic" OR "insufflation anaesthetics" OR "insufflation anesthesia" OR "insufflation anesthetic" OR "insufflation anesthetics" OR "Isoflurane" OR "Methoxyflurane" OR "Sevoflurane" OR "Vapor anaesthesia" OR "Vapor anaesthetic" OR "Vapor anaesthetics" OR "Vapor anesthesia" OR "Vapor anesthetic" OR "Vapor anesthetics" OR "Volatile agent" OR "Volatile agents" OR "Volatile Anaesthetic" OR "Volatile Anaesthetics" OR "Volatile anesthetic" OR "Volatile anesthetics" OR "Volatile drug" OR "Volatile drugs" OR "Volatile fluorinated liquid" OR "Volatile fluorinated liquids" OR "Volatile gas" OR "Volatile gases" OR "Volatile liquid agent" OR "Volatile liquid agents" OR "Anesthetic Gas" OR "Anesthetic Gases" OR "Inhalation Anaesthetic" OR "Inhalation Anaesthetics" OR "Inhalation Anesthetic" OR "Inhalation Anesthetics") AND ("intravenous anesthetic agent" OR "Intravenous Anesthesia" OR "Intravenous Anesthetics" OR "Intravenous Anaesthetics" OR "Intravenous Anesthetic" OR "Intravenous Anaesthetic" OR "Intravenous Anesthesia" OR "Intravenous Anaesthesia" OR "Dissociative Anesthetics" OR "Dissociative Anaesthetics" OR "Dissociative Anesthetic" OR "Dissociative Anaesthetic" OR "Dissociative Anesthesia" OR "Dissociative Anaesthesia" OR "2 (3 methoxyphenyl) 2 (ethylamino)cyclohexanone" OR "2 Oxo PCE" OR "Alfentanil" OR "Chloralose" OR "Diazepam" OR "Etomidate" OR "Fentanyl" OR "Ketamine" OR "Methohexital" OR "Midazolam" OR "Propanidid" OR "Propofol" OR "Sodium Oxybate" OR "Sufentanil" OR "Thiamylal" OR "Thiopental" OR "Tiletamine" OR "Urethane" OR "2 (3 methoxyphenyl) 2 (ethylamino)cyclohexanone" OR "2 Oxo PCE" OR "Alfentanil" OR "Chloralose" OR "Diazepam" OR "Etomidate" OR "Fentanyl" OR "intravenous anaesthesia" OR "intravenous anaesthesias" OR "Intravenous anaesthetic" OR "Intravenous anaesthetic agent" OR "Intravenous anaesthetic agents" OR "Intravenous anaesthetic drug" OR "Intravenous anaesthetic drugs" OR "Intravenous anaesthetics" OR "intravenous anesthesia" OR "intravenous anesthesias" OR "intravenous anesthetic" OR "Intravenous anesthetic agents" OR "Intravenous anesthetic agents" OR "Intravenous anesthetic drug" OR "Intravenous anesthetic drugs" OR "intravenous anesthetics" OR "IV anaesthesia" OR "IV anaesthetics" OR "IV anaesthetics" OR "IV anesthesia" OR "IV anesthetic" OR "IV anesthetics" OR "Ketamine" OR "Methohexital" OR "Midazolam" OR "Propanidid" OR "Propofol" OR "Sodium Oxybate" OR "Sufentanil" OR "Thiamylal" OR "Thiopental" OR "Tiletamine" OR "TIVA" OR "Total intravenous anaesthesia" OR "Total Intravenous anesthesia" OR "Urethane")):ti,ab,kw AND (("Carbon Footprint" OR "carbon footprint" OR "carbon footprint*" OR "Climate Change" OR "climate change" OR "CO2 emission" OR "CO2 emissions" OR "CO2 footprint" OR "CO2 footprint*" OR "environmental protection" OR "conservation of natural resources" OR "environmental protection" OR "Disposable Equipment" OR "Disposables" OR "Disposable" OR "ecological footprint" OR "ecological footprint*" OR "ecological sustainability" OR "environmental impact" OR "environmental impact" OR "environmental impact*" OR "environmental impacts" OR "environmental pollut*" OR "pollution" OR "environmental pollution" OR "environmental sustainab*" OR "environmental sustainability" OR "environmental sustainability" OR "Global Warming" OR "Global Warming" OR "Greenhouse Effect" OR "greenhouse effect*" OR "greenhouse effects" OR "greenhouse gas emission" OR "greenhouse gas emissions" OR "Greenhouse Gas" OR "greening" OR "hospital waste" OR "life cycle assessment" OR "environmental impact assessment" OR "life cycle assessment" OR "environmental impact assessment" OR "life cycle assess*" OR "life cycle assessment" OR "LCA" OR "LCAs" OR "life cycle inventory" OR "life cycle inventories" OR "Waste Disposal" OR "Hospital Waste" OR "medical waste" OR "Rising Sea Level" OR "Rising Sea Levels" OR "Sea Level Rise" OR "Sea Level Rise" OR "Waste Disposal" OR "waste water recycling" OR "Recycling" OR "recycling" OR "recycle*" OR "Equipment reuse" OR "Reusables" OR "reusable" OR "reuse" OR "reused" OR "reusing" OR "Waste Disposal" OR "Waste Management" OR "Plastic overuse" OR "Green surgery" OR "Emission reduction" OR "Emission reduction strategy" OR "air pollution control" OR "air pollution control" OR "acidification" OR "soil acidification" OR "ocean acidification" OR "acidification" OR "Acidification potential" OR "AP in kg SO2 equivalents" OR "eco-efficiency" OR "ecoefficiency" OR "eco-efficien*" OR "ecoefficien*" OR "ecotoxicity" OR "ecotoxicity" OR "ecotoxic*" OR "eco toxicity" OR "eco toxic*" OR "EP in kg PO4 equivalent" OR "Eutrophication" OR "eutrophication" OR "Eutrophication potential" OR "FAETP in kg DCB equivalent" OR "Freshwater Aquatic Ecotoxicity Potential" OR "GWP in kg CO2 equivalents" OR "H+ moles equivalents" OR "HTTP in kg Dichlorobenzene equivalent" OR "Human Toxicity Potential" OR "kg 2.4-D equivalents" OR "kg CFC-11 equivalent" OR "kg N equivalents" OR "kg NOx equivalents" OR "life cycle analysis" OR "ozone depletion" OR "Photochemical Ozone Depletion Potential" OR "POCP in kg ethane equivalent" OR "smog" OR "Waste" OR "waste" OR "wastes" OR "Ozone Depletion" OR "Smog" OR "Equipment reuse" OR "Greenhouse Gases" OR "Greenhouse Gas" OR "SO2 equiv*" OR "CO2 equiva*" OR "CFC-11 equiv*" OR "N equiv*" OR "Biodiversity" OR "Climatic change" OR "Green deal" OR "preservation of natural resources" OR "Refuse Disposal" OR "Waste Water" OR "Wastewater" OR "Water Purification" OR (("plastic*" OR "microplastic*") AND ("soup" OR "pollution" OR "overuse" OR "contamination")) OR "Sustainable Development" OR "Sustainable Development" OR (("Plastic" OR "plastics") AND "overuse") OR ("hydrogen*" AND "moles" AND "equiv*") OR ("Dichlorobenzen*" AND "equiv*") OR ("2,4-D" AND "equiv*") OR ("NOx" AND "equiv*") OR ("ethane" AND "equiv*") OR ("PO4" AND "equiv*") OR ("DCB" AND "equiv*") OR ("sustainability" AND ("environment*" OR "carbon")) OR (("Carbon Dioxide" OR "Carbon Dioxide" OR "CO2") AND ("pollution" OR "emission" OR "emissions" OR "waste" OR "environment" OR "environmental*" OR "footprint" OR "footprint*" OR "sustainable" OR "hazard" OR "hazard*"))):ti,ab,kw OR ("environmental" OR "sustainability"):ti)

**UV4.2**

(("inhalation anesthetic agent" OR "inhalation anesthesia" OR "Closed Circuit Anesthesia" OR "Endotracheal Anesthesia" OR "Closed Circuit Anaesthesia" OR "Endotracheal Anaesthesia" OR "Halothane" OR "Isoflurane" OR "Methoxyflurane" OR "Sevoflurane" OR "Anaesthetic gas" OR "Anaesthetic gases" OR "Anesthetic gas" OR "Anesthetic gases" OR "Desflurane" OR "Enflurane" OR "Flurane" OR "Fluranes" OR "Gaseous anaesthesia" OR "Gaseous anaesthetic" OR "Gaseous anaesthetics" OR "Gaseous anesthesia" OR "Gaseous anesthetic" OR "Gaseous anesthetics" OR "Halothane" OR "Inhalation anaesthesia" OR "inhalation anaesthetic" OR "inhalation anaesthetics" OR "Inhalation anesthesia" OR "inhalation anesthetic" OR "inhalation anesthetics" OR "Inhalational agents" OR "Inhalational anaesthesia" OR "Inhalational anaesthetic" OR "Inhalational anaesthetic agent" OR "Inhalational anaesthetic agents" OR "Inhalational anaesthetics" OR "Inhalational anesthesia" OR "Inhalational anesthetic" OR "Inhalational anesthetic agent" OR "Inhalational anesthetic agents" OR "Inhalational anesthetics" OR "Inhaled anaesthetic" OR "Inhaled anaesthetic agent" OR "Inhaled anaesthetic agents" OR "Inhaled anaesthetics" OR "Inhaled anesthetic" OR "Inhaled anesthetic agents" OR "Inhaled anesthetics" OR "insufflation anaesthesia" OR "insufflation anaesthetic" OR "insufflation anaesthetics" OR "insufflation anesthesia" OR "insufflation anesthetic" OR "insufflation anesthetics" OR "Isoflurane" OR "Methoxyflurane" OR "Sevoflurane" OR "Vapor anaesthesia" OR "Vapor anaesthetic" OR "Vapor anaesthetics" OR "Vapor anesthesia" OR "Vapor anesthetic" OR "Vapor anesthetics" OR "Volatile agent" OR "Volatile agents" OR "Volatile Anaesthetic" OR "Volatile Anaesthetics" OR "Volatile anesthetic" OR "Volatile anesthetics" OR "Volatile drug" OR "Volatile drugs" OR "Volatile fluorinated liquid" OR "Volatile fluorinated liquids" OR "Volatile gas" OR "Volatile gases" OR "Volatile liquid agent" OR "Volatile liquid agents" OR "Anesthetic Gas" OR "Anesthetic Gases" OR "Inhalation Anaesthetic" OR "Inhalation Anaesthetics" OR "Inhalation Anesthetic" OR "Inhalation Anesthetics") AND ("scavenging system" OR "anaesthetic gas scavenging system" OR "anesthetic gas scavenging system" OR "Contrafluran" OR "gas extract*" OR "gas extraction" OR "gas extraction system" OR "gas extraction systems" OR "gas scaveng*" OR "gas scavenger" OR "gas scavengers" OR "gas scavenging " OR "scavenging device" OR "scavenging device" OR "scavenging devices" OR "scavenging system" OR "scavenging systems" OR "vapour captur*" OR "vapour capture" OR "Vapour Capture Technology" OR "vapour recycl*" OR "vapour recycling technology" OR "vapour recycling")):ti,ab,kw **AND** (("Carbon Footprint" OR "carbon footprint" OR "carbon footprint*" OR "Climate Change" OR "climate change" OR "CO2 emission" OR "CO2 emissions" OR "CO2 footprint" OR "CO2 footprint*" OR "environmental protection" OR "conservation of natural resources" OR "environmental protection" OR "Disposable Equipment" OR "Disposables" OR "Disposable" OR "ecological footprint" OR "ecological footprint*" OR "ecological sustainability" OR "environmental impact" OR "environmental impact" OR "environmental impact*" OR "environmental impacts" OR "environmental pollut*" OR "pollution" OR "environmental pollution" OR "environmental sustainab*" OR "environmental sustainability" OR "environmental sustainability" OR "Global Warming" OR "Global Warming" OR "Greenhouse Effect" OR "greenhouse effect*" OR "greenhouse effects" OR "greenhouse gas emission" OR "greenhouse gas emissions" OR "Greenhouse Gas" OR "greening" OR "hospital waste" OR "life cycle assessment" OR "environmental impact assessment" OR "life cycle assessment" OR "environmental impact assessment" OR "life cycle assess*" OR "life cycle assessment" OR "LCA" OR "LCAs" OR "life cycle inventory" OR "life cycle inventories" OR "Waste Disposal" OR "Hospital Waste" OR "medical waste" OR "Rising Sea Level" OR "Rising Sea Levels" OR "Sea Level Rise" OR "Sea Level Rise" OR "Waste Disposal" OR "waste water recycling" OR "Recycling" OR "recycling" OR "recycle*" OR "Equipment reuse" OR "Reusables" OR "reusable" OR "reuse" OR "reused" OR "reusing" OR "Waste Disposal" OR "Waste Management" OR "Plastic overuse" OR "Green surgery" OR "Emission reduction" OR "Emission reduction strategy" OR "air pollution control" OR "air pollution control" OR "acidification" OR "soil acidification" OR "ocean acidification" OR "acidification" OR "Acidification potential" OR "AP in kg SO2 equivalents" OR "eco-efficiency" OR "ecoefficiency" OR "eco-efficien*" OR "ecoefficien*" OR "ecotoxicity" OR "ecotoxicity" OR "ecotoxic*" OR "eco toxicity" OR "eco toxic*" OR "EP in kg PO4 equivalent" OR "Eutrophication" OR "eutrophication" OR "Eutrophication potential" OR "FAETP in kg DCB equivalent" OR "Freshwater Aquatic Ecotoxicity Potential" OR "GWP in kg CO2 equivalents" OR "H+ moles equivalents" OR "HTTP in kg Dichlorobenzene equivalent" OR "Human Toxicity Potential" OR "kg 2.4-D equivalents" OR "kg CFC-11 equivalent" OR "kg N equivalents" OR "kg NOx equivalents" OR "life cycle analysis" OR "ozone depletion" OR "Photochemical Ozone Depletion Potential" OR "POCP in kg ethane equivalent" OR "smog" OR "Waste" OR "waste" OR "wastes" OR "Ozone Depletion" OR "Smog" OR "Equipment reuse" OR "Greenhouse Gases" OR "Greenhouse Gas" OR "SO2 equiv*" OR "CO2 equiva*" OR "CFC-11 equiv*" OR "N equiv*" OR "Biodiversity" OR "Climatic change" OR "Green deal" OR "preservation of natural resources" OR "Refuse Disposal" OR "Waste Water" OR "Wastewater" OR "Water Purification" OR (("plastic*" OR "microplastic*") AND ("soup" OR "pollution" OR "overuse" OR "contamination")) OR "Sustainable Development" OR "Sustainable Development" OR (("Plastic" OR "plastics") AND "overuse") OR ("hydrogen*" AND "moles" AND "equiv*") OR ("Dichlorobenzen*" AND "equiv*") OR ("2,4-D" AND "equiv*") OR ("NOx" AND "equiv*") OR ("ethane" AND "equiv*") OR ("PO4" AND "equiv*") OR ("DCB" AND "equiv*") OR ("sustainability" AND ("environment*" OR "carbon")) OR (("Carbon Dioxide" OR "Carbon Dioxide" OR "CO2") AND ("pollution" OR "emission" OR "emissions" OR "waste" OR "environment" OR "environmental*" OR "footprint" OR "footprint*" OR "sustainable" OR "hazard" OR "hazard*"))):ti,ab,kw OR ("environmental" OR "sustainability"):ti)

**UV4.3**

(("Local Anesthesia" OR "local anesthetic agent" OR "Nerve Block" OR "Local Anesthesia" OR "Local Anaesthesia" OR "Locoregional Anesthesia" OR "Locoregional Anaesthesia" OR "Local Anesthetics" OR "Local Anaesthetics" OR "Locoregional Anesthetics" OR "Locoregional Anaesthetics" OR "Local Anesthetic" OR "Local Anaesthetic" OR "Locoregional Anesthetic" OR "Locoregional Anaesthetic" OR "axillary block" OR "axillary block*" OR "Bier block" OR "Bier block*" OR "Bier's block" OR "Bier's block*" OR "brachial plexus block" OR "brachial plexus block*" OR "combined spinal epidural" OR "combined spinal epidural block" OR "combined spinal epidural block*" OR "epidural anaesthesia" OR "epidural anesthesia" OR "intravenous regional anesthaesia" OR "intravenous regional anesthesia" OR "ischiadic block" OR "ischiadic block*" OR "local infiltration" OR "lower extremity block" OR "lower extremity block*" OR "nerve block" OR "nerve block" OR "nerve block*" OR "nerve block*" OR "neuraxial anaesthesia" OR "neuraxial anesthesia" OR "neuraxial block" OR "neuraxial block*" OR "neuraxial technique" OR "neuraxial techniques" OR "peripheral nerve block" OR "peripheral nerve block*" OR "plexus block" OR "plexus block*" OR "plexus nerve block" OR "plexus nerve block*" OR "popliteal block" OR "popliteal block*" OR "regional anaesthesia" OR "regional anesthesia" OR "spinal anaesthesia" OR "spinal anaesthetic block" OR "spinal anaesthetic block*" OR "spinal anesthesia" OR "spinal anesthetic block" OR "spinal anesthetic block*" OR "supraclavicular block" OR "supraclavicular block*" OR "upper extremity block" OR "upper extremity block*") AND ("General Anesthesia" OR "general anesthesia" OR "general anaesthesia" OR "general anesthe*" OR "general anaesthe*" OR "inhalation anesthetic agent" OR "inhalation anesthesia" OR "Closed Circuit Anesthesia" OR "Endotracheal Anesthesia" OR "Closed Circuit Anaesthesia" OR "Endotracheal Anaesthesia" OR "Halothane" OR "Isoflurane" OR "Methoxyflurane" OR "Sevoflurane" OR "Anaesthetic gas" OR "Anaesthetic gases" OR "Anesthetic gas" OR "Anesthetic gases" OR "Desflurane" OR "Enflurane" OR "Flurane" OR "Fluranes" OR "Gaseous anaesthesia" OR "Gaseous anaesthetic" OR "Gaseous anaesthetics" OR "Gaseous anesthesia" OR "Gaseous anesthetic" OR "Gaseous anesthetics" OR "Halothane" OR "Inhalation anaesthesia" OR "inhalation anaesthetic" OR "inhalation anaesthetics" OR "Inhalation anesthesia" OR "inhalation anesthetic" OR "inhalation anesthetics" OR "Inhalational agents" OR "Inhalational anaesthesia" OR "Inhalational anaesthetic" OR "Inhalational anaesthetic agent" OR "Inhalational anaesthetic agents" OR "Inhalational anaesthetics" OR "Inhalational anesthesia" OR "Inhalational anesthetic" OR "Inhalational anesthetic agent" OR "Inhalational anesthetic agents" OR "Inhalational anesthetics" OR "Inhaled anaesthetic" OR "Inhaled anaesthetic agent" OR "Inhaled anaesthetic agents" OR "Inhaled anaesthetics" OR "Inhaled anesthetic" OR "Inhaled anesthetic agents" OR "Inhaled anesthetics" OR "insufflation anaesthesia" OR "insufflation anaesthetic" OR "insufflation anaesthetics" OR "insufflation anesthesia" OR "insufflation anesthetic" OR "insufflation anesthetics" OR "Isoflurane" OR "Methoxyflurane" OR "Sevoflurane" OR "Vapor anaesthesia" OR "Vapor anaesthetic" OR "Vapor anaesthetics" OR "Vapor anesthesia" OR "Vapor anesthetic" OR "Vapor anesthetics" OR "Volatile agent" OR "Volatile agents" OR "Volatile Anaesthetic" OR "Volatile Anaesthetics" OR "Volatile anesthetic" OR "Volatile anesthetics" OR "Volatile drug" OR "Volatile drugs" OR "Volatile fluorinated liquid" OR "Volatile fluorinated liquids" OR "Volatile gas" OR "Volatile gases" OR "Volatile liquid agent" OR "Volatile liquid agents" OR "Anesthetic Gas" OR "Anesthetic Gases" OR "Inhalation Anaesthetic" OR "Inhalation Anaesthetics" OR "Inhalation Anesthetic" OR "Inhalation Anesthetics" OR "intravenous anesthetic agent" OR "Intravenous Anesthesia" OR "Intravenous Anesthetics" OR "Intravenous Anaesthetics" OR "Intravenous Anesthetic" OR "Intravenous Anaesthetic" OR "Intravenous Anesthesia" OR "Intravenous Anaesthesia" OR "Dissociative Anesthetics" OR "Dissociative Anaesthetics" OR "Dissociative Anesthetic" OR "Dissociative Anaesthetic" OR "Dissociative Anesthesia" OR "Dissociative Anaesthesia" OR "2 (3 methoxyphenyl) 2 (ethylamino)cyclohexanone" OR "2 Oxo PCE" OR "Alfentanil" OR "Chloralose" OR "Diazepam" OR "Etomidate" OR "Fentanyl" OR "Ketamine" OR "Methohexital" OR "Midazolam" OR "Propanidid" OR "Propofol" OR "Sodium Oxybate" OR "Sufentanil" OR "Thiamylal" OR "Thiopental" OR "Tiletamine" OR "Urethane" OR "2 (3 methoxyphenyl) 2 (ethylamino)cyclohexanone" OR "2 Oxo PCE" OR "Alfentanil" OR "Chloralose" OR "Diazepam" OR "Etomidate" OR "Fentanyl" OR "intravenous anaesthesia" OR "intravenous anaesthesias" OR "Intravenous anaesthetic" OR "Intravenous anaesthetic agent" OR "Intravenous anaesthetic agents" OR "Intravenous anaesthetic drug" OR "Intravenous anaesthetic drugs" OR "Intravenous anaesthetics" OR "intravenous anesthesia" OR "intravenous anesthesias" OR "intravenous anesthetic" OR "Intravenous anesthetic agents" OR "Intravenous anesthetic agents" OR "Intravenous anesthetic drug" OR "Intravenous anesthetic drugs" OR "intravenous anesthetics" OR "IV anaesthesia" OR "IV anaesthetics" OR "IV anaesthetics" OR "IV anesthesia" OR "IV anesthetic" OR "IV anesthetics" OR "Ketamine" OR "Methohexital" OR "Midazolam" OR "Propanidid" OR "Propofol" OR "Sodium Oxybate" OR "Sufentanil" OR "Thiamylal" OR "Thiopental" OR "Tiletamine" OR "TIVA" OR "Total intravenous anaesthesia" OR "Total Intravenous anesthesia" OR "Urethane")):ti,ab,kw AND (("Carbon Footprint" OR "carbon footprint" OR "carbon footprint*" OR "Climate Change" OR "climate change" OR "CO2 emission" OR "CO2 emissions" OR "CO2 footprint" OR "CO2 footprint*" OR "environmental protection" OR "conservation of natural resources" OR "environmental protection" OR "Disposable Equipment" OR "Disposables" OR "Disposable" OR "ecological footprint" OR "ecological footprint*" OR "ecological sustainability" OR "environmental impact" OR "environmental impact" OR "environmental impact*" OR "environmental impacts" OR "environmental pollut*" OR "pollution" OR "environmental pollution" OR "environmental sustainab*" OR "environmental sustainability" OR "environmental sustainability" OR "Global Warming" OR "Global Warming" OR "Greenhouse Effect" OR "greenhouse effect*" OR "greenhouse effects" OR "greenhouse gas emission" OR "greenhouse gas emissions" OR "Greenhouse Gas" OR "greening" OR "hospital waste" OR "life cycle assessment" OR "environmental impact assessment" OR "life cycle assessment" OR "environmental impact assessment" OR "life cycle assess*" OR "life cycle assessment" OR "LCA" OR "LCAs" OR "life cycle inventory" OR "life cycle inventories" OR "Waste Disposal" OR "Hospital Waste" OR "medical waste" OR "Rising Sea Level" OR "Rising Sea Levels" OR "Sea Level Rise" OR "Sea Level Rise" OR "Waste Disposal" OR "waste water recycling" OR "Recycling" OR "recycling" OR "recycle*" OR "Equipment reuse" OR "Reusables" OR "reusable" OR "reuse" OR "reused" OR "reusing" OR "Waste Disposal" OR "Waste Management" OR "Plastic overuse" OR "Green surgery" OR "Emission reduction" OR "Emission reduction strategy" OR "air pollution control" OR "air pollution control" OR "acidification" OR "soil acidification" OR "ocean acidification" OR "acidification" OR "Acidification potential" OR "AP in kg SO2 equivalents" OR "eco-efficiency" OR "ecoefficiency" OR "eco-efficien*" OR "ecoefficien*" OR "ecotoxicity" OR "ecotoxicity" OR "ecotoxic*" OR "eco toxicity" OR "eco toxic*" OR "EP in kg PO4 equivalent" OR "Eutrophication" OR "eutrophication" OR "Eutrophication potential" OR "FAETP in kg DCB equivalent" OR "Freshwater Aquatic Ecotoxicity Potential" OR "GWP in kg CO2 equivalents" OR "H+ moles equivalents" OR "HTTP in kg Dichlorobenzene equivalent" OR "Human Toxicity Potential" OR "kg 2.4-D equivalents" OR "kg CFC-11 equivalent" OR "kg N equivalents" OR "kg NOx equivalents" OR "life cycle analysis" OR "ozone depletion" OR "Photochemical Ozone Depletion Potential" OR "POCP in kg ethane equivalent" OR "smog" OR "Waste" OR "waste" OR "wastes" OR "Ozone Depletion" OR "Smog" OR "Equipment reuse" OR "Greenhouse Gases" OR "Greenhouse Gas" OR "SO2 equiv*" OR "CO2 equiva*" OR "CFC-11 equiv*" OR "N equiv*" OR "Biodiversity" OR "Climatic change" OR "Green deal" OR "preservation of natural resources" OR "Refuse Disposal" OR "Waste Water" OR "Wastewater" OR "Water Purification" OR (("plastic*" OR "microplastic*") AND ("soup" OR "pollution" OR "overuse" OR "contamination")) OR "Sustainable Development" OR "Sustainable Development" OR (("Plastic" OR "plastics") AND "overuse") OR ("hydrogen*" AND "moles" AND "equiv*") OR ("Dichlorobenzen*" AND "equiv*") OR ("2,4-D" AND "equiv*") OR ("NOx" AND "equiv*") OR ("ethane" AND "equiv*") OR ("PO4" AND "equiv*") OR ("DCB" AND "equiv*") OR ("sustainability" AND ("environment*" OR "carbon")) OR (("Carbon Dioxide" OR "Carbon Dioxide" OR "CO2") AND ("pollution" OR "emission" OR "emissions" OR "waste" OR "environment" OR "environmental*" OR "footprint" OR "footprint*" OR "sustainable" OR "hazard" OR "hazard*"))):ti,ab,kw OR ("environmental" OR "sustainability"):ti)

**Emcare (OVID)**

**UV4.1**

((exp "inhalation anesthetic agent"/ OR exp "inhalation anesthesia"/ OR "Closed-Circuit Anesthesia".mp OR "Endotracheal Anesthesia".mp OR "Closed-Circuit Anaesthesia".mp OR "Endotracheal Anaesthesia".mp OR "Halothane"/ OR "Isoflurane"/ OR "Methoxyflurane"/ OR "Sevoflurane"/ OR "Anaesthetic gas".mp OR "Anaesthetic gases".mp OR "Anesthetic gas".mp OR "Anesthetic gases".mp OR "Desflurane".mp OR "Enflurane".mp OR "Flurane".mp OR "Fluranes".mp OR "Gaseous anaesthesia".mp OR "Gaseous anaesthetic".mp OR "Gaseous anaesthetics".mp OR "Gaseous anesthesia".mp OR "Gaseous anesthetic".mp OR "Gaseous anesthetics".mp OR "Halothane".mp OR "Inhalation anaesthesia".mp OR "inhalation anaesthetic".mp OR "inhalation anaesthetics".mp OR "Inhalation anesthesia".mp OR "inhalation anesthetic".mp OR "inhalation anesthetics".mp OR "Inhalational agents".mp OR "Inhalational anaesthesia".mp OR "Inhalational anaesthetic".mp OR "Inhalational anaesthetic agent".mp OR "Inhalational anaesthetic agents".mp OR "Inhalational anaesthetics".mp OR "Inhalational anesthesia".mp OR "Inhalational anesthetic".mp OR "Inhalational anesthetic agent".mp OR "Inhalational anesthetic agents".mp OR "Inhalational anesthetics".mp OR "Inhaled anaesthetic".mp OR "Inhaled anaesthetic agent".mp OR "Inhaled anaesthetic agents".mp OR "Inhaled anaesthetics".mp OR "Inhaled anesthetic".mp OR "Inhaled anesthetic agents".mp OR "Inhaled anesthetics".mp OR "insufflation anaesthesia".mp OR "insufflation anaesthetic".mp OR "insufflation anaesthetics".mp OR "insufflation anesthesia".mp OR "insufflation anesthetic".mp OR "insufflation anesthetics".mp OR "Isoflurane".mp OR "Methoxyflurane".mp OR "Sevoflurane".mp OR "Vapor anaesthesia".mp OR "Vapor anaesthetic".mp OR "Vapor anaesthetics".mp OR "Vapor anesthesia".mp OR "Vapor anesthetic".mp OR "Vapor anesthetics".mp OR "Volatile agent".mp OR "Volatile agents".mp OR "Volatile Anaesthetic".mp OR "Volatile Anaesthetics".mp OR "Volatile anesthetic".mp OR "Volatile anesthetics".mp OR "Volatile drug".mp OR "Volatile drugs".mp OR "Volatile fluorinated liquid".mp OR "Volatile fluorinated liquids".mp OR "Volatile gas".mp OR "Volatile gases".mp OR "Volatile liquid agent".mp OR "Volatile liquid agents".mp OR "Anesthetic Gas".mp OR "Anesthetic Gases".mp OR "Inhalation Anaesthetic".mp OR "Inhalation Anaesthetics".mp OR "Inhalation Anesthetic".mp OR "Inhalation Anesthetics".mp) AND (exp "intravenous anesthetic agent"/ OR exp "Intravenous Anesthesia"/ OR "Intravenous Anesthetics".mp OR "Intravenous Anaesthetics".mp OR "Intravenous Anesthetic".mp OR "Intravenous Anaesthetic".mp OR "Intravenous Anesthesia".mp OR "Intravenous Anaesthesia".mp OR "Dissociative Anesthetics".mp OR "Dissociative Anaesthetics".mp OR "Dissociative Anesthetic".mp OR "Dissociative Anaesthetic".mp OR "Dissociative Anesthesia".mp OR "Dissociative Anaesthesia".mp OR "2-(3-methoxyphenyl)-2-(ethylamino)cyclohexanone"/ OR "2-Oxo-PCE"/ OR "Alfentanil"/ OR "Chloralose"/ OR "Diazepam"/ OR "Etomidate"/ OR "Fentanyl"/ OR "Ketamine"/ OR "Methohexital"/ OR "Midazolam"/ OR "Propanidid"/ OR "Propofol"/ OR "Sodium Oxybate"/ OR "Sufentanil"/ OR "Thiamylal"/ OR "Thiopental"/ OR "Tiletamine"/ OR "Urethane"/ OR "2-(3-methoxyphenyl)-2-(ethylamino)cyclohexanone".mp OR "2-Oxo-PCE".mp OR "Alfentanil".mp OR "Chloralose".mp OR "Diazepam".mp OR "Etomidate".mp OR "Fentanyl".mp OR "intravenous anaesthesia".mp OR "intravenous anaesthesias".mp OR "Intravenous anaesthetic".mp OR "Intravenous anaesthetic agent".mp OR "Intravenous anaesthetic agents".mp OR "Intravenous anaesthetic drug".mp OR "Intravenous anaesthetic drugs".mp OR "Intravenous anaesthetics".mp OR "intravenous anesthesia".mp OR "intravenous anesthesias".mp OR "intravenous anesthetic".mp OR "Intravenous anesthetic agents".mp OR "Intravenous anesthetic agents".mp OR "Intravenous anesthetic drug".mp OR "Intravenous anesthetic drugs".mp OR "intravenous anesthetics".mp OR "IV anaesthesia".mp OR "IV anaesthetics".mp OR "IV anaesthetics".mp OR "IV anesthesia".mp OR "IV anesthetic".mp OR "IV anesthetics".mp OR "Ketamine".mp OR "Methohexital".mp OR "Midazolam".mp OR "Propanidid".mp OR "Propofol".mp OR "Sodium Oxybate".mp OR "Sufentanil".mp OR "Thiamylal".mp OR "Thiopental".mp OR "Tiletamine".mp OR "TIVA".mp OR "Total intravenous anaesthesia".mp OR "Total Intravenous anesthesia".mp OR "Urethane".mp)) AND ("Carbon Footprint"/ OR "carbon footprint".mp OR "carbon footprint*".mp OR exp "Climate Change"/ OR "climate change".mp OR "CO2 emission".mp OR "CO2 emissions".mp OR "CO2 footprint".mp OR "CO2 footprint*".mp OR exp "environmental protection"/ OR "conservation of natural resources".mp OR "environmental protection".mp OR "Disposable Equipment"/ OR "Disposables".mp OR "Disposable".mp OR "ecological footprint".mp OR "ecological footprint*".mp OR "ecological sustainability".mp OR exp "environmental impact"/ OR "environmental impact".mp OR "environmental impact*".mp OR "environmental impacts".mp OR "environmental pollut*".mp OR exp "pollution"/ OR "environmental pollution".mp OR "environmental sustainab*".mp OR "environmental sustainability"/ OR "environmental sustainability".mp OR "Global Warming"/ OR "Global Warming".mp OR "Greenhouse Effect"/ OR "greenhouse effect*".mp OR "greenhouse effects".mp OR "greenhouse gas emission".mp OR "greenhouse gas emissions".mp OR "Greenhouse Gas"/ OR "greening".mp OR "hospital waste".mp OR "life cycle assessment"/ OR "environmental impact assessment"/ OR "life cycle assess*".mp OR "life cycle assessment".mp OR "LCA".mp OR "LCAs".mp OR "life cycle inventory".mp OR "life cycle inventories".mp OR exp "Waste Disposal"/ OR exp "Hospital Waste"/ OR "medical waste".mp OR "Rising Sea Level".mp OR "Rising Sea Levels".mp OR "Sea Level Rise"/ OR "Sea Level Rise".mp OR "sustainability".ti OR "Waste Disposal".mp OR "waste water recycling"/ OR "Recycling"/ OR "recycling".mp OR "recycle*".mp OR "Equipment reuse".mp OR "Reusables".mp OR "reusable".mp OR "reuse".mp OR "reused".mp OR "reusing".mp OR exp "Waste Disposal"/ OR exp "Waste Management"/ OR "Plastic overuse".mp OR "Green surgery".mp OR "Emission reduction".mp OR "Emission reduction strategy".mp OR "air pollution control"/ OR "air pollution control".mp OR "Environment"/ OR "Environmental*".ti OR "acidification"/ OR "soil acidification"/ OR "ocean acidification"/ OR "acidification".mp OR "Acidification potential".mp OR "AP in kg SO2 equivalents".mp OR "eco-efficiency".mp OR "ecoefficiency".mp OR "eco-efficien*".mp OR "ecoefficien*".mp OR "ecotoxicity"/ OR "ecotoxicity".mp OR "ecotoxic*".mp OR "eco toxicity".mp OR "eco toxic*".mp OR "EP in kg PO4 equivalent".mp OR exp "Eutrophication"/ OR "eutrophication".mp OR "Eutrophication potential".mp OR "FAETP in kg DCB equivalent".mp OR "Freshwater Aquatic Ecotoxicity Potential".mp OR "GWP in kg CO2 equivalents".mp OR "H+ moles equivalents".mp OR "HTTP in kg Dichlorobenzene equivalent".mp OR "Human Toxicity Potential".mp OR "kg 2.4-D equivalents".mp OR "kg CFC-11 equivalent".mp OR "kg N equivalents".mp OR "kg NOx equivalents".mp OR "life cycle analysis".mp OR "ozone depletion".mp OR "Photochemical Ozone Depletion Potential".mp OR "POCP in kg ethane equivalent".mp OR "smog".mp OR exp "Waste"/ OR "waste".mp OR "wastes".mp OR "Ozone Depletion"/ OR "Smog"/ OR "Equipment reuse".mp OR "Greenhouse Gases".mp OR "Greenhouse Gas".mp OR "SO2 equiv*".mp OR "CO2 equiva*".mp OR "CFC-11 equiv*".mp OR "N equiv*".mp OR exp "Biodiversity"/ OR "Biodiversity".mp OR "Climatic change".mp OR "Green deal".mp OR "preservation of natural resources".mp OR "Refuse Disposal".mp OR exp "Wastewater"/ OR "Waste Water".mp OR "Wastewater".mp OR exp "Water Management"/ OR "Water Purification".mp OR (("plastic*".mp OR "microplastic*".mp) AND ("soup".mp OR "pollution".mp OR "overuse".mp OR "contamination".mp)) OR "Sustainable Development"/ OR "Sustainable Development".mp OR (("Plastic".mp OR "plastics".mp) AND "overuse".mp) OR ("hydrogen*".mp AND "moles".mp AND "equiv*".mp) OR ("Dichlorobenzen*".mp AND "equiv*".mp) OR ("2,4-D".mp AND "equiv*".mp) OR ("NOx".mp AND "equiv*".mp) OR ("ethane".mp AND "equiv*".mp) OR ("PO4".mp AND "equiv*".mp) OR ("DCB".mp AND "equiv*".mp) OR ("sustainability".mp AND ("environment*".mp OR "carbon".mp)) OR (("Carbon Dioxide"/ OR "Carbon Dioxide".mp OR "CO2".mp) AND ("pollution".mp OR "emission".mp OR "emissions".mp OR "waste".mp OR "environment".mp OR "environmental*".mp OR "footprint".mp OR "footprint*".mp OR "sustainable".mp OR "hazard".mp OR "hazard*".mp))) NOT (conference review or conference abstract).pt AND (2000 OR 2001 OR 2002 OR 2003 OR 2004 OR 2005 OR 2006 OR 2007 OR 2008 OR 2009 OR 2010 OR 2011 OR 2012 OR 2013 OR 2014 OR 2015 OR 2016 OR 2017 OR 2018 OR 2019 OR 2020 OR 2021 OR 2022).yr AND (*(exp "meta analysis"/ OR exp "meta analysis (topic)"/ OR metaanaly*.ti,ab OR "meta analy*".ti,ab OR metanaly*.ti,ab OR "systematic review"/ OR "cochrane database of systematic reviews".jn OR prisma.ti,ab OR prospero.ti,ab OR (((systemati* OR scoping OR umbrella OR "structured literature") ADJ3 (review* OR overview*)).ti,ab) OR ((systemic* ADJ1 review*).ti,ab) OR (((systemati* OR literature OR database* OR "data base*") ADJ10 search*).ti,ab) OR (((structured OR comprehensive* OR systemic*) ADJ3 search*).ti,ab) OR (((literature ADJ3 review*).ti,ab) AND (search*.ti,ab OR database*.ti,ab OR "data base*".ti,ab)) OR (("data extraction".ti,ab OR "data source*".ti,ab) AND "study selection".ti,ab) OR ("search strategy".ti,ab AND "selection criteria".ti,ab) OR ("data source*".ti,ab AND "data synthesis".ti,ab) OR medline.ab OR pubmed.ab OR embase.ab OR cochrane.ab OR (((critical OR rapid) ADJ2 (review* OR overview* OR synthes*)).ti) OR ((((critical* OR rapid*) ADJ3 (review* OR overview* OR synthes*)).ab) AND (search*.ab OR database*.ab OR "data base*".ab)) OR metasynthes*.ti,ab OR "meta synthes*".ti,ab)* OR (exp "clinical trial"/ OR exp "randomization"/ OR exp "single blind procedure"/ OR exp "double blind procedure"/ OR exp "crossover procedure"/ OR exp "placebo"/ OR exp "prospective study"/ OR rct.ti,ab OR random*.ti,ab OR "single blind".ti,ab OR "randomised controlled trial".ti,ab OR exp "randomized controlled trial"/ OR placebo*.ti,ab) OR (exp "Comparative Study"/ OR "comparison".ti,ab OR "comparative".ti,ab OR "compar*".ti,ab OR "major clinical study"/ OR "clinical study"/ OR "case control study"/ OR "family study"/ OR "longitudinal study"/ OR "retrospective study"/ OR "prospective study"/ OR "cohort analysis"/ OR cohort*.ti,ab OR (("case control" ADJ1 (study OR studies)).ti,ab) OR (("follow up" ADJ1 (study OR studies)).ti,ab) OR (observational ADJ1 (study OR studies)).ti,ab OR ((epidemiologic ADJ1 (study OR studies)).ti,ab) OR (("cross sectional" ADJ1 (study OR studies)).ti,ab)) OR ("life cycle assessment"/ OR "environmental impact assessment"/ OR "life cycle assess*".mp OR "life cycle assessment".mp OR "life cycle inventory".mp OR "LCA".mp OR "LCAs".mp OR "life cycle inventory".mp OR "life cycle inventories".mp))

**UV4.2**

(TS=("Closed-Circuit Anesthesia" OR "Endotracheal Anesthesia" OR "Closed-Circuit Anaesthesia" OR "Endotracheal Anaesthesia" OR "Halothane" OR "Isoflurane" OR "Methoxyflurane" OR "Sevoflurane" OR "Anaesthetic gas" OR "Anaesthetic gases" OR "Anesthetic gas" OR "Anesthetic gases" OR "Desflurane" OR "Enflurane" OR "Flurane" OR "Fluranes" OR "Gaseous anaesthesia" OR "Gaseous anaesthetic" OR "Gaseous anaesthetics" OR "Gaseous anesthesia" OR "Gaseous anesthetic" OR "Gaseous anesthetics" OR "Halothane" OR "Inhalation anaesthesia" OR "inhalation anaesthetic" OR "inhalation anaesthetics" OR "Inhalation anesthesia" OR "inhalation anesthetic" OR "inhalation anesthetics" OR "Inhalational agents" OR "Inhalational anaesthesia" OR "Inhalational anaesthetic" OR "Inhalational anaesthetic agent" OR "Inhalational anaesthetic agents" OR "Inhalational anaesthetics" OR "Inhalational anesthesia" OR "Inhalational anesthetic" OR "Inhalational anesthetic agent" OR "Inhalational anesthetic agents" OR "Inhalational anesthetics" OR "Inhaled anaesthetic" OR "Inhaled anaesthetic agent" OR "Inhaled anaesthetic agents" OR "Inhaled anaesthetics" OR "Inhaled anesthetic" OR "Inhaled anesthetic agents" OR "Inhaled anesthetics" OR "insufflation anaesthesia" OR "insufflation anaesthetic" OR "insufflation anaesthetics" OR "insufflation anesthesia" OR "insufflation anesthetic" OR "insufflation anesthetics" OR "Isoflurane" OR "Methoxyflurane" OR "Sevoflurane" OR "Vapor anaesthesia" OR "Vapor anaesthetic" OR "Vapor anaesthetics" OR "Vapor anesthesia" OR "Vapor anesthetic" OR "Vapor anesthetics" OR "Volatile agent" OR "Volatile agents" OR "Volatile Anaesthetic" OR "Volatile Anaesthetics" OR "Volatile anesthetic" OR "Volatile anesthetics" OR "Volatile drug" OR "Volatile drugs" OR "Volatile fluorinated liquid" OR "Volatile fluorinated liquids" OR "Volatile gas" OR "Volatile gases" OR "Volatile liquid agent" OR "Volatile liquid agents" OR "Anesthetics, Inhalation" OR "Anesthetic Gas" OR "Anesthetic Gases" OR "Inhalation Anaesthetic" OR "Inhalation Anaesthetics" OR "Inhalation Anesthetic" OR "Inhalation Anesthetics") AND TS=("scavenging system" OR "anaesthetic gas scavenging system" OR "anesthetic gas scavenging system" OR "Contrafluran" OR "gas extract*" OR "gas extraction" OR "gas extraction system" OR "gas extraction systems" OR "gas scaveng*" OR "gas scavenger" OR "gas scavengers" OR "gas scavenging " OR "scavenging device" OR "scavenging device" OR "scavenging devices" OR "scavenging system" OR "scavenging systems" OR "vapour captur*" OR "vapour capture" OR "Vapour Capture Technology" OR "vapour recycl*" OR "vapour recycling technology" OR "vapour recycling")) AND (TS=("Carbon Footprint" OR "carbon footprint" OR "carbon footprint*" OR "Climate Change" OR "climate change" OR "CO2 emission" OR "CO2 emissions" OR "CO2 footprint" OR "CO2 footprint*" OR "environmental protection" OR "conservation of natural resources" OR "environmental protection" OR "Disposable Equipment" OR "Disposables" OR "Disposable" OR "ecological footprint" OR "ecological footprint*" OR "ecological sustainability" OR "environmental impact" OR "environmental impact" OR "environmental impact*" OR "environmental impacts" OR "environmental pollut*" OR "pollution" OR "environmental pollution" OR "environmental sustainab*" OR "environmental sustainability" OR "environmental sustainability" OR "Global Warming" OR "Global Warming" OR "Greenhouse Effect" OR "greenhouse effect*" OR "greenhouse effects" OR "greenhouse gas emission" OR "greenhouse gas emissions" OR "Greenhouse Gas" OR "greening" OR "hospital waste" OR "life cycle assessment" OR "environmental impact assessment" OR "life cycle assess*" OR "life cycle assessment" OR "LCA" OR "LCAs" OR "life cycle inventory" OR "life cycle inventories" OR "Waste Disposal" OR "Hospital Waste" OR "medical waste" OR "Rising Sea Level" OR "Rising Sea Levels" OR "Sea Level Rise" OR "Sea Level Rise" OR "Waste Disposal" OR "waste water recycling" OR "Recycling" OR "recycling" OR "recycle*" OR "Equipment reuse" OR "Reusables" OR "reusable" OR "reuse" OR "reused" OR "reusing" OR "Waste Disposal" OR "Waste Management" OR "Plastic overuse" OR "Green surgery" OR "Emission reduction" OR "Emission reduction strategy" OR "air pollution control" OR "air pollution control" OR "acidification" OR "soil acidification" OR "ocean acidification" OR "acidification" OR "Acidification potential" OR "AP in kg SO2 equivalents" OR "eco-efficiency" OR "ecoefficiency" OR "eco-efficien*" OR "ecoefficien*" OR "ecotoxicity" OR "ecotoxicity" OR "ecotoxic*" OR "eco toxicity" OR "eco toxic*" OR "EP in kg PO4 equivalent" OR "Eutrophication" OR "eutrophication" OR "Eutrophication potential" OR "FAETP in kg DCB equivalent" OR "Freshwater Aquatic Ecotoxicity Potential" OR "GWP in kg CO2 equivalents" OR "H+ moles equivalents" OR "HTTP in kg Dichlorobenzene equivalent" OR "Human Toxicity Potential" OR "kg 2.4-D equivalents" OR "kg CFC-11 equivalent" OR "kg N equivalents" OR "kg NOx equivalents" OR "life cycle analysis" OR "ozone depletion" OR "Photochemical Ozone Depletion Potential" OR "POCP in kg ethane equivalent" OR "smog" OR "Waste" OR "waste" OR "wastes" OR "Ozone Depletion" OR "Smog" OR "Equipment reuse" OR "Greenhouse Gases" OR "Greenhouse Gas" OR "SO2 equiv*" OR "CO2 equiva*" OR "CFC-11 equiv*" OR "N equiv*" OR "Sustainable Development" OR "Sustainable Development" OR "Biodiversity" OR "Climatic change" OR "Green deal" OR "preservation of natural resources" OR "Refuse Disposal" OR "Waste Water" OR "Wastewater" OR "Water Purification" OR (("plastic*" OR "microplastic*") AND ("soup" OR "pollution" OR "overuse" OR "contamination")) OR (("Plastic" OR "plastics") AND "overuse") OR ("hydrogen*" AND "moles" AND "equiv*") OR ("Dichlorobenzen*" AND "equiv*") OR ("2,4-D" AND "equiv*") OR ("NOx" AND "equiv*") OR ("ethane" AND "equiv*") OR ("PO4" AND "equiv*") OR ("DCB" AND "equiv*") OR ("sustainability" AND ("environment*" OR "carbon")) OR (("Carbon Dioxide" OR "Carbon Dioxide" OR "CO2") AND ("pollution" OR "emission" OR "emissions" OR "waste" OR "environment" OR "environmental*" OR "footprint" OR "footprint*" OR "sustainable" OR "hazard" OR "hazard*"))) OR TI=("environmental*" OR "sustainab*")) NOT DT=(meeting abstract) AND (PY=(2000 OR 2001 OR 2002 OR 2003 OR 2004 OR 2005 OR 2006 OR 2007 OR 2008 OR 2009 OR 2010 OR 2011 OR 2012 OR 2013 OR 2014 OR 2015 OR 2016 OR 2017 OR 2018 OR 2019 OR 2020 OR 2021 OR 2022)) AND (TI=(*("Meta-Analysis" OR metaanaly* OR "meta-analy*" or metanaly* OR "Systematic Review" OR "Cochrane Database Syst Rev" OR "prisma" OR "preferred reporting items" OR "prospero" OR ((systemati* OR scoping OR umbrella OR "structured literature") NEAR4 (review* OR overview*)) OR "systematic review*" OR "scoping review*" OR "umbrella review*" OR "structured literature review*" OR "systematic qualitative review*" OR "systematic quantitative review*" OR "systematic search and review" OR "systematized review" OR "systematised review" OR "systemic review" OR "systematic literature review*" OR "systematic integrative literature review*" OR "systematically review*" OR "scoping literature review*" OR "systematic critical review" OR "systematic integrative review*" OR "systematic evidence review" OR "Systematic integrative literature review*" OR "Systematic mixed studies review*" OR "Systematized literature review*" OR "Systematic overview*" OR "Systematic narrative review*" OR (("systemati*" OR "literature" OR "database*" OR "data-base*" OR "structured" OR "comprehensive*" OR "systemic*") NEAR4 "search*") OR ("Literature" AND "review" AND ("database*" OR "data-base*" OR "search*")) OR (("data extraction" OR "data source*") AND "study selection") OR ("search strategy" AND "selection criteria") OR ("data source*" AND "data synthesis") OR "medline" OR "pubmed" OR "embase" OR "Cochrane" OR (("critical" OR "rapid") NEAR4 ("review*" OR "overview*" OR "synthes*")) OR ((("critical*" OR "rapid*") NEAR4 ("review*" OR "overview*" OR "synthes*") NEAR4 ("search*" OR "database*" OR "data-base*"))) OR metasynthes* OR "meta-synthes*")* OR ("Randomized Controlled Trial" OR random* OR "RCT"OR "RCTs" OR "pragmatic clinical trial*" OR "practical clinical trial*" OR "non-inferiority trial*" OR "noninferiority trial*" OR "superiority trial*" OR "equivalence clinical trial*") OR ("Comparative Study" OR "comparison" OR "comparative" OR "compar*" OR "major clinical study" OR "clinical study" OR "case control study" OR "family study" OR "longitudinal study" OR "retrospective study" OR "prospective study" OR "cohort analysis" OR cohort* OR (("case control" NEAR1 (study OR studies))) OR (("follow up" NEAR1 (study OR studies))) OR (observational NEAR1 (study OR studies)) OR ((epidemiologic NEAR1 (study OR studies))) OR (("cross sectional" NEAR1 (study OR studies)))) OR ("life cycle assess*" OR "life cycle assessment" OR "life cycle inventory" OR "LCA" OR "LCAs" OR "life cycle inventory" OR "life cycle inventories")) OR AB=(*("Meta-Analysis" OR metaanaly* OR "meta-analy*" or metanaly* OR "Systematic Review" OR "Cochrane Database Syst Rev" OR "prisma" OR "preferred reporting items" OR "prospero" OR ((systemati* OR scoping OR umbrella OR "structured literature") NEAR4 (review* OR overview*)) OR "systematic review*" OR "scoping review*" OR "umbrella review*" OR "structured literature review*" OR "systematic qualitative review*" OR "systematic quantitative review*" OR "systematic search and review" OR "systematized review" OR "systematised review" OR "systemic review" OR "systematic literature review*" OR "systematic integrative literature review*" OR "systematically review*" OR "scoping literature review*" OR "systematic critical review" OR "systematic integrative review*" OR "systematic evidence review" OR "Systematic integrative literature review*" OR "Systematic mixed studies review*" OR "Systematized literature review*" OR "Systematic overview*" OR "Systematic narrative review*" OR (("systemati*" OR "literature" OR "database*" OR "data-base*" OR "structured" OR "comprehensive*" OR "systemic*") NEAR4 "search*") OR ("Literature" AND "review" AND ("database*" OR "data-base*" OR "search*")) OR (("data extraction" OR "data source*") AND "study selection") OR ("search strategy" AND "selection criteria") OR ("data source*" AND "data synthesis") OR "medline" OR "pubmed" OR "embase" OR "Cochrane" OR (("critical" OR "rapid") NEAR4 ("review*" OR "overview*" OR "synthes*")) OR ((("critical*" OR "rapid*") NEAR4 ("review*" OR "overview*" OR "synthes*") NEAR4 ("search*" OR "database*" OR "data-base*"))) OR metasynthes* OR "meta-synthes*")* OR ("Randomized Controlled Trial" OR random* OR "RCT"OR "RCTs" OR "pragmatic clinical trial*" OR "practical clinical trial*" OR "non-inferiority trial*" OR "noninferiority trial*" OR "superiority trial*" OR "equivalence clinical trial*") OR ("Comparative Study" OR "comparison" OR "comparative" OR "compar*" OR "major clinical study" OR "clinical study" OR "case control study" OR "family study" OR "longitudinal study" OR "retrospective study" OR "prospective study" OR "cohort analysis" OR cohort* OR (("case control" NEAR1 (study OR studies))) OR (("follow up" NEAR1 (study OR studies))) OR (observational NEAR1 (study OR studies)) OR ((epidemiologic NEAR1 (study OR studies))) OR (("cross sectional" NEAR1 (study OR studies)))) OR ("life cycle assess*" OR "life cycle assessment" OR "life cycle inventory" OR "LCA" OR "LCAs" OR "life cycle inventory" OR "life cycle inventories"))**)**

**UV4.3**

((exp "Local Anesthesia"/ OR exp "local anesthetic agent"/ OR exp "Nerve Block"/ OR "Local Anesthesia".mp OR "Local Anaesthesia".mp OR "Locoregional Anesthesia".mp OR "Locoregional Anaesthesia".mp OR "Local Anesthetics".mp OR "Local Anaesthetics".mp OR "Locoregional Anesthetics".mp OR "Locoregional Anaesthetics".mp OR "Local Anesthetic".mp OR "Local Anaesthetic".mp OR "Locoregional Anesthetic".mp OR "Locoregional Anaesthetic".mp OR "axillary block".mp OR "axillary block*".mp OR "Bier block".mp OR "Bier block*".mp OR "Bier's block".mp OR "Bier's block*".mp OR "brachial plexus block".mp OR "brachial plexus block*".mp OR "combined spinal epidural".mp OR "combined spinal epidural block".mp OR "combined spinal epidural block*".mp OR "epidural anaesthesia".mp OR "epidural anesthesia".mp OR "intravenous regional anesthaesia".mp OR "intravenous regional anesthesia".mp OR "ischiadic block".mp OR "ischiadic block*".mp OR "local infiltration".mp OR "lower extremity block".mp OR "lower extremity block*".mp OR "nerve block".mp OR "nerve block".mp OR "nerve block*".mp OR "nerve block*".mp OR "neuraxial anaesthesia".mp OR "neuraxial anesthesia".mp OR "neuraxial block".mp OR "neuraxial block*".mp OR "neuraxial technique".mp OR "neuraxial techniques".mp OR "peripheral nerve block".mp OR "peripheral nerve block*".mp OR "plexus block".mp OR "plexus block*".mp OR "plexus nerve block".mp OR "plexus nerve block*".mp OR "popliteal block".mp OR "popliteal block*".mp OR "regional anaesthesia".mp OR "regional anesthesia".mp OR "spinal anaesthesia".mp OR "spinal anaesthetic block".mp OR "spinal anaesthetic block*".mp OR "spinal anesthesia".mp OR "spinal anesthetic block".mp OR "spinal anesthetic block*".mp OR "supraclavicular block".mp OR "supraclavicular block*".mp OR "upper extremity block".mp OR "upper extremity block*".mp) AND (exp "General Anesthesia"/ OR "general anesthesia".mp OR "general anaesthesia".mp OR "general anesthe*".mp OR "general anaesthe*".mp OR exp "inhalation anesthetic agent"/ OR exp "inhalation anesthesia"/ OR "Closed-Circuit Anesthesia".mp OR "Endotracheal Anesthesia".mp OR "Closed-Circuit Anaesthesia".mp OR "Endotracheal Anaesthesia".mp OR "Halothane"/ OR "Isoflurane"/ OR "Methoxyflurane"/ OR "Sevoflurane"/ OR "Anaesthetic gas".mp OR "Anaesthetic gases".mp OR "Anesthetic gas".mp OR "Anesthetic gases".mp OR "Desflurane".mp OR "Enflurane".mp OR "Flurane".mp OR "Fluranes".mp OR "Gaseous anaesthesia".mp OR "Gaseous anaesthetic".mp OR "Gaseous anaesthetics".mp OR "Gaseous anesthesia".mp OR "Gaseous anesthetic".mp OR "Gaseous anesthetics".mp OR "Halothane".mp OR "Inhalation anaesthesia".mp OR "inhalation anaesthetic".mp OR "inhalation anaesthetics".mp OR "Inhalation anesthesia".mp OR "inhalation anesthetic".mp OR "inhalation anesthetics".mp OR "Inhalational agents".mp OR "Inhalational anaesthesia".mp OR "Inhalational anaesthetic".mp OR "Inhalational anaesthetic agent".mp OR "Inhalational anaesthetic agents".mp OR "Inhalational anaesthetics".mp OR "Inhalational anesthesia".mp OR "Inhalational anesthetic".mp OR "Inhalational anesthetic agent".mp OR "Inhalational anesthetic agents".mp OR "Inhalational anesthetics".mp OR "Inhaled anaesthetic".mp OR "Inhaled anaesthetic agent".mp OR "Inhaled anaesthetic agents".mp OR "Inhaled anaesthetics".mp OR "Inhaled anesthetic".mp OR "Inhaled anesthetic agents".mp OR "Inhaled anesthetics".mp OR "insufflation anaesthesia".mp OR "insufflation anaesthetic".mp OR "insufflation anaesthetics".mp OR "insufflation anesthesia".mp OR "insufflation anesthetic".mp OR "insufflation anesthetics".mp OR "Isoflurane".mp OR "Methoxyflurane".mp OR "Sevoflurane".mp OR "Vapor anaesthesia".mp OR "Vapor anaesthetic".mp OR "Vapor anaesthetics".mp OR "Vapor anesthesia".mp OR "Vapor anesthetic".mp OR "Vapor anesthetics".mp OR "Volatile agent".mp OR "Volatile agents".mp OR "Volatile Anaesthetic".mp OR "Volatile Anaesthetics".mp OR "Volatile anesthetic".mp OR "Volatile anesthetics".mp OR "Volatile drug".mp OR "Volatile drugs".mp OR "Volatile fluorinated liquid".mp OR "Volatile fluorinated liquids".mp OR "Volatile gas".mp OR "Volatile gases".mp OR "Volatile liquid agent".mp OR "Volatile liquid agents".mp OR "Anesthetic Gas".mp OR "Anesthetic Gases".mp OR "Inhalation Anaesthetic".mp OR "Inhalation Anaesthetics".mp OR "Inhalation Anesthetic".mp OR "Inhalation Anesthetics".mp OR exp "intravenous anesthetic agent"/ OR exp "Intravenous Anesthesia"/ OR "Intravenous Anesthetics".mp OR "Intravenous Anaesthetics".mp OR "Intravenous Anesthetic".mp OR "Intravenous Anaesthetic".mp OR "Intravenous Anesthesia".mp OR "Intravenous Anaesthesia".mp OR "Dissociative Anesthetics".mp OR "Dissociative Anaesthetics".mp OR "Dissociative Anesthetic".mp OR "Dissociative Anaesthetic".mp OR "Dissociative Anesthesia".mp OR "Dissociative Anaesthesia".mp OR "2-(3-methoxyphenyl)-2-(ethylamino)cyclohexanone"/ OR "2-Oxo-PCE"/ OR "Alfentanil"/ OR "Chloralose"/ OR "Diazepam"/ OR "Etomidate"/ OR "Fentanyl"/ OR "Ketamine"/ OR "Methohexital"/ OR "Midazolam"/ OR "Propanidid"/ OR "Propofol"/ OR "Sodium Oxybate"/ OR "Sufentanil"/ OR "Thiamylal"/ OR "Thiopental"/ OR "Tiletamine"/ OR "Urethane"/ OR "2-(3-methoxyphenyl)-2-(ethylamino)cyclohexanone".mp OR "2-Oxo-PCE".mp OR "Alfentanil".mp OR "Chloralose".mp OR "Diazepam".mp OR "Etomidate".mp OR "Fentanyl".mp OR "intravenous anaesthesia".mp OR "intravenous anaesthesias".mp OR "Intravenous anaesthetic".mp OR "Intravenous anaesthetic agent".mp OR "Intravenous anaesthetic agents".mp OR "Intravenous anaesthetic drug".mp OR "Intravenous anaesthetic drugs".mp OR "Intravenous anaesthetics".mp OR "intravenous anesthesia".mp OR "intravenous anesthesias".mp OR "intravenous anesthetic".mp OR "Intravenous anesthetic agents".mp OR "Intravenous anesthetic agents".mp OR "Intravenous anesthetic drug".mp OR "Intravenous anesthetic drugs".mp OR "intravenous anesthetics".mp OR "IV anaesthesia".mp OR "IV anaesthetics".mp OR "IV anaesthetics".mp OR "IV anesthesia".mp OR "IV anesthetic".mp OR "IV anesthetics".mp OR "Ketamine".mp OR "Methohexital".mp OR "Midazolam".mp OR "Propanidid".mp OR "Propofol".mp OR "Sodium Oxybate".mp OR "Sufentanil".mp OR "Thiamylal".mp OR "Thiopental".mp OR "Tiletamine".mp OR "TIVA".mp OR "Total intravenous anaesthesia".mp OR "Total Intravenous anesthesia".mp OR "Urethane".mp)) AND ("Carbon Footprint"/ OR "carbon footprint".mp OR "carbon footprint*".mp OR exp "Climate Change"/ OR "climate change".mp OR "CO2 emission".mp OR "CO2 emissions".mp OR "CO2 footprint".mp OR "CO2 footprint*".mp OR exp "environmental protection"/ OR "conservation of natural resources".mp OR "environmental protection".mp OR "Disposable Equipment"/ OR "Disposables".mp OR "Disposable".mp OR "ecological footprint".mp OR "ecological footprint*".mp OR "ecological sustainability".mp OR exp "environmental impact"/ OR "environmental impact".mp OR "environmental impact*".mp OR "environmental impacts".mp OR "environmental pollut*".mp OR exp "pollution"/ OR "environmental pollution".mp OR "environmental sustainab*".mp OR "environmental sustainability"/ OR "environmental sustainability".mp OR "Global Warming"/ OR "Global Warming".mp OR "Greenhouse Effect"/ OR "greenhouse effect*".mp OR "greenhouse effects".mp OR "greenhouse gas emission".mp OR "greenhouse gas emissions".mp OR "Greenhouse Gas"/ OR "greening".mp OR "hospital waste".mp OR "life cycle assessment"/ OR "environmental impact assessment"/ OR "life cycle assess*".mp OR "life cycle assessment".mp OR "LCA".mp OR "LCAs".mp OR "life cycle inventory".mp OR "life cycle inventories".mp OR exp "Waste Disposal"/ OR exp "Hospital Waste"/ OR "medical waste".mp OR "Rising Sea Level".mp OR "Rising Sea Levels".mp OR "Sea Level Rise"/ OR "Sea Level Rise".mp OR "sustainability".ti OR "Waste Disposal".mp OR "waste water recycling"/ OR "Recycling"/ OR "recycling".mp OR "recycle*".mp OR "Equipment reuse".mp OR "Reusables".mp OR "reusable".mp OR "reuse".mp OR "reused".mp OR "reusing".mp OR exp "Waste Disposal"/ OR exp "Waste Management"/ OR "Plastic overuse".mp OR "Green surgery".mp OR "Emission reduction".mp OR "Emission reduction strategy".mp OR "air pollution control"/ OR "air pollution control".mp OR "Environment"/ OR "Environmental*".ti OR "acidification"/ OR "soil acidification"/ OR "ocean acidification"/ OR "acidification".mp OR "Acidification potential".mp OR "AP in kg SO2 equivalents".mp OR "eco-efficiency".mp OR "ecoefficiency".mp OR "eco-efficien*".mp OR "ecoefficien*".mp OR "ecotoxicity"/ OR "ecotoxicity".mp OR "ecotoxic*".mp OR "eco toxicity".mp OR "eco toxic*".mp OR "EP in kg PO4 equivalent".mp OR exp "Eutrophication"/ OR "eutrophication".mp OR "Eutrophication potential".mp OR "FAETP in kg DCB equivalent".mp OR "Freshwater Aquatic Ecotoxicity Potential".mp OR "GWP in kg CO2 equivalents".mp OR "H+ moles equivalents".mp OR "HTTP in kg Dichlorobenzene equivalent".mp OR "Human Toxicity Potential".mp OR "kg 2.4-D equivalents".mp OR "kg CFC-11 equivalent".mp OR "kg N equivalents".mp OR "kg NOx equivalents".mp OR "life cycle analysis".mp OR "ozone depletion".mp OR "Photochemical Ozone Depletion Potential".mp OR "POCP in kg ethane equivalent".mp OR "smog".mp OR exp "Waste"/ OR "waste".mp OR "wastes".mp OR "Ozone Depletion"/ OR "Smog"/ OR "Equipment reuse".mp OR "Greenhouse Gases".mp OR "Greenhouse Gas".mp OR "SO2 equiv*".mp OR "CO2 equiva*".mp OR "CFC-11 equiv*".mp OR "N equiv*".mp OR exp "Biodiversity"/ OR "Biodiversity".mp OR "Climatic change".mp OR "Green deal".mp OR "preservation of natural resources".mp OR "Refuse Disposal".mp OR exp "Wastewater"/ OR "Waste Water".mp OR "Wastewater".mp OR exp "Water Management"/ OR "Water Purification".mp OR (("plastic*".mp OR "microplastic*".mp) AND ("soup".mp OR "pollution".mp OR "overuse".mp OR "contamination".mp)) OR "Sustainable Development"/ OR "Sustainable Development".mp OR (("Plastic".mp OR "plastics".mp) AND "overuse".mp) OR ("hydrogen*".mp AND "moles".mp AND "equiv*".mp) OR ("Dichlorobenzen*".mp AND "equiv*".mp) OR ("2,4-D".mp AND "equiv*".mp) OR ("NOx".mp AND "equiv*".mp) OR ("ethane".mp AND "equiv*".mp) OR ("PO4".mp AND "equiv*".mp) OR ("DCB".mp AND "equiv*".mp) OR ("sustainability".mp AND ("environment*".mp OR "carbon".mp)) OR (("Carbon Dioxide"/ OR "Carbon Dioxide".mp OR "CO2".mp) AND ("pollution".mp OR "emission".mp OR "emissions".mp OR "waste".mp OR "environment".mp OR "environmental*".mp OR "footprint".mp OR "footprint*".mp OR "sustainable".mp OR "hazard".mp OR "hazard*".mp))) NOT (conference review or conference abstract).pt AND (2000 OR 2001 OR 2002 OR 2003 OR 2004 OR 2005 OR 2006 OR 2007 OR 2008 OR 2009 OR 2010 OR 2011 OR 2012 OR 2013 OR 2014 OR 2015 OR 2016 OR 2017 OR 2018 OR 2019 OR 2020 OR 2021 OR 2022).yr AND (*(exp "meta analysis"/ OR exp "meta analysis (topic)"/ OR metaanaly*.ti,ab OR "meta analy*".ti,ab OR metanaly*.ti,ab OR "systematic review"/ OR "cochrane database of systematic reviews".jn OR prisma.ti,ab OR prospero.ti,ab OR (((systemati* OR scoping OR umbrella OR "structured literature") ADJ3 (review* OR overview*)).ti,ab) OR ((systemic* ADJ1 review*).ti,ab) OR (((systemati* OR literature OR database* OR "data base*") ADJ10 search*).ti,ab) OR (((structured OR comprehensive* OR systemic*) ADJ3 search*).ti,ab) OR (((literature ADJ3 review*).ti,ab) AND (search*.ti,ab OR database*.ti,ab OR "data base*".ti,ab)) OR (("data extraction".ti,ab OR "data source*".ti,ab) AND "study selection".ti,ab) OR ("search strategy".ti,ab AND "selection criteria".ti,ab) OR ("data source*".ti,ab AND "data synthesis".ti,ab) OR medline.ab OR pubmed.ab OR embase.ab OR cochrane.ab OR (((critical OR rapid) ADJ2 (review* OR overview* OR synthes*)).ti) OR ((((critical* OR rapid*) ADJ3 (review* OR overview* OR synthes*)).ab) AND (search*.ab OR database*.ab OR "data base*".ab)) OR metasynthes*.ti,ab OR "meta synthes*".ti,ab)* OR (exp "clinical trial"/ OR exp "randomization"/ OR exp "single blind procedure"/ OR exp "double blind procedure"/ OR exp "crossover procedure"/ OR exp "placebo"/ OR exp "prospective study"/ OR rct.ti,ab OR random*.ti,ab OR "single blind".ti,ab OR "randomised controlled trial".ti,ab OR exp "randomized controlled trial"/ OR placebo*.ti,ab) OR (exp "Comparative Study"/ OR "comparison".ti,ab OR "comparative".ti,ab OR "compar*".ti,ab OR "major clinical study"/ OR "clinical study"/ OR "case control study"/ OR "family study"/ OR "longitudinal study"/ OR "retrospective study"/ OR "prospective study"/ OR "cohort analysis"/ OR cohort*.ti,ab OR (("case control" ADJ1 (study OR studies)).ti,ab) OR (("follow up" ADJ1 (study OR studies)).ti,ab) OR (observational ADJ1 (study OR studies)).ti,ab OR ((epidemiologic ADJ1 (study OR studies)).ti,ab) OR (("cross sectional" ADJ1 (study OR studies)).ti,ab)) OR ("life cycle assessment"/ OR "environmental impact assessment"/ OR "life cycle assess*".mp OR "life cycle assessment".mp OR "life cycle inventory".mp OR "LCA".mp OR "LCAs".mp OR "life cycle inventory".mp OR "life cycle inventories".mp))

### Topic 5: Operating room air ventilation

**PubMed**

("Operating Rooms"[Mesh] OR "operating room"[tw] OR "operating rooms"[tw] OR "operation room"[tw] OR "operation rooms"[tw] OR "operating theatre"[tw] OR "operating theatres"[tw] OR "operation theatre"[tw] OR "operation theatres"[tw] OR "operating theater"[tw] OR "operating theaters"[tw] OR "operation theater"[tw] OR "operation theaters"[tw] OR "surgery room"[tw] OR "surgery rooms"[tw] OR "operation room"[tw] OR "operation rooms"[tw] OR "surgery theatre"[tw] OR "surgery theatres"[tw] OR "operation theatre"[tw] OR "operation theatres"[tw] OR "surgery theater"[tw] OR "surgery theaters"[tw] OR "operation theater"[tw] OR "operation theaters"[tw] OR "surgical room"[tw] OR "surgical rooms"[tw] OR "operation room"[tw] OR "operation rooms"[tw] OR "surgical theatre"[tw] OR "surgical theatres"[tw] OR "operation theatre"[tw] OR "operation theatres"[tw] OR "surgical theater"[tw] OR "surgical theaters"[tw] OR "operation theater"[tw] OR "operation theaters"[tw] OR "operating department"[tw] OR "operating departments"[tw] OR ("operation"[ti] AND ("department"[ti] OR "departments"[ti])) OR "surgical suite"[tw] OR "surgical suites"[tw] OR "surgery suite"[tw] OR "surgery suites"[tw] OR "operation suite"[tw] OR "operation suites"[tw] OR "operating suite"[tw] OR "operating suites"[tw]) AND ("Ventilation"[Mesh] OR "ventilation"[tw] OR "ventilat*"[tw] OR "ventilation system"[tw] OR "ventilation systems"[tw] OR "air system"[tw] OR "air systems"[tw] OR "Air Conditioning"[Mesh] OR "Air Conditioning"[tw] OR "Air Condition*"[tw] OR "HVAC"[tw] OR "Heating Ventilation and Air-Conditioning"[tw] OR "Heating, Ventilation and Air-Conditioning"[tw] OR "hepa-filter"[tw] OR "hepafilter"[tw] OR "hepa filters"[tw] OR "hepafilters"[tw] OR "hepa filtration"[tw] OR "hepafiltration"[tw] OR "high efficiency particulate air"[tw] OR "Air Filters"[Mesh] OR "Air Filters"[tw] OR "Air Filter"[tw] OR "Airfilters"[tw] OR "Airfilter"[tw] OR "Air Filt*"[tw] OR "Airfilt*"[tw] OR "unidirectional downflow system"[tw] OR "unidirectional downflow"[tw] OR "unidirectional down flow"[tw] OR "uni directional down flow"[tw] OR "laminair downflow"[tw] OR "laminair down flow"[tw] OR "laminar downflow"[tw] OR "laminar down flow"[tw] OR "plenum"[tw] OR "opragon"[tw] OR "halton"[tw] OR "3 zones"[tw] OR "three zones"[tw] OR "3 zone"[tw] OR "three zone"[tw] OR "Temperature 18-23 degrees Celsius"[tw] OR (("Temperature"[mesh] OR "temperature"[tw] OR "temperatures"[tw]) AND ("18"[tw] OR "19"[tw] OR "20"[tw] OR "21"[tw] OR "22"[tw] OR "23"[tw]) AND ("celsius"[tw] OR "degree c"[tw] OR "degrees c"[tw])) OR "Humidity"[mesh] OR "humidity"[tw] OR "humid"[tw] OR "recovery time"[tw] OR "recovery times"[tw] OR "NEN 14644-3"[tw] OR "ISO 14644-3"[tw] OR "14644-3"[tw] OR "air change"[tw] OR "air changes"[tw] OR "air quality ISO 7"[tw] OR "air quality"[tw] OR "ISO 7"[tw] OR "NEN 14644-1"[tw] OR "ISO 14644-1"[tw] OR "14644-1"[tw] OR "146441"[tw] OR "2 zones"[tw] OR "2 zone"[tw] OR "two zones"[tw] OR "two zone"[tw] OR "mixing ventilation airflow"[tw] OR "MAF"[tw] OR "Mixing ventilation air flow"[tw] OR "mixing ventilation"[tw] OR (("ventilation"[tw] OR "ventilat*"[tw] OR "airflow"[tw] OR "air flow"[tw]) AND ("mixed"[tw] OR "mixing"[tw] OR "turbulent"[tw])) OR "conventional airflow"[tw] OR "conventional air flow"[tw] OR "ceiling diffusers"[tw] OR "ceiling diffuser"[tw] OR "ceiling diffus*"[tw] OR "circulatory system"[tw] OR "circulatory systems"[tw] OR "laminar flow"[tw] OR "unidirectional flow"[tw] OR "laminar-air-flow"[tw] OR "laminar airflow"[tw] OR "laminar flow*"[tw] OR "laminar-air-flow*"[tw] OR "laminar airflow*"[tw] OR "laminar air"[tw] OR "conventional ventilation"[tw] OR "ultraclean ventilation"[tw] OR "ultraclean air"[tw] OR "conventional ventilat*"[tw] OR "ultraclean air"[tw] OR "ultra clean ventilation"[tw] OR "ultra clean air"[tw] OR "ultraclean ventilat*"[tw] OR "ultraclean air*"[tw] OR "ultra clean ventilat*"[tw] OR "ultra clean air*"[tw] OR "clean-airflow"[tw] OR "clean-airflow*"[tw] OR "partial unidirectional airflow"[tw] OR "partial uni directional airflow"[tw] OR "partial unidirectional flow"[tw] OR "partial uni directional flow"[tw] OR "P-UDAF"[tw] OR "PUDAF"[tw] OR "unidirectional airflow"[tw] OR "uni directional airflow"[tw] OR "unidirectional airflow*"[tw] OR "uni directional airflow*"[tw] OR "unidirectional air*"[tw] OR "uni directional air*"[tw] OR "unidirectional flow*"[tw] OR "uni directional flow*"[tw] OR "displacement ventilation"[tw] OR "plenum ventilated"[tw] OR "plenum-ventilated"[tw] OR "plenum ventilation"[tw] OR "horizontal ventilation"[tw] OR "vertical ventilation"[tw]) AND ("Acidification potential"[tw] OR "acidification"[tw] OR "air pollution control"[tw] OR "AP in kg SO2 equivalents"[tw] OR "Biodiversity"[Mesh] OR "Biodiversity"[tw] OR "Carbon Footprint"[mesh] OR "carbon footprint"[tw] OR "carbon footprint*"[tw] OR "CFC-11 equiv*"[tw] OR "Climate Change"[Mesh] OR "climate change"[tw] OR "Climatic change"[tw] OR "CO2 emission"[tw] OR "CO2 emissions"[tw] OR "CO2 equiva*"[tw] OR "CO2 footprint"[tw] OR "CO2 footprint*"[tw] OR "conservation of natural resources"[mesh] OR "conservation of natural resources"[tw] OR "Disposable Equipment"[Mesh] OR "Disposable"[tw] OR "Disposables"[tw] OR "eco toxic*"[tw] OR "eco toxicity"[tw] OR "ecoefficien*"[tw] OR "eco-efficien*"[tw] OR "ecoefficiency"[tw] OR "eco-efficiency"[tw] OR "ecological footprint"[tw] OR "ecological footprint*"[tw] OR "ecological sustainability"[tw] OR "ecotoxic*"[tw] OR "ecotoxicity"[tw] OR "Emission reduction strategy"[tw] OR "Emission reduction"[tw] OR "Environment"[Mesh:noexp] OR "environmental impact"[tw] OR "environmental impact*"[tw] OR "environmental impacts"[tw] OR "environmental pollut*"[tw] OR "Environmental Pollution"[Mesh] OR "environmental pollution"[tw] OR "environmental protection"[tw] OR "environmental sustainab*"[tw] OR "environmental sustainability"[tw] OR "Environmental*"[ti] OR "EP in kg PO4 equivalent"[tw] OR "Equipment reuse"[mesh] OR "Equipment reuse"[tw] OR "Eutrophication potential"[tw] OR "Eutrophication"[Mesh] OR "eutrophication"[tw] OR "FAETP in kg DCB equivalent"[tw] OR "Freshwater Aquatic Ecotoxicity Potential"[tw] OR "Global Warming"[mesh] OR "Global Warming"[tw] OR "Green deal"[tw] OR "Green surgery"[tw] OR "Greenhouse Effect"[mesh] OR "greenhouse effect*"[tw] OR "greenhouse effects"[tw] OR "greenhouse gas emission"[tw] OR "greenhouse gas emissions"[tw] OR "Greenhouse Gas"[tw] OR "Greenhouse Gases"[mesh] OR "Greenhouse Gases"[tw] OR "greening"[tw] OR "GWP in kg CO2 equivalents"[tw] OR "H+ moles equivalents"[tw] OR "hospital waste"[tw] OR "HTTP in kg Dichlorobenzene equivalent"[tw] OR "Human Toxicity Potential"[tw] OR "kg 2.4-D equivalents"[tw] OR "kg CFC-11 equivalent"[tw] OR "kg N equivalents"[tw] OR "kg NOx equivalents"[tw] OR "LCA"[tw] OR "LCAs"[tw] OR "life cycle analysis"[tw] OR "life cycle assess*"[tw] OR "life cycle assessment"[tw] OR "life cycle inventories"[tw] OR "life cycle inventory"[tw] OR "Medical Waste Disposal"[mesh] OR "Medical Waste"[mesh] OR "medical waste"[tw] OR "N equiv*"[tw] OR "Ozone Depletion"[Mesh] OR "ozone depletion"[tw] OR "Photochemical Ozone Depletion Potential"[tw] OR "Plastic overuse"[tw] OR "POCP in kg ethane equivalent"[tw] OR "preservation of natural resources"[tw] OR "recycle*"[tw] OR "Recycling"[mesh] OR "recycling"[tw] OR "Refuse Disposal"[Mesh] OR "Refuse Disposal"[tw] OR "reusable"[tw] OR "Reusables"[tw] OR "reuse"[tw] OR "reused"[tw] OR "reusing"[tw] OR "Rising Sea Level"[tw] OR "Rising Sea Levels"[tw] OR "Sea Level Rise"[mesh] OR "Sea Level Rise"[tw] OR "Smog"[mesh] OR "smog"[tw] OR "SO2 equiv*"[tw] OR "sustainability"[ti] OR "Sustainable Development"[Mesh] OR "Sustainable Development"[tw] OR "Waste Disposal"[tw] OR "Waste Disposal, Fluid"[mesh] OR "Waste Management"[mesh] OR "Waste"[tw] OR "waste"[tw] OR "Waste Water"[Mesh] OR "Waste Water"[tw] OR "wastes"[tw] OR "Wastewater"[tw] OR "Water Purification"[Mesh] OR "Water Purification"[tw] OR (("plastic*"[tw] OR "microplastic*"[tw]) AND ("soop"[tw] OR "soup"[tw] OR "pollution"[tw] OR "overuse"[tw] OR "contamination"[tw])) OR (("Plastic"[tw] OR "plastics"[tw]) AND "overuse"[tw]) OR ("hydrogen*"[tw] AND "moles"[tw] AND "equiv*"[tw]) OR ("Dichlorobenzen*"[tw] AND "equiv*"[tw]) OR ("2,4-D"[tw] AND "equiv*"[tw]) OR ("NOx"[tw] AND "equiv*"[tw]) OR ("ethane"[tw] AND "equiv*"[tw]) OR ("PO4"[tw] AND "equiv*"[tw]) OR ("DCB"[tw] AND "equiv*"[tw]) OR ("sustainability"[tw] AND ("environment*"[tw] OR "carbon"[tw])) OR (("Carbon Dioxide"[mesh] OR "Carbon Dioxide"[tw] OR "CO2"[tw]) AND ("pollution"[tw] OR "emission"[tw] OR "emissions"[tw] OR "waste"[tw] OR "environment"[tw] OR "environmental*"[tw] OR "footprint"[tw] OR "footprint*"[tw] OR "sustainable"[tw] OR "hazard"[tw] OR "hazard*"[tw]))) AND ("1980/01/01"[PDAT] : "3000/12/31"[PDAT]) AND (*("Meta-Analysis"[Publication Type] OR "Meta-Analysis as Topic"[Mesh] OR metaanaly*[tiab] OR meta-analy*[tiab] or metanaly*[tiab] OR "Systematic Review"[Publication Type] OR systematic[sb] OR "Cochrane Database Syst Rev"[Journal] or prisma[tiab] OR preferred reporting items[tiab] OR prospero[tiab] OR ((systemati*[ti] OR scoping[ti] OR umbrella[ti] OR structured literature[ti]) AND (review*[ti] OR overview*[ti])) OR systematic review*[tiab] OR scoping review*[tiab] OR umbrella review*[tiab] OR structured literature review*[tiab] OR systematic qualitative review*[tiab] OR systematic quantitative review*[tiab] OR systematic search and review[tiab] OR systematized review[tiab] OR systematised review[tiab] OR systemic review[tiab] OR systematic literature review*[tiab] OR systematic integrative literature review*[tiab] OR systematically review*[tiab] OR scoping literature review*[tiab] OR systematic critical review[tiab] OR systematic integrative review*[tiab] OR systematic evidence review[tiab] OR Systematic integrative literature review*[tiab] OR Systematic mixed studies review*[tiab] OR Systematized literature review*[tiab] OR Systematic overview*[tiab] OR Systematic narrative review*[tiab] OR ((systemati*[tiab] OR literature[tiab] OR database*[tiab] OR data-base*[tiab] OR structured[tiab] OR comprehensive*[tiab] OR systemic*[tiab]) AND search*[tiab]) OR (Literature[ti] AND review[ti] AND (database*[tiab] OR data-base*[tiab] OR search*[tiab])) OR ((data extraction[tiab] OR data source*[tiab]) AND study selection[tiab]) OR (search strategy[tiab] AND selection criteria[tiab]) OR (data source*[tiab] AND data synthesis[tiab]) OR medline[tiab] OR pubmed[tiab] OR embase[tiab] OR Cochrane[tiab] OR ((critical[ti] OR rapid[ti]) AND (review*[ti] OR overview*[ti] OR synthes*[ti])) OR (((critical*[tiab] OR rapid*[tiab]) AND (review*[tiab] OR overview*[tiab] OR synthes*[tiab]) AND (search*[tiab] OR database*[tiab] OR data-base*[tiab]))) OR metasynthes*[tiab] OR meta-synthes*[tiab])* OR ("Randomized Controlled Trial"[Publication Type] OR random*[tiab] OR pragmatic clinical trial*[tiab] OR practical clinical trial*[tiab] OR non-inferiority trial*[tiab] OR noninferiority trial*[tiab] OR superiority trial*[tiab] OR equivalence clinical trial*[tiab]) NOT (("Animals"[Mesh]) OR "Models, Animal"[Mesh] NOT humans[mh]) NOT (letter[pt] OR comment[pt] OR editorial[pt]) OR ("Comparative Study"[Publication Type] OR "comparison"[tiab] OR "comparative"[tiab] OR "compar*"[tiab] OR "Epidemiologic studies"[mesh:noexp] OR "case control studies"[mesh] OR "cohort studies"[mesh] OR "Controlled Before-After Studies"[mesh] OR "Case control"[tw] OR cohort*[tw] OR "Cohort analy*"[tw] OR "Follow up stud*"[tw] OR "observational stud*"[tw] OR Longitudinal[tw] OR Retrospective*[tw] OR prospective*[tw] OR consecutive*[tw] OR Cross sectional[tw] OR "Cross-sectional studies"[mesh] OR "historically controlled study"[mesh] OR "interrupted time series analysis"[mesh]) OR ("life cycle assess*"[tw] OR "life cycle assessment"[tw] OR "life cycle inventory"[tw] OR "LCA"[tw] OR "LCAs"[tw] OR "life cycle inventory"[tw] OR "life cycle inventories"[tw]))

**Embase**

(exp "Operating Room"/ OR "operating room".mp OR "operating rooms".mp OR "operation room".mp OR "operation rooms".mp OR "operating theatre".mp OR "operating theatres".mp OR "operation theatre".mp OR "operation theatres".mp OR "operating theater".mp OR "operating theaters".mp OR "operation theater".mp OR "operation theaters".mp OR "surgery room".mp OR "surgery rooms".mp OR "operation room".mp OR "operation rooms".mp OR "surgery theatre".mp OR "surgery theatres".mp OR "operation theatre".mp OR "operation theatres".mp OR "surgery theater".mp OR "surgery theaters".mp OR "operation theater".mp OR "operation theaters".mp OR "surgical room".mp OR "surgical rooms".mp OR "operation room".mp OR "operation rooms".mp OR "surgical theatre".mp OR "surgical theatres".mp OR "operation theatre".mp OR "operation theatres".mp OR "surgical theater".mp OR "surgical theaters".mp OR "operation theater".mp OR "operation theaters".mp OR "operating department".mp OR "operating departments".mp OR ("operation".ti AND ("department".ti OR "departments".ti)) OR "surgical suite".mp OR "surgical suites".mp OR "surgery suite".mp OR "surgery suites".mp OR "operation suite".mp OR "operation suites".mp OR "operating suite".mp OR "operating suites".mp) AND ("room ventilation"/ OR "air conditioning"/ OR exp "ventilator"/ OR "ventilation".mp OR "ventilat*".mp OR "ventilation system".mp OR "ventilation systems".mp OR "air system".mp OR "air systems".mp OR "Air Conditioning".mp OR "Air Condition*".mp OR "HVAC".mp OR "Heating Ventilation and Air-Conditioning".mp OR "Heating, Ventilation and Air-Conditioning".mp OR "hepa-filter".mp OR "hepafilter".mp OR "hepa filters".mp OR "hepafilters".mp OR "hepa filtration".mp OR "hepafiltration".mp OR "high efficiency particulate air".mp OR exp "air filter"/ OR "Air Filters".mp OR "Air Filter".mp OR "Airfilters".mp OR "Airfilter".mp OR "Air Filt*".mp OR "Airfilt*".mp OR "unidirectional downflow system".mp OR "unidirectional downflow".mp OR "unidirectional down flow".mp OR "uni directional down flow".mp OR "laminair downflow".mp OR "laminair down flow".mp OR "laminar downflow".mp OR "laminar down flow".mp OR "plenum".mp OR "opragon".mp OR "halton".mp OR "3 zones".mp OR "three zones".mp OR "3 zone".mp OR "three zone".mp OR "Temperature 18-23 degrees Celsius".mp OR ((exp "Temperature"/ OR "temperature".mp OR "temperatures".mp) AND ("18".mp OR "19".mp OR "20".mp OR "21".mp OR "22".mp OR "23".mp) AND ("celsius".mp OR "degree c".mp OR "degrees c".mp)) OR exp "Humidity"/ OR "humidity".mp OR "humid".mp OR "recovery time".mp OR "recovery times".mp OR "NEN 14644-3".mp OR "ISO 14644-3".mp OR "14644-3".mp OR "air change".mp OR "air changes".mp OR "air quality ISO 7".mp OR "air quality".mp OR "ISO 7".mp OR "NEN 14644-1".mp OR "ISO 14644-1".mp OR "14644-1".mp OR "146441".mp OR "2 zones".mp OR "2 zone".mp OR "two zones".mp OR "two zone".mp OR "mixing ventilation airflow".mp OR "MAF".mp OR "Mixing ventilation air flow".mp OR "mixing ventilation".mp OR (("ventilation".mp OR "ventilat*".mp OR "airflow".mp OR "air flow".mp) AND ("mixed".mp OR "mixing".mp OR "turbulent".mp)) OR "airflow"/ OR "conventional airflow".mp OR "conventional air flow".mp OR "ceiling diffusers".mp OR "ceiling diffuser".mp OR "ceiling diffus*".mp OR "circulatory system".mp OR "circulatory systems".mp OR "laminar airflow"/ OR "laminar flow".mp OR "unidirectional flow".mp OR "laminar-air-flow".mp OR "laminar airflow".mp OR "laminar flow*".mp OR "laminar-air-flow*".mp OR "laminar airflow*".mp OR "laminar air".mp OR "conventional ventilation".mp OR "ultraclean ventilation".mp OR "ultraclean air".mp OR "conventional ventilat*".mp OR "ultraclean air".mp OR "ultra clean ventilation".mp OR "ultra clean air".mp OR "ultraclean ventilat*".mp OR "ultraclean air*".mp OR "ultra clean ventilat*".mp OR "ultra clean air*".mp OR "clean-airflow".mp OR "clean-airflow*".mp OR "partial unidirectional airflow".mp OR "partial uni directional airflow".mp OR "partial unidirectional flow".mp OR "partial uni directional flow".mp OR "P-UDAF".mp OR "PUDAF".mp OR "unidirectional airflow".mp OR "uni directional airflow".mp OR "unidirectional airflow*".mp OR "uni directional airflow*".mp OR "unidirectional air*".mp OR "uni directional air*".mp OR "unidirectional flow*".mp OR "uni directional flow*".mp OR "displacement ventilation".mp OR "plenum ventilated".mp OR "plenum-ventilated".mp OR "plenum ventilation".mp OR "horizontal ventilation".mp OR "vertical ventilation".mp) AND ("Carbon Footprint"/ OR "carbon footprint".mp OR "carbon footprint*".mp OR exp "Climate Change"/ OR "climate change".mp OR "CO2 emission".mp OR "CO2 emissions".mp OR "CO2 footprint".mp OR "CO2 footprint*".mp OR exp "environmental protection"/ OR "conservation of natural resources".mp OR "environmental protection".mp OR "Disposable Equipment"/ OR "Disposables".mp OR "Disposable".mp OR "ecological footprint".mp OR "ecological footprint*".mp OR "ecological sustainability".mp OR exp "environmental impact"/ OR "environmental impact".mp OR "environmental impact*".mp OR "environmental impacts".mp OR "environmental pollut*".mp OR exp "pollution"/ OR "environmental pollution".mp OR "environmental sustainab*".mp OR "environmental sustainability"/ OR "environmental sustainability".mp OR "Global Warming"/ OR "Global Warming".mp OR "Greenhouse Effect"/ OR "greenhouse effect*".mp OR "greenhouse effects".mp OR "greenhouse gas emission".mp OR "greenhouse gas emissions".mp OR "Greenhouse Gas"/ OR "greening".mp OR "hospital waste".mp OR "life cycle assessment"/ OR "environmental impact assessment"/ OR "life cycle assess*".mp OR "life cycle assessment".mp OR "LCA".mp OR "LCAs".mp OR "life cycle inventory".mp OR "life cycle inventories".mp OR exp "Waste Disposal"/ OR exp "Hospital Waste"/ OR "medical waste".mp OR "Rising Sea Level".mp OR "Rising Sea Levels".mp OR "Sea Level Rise"/ OR "Sea Level Rise".mp OR "sustainability".ti OR "Waste Disposal".mp OR "waste water recycling"/ OR "Recycling"/ OR "recycling".mp OR "recycle*".mp OR "Equipment reuse".mp OR "Reusables".mp OR "reusable".mp OR "reuse".mp OR "reused".mp OR "reusing".mp OR exp "Waste Disposal"/ OR exp "Waste Management"/ OR "Plastic overuse".mp OR "Green surgery".mp OR "Emission reduction".mp OR "Emission reduction strategy".mp OR "air pollution control"/ OR "air pollution control".mp OR "Environment"/ OR "Environmental*".ti OR "acidification"/ OR "soil acidification"/ OR "ocean acidification"/ OR "acidification".mp OR "Acidification potential".mp OR "AP in kg SO2 equivalents".mp OR "eco-efficiency".mp OR "ecoefficiency".mp OR "eco-efficien*".mp OR "ecoefficien*".mp OR "ecotoxicity"/ OR "ecotoxicity".mp OR "ecotoxic*".mp OR "eco toxicity".mp OR "eco toxic*".mp OR "EP in kg PO4 equivalent".mp OR exp "Eutrophication"/ OR "eutrophication".mp OR "Eutrophication potential".mp OR "FAETP in kg DCB equivalent".mp OR "Freshwater Aquatic Ecotoxicity Potential".mp OR "GWP in kg CO2 equivalents".mp OR "H+ moles equivalents".mp OR "HTTP in kg Dichlorobenzene equivalent".mp OR "Human Toxicity Potential".mp OR "kg 2.4-D equivalents".mp OR "kg CFC-11 equivalent".mp OR "kg N equivalents".mp OR "kg NOx equivalents".mp OR "life cycle analysis".mp OR "ozone depletion".mp OR "Photochemical Ozone Depletion Potential".mp OR "POCP in kg ethane equivalent".mp OR "smog".mp OR exp "Waste"/ OR "waste".mp OR "wastes".mp OR "Ozone Depletion"/ OR "Smog"/ OR "Equipment reuse".mp OR "Greenhouse Gases".mp OR "Greenhouse Gas".mp OR "SO2 equiv*".mp OR "CO2 equiva*".mp OR "CFC-11 equiv*".mp OR "N equiv*".mp OR exp "Biodiversity"/ OR "Biodiversity".mp OR "Climatic change".mp OR "Green deal".mp OR "preservation of natural resources".mp OR "Refuse Disposal".mp OR exp "Wastewater"/ OR "Waste Water".mp OR "Wastewater".mp OR exp "Water Management"/ OR "Water Purification".mp OR (("plastic*".mp OR "microplastic*".mp) AND ("soop".mp OR "soup".mp OR "pollution".mp OR "overuse".mp OR "contamination".mp)) OR "Sustainable Development"/ OR "Sustainable Development".mp OR (("Plastic".mp OR "plastics".mp) AND "overuse".mp) OR ("hydrogen*".mp AND "moles".mp AND "equiv*".mp) OR ("Dichlorobenzen*".mp AND "equiv*".mp) OR ("2,4-D".mp AND "equiv*".mp) OR ("NOx".mp AND "equiv*".mp) OR ("ethane".mp AND "equiv*".mp) OR ("PO4".mp AND "equiv*".mp) OR ("DCB".mp AND "equiv*".mp) OR ("sustainability".mp AND ("environment*".mp OR "carbon".mp)) OR (("Carbon Dioxide"/ OR "Carbon Dioxide".mp OR "CO2".mp) AND ("pollution".mp OR "emission".mp OR "emissions".mp OR "waste".mp OR "environment".mp OR "environmental*".mp OR "footprint".mp OR "footprint*".mp OR "sustainable".mp OR "hazard".mp OR "hazard*".mp))) NOT (conference review or conference abstract).pt AND 1980:2023.(sa_year) AND (*(exp "meta analysis"/ OR exp "meta analysis (topic)"/ OR metaanaly*.ti,ab OR "meta analy*".ti,ab OR metanaly*.ti,ab OR "systematic review"/ OR "cochrane database of systematic reviews".jn OR prisma.ti,ab OR prospero.ti,ab OR (((systemati* OR scoping OR umbrella OR "structured literature") ADJ3 (review* OR overview*)).ti,ab) OR ((systemic* ADJ1 review*).ti,ab) OR (((systemati* OR literature OR database* OR "data base*") ADJ10 search*).ti,ab) OR (((structured OR comprehensive* OR systemic*) ADJ3 search*).ti,ab) OR (((literature ADJ3 review*).ti,ab) AND (search*.ti,ab OR database*.ti,ab OR "data base*".ti,ab)) OR (("data extraction".ti,ab OR "data source*".ti,ab) AND "study selection".ti,ab) OR ("search strategy".ti,ab AND "selection criteria".ti,ab) OR ("data source*".ti,ab AND "data synthesis".ti,ab) OR medline.ab OR pubmed.ab OR embase.ab OR cochrane.ab OR (((critical OR rapid) ADJ2 (review* OR overview* OR synthes*)).ti) OR ((((critical* OR rapid*) ADJ3 (review* OR overview* OR synthes*)).ab) AND (search*.ab OR database*.ab OR "data base*".ab)) OR metasynthes*.ti,ab OR "meta synthes*".ti,ab)* OR (exp "clinical trial"/ OR exp "randomization"/ OR exp "single blind procedure"/ OR exp "double blind procedure"/ OR exp "crossover procedure"/ OR exp "placebo"/ OR exp "prospective study"/ OR rct.ti,ab OR random*.ti,ab OR "single blind".ti,ab OR "randomised controlled trial".ti,ab OR exp "randomized controlled trial"/ OR placebo*.ti,ab) OR (exp "Comparative Study"/ OR "comparison".ti,ab OR "comparative".ti,ab OR "compar*".ti,ab OR "major clinical study"/ OR "clinical study"/ OR "case control study"/ OR "family study"/ OR "longitudinal study"/ OR "retrospective study"/ OR "prospective study"/ OR "cohort analysis"/ OR cohort*.ti,ab OR (("case control" ADJ1 (study OR studies)).ti,ab) OR (("follow up" ADJ1 (study OR studies)).ti,ab) OR (observational ADJ1 (study OR studies)).ti,ab OR ((epidemiologic ADJ1 (study OR studies)).ti,ab) OR (("cross sectional" ADJ1 (study OR studies)).ti,ab)) OR ("life cycle assessment"/ OR "environmental impact assessment"/ OR "life cycle assess*".mp OR "life cycle assessment".mp OR "life cycle inventory".mp OR "LCA".mp OR "LCAs".mp OR "life cycle inventory".mp OR "life cycle inventories".mp))

**Web of Science**

TS=(("Operating Rooms" OR "operating room" OR "operating rooms" OR "operation room" OR "operation rooms" OR "operating theatre" OR "operating theatres" OR "operation theatre" OR "operation theatres" OR "operating theater" OR "operating theaters" OR "operation theater" OR "operation theaters" OR "surgery room" OR "surgery rooms" OR "operation room" OR "operation rooms" OR "surgery theatre" OR "surgery theatres" OR "operation theatre" OR "operation theatres" OR "surgery theater" OR "surgery theaters" OR "operation theater" OR "operation theaters" OR "surgical room" OR "surgical rooms" OR "operation room" OR "operation rooms" OR "surgical theatre" OR "surgical theatres" OR "operation theatre" OR "operation theatres" OR "surgical theater" OR "surgical theaters" OR "operation theater" OR "operation theaters" OR "operating department" OR "operating departments" OR "operationg department" OR "operation departments" OR "surgical suite" OR "surgical suites" OR "surgery suite" OR "surgery suites" OR "operation suite" OR "operation suites" OR "operating suite" OR "operating suites") AND ("Ventilation" OR "ventilation" OR "ventilat*" OR "ventilation system" OR "ventilation systems" OR "air system" OR "air systems" OR "Air Conditioning" OR "Air Conditioning" OR "Air Condition*" OR "HVAC" OR "Heating Ventilation and Air-Conditioning" OR "Heating, Ventilation and Air-Conditioning" OR "hepa-filter" OR "hepafilter" OR "hepa filters" OR "hepafilters" OR "hepa filtration" OR "hepafiltration" OR "high efficiency particulate air" OR "unidirectional downflow system" OR "unidirectional downflow" OR "unidirectional down flow" OR "uni directional down flow" OR "laminair downflow" OR "laminair down flow" OR "laminar downflow" OR "laminar down flow" OR "plenum" OR "opragon" OR "halton" OR "3 zones" OR "three zones" OR "3 zone" OR "three zone" OR "Temperature 18-23 degrees Celsius" OR (("Temperature" OR "temperature" OR "temperatures") AND ("18" OR "19" OR "20" OR "21" OR "22" OR "23") AND ("celsius" OR "degree c" OR "degrees c")) OR "Humidity" OR "humidity" OR "humid" OR "recovery time" OR "recovery times" OR "NEN 14644-3" OR "ISO 14644-3" OR "14644-3" OR "air change" OR "air changes" OR "air quality ISO 7" OR "air quality" OR "ISO 7" OR "NEN 14644-1" OR "ISO 14644-1" OR "14644-1" OR "146441" OR "2 zones" OR "2 zone" OR "two zones" OR "two zone" OR "mixing ventilation airflow" OR "MAF" OR "Mixing ventilation air flow" OR "mixing ventilation" OR (("ventilation" OR "ventilat*" OR "airflow" OR "air flow") AND ("mixed" OR "mixing" OR "turbulent")) OR "conventional airflow" OR "conventional air flow" OR "ceiling diffusers" OR "ceiling diffuser" OR "ceiling diffus*" OR "circulatory system" OR "circulatory systems" OR "laminar flow" OR "unidirectional flow" OR "laminar-air-flow" OR "laminar airflow" OR "laminar flow*" OR "unidirectional flow*" OR "laminar-air-flow*" OR "laminar airflow*" OR "laminar air" OR "conventional ventilation" OR "ultraclean ventilation" OR "ultraclean air" OR "clean-airflow" OR "conventional ventilat*" OR "ultraclean ventilation" OR "ultraclean air" OR "ultra clean ventilation" OR "ultra clean air" OR "ultraclean ventilat*" OR "ultraclean air*" OR "ultra clean ventilat*" OR "ultra clean air*" OR "clean-airflow" OR "clean-airflow*" OR "opragon" OR "halton" OR "partial unidirectional airflow" OR "partial uni directional airflow" OR "partial unidirectional flow" OR "partial uni directional flow" OR "P-UDAF" OR "PUDAF" OR "unidirectional airflow" OR "uni directional airflow" OR "unidirectional airflow*" OR "uni directional airflow*" OR "unidirectional air*" OR "uni directional air*" OR "unidirectional flow*" OR "uni directional flow*" OR "displacement ventilation" OR "plenum ventilated" OR "plenum-ventilated" OR "plenum ventilation" OR "horizontal ventilation" OR "vertical ventilation" OR "plenum" OR "halton")) AND (TS=("Carbon Footprint" OR "carbon footprint" OR "carbon footprint*" OR "Climate Change" OR "climate change" OR "CO2 emission" OR "CO2 emissions" OR "CO2 footprint" OR "CO2 footprint*" OR "environmental protection" OR "conservation of natural resources" OR "environmental protection" OR "Disposable Equipment" OR "Disposables" OR "Disposable" OR "ecological footprint" OR "ecological footprint*" OR "ecological sustainability" OR "environmental impact" OR "environmental impact" OR "environmental impact*" OR "environmental impacts" OR "environmental pollut*" OR "pollution" OR "environmental pollution" OR "environmental sustainab*" OR "environmental sustainability" OR "environmental sustainability" OR "Global Warming" OR "Global Warming" OR "Greenhouse Effect" OR "greenhouse effect*" OR "greenhouse effects" OR "greenhouse gas emission" OR "greenhouse gas emissions" OR "Greenhouse Gas" OR "greening" OR "hospital waste" OR "life cycle assessment" OR "environmental impact assessment" OR "life cycle assess*" OR "life cycle assessment" OR "LCA" OR "LCAs" OR "life cycle inventory" OR "life cycle inventories" OR "Waste Disposal" OR "Hospital Waste" OR "medical waste" OR "Rising Sea Level" OR "Rising Sea Levels" OR "Sea Level Rise" OR "Sea Level Rise" OR "Waste Disposal" OR "waste water recycling" OR "Recycling" OR "recycling" OR "recycle*" OR "Equipment reuse" OR "Reusables" OR "reusable" OR "reuse" OR "reused" OR "reusing" OR "Waste Disposal" OR "Waste Management" OR "Plastic overuse" OR "Green surgery" OR "Emission reduction" OR "Emission reduction strategy" OR "air pollution control" OR "air pollution control" OR "acidification" OR "soil acidification" OR "ocean acidification" OR "acidification" OR "Acidification potential" OR "AP in kg SO2 equivalents" OR "eco-efficiency" OR "ecoefficiency" OR "eco-efficien*" OR "ecoefficien*" OR "ecotoxicity" OR "ecotoxicity" OR "ecotoxic*" OR "eco toxicity" OR "eco toxic*" OR "EP in kg PO4 equivalent" OR "Eutrophication" OR "eutrophication" OR "Eutrophication potential" OR "FAETP in kg DCB equivalent" OR "Freshwater Aquatic Ecotoxicity Potential" OR "GWP in kg CO2 equivalents" OR "H+ moles equivalents" OR "HTTP in kg Dichlorobenzene equivalent" OR "Human Toxicity Potential" OR "kg 2.4-D equivalents" OR "kg CFC-11 equivalent" OR "kg N equivalents" OR "kg NOx equivalents" OR "life cycle analysis" OR "ozone depletion" OR "Photochemical Ozone Depletion Potential" OR "POCP in kg ethane equivalent" OR "smog" OR "Waste" OR "waste" OR "wastes" OR "Ozone Depletion" OR "Smog" OR "Equipment reuse" OR "Greenhouse Gases" OR "Greenhouse Gas" OR "SO2 equiv*" OR "CO2 equiva*" OR "CFC-11 equiv*" OR "N equiv*" OR "Sustainable Development" OR "Sustainable Development" OR "Biodiversity" OR "Climatic change" OR "Green deal" OR "preservation of natural resources" OR "Refuse Disposal" OR "Waste Water" OR "Wastewater" OR "Water Purification" OR (("plastic*" OR "microplastic*") AND ("soop" OR "soup" OR "pollution" OR "overuse" OR "contamination")) OR (("Plastic" OR "plastics") AND "overuse") OR ("hydrogen*" AND "moles" AND "equiv*") OR ("Dichlorobenzen*" AND "equiv*") OR ("2,4-D" AND "equiv*") OR ("NOx" AND "equiv*") OR ("ethane" AND "equiv*") OR ("PO4" AND "equiv*") OR ("DCB" AND "equiv*") OR ("sustainability" AND ("environment*" OR "carbon")) OR (("Carbon Dioxide" OR "Carbon Dioxide" OR "CO2") AND ("pollution" OR "emission" OR "emissions" OR "waste" OR "environment" OR "environmental*" OR "footprint" OR "footprint*" OR "sustainable" OR "hazard" OR "hazard*"))) OR TI=("environmental*" OR "sustainab*")) NOT DT=(meeting abstract) AND (PY=(1980 OR 1981 OR 1982 OR 1983 OR 1984 OR 1985 OR 1986 OR 1987 OR 1988 OR 1989 OR 1990 OR 1991 OR 1992 OR 1993 OR 1994 OR 1995 OR 1996 OR 1997 OR 1998 OR 1999 OR 2000 OR 2001 OR 2002 OR 2003 OR 2004 OR 2005 OR 2006 OR 2007 OR 2008 OR 2009 OR 2010 OR 2011 OR 2012 OR 2013 OR 2014 OR 2015 OR 2016 OR 2017 OR 2018 OR 2019 OR 2020 OR 2021 OR 2022)) AND (TI=(*("Meta-Analysis" OR metaanaly* OR "meta-analy*" or metanaly* OR "Systematic Review" OR "Cochrane Database Syst Rev" OR "prisma" OR "preferred reporting items" OR "prospero" OR ((systemati* OR scoping OR umbrella OR "structured literature") NEAR/4 (review* OR overview*)) OR "systematic review*" OR "scoping review*" OR "umbrella review*" OR "structured literature review*" OR "systematic qualitative review*" OR "systematic quantitative review*" OR "systematic search and review" OR "systematized review" OR "systematised review" OR "systemic review" OR "systematic literature review*" OR "systematic integrative literature review*" OR "systematically review*" OR "scoping literature review*" OR "systematic critical review" OR "systematic integrative review*" OR "systematic evidence review" OR "Systematic integrative literature review*" OR "Systematic mixed studies review*" OR "Systematized literature review*" OR "Systematic overview*" OR "Systematic narrative review*" OR (("systemati*" OR "literature" OR "database*" OR "data-base*" OR "structured" OR "comprehensive*" OR "systemic*") NEAR/4 "search*") OR ("Literature" AND "review" AND ("database*" OR "data-base*" OR "search*")) OR (("data extraction" OR "data source*") AND "study selection") OR ("search strategy" AND "selection criteria") OR ("data source*" AND "data synthesis") OR "medline" OR "pubmed" OR "embase" OR "Cochrane" OR (("critical" OR "rapid") NEAR/4 ("review*" OR "overview*" OR "synthes*")) OR ((("critical*" OR "rapid*") NEAR/4 ("review*" OR "overview*" OR "synthes*") NEAR/4 ("search*" OR "database*" OR "data-base*"))) OR metasynthes* OR "meta-synthes*")* OR ("Randomized Controlled Trial" OR random* OR "RCT"OR "RCTs" OR "pragmatic clinical trial*" OR "practical clinical trial*" OR "non-inferiority trial*" OR "noninferiority trial*" OR "superiority trial*" OR "equivalence clinical trial*") OR ("Comparative Study" OR "comparison" OR "comparative" OR "compar*" OR "major clinical study" OR "clinical study" OR "case control study" OR "family study" OR "longitudinal study" OR "retrospective study" OR "prospective study" OR "cohort analysis" OR cohort* OR (("case control" NEAR/1 (study OR studies))) OR (("follow up" NEAR/1 (study OR studies))) OR (observational NEAR/1 (study OR studies)) OR ((epidemiologic NEAR/1 (study OR studies))) OR (("cross sectional" NEAR/1 (study OR studies)))) OR ("life cycle assess*" OR "life cycle assessment" OR "life cycle inventory" OR "LCA" OR "LCAs" OR "life cycle inventory" OR "life cycle inventories")) OR AB=(*("Meta-Analysis" OR metaanaly* OR "meta-analy*" or metanaly* OR "Systematic Review" OR "Cochrane Database Syst Rev" OR "prisma" OR "preferred reporting items" OR "prospero" OR ((systemati* OR scoping OR umbrella OR "structured literature") NEAR/4 (review* OR overview*)) OR "systematic review*" OR "scoping review*" OR "umbrella review*" OR "structured literature review*" OR "systematic qualitative review*" OR "systematic quantitative review*" OR "systematic search and review" OR "systematized review" OR "systematised review" OR "systemic review" OR "systematic literature review*" OR "systematic integrative literature review*" OR "systematically review*" OR "scoping literature review*" OR "systematic critical review" OR "systematic integrative review*" OR "systematic evidence review" OR "Systematic integrative literature review*" OR "Systematic mixed studies review*" OR "Systematized literature review*" OR "Systematic overview*" OR "Systematic narrative review*" OR (("systemati*" OR "literature" OR "database*" OR "data-base*" OR "structured" OR "comprehensive*" OR "systemic*") NEAR/4 "search*") OR ("Literature" AND "review" AND ("database*" OR "data-base*" OR "search*")) OR (("data extraction" OR "data source*") AND "study selection") OR ("search strategy" AND "selection criteria") OR ("data source*" AND "data synthesis") OR "medline" OR "pubmed" OR "embase" OR "Cochrane" OR (("critical" OR "rapid") NEAR/4 ("review*" OR "overview*" OR "synthes*")) OR ((("critical*" OR "rapid*") NEAR/4 ("review*" OR "overview*" OR "synthes*") NEAR/4 ("search*" OR "database*" OR "data-base*"))) OR metasynthes* OR "meta-synthes*")* OR ("Randomized Controlled Trial" OR random* OR "RCT"OR "RCTs" OR "pragmatic clinical trial*" OR "practical clinical trial*" OR "non-inferiority trial*" OR "noninferiority trial*" OR "superiority trial*" OR "equivalence clinical trial*") OR ("Comparative Study" OR "comparison" OR "comparative" OR "compar*" OR "major clinical study" OR "clinical study" OR "case control study" OR "family study" OR "longitudinal study" OR "retrospective study" OR "prospective study" OR "cohort analysis" OR cohort* OR (("case control" NEAR/1 (study OR studies))) OR (("follow up" NEAR/1 (study OR studies))) OR (observational NEAR/1 (study OR studies)) OR ((epidemiologic NEAR/1 (study OR studies))) OR (("cross sectional" NEAR/1 (study OR studies)))) OR ("life cycle assess*" OR "life cycle assessment" OR "life cycle inventory" OR "LCA" OR "LCAs" OR "life cycle inventory" OR "life cycle inventories"))**)**

**Cochrane Library**

(("Operating Rooms" OR "operating room" OR "operating rooms" OR "operation room" OR "operation rooms" OR "operating theatre" OR "operating theatres" OR "operation theatre" OR "operation theatres" OR "operating theater" OR "operating theaters" OR "operation theater" OR "operation theaters" OR "surgery room" OR "surgery rooms" OR "operation room" OR "operation rooms" OR "surgery theatre" OR "surgery theatres" OR "operation theatre" OR "operation theatres" OR "surgery theater" OR "surgery theaters" OR "operation theater" OR "operation theaters" OR "surgical room" OR "surgical rooms" OR "operation room" OR "operation rooms" OR "surgical theatre" OR "surgical theatres" OR "operation theatre" OR "operation theatres" OR "surgical theater" OR "surgical theaters" OR "operation theater" OR "operation theaters" OR "operating department" OR "operating departments" OR "operationg department" OR "operation departments" OR "surgical suite" OR "surgical suites" OR "surgery suite" OR "surgery suites" OR "operation suite" OR "operation suites" OR "operating suite" OR "operating suites") AND ("Ventilation" OR "ventilation" OR "ventilation system" OR "ventilation systems" OR "air system" OR "air systems" OR "Air Conditioning" OR "Air Conditioning" OR "HVAC" OR "Heating Ventilation and Air Conditioning" OR "Heating, Ventilation and Air Conditioning" OR "hepa filter" OR "hepafilter" OR "hepa filters" OR "hepafilters" OR "hepa filtration" OR "hepafiltration" OR "high efficiency particulate air" OR "unidirectional downflow system" OR "unidirectional downflow" OR "unidirectional down flow" OR "uni directional down flow" OR "laminair downflow" OR "laminair down flow" OR "laminar downflow" OR "laminar down flow" OR "plenum" OR "opragon" OR "halton" OR "3 zones" OR "three zones" OR "3 zone" OR "three zone" OR "Temperature 18 23 degrees Celsius" OR (("Temperature" OR "temperature" OR "temperatures") NEAR/4 ("18" OR "19" OR "20" OR "21" OR "22" OR "23") AND ("celsius" OR "degree c" OR "degrees c")) OR "Humidity" OR "humidity" OR "humid" OR "recovery time" OR "recovery times" OR "NEN 14644 3" OR "ISO 14644 3" OR "14644 3" OR "air change" OR "air changes" OR "air quality ISO 7" OR "air quality" OR "ISO 7" OR "NEN 14644 1" OR "ISO 14644 1" OR "14644 1" OR "146441" OR "2 zones" OR "2 zone" OR "two zones" OR "two zone" OR "mixing ventilation airflow" OR "MAF" OR "Mixing ventilation air flow" OR "mixing ventilation" OR (("ventilation" OR "airflow" OR "air flow") NEAR/4 ("mixed" OR "mixing" OR "turbulent")) OR "conventional airflow" OR "conventional air flow" OR "ceiling diffusers" OR "ceiling diffuser" OR "circulatory system" OR "circulatory systems" OR "laminar flow" OR "unidirectional flow" OR "laminar air flow" OR "laminar airflow" OR "laminar air" OR "conventional ventilation" OR "ultraclean ventilation" OR "ultraclean air" OR "clean airflow" OR "ultraclean ventilation" OR "ultraclean air" OR "ultra clean ventilation" OR "ultra clean air" OR "clean airflow" OR "opragon" OR "halton" OR "partial unidirectional airflow" OR "partial uni directional airflow" OR "partial unidirectional flow" OR "partial uni directional flow" OR "P UDAF" OR "PUDAF" OR "unidirectional airflow" OR "uni directional airflow" OR "displacement ventilation" OR "plenum ventilated" OR "plenum ventilated" OR "plenum ventilation" OR "horizontal ventilation" OR "vertical ventilation" OR "plenum" OR "halton")):ti,ab,kw AND

(("Carbon Footprint" OR "carbon footprint" OR "carbon footprint*" OR "Climate Change" OR "climate change" OR "CO2 emission" OR "CO2 emissions" OR "CO2 footprint" OR "CO2 footprint*" OR "environmental protection" OR "conservation of natural resources" OR "environmental protection" OR "Disposable Equipment" OR "Disposables" OR "Disposable" OR "ecological footprint" OR "ecological footprint*" OR "ecological sustainability" OR "environmental impact" OR "environmental impact" OR "environmental impact*" OR "environmental impacts" OR "environmental pollut*" OR "pollution" OR "environmental pollution" OR "environmental sustainab*" OR "environmental sustainability" OR "environmental sustainability" OR "Global Warming" OR "Global Warming" OR "Greenhouse Effect" OR "greenhouse effect*" OR "greenhouse effects" OR "greenhouse gas emission" OR "greenhouse gas emissions" OR "Greenhouse Gas" OR "greening" OR "hospital waste" OR "life cycle assessment" OR "environmental impact assessment" OR "life cycle assessment" OR "environmental impact assessment" OR "life cycle assess*" OR "life cycle assessment" OR "LCA" OR "LCAs" OR "life cycle inventory" OR "life cycle inventories" OR "Waste Disposal" OR "Hospital Waste" OR "medical waste" OR "Rising Sea Level" OR "Rising Sea Levels" OR "Sea Level Rise" OR "Sea Level Rise" OR "Waste Disposal" OR "waste water recycling" OR "Recycling" OR "recycling" OR "recycle*" OR "Equipment reuse" OR "Reusables" OR "reusable" OR "reuse" OR "reused" OR "reusing" OR "Waste Disposal" OR "Waste Management" OR "Plastic overuse" OR "Green surgery" OR "Emission reduction" OR "Emission reduction strategy" OR "air pollution control" OR "air pollution control" OR "acidification" OR "soil acidification" OR "ocean acidification" OR "acidification" OR "Acidification potential" OR "AP in kg SO2 equivalents" OR "eco-efficiency" OR "ecoefficiency" OR "eco-efficien*" OR "ecoefficien*" OR "ecotoxicity" OR "ecotoxicity" OR "ecotoxic*" OR "eco toxicity" OR "eco toxic*" OR "EP in kg PO4 equivalent" OR "Eutrophication" OR "eutrophication" OR "Eutrophication potential" OR "FAETP in kg DCB equivalent" OR "Freshwater Aquatic Ecotoxicity Potential" OR "GWP in kg CO2 equivalents" OR "H+ moles equivalents" OR "HTTP in kg Dichlorobenzene equivalent" OR "Human Toxicity Potential" OR "kg 2.4-D equivalents" OR "kg CFC-11 equivalent" OR "kg N equivalents" OR "kg NOx equivalents" OR "life cycle analysis" OR "ozone depletion" OR "Photochemical Ozone Depletion Potential" OR "POCP in kg ethane equivalent" OR "smog" OR "Waste" OR "waste" OR "wastes" OR "Ozone Depletion" OR "Smog" OR "Equipment reuse" OR "Greenhouse Gases" OR "Greenhouse Gas" OR "SO2 equiv*" OR "CO2 equiva*" OR "CFC-11 equiv*" OR "N equiv*" OR "Biodiversity" OR "Climatic change" OR "Green deal" OR "preservation of natural resources" OR "Refuse Disposal" OR "Waste Water" OR "Wastewater" OR "Water Purification" OR (("plastic*" OR "microplastic*") AND ("soop" OR "soup" OR "pollution" OR "overuse" OR "contamination")) OR "Sustainable Development" OR "Sustainable Development" OR (("Plastic" OR "plastics") AND "overuse") OR ("hydrogen*" AND "moles" AND "equiv*") OR ("Dichlorobenzen*" AND "equiv*") OR ("2,4-D" AND "equiv*") OR ("NOx" AND "equiv*") OR ("ethane" AND "equiv*") OR ("PO4" AND "equiv*") OR ("DCB" AND "equiv*") OR ("sustainability" AND ("environment*" OR "carbon")) OR (("Carbon Dioxide" OR "Carbon Dioxide" OR "CO2") AND ("pollution" OR "emission" OR "emissions" OR "waste" OR "environment" OR "environmental*" OR "footprint" OR "footprint*" OR "sustainable" OR "hazard" OR "hazard*"))):ti,ab,kw OR ("environmental" OR "sustainability"):ti)

NOT DT=(meeting abstract)

**Emcare**

(exp "Operating Room"/ OR "operating room".mp OR "operating rooms".mp OR "operation room".mp OR "operation rooms".mp OR "operating theatre".mp OR "operating theatres".mp OR "operation theatre".mp OR "operation theatres".mp OR "operating theater".mp OR "operating theaters".mp OR "operation theater".mp OR "operation theaters".mp OR "surgery room".mp OR "surgery rooms".mp OR "operation room".mp OR "operation rooms".mp OR "surgery theatre".mp OR "surgery theatres".mp OR "operation theatre".mp OR "operation theatres".mp OR "surgery theater".mp OR "surgery theaters".mp OR "operation theater".mp OR "operation theaters".mp OR "surgical room".mp OR "surgical rooms".mp OR "operation room".mp OR "operation rooms".mp OR "surgical theatre".mp OR "surgical theatres".mp OR "operation theatre".mp OR "operation theatres".mp OR "surgical theater".mp OR "surgical theaters".mp OR "operation theater".mp OR "operation theaters".mp OR "operating department".mp OR "operating departments".mp OR ("operation".ti AND ("department".ti OR "departments".ti)) OR "surgical suite".mp OR "surgical suites".mp OR "surgery suite".mp OR "surgery suites".mp OR "operation suite".mp OR "operation suites".mp OR "operating suite".mp OR "operating suites".mp) AND ("room ventilation"/ OR "air conditioning"/ OR exp "ventilator"/ OR "ventilation".mp OR "ventilat*".mp OR "ventilation system".mp OR "ventilation systems".mp OR "air system".mp OR "air systems".mp OR "Air Conditioning".mp OR "Air Condition*".mp OR "HVAC".mp OR "Heating Ventilation and Air-Conditioning".mp OR "Heating, Ventilation and Air-Conditioning".mp OR "hepa-filter".mp OR "hepafilter".mp OR "hepa filters".mp OR "hepafilters".mp OR "hepa filtration".mp OR "hepafiltration".mp OR "high efficiency particulate air".mp OR exp "air filter"/ OR "Air Filters".mp OR "Air Filter".mp OR "Airfilters".mp OR "Airfilter".mp OR "Air Filt*".mp OR "Airfilt*".mp OR "unidirectional downflow system".mp OR "unidirectional downflow".mp OR "unidirectional down flow".mp OR "uni directional down flow".mp OR "laminair downflow".mp OR "laminair down flow".mp OR "laminar downflow".mp OR "laminar down flow".mp OR "plenum".mp OR "opragon".mp OR "halton".mp OR "3 zones".mp OR "three zones".mp OR "3 zone".mp OR "three zone".mp OR "Temperature 18-23 degrees Celsius".mp OR ((exp "Temperature"/ OR "temperature".mp OR "temperatures".mp) AND ("18".mp OR "19".mp OR "20".mp OR "21".mp OR "22".mp OR "23".mp) AND ("celsius".mp OR "degree c".mp OR "degrees c".mp)) OR exp "Humidity"/ OR "humidity".mp OR "humid".mp OR "recovery time".mp OR "recovery times".mp OR "NEN 14644-3".mp OR "ISO 14644-3".mp OR "14644-3".mp OR "air change".mp OR "air changes".mp OR "air quality ISO 7".mp OR "air quality".mp OR "ISO 7".mp OR "NEN 14644-1".mp OR "ISO 14644-1".mp OR "14644-1".mp OR "146441".mp OR "2 zones".mp OR "2 zone".mp OR "two zones".mp OR "two zone".mp OR "mixing ventilation airflow".mp OR "MAF".mp OR "Mixing ventilation air flow".mp OR "mixing ventilation".mp OR (("ventilation".mp OR "ventilat*".mp OR "airflow".mp OR "air flow".mp) AND ("mixed".mp OR "mixing".mp OR "turbulent".mp)) OR "airflow"/ OR "conventional airflow".mp OR "conventional air flow".mp OR "ceiling diffusers".mp OR "ceiling diffuser".mp OR "ceiling diffus*".mp OR "circulatory system".mp OR "circulatory systems".mp OR "laminar airflow"/ OR "laminar flow".mp OR "unidirectional flow".mp OR "laminar-air-flow".mp OR "laminar airflow".mp OR "laminar flow*".mp OR "laminar-air-flow*".mp OR "laminar airflow*".mp OR "laminar air".mp OR "conventional ventilation".mp OR "ultraclean ventilation".mp OR "ultraclean air".mp OR "conventional ventilat*".mp OR "ultraclean air".mp OR "ultra clean ventilation".mp OR "ultra clean air".mp OR "ultraclean ventilat*".mp OR "ultraclean air*".mp OR "ultra clean ventilat*".mp OR "ultra clean air*".mp OR "clean-airflow".mp OR "clean-airflow*".mp OR "partial unidirectional airflow".mp OR "partial uni directional airflow".mp OR "partial unidirectional flow".mp OR "partial uni directional flow".mp OR "P-UDAF".mp OR "PUDAF".mp OR "unidirectional airflow".mp OR "uni directional airflow".mp OR "unidirectional airflow*".mp OR "uni directional airflow*".mp OR "unidirectional air*".mp OR "uni directional air*".mp OR "unidirectional flow*".mp OR "uni directional flow*".mp OR "displacement ventilation".mp OR "plenum ventilated".mp OR "plenum-ventilated".mp OR "plenum ventilation".mp OR "horizontal ventilation".mp OR "vertical ventilation".mp) AND ("Carbon Footprint"/ OR "carbon footprint".mp OR "carbon footprint*".mp OR exp "Climate Change"/ OR "climate change".mp OR "CO2 emission".mp OR "CO2 emissions".mp OR "CO2 footprint".mp OR "CO2 footprint*".mp OR exp "environmental protection"/ OR "conservation of natural resources".mp OR "environmental protection".mp OR "Disposable Equipment"/ OR "Disposables".mp OR "Disposable".mp OR "ecological footprint".mp OR "ecological footprint*".mp OR "ecological sustainability".mp OR exp "environmental impact"/ OR "environmental impact".mp OR "environmental impact*".mp OR "environmental impacts".mp OR "environmental pollut*".mp OR exp "pollution"/ OR "environmental pollution".mp OR "environmental sustainab*".mp OR "environmental sustainability"/ OR "environmental sustainability".mp OR "Global Warming"/ OR "Global Warming".mp OR "Greenhouse Effect"/ OR "greenhouse effect*".mp OR "greenhouse effects".mp OR "greenhouse gas emission".mp OR "greenhouse gas emissions".mp OR "Greenhouse Gas"/ OR "greening".mp OR "hospital waste".mp OR "life cycle assessment"/ OR "environmental impact assessment"/ OR "life cycle assess*".mp OR "life cycle assessment".mp OR "LCA".mp OR "LCAs".mp OR "life cycle inventory".mp OR "life cycle inventories".mp OR exp "Waste Disposal"/ OR exp "Hospital Waste"/ OR "medical waste".mp OR "Rising Sea Level".mp OR "Rising Sea Levels".mp OR "Sea Level Rise"/ OR "Sea Level Rise".mp OR "sustainability".ti OR "Waste Disposal".mp OR "waste water recycling"/ OR "Recycling"/ OR "recycling".mp OR "recycle*".mp OR "Equipment reuse".mp OR "Reusables".mp OR "reusable".mp OR "reuse".mp OR "reused".mp OR "reusing".mp OR exp "Waste Disposal"/ OR exp "Waste Management"/ OR "Plastic overuse".mp OR "Green surgery".mp OR "Emission reduction".mp OR "Emission reduction strategy".mp OR "air pollution control"/ OR "air pollution control".mp OR "Environment"/ OR "Environmental*".ti OR "acidification"/ OR "soil acidification"/ OR "ocean acidification"/ OR "acidification".mp OR "Acidification potential".mp OR "AP in kg SO2 equivalents".mp OR "eco-efficiency".mp OR "ecoefficiency".mp OR "eco-efficien*".mp OR "ecoefficien*".mp OR "ecotoxicity"/ OR "ecotoxicity".mp OR "ecotoxic*".mp OR "eco toxicity".mp OR "eco toxic*".mp OR "EP in kg PO4 equivalent".mp OR exp "Eutrophication"/ OR "eutrophication".mp OR "Eutrophication potential".mp OR "FAETP in kg DCB equivalent".mp OR "Freshwater Aquatic Ecotoxicity Potential".mp OR "GWP in kg CO2 equivalents".mp OR "H+ moles equivalents".mp OR "HTTP in kg Dichlorobenzene equivalent".mp OR "Human Toxicity Potential".mp OR "kg 2.4-D equivalents".mp OR "kg CFC-11 equivalent".mp OR "kg N equivalents".mp OR "kg NOx equivalents".mp OR "life cycle analysis".mp OR "ozone depletion".mp OR "Photochemical Ozone Depletion Potential".mp OR "POCP in kg ethane equivalent".mp OR "smog".mp OR exp "Waste"/ OR "waste".mp OR "wastes".mp OR "Ozone Depletion"/ OR "Smog"/ OR "Equipment reuse".mp OR "Greenhouse Gases".mp OR "Greenhouse Gas".mp OR "SO2 equiv*".mp OR "CO2 equiva*".mp OR "CFC-11 equiv*".mp OR "N equiv*".mp OR exp "Biodiversity"/ OR "Biodiversity".mp OR "Climatic change".mp OR "Green deal".mp OR "preservation of natural resources".mp OR "Refuse Disposal".mp OR exp "Wastewater"/ OR "Waste Water".mp OR "Wastewater".mp OR exp "Water Management"/ OR "Water Purification".mp OR (("plastic*".mp OR "microplastic*".mp) AND ("soop".mp OR "soup".mp OR "pollution".mp OR "overuse".mp OR "contamination".mp)) OR "Sustainable Development"/ OR "Sustainable Development".mp OR (("Plastic".mp OR "plastics".mp) AND "overuse".mp) OR ("hydrogen*".mp AND "moles".mp AND "equiv*".mp) OR ("Dichlorobenzen*".mp AND "equiv*".mp) OR ("2,4-D".mp AND "equiv*".mp) OR ("NOx".mp AND "equiv*".mp) OR ("ethane".mp AND "equiv*".mp) OR ("PO4".mp AND "equiv*".mp) OR ("DCB".mp AND "equiv*".mp) OR ("sustainability".mp AND ("environment*".mp OR "carbon".mp)) OR (("Carbon Dioxide"/ OR "Carbon Dioxide".mp OR "CO2".mp) AND ("pollution".mp OR "emission".mp OR "emissions".mp OR "waste".mp OR "environment".mp OR "environmental*".mp OR "footprint".mp OR "footprint*".mp OR "sustainable".mp OR "hazard".mp OR "hazard*".mp))) NOT (conference review or conference abstract).pt AND 1980:2023.(sa_year) AND (*(exp "meta analysis"/ OR exp "meta analysis (topic)"/ OR metaanaly*.ti,ab OR "meta analy*".ti,ab OR metanaly*.ti,ab OR "systematic review"/ OR "cochrane database of systematic reviews".jn OR prisma.ti,ab OR prospero.ti,ab OR (((systemati* OR scoping OR umbrella OR "structured literature") ADJ3 (review* OR overview*)).ti,ab) OR ((systemic* ADJ1 review*).ti,ab) OR (((systemati* OR literature OR database* OR "data base*") ADJ10 search*).ti,ab) OR (((structured OR comprehensive* OR systemic*) ADJ3 search*).ti,ab) OR (((literature ADJ3 review*).ti,ab) AND (search*.ti,ab OR database*.ti,ab OR "data base*".ti,ab)) OR (("data extraction".ti,ab OR "data source*".ti,ab) AND "study selection".ti,ab) OR ("search strategy".ti,ab AND "selection criteria".ti,ab) OR ("data source*".ti,ab AND "data synthesis".ti,ab) OR medline.ab OR pubmed.ab OR embase.ab OR cochrane.ab OR (((critical OR rapid) ADJ2 (review* OR overview* OR synthes*)).ti) OR ((((critical* OR rapid*) ADJ3 (review* OR overview* OR synthes*)).ab) AND (search*.ab OR database*.ab OR "data base*".ab)) OR metasynthes*.ti,ab OR "meta synthes*".ti,ab)* OR (exp "clinical trial"/ OR exp "randomization"/ OR exp "single blind procedure"/ OR exp "double blind procedure"/ OR exp "crossover procedure"/ OR exp "placebo"/ OR exp "prospective study"/ OR rct.ti,ab OR random*.ti,ab OR "single blind".ti,ab OR "randomised controlled trial".ti,ab OR exp "randomized controlled trial"/ OR placebo*.ti,ab) OR (exp "Comparative Study"/ OR "comparison".ti,ab OR "comparative".ti,ab OR "compar*".ti,ab OR "major clinical study"/ OR "clinical study"/ OR "case control study"/ OR "family study"/ OR "longitudinal study"/ OR "retrospective study"/ OR "prospective study"/ OR "cohort analysis"/ OR cohort*.ti,ab OR (("case control" ADJ1 (study OR studies)).ti,ab) OR (("follow up" ADJ1 (study OR studies)).ti,ab) OR (observational ADJ1 (study OR studies)).ti,ab OR ((epidemiologic ADJ1 (study OR studies)).ti,ab) OR (("cross sectional" ADJ1 (study OR studies)).ti,ab)) OR ("life cycle assessment"/ OR "environmental impact assessment"/ OR "life cycle assess*".mp OR "life cycle assessment".mp OR "life cycle inventory".mp OR "LCA".mp OR "LCAs".mp OR "life cycle inventory".mp OR "life cycle inventories".mp))

## Appendix 4. Evidence tables

Evidence table included comparative studies (based on Drew, 2021)

### Table 1a: Study characteristics LCA studies

| Study reference |  |  | Study characteristics |  |  |  |  |  |  |  |
| --- | --- | --- | --- | --- | --- | --- | --- | --- | --- | --- |
| Author and year | Title | Journal | Type of study | Objective | LCA-method | Setting and country | Facility | Years of data collection | Surgical discipline(s) | Funding and conflict of interest |
| ***Topic 1 "Surgical techniques"*** |  |  |  |  |  |  |  |  |  |  |
| Papadopoulou et al. (2022) *(see S2)* |  |  |  |  |  |  |  |  |  |  |
| Power et al. (2012) | Environmental impact of minimally invasive surgery in the United States | Journal of Endourology | CFA without LCA-methods | To assess the additional climate impact of minimally invasive surgery (MIS) as compared with traditional open surgery | LCA-method undetermined (while it assesses CO₂ emissions, the study does not cover the full life cycle of the surgical process comprehensively, nor does it explicitly follow the defined LCA framework (functional unit, multiple impact categories, inventory database, etc.) | Inpatient and outpatient clinics in the US | Memorial Sloan-Kettering Cancer Center, New York City, NY, USA | 2009 | Gastroenterology, Obstetrics & Gynecology, Urology & Nephrology | Supported by The Sidney Kimmel Center for Prostate and Urologic Cancers and by Award Number U54CA137788/U54CA132378 from the National Cancer Institute.  No competing financial interests exist. |
| Thiel et al. (2015) | Environmental impacts of surgical procedures: Life cycle assessment of hysterectomy in the United States | Environmental Science & Technology | LCA | To assess the environmental impacts of four different surgical approaches to hysterectomy: vaginal, abdominal, laparoscopic, and robotic | Hybrid LCA (ISO 14040-44) | Hospital in the USA | Magee-Womens Hospital of the University of Pittsburgh Medical Center, Pittsburgh, PA, USA | 2011 | Obstetrics & Gynaecology | Financial support for the data collection came from Grant Number ULI RR024153 from the National Center for Research Resources (NCRR), a component of the National Institutes of Health (NIH), and NIH Roadmap for Medical Research. Support for graduate researchers came from Award No. 050434 from the National Science Foundation (NSF) Integrative Education and Research Traineeship (IGERT).   The authors declare no competing financial interest. |
| Woods et al. (2015) | Carbon footprint of robotically-assisted laparoscopy, laparoscopy and laparotomy: a comparison | International Journal of Medical Robotics and Computer Assisted Surgery | CFA without LCA-methods | To assess the climate impact three surgical modalities for endometrial cancer staging: laparotomy, laparoscopy, robotic-assisted laparoscopy | LCA-method undetermined | Hospital in the USA | Albert Einstein College of Medicine, New York City, NY, USA | 2008-2011 | Obstetrics & Gynaecology; Oncology | The authors declare no potential conflicts of interest. |
| ***Topic 2 "Disposables versus reusables"*** |  |  |  |  |  |  |  |  |  |  |
| Almutairi et al. (2022) | The planetary health effects of COVID-19 in dental care: a life cycle assessment approach | British Dental Journal | LCA | To assess and compare the environmental impact of different forms of PPE before and after COVID-19. | Attributional LCA (ISO 14040 - 14044) | Dental University Hospital, UK | Dublin Dental University Hospital (DDUH) | 2020-2021 | Dental care | The authors declare no conlicts of interest. |
| Boberg et al. (2022) | Environmental impact of single-use, reusable, and mixed trocar systems used for laparoscopic cholecystectomies | PLoS one | LCA | To assess the environmental impact of single-use, reusable, and mixed trocar systems used for laparoscopic cholecystectomies at three hospitals in southern Sweden. | Attributional LCA (ISO 14044) and conventional LCC | Three hospitals in Sweden | Landskrona Hospital; Helsingborg Hospital; Skane Unversity Hospital, Lund | - | Gastroenterology | The authors declare no competing interests. Funding was received from Lund University Agenda 2030 Graduate School and Swedish Government funding (grant number: 86626). The funders had no role in the study design, data collection and analysis, publishing and manuscript preparation. |
| Boucheron et al. (2022) | Cost and Environmental Impact of Disposable Flexible Cystoscopes Compared to Reusable Devices | Journal of Endourology | Other (Single-center retrospective study - waste and water audit) | To assess the environmental impact (waste and water use) and costs of cystoscopies with disposable and reusable cystoscopes. | - | Hospital in France | Assistance Publique-Hôpitaux de Marseille | 2020-2021 | Urology | The authors declare no competing and financial interests. |
| Davis et al. (2018) | Carbon Footprint in Flexible Ureteroscopy: A Comparative Study on the Environmental Impact of Reusable and Single-Use Ureteroscopes | Journal of Endourology | CFA without LCA-methods | To assess the climate change impacts of two types of flexible ureteroscopes: single-use (LithoVue™, Boston Scientific) and reusable (Olympus Flexible Video; typically 16 uses before repair and 180 uses before decommissioning) | LCA-method undetermined | Hospital in Australia | Austin Hospital, Melbourne, Victoria, Australia | - | Urology & Nephrology | - |
| Donahue et al. (2020) | A comparative carbon footprint analysis of disposable and reusable vaginal specula | American Journal of Obstetrics & Gynecology | CFA using LCA-methods | To assess the climate change impacts of three types of vaginal specula that are commonly used in practice (a single-use acrylic model and two reusable stainless steel models) | Attributional LCA (ISO 14040) | Hospital in the USA | Michigan Medicine, University of Michigan, Ann Arbor, MI, USA | - | Obstetrics & Gynaecology; Oncology | The authors report no conflict of interest. |
| Drew et al. (2021) *(see S2)* |  |  |  |  |  |  |  |  |  |  |
| Eckelman et al. (2012) | Comparative life cycle assessment of disposable and reusable laryngeal mask airways | Anesthesia & Analgesia | LCA | To assess the environmental impacts of two types of laryngeal mask airways (LMAs): single-use (Unique™) and reusable (Classic™; 40 lifetime uses) | Attributional LCA (ISO 14040) | Hospital in the USA | Yale-New Haven Hospital, New Haven, CT, USA | - | Anaesthesiology | The authors declare no conflict of interest. Funding came from the department of anesthesiology, Yale School of Medicine. |
| Friedericy et al. (2022) | Reducing the Environmental Impact of Sterilization Packaging for Surgical Instruments in the Operating Room: A Comparative Life Cycle Assessment of Disposable versus Reusable Systems | Sustainability | LCA | To assess the environmental impact of the reusable RSC compared to blue wrap with closed loop recycling. | Attributional LCA (ISO 14040 - 14044) | Hospital in the Netherlands | Leiden University Medical Centre, Leiden, The Netherlands | - | Nonspecific | None stated. |
| Grimmond et al. (2012) | Impact on carbon footprint: a life cycle assessment of disposable versus reusable sharps containers in a large US hospital | Waste Management & Research | CFA using LCA-methods | To assess the climate impacts of two different sharps container systems (disposable and reusable) over a 12 month period. | Attributional LCA (PAS 2050) | Hospital in the USA | Northwestern Memorial Hospital (NMH, Chicago) | - | Nonspecific | The authors declare no competing and financial interests. |
| Grimmond et al. (2021) | Before/after intervention study to determine impact on life-cycle carbon footprint of converting from single-use to reusable sharps containers in 40 UK NHS trusts | BMJ open | CFA using LCA-methods | To assess climate impacts of hospitals converting from single- use sharps containers to reusable sharps containers (SSC, RSC). | Attributional LCA (PAS 2050) | Acute care hospital trusts in the UK | 40 UK NHS hospital trusts using RSC | 2018-2019 | Nonspecific | Competing interests: TRG is an international consultant on sharps injury prevention and sharps containment to healthcare facilities, professional associations and contractors in the healthcare sector, and from time to time, Sharpsmart UK (the manufacturer of the RSC studied in this paper) use his services. All other authors are employed by, or associated with, trusts using Sharpsmart reusable sharps containers and declare no conflicts of interest. Funding: Funding Sharpsmart UK granted £5000 to the project which covered approximately 20% of the hours required for completion and publication. The funder had no role in study initiation, design, data analysis, journal selection, or content of the manuscript. No other grant or funding was received from any funding agency in the public, commercial, or not-for-profit sectors. |
| Hicks et al. (2016) | Environmental impacts of reusable nanoscale silver-coated hospital gowns compared to single-use, disposable gowns | Environmental Science: Nano from The Royal Society of Chemistry | LCA | To assess environmental impact of reusable patient hospital gowns coated with nAg (nanosilver) product to the use of disposable gowns. | Attributional LCA (unspecified) | USA | Hospital case study | - | Nonspecific | U.S. Environmental Protection Agency Assistance Agreement No. RD 83558001-0 funded this research. |
| Hogan et al. (2022) | Carbon footprint of single-use flexible cystoscopes compared with reusable cystoscopes | Journal of Endourology | CFA without LCA-methods | To assess the carbon footprint of single-use versus reusable flexible cystoscopes based on waste production and estimated carbon emissions. | LCA-method undetermined | Hospital in Cork, Ireland | Mercy University Hospital, Cork, Ireland | 2021 | Urology | The authors declare no conflict of interest. |
| Ibbotson et al. (2013) | Eco-efficiency of disposable and reusable surgical instruments - a scissors case | International Journal of Life Cycle Assessment | LCA | To assess the environmental and financial impacts of three types of surgical scissors: disposable plastic reinforced scissors, disposable stainless steel scissors, and reusable stainless steel scissors | Attributional LCA (ISO 14040) | Hospital in Germany | - | - | Nonspecific | - |
| Keil et al. (2022) *(see S2)* |  |  |  |  |  |  |  |  |  |  |
| Kemble et al. (2022) | Environmental impact of single-use and reusable flexible cystoscopes | BJUI | CFA without LCA-methods | To assess the carbon footprint of single-use and reusable flexible cystoscopes | LCA-method undetermined | USA | - | - | Urology | The authors declare no conflict of interest and no funding source |
| Le et al. (2022) | Environmental and health outcomes of single-use versus reusable duodenoscopes | Gastrointestinal Endoscopy | LCA | To assess the environmental and human health impacts of reusable and single-use duodenoscopes. | Attributional LCA (unspecified) | USA | All Saints Hospital, Racine, Wisconsin, USA | 2020 | Gastro-enterology | The following authors disclosed financial relationships: L. Hernandez: Contributor to Up-To-Date; associate editor for Gastro intestinal Endoscopy; consultant for Liquiglide and Iterative Scopes. N. Vakil: Consultant for Phathom Pharmaceuticals, Cinclus Pharmaceuticals, Isothrive, and Ironwood Pharmaceuticals; speaker for Astra Zeneca; writer for Merck. N. Guda: Consultant for Boston Scientific and Hemostatis LLC; speaker for Lupin Pharmaceuticals India. C. Patnode: Grant recipient and meeting fee compensation from Healthcare Without Harm. All other authors disclosed no financial relationships. |
| Leiden et al. (2020) | Life cycle assessment of a disposable and a reusable surgery instrument set for spinal fusion surgeries | Resources, Conservation & Recycling | LCA | To assess the environmental impacts of two types of instrument set for single-level lumbar fusion surgeries: disposable (Neo Pedicle Screw System from Neo Medical SA) and reusable (Viper 2 from DePuy Synthes, 300 uses). | Attributional LCA (DIN EN ISO 14040) | Hospital in Germany | - | - | Neurology | The study was funded by Neo Medical S.A., but it is stated that Neo Medical S.A. had no direct influence on the results of the study. |
| McGain et al. (2010) | The financial and environmental costs of reusable and single-use plastic anaesthetic drug trays | Anaesthesia and Intensive Care | LCA | To assess the environmental and financial impacts of two types of commonly used plastic anesthetic drug trays: a single-use polyurethane tray made in China and reusable (300 uses) nylon tray made in Australia. Impacts and financial costs of two cotton gauzes and one paper towel, which are included with most single-use trays, were separately modelled. | Attributional LCA (ISO 14040) | Hospital in Australia | Western Health, Melbourne, Victoria, Australia | - | Anaesthesiology | The authors declare no conflict of interest. |
| McGain et al. (2012) | A life cycle assessment of reusable and single-use venous catheter insertion kits | Anesthesia & Analgesia | LCA | To assess the environmental and financial impacts of two types of central venous catheter insertion kits: single-use and reusable | Attributional LCA (ISO 14040) | Hospital in Australia | Western Health, Melbourne, Victoria, Australia | - | Anaesthesiology | The authors declare no conflict of interest. They received funding through grants from the Australian and New Zealand Intensive Care Society and Sustainability Victoria. |
| McGain et al. (2017) | Financial and environmental costs of reusable and single-use anaesthetic equipment | British Journal of Anaesthesia | LCA | To assess environmental and financial impacts of reusable and single-use anesthetic equipment | Consequential LCA (ISO 14040) | Hospitals in Australia | Two hospitals in Melbourne, Victoria, Australia (Western Health) | - | Anaesthesiology | The authors declare no conflict of interest. They received funding for the project from the Australian and New Zealand College of anesthetists (project grant 13/025) |
| McPherson et al. (2019) | The impact on life cycle carbon footprint of converting from disposable to reusable sharps containers in a large US hospital geographically distant from manufacturing and processing facilities | PeerJ | CFA using LCA-methods | To assess the climate impacts of two different sharps container systems (disposable and reusable) over a 12 month period at Loma Linda University Health in California, USA, which is located considerably further away from manufacturers and reprocessors than is Northwestern Memorial Hospital (previously studied). | Attributional LCA (PAS 2050) | Hospital in the USA | Loma Linda University Health, San Bernardino, CA, USA | - | Nonspecific | Brett McPherson and Mihray Sharip declare no conflict of interest. Terry Grimmond is an international consultant in sharps injury prevention and waste management to healthcare and associated industries. Daniels Health, the manufacturer did not review, sight or have input into the design, content, methodology, results, write-up of the study or choice of journal for publication.  Daniels Health granted $2500 towards the cost of the study, which covered approximately 10% of expenses. No other grant or funding was received from any funding agency in the public, commercial, or not-for-profit sectors. The funders had no role in study design, data collection and analysis, decision to publish, or preparation of the manuscript. |
| Morris et al. (2022) | Life cycle assessment of stainless-steel reusable speculums versus disposable acrylic speculums in a university clinic setting: a case study | Environmental Research Communications | LCA | To assess the environmental impact of using disposable acrylic speculums versus using reusable stainless-steel speculums. | Attributional LCA (ISO 14040) | Unversity Clinic USA | University of Wisconsin's (UW) Madison campus women's health clinic (part of the University Health Services department) | - | Obstetrics & Gynaecology | The material is based upon work supported by the National Science Foundation Graduate Research Fellowship (Rodriguez Morris) under Grant No. DGE-1747503. This foundation has not reviewed this work, and opinions are from the authors alone. |
| Namburar et al. (2021) | Estimating the environmental impact of disposable endoscopic equipment and endoscopes | BMJ Journals Gut | Other (Cross-sectional study - waste audit) | To measure the amount of waste generated during endoscopic procedures and to understand the impact on waste of changing from reusable to single use endoscopes in the USA. | - | Two academic medical centers in the USA | VA White River Junction, Vermont, USA and Darthmouth Hitchcock Medical Center, New Hampshire, USA | 2020 | Gastro-enterology | The authors declare no conflict of interest and have not received funding. |
| Rizan et al. (2021) | Environmental impact and life cycle financial cost of hybrid (reusable/single-use) instruments versus single-use equivalents in laparoscopic cholecystectomy | Surgical Endoscopy | LCA | To assess environmental and financial impacts of hybrid and single-use instruments in laparoscopic cholecystectomy. | Attributional LCA (ISO 14044), consequential LCA and LCC | Hospital in the UK | - | 2020 | Gastro-enterology | Funded by Surgical Innovations Ltd., but played no pared in scientific conduct, analysis or writing of the manuscript. No conflict of interest was stated. |
| Sanchez et al. (2020) | Environmental and economic comparison of reusable and disposable blood pressure cuffs in multiple clinical settings | Resources, Conservation & Recycling | LCA | To assess the environmental and economic impacts of reusable and disposable blood pressure (BP) cuffs. | Attributional LCA (ISO 14040) | Outpatient clinic and ambulatory procedure rooms, regular ward and ICU in the US | Yale-New Haven Health (YNHH) System in New Haven, Connecticut, USA. | - | Nonspecific | The authors declare no conflict of interest. Funding: Dept. of Civil and Environmental Engineering, Northeastern University |
| Sherman et al. (2018) | Life Cycle Assessment and Costing Methods for Device Procurement: Comparing Reusable and Single-Use Disposable Laryngoscopes | Anesthesia & Analgesia | LCA | To assess the environmental and financial impacts of three different types of rigid laryngoscope handle and tongue blade: plastic single-use, metal single-use, and stainless steel reusable (under a range of cleaning options: low-level disinfection, high-level disinfection, sterilisation) | Attributional LCA (ISO 14040) | Hospital in the USA | Yale-New Haven Hospital, New Haven, CT, USA | - | Anaesthesiology | The authors declare no conflict of interest. J.D.S. was supported by an Anesthesia Patient Safety Foundation award. L.A.R. was supported by a Provost’s award for undergraduate research at Northeastern University. M.J.E. was supported by departmental start-up funds at Northeastern University. |
| Sorensen et al. (2022) | A review of environmental and economic aspects of medical devices, illustrated with a comparative study of double-lumen tubes for one-lung ventilation | Environment, development and sustainability | LCA | To assess the environmental impact of either using a single-use double lumen tube (DLT) combined with a reusable bronchoscope or a single-use DLT with an integrated single-use camera. To perform a literature review to obtain the current state of knowledge regarding the economic and environmental aspects of single-use versus reusable medical equipment. | Attributional LCA (ISO 14040) | Hospital in Denmark | Odense University Hospital | - | Thoracic surgery | The authors declare no financial interest. |
| Unger et al. (2014) | Comparative life cycle assessment of reused versus disposable dental burs | International Journal of Life Cycle Assessment | LCA | To assess the environmental impact of disposable and reusable dental burs. | Attributional LCA (ISO 14040 - 14044) | Dental office in the USA | Deer Valley Smiles dental office | - | Dental care, oral surgery | - |
| Vozzola et al. (2018) | Environmental considerations in the selection of isolation gowns: A life cycle assessment of reusable and disposable alternatives | American Journal of Infection Control (AJIC) | LCA | To assess the environmental impacts of two different isolation gowns: reusable and disposable | Attributional LCA (ISO 14040 - 14044) | USA | - | - | Nonspecific | The American Reusable Textile Association (ARTA) and International Association for Healthcare Textile Management (IAHTM) committees with reusable and disposable firms were essential in providing funding and field information for this study. |
| Vozzola et al. (2020) | An Environmental Analysis of Reusable and Disposable Surgical Gowns | AORN Journal | LCA | To assess the environmental impacts of two types of surgical gown: disposable and reusable | Attributional LCA (ISO 14040 - 14044) | USA | - | - | Nonspecific | All authors declare affiliations that could be perceived as posing a potential conflict of interest (all authors are consultants for the American Reusable Textile Association and the International Association for Healthcare Textiles, and are involved in Environmental Clarity, Inc.)  This study was funded by The American Reusable Textile Association (ARTA) Life Cycle Assessment Committee, Shawnee Mission, KS. |
| ***Topic 3 "Cover materials"*** |  |  |  |  |  |  |  |  |  |  |
| Nowack et al. (2012) *(see S2)* |  |  |  |  |  |  |  |  |  |  |
| ***Topic 4 "Anaesthetics"*** |  |  |  |  |  |  |  |  |  |  |
| Hu et al. (2021) | The carbon footprint of general anaesthetics: A case study in the UK | Resources, conservation & recycling | CFA using LCA-methods | To assess the carbon footprint of sevoflurane, isoflurane, desflurane and intravenous propofol and to provide evidence of the potential impact of Vapour Capture Technology. | Attributional LCA (ISO 14040 - 14044) | UK | - | 2018 | Anaesthesiology | HX, TT and MK were funded from the Innovate UK to the University of Exeter and SageTech Medical Equipment Ltd. The study was conducted independently and without the intervention of SageTech Medical Equipment Ltd. |
| McGain et al. (2021) | Carbon Footprint of General, Regional, and Combined Anesthesia for Total Knee Replacements | Anesthesiology | CFA using LCA-methods | To assess the carbon dioxide equivalent emissions associated with general anaesthesia, spinal anaesthesia and combined (general and spinal) anaesthesia during total knee replacement. | Atributional LCA (ISO 14040) | Hospital Australia | Williamstown Hospital, Western Health, Melbourne, Australia | 2019 | Anaesthesiology | In-kind support (no cash funding) was provided solely from Western Health Anaesthesia Department sources (Melbourne, Australia). The authors declare no conflict of interest. |
| Sherman et al. (2012) | Life Cycle Greenhouse Gas Emissions of Anesthetic Drugs | Anesthesia & Analgesia | CFA using LCA-methods | To assess the environmental impact of 5 anesthetic drugs – sevoflurane, desflurane, isoflurane, N2O and propofol – and to inform clinician drug selection on this basis. | Attributional LCA (Unspecified) | Hospital in the USA | Yale-New Haven Hospital | - | Anaesthesiology | No funding mentioned. The authors state no conflicts of interest. |
| Thiel et al. (2018) | Strategies to Reduce Greenhouse Gas Emissions from Laparoscopic Surgery | American Journal of Public Health (AJPH) | CFA using LCA-methods | To assess the carbon footprint of various sustainability interventions used for laparoscopic hysterectomy. | Hybrid LCA (ISO 14040-44) | Hospital in the USA | Magee-Womens Hospital of the University of Pittsburgh Medical Center (UPMC) | 2016 | Obstetrics & Gynaecology, Anaesthesiology | None stated. |
| Wang et al. (2022) | Assessing the Environmental Carbon Footprint of Spinal versus General Anesthesia in Single-Level Transforaminal Lumbar Interbody Fusions | World Neurosurgery | CFA without LCA-methods | To assess te potential savings in carbon emissions for general anaesthesia verus spinal anaesthesia in transforaminal lumbar interbody fusions (TLIFs) | LCA-method undetermined | Hospital in the USA | Tufts Medical Center, Boston, Massachusetts, USA | - | Anaesthesiology & Neurosurgery | A.Y. Wang was supported by the National Center for Advancing Translational Sciences, National Institutes of Health |
| ***Topic 5 "Operating room air ventilation and air handling systems"*** | |  |  |  |  |  |  |  |  |  |
| Alsved et al. (2018) | Temperature-controlled airflow ventilation in operating rooms compared with laminar airflow and turbulent mixed airflow | Journal of Hospital Infection | Other | To evaluate three types of ventilation systems for ORs with respect to air cleanliness, energy consumption and comfort of working environment as reported by surgical team members. | - | Three different ORs in a hospital in Sweden. | Helsingborg General Hospital, Helsingborg, Sweden | 2015-2016 | Orthopaedics | None stated. |
| Marsault et al. (2021) | Laminar airflow versus turbulent airflow in simulated total hip arthroplasty: measurements of colony-forming units, particles, and energy consumption | Journal of Hospital Infection | Other | To compare how large, high volume, laminar airflow (LAF) and turbulent airflow (TAF) ventilation systems perform during standardized simulated total hip arthroplasty (THA). | - | Operating room, Denmark. | Genthofte Hospital’s Department of Orthopaedic Surgery | 2014-2015 | Orthopaedics | None stated. |

### Table 1b: Methods LCA studies

| Study reference | Methods |  |  |  |  |  |  |
| --- | --- | --- | --- | --- | --- | --- | --- |
| Author and year | Goal and scope | Functional unit | System boundaries | Included stages | Stated excluded components | Inventory database | Allocation |
| ***Topic 1 "Surgical techniques"*** |  |  |  |  |  |  |  |
| Papadopoulou et al. (2022) *(see S2)* |  |  |  |  |  |  |  |
| Power et al. (2012) | To assess the carbon footprint of MIS in the US, to identify its potential role in global warming. However note: the calculation addresses the additional carbon emissions of MIS compared to open surgery. | One year of minimally invasive surgical procedures performed in the United States (2009; n=2,520,223) | Operating theatre door to door (intraoperative period, inferred from text) | Pharmaceuticals (transport, capture and compression included), disposal of disposable plastic instruments | Production, (other) transport, energy use, reuse, non-CO2 pharmaceuticals (all other lifecycle stages were considered equivalent to open surgery/laparotomy and thus excluded) | - | No |
| Thiel et al. (2015) | To assess the environmental emissions of a vaginal, an abdominal, a laparoscopic, and a robotic hysterectomy and thereby inform healthcare professionals and innovators to optimise a more sustainable system, and make informed decisions about the impact on future generations. | One hysterectomy | Operating theatre door to door (intraoperative period), cradle to grave | Production, transport, energy use, pharmaceuticals, reuse, disposal/waste | Infrastructure including machines and building; chemical manufacturing and cleaning products; hot water | USLCI, EcoInvent | Impacts of reusable materials and equipment were apportioned based on estimated lifespan/number of uses. |
| Woods et al. (2015) | To assess the CO2 footprint of robotically-assisted laparoscopy (RA-LSC), laparoscopy (LSC) and laparotomy (LAP). | One endometrial staging procedure | Operating theatre door to door (intraoperative period, inferred from text) | Energy, waste/disposal | Production, transport, pharmaceuticals, reuse | - | No |
| ***Topic 2 "Disposables versus reusables"*** |  |  |  |  |  |  |  |
| Almutairi et al. (2022) | To assess the environmental impact of different PPE before and after COVID-19. No specific goal and scope further mentioned. | Disposable and reusable gown: The PPE use for body protection to treat one patient within one clinical session.  Visor with disposable face shield and reusable visor: The PPE use for eye protection to treat one patient within one clinical session.   *Respiratory protection is left out of the analysis, since there is no reusable versus disposable comparison according to the PICO.* | Cradle to grave | All processes involved in manufacturing, packaging, transportation, reuse, disposal | None stated | EcoInvent | Impacts of reusables were apportioned based on estimated lifespan/number of uses. |
| Boberg et al. (2022) | To assess the environmental impacts of three trocar systems (reusable and single-use, reusable and single-use) and by performing this study with different conditions and assumptions than in a previous study, provide a more robust basis for decision-making. | 500 laparoscopic cholecystectomies | Cradle to grave | Raw material extraction, production, transportation, reuse, recycling and disposal | Material and processes needed to produce the used machines for the sterilisation process | EcoInvent | Water and energy-related impacts in the sterilisation process were allocated based on the trocars share of a fully loaded machine. |
| Boucheron et al. (2022) | To assess the amount of produced waste and for reusables the water used, and quantify the costs associated with the use of disposable or reusable cystoscopes. The goal is to inform about the associated costs and produced waste and used water regarding disposable and reusable cystoscopes for cystoscopies in healthcare. | - | - | Disposal (waste), water use (only for reusables) | Raw material extraction, production/manufacturing, transport, use | - | - |
| Davis et al. (2018) | To assess the environmental impact of single-use and reusable ureteroscopes. | Use of one ureteroscope during one endourologic case | Cradle to grave | Manufacturing, reuse, disposal | - | - | No |
| Donahue et al. (2020) | To assess the environmental impacts of three types of vaginal specula (one single-use and two reusable models), to be able to include environmental impact of these devices in the selection of medical products (next to patient safety, clinical efficacy, ease of use and cost). | Completion of 20 gynaecologic examinations using a speculum | Cradle to grave | Raw material extraction, production, transportation, reuse, and disposal | Excluded components were inks, bulk packaging, autoclave production, illumination pack for plastic specula, and lubrication (expected to have minimal impacts on results) | EcoInvent, IDEMAT, GREET, EPA WARM | No |
| Drew et al. (2021) *(see S2)* |  |  |  |  |  |  |  |
| Eckelman et al. (2012) | Compare the environmental impact of a disposable and a reusable LMA, from cradle to grave, to be able to include the impact on the environment and public health in the decision-making process of device selection. | Maintenance of 40 airways | Cradle to grave | Raw material extraction, production, packaging, transport, reuse, disposal | Excluded components were bulk packaging, machinery, and small components such as inks and labels on the packaging and on the sterilization indicator strips (expected to have negligible impacts) | EcoInvent | No |
| Friedericy et al. (2022) | To test the hypothesis, that in the European situation, the RSC has less environmental impact compared to single-use blue wrap, with the application of closed loop recycling. | Sterile packaging of a standard format instrument tray for 5000 sterilisation cycles. | Cradle to grave | Raw material extraction, transport, manufacturing, use, end-of-life (landfill and recycling). | - | EcoInvent, Idemat | Yes, for recycling |
| Grimmond et al. (2012) | Comparison of contribution of disposable (DSC) or reusable sharps containers (RSC) to the global warming potential (GWP), to help inform hospitals in reducing GHG emissions. | Provision for 100 occupied hospital beds over one year | Crade to grave | Raw material extraction, production, packaging, transport, reuse, disposal | Infrastructure and assets were excluded from both systems ("in accordance with product LCA principles") | GaBi | No |
| Grimmond et al. (2021) | To compare the life-cycle carbon footprint of 12-months usage of SSC with 12 months usage of RSC, to help and inform healthcare facilities to reduce GHG emissions. | Total fill line litres (FLL) of sharps containers needed to dispose of sharps for 1-year period in 40 trusts. | Cradle to grave | Manufacture, transport, decanting and decontamination and treatment and disposal | Capital machinery, infrastructure, vehicle life-cycle, labor, SC contents, non-GHG emissions | GaBi | Yes, annual emissions for RSC manufacturing were determined by dividing total manufacturing GHG by the years of life expectancy. |
| Hicks et al. (2016) | Analysis of the lifecycle impact of the synthesis of nAg, its application to textiles in a hospital setting and laundering of the textile, for this specific and other use scenarios. | 4600 ug of nAg (amount added to hospital gown) Per one wear and laundering (over a lifetime of 75 wearings) | Cradle to grave | Raw materials acquisition, manufacturing, use, end of life | - | EcoInvent | No |
| Hogan et al. (2022) | To assess the climate impact of reusable cystoscopes compared to single-use cystoscopes, with the goal to decrease the impact of healthcare. | - | Cradle to grave | Manufacture, transport, sterilisation, landfill, and incineration. | Repair and maintenance reusable cystoscopes | - | No |
| Ibbotson et al. (2013) | Assess the environmental and financial impacts of three surgical scissors, to compare their eco-efficiency, to be able to include this in purchasing decisions for users. | 4,500 use cycles of surgical scissors during 18 years | Cradle to grave | Raw material extraction, production, packaging, transport, reuse, disposal | - | EcoInvent, and Australian Data 2007 databases | No |
| Keil et al. (2022) *(see S2)* |  |  |  |  |  |  |  |
| Kemble et al. (2022) | To compare the carbon footprint of SU cystoscopes and reusable cystoscopes, to help guide policy decisions and promote environmental stewardship in urology. | - | Cradle to grave | Manufacturing, packaging, use, repair, sterilisation, reprocessing (inclusive PPE used), transportation, disposal | - | - | No |
| Le et al. (2022) | To compare cradle to grave environmental and human health burdens of SDs and RDs, with the goal to assisst in policy decision-making since the large-scale effects on environmental and public health have not been analysed before. | 1 ERCP procedure | Cradle to grave | Production, transportation, disposal, energy use | - | EcoInvent | No |
| Leiden et al. (2020) | To assess whether reusable or disposable surgical instrument sets for single-level lumbar fusion surgeries are advantageous from an environmental perspective. Also, the identification of hotspots for designing future sustainable surgical instruments. | The surgical instrument set required for one single-level lumbar fusion surgery involving the implantation of four screws and two rods | Cradle to grave | Raw material extraction, production, packaging, transport, reuse, disposal | - | EcoInvent | No |
| McGain et al. (2010) | To compare the financial and environmental costs of two commonly used anaesthetic drug trays. | Use of one anesthetic drug tray (+/- use of 2 cotton gauzes and 1 paper towel) | Cradle to grave | Raw material extraction, production, packaging, transport, reuse, disposal | Existing infrastructure for energy extraction and transportation was not included, nor was agricultural machinery, farm establishment, and forest establishment ("acquisition and infrastructure costs of machines or items that are already in place are routinely not included in LCAs"). | EcoInvent | No |
| McGain et al. (2012) | To compare the financial costs and environmental impacts of the life cycles of reusable and single-use venous catheter insertion kits and evaluate what effect the source of electricity has on the CO2 emissions. | Use of one central venous catheter kit to aid insertion of a single-use, central venous catheter in an operating room. | Cradle to grave | Raw material extraction, production, packaging, transport, reuse, disposal | Existing equipment (e.g. washers and sterilizers) were not included; Cotton gauze and antiseptic were not included, because they were common to insertion of all central venous catheters. | EcoInvent | No |
| McGain et al. (2017) | To compare the consequences from changing from one pattern of equipment to another (single-use/reusable), looking whether new labour would be required or where the next kilowatt hour of electricity would be sourced from. Thereby the environmental and financial consequences were defined. | Use of breathing circuits, face masks, LMAs, and direct and videolaryngoscopes at one hospital over one year | Cradle to grave | Raw material extraction, production, packaging, transport, reuse, disposal | Existing oil, gas, mining, energy, and transport infrastructure was not included; Maintenance and depreciation of washers and sterilizers were not included, 'these would be unaltered by the presence or absence of reusable anesthetic equipment'. | EcoInvent | No |
| McPherson et al. (2019) | To compare the climate impacts of two different sharps container systems over a 12 month period, to be able to reduce GHG emissions of hospitals. | Provision of sharps containers at one healthcare facility for one year | Cradle to grave | Raw material extraction, production, packaging, transport, reuse, disposal | Capital machinery and infrastructure, vehicles, labor, sharps container contents, as well as any inputs and outputs that constituted less than 1% or the systems total mass or energy (article cites "British Standards Institute, 2011" PAS 2050 guide) | GaBi | No |
| Morris et al. (2022) | To compare the envrionmental impact in six scenarios for the reusable stainless-steel and disposable acrylic speculums, to be able to aid in decision making. | One year of clinic operation, which is equivalent to conducting 5,000 pelvic exams per year | Cradle to grave | Raw material extraction and manufacturing, use phase and end of life | Transport, labor and overhead within the clinic, and manufacturing autoclave machine for sterilisation. Additional packaging, such as cardboards. | EcoInvent, ULSCI, ELCD | Yes, the impacts are allocated throughout the years of use for the reusable speculum (and different scenarios are included) |
| Namburar et al. (2021) | Quantify waste associated with endoscopic procedures, to understand the impact on waste when changing from reusable to single-use endoscopes. | - | - | Included in waste audit: Pre-procedure area, examination room and post-procedure area | Sharp objects in separate containers | - | No |
| Rizan et al. (2021) | Quantify reduction of the environmental (and financial) impact of hybrid surgical instruments compared to single-use. | The number of three types of instruments (clip appliers, laparoscopic scissors and ports) typically required to perform one laparoscopic cholecystectomy. | Cradle to grave | Raw material extraction, manufacture, transport, disposal, decontamination for reusable components of hybrid instruments | Other reusable instruments and consumables used to perform a laparoscopic cholecystectomy | EcoInvent, Industry data | Yes, hybrid instruments within a set comprise a certain percentage of the set, therefore this percentage is apportioned to the environmental harm of decontamination. |
| Sanchez et al. (2020) | To compare the environmental and economic performance for reusable and disposable BP cuffs, with a focus on cuff design and materials, cleaning agents and processes. This because disposables come into favour despite lack of information about environmental costs. | Providing blood pressure readings for a clinic room or ward, under four different health care delivery scenarios. | Cradle to grave | Materials and manufacturing, transport, usage, cleaning, disposal | - | US-EI database (which includes USLCI and EcoInvent data adjusted for the US energy system), US EPA database | No |
| Sherman et al. (2018) | To obtain environmental and financial impacts, since it is not clear, with the goal to facilitate anaesthesiologists making the best choice considering environmental and economic perspectives. | One handle or one blade for a single patient encounter | Cradle to grave | Raw material extraction, production, packaging, transport, use, reuse, disposal | Machinery and capital equipment; building operations | EcoInvent, US-EI database | No |
| Sorensen et al. (2022) | To obtain the environmental impact of either using a single-use double lumen tube (DLT) combined with a reusable bronchoscope or a single-use DLT with an integrated single-use camera and aid in decision making for healthcare providers. | One-lung operated patients, for whom single-lung ventilation was achieved using lung seperation with a DLT | Cradle to grave | Raw material extraction, manufacturing, use, disposal/recovery | Water-related impacts and toxicity impacts. Monitor of the single-use tube with integrated camera. | EcoInvent | No |
| Unger et al. (2014) | To evaluate the trade-offs regarding the environment between reused and disposable dental burs. | One reusable dental bur (maximum reuses 30) and the equivalente FU was the production and disposal of 30 disposable dental burs. | Cradle to grave | Manufacturing, packaging, transport, reuse, and disposal | Electricity use during the bur use. Manufacturing, production and delivery of the autoclave and ultrasonic machines were omitted. | EcoInvent, ULSCI, ELCD | Yes, for loading assumptions for the autoclave |
| Vozzola et al. (2018) | (1) to compare 4 environmental impacts (energy, global warming potential, water use, and solid waste consumption) of reusable and disposable isolation gowns; (2) to clearly show what parts of the life cycle are important to the result; and (3) to provide a sensitivity analysis for important parameters. | 1,000 isolation gown uses | Cradle to grave | Resource extraction, gown manufacture, gown use and/or reuse in healthcare settings, to end-of-life disposal. | The study did not include other medical textiles used in healthcare settings such as gloves, wipes, or masks. | Environmental Clarity | No |
| Vozzola et al. (2020) | Assessment of environmental impacts of disposable versus reusable surgical gowns, to provide transparant, scientific and complete environmental comparisons. | 1,000 uses of an extra large, single‐piece, long‐sleeved surgical gown in an operating room setting | Cradle to grave | Raw material extraction, production, packaging, transport, use, reuse, disposal | - | Environmental Clarity | No |
| ***Topic 3 "Cover materials"*** |  |  |  |  |  |  |  |
| Nowack et al. (2012) *(see S2)* |  |  |  |  |  |  |  |
| ***Topic 4 "Anaesthetics"*** |  |  |  |  |  |  |  |
| Hu et al. (2021) | The carbon footprint of general anaesthetics at the national level is presented to inform policy. | 1 minimum alveolar concentration hour (MAC-h), or MAC-h equivalent for propofol. | Cradle to grave | Raw material extraction, manufacturing, packaging, use, waste gases | Transport, energy consumptions of using general anaesthetics in the OR, disposables from inhalational anaesthetic gases (IAG) use (assumed to be equal). Waste from propofol use. | EcoInvent | No |
| McGain et al. (2021) | To aim to quantify the carbon dioxide equivalent emissions of general and spinal and combined anaesthesia to reduce GHG production and reduce the threat of climate change in healthcare. | All anaesthesia for a total knee replacement. | Cradle to grave | Raw material extraction, production, transport, drug usage phase, disposal | HVAC and surgical equipment data. | EcoInvent, the Australian Life Cycle Inventory | No |
| Sherman et al. (2012) | To compare the environmental impacts of 5 anaesthetic drugs to inform clinicians in drug selection. | 1 minimum alveolar concentration (MAC), or MAC-equivalent for propofol, for maintenance anesthesia for an average 70 kg adult patient for 1 hour (1 MAC-h) | Cradle to grave | Raw material extraction, production, transport, drug delivery, disposal | Baseline energy requirement for the anaesthesia machine (considered to be constant for all drugs), basic disposables (such as: endotracheal tubes, circuits, CO2 absorbents) were considered to be equivalent. | EcoInvent | No |
| Thiel et al. (2018) | To examine the efficacy of interventions to reduce GHG emissions in the OR with the goal to improve the emission rate of the healthcare sector and thereby human health. | One laparoscopic hysterectomy | Cradle to grave, intraoperative period | Production, transport, energy use, pharmaceuticals, reuse, disposal | Infrastructure including machines and building; chemical manufacturing and cleaning of products; hot water use | EcoInvent, USLCI | Yes, impacts of reusable materials and equipment were apportioned based on estimated lifespan/number of uses. |
| Wang et al. (2022) | To compare the use of spinal anaesthesia versus general anaesthesia in TLIF and to quantify the carbon footprint | - | Cradle to gate | Raw material extraction, production | Waste/disposal | Existing literature | No |
| ***Topic 5 "Operating room air ventilation and air handling systems"*** |  |  |  |  |  |  |  |
| Alsved et al. (2018) | - | - | - | - | - | - | - |
| Marsault et al. (2021) | - | - | - | - | - | - | - |

### Table 1c: Methods LCA studies

| Study reference |  |  |  |  |  |  |  |  |
| --- | --- | --- | --- | --- | --- | --- | --- | --- |
| Author and year | Normalisation and weighting | Impacts reported | Contribution analysis | Scenario analysis | Comparative analysis | Sensitivity analysis | Uncertainty analysis | Variance analysis |
| ***Topic 1 "Surgical techniques"*** |  |  |  |  |  |  |  |  |
| Papadopoulou et al. (2022) *(see S2)* |  |  |  |  |  |  |  |  |
| Power et al. (2012) | No | Yes, only climate impacts considered | Yes | No | No | No | No | No |
| Thiel et al. (2015) | No | Yes (graphically) | Yes | No | Yes | No | Yes (Monte-Carlo) | No |
| Woods et al. (2015) | No | Yes, only climate impacts considered | Yes | No | Yes | No | No | Yes (ANOVA) |
| ***Topic 2 "Disposables versus reusables"*** |  |  |  |  |  |  |  |  |
| Almutairi et al. (2022) | Yes, potential per person per year | Yes | Yes | No | Yes | No | No | No |
| Boberg et al. (2022) | No | Yes | Yes | No | Yes | Yes | Yes (Monte-Carlo) | No |
| Boucheron et al. (2022) | - | - | - | - | Yes | - | - | - |
| Davis et al. (2018) | No | Yes, only climate impacts considered | Yes | No | Yes | No | No | No |
| Donahue et al. (2020) | No | Yes, only climate change impacts | Yes | No | Yes | Yes, sensitivity analysis reports impacts based on different numbers of uses (1-500), autoclave loading practices, regional electricity grids, reprocessing method (autoclave vs H2O2) | No | No |
| Drew et al. (2021) *(see S2)* |  |  |  |  |  |  |  |  |
| Eckelman et al. (2012) | No | Yes | Not graphically, but hotspots are reported in text | No | Yes | Yes, tests alternative assumptions including transport mode, autoclave loading, number of reuse cycles (10-100), waste pathways, and labour | No | No |
| Friedericy et al. (2022) | No | Yes | Yes | Yes (two end-of-life scenarios) | Yes | Yes | No | No |
| Grimmond et al. (2012) | No | Yes, only climate impacts considered | Yes | No | Yes | Yes; tests impact of distribution distances by assuming RSCs were made at the DSC facility, and vice versa; tests impact of equal sized container volumes; tests impact of alternative electricity grids; tests transport vehicle load capacity; tests alternate disposal methods, e.g. shredding. | No | Yes |
| Grimmond et al. (2021) | No | Yes, only climate impacts considered | Yes | No | Yes | Yes; tests impact of larger vehicle size, transport distances, polymer and container manufacturing geographies, larger SSC container size and changing the lifespan from a base of 18 years to 1 year, theoretically maximum of 66 years and the ‘break-point’ at which life span RSC GWP matches SSC GWP. | No | No |
| Hicks et al. (2016) | No | Yes | Yes | Yes | Yes | Yes | Yes | No |
| Hogan et al. (2022) | No | Yes, only climate change impacts considered | Yes | No | Yes | No | No | No |
| Ibbotson et al. (2013) | No | Yes, graphically with log scale | Only for ReCiPe endpoint and CED results | No | Yes | Yes, tests alternative electricity mixes, sterilisation processes (gamma and gas), disposal method (incineration and recycling) | No | No |
| Keil et al. (2022) *(see S2)* |  |  |  |  |  |  |  |  |
| Kemble et al. (2022) | No | Yes, only climate change impacts considered | Yes | Yes | Yes | No | No | No |
| Le et al. (2022) | Yes, endpoint results were normalised to an average person's impact | Yes, climate change impacts, human health impact, ecosystems impact, and resource consumption, other impacts reported in supplementary material | Yes | Yes | Yes | Yes, to test the range of infection rates | No | No |
| Leiden et al. (2020) | No | No | Yes | No | Yes | Yes, tests alternate assumption, including: number of usage cycles for the reusable set (300-500) and loan (distributor rechecks and replaces missing components between each use) vs. consignment system (i.e. in-hospital reprocessing with requests to distributor for missing components) | No | No |
| McGain et al. (2010) | No | Yes, climate change impacts and water use | Yes, although alignment between reported contributions and lifecycle stages not totally clear. | No | Yes | No | Yes (Monte-Carlo) | No |
| McGain et al. (2012) | No | Yes, climate change impacts and water use impacts reported | Yes | No | Yes | Yes, tests altered electricity source for the reusable kit: brown coal (base case), gas cogeneration, American standard supply, European standard supply | Yes (Monte-Carlo) | No |
| McGain et al. (2017) | Yes, results were normalised to average annual per capita environmental impacts in Australia. | Yes | No | Yes | Yes | No | Yes (Monte-Carlo) | No |
| McPherson et al. (2019) | No | Yes, only climate change impacts considered | Yes | No | Yes | Yes, reducing reusable container lifespan, alternate electricity grids, reprocessing optimisation. | No | Yes |
| Morris et al. (2022) | No | Yes (graphically in breakeven graphs) | Yes | Yes | Yes | Yes | Yes (Monte-Carlo) | No |
| Namburar et al. (2021) | No | No, only the amount of waste | Yes | Yes | Yes | No | No | No |
| Rizan et al. (2021) | No | Yes | Yes | Yes | Yes | Yes, in the analysis they altered electricity source decontamination and changed way of transport | No | No |
| Sanchez et al. (2020) | No | Yes | Yes | Yes | Yes | Yes | No | No |
| Sherman et al. (2018) | No | Yes | Yes | Yes, various cleaning options | Yes | Yes, assuming a 100% recycling scenario | No | No |
| Sorensen et al. (2022) | No | Yes (graphically) | Yes | Yes, different scenarios with recycling or incineration of waste | Yes | No | No | No |
| Unger et al. (2014) | No | Yes (normalised) | Yes | Yes | Yes | No | No | No |
| Vozzola et al. (2018) | No | Yes, only climate impacts, blue water, natural resource energy and solid waste | Yes | Yes | Yes | Yes, only described in text (a 10% decrease in weight of disposables; decreasing from 63 to 45 grams per gown; a 10% decrease of reusable gown weight; a 10% decrease in laundry energy consumption) | No | No |
| Vozzola et al. (2020) | No | Yes, only climate impacts, blue water, natural resource energy and solid waste | Yes | No | Yes | Yes, modelled 0% and 100% reuse of end-of-life reusable gowns in other industries; if disposable gowns were instead manufactured in the US, 10% more energy efficient laundry processes. | No | No |
| ***Topic 3 "Cover materials"*** |  |  |  |  |  |  |  |  |
| Nowack et al. (2012) *(see S2)* |  |  |  |  |  |  |  |  |
| ***Topic 4 "Anaesthetics"*** |  |  |  |  |  |  |  |  |
| Hu et al. (2021) | No | Yes, only climate impacts considered | Yes | Yes | Yes | No | No | No |
| McGain et al. (2021) | No | Yes, only climate impacts considered | Yes | No | Yes | Yes | Yes (Monte-Carlo) | No |
| Sherman et al. (2012) | No | Yes, only climate impacts considered | Yes | Yes | Yes | No | No | No |
| Thiel et al. (2018) | No | Yes, only climate impacts considered | Yes | Yes | Yes | No | No | No |
| Wang et al. (2022) | No | Yes, only climate impacts considered | No | Yes | Yes | No | No | No |
| ***Topic 5 "Operating room air ventilation and air handling systems"*** |  |  |  |  |  |  |  |  |
| Alsved et al. (2018) | - | - | - | - | - | - | - | - |
| Marsault et al. (2021) | - | - | - | - | - | - | - | - |

### Table 1d: Data LCA studies

| Study reference | Data |  |
| --- | --- | --- |
| Author and year | Data collection | Characterisation method |
| ***Topic 1 "Surgical techniques"*** |  |  |
| Papadopoulou et al. (2022) *(see S2)* |  |  |
| Power et al. (2012) | Minimally invasive surgeries’ CO2 footprint is based on used CO2 for insufflation (transport, capture, compression, packaging and use) and disposal of surgical instruments (laparoscopic trocars). All other emissions were considered equivalent to open surgery.  For transportation the Memorial Sloan-Kettering Cancer was used as index case. Number of miles for CO2 cylinders were calculated. Carbon footprint calculator based on U.S. Department of Transportation (US DOT) fuel efficiency data and Greenhouse Gas Protocol initiative (GHGPI) mobile guides were used to estimate the carbon emissions. Carbon dioxide capture and compression were calculated using the Input-Output Life cycle assessment (EIO-LCA) model (i.e. based on economic activity of the largest medical CO2 supplier). To calculate the annual use of CO2 for MIS the number of procedures were identified in national databases. Average operative times were estimated based on institutional data. Number of cylinders and CO2 emissions were calculated from this data. Data for the number of disposable instruments, specifically laparoscopic trocars, were obtained from US market engineering research (2004). Weight of a laparoscopic trocar was estimated. Data for robot-assisted procedures were based on Intuitive Surgical procedure numbers, instrument catalogue unloaded weights an using a general rule of 10 uses before disposal. The carbon footprint was estimated with the assumption that incinerating 1 kg of plastic produces approximately 6 kg of CO2. | - |
| Thiel et al. (2015) | This research used a hybrid LCA framework, by incorporating process LCA data and Economic Input Output LCA (EIO-LCA) data. Monte Carlo simulations were used to quantify the variability and uncertainty in emissions for each component of a hysterectomy.  Waste audits were conducted from the surgeries of patients that underwent an abdominal, a laparoscopic or a robotic hysterectomy for noncancer related reasons. For the course of 1 year, waste from 62 cases of each type of hysterectomy were quantified and characterized (15 abdominal, vaginal and robotic, 17 laparoscopic).  Materials were matched with the most relevant unit process within either USLCI and EcoInvent. For complex items of medical equipment an economic input-output approach was taken (i.e. based on the price paid for each item). Patient records were used to calculate both pathogenic waste (uterine weight) and quantities of anesthesia and abdominal insufflation. Transportation impacts were calculated based on distances between facilities, according to waste hauling data from the relevant facility. Impacts of resuable materials and equipment were based on estimated lifespan/number of uses. Data on the sterilisation process for reusable materials and linens was sourced from the literature. | TRACI |
| Woods et al. (2015) | 150 staging procedures (50 per arm) for endometrial cancer for the three surgical modalities were reviewed. Collected information: patient age, body mass index (BMI), procedure type, operative time, history of prior abdominal surgery, length of stay, uterine weight and instruments used. Waste production and energy expenditure were determined for each surgical modality and included in the analysis. Solid waste was determined by weighing items used within procedures (drapes, gowns, gloves, consumables, sterile wrap, single-use devices) and multiplying weights by use data taken from OR records. Energy consumption was determined by using operative time records and multiplying duration of the procedure per unit time of four categories: environmental (HVAC, lighting), equipment, instruments, robotic system.   Data on energy and waste GHG equivalents were obtained from the National Energy Foundation (NEF), the US Energy Information Administration, and previous carbon footprinting studies. Assumed was that 1kg waste in a municipal landfill is considered equivalent to 1 kg CO2 equivalents released into the enviroment. Energy in kWh was converted using the NEF calculator conversion factor of 0.54 kg CO2eq/kWh. | - |
| ***Topic 2 "Disposables versus reusables"*** |  |  |
| Almutairi et al. (2022) | The EcoInvent database was utilised. The products selected for this research were based on PPE guidelines, and differences in guidelines before and after COVID-19 were considered. PPE products were gathered from clinics at Dublin Dental University Hospital (DDUH). The assessment covered manufacturing, packaging, transportation, and disposal for single-use products. For reusables, additional considerations were made, including requirements for reuse such as washing and disinfection. For transportation the shortest routes were included. PPE items were weighed, and various materials were identified per product. | ReCiPe 2016 |
| Boberg et al. (2022) | Both LCA and LCC methods were utilised. Data collection for the LCA included the use of the EcoInvent database. Data for the specifics of the trocars, processes at the hospital and hospital waste management were obtained using questionnaires sent to the practitioners. Input data for trocars was provided by the manufacturer or EcoInvent database, when not provided plastic materials were assumed to be either polycarbonate or high-density polyethylene (included in sensitivity analysis). Transportation distances were calculated using Google Maps. Different modes of transportation were included in a sensitivity analysis. Individual trocar package data was available from the manufacturer (reusable trocar system). Sterilisation wrap data was based on information from the index hospital. Getinge AB, the manufacturer of the sterilisation machines proved data on water, detergents and electricity. A sensitivity on elecricity-mix was performed. Conventional LCC was used to calculate financial costs. | IMPACT 2002+ |
| Boucheron et al. (2022) | To assess the environmental impact the amount of waste and water use was evaluated. Pre- and post-procedure waste was gathered and evaluated by mass. The water consumption for the reprocessing of reusable cystoscopes was reported by volume. The annual volume of water and waste was calculated. Descriptive statistics were outlined for the available variables. | - |
| Davis et al. (2018) | The environmental impact of single-use flexible ureteroscopes with reusable flexible ureteroscopes were compared. An LCA of the LithoVue (Boston Scientific) single-use digital flexible ureteroscope and Olympus Flexible Video Ureteroscope (URV-F) was performed. Data on manufacturing, reuse and disposal of the instruments was obtained. The solid waste generated (kg) and energy consumed (kWh) during each case were quantified and used to calculate the CO2 footprint. Data on equivalent mass of CO2 per kg of material manufacturing was obtained from previous studies, as well as data of CO2 eq for solid waste disposal, repackaging, repair, and sterilisation. Disposal was assumed to be 1kg CO2 eq per kg solid waste. Washing and Sterilisation impacts for reusable ureteroscopes were estimated based on the specifications and energy requirements of an Olympus ETD4™ endoscope washer disinfector. Repair impacts were calculated as 5 kg CO2 equivalents per repair. | - |
| Donahue et al. (2020) | Life cycle assessment methods were applied to evaluate the carbon footprints of 3 vaginal specula: a single-use acrylic model and two reusable stainless steel models (reusable stainless steel grade 304 speculum and the reusable stainless steel grade 316 speculum). The data were obtained regarding speculum and packaging composition and weight. There were no data available on production processes for the specula. For this reason, assumptions were made. For the acrylic specula injection molding was assumed and for the reusable specula a combination of hot extrusion, milling/turning, deformation and heat treatment was assumed, based on literature. The transportation was based on manufacturer and general industry data. Reuse for the steel reusable specula was estimated based on autoclave manufacturer specifications. Disposal was modeled with the use of the EPA WARM model, which estimates the average greenhouse gas (GHG) emissions that are associated with disposal of various materials in the United States (US). | IPCC |
| Drew et al. (2021) *(see S2)* |  |  |
| Eckelman et al. (2012) | The environmental impacts of two types of laryngeal mask airways (LMAs): single-use (Unique™) and reusable (Classic™; 40 lifetime uses) were assessed by using a life cycle assessment method. Raw material extraction, production, packaging, transport, reuse and disposal were included in the analysis. The material composition and weights were established on the basis of manufacturer information and density testing. Materials were matched with the most appropriate Life Cycle Inventory (LCI) records from EcoInvent (database). Production processes for hard and soft plastics were assumed to be injection molding and thermoforming, respectively. Data was obtained from distributors to estimate distances and mode of transport. Reprocessing of reusable LMAs was estimated using data from Yale New Haven Hospital and autoclave specifications. Disposal was modelled using US average statistics for solid waste. | BEES |
| Friedericy et al. (2022) | The carbon footprint, ReCiPe endpoints and eco-costs of the RSC and blue wrap were assessed. Data was collected regarding the materials, fabric and the production process. Travel distances were estimated using Google Maps. Data on sterilisation was gathered (the amount of energy needed to heat the packaging material, and washing process based on the PG8930 Miele washing machines). Two end of life scenarios were calculated (open and closed loop recycling for blue wrap, and landfill or recycling for the metals of the RSC). | IPCC, ReCiPe, eco-costs ISO 14008:2019 |
| Grimmond et al. (2012) | An LCA framework was used to assess the climate impacts of two different sharps container systems (disposable and reusable) over a 12-month period. Data was collected regarding the size, type, and number of containers used, as well as modification protocols. Both systems were taken into account from cradle to grave. The data comes from a variety of industry and government sources and combined with an LCI/LCA tool developed by the Waterman Group UK. | IPCC |
| Grimmond et al. (2021) | The climate impacts of hospitals converting from single-use sharps containers (SSC) to reusable sharps containers (RSC) were compared by using an attributional LCA model. The intervention in this study was conversion from SSC to RSC. Twelve months of usage of SSC was compared with twelve months usage of RSC. SSC and RSC usage details in 17 baseline trusts immediately prior to 2018 were applied to the RSC usage details of the 40 trusts using RSC in 2019. GWP was calculated in carbon dioxide equivalents (CO2 equivalents) generated in the manufacture, transport, service and disposal of 12 months, hospital-wide usage of both sharps containment systems in the 40 trusts. | IPCC |
| Hicks et al. (2016) | An LCA was conducted to compare the environmental impact of reusable patient hospital gowns coated with nAg product compared to the use of disposable gowns. First, the environmental impact of synthesis and attachment of 4600 ug nAg was determined (the amount added to a hospital gown). Second the life cycle impacts of nanoscale silver (nAg)-enabled reusable hospital gowns per one wear are modelled and midpoint environmental data are compared. | TRACI |
| Hogan et al. (2022) | Total 40 cystoscopies were observed (20 single-use and 20 reusable). The lifespan for a reusable cystoscope was 7 years. CO2 production for sterilisation was obtained from Davis et al. (2018), a study conducted in Australia. Weights were recorded using a scale, and the total waste was coverted to carbon dioxide equivalents, based on mass and way of disposal (data from HSE, waste management awareness handbook). Manufacturing costs (CO2 equivalents) were also based on the study from Davis et al. (2018). Transport was calculated using data from Hopkin (2008). Data analysis was performed using Stata. | - |
| Ibbotson et al. (2013) | The environmental and financial impacts of three surgical scissors which are (1) disposable scissors made of plastic (fibre reinforced), (2) disposable scissors made of stainless steel and (3) reusable scissors made of stainless steel were assessed using a life cycle assessment and life cycle costing method. The data was compared for the use of 4,500 cycles if usage in Germany. The data on raw material, manufacturing (including electricity consumption), transport, and disposal process were obtained from a medical company in Europe. Missing data (e.g. sterilisation processes for reusable scissors) were obtained from the literature or expert opinion. Electricity data that was missing was adjusted from the International Energy Agency (IEA). Incineration of plastics, cardboard and municipal solid waste were assumed based on Swiss plants in 2000 (from EcoInvent). For stainless steel recycling, data from Australian data 2007 was used (which may not reflect the German setting). | CED, ReCiPe |
| Keil et al. (2022) *(see S2)* |  |  |
| Kemble et al. (2022) | Manufacturing data for SU cystoscopes was obtained from manufacturers of the cystoscopes and confirmed using a previous study. However, authors of this previous study (Davis et al.) claim to have used 'standardized carbon footprint protocol guidelines,' but no specific reference to the protocol is provided. The authors of this study also refer to a 'standardised CO2 protocol', which is unclear what the protocol is. For reusables dat were obtained from an insitutional device performance database, but no reference is given in the methodology section.  Data on packaging, transportation, sterilisation, incineration, repair, solid waste and energy use were obtained from previous studies. | - |
| Le et al. (2022) | Three duodenoscopes were evaluated: conventional reusable duodenoscope (RD), an RD with disposable endcaps and a single-use duodenoscope (SD). The weights were obtained from the manufacturers. Values of composition were obtained from available literature data (Davis et al.). Electricity use during the procedure was calculated based on the manufacturer's manual. The RDs were assumed to be reused 125 times per year for 5 years (totalling 625 reuses). The average energy consumption for reprocessing was recorded using a kilowatt meter. Detergent use was also included. | ReCiPe |
| Leiden et al. (2020) | The difference in contribution to the environmental impact of a disposable and a reusable surgery instrument set for lumbar fusion surgeries in Germany is investigated. It is important to take into account the content of the tray; the reusable set consists of 45.5 kg per set, and depending on the requirements for the surgery only a part of the set is applied. The disposable set is only 2.0 kg.  Data on manufacturing was based on weight, material and form of instruments, data of transportation on mode and calculated distances between producer, distributor, and hospital and washing and steam sterilization data was specific to a German hospital. Disposal was modelled using EcoInvent waste incineration processes. Outcome measures were climate change and acidification.  The disposable and reusable set are compared for 1 surgery. There has been a correction for the reusable set; only a three hundredth of the weight is taken into account for the production and disposal processes. The analysis would reflect the reality more if the functional unit would have been 300 surgeries.  For comparison purposes it is also important to note that sterilisation was performed outside the hospital. A sensitivity analysis has been conducted, wherein the reusable set has been reused multiple times, and there has been a shift from the loaner system to the hospital's consignment of the instruments. This results in a reduced need for disinfection and sterilisation, requiring only one-time sterilisation instead of the previous combination of sterilisation, cleaning, and disinfection.  The study was funded by Neomedical S.A., the producer of the disposable instrument set. | CML, ReCiPe |
| McGain et al. (2010) | The financial and environmental costs of two commonly used anaesthetic drug trays were modelled using LCA. This study was performed at the Western Hospital in Melbourne, Victoria. The reusable tray, the single-use tray and the single-use tray with cotton and paper were compared. Data was collected directly from measurements and from databases (EcoInvent). The single-use trays were plastic Chinese-made trays and the reusable trays were Australian made nylon trays. Since not all data was directly available, and some data were also not available as average data, for the single-use trays the European energy mix is used, however the Chinese energy mix might be more coal reliant. Reusable kits were assumed to have a lifespan of 300 uses (sharpening of metal was required every 100 uses) based on an estimate from staff. | - |
| McGain et al. (2012) | The environmental and financial impacts of two type of central venous catheter insertion kits (single-use and disposable) were assessed at the Western Health group of hospitals in Melbourne, Victoria, Australia. Next to the environmental and financial impacts, they investigated the effect of the source of electricity upon CO2 emissions. The functional unit was the use of one central venous catheter kit to aid insertion of a single-use, central venous catheter in an operating room. Data on the components of the central venous catheter kits was obtained by weighing with an electronic balance and receiving data from the manufacturer. Direct data regarding materials and energy required to reprocess reusable kits (i.e. from the washer and steriliser) were collected using a 'time-in-motion' study. Most other inputs were acquired from LCI databases or industry data. Electricity requirements (kWh) and volumes of hot (gas heated) and cold water used by the washer and steriliser were measured. Data on waste disposal processes were obtained indirectly from industry data (sodium hypochlorite or incineration). | - |
| McGain et al. (2017) | The environmental and financial impacts of reusable and single-use anesthetic equipment were assessed through the exploration of 2 base cases and 3 modelled scenarios using a consequential LCA approach. The first base case was situated at a hospital in Melbourne, Australia with "mainly single-use" anesthetic equipment (reusable anesthetic circuits, face masks, ‘Proseal’VR (Teleflex, Westneath, Ireland) LMAs, and direct and videolaryngoscope blades and handles. The second base case was situated at another hospital in Melbourne, Australia with "mainly single-use" anesthetic equipment (disposable anesthetic circuits, single-use face masks, LMAs, and direct laryngoscope blades, but using reusable direct laryngoscope handles and reusable videolaryngoscopes). The five scenarios included: "completely single-use", "reusables except for single-use face masks", "reusables except for single-use laryngoscope blades", "reusables (Europe)", "reusables (USA)". Data on equipment were obtained from two hospitals in Melbourne, Australia in 2015 and each piece of equipment was weighed with an electronic balance (accurate to within 1g). Sterilization records and input from senior Central Sterile and Supply Department staff at hospital 1 were used to define sterilization mode and load information. Washer and steam sterilizer utility usage data were taken from a previous study by the same authors (0.15 kWh and 40 litres of water per kg of anesthetic equipment steam sterilized), while electricity consumption of a standard H2O2 sterilizer was directly measured over several days at hospital 1. | - |
| McPherson et al. (2019) | This study followed a very similar methodology as that by Grimmond et al., 2012, including the use of the same cradle-to-grave LCI and calculation tool. The disposable sharps containment system was assessed for a 12 month period prior to Loma Linda University Health's (LLUH) transition to a reusable-based system. The reusable system (certified for 500 uses) was assessed for another 12 month period two years later once the transition was complete. Data were directly collected from LLUH regarding size, type, and number of containers used, as well as changeout protocols. Disposable sharps containers (DSCs) were made from US-sourced polymer in Illinois, packaged in cardboard, transported 3,200km to LLUH, and autoclaved and landfilled in California post-use. Reusable sharps containers (RSCs) were made in Michigan from Korean-sourced polymer, transported 3,500km in reusable transport containers, and reprocessed in California 440km from LLUH. Instead of normalising the results to occupied beds, as was the case in Grimmond et al., 2012, total 'Adjusted Patient Days' was instead used as the workload indicator to which results were normalised. | IPCC |
| Morris et al. (2022) | The environmental impacts of the acrylic disposable speculum and reusable stainless steel in different scenarios. Data was collected using different databases (EcoInvent, ELCD an USCLI). The composition of the material from the disposable speculum was assumed since it was not provided by the manufacturer. For the reusable speculum, the lifetime was gathered through information from the clinic, but different lifetime acquisition and replacement options were modelled. The exact type of stainless steel could not be determined, therefore an assumption was made. Energy consumption of the autoclave process was directly measured. Other data regarding the use phase (washing, disinfecting and sterilisation) was also determined in the clinic by direct observations/measurements. | TRACI |
| Namburar et al. (2021) | An audit of waste was performed during endoscopic procedures at a low and high endoscopy volume academic medical center (VA White River Junction, Vermont, USA and Darthmouth Hitchcock Medical Center, New Hampshire, USA) over a 5-day work period in 2020. Colonoscopies, upper endoscopies and endoscopic retrograde cholangiopancreatography (ERCP) were included. The waste from the pre-procedure area, examination room and post-procedure area was collected and documented as mass and volume. In the high volume hospital the waste from endoscope reprocessing was also obtained. An estimation of the contribution of single-use (compared to reusable) waste was made in the following three scenarios: (1) all reusable endoscopes, (2) colonoscopies and ERCPs were performed with single-use endoscopes (colonoscopes/duodenoscopes) and (3) all single-use endoscopes. | - |
| Rizan et al. (2021) | The environmental and financial impacts of hybrid and single-use instruments in laparoscopic cholecystectomy were assessed using life cycle assessment. The number of three types of instruments (clip appliers, laparoscopic scissors and ports) typically required to perform one laparoscopic cholecystectomy were included in the analysis (two small diameter ports, two large diameter ports, one laparoscopic scissor and one laparoscopic clip applier). The stages of raw material extraction, manufacture, transport, disposal and decontamination for reusable components of hybrid instruments were included. Data was obtained from manufacturers and databases. | ReCiPe |
| Sanchez et al. (2020) | The environmental and economic impacts of reusable and disposable blood pressure cuffs were assessed by using life cycle assessment. Data on materials and manufacturing was gathered through a combination of manufacturer information and physical testing, by weighing components on a scale. Components were identified and matched with information from inventory databases (US-EI LCI database). US EPA database was used for transport packaging information. Multiple cleaning scenarios were developed to represent a diversity of clinical settings in using and cleaning. Only landfill and incineration were included for disposal data and recycling was not taken into account (“as recycling is uncommon (though possible) given the types of plastics an mixed materials employed in the BP cuffs”). | TRACI |
| Sherman et al. (2018) | The environmental and financial impacts of three different types of rigid laryngoscope handles and tongue blades were assessed: plastic single-use, metal single-use, and stainless steel reusable (under a range of cleaning options: low-level disinfection, high-level disinfection, sterilisation) by using life cycle assessment and life cycle costing at the Yale-New Haven Hospital, New Haven, CT, USA. To determine the material composition of handles and blades a combination of manufacturer specifications, deconstruction, and density testing were used, and after each material was weighed. Foreground data specific to Yale-New Haven Hospital (YNHH) were collected, including transportation mode and distance; washer and autoclave-related energy, water, and chemical use (based on machine specification and apportioned based on an assumed full-load). Reusable components were assumed to have a lifespan of 4000 uses and require refurbishment every 40 uses, according to rated lifetimes of each component (i.e. 1/4000th of the manufacturing, transportation, and disposal impacts were assigned to 1 use of a reusable device). Standard US waste management was assumed: 6% of plastics are recycled, 30%–70% of metals, and remaining solid waste is either landfilled (80%) or incinerated (20%). | TRACI |
| Sorensen et al. (2022) | Data on processing and material use were used from EcoInvent. The background system is based on the Danish system for heat and electricity rates. Hospital waste is 99% incinerated (hazardous waste) with energy recovery. Data on reprocessing the reusable bronchoscope was assessed from the procedure in a Danish hospital (Odense University Hospital). Material composition of a tube combined with a reusable bronchoscope was estimated based on weight. For the single-use tube with integrated camera it is not clear if it was estimated, but weights of different materials are shown. The monitor is reusable and is not included in the analysis. Transport distances were assumed to be 12,000 km. | - |
| Unger et al. (2014) | Primary data were directly collected, including: operating paramaters for ultrasonic cleaning (voume of water and energy consumed), parameters for manual washing (water used), and operating parameters for the autoclave (water and energy use). Secondary data for raw mateiral extraction and production were collected fom databases. The packaging of the burs was obtained and weighed. The Arizona electricity mix was modeled using exicsting data by the US Energy Information Administration (EIA). The burs were manufactured in Germany and transported by air freight and truck. Distances were calculated using Google Maps. Electricity use during the bur use was not included, since it was assumed to be identical for both burs. The water and electiricty data for cleaning (ultrasonic machine) were obtained from independent trials, where the electricity and water use were directly measured. For the autoclave, data was obtained from the manufacturer. Manufacturing, production and delivery of the autoclave and ultrasonic machines were omitted. It was assumed that the ultrasonic and autoclave machines loading only consisted of dental burs. | TRACI |
| Vozzola et al. (2018) | An LCA was conducted to assess the environmental impacts of two different isolation gowns: reusable and disposable. The functional unit was 1000 isolation gowns uses. This study is an analysis from cradle to grave including manufacturing, use and end-of-life stages of the gown systems. The Environmental Clarity, Inc. LCA database was used to evaluate the life cycles of both isolation gown systems. Sixteen disposable isolations gowns from 5 suppliers were studied, composed primarily of nonwoven polypropylene fabric. Eight reusable isolation gowns were studied, composed of primarily woven polyester fabric. The outcome measures were climate change and waste. | - |
| Vozzola et al. (2020) | LCA of reusable versus disposable gowns to assess the environmental impact of these surgical gowns in the USA. An LCA was conducted according to the standards from the International Organization for Standardization. The Environmental Clarity, Inc, LCA database was used to evaluate the life cycles of both surgical gown systems. The reusable gown was manufactured in Canada using European materials, while the disposable gown was produced in China. Subsequently, both types of gowns were transported to the United States. It was assumed that disposable gowns underwent sterilisation using ethylene oxide before use. The reusable gowns were assumed to have a lifespan of 60 uses. To determine the reprocessing-related impacts for reusable gowns, data on energy and water use from 21 North American laundry companies were employed. Additionally, both types of gowns were assumed to end up in landfills, and the transport between landfill and laundry facilities was taken into consideration. | - |
| ***Topic 3 "Cover materials"*** |  |  |
| Nowack et al. (2012) *(see S2)* |  |  |
| ***Topic 4 "Anaesthetics"*** |  |  |
| Hu et al. (2021) | A life cycle assessment was conducted to calculate the carbon footprint of general anaesthetics. Thereby the potential impact of Vapour Capture Technology was provided. The functional unit for the general anaesthetics in the LCI analysis was 1 minimum alveolar concentration hour (MAC-h), or MAC-h equivalent for propofol. Values of 2.2%, 1.2% and 6.7% respectively for sevoflurane, isoflurane and desflurane were used as basis of the modelling. Raw material extraction, manufacturing, packaging, transport, use and disposal were included in the analysis. Ecoinvent was used as an inventory database. Since information on synthesizing general anaesthesic agents was not publicly available, two methods were modelled for the manufacturing process of the drugs. Method ‘A’: relatively older processes and method ‘B’: newer processes in manufacturing. Synthesis of propofol liquid was included. Transportation was assumed to be similar, and therefore excluded from the analysis, as was energy consumption for using general anaesthetics in the OR, the use of disposables and propofol end of life waste. For the Vapour Capture Technology effect, it was assumed that IAG can only be recycled once. Two stages were employed: 1) IAG is used for 1 MAC-h, 70% is recycled and 2) recycled drug with manufactured drug is used for another MAC-h. Results are shown for US and UK scenarios; general scenario where NO2 is used as a carrier gas and two scenarios were it is not used. The outcome measure was climate change. | - |
| McGain et al. (2021) | An LCA was conducted to examine the carbon dioxide equivalent emissions associated with general anaesthesia (propofol and sevoflurane), spinal anaesthesia and combined (general and spinal) anaesthesia during total knee replacement. The functional unit was all anaesthesia for a total knee replacement. Anaesthesia data were obtained from 30 patients undergoing total knee replacement in an Australian hospital. Data from literature and databases such as EcoInvent and Australian Life Cycle Inventory were used. | - |
| Sherman et al. (2012) | An LCA was conducted to compare the environmental impact of 5 types of anaesthetic drugs – sevoflurane, desflurane, isoflurane, N2O and propofol – to inform clinician drug selection on this basis. The functional unit was 1 minimum alveolar concentration (MAC), or MAC-equivalent for propofol, for maintenance anesthesia for an average 70 kg adult patient for 1 hour (1 MAC-h). Included stages in the life cycle of the drug were raw material extraction, production, transport (to health care facilities), drug delivery (to the patient) and disposal. Besides that, the waste gas of the agent in the atmosphere and N2O release were considered (O2/air admixture and N2O/O2 admixture for administration were considered). Data collection on transport, drug transportation, energy requirements and disposal was specific to the Yale-New Haven Hospital. EcoInvent was used as primary data source. When data regarding the drugs was unavailable in EcoInvent, proxies that best matched the production characteristics of the drug were used. | Sulbaeck et al. 2010, IPCC |
| Thiel et al. (2018) | A hybrid LCA was conducted to examine the efficacy of sustainable interventions to reduce GHG emissions in the OR. Baseline emissions for laparoscopic hysterectomy were calculated from an average of 17 hysterectomies extracted from a previous study in the USA (Thiel, 2017). Further data was obtained from EIO-LCA and EcoInvent. Life cycle GHGs were calculated for interventions regarding anaesthetics (type of anaesthetic), surgical materials and energy. To model anaesthetic interventions, an average anaesthetic duration of 150 minutes was assumed. | TRACI |
| Wang et al. (2022) | A retrospective study was conducted with a cohort of 100 patients who underwent single-level TLIF to quantify the carbon footprint of the use of general anaesthesia versus spinal anaesthesia. The data was gathered from a single surgeon, who shifted practice after 50 times the use of general anaesthesia to the use of spinal anaesthesia. Anaesthetic agents were gathered from intraoperative anaesthesia records. For intravenous, intramuscular, and oral agents the total amount of the medication that was used was gathered, and for inhalational agents, the used mass was calculated. The carbon footprint was calculated using the GWP100 from previous literature. | - |
| ***Topic 5 "Operating room air ventilation and air handling systems"*** |  |  |
| Alsved et al. (2018) | Comparison: a) Vertical laminar airflow (LAF) and turbulent mixed airflow (TMA) b) Temperature-controlled airflow (TcAF)  Cfu concentrations were measured at three locations in an OR between January 2015 and February 2016 during 45 orthopaedic procedures: 1. Close to the wound (<40 cm) 2. At the instrument table  3. Peripherally in the room  A questionnaire answered by the OR team evaluated the comfort of the working environment. Energy consumption was evaluated. | - |
| Marsault et al. (2021) | Comparison of: a) Laminar airflow (LAF)  b) Turbulent mixed airflow (TMA) During total hip arthroplasty surgery (THA).   Measurements were taken at two ORs between October 2014 and January 2015 at two identical full-size operating theatres. One was equipped with a large, high volume LAF system and the other with TAF ventilation. Total 32 simulations were included. LAF uses 2 HEPA filters, airflow rate of fresh air is 2760 m3/h and recirculated air is 7075 m3/h. TAF ventilation uses 1 HEPA filter and airflow rate is 2533 m3/h, which is all outside air. Primary outcomes were comparison of particle counts, cfu, and energy consumption between LAF and TAF at 100% fresh air influx. Secondary, same parameters were evaluated but with different comparisons: LAF100 and LAF50, TAF100 and TAF50, LAF50 and TAF100. | - |

### Table 1e: Results LCA studies

| Study reference | Results |  |
| --- | --- | --- |
| Author and year | Outcomes per included outcome | Interpretation |
| ***Topic 1 "Surgical techniques"*** |  |  |
| Papadopoulou et al. (2022) *(see S2)* |  |  |
| Power et al. (2012) | 1. Climate Change • CO2 emission used for insufflation: 303 tonnes CO2 • CO2 emission from CO2 capture/compression: 351,400 tonnes CO2. Wherein 251,000 can be attributed to industrial gas manufacturing, 83,700 to power generation and supply and 16,700 to gas extraction.  • CO2 emission used for transportation: 2970 tonnes CO2  • CO2 emission from incineration of disposable instruments (laparoscopic trocars and robotic instruments): 1251 Tonnes CO2  Total CO2 emissions from minimally invasive surgery were estimated at 355,924 tonnes/year more in comparison to open surgery.   2. Waste A total of 208,441 kg of plastic biomedical waste from disposable trocar and laparoscopic instrument use for MIS per year. This consisted of 186,000 kg of plastic from laparoscopic trocars (6,200,000 trocars) and 22,441 kg of plastic from robotic instruments.   3. Acidification No results in this study.   4. Eutrophication No results in this study.   5. Human Toxicity No results in this study.   6. Ecotoxicity No results in this study.   7. Ozone Depletion No results in this study. | This LCA of MIS in the USA, shows that the biggest contributor in CO2 emissions is the capture/compression of CO2 for insufflation. The industrial gas manufacturing in this part contributes most, followed by the actual use of the CO2 for insufflation, the transportation of the gas and the incineration of the disposable instruments. However, given the limitations of this study, e.g. the different methodologies used, the results should be interpreted with caution. |
| Thiel et al. (2015) | 1. Climate change This study reported a difference in greenhouse gas (GHG) emissions between the four approaches for a hysterectomy. Robotic hysterectomies have the highest impact (100%) in greenhouse gas emissions, followed by the laparoscopic (65-70%), abdominal (35-40%) and vaginal approach (30-35%). The laparoscopic approach resulted in 30-35% less contribution to GHG emissions than a robotic approach, abdominal and vaginal approaches in between 60-70% less contribution in comparison to the robotic approach.  Biggest contributors were: the use of anaesthetic gases (in all surgical modalities) and single-use surgical instruments (for laparoscopic and robotic approach). Other contributors were production of single-use materials (gowns, gloves etc.) and energy use (mainly HVAC).   2. Waste The robotic approach for a hysterectomy produced the highest amount of waste, following the laparoscopic, abdominal and vaginal approaches. Robotic hysterectomies produced 13.7 kg municipal solid waste (MSW) per case, which resulted in a total of 30% more waste compared to the average of the other approaches. This consisted of 22% by weight by gowns, drapes and bluewrap (SMS PP), 50% gloves and other plastics, 18% paper and 5% cotton. Abdominal hysterectomies had an average of 9.2 kg of MSW production and produced the largest amount of cotton waste (blue towels, laparotomy pads) at 1 kg per average surgery (11% of the MSW by weight). Across all four surgeries, SMS PP material were the majority of the MSW by weight, from 22% of the weight of a robotic hysterectomy and 35% for a laparoscopic hysterectomy. Other types of plastics, from thin film packaging wrappers to hard plastic trays, made up a minimum of 36% of the total MSW weight for vaginal hysterectomies and a maximum of 46% for robotic hysterectomies.  3. Acidification The robotic approach had the highest impact on acidification (100%), followed by the laparoscopic (70-75%), abdominal (25%) and lastly the vaginal approach (20%).   The great difference between the robotic and laparoscopic approach in comparison to the vaginal and abdominal approach can be attributed to the use of single-use surgical instruments (contribution of the production of single-use surgical instruments).   4. Eutrophication The robotic approach had the highest impact on eutrophication (100%), followed by the laparoscopic (70-75%), abdominal (60-65%) and lastly the vaginal approach (55%).   Greatest contributors are disposal of MSW, and production of single-use surgical instruments and single-use materials.  5. Human Toxicity The impact was divided into two subcategories: carcinogenic and non-carcinogenic potential. The robotic approach had the highest impact in both carcinogenic and non-carcinogenic potential categories (100%). The carcinogenic and non-carcinogenic impact of the laparoscopic approach resulted in respectively 80% and 85-90%. For the abdominal approach 90-100% and 80-90% and for the vaginal approach 80% and 70-75%.   Production of single-use materials contributed most in all surgical modalities.   6. Ecotoxicity The robotic approach had the highest impact on ecotoxicity (100%), followed by the laparoscopic (90-95%), abdominal (90%) and lastly the vaginal approach (75%).   Production of single-use materials contributed most in all surgical modalities.   7. Ozone Depletion The robotic approach had the highest impact on ozone depletion (100%), followed by the laparoscopic (60-65%), abdominal and the vaginal approach (0-5%).   The production of single-use surgical instruments contributed most. | This life cycle assessment shows the biggest environmental impact is attributable to the robotic and laparoscopic surgical approaches for a hysterectomy. Within these modalities, the production of single-use instruments, single-use materials (gowns, gloves, etc.), the use of anesthetic gases are the biggest contributors to the environmental impact, and to a lesser extent in these impact categories the use of energy (HVAC) and the disposal of MSW. |
| Woods et al. (2015) | 1. Climate change The CO2 footprint for all 150 endometrial staging procedures, including solid waste and energy consumed, was corresponding with 4498 CO2eq, averaging 30 kg CO2eq/patient.  A robotically-assisted laparoscopy (RA-LSC) procedure resulted in a CO2 footprint of 40.3 kg CO2eq/patient, based on energy use and waste. The CO2 emission from this energy use was 26 kg CO2eq, calculated by energy use (kWh) and operative time (min).  A laparoscopy (LSC) procedure emitted 29.2 kg CO2e/patient. The CO2 emission from this energy use was 18 kg CO2eq. A laparotomy (LAP) resulted in the emission of 22.7 kg CO2eq/patient. The CO2 emission from this energy use was 14.4 kg CO2eq.  2. Waste The RA-LSC group produced a total amount of 14.3 kg (14.3 kg CO2eq/patient) of solid waste, which existed of 6.90 kg consumable waste (6.90 kg CO2eq/patient), 2.47 kg single-use device waste (2.47 kg CO2eq/patient), 4.03 kg infection control waste (4.03 kg CO2eq/patient) and 0.88 kg waste from sterile wraps (0.88 kg CO2eq/patient).  The LSC group produced 11.2 kg (11.2 kg CO2eq/patient) of solid waste, which existed of 6.03 kg consumable waste (6.03 kg CO2eq/patient), 3.35 kg single-use device waste (3.35 kg CO2eq/patient), 1.60 kg infection control waste (1.60 kg CO2eq/patient) and 0.99 kg waste from sterile wraps (0.99 kg CO2eq/patient).  The LAP group produced 8.3 kg (8.3 kg CO2eq/patient) of solid waste, which existed of 5.86 kg consumable waste (5.86 kg CO2eq/patient), 0.82 kg single-use device waste (0.82 kg CO2eq/patient), 1.60 kg infection control waste (1.60 kg CO2eq/patient) and 0.44 kg waste from sterile wraps (0.44 kg CO2eq/patient).   The solid waste production of RA-LSC represented a 74% increase over LAP and a 36% increase over LSC.  3. Acidification No results in this study.   4. Eutrophication No results in this study.   5. Human Toxicity No results in this study.   6. Ecotoxicity No results in this study.   7. Ozone Depletion No results in this study. | Comparing the three surgical modalities, the RA-LSC turns out to have the biggest CO2 footprint. Emissions from both energy use and waste are greatest in the RA-LSC group. The energy use of all groups seem comparable, but because of the use of the Da Vinci robot, the RA-LSC energy consumption turns out highest. The energy use for equipment is highest in the LSC group.   The biggest contributors in waste in the RA-LSC group are consumables and infection control waste, in the LSC group consumables and single-use devices and in the LAP group consumables.   Energy use adds more to the CO2 footprint than waste.  Results should be interpreted with caution due to the limitations (see limitations). |
| ***Topic 2 "Disposables versus reusables"*** |  |  |
| Almutairi et al. (2022) | Disposable vs. reusable gown 1. Climate change Climate change is 101% higher for disposable gowns .  2. Waste No results in this study.   3. Acidification Freshwater and terrestrial acidification is 57% higher for disposables compared to reusables.  4. Eutrophication Freshwater eutrophication is 56% higher for disposables. Terrestrial eutrophication is 25% higher for disposables. Impact in marine eutrophication was higher for reusables (+/- 50% (interpreted from figure 1)).   5. Human Toxicity No results in this study.  6. Ecotoxicity No results in this study.   7. Ozone Depletion Ozone layer depletion was higher for reusables compared to disposables (+/- 50% (interpreted from figure 1)). The impact for both reusable and disposable lies between 0 and 0.00002 kg CFC11 equivalents per person per year.   Disposable vs. reusable visor 1. Climate change The reusable visor accounts for 19.44% of the total climate change impact caused by the disposable visor (100%).  2. Waste No results in this study.   3. Acidification Higher burden for disposables compared to reusables for freshwater and terrestrial acidification (+/- 75% more than reusables (interpreted from figure 3).  4. Eutrophication Higher burden for disposables compared to reusables for freshwater eutrophication (+/- 50% more than reusables (interpreted from figure 3), for terrestrial eutrophication (+/- 75% more than reusables (interpreted from figure 3), and for marine eutrophication (+/- 75% more than reusables (interpreted from figure 3).  5. Human Toxicity No results in this study.   6. Ecotoxicity No results in this study.   7. Ozone Depletion Higher burden for disposables compared to reusables for ozone layer depletion (+/- 66% more than reusables (interpreted from figure 3). | Regarding disposable and reusable gowns, the disposable gown had a higher impact in 8 from a total of 13 impact categories, including fossils, climate change, freshwater eutrophication, freshwater and terrestrial acidification, photochemical ozone creation, terrestrial eutrophication, respiratory effects (inorganics), and ionising radiation. The results show that 99% of human health damage comes from global warming and water consumption, where global warming was the main contributor for disposable gowns and water consumption for reusable gowns. Water dissipation was way five times higher for the reusable gown. For disposables, highest contributors in climate change impacts were hazardous waste incineration and the market for polyethylene and polypropylene. For the impact on fossils, the main contributor was the manufacturing process (especially for polyethylene and polypropylene). On most impacts, reusables favor disposables; however, it should be carefully considered from which material the gown is made and the weight of the gown before interpreting these results for your own facility. Increasing weight increases the impact, and cotton as a raw material has a higher environmental impact instead of, for example, polyester. However, it is important to note that when reused this can result in lower overall impacts.   For the reusable vs. disposable visor, the disposable visor had the highest impact in all environmental impact categories. The disposable face shield has a four time higher impact compared to the reusable visor. The reusable visor is thereby a environmentally better choice. |
| Boberg et al. (2022) | 1. Climate change The single-use trocar system’s impact on climate change was 379% higher than the reusable system’s impact and 12% higher than the mixed system’s impact [median difference of 446 kg CO2eq (413–483) and 55 kg CO2eq (25–87), respectively]  2. Waste No results in this study.   3. Acidification The single-use trocar system’s impact on aquatic acidificiation was 315% higher than the reusable system’s impact and 13% higher than the mixed system’s impact [median difference of 1.3 kg SO2eq (1.2-1.5) and 0.22 kg SO2eq (0.13-0.34), respectively] The single-use trocar system’s impact on terrestrial acidificiation was 329% higher than the reusable system’s impact and 13% higher than the mixed system’s impact [median difference of 4.8 kg SO2eq (4.4-5.7) and 0.9 kg SO2eq (0.5-1.8), respectively]  4. Eutrophication The single-use trocar system’s impact on aquatic eutrophication was 150% higher than the reusable system’s impact and 25% higher than the mixed system’s impact [median difference of 0.03 kg PO4 P-lim (0.02-0.05) and 0.008 kg PO4 P-lim (0.003-0.016), respectively]  5. Human Toxicity The single-use trocar system’s impact on non-carcinogens was 60% higher than the reusable system’s impact and 11% lower than the mixed system’s impact [median difference of 2.5 kg C2H3CL eq (-0.4-6) and -1.6 kg C2H3CL eq (-4.7-0.9), respectively] The single-use trocar system’s impact on non-carcinogens was 437% higher than the reusable system’s impact and 22% higher than the mixed system’s impact [median difference of 18 kg C2H3CL eq (17-19) and 4.3 kg C2H3CL eq (3.6-4.9), respectively]  6. Ecotoxicity The single-use trocar system’s impact on aquatic ecotoxicity was 3% higher than the reusable system’s impact and 12% lower than the mixed system’s impact [median difference of 2160 kg TEG water (-23100-14400) and -4650 TEG water (-29300-4190), respectively] The single-use trocar system’s impact on terrestrial ecotoxicity was 2% lower than the reusable system’s impact and 17% lower than the mixed system’s impact [median difference of 274 kg TEG soil (-6670-4000) and -1730 kg TEG soil (-7350-756), respectively]  7. Ozone Depletion The single-use trocar system’s impact on ozone layer depletion was 50% higher than the reusable system’s impact and 6% higher than the mixed system’s impact [median difference of 5.4*10^-6 kg CFC-11 eq (-4*10^-8-1.9*10^-5) and 1.1*10^-6 kg CFC-11 eq (-2.2*10^-6-8.1*10^-6), respectively] | The single-use system has the highest impact on the majority of the midpoint categories, compared to the reusable and mixed trocar system.  The production and packaging of all three systems contributed most to the resource endpoint, as well as for the contribution to climate change. Production also contributed for a great part to the ecosystem quality endpoint, but the sterilisation for the mixed an reusable system also contributed here. Production of all three systems contributed greatly on the human health endpoint. Overall, it can be interpreted that the production phase in the life cycle seems to be the most important contributor in the endpoints impact for all three systems, and also sterilisation for the reusable and mixed systems.   The impact of the single-use system compared to reusable remained larger on climate change and human health in all sensitivity analyses. For the resource and ecosystem quality endpoints, this difference changes when using a fossil heavy electricity mix in the sterilisation process. The ecosystem quality endpoint also changed negatively for the reusable system when reducing the numer of reuses, and increased allocation in the sterilisation process. The mixed system remained larger than reusable in all sensitivity analyses on all climate change, human health and resources. Regarding ecosystem quality, the mixed system was sensitive for the increased allocation in the sterilisation process. Overall, it seems that the electricity mix, number of reuses, allocation in the sterilisation process and also the change to air freight as main mode of transportation play important factors in the overall environmental impact regarding the three systems, and should be considered per specific hospital/setting before implementing the reusable system. |
| Boucheron et al. (2022) | 1. Climate change No results in this study.  2. Waste The reusable cystoscope produced 800 grams of waste per procedure. The disposable cystoscope produced 200 grams of waste.   3. Acidification No results in this study.  4. Eutrophication No results in this study.  5. Human Toxicity No results in this study.  6. Ecotoxicity No results in this study.  7. Ozone Depletion No results in this study. | The reusable flexible cystoscope used in cystoscopy results in four times more waste compared to the single-use flexible cystoscope.  In this study, the waste produced is considered, but the entire life cycle of the product is not examined (e.g., raw material extraction, production/manufacturing, transport, use, and disposal). Readers should exercise caution as this is a waste audit; drawing conclusions about environmental impact based solely on this data, which includes only waste and water use, may be challenging.  Furthermore, specific details about the type of waste are not provided. This aspect could be further be defined to enable the evaluation and optimisation of different strategies for reducing (unnecessary) waste. It is possible that more waste is being generated than necessary. |
| Davis et al. (2018) | 1. Climate Change The CO2 footprint per case was calculated. For the single-use ureteroscope the total CO2 footprint per case is 4.43 kg CO2 equivalents. This consisted of manufacturing costs, solid waste and sterilisation. The manufacturing costs resulted in 3.83 kg CO2, solid waste in 0.3 kg CO2 and sterilization 0.3 kg CO2 .  The total CO2 footprint of the reusable ureteroscope was 4.47 kg CO2 per case. This consisted of manufacturing costs (0.06 kg CO2), washing/sterilisation (3.95 kg CO2), repackaging theatre wrap (<0.005 kg CO2), repair costs (0.45 kg CO2) and solid waste (0.005 kg CO2).    2. Waste Solid waste for the disposable ureteroscope resulted in 0.3 kg CO2 per case.   Solid waste for the reusable ureteroscope resulted in 0.005 kg CO2 per case.   3. Acidification No results in this study.   4. Eutrophication No results in this study.   5. Human Toxicity No results in this study.   6. Ecotoxicity No results in this study.   7. Ozone Depletion No results in this study. | The study suggests that the environmental costs of disposable and reusable ureteroscopes are comparable. However, it is important to consider that the study was conducted in Australia, where the energy mix is predominantly brown coal, unlike in regions such as Europe or the USA. This could place reusable ureteroscopes at a greater disadvantage. Additionally, the results should be interpreted with caution. While the entire life cycle appears to be included, the data was derived from previous studies without applying a Life Cycle Assessment (LCA) methodology. This reliance on external data introduces limitations, as it involves differing assumptions and contexts from other settings. It is therefore also not clear of the data from the previous studies is all based on an Australian setting or not. |
| Donahue et al. (2020) | 1. Climate Change Donahue (2020) demonstrated the reusable grade 304 speculum produces lower CO2 emissions than the equivalent number of disposable acrylic specula after 2 completed examinations (2.11 kg CO2eq compared to 2.63 kg CO2eq). The reusable grade 316 produces fewer CO2eq after 3 completed examinations (3.11 kg CO2e compared to 3.51 kg CO2e). The reusable stainless steel grade 304 speculum is less carbon intensive to produce compared to the grade 316 speculum, which is the reason why the grade 304 remains less in its total life cycle CO2e emissions over a wide range of uses.   After 500 examinations the difference becomes more apparent (grade 316 – 107.52, grade 304 – 101.31 and acrylic – 438.55 kg CO2e).   The contribution of the stages differs between the specula. The largest contributor for the disposable acrylic speculum is material production and manufacturing (90.6%), followed by transportation (6.5%) and waste/end-of-life (2.9%). For the reusable stainless steel grade 304 speculum the largest source of CO2 emissions is use/reprocessing (74.1%), followed by material production and manufacturing (24.9%) and transportation (0.46%). The biggest contributor in total life cycle emissions for the grade 316 speculum was use/reprocessing (65.2%), followed by production (34.4%) and transportation (0.4%).  2. Waste No results in this study.   3. Acidification No results in this study.   4. Eutrophication No results in this study.   5. Human Toxicity No results in this study.   6. Ecotoxicity No results in this study.   7. Ozone Depletion No results in this study. | The study shows the disposable acrylic speculum has the a greater climate change impact. This is mainly due to material production and manufacturing. This phase offers opportunities to decrease this impact.   For the reusable stainless steel specula the main contributor is the energy used to power autoclaves. Here is an opportunity to reduce this by increasing the efficiency of energy-use and by making a transition to more renewable energy sources.   In the sensitivity analysis it became clear that the impact increased significantly when shifting to individually sterilising the specula, instead of sterilising multiple at the same time (increase of 189-219%). However, doubling the autoclave load (4 Pouches (base case) to 8 Pouches (full load)) did not have a great difference in the overall impact (20-39% decrease in greenhouse gas emissions).   Changing from the most carbon intensive electricity grid to the least carbon intensive resulted in a 33-36% reduction of CO2e emissions. Regardless of the grid used, the stainless steel life cycle greenhouse gas emission remained lower than the acrylic specula.   Using high level disinfectant instead of autoclave sterilisation, resulted in a 11-12% increase in greenhouse gas emissions. |
| Drew et al. (2021) *(see S2)* |  |  |
| Eckelman et al. (2012) | 1. Climate Change Eckelman (2012) demonstrated the results on climate change specifically to be 7.4 kg CO2e of GHG over its life cycle for the reusable LMA and 11.3 kg CO2e for the disposable LMA. For all outcomes in this study, results are expressed in percentages, whereas the LMA with the highest impact is defined as 100% and the other LMA is relatively compared to the LMA with the highest impact. For the outcome climate change, the disposable LMA had the highest impact (100%) compared to the reusable LMA (65%). The largest source for the disposable LMA is the polymerization of PVC (23%), which is the main material used. The majority of the remaining contributors are polycarbonate production (14%), transportation via truck (15%), thermoforming (13%) and waste disposable (11%). The majority of the GHG emissions for the reusable LMA (77%) is from natural gas production and combustion, which is to produce steam for the autoclave.  2. Waste No results in this study.   3. Acidification For the outcome acidification, the disposable LMA had the highest impact (100%) compared to the reusable LMA (20-30%).  4. Eutrophication For the outcome eutrophication, the disposable LMA had the highest impact (100%) compared to the reusable LMA (90-100%).  5. Human Toxicity The human toxicity, stated as human health (HH) in this study, was defined in three different groups: HH cancer, HH noncancer and HH air pollutants. For the outcome HH cancer, the disposable LMA had the highest impact (100%) compared to the reusable LMA (0-10%). For the outcome HH noncancer, the disposable LMA had the highest impact (100%) compared to the reusable LMA (0-10%) and the outcome HH air pollutants, resulted in the highest impact for yet the disposable LMA (100%) compared to 20-30% for the reusable LMA.   6. Ecotoxicity For the outcome ecotoxicity, the disposable LMA had the highest impact (100%) compared to the reusable LMA (10-20%).  7. Ozone Depletion For the outcome ozone depletion, the disposable LMA had the highest impact (100%) compared to the reusable LMA (20-30%). | This study demonstrates the disposable LMA has a bigger environmental impact compared to the reusable LMA. In the outcome measure climate change, this is mainly due to the production of the material for the disposable LMAs that is used. A change of material production, or a change in type of material which has a lesser impact on the environment could be a way to help reduce the impact for the disposable LMA. Next to that the biggest contributor for the reusable LMA is the production of steam for the autoclave. If this could be done in some other way, the environmental impact of the reusable LMA could decrease.   Alternate assumptions are also made in this study. It shows the effect of alternate modes of transport, compared to the base case (rail), was quite small for the reusable LMA but more interesting for the disposable LMA, leading to a decrease in GHG emissions (-9%) changing to transport by road, and an increase (+81%) by using air transportation.   Individually autoclaving the reusable LMA resulted in an increase of life cycle GHG emissions by >400%, whereas loading with 10 LMAs per cycle (compared to the base case 5 per cycle) resulted in a decrease of 25%. Using a more capital intensive option to increase the energy efficiency of the machines by 10% results in a decrease of GHG emissions of 8%.   The human toxicity impacts are dominated by the production and use of plastics for the disposable LMA. Increasing the amount of PVC by 10% leads to a 5% increase in cancer and noncancer effects.   Premature disposal of the reusable LMA has its direct effects on GHG emissions, by a >50% increase if the LMA is disposed at 10 reuse cycles. Extending the reuse cycle of reusable LMAs to 80 cycles (doubling lifetime) results in a decrease of GHG emissions by 9%.   In waste management, by switching from 100% incineration to 100% landfill, reduces the impacts across all categories by 5-10%.   Including the labor for cleaning impacts (base case not included) resulted only in a nominally increase for total GHG emissions and water impacts of reusable LMAs. |
| Friedericy et al. (2022) | 1. Climate Change Blue wrap with open loop recycling: 1869 kg CO2eq/5000 cycles. Blue wrap with closed loop recycling: 883 kg CO2eq/5000 cycles.  RSC landfill: 285 kg CO2eq/5000 cycles.  RSC recycling: 270 kg CO2eq/5000 cycles.   2. Waste No results in this study.   3. Acidification No results in this study.   4. Eutrophication No results in this study.   5. Human Toxicity No results in this study.   6. Ecotoxicity No results in this study.   7. Ozone Depletion No results in this study. | The use of RSC compared to blue wrap results in a decrease of the carbon footprint, eco-costs and ReCiPe endpoints. Main contributors are the use phase for the RSC, and for the blue wraps the production phase. Open loop compared to closed loop recycling had a considerable impact for the blue wrap regarding carbon footprint, however this was minimal for the RSC. |
| Grimmond et al. (2012) | 1. Climate Change Annual greenhouse gas (GHG) emissions resulted in of 139.1 metric tons of (MT) CO2 equivalents for DSC and 25.1 MTCO2 equivalents for reusable sharps containers (RSC). Stratified to 100 hospital beds over one year this resulted in 24.2 MTCO2 equivalents per 100 OB-year for DSC and 4.0 MTCO2 equivalents per 100 OB-year for RSC. Use of RSC reduces climate impacts by 83.5%.   2. Waste Annual waste for DSC resulted in 30,920 kg landfilled plastic and 5020 kg of cardboard boxes for 34,396 manufactured and 33, 759 landfilled DSC (chemotherapy DSC were incinerated). Whereas RSC only caused 123 kg of plastic waste (calculated for the end of life of the RSC, during the study no RSC were landfilled) and 116 kg of waste from carboard boxes (this were the chemotherapy DSC, which were used in both systems if there was an indication for chemotherapy). In total 2481 RSC were manufactured and 47 containers were landfilled.   3. Acidification No results in this study.   4. Eutrophication No results in this study.   5. Human Toxicity No results in this study.   6. Ecotoxicity No results in this study.   7. Ozone Depletion No results in this study. | Use of RSC leads to reduction of climate impacts and waste.  The manufacturing process is the biggest contributor in climate impacts for DSC, and thereby gives the largest difference between the two containment systems. This is a function of resin weight; container manufacturing and low annual RSC manufacturing emissions because of their long lasting life span.   The washing process is the biggest contributor for RSC. Decanting and washing contributed for 52.5% of the systems total climate impacts.   The sensitivity analysis showed that the choice of a ‘clean’ electrical source (e.g. windfarm vs. coal) can alter manufacturing climate impacts by 15% in the US. Thereby, it showed that water usage in RSC processing was associated with 40% of this process and reduction of water volumes would reduce climate impacts.   Reclamation of energy and material will reduce manufacturing GWP in both systems.   Costs were reduced by 19.2% by using RSC. |
| Grimmond et al. (2021) | 1. Climate Change Annual greenhouse gas (GHG) emissions in 40 trusts resulted in a climate impact of 3896.4 metric tons of (MT) CO2 equivalents for SSC and 628.9 MTCO2 equivalents for RSC (-83.9%).  2. Waste Annual waste for SSC resulted in 928.7 kg incinerated plastic and 136.6 kg of cardboard boxes for 1 748 851 manufactured and 1 748 851 incinerated SSC. Whereas RSC were not incinerated – all parts were either reused or recycled. Waste in the RSC study-year came from SSCs used in study-year.   3. Acidification No results in this study.   4. Eutrophication No results in this study.   5. Human Toxicity No results in this study.   6. Ecotoxicity No results in this study.   7. Ozone Depletion No results in this study. | Use of RSC leads to reduction of climate impacts and waste.  The manufacturing process is the biggest contributor in climate impacts for SSC, and thereby gives the largest difference between the two containment systems.   Transport is the biggest contributor for RSC. It resulted in 442 MT CO2 equivalents of the total of 628.9 MT CO2 equivalents annually for 40 hospital trusts.  The sensitivity analysis showed that that changes achieved by changing processes/geography within life stages, were not mirrored in the final climate impacts comparisons, which in all but one alternative scenario did not achieve changes for more than 5%. This was the RSC lifespan of 1 year, which was an academic exercise and is not expected in real life. Using larger vehicles for transport and optimisation for reprocessing medical devices is recommended to lower GHG. |
| Hicks et al. (2016) | 1. Climate Change Results using nanosilver (nAg) as an antimicrobial agent for patient hospital gowns. Given the observed loss of nAg, the silver could be reapplied at each set of 17 launderings for reusable gowns and needed to be reapplied for every single disposable gown. Greenhouse gas (GHG) emissions for synthesis of 4600 ug nanosilver resulted in a climate impact of 1.17 x 10-3 kg CO2 equivalents. Nanosilver attachment resulted in 7.90 x 10-2 kg CO2 equivalents per hospital gown.   2. Waste No results in this study.   3. Acidification Acidification for synthesis of 4600 ug nanosilver resulted in 9.99 x 10-4 mol H+ equivalents. Nanosilver attachment resulted in 2.66 x 10-2 mol H+ equivalents per hospital gown.   4. Eutrophication Eutrophication for synthesis of 4600 ug nanosilver resulted in 5.83 x 10-5 kg N equivalents. Nanosilver attachment resulted in 2.63 x 10-4 kg equivalents per hospital gown.   5. Human Toxicity Human toxicity in carcinogenics for synthesis of 4600 ug nanosilver resulted in 4.66 x 10-10 CTUh. Nanosilver attachment resulted in 4.28 x 10-9 CTUh per hospital gown.   Human toxicity in non-carcinogenics for synthesis of 4600 ug nanosilver resulted in 6.37 x 10-9 CTUh. Nanosilver attachment resulted in 4.28 x 10-8 CTUh per hospital gown.   6. Ecotoxicity Ecotoxicity for synthesis of 4600 ug nanosilver resulted in 2.36 x 10-2 CTUe. Nanosilver attachment resulted in 1.51 x 10-1 CTUe per hospital gown.   7. Ozone Depletion Ozone depletion for synthesis of 4600 ug nanosilver resulted in 1.29 x 10-10 kg CFC-11 equivalents. Nanosilver attachment resulted in 5.70 x 10-9 kg CFC-11 equivalents per hospital gown. | Nanosilver (nAg) can be used for patient hospital gowns due to its antimicrobial nature.   The results show it is necessary to synthesize the nAg and thereafter attach the silver to the gown. The impact is greater to attach the nAg to the textile than it is to synthesize it. For reusable gowns the silver could be reapplied at each set of 17 launderings. This means the attachment has to be applied more often in disposable gowns, which would lead to a higher environmental impact. Next to that, the sensitivity analysis shows reapplying the nAg every wash cycle for the reusable gown leads to a higher environmental impact for the reusable compared to the disposable gown, but after 28 cycles the impact of the reusable gown is lower compared to the disposable gown. When reapplying after every 17th cycle, the reusable gown has a lower impact compared to the disposable already at first use.   This study shows that disposable patient hospital gowns coated with nAg lead to a higher environmental impact for compared nAg coated reusable gowns. |
| Hogan et al. (2022) | 1. Climate Change The single-use cystoscope has a manufacturing carbon footprint of 1.34 kg CO2 per cystoscope (total weight is 158 g; 146.31 g plastic, 6.32 g steel, 2.84 g electronics, 2.53 g rubber). Waste to landfill produced 0.11 kg CO2, sterilisation 0.3 kg CO2, transport produced 0.049 kg CO2. Total median carbon footprint was 2.41 kg CO2 for the single-use cystoscope.   The manufacturing of a reusable cystoscope resulted in 14.94 kg CO2 per cystoscope. One cystoscopes performes roughly 1120 cystoscopies in a lifetime, which results in 0.013 kg per cystoscope. Waste disposed by incineration resulted in 0.52 kg CO2 and to landfill 0.22 kg CO2. Sterilisation results in 3.5 kg CO2 per case. The median carbon footprint was 4.23 kg CO2 per case.  2. Waste Median weight of waste was 622 g for the single-use cystoscope compared with 671.5 g for the reusable (p<0.0001). The single-use accounted for 25.4% of total waste and 27.3% when packaging was included. Waste for incineration was 496 g for single-use cystoscopy compared to 415 g for reusable. After reusable cystoscopy, more went to landfills, namely 256 g vs. 126 g after single-use cystoscopy. More waste was produced after a male cystoscopy (622.5 g) versus a femal cystoscopy (671.5 g).  3. Acidification No results in this study.   4. Eutrophication No results in this study.   5. Human Toxicity No results in this study.   6. Ecotoxicity No results in this study.   7. Ozone Depletion No results in this study. | The carbon footprint for reusable cystoscopes is higher compared to single-use. Median waste was also higher for the reusable cystoscope.  The gathering of the data seems to rely on different data sources, from different countries. Therefore, it seems it is not totally representable for the investigated country (Ireland). The sterilisation is based on Australian data, where a more coal based energy mix is used. This can lead to higher sterilisation numbers, and eventually the higher carbon footprint. Furthermore, it is unclear what is included in the waste calculations for both disposable and reusable options, leaving room for uncertainty about the actual amount of waste items. The study uses data and refers to the study from Davis et al. However, the authors from Davis et al. claim to have used 'standardized carbon footprint protocol guidelines,' but no specific reference to the protocol is provided. Consequently, it is unclear how the assessments were determined. As a result, the findings should be interpreted with caution. |
| Ibbotson et al. (2013) | 1. Climate Change Ibbotson (2013) reported the results on climate change graphically in Figure 4 of the article (Ibbotson, 2013). The figure shows the results on a log scale and the outcomes are extracted from this figure. It demonstrates that after 4,500 use cycles the disposable stainless steel scissor has the highest impact in this category (+/- 10,000 kg CO2-equivalents), followed by the disposable plastic scissor (+/- 5500 kg CO2-equivalents) and eventually the reusable stainless steel scissor (+/- 550 kg CO2-equivalents).  2. Waste No results in this study.   3. Acidification Ibbotson (2013) reported the results on acidification in Figure 4 of the article (Ibbotson, 2013). The figure shows the results on a log scale and the outcomes are extracted from this figure. It demonstrates that after 4,500 use cycles the disposable stainless steel scissor has the highest impact in this category (+/- 90 kg SO2-equivalents), followed by the disposable plastic scissor (+/- 20 kg CO2-equivalents) and eventually the reusable stainless steel scissor (+/- 0.8 kg SO2-equivalents).   4. Eutrophication Ibbotson (2013) reported the results on eutrophication in Figure 4 of the article (Ibbotson, 2013). The figure shows the results on a log scale and the outcomes are extracted from this figure. Freshwater and marine eutrophication are described separately. Regarding freshwater eutrophication, the results demonstrate that after 4,500 use cycles the disposable stainless steel scissor has the highest impact in this category (+/- 1 kg P-equivalents), followed by the disposable plastic scissor (+/- 0.55 kg P-equivalents) and eventually the reusable stainless steel scissor (+/- 0.3 kg P-equivalents). Next to that, with regard to marine eutrophication, the results demonstrate that after 4,500 use cycles the disposable stainless steel scissor has the highest impact in this category (+/- 10 kg N-equivalents), followed by the disposable plastic scissor (+/- 6 kg N-equivalents) and eventually the reusable stainless steel scissor (+/- 0.2 kg N-equivalents).  5. Human Toxicity Ibbotson (2013) reported the results on human toxicity in Figure 4 of the article (Ibbotson, 2013). The figure shows the results on a log scale and the outcomes are extracted from this figure. It demonstrates that after 4,500 use cycles the disposable stainless steel scissor has the highest impact in this category (+/- 7750 kg 1.4-DB equivalents), followed by the disposable plastic scissor (+/- 750 kg 1.4-DB equivalents) and eventually the reusable stainless steel scissor (+/- 200 kg 1.4-DB equivalents).   6. Ecotoxicity Ibbotson (2013) reported the results on ecotoxicity graphically in Figure 4 of the article (Ibbotson, 2013). The figure shows the results on a log scale and the outcomes are extracted from this figure. Terrestrial and freshwater ecotoxicity are described separately. Regarding terrestrial ecotoxicity, the results demonstrate that after 4,500 use cycles the disposable stainless steel scissor has the highest impact in this category (+/- 2 kg 1.4-DB equivalents), followed by the disposable plastic scissor (+/- 0.4 kg 1.4-DB equivalents) and eventually the reusable stainless steel scissor (+/- 0.03 kg 1.4-DB equivalents). Next to that, with regard to freshwater ecotoxicity, the results demonstrate that after 4,500 use cycles the disposable stainless steel scissor has the highest impact in this category (+/- 500 kg 1.4-DB equivalents), followed by the disposable plastic scissor (+/- 55 kg 1.4-DB equivalents) and eventually the reusable stainless steel scissor (+/- 4 kg 1.4-DB equivalents).  7. Ozone Depletion Ibbotson (2013) reported the results on ozone depletion in Figure 4 of the article (Ibbotson, 2013). The figure shows the results on a log scale and the outcomes are extracted from this figure. It demonstrates that after 4,500 use cycles the disposable stainless steel scissor has the highest impact in this category (0.00055 kg CFC-11 equivalents), followed by the disposable plastic scissor (0.0001 kg CFC-11 equivalents) and eventually the reusable stainless steel scissor (+/- 0.00004 kg CFC-11 equivalents). | The study shows that the reusable stainless steel scissor is the choice with the lowest environmental impact in all the impact categories investigated. This is followed by the disposable plastic scissor and eventually the disposable stainless steel scissor, which has the highest impact.   The hotspots for the disposable scissors were found in the material and manufacturing process and for the reusable scissor this was found in the usage phase, which could be appointed to the washing, disinfection and sterilisation cycles and the repair and service cycles. For stainless steel recycling, data from Australian data 2007 was used (which may not reflect the German setting). |
| Keil et al. (2022) *(see S2)* |  |  |
| Kemble et al. (2022) | 1. Climate Change Total estimated per case carbon footprint of SU cystoscopes was 2.40 kg CO2. For reusables, this was 0.53 kg CO2. The carbon footprint remains higher for SU regardless of case volume. When using another endoscope reprocessor for the reusable (EvoTech ECR endoscope reprocessor) the per-case carbon footprint resulted in 1.04kg CO2.  The single-use cystoscope has a manufacturing carbon footprint of 1.37 kg CO2 per cystoscope.Packaging was 0.22 kg CO2/case. Sterilisation has a carbon footprint of 0.3 kg CO2. Transportation was 0.2 kg CO2 per scope.  The manufacturing of a reusable cystoscope resulted in 6.55 kg CO2 per cystoscope. One cystoscope performes roughly 3920 cystoscopies in a lifetime, which results in 0.0017 kg per cystoscope. Sterilisation resulted in 0.20 kg CO2 per scope using the Medivators Advantage Plus Endoscope Reprocessing System, and 0.71 kg CO2 when using the ASP Evotech ECR. Repackaging was based on waste and calculated at <0.005 kg CO2 per case. Repair costs at 5 kg CO2/repair, the average per-case repair costs resulted in 0.024 kg CO2.   2. Waste Solid waste for SU scopes resulted in 0.31 kg CO2. Solid waste for the reusable cystoscope resulted in 0.000145 kg CO2.  3. Acidification No results in this study.   4. Eutrophication No results in this study.   5. Human Toxicity No results in this study.   6. Ecotoxicity No results in this study.   7. Ozone Depletion No results in this study. | The carbon footprint of reusable cystoscopes is lower compared to single-use (SU) cystoscopes. When using a different reprocessing system for reusable endoscopes, the carbon footprint increases but still remains lower than that of SU cystoscopes. This highlights the significant impact of the reprocessing system on overall emissions.  Importantly, the data used for calculating the CO₂ footprint is derived from previous studies, with limited transparency regarding how certain assessments were conducted. Differences in settings and assumptions are not fully accounted for, which introduces uncertainty. As a result, the findings should be interpreted with caution. |
| Le et al. (2022) | 1. Climate Change Performing an ERCP with a SD results in a carbon footprint between 36.3 and 71.5 kg CO2 eq., wich is 24 to 47 times more than with an RD (1.53 kg CO2 eq) and an RD with a disposable endcap (1.54 kg CO2 eq).   The biggest contributor for the SD is the manufacturing phase, wich accounts for 91 to 96% of the GHG emissions. The second highest contributor is disposal (1,8 kg CO2 eq, 3-5% of GHG emissions).  For the RD, the biggest contributor is the electricity use during the procedure (62%), followed by cleaning and disinfection of the RD (26%).   2. Waste No results in this study.   3. Acidification The normalised impact of performing an ERCP procedure on terrestrial acidification using an RD is 2.45E-06, using a RD with disposable endcap 2.43E-06, using a SD (lower bound) 4.40E-05, using a SD (upper bound) 8.84E-05.  4. Eutrophication The normalised impact of performing an ERCP procedure on freshwater eutrophication using an RD is 8.85E-07, using a RD with disposable endcap 8.86E-07, using a SD (lower bound) 4.28E-05, using a SD (upper bound) 9.01E-05.  The normalised impact of performing an ERCP procedure on marine eutrophication using an RD is 5.24E-10, using a RD with disposable endcap 5.25E-10, using a SD (lower bound) 4.32E-09, using a SD (upper bound) 8.72E-09.   5. Human Toxicity The normalised impact of performing an ERCP procedure on human non-carcinogenic toxicity using an RD is 2.44E-05, using a RD with disposable endcap 2.45E-05, using a SD (lower bound) 2.97E-03, using a SD (upper bound) 6.37E-03.  The normalised impact of performing an ERCP procedure on human carcinogenic toxicity using an RD is 1.23E-05, using a RD with disposable endcap 1.24E-05, using a SD (lower bound) 3.15E-04, using a SD (upper bound) 6.18E-04.   6. Ecotoxicity The normalised impact of performing an ERCP procedure on freshwater ecotoxicity using an RD is 1.51E-07, using a RD with disposable endcap 1.52E-07, using a SD (lower bound) 1.92E-05, using a SD (upper bound) 4.13E-05.  The normalised impact of performing an ERCP procedure on marine ecotoxicity using an RD is 2.96E-08, using a RD with disposable endcap 2.98E-08, using a SD (lower bound) 3.84E-06, using a SD (upper bound) 8.23E-06. The normalised impact of performing an ERCP procedure on terrestrial ecotoxicity using an RD is 4.57E-08, using a RD with disposable endcap 4.66E-08, using a SD (lower bound) 1.71E-06, using a SD (upper bound) 3.34E-06.  7. Ozone Depletion The normalised impact of performing an ERCP procedure on stratospheric ozone depletion using an RD is 1.26E-08, using a RD with disposable endcap 1.28E-08, using a SD (lower bound) 4.67E-07, using a SD (upper bound) 9.35E-07. | The carbon footprint of reusable duodenoscopes (with or without disposable endcap) is lower compared to single-use duodenoscopes. The greatest impact can be attributed to manufacturing for SD and to electricity use during the procedure for RD.   Across nearly all other impact categories, the reusable duodenoscope demonstrates the lowest environmental impact, making it the more favorable option overall. |
| Leiden et al. (2020) | 1. Climate Change Leiden (2020) reported the results in percentages. They are displayed as percentage of the maximum value of each impact category. For the outcome climate change, the reusable set had the highest impact (100%) compared to the disposable set (10-20%) after 1 surgery. For the disposable surgical set the production phase had the biggest contribution and for the reusable set the sterilization process.  2. Waste No results in this study.   3. Acidification Leiden (2020) reported the results in percentages. They are displayed as percentage of the maximum value of each impact category. For the outcome climate Acidification, the reusable set had the highest impact (100%) compared to the disposable set (30-40%) after 1 surgery. For the disposable surgical set the production phase had the biggest contribution and for the reusable set the sterilization process.   4. Eutrophication No results in this study.   5. Human Toxicity No results in this study.   6. Ecotoxicity No results in this study.   7. Ozone Depletion No results in this study. | The results show the reusable surgical set has a bigger environmental impact compared to the disposable set.   The disposable and reusable set are compared for 1 surgery. There has been a correction for the reusable set; only a three hundredth of the weight is taken into account for the production and disposal processes. The analysis would reflect the reality more if the functional unit would have been 300 surgeries.  It is important to take into account the content of the tray; the reusable set consists of 45.5 kg per set, and depending on the requirements for the surgery only a part of the set is applied. The disposable set is only 2.0 kg. Streamlining the reusable set could reduce the environmental impact of the reusable set.   Furthermore, sterilisation is conducted twice—both before and after the surgery. However, a better representation if a hospital owns the instruments, would involve including it only once. This dual inclusion could potentially have a negative impact on the results for the reusable tray. This has been included in the sensitivity analysis.   A sensitivity analysis has been conducted, wherein the reusable set has been reused multiple times, and there has been a shift from the loaner system to the hospital's consignment of the instruments. This results in a reduced need for disinfection and sterilisation, requiring only one-time sterilisation instead of the previous combination of sterilisation, cleaning, and disinfection. This leads to an approximate 50% reduction in ReCiPe endpoints for the reusable set. Furthermore, by further reducing the number of instruments, the overall impact could be further minimised.  The most significant hotspots are clearly identified. In the case of the reusable set, the sterilisation process emerges as the primary contributor to the environmental impact, while for the disposable set, it is the production process. |
| McGain et al. (2010) | 1. Climate Change The reusable tray produced 110 g of CO2 (95% CI 98 to 122 g CO2), the single use tray alone produced 126 g CO2 (95% CI 104 to 151 g) with a mean difference of 16 g CO2 (95% CI -8 to 40 g CO2). The single use tray with cotton and paper produced 203 g CO2 (95% CI 166 to 268 g CO2).  2. Waste No results in this study.   3. Acidification No results in this study.   4. Eutrophication No results in this study.   5. Human Toxicity No results in this study.   6. Ecotoxicity No results in this study.   7. Ozone Depletion No results in this study. | The CO2 production of single-use trays showed only a non-significant 15% increase. However, when modeling the single-use tray with cotton and paper, there was a notable rise in CO2 production.  In the case of the reusable tray, the washing process is the primary contributor to the total impact. Conversely, for the disposable tray, it is the production process of the polyurethane tray. Regarding cotton gauzes and paper towels, the production of the gauzes has the most significant impact.  It is important to note, that instead of the Chinese data (not possible to obtain), entirely European data was used. |
| McGain et al. (2012) | 1. Climate Change McGain (2012) described the results on climate change. One reusable kit produced 1211 grams of CO2 in total and one disposable kit 407 grams of CO2. The biggest contributor for the reusable kit is the washing and sterilisation process (256 resp. 830 grams of CO2), whereas for the single-use kit this is the plastic used (284 grams of CO2). A sensitivity analysis showed the influence of different energy mixes on the outcome for the reusable kit, with a Monte Carlo analysis to calculate confidence intervals (CI). Using a brown coal energy mix for the reusable kit resulted in 1211 (95% CI 1099-1323) grams of CO2 emissions, hospital gas cogeneration in 436 (95% CI 410-473) grams of CO2 emissions, United States electricity mix in 764 (95% CI 509-1174) grams of CO2 emissions and a European electricity mix in 572 (95% CI 470-713) grams of CO2 emissions.  2. Waste No results in this study.   3. Acidification No results in this study.   4. Eutrophication No results in this study.   5. Human Toxicity No results in this study.   6. Ecotoxicity No results in this study.   7. Ozone Depletion No results in this study. | The environmental and financial impacts of two type of central venous catheter insertion kits (single-use and disposable) are assessed. The results show the reusable kit has a bigger environmental impact compared to the disposable kit.   The biggest contributor for the reusable kit is the washing and sterilisation process, whereas for the single-use kit this is the use of plastic. The washing and sterilisation process could be a hotspot to minimalize the impact, as well as for the disposable kit a different source of material could be of great value. |
| McGain et al. (2017) | 1. Climate Change McGain (2017) described the five scenarios as following: (1) completely reusable, (2) mainly single-use except for reusable laryngoscope handles, (3) completely single-use (4) reusables (except the single-use face masks), (5) reusables (except single-use laryngoscope blades) in an Australian hospital. Using reusables (scenario 1) had a higher impact [5575 kg CO2 equivalents (95% CI 5542-5608)] compared to using mainly single use [scenario 2; 5095 kg CO2 equivalents (95% CI 4614-5658)]. For the reusable approach (4807 kg CO2 equivalents (86%)) was for washer electricity and 387 kg CO2 equivalents (7%) for H2O2 sterilizer electricity, with all other contributing for 381 kg CO2 equivalents (7%). For scenario 2 (mainly single-use), the majority of the CO2 emissions (2695 kg CO2 equivalents, 52%) was for purchasing single use face masks (n=9900) and 1396 kg CO2 equivalents (27%) for the single-use direct laryngoscope blades (n=9900) and all other items contributed for 1052 kg CO2 equivalents (21%). Scenario 3 resulted in 5775 kg CO2 equivalents. Scenarios 4 and 5 led to 6556 and 6763 kg CO2 equivalents emissions respectively, because 365 and 550 washer loads, respectively, remained. The substitution of one reusable with a single-use item (Scenarios 4 and 5) led to higher CO2 emissions than either completely reusable or single-use equipment (Scenarios 1–3).  An analysis was performed to model results as if the hospital was based in UK/Europe. This led to different results compared to when the hospital was based in Australia. By switching from single-use (5095 kg CO2 equivalents) to reusable anaesthetic equipment, this would have led in a decrease of 84% (802 kg CO2 equivalents). This can be explained by the majority of the next kilowatt hour of UK/European electricity generation arising from renewables (mainly wind).   2. Waste Using reusables (scenario 1) resulted in less waste (250 kg) compared to using mainly single use (scenario 2; 1222 kg of waste). Scenario 3 had the highest amount of waste (1542 kg) and scenarios 4 and 5 led to 375 and 917 kg of waste, respectively.  3. Acidification No results in this study.   4. Eutrophication These outcomes resulted all in a low impact on eutrophication. Using reusables (scenario 1) resulted in 0.000 kg P equivalents whereas using mainly single use (scenario 2) led to 0.12 kg P equivalents. Scenario 3, 4 and 5 led to 0.12, 0.04 and 0.07 kg P equivalents, respectively.  5. Human Toxicity Using reusables (scenario 1) resulted in 12 kg 1.4-DB equivalents whereas scenario 2 resulted in the highest impact of all scenarios (713 kg 1.4-DB equivalents). Scenario 3, 4 and 5 led to 1.023, 195 and 491 kg 1.4-DB equivalents, respectively.  6. Ecotoxicity The outcome ecotoxicity was divided in three different outcomes: terrestrial, freshwater and marine ecotoxicity. For terrestrial ecotoxicity, using reusables (scenario 1) resulted in 0.011 kg 1.4-DB equivalents whereas scenario 2 resulted in 0.4 kg 1.4-DB equivalents. Scenario 3, 4 and 5 led to 0.405, 0.118 and 0.2 kg 1.4-DB equivalents, respectively. For freshwater ecotoxicity, using reusables (scenario 1) resulted in 0.7 kg 1.4-DB equivalents whereas scenario 2 resulted in 91 kg 1.4-DB equivalents. Scenario 3, 4 and 5 led to 93.4, 3.1 and 88 kg 1.4-DB equivalents, respectively. For marine ecotoxicity, using reusables (scenario 1) resulted in 0.7 kg 1.4-DB equivalents whereas scenario 2 resulted in 94.5 kg 1.4-DB equivalents. Scenario 3, 4 and 5 led to 97.2, 2.8 and 92.3 kg 1.4-DB equivalents, respectively. Moreover, using single-use equipment (scenario 2 and 3) has the highest impact on ecotoxicity.   7. Ozone Depletion No results in this study. | The results of this study result in a clear overview on how environmental impacts of the same type of equipment (e.g. reusable) can vary between different continents. Where the single-use equipment seem to have a lower environmental impact in Australia, the results suggest the impact is lower in the USA, UK and in Europe. This is due to the energy mix used in the different continents.   In Australia the impact of single-use equipment is lower compared to the other continents, where it is beneficial for the environment to use the reusable anaesthetic equipment. |
| McPherson et al. (2019) | 1. Climate Change Annual greenhouse gas (GHG) emissions resulted in climate impacts of 248.62 metric tons of (MT) CO2 equivalents for DSC and 86.19 MTCO2 equivalents for reusable sharps containers (RSC). Adjusted patient days (APD) were used as the workload indicator to which results were normalized. This resulted in 8.37 MTCO2 equivalents per 10,000 APD for DSC and 2.90 MTCO2 equivalents per 10,000 APD for RSC. Use of RSC reduces GWP by 162.4 MTCO2eq (65.3%, P<0.001, RR 2.27-3.71).  2. Waste Annual waste for DSC resulted in 31.8 tonnes of landfilled plastic, 18.8 tonnes of incinerated plastic and 8.2 tonnes of cardboard boxes for 48,460 manufactured and 35,925 landfilled DSC (chemotherapy DSC were incinerated).  Whereas RSC only caused 0.4 tonnes of plastic waste (Tonnes of chemo/pharma DSC incinerated; 412 chemo DSC were used during RSC year) and 0.1 kg of waste from carboard boxes (this were the chemotherapy DSC, which were used in both systems if there was an indication for chemotherapy). In total 3195 RSC were manufactured and 0 containers were landfilled (all parts were either reused or recycled).   3. Acidification No results in this study.   4. Eutrophication No results in this study.   5. Human Toxicity No results in this study.   6. Ecotoxicity No results in this study.   7. Ozone Depletion No results in this study. | Use of RSC leads to reduction of climate impacts and waste.  The manufacturing process is the biggest contributor in climate impacts for DSC, and thereby gives the largest difference between the two systems. It is predominantly a function of the energy required for the higher total polymer weight needed to be annually manufactured and molded for DSC.   Transport is the biggest contributor for RSC. Although more DSC required transportation, the daily transport of RSC resulted in similar GHG over the year between RSC and DSC.  The sensitivity analysis revealed variations in RSC lifespan contributed little to the GHG result. It showed that differing electricity sources can alter the GHG contribution of the manufacturing process. It can alter DSC GHG by 23% and RSC GHG by 10%. RSC reprocessing accounted for 5.6% of the RSC life cycle. Material reclamation could reduce DSC life cycle GHG. |
| Morris et al. (2022) | 1. Climate Change Breakeven analysis: The acrylic speculum (incinerated) surpasses the impact global warming compared to the reusable at the 3rd use. If landfilled or recycled, after the 4th use.   2. Waste No results in this study.  3. Acidification Breakeven analysis: The acrylic speculums breakeven with the stainless-steel systems in the 4th use.  4. Eutrophication Breakeven analysis: The acrylic speculums breakeven with the stainless-steel systems in the 17th, 18th and 19th use.  5. Human Toxicity Breakeven analysis: Non-carcinogenics: The acrylic speculums breakeven with the stainless-steel systems in the 44-47th use.  Breakeven analysis: Carcinogenics: The acrylic speculums breakeven with the stainless-steel systems in the 59-61th use.  6. Ecotoxicity Breakeven analysis: The acrylic speculums breakeven with the stainless-steel systems in the 40-42nd use.  7. Ozone Depletion Breakeven analysis: Stainless-steel reusable speculms have more impact regarding ozone depletion compared to the disposable speculum. This is attributable to the use phase, mainly through production if nitril gloves. | For almost all impact categories, in the breakeven analysis the reusable systems eventually have a lower impact compared to the disposable systems, except for ozone depletion. This is mainly due to the nitrile glove use in the use phase. This use phase contributes most for the reusable. Where the reusable was kept in the circulation for shorter time periods (1 year or 3 year scenario), the raw materials and manufacturing contributed more to certain impacts. Therefore, it is preferable to have a long lifetime for the reusable speculum.  In the use phase of the reusable speculum multiple products are used (such as gloves, gowns or nonionic surfactant) which also are remarkable contributors, next to electricity use for the autoclaves (mainly from electricity produced from coal), which is most dominant in this contribution analysis.  The acrylic speculum recycling scenarios had a lesser impact compared to incineration or landfill for all impact categories, therefore this is preferred in the disposal phase.   Stainless steel production is the most sensitive input for reusable speculums on different impact categories. Tapwater, if increased, had the least impact. For the acrylic speculum the recycling of the speculum itself is the most sensitive input. |
| Namburar et al. (2021) | 1. Climate Change No results in this study.   2. Waste The annual waste produced during endoscopic procedures in the US for the three different scenarios show that the ‘all reusable’ endoscopes (scenario 1) produce the least amount of waste (43,500 metric tons of waste for 18 million endoscopies annually in the US), followed by using single-use colonoscopes/duodenoscopes (scenario 2; 54,375 metric tons of waste) and all single-use endoscopes (scenario 3; 60,900 metric tons of waste).   3. Acidification No results in this study.   4. Eutrophication No results in this study.   5. Human Toxicity No results in this study.   6. Ecotoxicity No results in this study.   7. Ozone Depletion No results in this study. | The study suggests the least amount of waste is produced by using ‘all reusable’ endoscopes. When only focusing on waste, this should be the best option following the three given scenarios. However there are no further calculations regarding environmental impact. With these calculations, as the authors suggest in the discussion, this would give a better overview of the environmental impact of the procedures, taking the whole life cycle into of the endoscopes (and procedures) into account. |
| Rizan et al. (2021) | 1. Climate Change The carbon footprint of the hybrid laparoscopic instruments is lower compared to the single-use instruments. Compared to its single-use equivalent, the hybrid clip applier’s carbon footprint was 17% (445 g vs 2559 g CO2 eq), the scissor 33% (378g vs 1139 g CO2 eq) and the four ports 27% (933 g vs 3495 CO2 eq). All combined, the carbon footprint of using all hybrid instruments was 24% of that of single-use equivalents (1756 g vs 7194 g CO2 eq), saving 5.4 kg CO2 eq. The majority of the carbon footprint of the hybrid instruments was due to single-use components (mean 62%, range 43-79%), followed by decontamination of reusable components (mean 37%, range 21-56%). For the single-use instruments the biggest hotspots were raw material extraction and manufacturing (mean 57%, range 52-61%), followed by onward transportation (mean 29%, range 24-36%) and waste (mean 14%, range 12-16%). The scenario modelling resulted in the following results. When packaging and decontaminating separately, the CO2 footprint of the hybrid clip applier increased 3.7-fold to 1650 g CO2 eq. The scissor increases to 394 g CO2 eq per use (4% increase) and the ports 999 g CO2 eq per use (7% increase). For all hybrid instruments, CO2 footprint was lower than the single-use equivalents when used more than twice. The CO2 footprint of the decontamination process of hybrid instruments increased with 54% when using Australian electricity, which increased the CO2 footprint of the hybrid instruments by 11-30%, but this remained lower than the single-use equivalents (63-77%). Shipping in place of airfreight (for single-use items) reduced the CO2 footprint by 22-33%. Using three hybrid 5 mm ports and one 10 mm port (635 g CO2 eq/operation) resulted in a 32% reduction compared to the base case (5 mm single-use ports based on a dual pack).  2. Waste No results in this study.   3. Acidification Rizan (2021) reported the results of the three different instruments. The ports had the highest impact in this category (single-use vs. hybrid, 8.91 vs. 2.08 g SO2 eq), followed by the laparoscopic clip applier (single-use vs. hybrid, 8.53 vs. 1.18 g SO2 eq) and the laparoscopic scissors (single-use vs. hybrid, 4.46 vs. 1.44 g SO2 eq).   4. Eutrophication Rizan (2021) reported the results of the three different instruments on eutrophication divided in two categories: freshwater and marine eutrophication. The laparoscopic clip applier had the highest impact in the category “freshwater eutrophication” (single-use vs. hybrid, 0.62 vs. 0.12 g SO2 eq), followed by the ports (single-use vs. hybrid, 0.43 vs. 0.17 g SO2 eq) and the laparoscopic scissors (single-use vs. hybrid, 0.26 vs. 0.17 g SO2 eq). For the category “marine eutrophication” this resulted in the highest impact for the ports (single-use vs. hybrid, 0.12 vs. 0.07 g SO2 eq), followed by the laparoscopic clip applier (single-use vs. hybrid, 0.09 vs. 0.06 g SO2 eq) and the laparoscopic scissors (single-use vs. hybrid, 0.05 vs. 0.04 g SO2 eq).  5. Human Toxicity Rizan (2021) reported the results of the three different instruments on human toxicity divided in two categories: carcinogenic and non-carcinogenic human toxicity. Overall, the hybrid instruments have a lower environmental impact in this category. The laparoscopic clip applier had the highest impact in the category “carcinogenic human toxicity” (single-use vs. hybrid, 203 vs. 45 g 1.4-DCB eq), followed by the ports (single-use vs. hybrid, 117 vs. 43 g 1.4-DCB eq) and the laparoscopic scissors (single-use vs. hybrid, 91 vs. 65 g 1.4-DCB eq). Although, the hybrid port has a higher impact than the hybrid laparoscopic scissor. For the category “noncarcinogenic human toxicity” the results were as following (from greatest environmental impact to lowest impact): Single-use laparoscopic clip applier (2871 g 1.4-DCB eq), single-use laparoscopic scissor (1386 g 1.4-DCB eq), single-use ports (1013 g 1.4-DCB eq), hybrid laparoscopic scissor (952 g 1.4-DCB eq), hybrid laparoscopic clip applier (576 g 1.4-DCB eq) and hybrid ports (390 g 1.4-DCB eq).   6. Ecotoxicity Rizan (2021) reported the results of the three different instruments on ecotoxicity divided in three categories: “terrestrial”, “freshwater” and “marine” ecotoxicity. Overall, the hybrid instruments have a lower environmental impact in this category, except for the laparoscopic scissors in freshwater and marine ecotoxicity. For terrestrial ecotoxicity, the results were as following (from greatest environmental impact to lowest impact): Single-use laparoscopic clip applier (19,767 g 1.4-DCB eq), single-use laparoscopic scissor (8939 g 1.4-DCB eq), hybrid laparoscopic scissor (5628 g 1.4-DCB eq), single-use ports (4142 g 1.4-DCB eq), hybrid laparoscopic clip applier (3976 g 1.4-DCB eq) and hybrid ports (1171 g 1.4-DCB eq). For freshwater ecotoxicity, the results were as following (from greatest environmental impact to lowest impact): Single-use laparoscopic clip applier (176 g 1.4-DCB eq), hybrid laparoscopic scissor (97 g 1.4-DCB eq), single-use laparoscopic scissor (91 g 1.4-DCB eq), single-use ports (39 g 1.4-DCB eq), hybrid laparoscopic clip applier (36 g 1.4-DCB eq) and hybrid ports (17 g 1.4-DCB eq). For marine ecotoxicity, the results were as following (from greatest environmental impact to lowest impact): Single-use laparoscopic clip applier (230 g 1.4-DCB eq), hybrid laparoscopic scissor (122 g 1.4-DCB eq), single-use laparoscopic scissor (118 g 1.4-DCB eq), single-use ports (54 g 1.4-DCB eq), hybrid laparoscopic clip applier (47 g 1.4-DCB eq) and hybrid ports (23 g 1.4-DCB eq)..   7. Ozone Depletion Rizan (2021) reported the results of the three different instruments on ozone depletion as following (from greatest environmental impact to lowest impact): Single-use ports (0.0013 g CFC11 eq), single-use laparoscopic clip applier (0.0008 g CFC11 eq), single-use laparoscopic scissor (0.0005 g CFC11 eq), hybrid ports (0.0004 g CFC11 eq), hybrid laparoscopic clip applier (0.0002 g CFC11 eq) and hybrid laparoscopic scissor (0.0001 g CFC11 eq). | The CO2 footprint of using hybrid scissors, ports and clip appliers was 76% lower than using single-use equivalents, saving 5.4 kg CO2eq per operation. Overall, the environmental impact of the hybrid instruments are lower compared to the single-use instruments. This is mainly due to the manufacturing and raw material extraction process. |
| Sanchez et al. (2020) | 1. Climate Change Sanchez (2020) reported outcomes using 4 different scenarios: (1) Day office, (2) 1 Day Ambulatory Procedure, (3) 1 Day Regular Ward and (4) 1 Day ICU. Within these scenarios, a division was made between: reusable incineration (1 cleaning/encounter or 1 cleaning/day), reusable landfill (1 cleaning/encounter or 1 cleaning/day), disposable incineration (1 cleaning/encounter or 1 cleaning/day), disposable landfill (1 cleaning/encounter or 1 cleaning/day). The results of these different scenarios are summarized in the supplemental material of the study (Sanchez, 2020). For the outcome measure climate change, the overall results show reusable blood pressure cuffs have a lesser environmental impact compared to the disposable variant. The biggest contributor for the disposable is the material and manufacturing process, whereas for the reusable blood pressure cuff the main contributor is the production of the chemical wipes (which are used for cleaning).   2. Waste No results in this study.   3. Acidification The disposable blood pressure cuffs have a higher environmental impact considering acidification compared to the reusable variant. For the disposable cuff, this is especially related to the manufacturing process. For the reusable variant the biggest contributor is the production of the cleaning wipes.   4. Eutrophication The disposable blood pressure cuffs have a higher environmental impact considering eutrophication compared to the reusable variant. For the disposable cuff, this is especially related to the manufacturing process and the disposal of the cuffs. For the reusable variant the biggest contributor is the production of the cleaning wipes and partly the disposal of these wipes.  5. Human Toxicity The disposable blood pressure cuffs have a higher environmental impact considering human toxicity (non-carcinogens and carcinogens) compared to the reusable variant. For the disposable cuff, this is especially related to the manufacturing process and the disposal of the cuffs. For the reusable variant this is mainly due to the production of the cleaning wipes.  6. Ecotoxicity The disposable blood pressure cuffs have a higher environmental impact considering ecotoxicity compared to the reusable variant. For the disposable cuff, this is especially related to the manufacturing process and the disposal of the cuffs. For the reusable variant this is mainly due to the production of the cleaning wipes.  7. Ozone Depletion The disposable blood pressure cuffs have a higher environmental impact considering ozone depletion compared to the reusable variant. This is especially related to the manufacturing process. | The overall results show the reusable blood pressure cuff has a lower environmental impact on all impact categories compared to the disposable cuff. The main contributors for the disposable cuff are the production process and the disposal. For the reusable cuff this is mainly due to the production process of the cleaning wipes. However, the environmental impact of the reusable blood pressure cuff remains lower compared to the disposable. |
| Sherman et al. (2018) | 1. Climate Change Sherman (2018) reported outcomes on climate change on both laryngoscope handles and blades (reusable or single-use) as well as on different cleaning scenarios (low-level disinfection levels (LDL), high-level disinfection levels (HDL) and sterilisation). The most favourable scenario for the handles is the reusable stainless steel handle, treated to HDL. Choosing LDL will result in a 40% increase of the CO2 footprint (0.08 kg CO2 eq per use). Sterilisation will lead to a 400% increase (0.23 kg CO2 eq per use). The single-use handle has a 25 times bigger CO2 footprint compared to the reusable version (1.41 kg CO2 eq and 1.60 kg CO2 eq for the plastic and metal handles, respectively). The most favorable scenario for the blades is the reusable steel tongue blade treated to (the minimum) HDL standards. Sterilisation will lead to a 400% increase (0.22 kg CO2 eq per use) compared to HDL (0.06 kg CO2 eq per use). Single-use options for the blades will result in an 6-8 times increase of CO2 footprint (0.38 kg CO2 eq and 0.44 kg CO2 eq for the plastic and metal blades, respectively).   2. Waste No results in this study.   3. Acidification Sherman (2018) reported outcomes on acidification on both laryngoscope handles and blades (reusable or single-use) as well as on different cleaning scenarios (low-level disinfection levels (LDL), high-level disinfection levels (HDL) and sterilisation). The most favorable scenario for the handles is the reusable stainless steel handle, treated to HDL. Choosing LDL will result in a 70% increase of the CO2 footprint. Sterilisation will lead to a 200% increase. The single-use handle has a 33 times bigger CO2 footprint compared to the reusable version. The most favourable scenario for the blades is the reusable steel tongue blade treated to (the minimum) HDL standards. Sterilisation will lead to a 350% increase compared to HDL. Single-use options for the blades will result in an 5-10 times increase of CO2 footprint.  4. Eutrophication Sherman (2018) reported outcomes on eutrophication on both laryngoscope handles and blades (reusable or single-use) as well as on different cleaning scenarios (low-level disinfection levels (LDL), high-level disinfection levels (HDL) and sterilisation). The most favourable scenario for the handles is the reusable stainless steel handle, treated to HDL. Choosing LDL will result in a 160% increase of the CO2 footprint. Sterilisation will lead to a 100% increase. The single-use handle has a 65 times bigger CO2 footprint compared to the reusable version. The most favourable scenario for the blades is the reusable steel tongue blade treated to (the minimum) HDL standards. Sterilisation will lead to a 150% increase compared to HDL. Single-use options for the blades will result in an 8-15 times increase of CO2 footprint.  5. Human Toxicity Sherman (2018) reported outcomes on human toxicity on both laryngoscope handles and blades (reusable or single-use) as well as on different cleaning scenarios (low-level disinfection levels (LDL), high-level disinfection levels (HDL) and sterilisation). This outcome is divided in carcinogenics as well as noncarcinogenics. For the carcinogenics, the most favourable scenario for the handles is the reusable stainless steel handle, treated to HDL. Choosing LDL will result in a 200% increase of the CO2 footprint. Sterilisation will lead to a 150% increase. The single-use handle has a 45 (plastic) and 250 (steel) times bigger CO2 footprint compared to the reusable version. The most favorable scenario for the blades is the reusable steel tongue blade treated to (the minimum) HDL standards. Sterilisation will lead to a 150% increase compared to HDL. Single-use options for the blades will result in an 7-160 times increase of CO2 footprint. For the noncarcinogenics, the most favorable scenario for the handles is the reusable stainless steel handle, treated to HDL. Choosing LDL will result in a 100% increase of the CO2 footprint. Sterilisation will lead to a 150% increase. The single-use handle has a 135 (plastic) and 180 (steel) times bigger CO2 footprint compared to the reusable version. The most favourable scenario for the blades is the reusable steel tongue blade treated to (the minimum) HDL standards. Sterilisation will lead to a 200% increase compared to HDL. Single-use options for the blades will result in an 10-42 times increase of CO2 footprint.  6. Ecotoxicity Sherman (2018) reported outcomes on ecotoxicity on both laryngoscope handles and blades (reusable or single-use) as well as on different cleaning scenarios (low-level disinfection levels (LDL), high-level disinfection levels (HDL) and sterilization). This outcome is divided in carcinogenics as well as noncarcinogenics. For the carcinogenics, the most favourable scenario for the handles is the reusable stainless steel handle, treated to HDL. Choosing LDL will result in a 400% increase of the CO2 footprint. Sterilisation will lead to a 100% increase. The single-use handle has a 130 (plastic) and 225 (steel) times bigger CO2 footprint compared to the reusable version. The most favourable scenario for the blades is the reusable steel tongue blade treated to (the minimum) HDL standards. Sterilisation will lead to a 150% increase compared to HDL. Single-use options for the blades will result in an 13-95 times increase of CO2 footprint.  7. Ozone Depletion Sherman (2018) reported outcomes on ozone depletion on both laryngoscope handles and blades (reusable or single-use) as well as on different cleaning scenarios (low-level disinfection levels (LDL), high-level disinfection levels (HDL) and sterilisation). This outcome is divided in carcinogenics as well as noncarcinogenics. For the carcinogenics, the most favourable scenario for the handles is the reusable stainless steel handle, treated to HDL. Choosing LDL will result in a 3000% increase of the CO2 footprint. Sterilisation will lead to a 200% increase. The single-use handle has a 17 times bigger CO2 footprint compared to the reusable version. The most favourable scenario for the blades is the reusable steel tongue blade treated to (the minimum) HDL standards. Sterilisation will lead to a 300% increase compared to HDL. Single-use options for the blades will result in an 3-7 times increase of CO2 footprint. | The environmental impact of the reusable stainless steel laryngoscope blades and handles is lowest. The greater impact of the disposable variants is due to the material manufacturing and device assembly. The reusables create emissions mainly from reprocessing and are thus reliable on the source of cleaning. |
| Sorensen et al. (2022) | 1. Climate Change This study reported a difference in the outcome climate change between the tube combined with reusable scope (waste incinerated) compared to the single-use tube (waste incinerated). The tube combined with the reusable scope has the highest impact (100% - 2.1 kg CO2 eq.) in the outcome climate change compared to the single-use tube (59.5% - 1.25 kg CO2 eq). The biggest contributors were: the amount of material used, the incineration of packagages and PET, the material and energy use needed for the washing and handling during the cleaning of scopes.  In the scenario analysis the effect on the carbon footprint with different recycling scenarios is included. The tube with reusable scope can be reduced to 0.6 kg CO2 eq, and the single-use to 0.3 kg CO2 with 100% recycling of paper and plastics.  2. Waste No results in this study.   3. Acidification This study reported a difference in acidification between the tube combined with reusable scope (waste incinerated) compared to the single-use tube (waste incinerated). The tube combined with the reusable scope has the highest impact (100%- read from graph) compared to the single-use tube (40% - read from graph).  4. Eutrophication No results in this study.  5. Human Toxicity No results in this study.  6. Ecotoxicity No results in this study.  7. Ozone Depletion This study reported a difference in ozone depletion between the tube combined with reusable scope (waste incinerated) compared to the single-use tube (waste incinerated). The tube combined with the reusable scope has the lowest impact (+/-1% - read from graph ) compared to the single-use tube (100% - read from graph). | The carbon footprint of the compared tubes is lower for the single-use tube with integrated camera (1.25 vs. 2.1 kg CO2 eq) compared with a single-use double lumen tube (DLT) combined with a reusable bronchoscope, and can be further reduced when recycling paper and plastics (0.3 vs. 0.6 kg CO2 eq). However, it is important to note that for the DLT with reusable bronchoscope, the reusable bronchoscope is included in the analysis, but for the DLT with single-use integrated camera, the reusable monitor is not included in the analysis. For other outcomes than climate impacts, only percentages are shown. The different impact categories shift in highest impact between the compared tubes. As stated in the paper, in the scenario analysis a reduction of the carbon footprint can be reached with 100% recycling of paper and plastics, however this is practically a challenge to reach this 100%. However, recycling of the materials leads to a reduced carbon footprint and should be considered instead of waste incineration. The environmental hotspots that can be tackled are: the amount of material used, the incineration of packages and PET, the material and energy use needed for the washing and handling during the cleaning of scopes. The reduction in environmental impact can be made by focussing on these contributors. |
| Unger et al. (2014) | 1. Climate Change This study reported a difference in the outcome climate change between the disposable and reusable dental burs. When the autoclave and ultrasonic were fully loaded (best case scenario - 30 dental burs) the reusable shows a lower environmental impact in the category global warming (1.19 kg CO2 eq versus 0.42 kg CO2 eq.)  The glass fiber reinforced plastic used in pakcaging was a significant contributor.  Outcomes mid-case scenario (loading capacity 66% - 20 dental burs): normalised disposable 1.0 versus reusable 0.99 - read from graph Outcomes worst-case scenario (loading capacity 33% - 10 dental burs): normalised disposable 0.96 versus reusable 1.0 - read from graph  2. Waste No results in this study.   3. Acidification This study reported a difference in the outcome acidification between the disposable and reusable dental burs. When the autoclave and ultrasonic were fully loaded (best case scenario - 30 dental burs) the reusable shows a lower environmental impact in the category acidification (normalised disposable 1.0 versus reusable 0.48 - read from graph).  Outcomes mid-case scenario (loading capacity 66% - 20 dental burs): normalised disposable 0.75 versus reusable 1.0 - read from graph Outcomes worst-case scenario (loading capacity 33% - 10 dental burs): normalised disposable 0.45 versus reusable 1.0 - read from graph  4. Eutrophication This study reported a difference in the outcome eutrophication between the disposable and reusable dental burs. When the autoclave and ultrasonic were fully loaded (best case scenario - 30 dental burs) the reusable shows a lower environmental impact in this category (normalised disposable 1.0 versus reusable 0.6 - read from graph).  The glass fiber reinforced plastic used in pakcaging was a significant contributor.  Outcomes mid-case scenario (loading capacity 66% - 20 dental burs): normalised disposable 0.58 versus reusable 1.0 - read from graph Outcomes worst-case scenario (loading capacity 33% - 10 dental burs): normalised disposable 0.34 versus reusable 1.0 - read from graph  5. Human Toxicity This study reported a difference in the outcome non-carcinogenics between the disposable and reusable dental burs. When the autoclave and ultrasonic were fully loaded (best case scenario - 30 dental burs) the reusable shows a lower environmental impact in this category (normalised disposable 1.0 versus reusable 0.51 - read from graph).  This study reported a difference in the outcome carcinogenics between the disposable and reusable dental burs. When the autoclave and ultrasonic were fully loaded (best case scenario - 30 dental burs) the reusable shows a lower environmental impact in this category (normalised disposable 1.0 versus reusable 0.44 - read from graph).  Outcomes mid-case scenario non-carcinogenics (loading capacity 66% - 20 dental burs): normalised disposable 0.68 versus reusable 1.0 - read from graph Outcomes worst-case scenario non-carcinogenics (loading capacity 33% - 10 dental burs): normalised disposable 0.42 versus reusable 1.0 - read from graph  Outcomes mid-case scenario carcinogenics (loading capacity 66% - 20 dental burs): normalised disposable 0.8 versus reusable 1.0 - read from graph Outcomes worst-case scenario carcinogenics (loading capacity 33% - 10 dental burs): normalised disposable 0.48 versus reusable 1.0 - read from graph  6. Ecotoxicity This study reported a difference in the outcome ecotoxicity between the disposable and reusable dental burs. When the autoclave and ultrasonic were fully loaded (best case scenario - 30 dental burs) the reusable shows a lower environmental impact in this category (normalised disposable 1.0 versus reusable 0.25 - read from graph).  The glass fiber reinforced plastic used in pakcaging was a significant contributor.  Outcomes mid-case scenario (loading capacity 66% - 20 dental burs): normalised disposable 1.0 versus reusable 0.69 - read from graph Outcomes worst-case scenario (loading capacity 33% - 10 dental burs): normalised disposable 0.88 versus reusable 1.0 - read from graph  7. Ozone Depletion This study reported a difference in the outcome ozone depletion between the disposable and reusable dental burs. When the autoclave and ultrasonic were fully loaded (best case scenario - 30 dental burs) the reusable shows a lower environmental impact in this category (normalised disposable 1.0 versus reusable 0.15 - read from graph).  The glass fiber reinforced plastic used in pakcaging was a significant contributor.  Outcomes mid-case scenario (loading capacity 66% - 20 dental burs): normalised disposable 1.0 versus reusable 0.41 - read from graph Outcomes worst-case scenario (loading capacity 33% - 10 dental burs): normalised disposable 1.0 versus reusable 0.67 - read from graph | In the best case scenario, the reusable dental bur is a better choice considering environmental impacts. However, when the loading efficiency decreases, one should be aware that the impacts can change towards a more positive scenario for the disposable dental burs. It shows that this is an important factor in decreasing the environmental impact. Furthermore, packaging of the dental burs was an important contributor in all impact categories, and therefore possibly an area of improvement. |
| Vozzola et al. (2018) | 1. Climate change The CO2 footprint of reusable isolation gowns resulted in 218 kg CO2 equivalents for 1000 uses. The disposable resulted in 310 kg CO2 equivalents per 1000 uses.   Switching to reusable resulted in a 30% decrease of the carbon footprint.  For the disposable isolation gowns the manufacturing process contributed most to the carbon footprint (accounting for 97% of the energy consumption and climate impacts and 100% of the blue water consumption).   The laundry steps had a large influence on the environmental indicators for reusable isolation gowns, accounting for 68% of energy consumption, 67% of greenhouse gas emissions, and 20% of blue water consumption. Nevertheless, the reduction in environmental impact achieved by producing fewer gowns (when using reusables) outweighed the added load imposed by the laundering process of reusables.  2. Waste The amount of solid waste of reusable isolation gowns resulted in 0.413-4.42 kg for 1000 uses (range based on 0-100% reuse in other industries after disposal). The disposable resulted in 63.4 kg per 1000 uses.   Switching to reusable resulted in at least a 93% decrease of solid waste.  3. Acidification No results in this study.   4. Eutrophication No results in this study.   5. Human Toxicity No results in this study.   6. Ecotoxicity No results in this study.   7. Ozone Depletion No results in this study. | The reusable isolation gowns have a lower environmental impact and produce less waste compared to the disposable variant.  The biggest contributor in CO2 footprint for the disposable coverall is the manufacturing process. For the reusable variant this is the laundry process. The weight of the disposable gown is important, when decreasing the weight, the environmental impact would also decrease drastically. For reusables, this was less significant. For the reusable, the energy-efficiency in the laundry process is an important factor in decreasing the impact. |
| Vozzola et al. (2020) | 1. Climate Change The total GWP for 1,000 uses of the reusable surgical gown is 557 kg CO2eq, and for the disposable 1636 kg CO2eq. By selecting the reusable surgical gown, this will result in a 66% reduction of GWP.   The gown manufacturing and supply chain resulted for 1,000 uses of the reusable gown in 134 kg CO2eq and for the disposable gown 1495 kg CO2eq.   The packaging manufacturing and supply chain resulted for 1,000 uses of the reusable gown in 76.7 kg CO2eq and for the disposable gown in 121 kg CO2eq.   Laundry resulted in 278 kg CO2eq for the reusable gown, and there was 0 kg CO2eq used for the disposable gowns.   The sterilisation of the gowns resulted in 19.8 kg CO2eq for the reusable and 6.26 kg CO2eq for the disposable gown.  The use phase transport of 1,000 reusable gowns resulted in 38.7 kg CO2eq for the reusable gown and 2.47 kg CO2eq for the disposable gown.   The end of life contribution to the GWP resulted in 1.40 kg CO2eq for the reusable variant and 10.9 kg CO2eq for the disposables.   2. Waste Solid waste per 1,000 uses/1,000 gowns resulted in 35.5-43.4 kg for the reusable and 265 kg for the disposable gown.  Gown manufacturing resulted in 0-7.9 kg solid waste for the reusable and 224 kg solid waste for the disposable gown (1,000 uses/gowns).   Packaging manufacturing and supply chain yielded 35.5 kg solid waste for the reusable gown and 40.3 kg for the disposable (1,000 uses/gowns).   End of life resulted in 0-0.00842 kg solid waste for the reusable and 0.505 for disposable gowns (1,000 uses/gowns).   3. Acidification No results in this study.   4. Eutrophication No results in this study.   5. Human Toxicity No results in this study.   6. Ecotoxicity No results in this study.   7. Ozone Depletion No results in this study. | The reusable surgical gown has lesser impact on the environment in terms of Climate Change and waste.   The biggest contributor for the disposable gown is the manufacturing process, as well for the GWP as in waste production.   For the reusable surgical gown the laundry phase has the greatest impact. |
| ***Topic 3 "Cover materials"*** |  |  |
| Nowack et al. (2012) *(see S2)* |  |  |
| ***Topic 4 "Anaesthetics"*** |  |  |
| Hu et al. (2021) | 1. Climate Change The results on climate change are graphically shown in figure 2-4 (Hu, 2021). Three different scenarios are studied:  1. Fresh gas flow of 1L (UK) or 2L (US)/min, % gas flow O2/N2O = 40/60 2. Fresh gas flow of 1L (UK) or 2L (US)/min, % gas flow O2/N2O = 100/0 3. Fresh gas flow of 0.5L/min, % gas flow O2/N2O = 100/0 Desflurane has the highest carbon footprint in all scenarios, however sevoflurane is close in scenario 1. Propofol has the lowest carbon footprint, half of this is due to energy used to manufacturing the syringes. Scenario 2 (eliminating NO2) leads to lower carbon footprints for isoflurane and sevoflurane compared to scenario 1, however desflurane increases due to the high GWP. In scenario 3 the carbon footprint is lowest for all IAGs. The manufacturing process with method-B (lower impact) has a lower carbon footprint compared to method-A (higher impact) for all three anaesthetic gases. Reduction of the carbon footprint for the production of sevoflurane can be achieved by avoiding the use of tetrafluoroethylene (84% reduction). For isoflurane and desflurane these differences are smaller. For sevoflurane and isoflurane scenario 3, with method-B results in the lowest carbon footprint. Using method-A in all scenarios leads to a higher carbon footprint for sevoflurane compared to isoflurane, which is attributable to the manufacturing process. The US method (fresh gas flow of 2L/min), leads to a higher impact for sevoflurane compared to isoflurane. When changing this to 1L/min or 0.5L/min, combined with method-B, the carbon footprint of sevoflurane is lower than that of isoflurane. Using Vapour Capture Technology (VCT) results in lower carbon footprints for all anaesthetic gases. When using a fresh gas flow rate of 0.5L/min, with method-B as the manufacturing process, the carbon footprint is comparable to that of propofol. However, when the manufacturer of propofol uses renewable energy the carbon footprint can be cut by half. Overall, the biggest hotspot for desflurane and isoflurane is the waste IAG, for sevoflurane the manufacturing process and for propofol drug administration.   2. Waste No results in this study.   3. Medicine residue in water  No results in this study.   4. Human toxicity No results in this study.   5. Ozone Depletion No results in this study. | Propofol use results in a low carbon footprint, which can be further reduced by incorporating renewable energy into its manufacturing process. However, the environmental impact of propofol drug waste (e.g., urine excretion into sewer systems) has not been studied.  Among the inhaled anaesthetic gases (IAGs), desflurane has the highest global warming potential (GWP) and consequently the highest carbon footprint, compared to isoflurane and sevoflurane.  To lower the carbon footprint of IAGs: - Use low fresh gas flow rates (e.g., 0.5 or 1 L/min). - Avoid using tetrafluoroethylene as a raw material during synthesis. - Avoid using nitrous oxide (N₂O) during the production of isoflurane and sevoflurane.  In this study, sevoflurane has a lower carbon footprint than isoflurane when low fresh gas flow rates are used and when a more sustainable manufacturing method (Method-B, which excludes tetrafluoroethylene) is employed.  The use of vapor capture technology (VCT) could significantly reduce the carbon footprint of desflurane and, according to the authors, may make sevoflurane less carbon-intensive than propofol—assuming a recycling rate of 70%. However, this approach has not been tested in real clinical settings. Currently, the recycling and reuse of anaesthetic gases are not permitted until their safety and efficacy have been conclusively demonstrated. Also the recycling rate is an assumption. Further research on VCT is needed to assess its environmental benefits as well as its safety and efficacy in human trials. |
| McGain et al. (2021) | 1. Climate Change Three hours of propofol at 700 mg/h will result in less than 50 g CO2e. The total emissions of general anaesthesia were 14.9 kg CO2e (95% CI, 9.7 to 22.5); spinal anaesthesia 16.9 kg CO2e (95% CI, 13.2 to 20.5); and combination anaesthesia 18.5 kg CO2e (95% CI, 12.5 to 27.3). The average anaesthesia duration times were: general 161 (113 to 193) min, spinal 200 (168 to 288) min, combination 189 (128 to 241) min.   The average/mean duration of spinal and combined anaesthesia were approximately 40 and 30 minutes more than general. This leads to increased energy use (0.8 and 0.6 kg CO2e) and oxygen use (0.6 kg CO2e). Spinal anaesthetic of shorter duration will result in a decrease of 1.4 kg CO2e.  Electricity for the patient air warmer was responsible for at least 2.46 kg CO2e for all approaches. Total single-use plastics, glass and so forth were responsible for 3.5 (general), 3.4 (spinal) and 4.3 (combination) CO2e. The majority was from single-use plastics. Pharmaceuticals beyond gases were responsible for 1.2 to 1.3 CO2e (7 to 8%). For general anaesthesia, sevoflurane was responsible for an average of 4.7 kg CO2e (32%), range 2.7 to 8.6 kg CO2e. The patient who received propofol represented the minimum of 8.4 kg CO2e in the general anaesthesia group. In the combination group sevoflurane contributed for 3.1 kg CO2e (17%), range 0.6 to 10 kg CO2e.  Fro spinal and combination anesthesia, washing an sterilizing reusable gowns, plastic spinal trays and so forth contributed for 4.5 kg and 4.0 kgCO2e, respectively. Oxygen use was important for spinal, resulting in 2.8 kg CO2e (16%) with flow rates from 6 to 10 l/min (compared to 0.5 to 3.1 l/min for general and combination).   International comparisons were made by changing energy sources. Australia and China are more coal reliant, where the European Union (and UK) are more dependent on nuclear and hydro/wind/solar sources. This modelling changed the CO2e for washing and sterilizing reusable equipment and electricity for patient warming. In the EU spinal anaesthesia has a carbon footprint of approximately 60% (9.9/16.9 CO2e) of that in Australia. The general anaesthesia in Australia (total intravenous) is less than the EU average (8.4 kg vs. 11.9 kg CO2e). The minimum for spinal anaesthesia in Australia is higher than the EU average (14.7 vs 9.9 kg CO2e), due to high carbon intensity electricity required to clean reusable anaesthesia equipment.   2. Waste The total masses of single-use equipment were: general anaesthesia (mean 996 g; interquartile range 873 to 1,033 g; range 725 to 1,392 g), spinal anaesthesia (mean 997 g; interquartile range 934 to 1,076 g; range 885 to 1,184 g) and combination anaesthesia (mean 1,237g; interquartile range 1,100 to 1,285 g; range 1,009 to 1,678 g). The majority of the waste was from total plastics: average for general anaesthesia 783/996 g (78%); spinal 729/997 g (73%); and combination 932/1,237 (75%). Glass was the next most discarded material.   3. Medicine residue in water  No results in this study.   4. Human toxicity No results in this study.   5. Ozone Depletion No results in this study. | The study shows total intravenous anaesthesia has the lowest carbon footprint compared to general anaesthesia with sevoflurane, spinal anaesthesia and the two combined. The latter are comparable. Great contributors are the agents itself, oxygen use, single-use products and energy-use. International comparisons show reusables are a good alternative for single-use products when using a renewable energy sources (e.g. nuclear/wind/solar energy). |
| Sherman et al. (2012) | 1. Climate Change The results on climate change are only graphically reported in Figure 1 (Sherman, 2012). The figure shows two graphs, whereas panel A shows the results on the life cycle (as mentioned before) of the drug, agent release and N2O release and panel B shows nonwasted anaesthetic gas emissions (life cycle) from drug manufacturing, transport, drug delivery and disposal.   Considering the N2O/O2 admixture, desflurane has the biggest impact with approximately 56,000 g CO2e including agent release and N2O release, followed by sevoflurane with 46,000 g CO2e, isoflurane 24,000 g CO2e and propofol. The emissions of propofol can not be depicted from the figure (too small). When choosing for the O2/air admixture, desflurane has the greatest impact followed by isoflurane and sevoflurane.  Regarding the life cycle, desflurane has the biggest impact of 700 g CO2e, followed by sevoflurane (430 g CO2e), isoflurane (200 g CO2e) and propofol (25 g CO2e).   2. Waste No results in this study.  3. Medicine residue in water  No results in this study.  4. Human toxicity No results in this study.  5. Ozone Depletion No results in this study. | Overall desflurane has the greatest impact on the outcome climate change. Propofol is the best choice considering this outcome. The admixture of O2/air instead of NO2/O2 is the better environmental choice.  The biggest hotspot of the GHG emissions is the N2O release, followed by agent release and lastly the life cycle of the agent. When choosing for the O2/air admixture, desflurane has the greatest impact followed by isoflurane and sevoflurane. The change in impact between isoflurane and sevoflurane when using the O2/air mixture instead of NO2/O2 is attributable to the higher GWP for isoflurane and conversely the higher gas flow requirements for sevoflurane when using N2O/O2 (more N2O is used).   Considering the lifecycle of the agents, for desflurane the greatest hotspot is the agent manufacturing, followed by delivery of the drug to the patient (electricity required for volatilization) and N2O manufacturing. Sevoflurane and isoflurane have more similar profiles, with the greatest hotspot being N2O manufacturing, followed by agent manufacturing and packaging. GHG impacts of propofol are comparatively quite small and most of it is attributable to the drug delivery to the patient (energy needed to operate the syringe pump) and waste management. Although isoflurane is a stronger greenhouse gas (GWP100 510), the entire lifecycle of sevoflurane (GWP100 130) has a greater environmental impact due to the production process. This is explained by the fact that more sevoflurane (8.0 mL) than isoflurane (2.0 mL) is used per 1-MAC/hour, resulting in the need for more sevoflurane production. Consequently, the quantity of gases used must be considered when making this comparison. Nitrous oxide (310 GWP) also acts as a GHG and has a negative impact on the environment, which contributes significantly to the environmental impact. Desflurane shows a significant environmental impact, primarily due to its potent greenhouse gas (GHG) properties (GWP100 2540). Despite the use of VCT, or the avoidance of nitrous oxide, desflurane continues to have a greater impact compared to sevoflurane or isoflurane. |
| Thiel et al. (2018) | 1. Climate Change The baseline case is an average of the combination of anaesthetic approaches used in each of the 17 laparoscopic hysterectomies. This resulted with anaesthesia in 562 kg CO2e and without anaesthesia in 402 kg CO2e. Using desflurane only, resulted in an emission of 762 kg CO2e, desflurane with NO2 in 757 kg CO2e, sevoflurane with NO2 in 416 kg CO2e, sevoflurane only in 410 kg CO2e and propofol only in 402 kg CO2e.   2. Waste No results in this study.   3. Medicine residue in water  No results in this study.   4. Human toxicity No results in this study.   5. Ozone Depletion  No results in this study. | Volatile anaesthetics have a greater environmental impact compared to intravenous anaesthetics. The use of N2O increases this impact and should be avoided if possible. If a volatile anaesthetic should be used, sevoflurane seems to be the most environmentally sound choice. |
| Wang et al. (2022) | 1. Climate Change The median total carbon footprint for general anaesthesia was 4725 kg versus 70 grams for spinal anaesthesia. In total, 6 general anaesthesia cases used desflurane, which contributed to a high carbon footprint. The mean carbon footprint by general anaesthesia resulted in 22,707 g versus 63 grams for spinal anaesthesia.   Another comparison involved the carbon footprint of general anaesthesia with the use of desflurane versus the use of only sevoflurane. For the patients that used only sevoflurane, the carbon footprint resulted in a total of 4802 grams, and the 6 cases where desflurane was used resulted in 154,008 grams CO2 eq.   2. Waste No results in this study.   3. Medicine residue in water  No results in this study.   4. Human toxicity No results in this study.   5. Ozone Depletion  No results in this study. | Spinal anaesthesia for TLIF resulted in a lower carbon footprint compared to general anaesthesia. Desflurane is a greater contributor to climate change compared to the use of sevoflurane for general anaesthesia. |
| ***Topic 5 "Operating room air ventilation and air handling systems"*** |  |  |
| Alsved et al. (2018) | 1. Climate Change No results.   2. Energy use Energy use for ventilation power per type of airflow was expressed in kW. For TMA this resulted in 2.8 kW, LAF in 8.0 kW and TcAF in 5.7 kW. | LAF lead to the greatest energy use, followed by TcAF and TMA. To reduce the energy use, and thereby environmental impact, airflow using a lower energy setting can be used. TcAF is more energy efficient than LAF, and still provides high air cleanliness. |
| Marsault et al. (2021) | 1. Climate Change No results reported.   2. Energy use When decreasing air influx from 100% to 50% using LAF, energy consumption was reduced by 41% (P = 0.0007) (Table V, Marsault, 2021). For TAF energy consumption was reduced by 51% (P = 0.0007) when reducing air influx from 100% to 50%. | Decreasing air influx leads to lower energy consumption. Next to that, TAF has a lower energy consumption compared to LAF, however it is a small difference. |

### Table 1f: Comments LCA studies

| Study reference | Comments |  |
| --- | --- | --- |
| Author and year | Authors conclusion | Limitations study |
| ***Topic 1 "Surgical techniques"*** |  |  |
| Papadopoulou et al. (2022) *(see S2)* |  |  |
| Power et al. (2012) | The CO2 emissions of MIS in the US, when considering both direct and indirect factors, have a significant environmental impact. This should be considered to reduce healthcare’s CO2 footprint while maximizing healthcare quality. | Not all disposable instruments were included in the analysis, and emissions related to manufacturing, transportation, and energy use of these instruments were excluded. Additionally, emissions associated with preoperative and postoperative stays were not considered. These emissions can vary depending on the surgical procedure and the length of stay, which can significantly impact the overall CO2 footprint.  Electricity usage was assumed to be equivalent across operating techniques, though it likely differs between methods. For example, minimally invasive surgery (MIS) relies on electricity-driven instruments and cameras, while robotic-assisted surgery involves robotic arms that consume additional electricity.  Moreover, different methodologies were employed to quantify the carbon footprint. The EIO-LCA model was used to estimate emissions from CO2 capture and compression. Transportation-related emissions were calculated using a carbon footprint calculator based on US DOT fuel efficiency data and GHGPI mobile guides. For plastic incineration, a carbon footprint calculator was used with the assumption that 1 kg of plastic produces 6 kg of CO2.  Given these limitations, the results should be interpreted with caution. |
| Thiel et al. (2015) | The trend is towards MIS that drives up the amount of pollution generated within the OR. | The environmental impact of postoperative stay was not taken into account. |
| Woods et al. (2015) | An increased environmental impact of RA-LSC and LSC over LAP is identified. The future healthcare sustainability research should include development of strategies to mitigate the environmental effects of healthcare while improving safety, quality and cost-effectiveness. | The results are based solely on disposal and energy use, excluding emissions from production, transportation, and product reuse. As a result, the entire life cycle is not accounted for. Additionally, the life cycle of pharmaceuticals and emissions associated with preoperative and postoperative stays (including length of stay) were not considered.   Furthermore, no comprehensive life cycle assessment (LCA) method was applied. Data on energy and waste greenhouse gas (GHG) equivalents were sourced from the National Energy Foundation (NEF), the US Energy Information Administration, and previous carbon footprinting studies. It was assumed that 1 kg of waste in a municipal landfill is equivalent to 1 kg of CO2 released into the environment. Energy usage, measured in kWh, was converted using the NEF calculator's conversion factor of 0.54 kg CO2eq/kWh. These broad assumptions introduce uncertainties into the analysis.  Therefore, the results should be interpreted with caution. |
| ***Topic 2 "Disposables versus reusables"*** |  |  |
| Almutairi et al. (2022) | PPE selection could be enhanced by considering their environmental impact, materials, and country of origin. Overall, it is recommended to prioritise reusable PPE over disposable options. | The incineration plant was very close to the hospital, whereas the impact from transportation could not reflect the average travel distance for other facilities. Incineration was modeled despite the fact that the waste management company does not incinerate all decontamination waste, but this was done to make the paper more applicable to most international settings. |
| Boberg et al. (2022) | In the Swedish setting, the reusable trocar system is preferred over the single-use and mixed trocar system to reduce the environmental impact and financial costs of laparoscopic cholecystectomies, with no harm to quality of care. | Validity of the findings uncertain for other trocar systems that are not included in the study. There are uncertainties in the modelling and input data. Materials and productions of capital goods included in the sterilisation process were excluded. |
| Boucheron et al. (2022) | Implementing single-use cystoscopes would lead to a waste reduction. Further studies are needed to provide a comprehensive and rigorous life cycle assessment. | Economic evaluation is not generalisable. Furthermore, the entire lifecycle is not included, so waste and water use is evaluated but not environmental impact of the whole life cycle. As the authors recognise: comparing the environmental impact based solely on waste is limited. It is misleading that the author use 'environmental impact' in the title of the article. Furthermore, as mentioned in the interpretation: specific details about the type of waste are not provided. This aspect could be further be defined to enable the evaluation and optimisation of different strategies for reducing (unnecessary) waste. It is possible that more waste is being generated than necessary. |
| Davis et al. (2018) | The carbon footprint of the single use and reusable ureteroscopes is comparable. Informed clinicians should be willing to advocate for changes within the healthcare delivery and within the manufacturing industry to maintain healthcare quality, cost-effectiveness and safety in the future. | The data was sourced from online databases without the use of a Life Cycle Assessment (LCA) methodology (see interpretation section). The study was conducted in a single centre, and while the authors claim to have used 'standardized carbon footprint protocol guidelines,' no specific reference to the protocol is provided. Consequently, it is unclear how the assessments were determined. |
| Donahue et al. (2020) | By using acrylic specula for over a period of 1 year (5875 disposable acrylic specula), 5153 kg CO2e and 5462 kg solid waste were produced. By changing to steel grade 304 of grade 316 specula (100 uses average), greenhouse gas emissions could have been reduced by 75& and 74% respectively with a significant decline in end-of-life waste generation (both 64.43 kg). Health systems might consider environmental impact in addition to costs and clinical efficacy when choosing medical instruments. | A limitation could be that multiple assumptions were made in the analysis, mainly regarding production and reprocessing, due to lack of data from manufacturers and other sources. The authors choose to use the less carbon intensive approach for the acrylic specula and the more carbon intensive approach for the steel specula, to ensure any difference shown would be robust. Next to that, the study was limited by the lack of life cycle data on high level disinfectants such as glutaraldehyde, ortho-phthalaldehyde and peracetic acid. |
| Drew et al. (2021) *(see S2)* |  |  |
| Eckelman et al. (2012) | The results suggest the reusable LMA has a lower life cycle environmental impact compared to the disposable LMA at Yale New Haven Hospital, across all categories of concern. | This study did not analyse the environmental health impacts during the use of an LMA, where intraoperative exposure to some parts of the plastics could contribute increasing the outcome human toxicity. |
| Friedericy et al. (2022) | The rigid sterilisation container has 84% less eco-costs compared to blue wrap. The container is more environmentally friendly. | Only outcomes on eco-costs are shown in sensitivity analysis for use phase. |
| Grimmond et al. (2012) | RSC significantly reduced climate impacts over DSC, with manufacturing and transport as the major contributors to the climate impacts of DSC. Larger containers have less climate impacts, transport distances and electricity sources are important factors. | The study is conducted in the USA with all processes related to 1 hospital, outcomes might changes for other hospitals and countries. |
| Grimmond et al. (2021) | RSC achieved significant GHG reductions over SSC, container manufacture was the largest contributor in SSC, for RSC it was transport. RSC lifespans can be reduced and achieve marked GWP reductions over SSC. Adoption of reusable over SSC can reduce GHG emissions permanently with minimal staff behavioural change. | Results of SSC has been extrapolated from 17 trusts to 40 trusts and therefore the representativeness of data might not be accurate. |
| Hicks et al. (2016) | The energy consumption was found to be much less during the lifetime of the reusable hospital gown than continuously using disposables. This suggests that nAg-enabling of reusable hospital gowns may be a method for simultaneously lowering the environmental impact and maintaining the antimicrobial performance needed to combat pathogen transmission. | Only one attachment and synthesis process was analysed. The environmental impact of excess silver during synthesis and the silver lost is not explored. The comparisons of reusable and disposable gowns relies on prior work and utilises only one impact category. |
| Hogan et al. (2022) | It is important to define the carbon footprint of new technologies. According to this study, the carbon footprint of single-use cystoscopes is higher than that of reusables. | Data are limited to a single-centre. The data is from online sources, and not directly attributable to the investigated centre. Sterilisation is based on Australian data. It is not clear what is included in the waste calculations. Authors also refer to Davis et al., where the authors of Davis et al. claim to have used 'standardized carbon footprint protocol guidelines,' but no specific reference to the protocol is provided. Consequently, it is unclear how the assessments were determined. Also see interpretation. |
| Ibbotson et al. (2013) | The eco-efficiency results indicated that the stainless steel reusable scissor is the option with the lowest environmental impact and is next to that, cheapest. | Data sources were not comparable between the scissors, since the plastic disposable and stainless steel reusable data was obtained from company data and the stainless steel disposable scissor data was obtained from literature. Data on electricity was not available (located in Asian countries), so another energy mix was used. This also accounted for other data like recycling data - for stainless steel recycling, data from Australian data 2007 was used (which may not reflect the German setting). This results in a situation that could not be totally applicable for the German situations studied. |
| Keil et al. (2022) *(see S2)* |  |  |
| Kemble et al. (2022) | The carbon footprint of reusable cystoscopes is less compared to SU, regardless of case volume. Energy consumption of reprocessing is an environmental hotspot for the reusable device. | The data used for calculating the CO₂ footprint is derived from previous studies, with limited transparency regarding how certain assessments were conducted. Differences in settings and assumptions are not fully accounted for, which introduces uncertainty. As a result, the findings should be interpreted with caution. Furthermore, the impact of detergents is not included. |
| Le et al. (2022) | Using SD comes to a higher cost for the environment compared with reusable duodenoscopes. | Ascertainment bias can be a limitation regarding infection cases. Key assumptions have been made, however clearly described. Direct information from manufacturers on material composition and manufacturing energy is lacking. Instead, data from previous studies was used, with unclear origins. Recycling was not accounted for. |
| Leiden et al. (2020) | The authors conclude the environmental impact of the disposable system was significantly lower in all impact categories. This is mainly due to the high impact of the steam sterilisation process and the big size of the reusable instruments sets. | The comparison between the disposable and reusable sets is based on a single surgery, with a correction made for the reusable set, accounting for only a three hundredth of the weight in production and disposal processes. To better reflect reality, the functional unit should have been 300 surgeries, highlighting a limitation in the analysis.  Considering the tray content is crucial; the reusable set weighs 45.5 kg, but only a portion is used based on surgery requirements, while the disposable set is a fixed 2.0 kg. Streamlining the reusable set could mitigate its environmental impact.  Moreover, sterilisation is performed twice—before and after surgery. For a more accurate representation if the instruments are owned by the hospital, it should be considered only once, as the dual inclusion may negatively impact the results for the reusable tray, this is addressed in the sensitivity analysis. |
| McGain et al. (2010) | The author concludes that financial and environmental savings of a hospital converting to reusable trays are important, and that it seems difficult to justify persisting with single-use drug trays, particularly with added cotton gauze | Data were average industry data and not directly measured (as with most LCA models). Data from tray manufacturers were unavailable, therefore data of average manufacturing effects were used. For the single-use trays the European energy mix is used, however the Chinese energy mix might be more coal reliant. |
| McGain et al. (2012) | For hospitals using coal-fired electricity, the environmental effects are greater when using reusable kits instead of single-use. Reducing the environmental impact of the reusable kit is possible by focusing on the inefficiencies and energy sources of steam sterilisers. | No life cycle inventory data was available for the single-use kit (from China and Pakistan) and therefore local Australian inventory data was used for manufacturing of plastics for both reusable and single-use kits, and European data for metal components. |
| McGain et al. (2017) | The financial and environmental impact of anaesthetic equipment are investigated. Using single-use equipment costs more than using reusables, in all scenarios. Converting from single-use to reusable leads to an increase in CO2 emissions of almost 10%, where it decreases when converting in the US (50%) and UK/Europe (85%). | Sterilisation records and input from senior Central Sterile and Supply Department staff at hospital 1 were used to define sterilisation mode and load information, when 2 hospitals were involved. Comparing or using data from both hospitals would have been more accurate. This also accounts for electricity consumption of the steriliser. |
| McPherson et al. (2019) | While large RSC transport distances reduce the differential between DSC and RSC GHG, RSC still achieves significant GHG reductions over DSC. Transport and electricity cleanliness play a crucial role. The lifespan of RSC has a minimal effect on GHG emissions. Purchasing decisions can contribute to reduction strategies. Institution-wide adoption of RSC can effectively reduce GHG emissions with minimal staff behaviour change. | A limitation was the assumption made in the location of the polymer manufacturer for DSC. It was assumed to be close to the DSC manufacturer. Second, the use of the UK database for transport (because it used tonne.km). |
| Morris et al. (2022) | The impact of stainless steel speculums versus disposable speculums were calculated and analysed. All scenarios of stainless-steel speculums have less impacts in five impact categories, while the acrylic scenarios outperformed on one impact category. Four other impact categories were inconclusive, since there was overlap in the uncertainty analysis. Through breakeven analysis the authors found the disposable speculum surpasses for multiple impact categories, except ozone depletion. | Assumptions had to be made since there was lack of data from manufacturers, however this is a very common problem in LCAs and has been accounted for in sensitivity analyses. The material of the stainless steel speculum was not clear, since the manufacturer could not be recognised from the speculum, or it could not be obtained. |
| Namburar et al. (2021) | The quantitative assessment shows that endoscopic procedures generate a large amount of waste from disposable instruments. Net waste is increase by using single-use endoscopes. | The study suggests to estimate the environmental impact of an endoscopic procedure, however only describes the amount of waste and does not calculate the actual environmental impact. |
| Rizan et al. (2021) | The CO2 footprint of using hybrid instruments for laparoscopic cholecystectomy is around a quarter of that for single-use equivalents and the financial costs around half. | Data is limited by assumptions (as with all LCAs), however clearly explained. |
| Sanchez et al. (2020) | Environmental considerations will never be paramount in decision making around medical devices or healthcare delivery, however this work shows there are many opportunities to reduce resource use, waste and environmental impact. | There is data uncertainty associated with some of the modelling parameters (e.g. energy and BP cuff materials). |
| Sherman et al. (2018) | The results demonstrate a clear benefit of reusable laryngoscope handles and blades over single-use alternatives, with HLD as the least polluting reprocessing method. | The outcomes are only expressed in percentages (except climate change). It would give a more clear view of the absolute impact if the absolute numbers were stated. The authors state there is an uncertainty test undertaken, however results are not discussed. |
| Sorensen et al. (2022) | The authors conclude three things are learned from this study: 1. The total material use should be considered (e.g. also ppe use in the reuse phase) 2. Energy usage should be considered in the reuse phase 3. Reusing materials of single-use products lowers the environmental impact of the product | There is uncertainty regarding the results related to energy use in the reuse phase (washing and drying). It was hard to obtain empirical data, and therefore technical data was used. Data related to the phthalates in the tubes was not included. The reusable monitor for the tube with the integrated camera is not included. |
| Unger et al. (2014) | The reusable dental bur will decrease negative environmental impacts, when efficient loading capacity is achieved. Furthermore packaging should be optimised and other packaging materials with lower environmental impacts should be explored as a next step. | Data input was general, however the authors state that more specific data on e.g. electricity mix could have improved the results' accuracy. The disposal was based on Swedish inventory data, since US data was not available. |
| Vozzola et al. (2018) | This analysis, combined with agreement of previous partial life cycle studies of other medical textiles, makes it clear that the environmental benefit of reusable isolation gowns is significant. | Funding could potentially be a source of bias. Different energy mixes are not taken into account, this potentially limits the representativeness of the results for other parts of the world |
| Vozzola et al. (2020) | The current study adds to the body of evidence that shows the environmental superiority of reusable surgical gowns. | The blue water comparisons’ accuracy is limited due to lack of data on water content of soiled gowns.  Not all disposable gowns are produced in China or sterilised with ethylene oxide (what is used in this study).  Packaging of disposable and reusables vary. |
| ***Topic 3 "Cover materials"*** |  |  |
| Nowack et al. (2012) *(see S2)* |  |  |
| ***Topic 4 "Anaesthetics"*** |  |  |
| Hu et al. (2021) | Both isoflurane and sevoflurane have a smaller life-cycle carbon footprint compared to desflurane in all scenarios. It is optimal to use a low fresh gas flow rate, avoid using tetrafluoroethylene as raw material to synthesize IAGs and to avoid using NO2 when using isoflurane or sevoflurane. VCT reduces carbon footprint of IAGs. | Assumptions has been made and data should be interpreted with caution.  - recycling rate and reuse of VCT is an assumption |
| McGain et al. (2021) | Carbon footprints for knee replacement anaesthesia (general, spinal, combined) were similar, with significant overlap between the CIs. | Small study with 30 samples, however possibly not more needed. One centre, difficult to generalize results. Lack of other anaesthetics (e.g. desflurane, intravenous anaesthesia separately). Great contributors to the use of spinal anaesthesia are oxygen use, and the use of sterile gowns and towels. It can be questioned if this is really necessary for spinal anaesthesia. |
| Sherman et al. (2012) | The results reiterate previous published data, while providing the life cycle data on the anaesthetic drugs. Clinicians should consider the full environmental and human health impacts from anaesthetic use. | There is uncertainty regarding the synthesis of propofol and the volatile drugs and results should be carefully interpreted. |
| Thiel et al. (2018) | Available interventions can be used with promising results to reduce the carbon footprint. | Uncertainty due to limited or lack of LCA data in healthcare. One location included in calculations, poor generalizability. More interventions possible than studied. |
| Wang et al. (2022) | Spinal anaesthesia use in single-level TLIF is superior compared to general anaesthesia for reducing the carbon footprint and it is also effective and safe in lumbar surgery. | Only anaesthetic agents are included in the calculations, and materials and energy use used are excluded. Also only cradle-to-gate is included, so the use phase and disposal are excluded. Also for the inhaled anaesthetic agents, production is excluded. |
| ***Topic 5 "Operating room air ventilation and air handling systems"*** |  |  |
| Alsved et al. (2018) | Comparison of three ventilation systems in three identical ORs showed that LAF and TcAF provide high air cleanliness. Cfu levels of TMA are too high. TcAF is more energy efficient and comfortable to work in than LAF, and still provides high air cleanliness. | Many designs exist for TMA and LAF ventilations (e.g. higher/lower airflows). Many other versions are available and not included in the study. |
| Marsault et al. (2021) | Lowering air influx by 50% in LAF did not significantly affect cfu or particles, but reduced energy consumption. | A heated dummy was used instead of a patient, as a result there was no bleeding or moisture what could have influenced results on e.g. particle count. |

### Table 2a: Systematic reviews evidence table

| Study reference | Study reference | Study characteristics |  |  |  |  |  |  |
| --- | --- | --- | --- | --- | --- | --- | --- | --- |
| Author and year | Title | Journal | Aim/objective | Literature search | Framework used | Study design | Setting and country | Source of funding and conflict of interest |
| ***Module 1 "Surgical techniques"*** |  |  |  |  |  |  |  |  |
| Papadopoulou et al. (2022) | Environmental sustainability in robotic and laparoscopic surgery: systematic review | British Journal of Surgery | To systematically review the MIS literature with the goal to investigate the carbon footprint of MIS, and other environmental impacts of MIS. | Literature search between 1974 and July 2021 | In accordance with 2020 PRISMA guidelines and the Cochrane Handbook of Systematic Reviews of Interventions | LCA, observational and interventional studies | MIS, UK | A grant by the funding body University College London was received, under the UCL Connected Learning Internships Scheme, project ID: 35. The funding body did not have a role in the study design, collection, analysis and interpretation of data, writing the report and the decision to submit for publication. |
| ***Module 2.1 "Disposables versus reusables"*** |  |  |  |  |  |  |  |  |
| Drew et al. (2021) | Operating in a Climate Crisis: A State-of-the-Science Review of Life Cycle Assessment within Surgical and Anesthetic Care | Environmental Health Perspectives | To summarise the state of LCA practice via review of literature assessing the environmental impact of related services, procedures, equipment and pharmaceuticals. | Literature search up to May 2020 | The review used STARR-LCA, which is a PRISMA-based framework. | LCA | Anaesthetic and surgical care, Canada. | None stated |
| Keil et al. (2022) | The impact of switching from single-use to reusable healthcare products: a transparency checklist and systematic review of life-cycle assessments | European Journal of Public Health | To examine the change in environmental impact when switching from single-use to reusable products in healthcare. | Literature search up to September 2021 | The review used the PRISMA framework and used STARR-LCA. | LCA | Focus on healthcare products, study conducted in Germany | No conflicts of interest. Research was carried out on behalf of two German universities, which are publicly funded. No further funding was received. |
| ***Module 3 "Cover materials"*** |  |  |  |  |  |  |  |  |
| Nowack et al. (2012) | Review and downscaling of life cycle decision support tools for the procurement of low-value products | International Journal of Life Cycle Assessment | SR of three life cycle assessments to identify environmental indicators for procurement decisions of low-value products. | Literature search up to 2012 | - | LCA | Germany | None stated |
| ***Module 4 "Anaesthetics"*** |  |  |  |  |  |  |  |  |
| - |  |  |  |  |  |  |  |  |
| ***Module 5 "Operating room air ventilation and air handling systems"*** |  |  |  |  |  |  |  |  |
| - |  |  |  |  |  |  |  |  |

### Table 2b: Systematic reviews evidence table

| Study reference | Product/service characteristics |  |  |  | Intervention (I) and Comparison/Control (C) | Follow-up |  |
| --- | --- | --- | --- | --- | --- | --- | --- |
| Author and year | Inclusion criteria | Exclusion criteria | Functional unit (as stated in SR) | Included studies | I and C | End-point follow-up | For how many participants no complete outcome data available? |
| ***Module 1 "Surgical techniques"*** |  |  |  |  |  |  |  |
| Papadopoulou et al. (2022) | Full-text articles in English, addressing environmental sustainability and MIS. LCA's, observational and intervention studies were included. | Case reports, conference abstracts, letter to the editor, opinion articles, reviews and articles not written in English. Studies including open surgery and MIS where findings were not seperately reported were excluded. Cost-effectiveness studies not reporting about environmental sustainability were excluded. | - | 16 included studies | These studies examined the environmental impact from MIS. | - | - |
| ***Module 2.1 "Disposables versus reusables"*** |  |  |  |  |  |  |  |
| Drew et al. (2021) | Studies which assessed the environmental impact(s) of (1) an operating room(s) using LCA, (2) a specific surgical procedure(s) using LCA or (3) equipment or pharmaceuticals used in surgical settings. | No access, no English language, no research in relation to healthcare, healthcare related but not related to surgery or anaesthesiology, no use of LCAs. | - | 44 included studies | These studies examined the impact contributions from  (A) ORs generally (n=1)  (B) specific surgical procedures (n=10) (C) provision and use of surgical or anaesthetic equipment or pharmaceuticals (n=33) | - | - |
| Keil et al. (2022) | Written in English or German, comparison of single-use and reusable healthcare products, reported quantitative outcomes for at least one type of environmental impact. | full cradle-to-grave analysis. | - | 27 included studies | The studies examined the environmental impact of single-use versus reusable healthcare products. | - | - |
| ***Module 3 "Cover materials"*** |  |  |  |  |  |  |  |
| Nowack et al. (2012) | Sound methodology, verifiability, completeness and actuality. | Foreign language | A: 1 gown B: 1 operation C: 1 gown | 3 included studies  A: Schmidt, 2000 B: IFEU, 1996 C: Ponder, 2009 | Intervention: A: Reusable OR textiles (CO/PES PES) B: Reusable OR textiles (CO, CO/PES) C: Reusable OR textiles (CO/PES)  Control: A: Single-use OR textiles (Pulp/PES, pulp/PES/PE) B: Single-use OR textiles (Pulp/PE/PES) C: Single-use OR textiles (PP SMS) | - | - |
| ***Module 4 "Anaesthetics"*** |  |  |  |  |  |  |  |
| - |  |  |  |  |  |  |  |
| ***Module 5 "Operating room air ventilation and air handling systems"*** |  |  |  |  |  |  |  |
| - |  |  |  |  |  |  |  |

### Table 2c: Systematic reviews evidence table

| Study reference | Outcome measures and effect size | Comments |  |
| --- | --- | --- | --- |
| Author and year |  | Authors conclusion | Interpretation of results |
| ***Module 1 "Surgical techniques"*** |  |  |  |
| Papadopoulou et al. (2022) | 1. Climate change Six studies reported on carbon footprint, of which three provided the carbon footpint per case (cataract phacoemulsification - India; endometrial cancer staging laparoscopy - USA; endometrial cancer staging RA-laparoscopy - USA; laparoscopic hysterectomy - USA; robotic hysterectomy - USA). Lowest carbon footprint is 6 kg CO2 eq per case for cataract phacoemulsification in India, and highest for robotic hysterectomy in the USA (814 kg CO2 eq).  Production of disposables and anaesthetics were the main contributors in high emission minimally invasive hysterectomies. Robotic procedures had increased emissions over laparoscopic procedures. Two studies calculated yearly CO2 emissions, and this resulted in 355924 tonnes CO2/year in the USA and 35384 tonnes CO2 in 2007 in Japan (however, scope 1 emissions were excluded).  2. Waste 11 studies addressed waste, of which 4 studies reported the waste per case (cataract phacoemulsification - India; phacoemulsification surgery - Malaysia; endometrial cancer staging laparoscopy - USA; endometrial cancer staging RA-laparoscopy - USA; laparoscopic hysterectomy - USA; robotic hysterectomy - USA). Cataract phacoemulsification in India had the lowest amount of waste, producing 0.25 kg per case. Robot-assisted laparoscopic hysterectomy had the highest waste production, higher than laparoscopic surgery (14.3 vs. 11.2 kg). Six of the eleven studies provided data on waste classification. Three studies showed interventions to decrease the amount of waste.  3. Acidification The production of disposables is the main contributor to acidification (regarding hysterectomy in the USA). Regarding phacoemulsification in India, the main contributor is the production and processing of reusables.  4. Eutrophication The production of disposables is the main contributor to eutrophication (regarding hysterectomy in the USA). Regarding phacoemulsification in India, the main contributor is the production and processing of reusables.  5. Human Toxicity The production of disposables is the main contributor to human toxicity (regarding hysterectomy in the USA). Regarding phacoemulsification in India, the main contributor is the production and processing of reusables.  6. Ecotoxicity The production of disposables is the main contributor to ecotoxicity (regarding hysterectomy in the USA). Regarding phacoemulsification in India, the main contributor is the production and processing of reusables.  7. Ozone Depletion Main contributor to ozone depletion is the production of disposables and anaesthetic use (regarding hysterectomy in the USA). Regarding phacoemulsification in India, the main contributor is the production and processing of reusables. | MIS contributes to the overall GHG of healthcare, and the main contributors are the production of disposables, anaesthetic gases, energy use and disposal. Hotspots differ per setting, however robotic procedures seem to have a higher carbon footprint and waste production. Effective interventions need to be identified and implemented. | Overall, MIS contributes to the environmental impact of surgery. Main hotspots are production of disposables, anaesthetic gases, energy use and disposal. Robotic procedures should be carefully considered per case, since they have a greater environmental impact compared to conventional laparoscopic surgery. Environmental sustainability should be considered already by the design phase of (new) technologies. |
| ***Module 2.1 "Disposables versus reusables"*** |  |  |  |
| Drew et al. (2021) | 1. Climate change (A) Operating rooms The climate impact of the hospitals’ surgical suites ranged from 3,200,000 to 5,200,000 kg CO2e per year and between 146 and 232 kg CO2e per operation (when compared on a caseload basis).   (B) Surgical procedures The outcomes on climate change were found to vary considerably (6-1,007 kg CO2e). See figure 2 (Drew, 2021).   (C) Equipment and materials and pharmaceuticals  Most disposable equipments/materials were more harmful for the environment compared to reusables. Figure 4 (Drew, 2021) includes the other outcome measures for provision and use of disposables relative to functionally equivalent reusables. For use of pharmaceuticals, GHG emissions from propofol were considerably lower than inhalational agents (i.e., desflurane, isoflurane and sevoflurane).   2. Waste No results specifically reported in this SR.   3. Acidification No results specifically reported in this SR.   4. Eutrophication No results specifically reported in this SR.   5. Human Toxicity No results specifically reported in this SR.   6. Ecotoxicity No results specifically reported in this SR.   7. Ozone Depletion No results specifically reported in this SR. | LCA data indicates the environmental burden attributable to the services is substantial and effective mitigation strategies are already available. Eligible studies varied in terms of quality, completeness and risk of bias, with critical appraisal scores varying between 44% and 89%.   (A) Only one study is found comparing different ORs on environmental impact and identifying hotpots. Results could not be pooled. (B) The studies varied considerably in their system boundaries and functional units, which leads to heterogeneity of the studies. Results could not be pooled. (C) Functional units varied considerably between the studies. There is a high degree of heterogeneity, in terms of studied items and methodology. | (A) For the OR certain emission hotspots were identified: use of anaesthetic gases and use of HVAC. (B) OR energy was a great hotspot, mainly due to HVAC. Next to that provision and use of anaesthetic gases and production of equipment and consumables contribute mainly.  (C) Considering the life cycle of single-use items, the most contributing phase is the production phase. Single-use items are more often worse for the environment compared to reusables. When using reusables the energy source has to be taken into account, since the reuse phase is the biggest contributor, which requires energy. |
| Keil et al. (2022) | 1. Climate change Non-invasive medical devices: four of six paper reported reductions in GHG emissions (13-100%) Inhalers: two studies reported reduction in GHG emissions of 50% and 63% when switching from single-use to reusable RESPIMAT inhalers Invasive medical inhalers: This category was most heterogenous. Most of the ten studies reported GHG Emissions, which varied between a reduction of 99% and an increase of 227%. Mean value was -47%. Using the Australian energy mix (coal-based) resulted in higher GHG emissions for reusables. PE: All studies reported lower GHG emissions for reusables.  2. Waste Non-invasive medical devices: four of six paper reported reductions in waste (99%)  3. Acidification No results specifically reported in this SR (only in boxplot).   4. Eutrophication No results specifically reported in this SR (only in boxplot).   5. Human Toxicity No results specifically reported in this SR (only in boxplot).   6. Ecotoxicity PE: Marine ecotoxicity increase (+13%) for 1 study, other studies show decrease.  7. Ozone Depletion Data from Morris and Hicks show a greater ozone depletion potential (7300-7500%), but other five studies show reduction of an average of 43%. | The switch from single-use to reusable healthcare products results environmental impacts in all categories, except water use. It is important to distinguish between different groups, since differences can appear. When switching to reusables, it is important to increase the number of uses and use full loading capacity in the autoclave for cleaning and disinfection. | Overall, reusables seem to have an advantage over single-use healthcare products. However, it is important to be aware of the energy mix, as this can work negatively towards the use of reusables. Furthermore, when using reusables, the number of uses should be maximised, and autoclave loading capacity should be fully utilised. |
| ***Module 3 "Cover materials"*** |  |  |  |
| Nowack et al. (2012) | 1. Climate change (CO2 footprint/Global Warming Potential (GWP)) A: Schmidt (2000) describes only surgical gowns and no surgical drapes. Surgical gowns are not included in the research question of this module. Therefore this study is no longer included in the results.  B: IFEU (1996) describes the outome measure CO2 (g) of 30 use cycles (uc) and 75 uc of reusable and single-use drapes. There are three different kind of reusable drapes (cotton, blended fabric and microfiber) compared to single-use non woven drapes. The impact on CO2 (g) for the reusable cotton drape is 6,037 g CO2 for 30 uc and 5,075 g CO2 for 75 uc. For the reusable blended fabric drape this results in 5,110 g CO2 for 30 uc and 4,154 g CO2 for 75 uc and for the reusable microfiber drape in 5,940 g CO2 for 30 uc and 4,716 g CO2 for 75 uc. The single-use non woven drape results in an impact of 3,886 g CO2 for 30 uc and 3,886 g CO2 for 75 uc.  C: Ponder (2009) describes the outcome measure CO2 (kg) of 75 use cycles of reusable (CO/PES) drapes in comparison to 75 use cycles of single-use (PP-SMS) drapes. The CO2 (kg) for the reusable variant is 5.71 kg CO2 compared to 20.50 kg CO2 for the single-use drapes.  2. Waste B: IFEU (1996) describes the outome measure waste (g) of 30 use cycles (uc) and 75 uc of reusable and single-use drapes. There are three different kind of reusable drapes (cotton, blended fabric and microfiber) compared to single-use non woven drapes. The waste (g) from the reusable cotton drape is 6,163 g for 30 uc and 4,210 g for 75 uc. For the reusable blended fabric drape this results in 5,830 g for 30 uc and 3,890 g for 75 uc and for the reusable microfiber drape in 7,057 g for 30 uc and 4,672 g for 75 uc. The single-use non woven drape results in 3,735 g for 30 uc and 3,735 g for 75 uc.  C: Ponder (2009) describes no results on the outcome waste.  3. Water use  B: IFEU (1996) describes the outome measure water consumption (l) of 30 use cycles (uc) and 75 uc of reusable and single-use drapes. There are three different kind of reusable drapes (cotton, blended fabric and microfiber) compared to single-use non woven drapes. The water consumption (l) from the reusable cotton drape is 4,690 l for 30 uc and 1,965 l for 75 uc. For the reusable blended fabric drape this results in 2,891.30 l for 30 uc and 1,241.90 l for 75 uc and for the reusable microfiber drape in 239.4 l for 30 uc and 192.80 l for 75 uc. The single-use non woven drape results in 22.2 l for 30 uc and 22.2 l for 75 uc.  C: Ponder (2009) describes the outcome measure water consumption (kg) of 75 use cycles of reusable (CO/PES) drapes in comparison to 75 use cycles of single-use (PP-SMS) drapes. The water consumption (kg) for the reusable variant is 1,373.83 kg compared to 0.00 kg for the single-use drapes.  4. Energy use  B: IFEU (1996) describes the outome measure energy use as energy consumption (MJ) of 30 use cycles (uc) and 75 uc of reusable and single-use drapes. There are three different kind of reusable drapes (cotton, blended fabric and microfiber) compared to single-use non woven drapes. The energy consumption (MJ) from the reusable cotton drape is 99,314 MJ for 30 uc and 83,567 MJ for 75 uc. For the reusable blended fabric drape this results in 94,174 MJ for 30 uc and 72,878 MJ for 75 uc and for the reusable microfiber drape in 111,616 MJ for 30 uc and 85,527 MJ for 75 uc. The single-use non woven drape results in 96,428 MJ for 30 uc and 96,428 MJ for 75 uc.  C: Ponder (2009) describes the outcome measure energy use as net energy input (input-recovery) in MJ of 75 use cycles of reusable (CO/PES) drapes in comparison to 75 use cycles of single-use (PP-SMS) drapes. The net energy input (input-recovery) for the reusable variant is 65.05 MJ compared to 225.95 MJ for the single-use drapes. | This review on the existing LCAs available on OR textiles show it is not recommended to base the procurement decision on the existing LCAs. This is due to the variance of methodological strength, incompleteness of data, outdated data, variability of data and complexity. | The study from IFEU 1996 is outdated, since OR textiles have developed over the years. Next to that, it is not clear how the data is gathered and what impact the different phases of the life cycle have (e.g. production phase, use phase, disposal etc.) and if all the phases are even taken into account. The most recent study from Ponder (2009) suggests the reusable drapes have a lower environmental impact compared to the disposable drape. However, it is still necessary to conduct more research since the authors state the data can substantially differ between countries. In contrast to IFEU (1996), Ponder has not included other scenarios (e.g. different uc or different energy mixes) in the analysis.   It was unable to include and pool the data from the different studies included in the review, since they were not comparable, and not available in English or Dutch language. |
| ***Module 4 "Anaesthetics"*** |  |  |  |
| - |  |  |  |
| ***Module 5 "Operating room air ventilation and air handling systems"*** |  |  |  |
| - |  |  |  |

## Appendix 5. Critical appraisal and GRADE

### Table 3: Risk of bias table for intervention studies

(cohort studies based on risk of bias tool by the CLARITY Group at McMaster University)

| Author, year | Selection of participants  Was selection of exposed and non-exposed cohorts drawn from the same population? | Exposure  Can we be confident in the assessment of exposure? | Outcome of interest  Can we be confident that the outcome of interest was not present at start of study? | Confounding-assessment  Can we be confident in the assessment of confounding factors? | Confounding-analysis  Did the study match exposed and unexposed for all variables that are associated with the outcome of interest or did the statistical analysis adjust for these confounding variables? | Assessment of outcome  Can we be confident in the assessment of outcome? | Follow up  Was the follow up of cohorts adequate? In particular, was outcome data complete or imputed? | Co-interventions  Were co-interventions similar between groups? | Overall Risk of bias |
| --- | --- | --- | --- | --- | --- | --- | --- | --- | --- |
| Alsved (2018) | Definitely no | Probably yes | Definitely yes | Definitely no | Definitely no | Probably yes | Probably yes | No information | Some concerns |
| Boucheron (2022) | Probably no | Probably yes | Definitely yes | Definitely no | Definitely no | Probably yes | N/A | N/A | Some concerns |
| Marsault (2021) | Definitely no | Probably no | Probably no | Definitely no | Definitely no | Probably yes | Probably yes | No information | High |
| Namburar (2021) | Probably no | Probably yes | Definitely yes | Definitely no | Definitely no | Probably yes | N/A | N/A | Some concerns |

### Table 4: Table of quality assessment for systematic reviews of RCTs/LCAs and observational studies

(Based on AMSTAR checklist (Shea et al.; 2007, BMC Methodol 7: 10; doi:10.1186/1471-2288-7-10) and PRISMA checklist (Moher et al 2009, PLoS Med 6: e1000097; doi:10.1371/journal.pmed1000097))

| Study reference | Appropriate and clearly focused question? | Comprehensive and systematic literature search? | Description of included and excluded studies? | Description of relevant characteristics of included studies? | Appropriate adjustment for potential confounders in observational studies? | Assessment of scientific quality of included studies? | Enough similarities between studies to make combining them reasonable? | Potential risk of publication bias taken into account? | Potential conflicts of interest reported? |
| --- | --- | --- | --- | --- | --- | --- | --- | --- | --- |
| Papadopoulou et al. (2022) | Yes appropriate, but a rather broad question. Study aims to review the MIS literature with the puropose of investigating the carbon footprint, material footprint related to waste or other environmental impact of MIS in the OR. | Yes, according to PRISMA and Cochrane guidelines. Databases include Medline, Embase and Web of Science. | Yes, clearly described in text. PRISMA flow diagram used for overview of included studies. | Yes, clearly described in text. | N/A | LCAs were evaluated using modified criteria from Drew et al. and pre-post non-controlled studies by using the Cochrane Risk of Bias in Non-Randomized Studies of Interventions (ROBINS-I) tool. For observational studies with environmental outcomes a tool created by Rizan et al. was used. | No, difficult to combine these LCAs e.g. because of different system boundaries, conversion factors etc. | Not mentioned in text. | Yes |
| Drew et al. (2021) | Yes appropriate, but a rather broad question. To assess the evidence in surgical and anaesthetic care. | This review is guided by the Standardized Technique for Assessing and Reporting Reviews of Life Cycle Assessment Data (STARR-LCA), a PRISMA-based framework. The search is clearly comprehensive and systematic and databases used are: Scopus, Pubmed and Embase. | Clearly described in text. | Yes, clearly described in text. | N/A | Using a criticial appraisel based on Weidema's guidelines for critical review of LCA. | No, difficult to combine these LCAs e.g. because of different system boundaries, conversion factors etc. | Not mentioned in text. | Yes |
| Keil et al. (2022) | Yes appropriate, but a rather broad question. The goal of the study is to examine the changed environmental impact when single-use healthcare products are replaced by reusables. | Yes, according to PRISMA guidelines. Databases include Pubmed, ProQuest and Web of Science. | Clearly described in text. | Yes, clearly described in text. | N/A | Checklist developed and used where only transparency of the studies has been assessed. | No, difficult to combine these LCAs e.g. because of different system boundaries, conversion factors etc. | Not mentioned in text. | Yes |
| Nowack (2012) | Clearly focused question.  How can environmental aspects be derived from life cycle management (LCM) Instruments for procurement decisions regarding low-value products such as operating room textiles (O.R. textiles)? | PICO is not clearly stated, only keywords and databases. Databases do not include Medline or EMBASE. | Included and excluded studies are described with reason. | Characteristics of the studies are relevant to the search. | N/A | Yes, studies have been critically evaluated for sound methodology, verifiability, completeness, and actuality. | No | No | No |

### Table 5a: Critical appraisal all studies (Drew et al. 2021)

| Appraisal criteria | Indicator(s) | Key effect modifiers | Almutairi et al., 2022 | Boberg et al., 2022 | Boucheron et al., 2022 | Davis et al., 2018 | Donahue et al., 2020 | Eckelman et al., 2012 | Friedericy et al., 2022 | Grimmond et al., 2012 | Grimmond et al. 2021 | Hicks et al 2016 | Hogan et al. 2022 |
| --- | --- | --- | --- | --- | --- | --- | --- | --- | --- | --- | --- | --- | --- |
| Phase 1: Goal & Scope (13 points) |  |  |  |  |  |  |  |  |  |  |  |  |  |
| Study goal is clearly stated, including the study's rationale (1), intended application (1), and intended audience (1) | Transparency |  | 1 | 3 | 3 | 2 | 3 | 3 | 3 | 2 | 2 | 2 | 2 |
| Lifecycle assessment method is clearly stated (1) | Transparency | Process-based life-cycle assessment, which is well suited to product-level analysis, may underestimate environmental impacts (i.e. from truncation error); economic input-output lifecycle assessment (EIO-LCA), which uses aggregate data and is well-suited to sector-level analysis, may overestimate environmental impacts | 1 | 1 | 0 | 0 | 0 | 1 | 1 | 1 | 1 | 1 | 0 |
| Functional unit is clearly defined and measurable (1), justified (1), and consistent with the study's intended application (1) | Consistency |  | 2 | 3 | 0 | 3 | 2 | 3 | 3 | 1 | 2 | 2 | 0 |
| The system to be studied is adequately described with clearly stated system boundaries (1), lifecycle stages (1), and appropriate justification of any omitted stages (1) | Transparency; Bias | Assessments with narrow system boundaries that exclude a number of lifecycle stages are prone to underestimating life-cycle environmental impacts | 2 | 3 | 0 | 2 | 3 | 3 | 2 | 2 | 2 | 2 | 1 |
| The system covers production (1), use/reuse (1) and disposal (1) of materials and energy (half mark if only for energy and vice versa) | Internal Validity, Completeness |  | 3 | 3 | 0.5 | 3 | 3 | 3 | 3 | 3 | 3 | 3 | 1 |
| Phase 2: Inventory analysis (7 points) |  |  |  |  |  |  |  |  |  |  |  |  |  |
| The data collection process is clearly explained, including the source(s) of foreground material weights and energy values (1); the source(s) of reference data (e.g. inventory database; 1); and what data are included (e.g. production and disposal of unit processes; 1) | Transparency, Internal Validity |  | 2 | 3 | 3 | 2 | 3 | 3 | 2 | 3 | 3 | 3 | 3 |
| Representativeness of the data is discussed (1), differences in electricity generating mix are accounted for (1), and the potential significance of exclusions or assumptions is addressed (1) | Internal validity; External validity |  | 0 | 3 | 0 | 0 | 2 | 1 | 3 | 2 | 1 | 1 | 0 |
| Allocation procedures, where necessary, are described and appropriately justified (1; mark given if no allocation used) | Transparency; Bias |  | 1 | 1 | 1 | 1 | 1 | 1 | 1 | 1 | 1 | 1 | 1 |
| Phase 3: Impact assessment (6 points) |  |  |  |  |  |  |  |  |  |  |  |  |  |
| Impact categories (1), characterization method (1), and software used (1) are documented transparently | Transparency |  | 3 | 3 | 0 | 1 | 3 | 3 | 3 | 2 | 2 | 3 | 1 |
| Results are clearly reported in the context of the functional unit (1) (0.5 if graphically, 0 if only normalized results reported) | Consistency; Transparency |  | 0 | 1 | 0 | 1 | 1 | 1 | 1 | 1 | 1 | 1 | 0 |
| A contribution analysis is performed and clearly reported (1), and hotspots are identified (1) |  |  | 2 | 2 | 0 | 2 | 2 | 1 | 2 | 2 | 2 | 2 | 1 |
| Phase 4: Interpretation (9 points) |  |  |  |  |  |  |  |  |  |  |  |  |  |
| Conclusions are consistent with the goal and scope (1) and supported by the impact assessment results (1) | Internal validity; Consistency |  | 2 | 2 | 1 | 2 | 2 | 2 | 2 | 2 | 2 | 2 | 1 |
| Results are contextualized through the use of sensitivity analysis (1) and uncertainty analysis (1) | Internal validity |  | 0 | 2 | 0 | 0 | 1 | 1 | 1 | 1 | 1 | 2 | 0 |
| Limitations are adequately discussed (1), and the potential impact of omissions or assumptions on the study's outcomes are described (1) | Bias |  | 2 | 2 | 1 | 0 | 1 | 1 | 2 | 0 | 1 | 1 | 0 |
| The assessment has been critically appraised (i.e. peer review if journal article or independent, external critical review if report/thesis; 1) | Bias |  | 1 | 1 | 1 | 1 | 1 | 1 | 1 | 1 | 1 | 1 | 1 |
| Source(s) of funding and any potential conflict(s) of interest are disclosed (1), and are unlikely to be a source of bias (1) | Bias |  | 1 | 2 | 2 | 1 | 1 | 2 | 0 | 1 | 1 | 1 | 2 |
|  |  | Total (/35) | 23 | 35 | 12 | 21 | 29 | 30 | 30 | 25 | 26 | 28 | 14 |
|  |  | Percentage score | 66% | 100% | 34% | 60% | 83% | 86% | 86% | 71% | 74% | 80% | 40% |

### Table 5b: Critical appraisal all studies (Drew et al. 2021)

| Appraisal criteria | Indicator(s) | Key effect modifiers | Hu et al., 2021 | Ibbotson et al., 2013 | Kemble et al., 2022 | Le et al., 2022 | Leiden et al., 2020 | McGain et al., 2010 | McGain et al., 2012 | McGain et al., 2017 | McGain et al., 2021 | McPherson et al., 2019 | Morris et al., 2022 |
| --- | --- | --- | --- | --- | --- | --- | --- | --- | --- | --- | --- | --- | --- |
| Phase 1: Goal & Scope (13 points) |  |  |  |  |  |  |  |  |  |  |  |  |  |
| Study goal is clearly stated, including the study's rationale (1), intended application (1), and intended audience (1) | Transparency |  | 3 | 3 | 3 | 3 | 2 | 2 | 2 | 1 | 3 | 2 | 2 |
| Lifecycle assessment method is clearly stated (1) | Transparency | Process-based life-cycle assessment, which is well suited to product-level analysis, may underestimate environmental impacts (i.e. from truncation error); economic input-output lifecycle assessment (EIO-LCA), which uses aggregate data and is well-suited to sector-level analysis, may overestimate environmental impacts | 1 | 1 | 1 | 1 | 1 | 1 | 1 | 1 | 1 | 1 | 1 |
| Functional unit is clearly defined and measurable (1), justified (1), and consistent with the study's intended application (1) | Consistency |  | 3 | 3 | 0 | 2 | 3 | 1 | 1 | 0 | 2 | 2 | 2 |
| The system to be studied is adequately described with clearly stated system boundaries (1), lifecycle stages (1), and appropriate justification of any omitted stages (1) | Transparency; Bias | Assessments with narrow system boundaries that exclude a number of lifecycle stages are prone to underestimating life-cycle environmental impacts | 3 | 2 | 2 | 3 | 2 | 3 | 2 | 3 | 3 | 2 | 3 |
| The system covers production (1), use/reuse (1) and disposal (1) of materials and energy (half mark if only for energy and vice versa) | Internal Validity, Completeness |  | 2 | 3 | 2 | 3 | 3 | 3 | 3 | 3 | 3 | 3 | 3 |
| Phase 2: Inventory analysis (7 points) |  |  |  |  |  |  |  |  |  |  |  |  |  |
| The data collection process is clearly explained, including the source(s) of foreground material weights and energy values (1); the source(s) of reference data (e.g. inventory database; 1); and what data are included (e.g. production and disposal of unit processes; 1) | Transparency, Internal Validity |  | 3 | 3 | 2 | 3 | 2 | 3 | 3 | 3 | 3 | 3 | 3 |
| Representativeness of the data is discussed (1), differences in electricity generating mix are accounted for (1), and the potential significance of exclusions or assumptions is addressed (1) | Internal validity; External validity |  | 2 | 1 | 2 | 0 | 1 | 2 | 2 | 2 | 3 | 2 | 1 |
| Allocation procedures, where necessary, are described and appropriately justified (1; mark given if no allocation used) | Transparency; Bias |  | 1 | 1 | 1 | 1 | 1 | 1 | 1 | 1 | 1 | 1 | 1 |
| Phase 3: Impact assessment (6 points) |  |  |  |  |  |  |  |  |  |  |  |  |  |
| Impact categories (1), characterization method (1), and software used (1) are documented transparently | Transparency |  | 3 | 3 | 1 | 3 | 3 | 2 | 2 | 2 | 2 | 2 | 3 |
| Results are clearly reported in the context of the functional unit (1) (0.5 if graphically, 0 if only normalized results reported) | Consistency; Transparency |  | 0,5 | 0,5 | 0 | 1 | 0 | 1 | 1 | 1 | 1 | 1 | 0.5 |
| A contribution analysis is performed and clearly reported (1), and hotspots are identified (1) |  |  | 2 | 1 | 2 | 2 | 2 | 2 | 2 | 1 | 2 | 2 | 2 |
| Phase 4: Interpretation (9 points) |  |  |  |  |  |  |  |  |  |  |  |  |  |
| Conclusions are consistent with the goal and scope (1) and supported by the impact assessment results (1) | Internal validity; Consistency |  | 2 | 2 | 2 | 2 | 2 | 2 | 2 | 1 | 2 | 2 | 2 |
| Results are contextualized through the use of sensitivity analysis (1) and uncertainty analysis (1) | Internal validity |  | 1 | 1 | 0 | 1 | 1 | 1 | 2 | 1 | 2 | 1 | 2 |
| Limitations are adequately discussed (1), and the potential impact of omissions or assumptions on the study's outcomes are described (1) | Bias |  | 1 | 1 | 2 | 2 | 1 | 2 | 2 | 0 | 1 | 1 | 1 |
| The assessment has been critically appraised (i.e. peer review if journal article or independent, external critical review if report/thesis; 1) | Bias |  | 1 | 1 | 1 | 1 | 1 | 1 | 1 | 1 | 1 | 1 | 1 |
| Source(s) of funding and any potential conflict(s) of interest are disclosed (1), and are unlikely to be a source of bias (1) | Bias |  | 1 | 0 | 2 | 1 | 0,5 | 1 | 2 | 2 | 2 | 1 | 2 |
|  |  | Total (/35) | 29,5 | 26,5 | 23 | 29 | 25,5 | 28 | 29 | 23 | 32 | 27 | 29 |
|  |  | Percentage score | 84% | 76% | 66% | 83% | 73% | 80% | 83% | 66% | 91% | 77% | 83% |

### Table 5c: Critical appraisal all studies (Drew et al. 2021)

| Appraisal criteria | Indicator(s) | Key effect modifiers | Namburar et al., 2012 | Power et al., 2012 | Rizan et al., 2021 | Sanchez 2020 | Sherman et al., 2012 | Sherman et al., 2018 | Sorensen et al., 2022 | Thiel et al., 2015 | Thiel et al., 2018 | Unger et al., 2014 |
| --- | --- | --- | --- | --- | --- | --- | --- | --- | --- | --- | --- | --- |
| Phase 1: Goal & Scope (13 points) |  |  |  |  |  |  |  |  |  |  |  |  |
| Study goal is clearly stated, including the study's rationale (1), intended application (1), and intended audience (1) | Transparency |  | 3 | 3 | 3 | 3 | 3 | 3 | 3 | 3 | 3 | 3 |
| Lifecycle assessment method is clearly stated (1) | Transparency | Process-based life-cycle assessment, which is well suited to product-level analysis, may underestimate environmental impacts (i.e. from truncation error); economic input-output lifecycle assessment (EIO-LCA), which uses aggregate data and is well-suited to sector-level analysis, may overestimate environmental impacts | 0 | 1 | 1 | 1 | 0 | 1 | 1 | 1 | 1 | 1 |
| Functional unit is clearly defined and measurable (1), justified (1), and consistent with the study's intended application (1) | Consistency |  | 0 | 0 | 2 | 2 | 3 | 3 | 2 | 3 | 3 | 2 |
| The system to be studied is adequately described with clearly stated system boundaries (1), lifecycle stages (1), and appropriate justification of any omitted stages (1) | Transparency; Bias | Assessments with narrow system boundaries that exclude a number of lifecycle stages are prone to underestimating life-cycle environmental impacts | 2 | 1 | 3 | 2 | 3 | 2 | 3 | 2 | 2 | 3 |
| The system covers production (1), use/reuse (1) and disposal (1) of materials and energy (half mark if only for energy and vice versa) | Internal Validity, Completeness |  | 0.5 | 2 | 3 | 3 | 3 | 3 | 3 | 3 | 3 | 3 |
| Phase 2: Inventory analysis (7 points) |  |  |  |  |  |  |  |  |  |  |  |  |
| The data collection process is clearly explained, including the source(s) of foreground material weights and energy values (1); the source(s) of reference data (e.g. inventory database; 1); and what data are included (e.g. production and disposal of unit processes; 1) | Transparency, Internal Validity |  | 3 | 3 | 3 | 3 | 3 | 3 | 2 | 3 | 3 | 3 |
| Representativeness of the data is discussed (1), differences in electricity generating mix are accounted for (1), and the potential significance of exclusions or assumptions is addressed (1) | Internal validity; External validity |  | 0 | 0 | 0 | 1 | 2 | 2 | 1 | 2 | 2 | 2 |
| Allocation procedures, where necessary, are described and appropriately justified (1; mark given if no allocation used) | Transparency; Bias |  | 1 | 1 | 1 | 1 | 1 | 1 | 1 | 1 | 1 | 1 |
| Phase 3: Impact assessment (6 points) |  |  |  |  |  |  |  |  |  |  |  |  |
| Impact categories (1), characterization method (1), and software used (1) are documented transparently | Transparency |  | 0 | 1 | 3 | 3 | 3 | 3 | 3 | 2 | 2 | 3 |
| Results are clearly reported in the context of the functional unit (1) (0.5 if graphically, 0 if only normalized results reported) | Consistency; Transparency |  | 0 | 1 | 1 | 1 | 1 | 1 | 1 | 0 | 1 | 0 |
| A contribution analysis is performed and clearly reported (1), and hotspots are identified (1) |  |  | 1 | 2 | 2 | 2 | 2 | 2 | 2 | 2 | 2 | 2 |
| Phase 4: Interpretation (9 points) |  |  |  |  |  |  |  |  |  |  |  |  |
| Conclusions are consistent with the goal and scope (1) and supported by the impact assessment results (1) | Internal validity; Consistency |  | 1 | 0 | 2 | 2 | 2 | 2 | 2 | 2 | 2 | 2 |
| Results are contextualized through the use of sensitivity analysis (1) and uncertainty analysis (1) | Internal validity |  | 0 | 0 | 0 | 1 | 0 | 1 | 0 | 1 | 0 | 0 |
| Limitations are adequately discussed (1), and the potential impact of omissions or assumptions on the study's outcomes are described (1) | Bias |  | 1 | 1 | 2 | 1 | 1 | 0 | 1 | 0 | 1 | 2 |
| The assessment has been critically appraised (i.e. peer review if journal article or independent, external critical review if report/thesis; 1) | Bias |  | 1 | 1 | 1 | 1 | 1 | 1 | 1 | 1 | 1 | 1 |
| Source(s) of funding and any potential conflict(s) of interest are disclosed (1), and are unlikely to be a source of bias (1) | Bias |  | 1 | 2 | 2 | 2 | 1 | 2 | 1 | 2 | 0 | 0 |
|  |  | Total (/35) | 14 | 19 | 29 | 29 | 29 | 30 | 27 | 28 | 27 | 28 |
|  |  | Percentage score | 40% | 54% | 83% | 83% | 83% | 86% | 77% | 80% | 77% | 80% |

### Table 5d: Critical appraisal all studies (Drew et al. 2021)

| Appraisal criteria | Indicator(s) | Key effect modifiers | Vozzola et al., 2018 | Vozzola et al., 2020 | Wang et al. 2022 | Woods et al., 2015 |
| --- | --- | --- | --- | --- | --- | --- |
| Phase 1: Goal & Scope (13 points) |  |  |  |  |  |  |
| Study goal is clearly stated, including the study's rationale (1), intended application (1), and intended audience (1) | Transparency |  | 3 | 3 | 3 | 3 |
| Lifecycle assessment method is clearly stated (1) | Transparency | Process-based life-cycle assessment, which is well suited to product-level analysis, may underestimate environmental impacts (i.e. from truncation error); economic input-output lifecycle assessment (EIO-LCA), which uses aggregate data and is well-suited to sector-level analysis, may overestimate environmental impacts | 1 | 1 | 0 | 1 |
| Functional unit is clearly defined and measurable (1), justified (1), and consistent with the study's intended application (1) | Consistency |  | 2 | 2 | 0 | 0 |
| The system to be studied is adequately described with clearly stated system boundaries (1), lifecycle stages (1), and appropriate justification of any omitted stages (1) | Transparency; Bias | Assessments with narrow system boundaries that exclude a number of lifecycle stages are prone to underestimating life-cycle environmental impacts | 2 | 2 | 3 | 1 |
| The system covers production (1), use/reuse (1) and disposal (1) of materials and energy (half mark if only for energy and vice versa) | Internal Validity, Completeness |  | 3 | 3 | 0.5 | 2 |
| Phase 2: Inventory analysis (7 points) |  |  |  |  |  |  |
| The data collection process is clearly explained, including the source(s) of foreground material weights and energy values (1); the source(s) of reference data (e.g. inventory database; 1); and what data are included (e.g. production and disposal of unit processes; 1) | Transparency, Internal Validity |  | 3 | 3 | 0 | 3 |
| Representativeness of the data is discussed (1), differences in electricity generating mix are accounted for (1), and the potential significance of exclusions or assumptions is addressed (1) | Internal validity; External validity |  | 0 | 2 | 0 | 0 |
| Allocation procedures, where necessary, are described and appropriately justified (1; mark given if no allocation used) | Transparency; Bias |  | 1 | 1 | 1 | 1 |
| Phase 3: Impact assessment (6 points) |  |  |  |  |  |  |
| Impact categories (1), characterization method (1), and software used (1) are documented transparently | Transparency |  | 1 | 1 | 1 | 1 |
| Results are clearly reported in the context of the functional unit (1) (0.5 if graphically, 0 if only normalized results reported) | Consistency; Transparency |  | 1 | 1 | 0 | 1 |
| A contribution analysis is performed and clearly reported (1), and hotspots are identified (1) |  |  | 2 | 2 | 0 | 2 |
| Phase 4: Interpretation (9 points) |  |  |  |  |  |  |
| Conclusions are consistent with the goal and scope (1) and supported by the impact assessment results (1) | Internal validity; Consistency |  | 2 | 2 | 1 | 2 |
| Results are contextualized through the use of sensitivity analysis (1) and uncertainty analysis (1) | Internal validity |  | 1 | 1 | 0 | 0 |
| Limitations are adequately discussed (1), and the potential impact of omissions or assumptions on the study's outcomes are described (1) | Bias |  | 0 | 2 | 1 | 1 |
| The assessment has been critically appraised (i.e. peer review if journal article or independent, external critical review if report/thesis; 1) | Bias |  | 1 | 1 | 1 | 1 |
| Source(s) of funding and any potential conflict(s) of interest are disclosed (1), and are unlikely to be a source of bias (1) | Bias |  | 0,5 | 1 | 0 | 1 |
|  |  | Total (/35) | 23,5 | 28 | 11 | 20 |
|  |  | Percentage score | 67% | 80% | 31% | 57% |

### Table 6: GRADE assessment for LCA studies

| **GRADE for LCA's** | |  |  |  |  |  |  |  |
| --- | --- | --- | --- | --- | --- | --- | --- | --- |
| 1. Initial level of certainty | | 2. Factors that an influence the quality of the evidence | | | 3. Final level of certainty in an estimate of effect | | |  |
| Study design | Initial certainty | Lower if |  | Higher if |  |  |  |  |
| LCAs or CFA using LCA-methods | High | Limitations in study design or execution (risk of bias) ↓ 1 or 2 levels | | Large magnitude of effect ↑ 1 or 2 levels | High | | | |
|  |  | Inconsistency of results ↓ 1 or 2 levels | | All plausible confounding would reduce the demonstrated effect or increase the effect if no effect was observed ↑ 1 or 2 levels | Moderate | | | |
| Observational studies | Low | Indirectness of evidence ↓ 1 or 2 levels | | Dose reponse gradient ↑ 1 or 2 levels | Low | | | |
|  |  | Imprecision ↓ 1 or 2 levels | |  | Very low | | | |
|  |  | Publication bias ↓ 1 or 2 levels | |  |  |  |  |  |

### Table 7: Topic 1. Critical appraisal points per study

| **Study** | **Phase** | **Points given** |  |  |
| --- | --- | --- | --- | --- |
| **Power et al. (2012)** | Phase 1 |  |  |  |
| Transparency |  | Study's rationale clearly stated (1) | Intended Application clearly stated (1) | Intended audience clearly stated (1) |
| Transparency |  | LCA method stated (1) |  |  |
| Consistency |  | FU defined and measurable (1) | FU justified (1) | FU consistent (1) |
| Transparency; Bias |  | System boundaries clearly stated (1) | Lifecycle stages clearly stated (1) | Appropriate justification of any omitted stages (1) |
| Internal Validity, Completeness | *Half mark if only for energy and vice versa* | System covers: production (1) of materials and energy | Use/reuse (1) of materials and energy | Disposal (1) of materials and energy |
|  | Phase 2 |  |  |  |
| Transparency, Internal Validity |  | Clear data collection (1) | Sources of data described (1) | Included data described (1) |
| Internal validity; External validity |  | Representativeness of the data is discussed (1) | Differences in electricity generating mix are accounted for (1) | Potential significance of exclusions or assumptions is addressed (1) |
| Transparency; Bias | *One mark if no allocation used* | Allocation if necessary described and justified (1) |  |  |
|  | Phase 3 |  |  |  |
| Transparency |  | Documented transparently: Impact categories (1) | Characterization method (1) | Software used (1) |
| Consistency; Transparency | *(0.5 if graphically, 0 if only normalized results reported)* | Results are clearly reported in the context of the functional unit (1) |  |  |
|  |  | A contribution analysis is performed and clearly reported (1) | Hotspots are identified (1) |  |
|  | Phase 4 |  |  |  |
| Internal validity; Consistency |  | Conclusions are consistent with the goal and scope (1) | Conclusions are supported by the impact assessment results (1) |  |
| Internal validity |  | Results are contextualized through the use of sensitivity analysis (1) | Results are contextualized through the use of uncertainty analysis (1) |  |
| Bias |  | Limitations are adequately discussed (1) | The potential impact of omissions or assumptions on the study's outcomes are described (1) |  |
| Bias |  | The assessment has been critically appraised (i.e. peer review if journal article or independent, external critical review if report/thesis; 1) |  |  |
| Bias |  | Source(s) of funding and any potential conflict(s) of interest are disclosed (1) | Sources and potential conflicts are unlikely to be a source of bias (1) |  |
|  |  |  |  |  |
|  |  |  |  |  |
| **Study** | **Phase** | **Points given** |  |  |
| **Thiel et al. (2015)** | Phase 1 |  |  |  |
| Transparency |  | Study's rationale clearly stated (1) | Intended Application clearly stated (1) | Intended audience clearly stated (1) |
| Transparency |  | LCA method stated (1) |  |  |
| Consistency |  | FU defined and measurable (1) | FU justified (1) | FU consistent (1) |
| Transparency; Bias |  | System boundaries clearly stated (1) | Lifecycle stages clearly stated (1) | Appropriate justification of any omitted stages (1) |
| Internal Validity, Completeness | *Half mark if only for energy and vice versa* | System covers: production (1) of materials and energy | Use/reuse (1) of materials and energy | Disposal (1) of materials and energy |
|  | Phase 2 |  |  |  |
| Transparency, Internal Validity |  | Clear data collection (1) | Sources of data described (1) | Included data described (1) |
| Internal validity; External validity |  | Representativeness of the data is discussed (1) | Differences in electricity generating mix are accounted for (1) | Potential significance of exclusions or assumptions is addressed (1) |
| Transparency; Bias | *One mark if no allocation used* | Allocation if necessary described and justified (1) |  |  |
|  | Phase 3 |  |  |  |
| Transparency |  | Documented transparently: Impact categories (1) | Characterization method (1) | Software used (1) |
| Consistency; Transparency | *(0.5 if graphically, 0 if only normalized results reported)* | Results are clearly reported in the context of the functional unit (1) |  |  |
|  |  | A contribution analysis is performed and clearly reported (1) | Hotspots are identified (1) |  |
|  | Phase 4 |  |  |  |
| Internal validity; Consistency |  | Conclusions are consistent with the goal and scope (1) | Conclusions are supported by the impact assessment results (1) |  |
| Internal validity |  | Results are contextualized through the use of sensitivity analysis (1) | Results are contextualized through the use of uncertainty analysis (1) |  |
| Bias |  | Limitations are adequately discussed (1) | The potential impact of omissions or assumptions on the study's outcomes are described (1) |  |
| Bias |  | The assessment has been critically appraised (i.e. peer review if journal article or independent, external critical review if report/thesis; 1) |  |  |
| Bias |  | Source(s) of funding and any potential conflict(s) of interest are disclosed (1) | Sources and potential conflicts are unlikely to be a source of bias (1) |  |
|  |  |  |  |  |
|  |  |  |  |  |
| **Study** | **Phase** | **Points given** |  |  |
| **Woods et al. (2015)** | Phase 1 |  |  |  |
| Transparency |  | Study's rationale clearly stated (1) | Intended Application clearly stated (1) | Intended audience clearly stated (1) |
| Transparency |  | LCA method stated (1) |  |  |
| Consistency |  | FU defined and measurable (1) | FU justified (1) | FU consistent (1) |
| Transparency; Bias |  | System boundaries clearly stated (1) | Lifecycle stages clearly stated (1) | Appropriate justification of any omitted stages (1) |
| Internal Validity, Completeness | *Half mark if only for energy and vice versa* | System covers: production (1) of materials and energy | Use/reuse (1) of materials and energy | Disposal (1) of materials and energy |
|  | Phase 2 |  |  |  |
| Transparency, Internal Validity |  | Clear data collection (1) | Sources of data described (1) | Included data described (1) |
| Internal validity; External validity |  | Representativeness of the data is discussed (1) | Differences in electricity generating mix are accounted for (1) | Potential significance of exclusions or assumptions is addressed (1) |
| Transparency; Bias | *One mark if no allocation used* | Allocation if necessary described and justified (1) |  |  |
|  | Phase 3 |  |  |  |
| Transparency |  | Documented transparently: Impact categories (1) | Characterization method (1) | Software used (1) |
| Consistency; Transparency | *(0.5 if graphically, 0 if only normalized results reported)* | Results are clearly reported in the context of the functional unit (1) |  |  |
|  |  | A contribution analysis is performed and clearly reported (1) | Hotspots are identified (1) |  |
|  | Phase 4 |  |  |  |
| Internal validity; Consistency |  | Conclusions are consistent with the goal and scope (1) | Conclusions are supported by the impact assessment results (1) |  |
| Internal validity |  | Results are contextualized through the use of sensitivity analysis (1) | Results are contextualized through the use of uncertainty analysis (1) |  |
| Bias |  | Limitations are adequately discussed (1) | The potential impact of omissions or assumptions on the study's outcomes are described (1) |  |
| Bias |  | The assessment has been critically appraised (i.e. peer review if journal article or independent, external critical review if report/thesis; 1) |  |  |
| Bias |  | Source(s) of funding and any potential conflict(s) of interest are disclosed (1) | Sources and potential conflicts are unlikely to be a source of bias (1) |  |
|  |  |  |  |  |

### Table 8: Topic 1. GRADE assessment per included outcome

| **Outcome** |  |  |  |  |  |  |  |  |  |  |  |  |
| --- | --- | --- | --- | --- | --- | --- | --- | --- | --- | --- | --- | --- |
| Climate change |  |  | **Missing according to critical appraisal** | |  |  |  |  |  |  |  |  |
| *Power (2012), Thiel (2015), Woods (2015)* | | | Phase 1 (bias) | Consistency - FU not clearly defined and measurable, FU not justified and/or FU not consistent | | | | | |  |  |  |
|  |  |  | Phase 1 (bias) | Transparency, bias - System boundaries and/or life cycle stages not clearly stated and/or no appropriate justification of any omitted stages | | | | | | | | |
|  |  |  | Phase 2 (indi) | Internal/external validity - Representativeness of data is not discussed and/or differences energy mix are not accounted for and/or potential significance of exclusions or assumptions is not addressed | | | | | | | | |
|  |  |  | Phase 3 (bias) | Transparency - Impact categories not documented transparently and/or missing information on characterization method and/or software | | | | | | | | |
|  |  |  | Phase 4 (bias) | Internal validity - Impacts not reported through sensitivity and/or uncertainty analysis | | | | | |  |  |  |
|  |  |  | Phase 4 (bias) | Bias - Limitations not adequately discussed and/or potential impact of omissions or assumptions on the study's outcomes are not described and/or study not critically appraised and/or no disclosed funding source/conflicts of interest and/or funding and conflicts of interest likely to be a source of bias | | | | | | | | |
|  |  |  |  |  |  |  |  |  |  |  |  |  |
| Waste |  |  | **Missing according to critical appraisal** | | |  |  |  |  |  |  |  |
| *Power (2012), Thiel (2015), Woods (2015)* | | | Phase 1 (bias) | Consistency - FU not clearly defined and measurable, FU not justified and/or FU not consistent | | | | | |  |  |  |
|  |  |  | Phase 1 (bias) | Transparency, bias - System boundaries and/or life cycle stages not clearly stated and/or no appropriate justification of any omitted stages | | | | | | | | |
|  |  |  | Phase 1 (bias) | Internal validity/completeness - Missing system coverage of production and/or use/reuse and/or disposal of materials and/or energy | | | | | | | | |
|  |  |  | Phase 2 (indi) | Internal/external validity - Representativeness of data is not discussed and/or differences energy mix are not accounted for and/or potential significance of exclusions or assumptions is not addressed | | | | | | | | |
|  |  |  | Phase 3 (bias) | Transparency - Impact categories not documented transparently and/or missing information on characterization method and/or software | | | | | | | | |
|  |  |  | Phase 4 (bias) | Internal validity - Impacts not reported through sensitivity and/or uncertainty analysis | | | | | |  |  |  |
|  |  |  | Phase 4 (bias) | Bias - Limitations not adequately discussed and/or potential impact of omissions or assumptions on the study's outcomes are not described and/or study not critically appraised and/or no disclosed funding source/conflicts of interest and/or funding and conflicts of interest likely to be a source of bias | | | | | | | | |
|  |  |  |  |  |  |  |  |  |  |  |  |  |
| Acidification |  |  | **Missing according to critical appraisal** | | |  |  |  |  |  |  |  |
| *Thiel (2015)* |  |  | Phase 1 (bias) | Transparency, bias - System boundaries and/or life cycle stages not clearly stated and/or no appropriate justification of any omitted stages | | | | | | | |  |
|  |  |  | Phase 2 (indi) | Internal/external validity - Representativeness of data is not discussed and/or differences energy mix are not accounted for and/or potential significance of exclusions or assumptions is not addressed | | | | | | | | |
|  |  |  | Phase 3 (bias) | Consistency, transparency - Results are not clearly reported in context of the functional unit and/or no contribution analysis and/or hotspots not identified | | | | | | | | |
|  |  |  | Phase 4 (bias) | Internal validity - Impacts not reported through sensitivity and/or uncertainty analysis | | | | | |  |  |  |
|  |  |  | Phase 4 (bias) | Bias - Limitations not adequately discussed and/or potential impact of omissions or assumptions on the study's outcomes are not described and/or study not critically appraised and/or no disclosed funding source/conflicts of interest and/or funding and conflicts of interest likely to be a source of bias | | | | | | | | |
|  |  |  | (indirectness) | One study |  |  |  |  |  |  |  |  |
|  |  |  |  |  |  |  |  |  |  |  |  |  |
| Eutrophication |  |  | **Missing according to critical appraisal** | | |  |  |  |  |  |  |  |
| *Thiel (2015)* |  |  | Phase 1 (bias) | Transparency, bias - System boundaries and/or life cycle stages not clearly stated and/or no appropriate justification of any omitted stages | | | | | | | |  |
|  |  |  | Phase 2 (indi) | Internal/external validity - Representativeness of data is not discussed and/or differences energy mix are not accounted for and/or potential significance of exclusions or assumptions is not addressed | | | | | | | | |
|  |  |  | Phase 3 (bias) | Consistency, transparency - Results are not clearly reported in context of the functional unit and/or no contribution analysis and/or hotspots not identified | | | | | | | | |
|  |  |  | Phase 4 (bias) | Internal validity - Impacts not reported through sensitivity and/or uncertainty analysis | | | | | |  |  |  |
|  |  |  | Phase 4 (bias) | Bias - Limitations not adequately discussed and/or potential impact of omissions or assumptions on the study's outcomes are not described and/or study not critically appraised and/or no disclosed funding source/conflicts of interest and/or funding and conflicts of interest likely to be a source of bias | | | | | | | | |
|  |  |  | (indirectness) | One study |  |  |  |  |  |  |  |  |
|  |  |  |  |  |  |  |  |  |  |  |  |  |
| Human toxicity |  |  | **Missing according to critical appraisal** | | |  |  |  |  |  |  |  |
| *Thiel (2015)* |  |  | Phase 1 (bias) | Transparency, bias - System boundaries and/or life cycle stages not clearly stated and/or no appropriate justification of any omitted stages | | | | | | | |  |
|  |  |  | Phase 2 (indi) | Internal/external validity - Representativeness of data is not discussed and/or differences energy mix are not accounted for and/or potential significance of exclusions or assumptions is not addressed | | | | | | | | |
|  |  |  | Phase 3 (bias) | Consistency, transparency - Results are not clearly reported in context of the functional unit and/or no contribution analysis and/or hotspots not identified | | | | | | | | |
|  |  |  | Phase 4 (bias) | Internal validity - Impacts not reported through sensitivity and/or uncertainty analysis | | | | | |  |  |  |
|  |  |  | Phase 4 (bias) | Bias - Limitations not adequately discussed and/or potential impact of omissions or assumptions on the study's outcomes are not described and/or study not critically appraised and/or no disclosed funding source/conflicts of interest and/or funding and conflicts of interest likely to be a source of bias | | | | | | | | |
|  |  |  | (indirectness) | One study |  |  |  |  |  |  |  |  |
|  |  |  |  |  |  |  |  |  |  |  |  |  |
| Ecotoxicity |  |  | **Missing according to critical appraisal** | | |  |  |  |  |  |  |  |
| *Thiel (2015)* |  |  | Phase 1 (bias) | Transparency, bias - System boundaries and/or life cycle stages not clearly stated and/or no appropriate justification of any omitted stages | | | | | | | |  |
|  |  |  | Phase 2 (indi) | Internal/external validity - Representativeness of data is not discussed and/or differences energy mix are not accounted for and/or potential significance of exclusions or assumptions is not addressed | | | | | | | | |
|  |  |  | Phase 3 (bias) | Consistency, transparency - Results are not clearly reported in context of the functional unit and/or no contribution analysis and/or hotspots not identified | | | | | | | | |
|  |  |  | Phase 4 (bias) | Internal validity - Impacts not reported through sensitivity and/or uncertainty analysis | | | | | |  |  |  |
|  |  |  | Phase 4 (bias) | Bias - Limitations not adequately discussed and/or potential impact of omissions or assumptions on the study's outcomes are not described and/or study not critically appraised and/or no disclosed funding source/conflicts of interest and/or funding and conflicts of interest likely to be a source of bias | | | | | | | | |
|  |  |  | (indirectness) | One study |  |  |  |  |  |  |  |  |
|  |  |  |  |  |  |  |  |  |  |  |  |  |
| Ozone depletion |  |  | **Missing according to critical appraisal** | | |  |  |  |  |  |  |  |
| *Thiel (2015)* |  |  | Phase 1 (bias) | Transparency, bias - System boundaries and/or life cycle stages not clearly stated and/or no appropriate justification of any omitted stages | | | | | | | |  |
|  |  |  | Phase 2 (indi) | Internal/external validity - Representativeness of data is not discussed and/or differences energy mix are not accounted for and/or potential significance of exclusions or assumptions is not addressed | | | | | | | | |
|  |  |  | Phase 3 (bias) | Consistency, transparency - Results are not clearly reported in context of the functional unit and/or no contribution analysis and/or hotspots not identified | | | | | | | | |
|  |  |  | Phase 4 (bias) | Internal validity - Impacts not reported through sensitivity and/or uncertainty analysis | | | | | |  |  |  |
|  |  |  | Phase 4 (bias) | Bias - Limitations not adequately discussed and/or potential impact of omissions or assumptions on the study's outcomes are not described and/or study not critically appraised and/or no disclosed funding source/conflicts of interest and/or funding and conflicts of interest likely to be a source of bias | | | | | | | | |
|  |  |  | (indirectness) | One study |  |  |  |  |  |  |  |  |

| **Low GRADE** |  |  |  |  |  |  |  |  |  |
| --- | --- | --- | --- | --- | --- | --- | --- | --- | --- |
| Three studies (Power, 2012; Thiel, 2015; Woods, 2015) reported on ‘climate change’. The level of evidence of this outcome measure was downgraded with 2 levels to low due to: Risk of bias (-2; FU not clearly defined and measurable, FU not justified and/or FU not consistent; System boundaries and/or life cycle stages not clearly stated and/or no appropriate justification of any omitted stages; Impact categories not documented transparently and/or missing information on characterization method and/or software; Impacts not reported through sensitivity and/or uncertainty analysis; Limitations not adequately discussed and/or potential impact of omissions or assumptions on the study's outcomes are not described and/or study not critically appraised and/or no disclosed funding source/conflicts of interest and/or funding and conflicts of interest likely to be a source of bias). | | | | | | | | |  |
|  |  |  |  |  |  |  |  |  |  |
|  |  |  |  |  |  |  |  |  |  |
|  |  |  |  |  |  |  |  |  |  |
|  |  |  |  |  |  |  |  |  |  |
|  |  |  |  |  |  |  |  |  |  |
|  |  |  |  |  |  |  |  |  |  |
| **Low GRADE** | |  |  |  |  |  |  |  |  |
| Three studies (Power, 2012; Woods, 2015; Thiel, 2015) reported on ‘waste’. The level of evidence of this outcome measure was downgraded with 2 levels to low due to: Risk of bias (-2; FU not clearly defined and measurable, FU not justified and/or FU not consistent; System boundaries and/or life cycle stages not clearly stated and/or no appropriate justification of any omitted stages; Missing system coverage of production and/or use/reuse and/or disposal of materials and/or energy; Impact categories not documented transparently and/or missing information on characterization method and/or software; Impacts not reported through sensitivity and/or uncertainty analysis; Limitations not adequately discussed and/or potential impact of omissions or assumptions on the study's outcomes are not described and/or study not critically appraised and/or no disclosed funding source/conflicts of interest and/or funding and conflicts of interest likely to be a source of bias) | | | | | | | | |  |
|  |  |  |  |  |  |  |  |  |  |
|  |  |  |  |  |  |  |  |  |  |
|  |  |  |  |  |  |  |  |  |  |
|  |  |  |  |  |  |  |  |  |  |
|  |  |  |  |  |  |  |  |  |  |
|  |  |  |  |  |  |  |  |  |  |
|  |  |  |  |  |  |  |  |  |  |
| **Very low GRADE** | |  |  |  |  |  |  |  |  |
| One study (Thiel, 2015) reported on ‘acidification’. The level of evidence of this outcome measure was downgraded with 4 levels to very low due to: Risk of bias (-2; System boundaries and/or life cycle stages not clearly stated and/or no appropriate justification of any omitted stages; Results are not clearly reported in context of the functional unit and/or no contribution analysis and/or hotspots not identified; Impacts not reported through sensitivity and/or uncertainty analysis; Limitations not adequately discussed and/or potential impact of omissions or assumptions on the study's outcomes are not described and/or study not critically appraised and/or no disclosed funding source/conflicts of interest and/or funding and conflicts of interest likely to be a source of bias) and indirectness (-2; data were only collected from one study; Representativeness of data is not discussed and/or differences energy mix are not accounted for and/or potential significance of exclusions or assumptions is not addressed). | | | | | | | | |  |
|  |  |  |  |  |  |  |  |  |  |
|  |  |  |  |  |  |  |  |  |  |
|  |  |  |  |  |  |  |  |  |  |
|  |  |  |  |  |  |  |  |  |  |
|  |  |  |  |  |  |  |  |  |  |
|  |  |  |  |  |  |  |  |  |  |
| **Very low GRADE** | |  |  |  |  |  |  |  |  |
| One study (Thiel, 2015) reported on ‘eutrophication’. The level of evidence of this outcome measure was downgraded with 4 levels to very low due to: Risk of bias (-2; System boundaries and/or life cycle stages not clearly stated and/or no appropriate justification of any omitted stages; Results are not clearly reported in context of the functional unit and/or no contribution analysis and/or hotspots not identified; Impacts not reported through sensitivity and/or uncertainty analysis; Limitations not adequately discussed and/or potential impact of omissions or assumptions on the study's outcomes are not described and/or study not critically appraised and/or no disclosed funding source/conflicts of interest and/or funding and conflicts of interest likely to be a source of bias) and indirectness (-2; data were only collected from one study; Representativeness of data is not discussed and/or differences energy mix are not accounted for and/or potential significance of exclusions or assumptions is not addressed). | | | | | | | | |  |
|  |  |  |  |  |  |  |  |  |  |
|  |  |  |  |  |  |  |  |  |  |
|  |  |  |  |  |  |  |  |  |  |
|  |  |  |  |  |  |  |  |  |  |
|  |  |  |  |  |  |  |  |  |  |
|  |  |  |  |  |  |  |  |  |  |
| **Very low GRADE** | |  |  |  |  |  |  |  |  |
| One study (Thiel, 2015) reported on ‘human toxicity’. The level of evidence of this outcome measure was downgraded with 4 levels to very low due to: Risk of bias (-2; System boundaries and/or life cycle stages not clearly stated and/or no appropriate justification of any omitted stages; Results are not clearly reported in context of the functional unit and/or no contribution analysis and/or hotspots not identified; Impacts not reported through sensitivity and/or uncertainty analysis; Limitations not adequately discussed and/or potential impact of omissions or assumptions on the study's outcomes are not described and/or study not critically appraised and/or no disclosed funding source/conflicts of interest and/or funding and conflicts of interest likely to be a source of bias) and indirectness (-2; data were only collected from one study; Representativeness of data is not discussed and/or differences energy mix are not accounted for and/or potential significance of exclusions or assumptions is not addressed). | | | | | | | | |  |
|  |  |  |  |  |  |  |  |  |  |
|  |  |  |  |  |  |  |  |  |  |
|  |  |  |  |  |  |  |  |  |  |
|  |  |  |  |  |  |  |  |  |  |
|  |  |  |  |  |  |  |  |  |  |
|  |  |  |  |  |  |  |  |  |  |
| **Very low GRADE** | |  |  |  |  |  |  |  |  |
| One study (Thiel, 2015) reported on ‘ecotoxicity’. The level of evidence of this outcome measure was downgraded with 4 levels to very low due to: Risk of bias (-2; System boundaries and/or life cycle stages not clearly stated and/or no appropriate justification of any omitted stages; Results are not clearly reported in context of the functional unit and/or no contribution analysis and/or hotspots not identified; Impacts not reported through sensitivity and/or uncertainty analysis; Limitations not adequately discussed and/or potential impact of omissions or assumptions on the study's outcomes are not described and/or study not critically appraised and/or no disclosed funding source/conflicts of interest and/or funding and conflicts of interest likely to be a source of bias) and indirectness (-2; data were only collected from one study; Representativeness of data is not discussed and/or differences energy mix are not accounted for and/or potential significance of exclusions or assumptions is not addressed). | | | | | | | | |  |
|  |  |  |  |  |  |  |  |  |  |
|  |  |  |  |  |  |  |  |  |  |
|  |  |  |  |  |  |  |  |  |  |
|  |  |  |  |  |  |  |  |  |  |
|  |  |  |  |  |  |  |  |  |  |
|  |  |  |  |  |  |  |  |  |  |
| **Very low GRADE** | |  |  |  |  |  |  |  |  |
| One study (Thiel, 2015) reported on ‘ozone depletion’. The level of evidence of this outcome measure was downgraded with 4 levels to very low due to: Risk of bias (-2; System boundaries and/or life cycle stages not clearly stated and/or no appropriate justification of any omitted stages; Results are not clearly reported in context of the functional unit and/or no contribution analysis and/or hotspots not identified; Impacts not reported through sensitivity and/or uncertainty analysis; Limitations not adequately discussed and/or potential impact of omissions or assumptions on the study's outcomes are not described and/or study not critically appraised and/or no disclosed funding source/conflicts of interest and/or funding and conflicts of interest likely to be a source of bias) and indirectness (-2; data were only collected from one study; Representativeness of data is not discussed and/or differences energy mix are not accounted for and/or potential significance of exclusions or assumptions is not addressed). | | | | | | | | |  |
|  |  |  |  |  |  |  |  |  |  |
|  |  |  |  |  |  |  |  |  |  |
|  |  |  |  |  |  |  |  |  |  |
|  |  |  |  |  |  |  |  |  |  |
|  |  |  |  |  |  |  |  |  |  |

### Table 9: Topic 2. Critical appraisal points per study

| **Study** | **Phase** | **Points given** |  |  |
| --- | --- | --- | --- | --- |
| **Almutairi (2022)** | Phase 1 |  |  |  |
| Transparency |  | Study's rationale clearly stated (1) | Intended Application clearly stated (1) | Intended audience clearly stated (1) |
| Transparency |  | LCA method stated (1) |  |  |
| Consistency |  | FU defined and measurable (1) | FU justified (1) | FU consistent (1) |
| Transparency; Bias |  | System boundaries clearly stated (1) | Lifecycle stages clearly stated (1) | Appropriate justification of any omitted stages (1) |
| Internal Validity, Completeness | *Half mark if only for energy and vice versa* | System covers: production (1) of materials and energy | Use/reuse (1) of materials and energy | Disposal (1) of materials and energy |
|  | Phase 2 |  |  |  |
| Transparency, Internal Validity |  | Clear data collection (1) | Sources of data described (1) | Included data described (1) |
| Internal validity; External validity |  | Representativeness of the data is discussed (1) | Differences in electricity generating mix are accounted for (1) | Potential significance of exclusions or assumptions is addressed (1) |
| Transparency; Bias | *One mark if no allocation used* | Allocation if necessary described and justified (1) (no allocation used) |  |  |
|  | Phase 3 |  |  |  |
| Transparency |  | Documented transparantly: Impact categories (1) | Characterization method (1) | Software used (1) |
| Consistency; Transparency | *(0.5 if graphically, 0 if only normalized results reported)* | Results are clearly reported in the context of the functional unit (1) |  |  |
|  |  | A contribution analysis is performed and clearly reported (1) | Hotspots are identified (1) |  |
|  | Phase 4 |  |  |  |
| Internal validity; Consistency |  | Conclusions are consistent with the goal and scope (1) | Conclusions are supported by the impact assessment results (1) |  |
| Internal validity |  | Results are contextualized through the use of sensitivity analysis (1) | Results are contextualized through the use of uncertainty analysis (1) |  |
| Bias |  | Limitations are adequately discussed (1) | The potential impact of omissions or assumptions on the study's outcomes are described (1) |  |
| Bias |  | The assessment has been critically appraised (i.e. peer review if journal article or independent, external critical review if report/thesis; 1) |  |  |
| Bias |  | Source(s) of funding and any potential conflict(s) of interest are disclosed (1) | Sources and potential conflicts are unlikely to be a source of bias (1) |  |
|  |  |  |  |  |
|  |  |  |  |  |
| **Study** | **Phase** | **Points given** |  |  |
| **Boberg (2022)** | Phase 1 |  |  |  |
| Transparency |  | Study's rationale clearly stated (1) | Intended Application clearly stated (1) | Intended audience clearly stated (1) |
| Transparency |  | LCA method stated (1) |  |  |
| Consistency |  | FU defined and measurable (1) | FU justified (1) | FU consistent (1) |
| Transparency; Bias |  | System boundaries clearly stated (1) | Lifecycle stages clearly stated (1) | Appropriate justification of any omitted stages (1) |
| Internal Validity, Completeness | *Half mark if only for energy and vice versa* | System covers: production (1) of materials and energy | Use/reuse (1) of materials and energy | Disposal (1) of materials and energy |
|  | Phase 2 |  |  |  |
| Transparency, Internal Validity |  | Clear data collection (1) | Sources of data described (1) | Included data described (1) |
| Internal validity; External validity |  | Representativeness of the data is discussed (1) | Differences in electricity generating mix are accounted for (1) | Potential significance of exclusions or assumptions is addressed (1) |
| Transparency; Bias | *One mark if no allocation used* | Allocation if necessary described and justified (1) (no allocation used) |  |  |
|  | Phase 3 |  |  |  |
| Transparency |  | Documented transparantly: Impact categories (1) | Characterization method (1) | Software used (1) |
| Consistency; Transparency | *(0.5 if graphically, 0 if only normalized results reported)* | Results are clearly reported in the context of the functional unit (1) |  |  |
|  |  | A contribution analysis is performed and clearly reported (1) | Hotspots are identified (1) |  |
|  | Phase 4 |  |  |  |
| Internal validity; Consistency |  | Conclusions are consistent with the goal and scope (1) | Conclusions are supported by the impact assessment results (1) |  |
| Internal validity |  | Results are contextualized through the use of sensitivity analysis (1) | Results are contextualized through the use of uncertainty analysis (1) |  |
| Bias |  | Limitations are adequately discussed (1) | The potential impact of omissions or assumptions on the study's outcomes are described (1) |  |
| Bias |  | The assessment has been critically appraised (i.e. peer review if journal article or independent, external critical review if report/thesis; 1) |  |  |
| Bias |  | Source(s) of funding and any potential conflict(s) of interest are disclosed (1) | Sources and potential conflicts are unlikely to be a source of bias (1) |  |
|  |  |  |  |  |
|  |  |  |  |  |
| **Study** | **Phase** | **Points given** |  |  |
| **Boucheron (2022)** | Phase 1 |  |  |  |
| Transparency |  | Study's rationale clearly stated (1) | Intended Application clearly stated (1) | Intended audience clearly stated (1) |
| Transparency |  | LCA method stated (1) |  |  |
| Consistency |  | FU defined and measurable (1) | FU justified (1) | FU consistent (1) |
| Transparency; Bias |  | System boundaries clearly stated (1) | Lifecycle stages clearly stated (1) | Appropriate justification of any omitted stages (1) |
| Internal Validity, Completeness | *Half mark if only for energy and vice versa* | System covers: production (1) of materials and energy | Use/reuse (1) of materials and energy | Disposal (1) of materials and energy |
|  | Phase 2 |  |  |  |
| Transparency, Internal Validity |  | Clear data collection (1) | Sources of data described (1) | Included data described (1) |
| Internal validity; External validity |  | Representativeness of the data is discussed (1) | Differences in electricity generating mix are accounted for (1) | Potential significance of exclusions or assumptions is addressed (1) |
| Transparency; Bias | *One mark if no allocation used* | Allocation if necessary described and justified (1) (no allocation used) |  |  |
|  | Phase 3 |  |  |  |
| Transparency |  | Documented transparantly: Impact categories (1) | Characterization method (1) | Software used (1) |
| Consistency; Transparency | *(0.5 if graphically, 0 if only normalized results reported)* | Results are clearly reported in the context of the functional unit (1) |  |  |
|  |  | A contribution analysis is performed and clearly reported (1) | Hotspots are identified (1) |  |
|  | Phase 4 |  |  |  |
| Internal validity; Consistency |  | Conclusions are consistent with the goal and scope (1) | Conclusions are supported by the impact assessment results (1) |  |
| Internal validity |  | Results are contextualized through the use of sensitivity analysis (1) | Results are contextualized through the use of uncertainty analysis (1) |  |
| Bias |  | Limitations are adequately discussed (1) | The potential impact of omissions or assumptions on the study's outcomes are described (1) |  |
| Bias |  | The assessment has been critically appraised (i.e. peer review if journal article or independent, external critical review if report/thesis; 1) |  |  |
| Bias |  | Source(s) of funding and any potential conflict(s) of interest are disclosed (1) | Sources and potential conflicts are unlikely to be a source of bias (1) |  |
|  |  |  |  |  |
|  |  |  |  |  |
| **Study** | **Phase** | **Points given** |  |  |
| **Davis (2018)** | Phase 1 |  |  |  |
| Transparency |  | Study's rationale clearly stated (1) | Intended Application clearly stated (1) | Intended audience clearly stated (1) |
| Transparency |  | LCA method stated (1) |  |  |
| Consistency |  | FU defined and measurable (1) | FU justified (1) | FU consistent (1) |
| Transparency; Bias |  | System boundaries clearly stated (1) | Lifecycle stages clearly stated (1) | Appropriate justification of any omitted stages (1) |
| Internal Validity, Completeness | *Half mark if only for energy and vice versa* | System covers: production (1) of materials and energy | Use/reuse (1) of materials and energy | Disposal (1) of materials and energy |
|  | Phase 2 |  |  |  |
| Transparency, Internal Validity |  | Clear data collection (1) | Sources of data described (1) | Included data described (1) |
| Internal validity; External validity |  | Representativeness of the data is discussed (1) | Differences in electricity generating mix are accounted for (1) | Potential significance of exclusions or assumptions is addressed (1) |
| Transparency; Bias | *One mark if no allocation used* | Allocation if necessary described and justified (1) (no allocation) |  |  |
|  | Phase 3 |  |  |  |
| Transparency |  | Documented transparantly: Impact categories (1) | Characterization method (1) | Software used (1) |
| Consistency; Transparency | *(0.5 if graphically, 0 if only normalized results reported)* | Results are clearly reported in the context of the functional unit (1) |  |  |
|  |  | A contribution analysis is performed and clearly reported (1) | Hotspots are identified (1) |  |
|  | Phase 4 |  |  |  |
| Internal validity; Consistency |  | Conclusions are consistent with the goal and scope (1) | Conclusions are supported by the impact assessment results (1) |  |
| Internal validity |  | Results are contextualized through the use of sensitivity analysis (1) | Results are contextualized through the use of uncertainty analysis (1) |  |
| Bias |  | Limitations are adequately discussed (1) | The potential impact of omissions or assumptions on the study's outcomes are described (1) |  |
| Bias |  | The assessment has been critically appraised (i.e. peer review if journal article or independent, external critical review if report/thesis; 1) |  |  |
| Bias |  | Source(s) of funding and any potential conflict(s) of interest are disclosed (1) | Sources and potential conflicts are unlikely to be a source of bias (1) | Only ccompeting financial interest 0.5+0.5=1 |
|  |  |  |  |  |
|  |  |  |  |  |
| **Study** | **Phase** | **Points given** |  |  |
| **Donahue (2020)** | Phase 1 |  |  |  |
| Transparency |  | Study's rationale clearly stated (1) | Intended Application clearly stated (1) | Intended audience clearly stated (1) |
| Transparency |  | LCA method stated (1) |  |  |
| Consistency |  | FU defined and measurable (1) | FU justified (1) | FU consistent (1) |
| Transparency; Bias |  | System boundaries clearly stated (1) | Lifecycle stages clearly stated (1) | Appropriate justification of any omitted stages (1) |
| Internal Validity, Completeness | *Half mark if only for energy and vice versa* | System covers: production (1) of materials and energy | Use/reuse (1) of materials and energy | Disposal (1) of materials and energy |
|  | Phase 2 |  |  |  |
| Transparency, Internal Validity |  | Clear data collection (1) | Sources of data described (1) | Included data described (1) |
| Internal validity; External validity |  | Representativeness of the data is discussed (1) | Differences in electricity generating mix are accounted for (1) | Potential significance of exclusions or assumptions is addressed (1) |
| Transparency; Bias | *One mark if no allocation used* | Allocation if necessary described and justified (1) (no allocation) |  |  |
|  | Phase 3 |  |  |  |
| Transparency |  | Documented transparantly: Impact categories (1) | Characterization method (1) | Software used (1) |
| Consistency; Transparency | *(0.5 if graphically, 0 if only normalized results reported)* | Results are clearly reported in the context of the functional unit (1) |  |  |
|  |  | A contribution analysis is performed and clearly reported (1) | Hotspots are identified (1) |  |
|  | Phase 4 |  |  |  |
| Internal validity; Consistency |  | Conclusions are consistent with the goal and scope (1) | Conclusions are supported by the impact assessment results (1) |  |
| Internal validity |  | Results are contextualized through the use of sensitivity analysis (1) | Results are contextualized through the use of uncertainty analysis (1) |  |
| Bias |  | Limitations are adequately discussed (1) | The potential impact of omissions or assumptions on the study's outcomes are described (1) |  |
| Bias |  | The assessment has been critically appraised (i.e. peer review if journal article or independent, external critical review if report/thesis; 1) |  |  |
| Bias |  | Source(s) of funding and any potential conflict(s) of interest are disclosed (1) | Sources and potential conflicts are unlikely to be a source of bias (1) | Only confilict of interest 0.5+0.5=1, no funding or financial |
|  |  |  |  |  |
|  |  |  |  |  |
| **Study** | **Phase** | **Points given** |  |  |
| **Eckelman (2012)** | Phase 1 |  |  |  |
| Transparency |  | Study's rationale clearly stated (1) | Intended Application clearly stated (1) | Intended audience clearly stated (1) |
| Transparency |  | LCA method stated (1) |  |  |
| Consistency |  | FU defined and measurable (1) | FU justified (1) | FU consistent (1) |
| Transparency; Bias |  | System boundaries clearly stated (1) | Lifecycle stages clearly stated (1) | Appropriate justification of any omitted stages (1) |
| Internal Validity, Completeness | *Half mark if only for energy and vice versa* | System covers: production (1) of materials and energy | Use/reuse (1) of materials and energy | Disposal (1) of materials and energy |
|  | Phase 2 |  |  |  |
| Transparency, Internal Validity |  | Clear data collection (1) | Sources of data described (1) | Included data described (1) |
| Internal validity; External validity |  | Representativeness of the data is discussed (1) | Differences in electricity generating mix are accounted for (1) | Potential significance of exclusions or assumptions is addressed (1) |
| Transparency; Bias | *One mark if no allocation used* | Allocation if necessary described and justified (1) (no allocation) |  |  |
|  | Phase 3 |  |  |  |
| Transparency |  | Documented transparantly: Impact categories (1) | Characterization method (1) | Software used (1) |
| Consistency; Transparency | *(0.5 if graphically, 0 if only normalized results reported)* | Results are clearly reported in the context of the functional unit (1) |  |  |
|  |  | A contribution analysis is performed and clearly reported (1) | Hotspots are identified (1) |  |
|  | Phase 4 |  |  |  |
| Internal validity; Consistency |  | Conclusions are consistent with the goal and scope (1) | Conclusions are supported by the impact assessment results (1) |  |
| Internal validity |  | Results are contextualized through the use of sensitivity analysis (1) | Results are contextualized through the use of uncertainty analysis (1) |  |
| Bias |  | Limitations are adequately discussed (1) | The potential impact of omissions or assumptions on the study's outcomes are described (1) |  |
| Bias |  | The assessment has been critically appraised (i.e. peer review if journal article or independent, external critical review if report/thesis; 1) |  |  |
| Bias |  | Source(s) of funding and any potential conflict(s) of interest are disclosed (1) | Sources and potential conflicts are unlikely to be a source of bias (1) |  |
|  |  |  |  |  |
| **Study** | **Phase** | **Points given** |  |  |
| **Friedericy (2022)** | Phase 1 |  |  |  |
| Transparency |  | Study's rationale clearly stated (1) | Intended Application clearly stated (1) | Intended audience clearly stated (1) |
| Transparency |  | LCA method stated (1) |  |  |
| Consistency |  | FU defined and measurable (1) | FU justified (1) | FU consistent (1) |
| Transparency; Bias |  | System boundaries clearly stated (1) | Lifecycle stages clearly stated (1) | Appropriate justification of any omitted stages (1) |
| Internal Validity, Completeness | *Half mark if only for energy and vice versa* | System covers: production (1) of materials and energy | Use/reuse (1) of materials and energy | Disposal (1) of materials and energy |
|  | Phase 2 |  |  |  |
| Transparency, Internal Validity |  | Clear data collection (1) | Sources of data described (1) | Included data described (1) |
| Internal validity; External validity |  | Representativeness of the data is discussed (1) | Differences in electricity generating mix are accounted for (1) | Potential significance of exclusions or assumptions is addressed (1) |
| Transparency; Bias | *One mark if no allocation used* | Allocation if necessary described and justified (1) (no allocation used) |  |  |
|  | Phase 3 |  |  |  |
| Transparency |  | Documented transparantly: Impact categories (1) | Characterization method (1) | Software used (1) |
| Consistency; Transparency | *(0.5 if graphically, 0 if only normalized results reported)* | Results are clearly reported in the context of the functional unit (1) |  |  |
|  |  | A contribution analysis is performed and clearly reported (1) | Hotspots are identified (1) |  |
|  | Phase 4 |  |  |  |
| Internal validity; Consistency |  | Conclusions are consistent with the goal and scope (1) | Conclusions are supported by the impact assessment results (1) |  |
| Internal validity |  | Results are contextualized through the use of sensitivity analysis (1) | Results are contextualized through the use of uncertainty analysis (1) |  |
| Bias |  | Limitations are adequately discussed (1) | The potential impact of omissions or assumptions on the study's outcomes are described (1) |  |
| Bias |  | The assessment has been critically appraised (i.e. peer review if journal article or independent, external critical review if report/thesis; 1) |  |  |
| Bias |  | Source(s) of funding and any potential conflict(s) of interest are disclosed (1) | Sources and potential conflicts are unlikely to be a source of bias (1) |  |
|  |  |  |  |  |
|  |  |  |  |  |
| **Study** | **Phase** | **Points given** |  |  |
| **Grimmond (2012)** | Phase 1 |  |  |  |
| Transparency |  | Study's rationale clearly stated (1) | Intended Application clearly stated (1) | Intended audience clearly stated (1) |
| Transparency |  | LCA method stated (1) |  |  |
| Consistency |  | FU defined and measurable (1) | FU justified (1) | FU consistent (1) |
| Transparency; Bias |  | System boundaries clearly stated (1) | Lifecycle stages clearly stated (1) | Appropriate justification of any omitted stages (1) |
| Internal Validity, Completeness | *Half mark if only for energy and vice versa* | System covers: production (1) of materials and energy | Use/reuse (1) of materials and energy | Disposal (1) of materials and energy |
|  | Phase 2 |  |  |  |
| Transparency, Internal Validity |  | Clear data collection (1) | Sources of data described (1) | Included data described (1) |
| Internal validity; External validity |  | Representativeness of the data is discussed (1) | Differences in electricity generating mix are accounted for (1) | Potential significance of exclusions or assumptions is addressed (1) |
| Transparency; Bias | *One mark if no allocation used* | Allocation if necessary described and justified (1) (no allocation used) |  |  |
|  | Phase 3 |  |  |  |
| Transparency |  | Documented transparantly: Impact categories (1) | Characterization method (1) | Software used (1) |
| Consistency; Transparency | *(0.5 if graphically, 0 if only normalized results reported)* | Results are clearly reported in the context of the functional unit (1) |  |  |
|  |  | A contribution analysis is performed and clearly reported (1) | Hotspots are identified (1) |  |
|  | Phase 4 |  |  |  |
| Internal validity; Consistency |  | Conclusions are consistent with the goal and scope (1) | Conclusions are supported by the impact assessment results (1) |  |
| Internal validity |  | Results are contextualized through the use of sensitivity analysis (1) | Results are contextualized through the use of uncertainty analysis (1) |  |
| Bias |  | Limitations are adequately discussed (1) | The potential impact of omissions or assumptions on the study's outcomes are described (1) |  |
| Bias |  | The assessment has been critically appraised (i.e. peer review if journal article or independent, external critical review if report/thesis; 1) |  |  |
| Bias |  | Source(s) of funding and any potential conflict(s) of interest are disclosed (1) | Sources and potential conflicts are unlikely to be a source of bias (1) |  |
|  |  |  |  |  |
|  |  |  |  |  |
| **Study** | **Phase** | **Points given** |  |  |
| **Grimmond (2021)** | Phase 1 |  |  |  |
| Transparency |  | Study's rationale clearly stated (1) | Intended Application clearly stated (1) | Intended audience clearly stated (1) |
| Transparency |  | LCA method stated (1) |  |  |
| Consistency |  | FU defined and measurable (1) | FU justified (1) | FU consistent (1) |
| Transparency; Bias |  | System boundaries clearly stated (1) | Lifecycle stages clearly stated (1) | Appropriate justification of any omitted stages (1) |
| Internal Validity, Completeness | *Half mark if only for energy and vice versa* | System covers: production (1) of materials and energy | Use/reuse (1) of materials and energy | Disposal (1) of materials and energy |
|  | Phase 2 |  |  |  |
| Transparency, Internal Validity |  | Clear data collection (1) | Sources of data described (1) | Included data described (1) |
| Internal validity; External validity |  | Representativeness of the data is discussed (1) | Differences in electricity generating mix are accounted for (1) | Potential significance of exclusions or assumptions is addressed (1) |
| Transparency; Bias | *One mark if no allocation used* | Allocation if necessary described and justified (1) (no allocation used) |  |  |
|  | Phase 3 |  |  |  |
| Transparency |  | Documented transparantly: Impact categories (1) | Characterization method (1) | Software used (1) |
| Consistency; Transparency | *(0.5 if graphically, 0 if only normalized results reported)* | Results are clearly reported in the context of the functional unit (1) |  |  |
|  |  | A contribution analysis is performed and clearly reported (1) | Hotspots are identified (1) |  |
|  | Phase 4 |  |  |  |
| Internal validity; Consistency |  | Conclusions are consistent with the goal and scope (1) | Conclusions are supported by the impact assessment results (1) |  |
| Internal validity |  | Results are contextualized through the use of sensitivity analysis (1) | Results are contextualized through the use of uncertainty analysis (1) |  |
| Bias |  | Limitations are adequately discussed (1) | The potential impact of omissions or assumptions on the study's outcomes are described (1) |  |
| Bias |  | The assessment has been critically appraised (i.e. peer review if journal article or independent, external critical review if report/thesis; 1) |  |  |
| Bias |  | Source(s) of funding and any potential conflict(s) of interest are disclosed (1) | Sources and potential conflicts are unlikely to be a source of bias (1) |  |
|  |  |  |  |  |
|  |  |  |  |  |
| **Study** | **Phase** | **Points given** |  |  |
| **Hicks (2016)** | Phase 1 |  |  |  |
| Transparency |  | Study's rationale clearly stated (1) | Intended Application clearly stated (1) | Intended audience clearly stated (1) |
| Transparency |  | LCA method stated (1) |  |  |
| Consistency |  | FU defined and measurable (1) | FU justified (1) | FU consistent (1) |
| Transparency; Bias |  | System boundaries clearly stated (1) | Lifecycle stages clearly stated (1) | Appropriate justification of any omitted stages (1) |
| Internal Validity, Completeness | *Half mark if only for energy and vice versa* | System covers: production (1) of materials and energy | Use/reuse (1) of materials and energy | Disposal (1) of materials and energy |
|  | Phase 2 |  |  |  |
| Transparency, Internal Validity |  | Clear data collection (1) | Sources of data described (1) | Included data described (1) |
| Internal validity; External validity |  | Representativeness of the data is discussed (1) | Differences in electricity generating mix are accounted for (1) | Potential significance of exclusions or assumptions is addressed (1) |
| Transparency; Bias | *One mark if no allocation used* | Allocation if necessary described and justified (1) (no allocation) |  |  |
|  | Phase 3 |  |  |  |
| Transparency |  | Documented transparantly: Impact categories (1) | Characterization method (1) | Software used (1) |
| Consistency; Transparency | *(0.5 if graphically, 0 if only normalized results reported)* | Results are clearly reported in the context of the functional unit (1) |  |  |
|  |  | A contribution analysis is performed and clearly reported (1) | Hotspots are identified (1) |  |
|  | Phase 4 |  |  |  |
| Internal validity; Consistency |  | Conclusions are consistent with the goal and scope (1) | Conclusions are supported by the impact assessment results (1) |  |
| Internal validity |  | Results are contextualized through the use of sensitivity analysis (1) | Results are contextualized through the use of uncertainty analysis (1) |  |
| Bias |  | Limitations are adequately discussed (1) | The potential impact of omissions or assumptions on the study's outcomes are described (1) |  |
| Bias |  | The assessment has been critically appraised (i.e. peer review if journal article or independent, external critical review if report/thesis; 1) |  |  |
| Bias |  | Source(s) of funding and any potential conflict(s) of interest are disclosed (1) | Sources and potential conflicts are unlikely to be a source of bias (1) |  |
|  |  |  |  |  |
|  |  |  |  |  |
| **Study** | **Phase** | **Points given** |  |  |
| **Hogan (2022)** | Phase 1 |  |  |  |
| Transparency |  | Study's rationale clearly stated (1) | Intended Application clearly stated (1) | Intended audience clearly stated (1) |
| Transparency |  | LCA method stated (1) |  |  |
| Consistency |  | FU defined and measurable (1) | FU justified (1) | FU consistent (1) |
| Transparency; Bias |  | System boundaries clearly stated (1) | Lifecycle stages clearly stated (1) | Appropriate justification of any omitted stages (1) |
| Internal Validity, Completeness | *Half mark if only for energy and vice versa* | System covers: production (1) of materials and energy | Use/reuse (1) of materials and energy | Disposal (1) of materials and energy |
|  | Phase 2 |  |  |  |
| Transparency, Internal Validity |  | Clear data collection (1) | Sources of data described (1) | Included data described (1) |
| Internal validity; External validity |  | Representativeness of the data is discussed (1) | Differences in electricity generating mix are accounted for (1) | Potential significance of exclusions or assumptions is addressed (1) |
| Transparency; Bias | *One mark if no allocation used* | Allocation if necessary described and justified (1) (no allocation) |  |  |
|  | Phase 3 |  |  |  |
| Transparency |  | Documented transparantly: Impact categories (1) | Characterization method (1) | Software used (1) |
| Consistency; Transparency | *(0.5 if graphically, 0 if only normalized results reported)* | Results are clearly reported in the context of the functional unit (1) |  |  |
|  |  | A contribution analysis is performed and clearly reported (1) | Hotspots are identified (1) |  |
|  | Phase 4 |  |  |  |
| Internal validity; Consistency |  | Conclusions are consistent with the goal and scope (1) | Conclusions are supported by the impact assessment results (1) |  |
| Internal validity |  | Results are contextualized through the use of sensitivity analysis (1) | Results are contextualized through the use of uncertainty analysis (1) |  |
| Bias |  | Limitations are adequately discussed (1) | The potential impact of omissions or assumptions on the study's outcomes are described (1) |  |
| Bias |  | The assessment has been critically appraised (i.e. peer review if journal article or independent, external critical review if report/thesis; 1) |  |  |
| Bias |  | Source(s) of funding and any potential conflict(s) of interest are disclosed (1) | Sources and potential conflicts are unlikely to be a source of bias (1) |  |
|  |  |  |  |  |
|  |  |  |  |  |
| **Study** | **Phase** | **Points given** |  |  |
| **Ibbotson (2013)** | Phase 1 |  |  |  |
| Transparency |  | Study's rationale clearly stated (1) | Intended Application clearly stated (1) | Intended audience clearly stated (1) |
| Transparency |  | LCA method stated (1) |  |  |
| Consistency |  | FU defined and measurable (1) | FU justified (1) | FU consistent (1) |
| Transparency; Bias |  | System boundaries clearly stated (1) | Lifecycle stages clearly stated (1) | Appropriate justification of any omitted stages (1) |
| Internal Validity, Completeness | *Half mark if only for energy and vice versa* | System covers: production (1) of materials and energy | Use/reuse (1) of materials and energy | Disposal (1) of materials and energy |
|  | Phase 2 |  |  |  |
| Transparency, Internal Validity |  | Clear data collection (1) | Sources of data described (1) | Included data described (1) |
| Internal validity; External validity |  | Representativeness of the data is discussed (1) | Differences in electricity generating mix are accounted for (1) | Potential significance of exclusions or assumptions is addressed (1) |
| Transparency; Bias | *One mark if no allocation used* | Allocation if necessary described and justified (1) (no allocation) |  |  |
|  | Phase 3 |  |  |  |
| Transparency |  | Documented transparantly: Impact categories (1) | Characterization method (1) | Software used (1) |
| Consistency; Transparency | *(0.5 if graphically, 0 if only normalized results reported)* | Results are clearly reported in the context of the functional unit (1) (onl y graphically with log scale) |  |  |
|  |  | A contribution analysis is performed and clearly reported (1) | Hotspots are identified (1) |  |
|  | Phase 4 |  |  |  |
| Internal validity; Consistency |  | Conclusions are consistent with the goal and scope (1) | Conclusions are supported by the impact assessment results (1) |  |
| Internal validity |  | Results are contextualized through the use of sensitivity analysis (1) | Results are contextualized through the use of uncertainty analysis (1) |  |
| Bias |  | Limitations are adequately discussed (1) | The potential impact of omissions or assumptions on the study's outcomes are described (1) |  |
| Bias |  | The assessment has been critically appraised (i.e. peer review if journal article or independent, external critical review if report/thesis; 1) |  |  |
| Bias |  | Source(s) of funding and any potential conflict(s) of interest are disclosed (1) | Sources and potential conflicts are unlikely to be a source of bias (1) |  |
|  |  |  |  |  |
|  |  |  |  |  |
| **Study** | **Phase** | **Points given** |  |  |
| **Kemble (2022)** | Phase 1 |  |  |  |
| Transparency |  | Study's rationale clearly stated (1) | Intended Application clearly stated (1) | Intended audience clearly stated (1) |
| Transparency |  | LCA method stated (1) |  |  |
| Consistency |  | FU defined and measurable (1) | FU justified (1) | FU consistent (1) |
| Transparency; Bias |  | System boundaries clearly stated (1) | Lifecycle stages clearly stated (1) | Appropriate justification of any omitted stages (1) |
| Internal Validity, Completeness | *Half mark if only for energy and vice versa* | System covers: production (1) of materials and energy | Use/reuse (1) of materials and energy | Disposal (1) of materials and energy |
|  | Phase 2 |  |  |  |
| Transparency, Internal Validity |  | Clear data collection (1) | Sources of data described (1) | Included data described (1) |
| Internal validity; External validity |  | Representativeness of the data is discussed (1) | Differences in electricity generating mix are accounted for (1) | Potential significance of exclusions or assumptions is addressed (1) |
| Transparency; Bias | *One mark if no allocation used* | Allocation if necessary described and justified (1) (no allocation used) |  |  |
|  | Phase 3 |  |  |  |
| Transparency |  | Documented transparantly: Impact categories (1) | Characterization method (1) | Software used (1) |
| Consistency; Transparency | *(0.5 if graphically, 0 if only normalized results reported)* | Results are clearly reported in the context of the functional unit (1) |  |  |
|  |  | A contribution analysis is performed and clearly reported (1) | Hotspots are identified (1) |  |
|  | Phase 4 |  |  |  |
| Internal validity; Consistency |  | Conclusions are consistent with the goal and scope (1) | Conclusions are supported by the impact assessment results (1) |  |
| Internal validity |  | Results are contextualized through the use of sensitivity analysis (1) | Results are contextualized through the use of uncertainty analysis (1) |  |
| Bias |  | Limitations are adequately discussed (1) | The potential impact of omissions or assumptions on the study's outcomes are described (1) |  |
| Bias |  | The assessment has been critically appraised (i.e. peer review if journal article or independent, external critical review if report/thesis; 1) |  |  |
| Bias |  | Source(s) of funding and any potential conflict(s) of interest are disclosed (1) | Sources and potential conflicts are unlikely to be a source of bias (1) |  |
|  |  |  |  |  |
|  |  |  |  |  |
| **Study** | **Phase** | **Points given** |  |  |
| **Le (2022)** | Phase 1 |  |  |  |
| Transparency |  | Study's rationale clearly stated (1) | Intended Application clearly stated (1) | Intended audience clearly stated (1) |
| Transparency |  | LCA method stated (1) |  |  |
| Consistency |  | FU defined and measurable (1) | FU justified (1) | FU consistent (1) |
| Transparency; Bias |  | System boundaries clearly stated (1) | Lifecycle stages clearly stated (1) | Appropriate justification of any omitted stages (1) |
| Internal Validity, Completeness | *Half mark if only for energy and vice versa* | System covers: production (1) of materials and energy | Use/reuse (1) of materials and energy | Disposal (1) of materials and energy |
|  | Phase 2 |  |  |  |
| Transparency, Internal Validity |  | Clear data collection (1) | Sources of data described (1) | Included data described (1) |
| Internal validity; External validity |  | Representativeness of the data is discussed (1) | Differences in electricity generating mix are accounted for (1) | Potential significance of exclusions or assumptions is addressed (1) |
| Transparency; Bias | *One mark if no allocation used* | Allocation if necessary described and justified (1) (no allocation used) |  |  |
|  | Phase 3 |  |  |  |
| Transparency |  | Documented transparantly: Impact categories (1) | Characterization method (1) | Software used (1) |
| Consistency; Transparency | *(0.5 if graphically, 0 if only normalized results reported)* | Results are clearly reported in the context of the functional unit (1) |  |  |
|  |  | A contribution analysis is performed and clearly reported (1) | Hotspots are identified (1) |  |
|  | Phase 4 |  |  |  |
| Internal validity; Consistency |  | Conclusions are consistent with the goal and scope (1) | Conclusions are supported by the impact assessment results (1) |  |
| Internal validity |  | Results are contextualized through the use of sensitivity analysis (1) | Results are contextualized through the use of uncertainty analysis (1) |  |
| Bias |  | Limitations are adequately discussed (1) | The potential impact of omissions or assumptions on the study's outcomes are described (1) |  |
| Bias |  | The assessment has been critically appraised (i.e. peer review if journal article or independent, external critical review if report/thesis; 1) |  |  |
| Bias |  | Source(s) of funding and any potential conflict(s) of interest are disclosed (1) | Sources and potential conflicts are unlikely to be a source of bias (1) |  |
|  |  |  |  |  |
|  |  |  |  |  |
| **Study** | **Phase** | **Points given** |  |  |
| **Leiden (2020)** | Phase 1 |  |  |  |
| Transparency |  | Study's rationale clearly stated (1) | Intended Application clearly stated (1) | Intended audience clearly stated (1) |
| Transparency |  | LCA method stated (1) |  |  |
| Consistency |  | FU defined and measurable (1) | FU justified (1) | FU consistent (1) |
| Transparency; Bias |  | System boundaries clearly stated (1) | Lifecycle stages clearly stated (1) | Appropriate justification of any omitted stages (1) |
| Internal Validity, Completeness | *Half mark if only for energy and vice versa* | System covers: production (1) of materials and energy | Use/reuse (1) of materials and energy | Disposal (1) of materials and energy |
|  | Phase 2 |  |  |  |
| Transparency, Internal Validity |  | Clear data collection (1) | Sources of data described (1) | Included data described (1) |
| Internal validity; External validity |  | Representativeness of the data is discussed (1) | Differences in electricity generating mix are accounted for (1) | Potential significance of exclusions or assumptions is addressed (1) |
| Transparency; Bias | *One mark if no allocation used* | Allocation if necessary described and justified (1) (no allocation) |  |  |
|  | Phase 3 |  |  |  |
| Transparency |  | Documented transparantly: Impact categories (1) | Characterization method (1) | Software used (1) |
| Consistency; Transparency | *(0.5 if graphically, 0 if only normalized results reported)* | Results are clearly reported in the context of the functional unit (1) |  |  |
|  |  | A contribution analysis is performed and clearly reported (1) | Hotspots are identified (1) |  |
|  | Phase 4 |  |  |  |
| Internal validity; Consistency |  | Conclusions are consistent with the goal and scope (1) | Conclusions are supported by the impact assessment results (1) |  |
| Internal validity |  | Results are contextualized through the use of sensitivity analysis (1) | Results are contextualized through the use of uncertainty analysis (1) |  |
| Bias |  | Limitations are adequately discussed (1) | The potential impact of omissions or assumptions on the study's outcomes are described (1) |  |
| Bias |  | The assessment has been critically appraised (i.e. peer review if journal article or independent, external critical review if report/thesis; 1) |  |  |
| Bias |  | Source(s) of funding and any potential conflict(s) of interest are disclosed (1) | Sources and potential conflicts are unlikely to be a source of bias (1) |  |
|  |  |  |  |  |
|  |  |  |  |  |
| **Study** | **Phase** | **Points given** |  |  |
| **McGain (2010)** | Phase 1 |  |  |  |
| Transparency |  | Study's rationale clearly stated (1) | Intended Application clearly stated (1) | Intended audience clearly stated (1) |
| Transparency |  | LCA method stated (1) |  |  |
| Consistency |  | FU defined and measurable (1) | FU justified (1) | FU consistent (1) |
| Transparency; Bias |  | System boundaries clearly stated (1) | Lifecycle stages clearly stated (1) | Appropriate justification of any omitted stages (1) |
| Internal Validity, Completeness | *Half mark if only for energy and vice versa* | System covers: production (1) of materials and energy | Use/reuse (1) of materials and energy | Disposal (1) of materials and energy |
|  | Phase 2 |  |  |  |
| Transparency, Internal Validity |  | Clear data collection (1) | Sources of data described (1) | Included data described (1) |
| Internal validity; External validity |  | Representativeness of the data is discussed (1) | Differences in electricity generating mix are accounted for (1) | Potential significance of exclusions or assumptions is addressed (1) |
| Transparency; Bias | *One mark if no allocation used* | Allocation if necessary described and justified (1) (no allocation) |  |  |
|  | Phase 3 |  |  |  |
| Transparency |  | Documented transparantly: Impact categories (1) | Characterization method (1) | Software used (1) |
| Consistency; Transparency | *(0.5 if graphically, 0 if only normalized results reported)* | Results are clearly reported in the context of the functional unit (1) |  |  |
|  |  | A contribution analysis is performed and clearly reported (1) | Hotspots are identified (1) |  |
|  | Phase 4 |  |  |  |
| Internal validity; Consistency |  | Conclusions are consistent with the goal and scope (1) | Conclusions are supported by the impact assessment results (1) |  |
| Internal validity |  | Results are contextualized through the use of sensitivity analysis (1) | Results are contextualized through the use of uncertainty analysis (1) |  |
| Bias |  | Limitations are adequately discussed (1) | The potential impact of omissions or assumptions on the study's outcomes are described (1) |  |
| Bias |  | The assessment has been critically appraised (i.e. peer review if journal article or independent, external critical review if report/thesis; 1) |  |  |
| Bias |  | Source(s) of funding and any potential conflict(s) of interest are disclosed (1) | Sources and potential conflicts are unlikely to be a source of bias (1) |  |
|  |  |  |  |  |
|  |  |  |  |  |
| **Study** | **Phase** | **Points given** |  |  |
| **McGain (2012)** | Phase 1 |  |  |  |
| Transparency |  | Study's rationale clearly stated (1) | Intended Application clearly stated (1) | Intended audience clearly stated (1) |
| Transparency |  | LCA method stated (1) |  |  |
| Consistency |  | FU defined and measurable (1) | FU justified (1) | FU consistent (1) |
| Transparency; Bias |  | System boundaries clearly stated (1) | Lifecycle stages clearly stated (1) | Appropriate justification of any omitted stages (1) |
| Internal Validity, Completeness | *Half mark if only for energy and vice versa* | System covers: production (1) of materials and energy | Use/reuse (1) of materials and energy | Disposal (1) of materials and energy |
|  | Phase 2 |  |  |  |
| Transparency, Internal Validity |  | Clear data collection (1) | Sources of data described (1) | Included data described (1) |
| Internal validity; External validity |  | Representativeness of the data is discussed (1) | Differences in electricity generating mix are accounted for (1) | Potential significance of exclusions or assumptions is addressed (1) |
| Transparency; Bias | *One mark if no allocation used* | Allocation if necessary described and justified (1) (no allocation) |  |  |
|  | Phase 3 |  |  |  |
| Transparency |  | Documented transparantly: Impact categories (1) | Characterization method (1) | Software used (1) |
| Consistency; Transparency | *(0.5 if graphically, 0 if only normalized results reported)* | Results are clearly reported in the context of the functional unit (1) |  |  |
|  |  | A contribution analysis is performed and clearly reported (1) | Hotspots are identified (1) |  |
|  | Phase 4 |  |  |  |
| Internal validity; Consistency |  | Conclusions are consistent with the goal and scope (1) | Conclusions are supported by the impact assessment results (1) |  |
| Internal validity |  | Results are contextualized through the use of sensitivity analysis (1) | Results are contextualized through the use of uncertainty analysis (1) |  |
| Bias |  | Limitations are adequately discussed (1) | The potential impact of omissions or assumptions on the study's outcomes are described (1) |  |
| Bias |  | The assessment has been critically appraised (i.e. peer review if journal article or independent, external critical review if report/thesis; 1) |  |  |
| Bias |  | Source(s) of funding and any potential conflict(s) of interest are disclosed (1) | Sources and potential conflicts are unlikely to be a source of bias (1) |  |
|  |  |  |  |  |
|  |  |  |  |  |
| **Study** | **Phase** | **Points given** |  |  |
| **McGain (2017)** | Phase 1 |  |  |  |
| Transparency |  | Study's rationale clearly stated (1) | Intended Application clearly stated (1) | Intended audience clearly stated (1) |
| Transparency |  | LCA method stated (1) |  |  |
| Consistency |  | FU defined and measurable (1) | FU justified (1) | FU consistent (1) |
| Transparency; Bias |  | System boundaries clearly stated (1) | Lifecycle stages clearly stated (1) | Appropriate justification of any omitted stages (1) |
| Internal Validity, Completeness | *Half mark if only for energy and vice versa* | System covers: production (1) of materials and energy | Use/reuse (1) of materials and energy | Disposal (1) of materials and energy |
|  | Phase 2 |  |  |  |
| Transparency, Internal Validity |  | Clear data collection (1) | Sources of data described (1) | Included data described (1) |
| Internal validity; External validity |  | Representativeness of the data is discussed (1) | Differences in electricity generating mix are accounted for (1) | Potential significance of exclusions or assumptions is addressed (1) |
| Transparency; Bias | *One mark if no allocation used* | Allocation if necessary described and justified (1) (no allocation) |  |  |
|  | Phase 3 |  |  |  |
| Transparency |  | Documented transparantly: Impact categories (1) | Characterization method (1) | Software used (1) |
| Consistency; Transparency | *(0.5 if graphically, 0 if only normalized results reported)* | Results are clearly reported in the context of the functional unit (1) |  |  |
|  |  | A contribution analysis is performed and clearly reported (1) | Hotspots are identified (1) |  |
|  | Phase 4 |  |  |  |
| Internal validity; Consistency |  | Conclusions are consistent with the goal and scope (1) | Conclusions are supported by the impact assessment results (1) |  |
| Internal validity |  | Results are contextualized through the use of sensitivity analysis (1) | Results are contextualized through the use of uncertainty analysis (1) |  |
| Bias |  | Limitations are adequately discussed (1) | The potential impact of omissions or assumptions on the study's outcomes are described (1) |  |
| Bias |  | The assessment has been critically appraised (i.e. peer review if journal article or independent, external critical review if report/thesis; 1) |  |  |
| Bias |  | Source(s) of funding and any potential conflict(s) of interest are disclosed (1) | Sources and potential conflicts are unlikely to be a source of bias (1) |  |
|  |  |  |  |  |
|  |  |  |  |  |
| **Study** | **Phase** | **Points given** |  |  |
| **McPherson (2019)** | Phase 1 |  |  |  |
| Transparency |  | Study's rationale clearly stated (1) | Intended Application clearly stated (1) | Intended audience clearly stated (1) |
| Transparency |  | LCA method stated (1) |  |  |
| Consistency |  | FU defined and measurable (1) | FU justified (1) | FU consistent (1) |
| Transparency; Bias |  | System boundaries clearly stated (1) | Lifecycle stages clearly stated (1) | Appropriate justification of any omitted stages (1) |
| Internal Validity, Completeness | *Half mark if only for energy and vice versa* | System covers: production (1) of materials and energy | Use/reuse (1) of materials and energy | Disposal (1) of materials and energy |
|  | Phase 2 |  |  |  |
| Transparency, Internal Validity |  | Clear data collection (1) | Sources of data described (1) | Included data described (1) |
| Internal validity; External validity |  | Representativeness of the data is discussed (1) | Differences in electricity generating mix are accounted for (1) | Potential significance of exclusions or assumptions is addressed (1) |
| Transparency; Bias | *One mark if no allocation used* | Allocation if necessary described and justified (1) (no allocation) |  |  |
|  | Phase 3 |  |  |  |
| Transparency |  | Documented transparantly: Impact categories (1) | Characterization method (1) | Software used (1) |
| Consistency; Transparency | *(0.5 if graphically, 0 if only normalized results reported)* | Results are clearly reported in the context of the functional unit (1) |  |  |
|  |  | A contribution analysis is performed and clearly reported (1) | Hotspots are identified (1) |  |
|  | Phase 4 |  |  |  |
| Internal validity; Consistency |  | Conclusions are consistent with the goal and scope (1) | Conclusions are supported by the impact assessment results (1) |  |
| Internal validity |  | Results are contextualized through the use of sensitivity analysis (1) | Results are contextualized through the use of uncertainty analysis (1) |  |
| Bias |  | Limitations are adequately discussed (1) | The potential impact of omissions or assumptions on the study's outcomes are described (1) |  |
| Bias |  | The assessment has been critically appraised (i.e. peer review if journal article or independent, external critical review if report/thesis; 1) |  |  |
| Bias |  | Source(s) of funding and any potential conflict(s) of interest are disclosed (1) | Sources and potential conflicts are unlikely to be a source of bias (1) |  |
|  |  |  |  |  |
|  |  |  |  |  |
| **Study** | **Phase** | **Points given** |  |  |
| **Morris (2022)** | Phase 1 |  |  |  |
| Transparency |  | Study's rationale clearly stated (1) | Intended Application clearly stated (1) | Intended audience clearly stated (1) |
| Transparency |  | LCA method stated (1) |  |  |
| Consistency |  | FU defined and measurable (1) | FU justified (1) | FU consistent (1) |
| Transparency; Bias |  | System boundaries clearly stated (1) | Lifecycle stages clearly stated (1) | Appropriate justification of any omitted stages (1) |
| Internal Validity, Completeness | *Half mark if only for energy and vice versa* | System covers: production (1) of materials and energy | Use/reuse (1) of materials and energy | Disposal (1) of materials and energy |
|  | Phase 2 |  |  |  |
| Transparency, Internal Validity |  | Clear data collection (1) | Sources of data described (1) | Included data described (1) |
| Internal validity; External validity |  | Representativeness of the data is discussed (1) | Differences in electricity generating mix are accounted for (1) | Potential significance of exclusions or assumptions is addressed (1) |
| Transparency; Bias | *One mark if no allocation used* | Allocation if necessary described and justified (1) (no allocation used) |  |  |
|  | Phase 3 |  |  |  |
| Transparency |  | Documented transparantly: Impact categories (1) | Characterization method (1) | Software used (1) |
| Consistency; Transparency | *(0.5 if graphically, 0 if only normalized results reported)* | Results are clearly reported in the context of the functional unit (1) |  |  |
|  |  | A contribution analysis is performed and clearly reported (1) | Hotspots are identified (1) |  |
|  | Phase 4 |  |  |  |
| Internal validity; Consistency |  | Conclusions are consistent with the goal and scope (1) | Conclusions are supported by the impact assessment results (1) |  |
| Internal validity |  | Results are contextualized through the use of sensitivity analysis (1) | Results are contextualized through the use of uncertainty analysis (1) |  |
| Bias |  | Limitations are adequately discussed (1) | The potential impact of omissions or assumptions on the study's outcomes are described (1) |  |
| Bias |  | The assessment has been critically appraised (i.e. peer review if journal article or independent, external critical review if report/thesis; 1) |  |  |
| Bias |  | Source(s) of funding and any potential conflict(s) of interest are disclosed (1) | Sources and potential conflicts are unlikely to be a source of bias (1) |  |
|  |  |  |  |  |
|  |  |  |  |  |
| **Study** | **Phase** | **Points given** |  |  |
| **Namburar (2012)** | Phase 1 |  |  |  |
| Transparency |  | Study's rationale clearly stated (1) | Intended Application clearly stated (1) | Intended audience clearly stated (1) |
| Transparency |  | LCA method stated (1) |  |  |
| Consistency |  | FU defined and measurable (1) | FU justified (1) | FU consistent (1) |
| Transparency; Bias |  | System boundaries clearly stated (1) | Lifecycle stages clearly stated (1) | Appropriate justification of any omitted stages (1) |
| Internal Validity, Completeness | *Half mark if only for energy and vice versa* | System covers: production (1) of materials and energy | Use/reuse (1) of materials and energy | Disposal (1) of materials and energy |
|  | Phase 2 |  |  |  |
| Transparency, Internal Validity |  | Clear data collection (1) | Sources of data described (1) | Included data described (1) |
| Internal validity; External validity |  | Representativeness of the data is discussed (1) | Differences in electricity generating mix are accounted for (1) | Potential significance of exclusions or assumptions is addressed (1) |
| Transparency; Bias | *One mark if no allocation used* | Allocation if necessary described and justified (1) (no allocation used) |  |  |
|  | Phase 3 |  |  |  |
| Transparency |  | Documented transparantly: Impact categories (1) | Characterization method (1) | Software used (1) |
| Consistency; Transparency | *(0.5 if graphically, 0 if only normalized results reported)* | Results are clearly reported in the context of the functional unit (1) |  |  |
|  |  | A contribution analysis is performed and clearly reported (1) | Hotspots are identified (1) |  |
|  | Phase 4 |  |  |  |
| Internal validity; Consistency |  | Conclusions are consistent with the goal and scope (1) | Conclusions are supported by the impact assessment results (1) |  |
| Internal validity |  | Results are contextualized through the use of sensitivity analysis (1) | Results are contextualized through the use of uncertainty analysis (1) |  |
| Bias |  | Limitations are adequately discussed (1) | The potential impact of omissions or assumptions on the study's outcomes are described (1) |  |
| Bias |  | The assessment has been critically appraised (i.e. peer review if journal article or independent, external critical review if report/thesis; 1) |  |  |
| Bias |  | Source(s) of funding and any potential conflict(s) of interest are disclosed (1) | Sources and potential conflicts are unlikely to be a source of bias (1) |  |
|  |  |  |  |  |
|  |  |  |  |  |
| **Study** | **Phase** | **Points given** |  |  |
| **Rizan (2021)** | Phase 1 |  |  |  |
| Transparency |  | Study's rationale clearly stated (1) | Intended Application clearly stated (1) | Intended audience clearly stated (1) |
| Transparency |  | LCA method stated (1) |  |  |
| Consistency |  | FU defined and measurable (1) | FU justified (1) | FU consistent (1) |
| Transparency; Bias |  | System boundaries clearly stated (1) | Lifecycle stages clearly stated (1) | Appropriate justification of any omitted stages (1) |
| Internal Validity, Completeness | *Half mark if only for energy and vice versa* | System covers: production (1) of materials and energy | Use/reuse (1) of materials and energy | Disposal (1) of materials and energy |
|  | Phase 2 |  |  |  |
| Transparency, Internal Validity |  | Clear data collection (1) | Sources of data described (1) | Included data described (1) |
| Internal validity; External validity |  | Representativeness of the data is discussed (1) | Differences in electricity generating mix are accounted for (1) | Potential significance of exclusions or assumptions is addressed (1) |
| Transparency; Bias | *One mark if no allocation used* | Allocation if necessary described and justified (1) (no allocation) |  |  |
|  | Phase 3 |  |  |  |
| Transparency |  | Documented transparantly: Impact categories (1) | Characterization method (1) | Software used (1) |
| Consistency; Transparency | *(0.5 if graphically, 0 if only normalized results reported)* | Results are clearly reported in the context of the functional unit (1) |  |  |
|  |  | A contribution analysis is performed and clearly reported (1) | Hotspots are identified (1) |  |
|  | Phase 4 |  |  |  |
| Internal validity; Consistency |  | Conclusions are consistent with the goal and scope (1) | Conclusions are supported by the impact assessment results (1) |  |
| Internal validity |  | Results are contextualized through the use of sensitivity analysis (1) | Results are contextualized through the use of uncertainty analysis (1) |  |
| Bias |  | Limitations are adequately discussed (1) | The potential impact of omissions or assumptions on the study's outcomes are described (1) |  |
| Bias |  | The assessment has been critically appraised (i.e. peer review if journal article or independent, external critical review if report/thesis; 1) |  |  |
| Bias |  | Source(s) of funding and any potential conflict(s) of interest are disclosed (1) | Sources and potential conflicts are unlikely to be a source of bias (1) |  |
|  |  |  |  |  |
|  |  |  |  |  |
| **Study** | **Phase** | **Points given** |  |  |
| **Sanchez (2020)** | Phase 1 |  |  |  |
| Transparency |  | Study's rationale clearly stated (1) | Intended Application clearly stated (1) | Intended audience clearly stated (1) |
| Transparency |  | LCA method stated (1) |  |  |
| Consistency |  | FU defined and measurable (1) | FU justified (1) | FU consistent (1) |
| Transparency; Bias |  | System boundaries clearly stated (1) | Lifecycle stages clearly stated (1) | Appropriate justification of any omitted stages (1) |
| Internal Validity, Completeness | *Half mark if only for energy and vice versa* | System covers: production (1) of materials and energy | Use/reuse (1) of materials and energy | Disposal (1) of materials and energy |
|  | Phase 2 |  |  |  |
| Transparency, Internal Validity |  | Clear data collection (1) | Sources of data described (1) | Included data described (1) |
| Internal validity; External validity |  | Representativeness of the data is discussed (1) | Differences in electricity generating mix are accounted for (1) | Potential significance of exclusions or assumptions is addressed (1) |
| Transparency; Bias | *One mark if no allocation used* | Allocation if necessary described and justified (1) (no allocation) |  |  |
|  | Phase 3 |  |  |  |
| Transparency |  | Documented transparantly: Impact categories (1) | Characterization method (1) | Software used (1) |
| Consistency; Transparency | *(0.5 if graphically, 0 if only normalized results reported)* | Results are clearly reported in the context of the functional unit (1) |  |  |
|  |  | A contribution analysis is performed and clearly reported (1) | Hotspots are identified (1) |  |
|  | Phase 4 |  |  |  |
| Internal validity; Consistency |  | Conclusions are consistent with the goal and scope (1) | Conclusions are supported by the impact assessment results (1) |  |
| Internal validity |  | Results are contextualized through the use of sensitivity analysis (1) | Results are contextualized through the use of uncertainty analysis (1) |  |
| Bias |  | Limitations are adequately discussed (1) | The potential impact of omissions or assumptions on the study's outcomes are described (1) |  |
| Bias |  | The assessment has been critically appraised (i.e. peer review if journal article or independent, external critical review if report/thesis; 1) |  |  |
| Bias |  | Source(s) of funding and any potential conflict(s) of interest are disclosed (1) | Sources and potential conflicts are unlikely to be a source of bias (1) |  |
|  |  |  |  |  |
|  |  |  |  |  |
| **Study** | **Phase** | **Points given** |  |  |
| **Sherman (2018)** | Phase 1 |  |  |  |
| Transparency |  | Study's rationale clearly stated (1) | Intended Application clearly stated (1) | Intended audience clearly stated (1) |
| Transparency |  | LCA method stated (1) |  |  |
| Consistency |  | FU defined and measurable (1) | FU justified (1) | FU consistent (1) |
| Transparency; Bias |  | System boundaries clearly stated (1) | Lifecycle stages clearly stated (1) | Appropriate justification of any omitted stages (1) |
| Internal Validity, Completeness | *Half mark if only for energy and vice versa* | System covers: production (1) of materials and energy | Use/reuse (1) of materials and energy | Disposal (1) of materials and energy |
|  | Phase 2 |  |  |  |
| Transparency, Internal Validity |  | Clear data collection (1) | Sources of data described (1) | Included data described (1) |
| Internal validity; External validity |  | Representativeness of the data is discussed (1) | Differences in electricity generating mix are accounted for (1) | Potential significance of exclusions or assumptions is addressed (1) |
| Transparency; Bias | *One mark if no allocation used* | Allocation if necessary described and justified (1) (no allocation) |  |  |
|  | Phase 3 |  |  |  |
| Transparency |  | Documented transparantly: Impact categories (1) | Characterization method (1) | Software used (1) |
| Consistency; Transparency | *(0.5 if graphically, 0 if only normalized results reported)* | Results are clearly reported in the context of the functional unit (1) |  |  |
|  |  | A contribution analysis is performed and clearly reported (1) | Hotspots are identified (1) |  |
|  | Phase 4 |  |  |  |
| Internal validity; Consistency |  | Conclusions are consistent with the goal and scope (1) | Conclusions are supported by the impact assessment results (1) |  |
| Internal validity |  | Results are contextualized through the use of sensitivity analysis (1) | Results are contextualized through the use of uncertainty analysis (1) |  |
| Bias |  | Limitations are adequately discussed (1) | The potential impact of omissions or assumptions on the study's outcomes are described (1) |  |
| Bias |  | The assessment has been critically appraised (i.e. peer review if journal article or independent, external critical review if report/thesis; 1) |  |  |
| Bias |  | Source(s) of funding and any potential conflict(s) of interest are disclosed (1) | Sources and potential conflicts are unlikely to be a source of bias (1) |  |
|  |  |  |  |  |
|  |  |  |  |  |
| **Study** | **Phase** | **Points given** |  |  |
| **Sorensen (2022)** | Phase 1 |  |  |  |
| Transparency |  | Study's rationale clearly stated (1) | Intended Application clearly stated (1) | Intended audience clearly stated (1) |
| Transparency |  | LCA method stated (1) |  |  |
| Consistency |  | FU defined and measurable (1) | FU justified (1) | FU consistent (1) |
| Transparency; Bias |  | System boundaries clearly stated (1) | Lifecycle stages clearly stated (1) | Appropriate justification of any omitted stages (1) |
| Internal Validity, Completeness | *Half mark if only for energy and vice versa* | System covers: production (1) of materials and energy | Use/reuse (1) of materials and energy | Disposal (1) of materials and energy |
|  | Phase 2 |  |  |  |
| Transparency, Internal Validity |  | Clear data collection (1) | Sources of data described (1) | Included data described (1) |
| Internal validity; External validity |  | Representativeness of the data is discussed (1) | Differences in electricity generating mix are accounted for (1) | Potential significance of exclusions or assumptions is addressed (1) |
| Transparency; Bias | *One mark if no allocation used* | Allocation if necessary described and justified (1) (no allocation used) |  |  |
|  | Phase 3 |  |  |  |
| Transparency |  | Documented transparantly: Impact categories (1) | Characterization method (1) | Software used (1) |
| Consistency; Transparency | *(0.5 if graphically, 0 if only normalized results reported)* | Results are clearly reported in the context of the functional unit (1) |  |  |
|  |  | A contribution analysis is performed and clearly reported (1) | Hotspots are identified (1) |  |
|  | Phase 4 |  |  |  |
| Internal validity; Consistency |  | Conclusions are consistent with the goal and scope (1) | Conclusions are supported by the impact assessment results (1) |  |
| Internal validity |  | Results are contextualized through the use of sensitivity analysis (1) | Results are contextualized through the use of uncertainty analysis (1) |  |
| Bias |  | Limitations are adequately discussed (1) | The potential impact of omissions or assumptions on the study's outcomes are described (1) |  |
| Bias |  | The assessment has been critically appraised (i.e. peer review if journal article or independent, external critical review if report/thesis; 1) |  |  |
| Bias |  | Source(s) of funding and any potential conflict(s) of interest are disclosed (1) | Sources and potential conflicts are unlikely to be a source of bias (1) |  |
|  |  |  |  |  |
|  |  |  |  |  |
| **Study** | **Phase** | **Points given** |  |  |
| **Unger (2014)** | Phase 1 |  |  |  |
| Transparency |  | Study's rationale clearly stated (1) | Intended Application clearly stated (1) | Intended audience clearly stated (1) |
| Transparency |  | LCA method stated (1) |  |  |
| Consistency |  | FU defined and measurable (1) | FU justified (1) | FU consistent (1) |
| Transparency; Bias |  | System boundaries clearly stated (1) | Lifecycle stages clearly stated (1) | Appropriate justification of any omitted stages (1) |
| Internal Validity, Completeness | *Half mark if only for energy and vice versa* | System covers: production (1) of materials and energy | Use/reuse (1) of materials and energy | Disposal (1) of materials and energy |
|  | Phase 2 |  |  |  |
| Transparency, Internal Validity |  | Clear data collection (1) | Sources of data described (1) | Included data described (1) |
| Internal validity; External validity |  | Representativeness of the data is discussed (1) | Differences in electricity generating mix are accounted for (1) | Potential significance of exclusions or assumptions is addressed (1) |
| Transparency; Bias | *One mark if no allocation used* | Allocation if necessary described and justified (1) (no allocation used) |  |  |
|  | Phase 3 |  |  |  |
| Transparency |  | Documented transparantly: Impact categories (1) | Characterization method (1) | Software used (1) |
| Consistency; Transparency | *(0.5 if graphically, 0 if only normalized results reported)* | Results are clearly reported in the context of the functional unit (1) |  |  |
|  |  | A contribution analysis is performed and clearly reported (1) | Hotspots are identified (1) |  |
|  | Phase 4 |  |  |  |
| Internal validity; Consistency |  | Conclusions are consistent with the goal and scope (1) | Conclusions are supported by the impact assessment results (1) |  |
| Internal validity |  | Results are contextualized through the use of sensitivity analysis (1) | Results are contextualized through the use of uncertainty analysis (1) |  |
| Bias |  | Limitations are adequately discussed (1) | The potential impact of omissions or assumptions on the study's outcomes are described (1) |  |
| Bias |  | The assessment has been critically appraised (i.e. peer review if journal article or independent, external critical review if report/thesis; 1) |  |  |
| Bias |  | Source(s) of funding and any potential conflict(s) of interest are disclosed (1) | Sources and potential conflicts are unlikely to be a source of bias (1) |  |
|  |  |  |  |  |
| **Study** | **Phase** | **Points given** |  |  |
| **Vozzola (2018)** | Phase 1 |  |  |  |
| Transparency |  | Study's rationale clearly stated (1) | Intended Application clearly stated (1) | Intended audience clearly stated (1) |
| Transparency |  | LCA method stated (1) |  |  |
| Consistency |  | FU defined and measurable (1) | FU justified (1) | FU consistent (1) |
| Transparency; Bias |  | System boundaries clearly stated (1) | Lifecycle stages clearly stated (1) | Appropriate justification of any omitted stages (1) |
| Internal Validity, Completeness | *Half mark if only for energy and vice versa* | System covers: production (1) of materials and energy | Use/reuse (1) of materials and energy | Disposal (1) of materials and energy |
|  | Phase 2 |  |  |  |
| Transparency, Internal Validity |  | Clear data collection (1) | Sources of data described (1) | Included data described (1) |
| Internal validity; External validity |  | Representativeness of the data is discussed (1) | Differences in electricity generating mix are accounted for (1) | Potential significance of exclusions or assumptions is addressed (1) |
| Transparency; Bias | *One mark if no allocation used* | Allocation if necessary described and justified (1) (no allocation) |  |  |
|  | Phase 3 |  |  |  |
| Transparency |  | Documented transparantly: Impact categories (1) | Characterization method (1) | Software used (1) |
| Consistency; Transparency | *(0.5 if graphically, 0 if only normalized results reported)* | Results are clearly reported in the context of the functional unit (1) |  |  |
|  |  | A contribution analysis is performed and clearly reported (1) | Hotspots are identified (1) |  |
|  | Phase 4 |  |  |  |
| Internal validity; Consistency |  | Conclusions are consistent with the goal and scope (1) | Conclusions are supported by the impact assessment results (1) |  |
| Internal validity |  | Results are contextualized through the use of sensitivity analysis (1) | Results are contextualized through the use of uncertainty analysis (1) |  |
| Bias |  | Limitations are adequately discussed (1) | The potential impact of omissions or assumptions on the study's outcomes are described (1) |  |
| Bias |  | The assessment has been critically appraised (i.e. peer review if journal article or independent, external critical review if report/thesis; 1) |  |  |
| Bias |  | Source(s) of funding and any potential conflict(s) of interest are disclosed (1) | Sources and potential conflicts are unlikely to be a source of bias (1) |  |
|  |  |  |  |  |
| **Study** | **Phase** | **Points given** |  |  |
| **Vozzola (2020)** | Phase 1 |  |  |  |
| Transparency |  | Study's rationale clearly stated (1) | Intended Application clearly stated (1) | Intended audience clearly stated (1) |
| Transparency |  | LCA method stated (1) |  |  |
| Consistency |  | FU defined and measurable (1) | FU justified (1) | FU consistent (1) |
| Transparency; Bias |  | System boundaries clearly stated (1) | Lifecycle stages clearly stated (1) | Appropriate justification of any omitted stages (1) |
| Internal Validity, Completeness | *Half mark if only for energy and vice versa* | System covers: production (1) of materials and energy | Use/reuse (1) of materials and energy | Disposal (1) of materials and energy |
|  | Phase 2 |  |  |  |
| Transparency, Internal Validity |  | Clear data collection (1) | Sources of data described (1) | Included data described (1) |
| Internal validity; External validity |  | Representativeness of the data is discussed (1) | Differences in electricity generating mix are accounted for (1) | Potential significance of exclusions or assumptions is addressed (1) |
| Transparency; Bias | *One mark if no allocation used* | Allocation if necessary described and justified (1) (no allocation) |  |  |
|  | Phase 3 |  |  |  |
| Transparency |  | Documented transparantly: Impact categories (1) | Characterization method (1) | Software used (1) |
| Consistency; Transparency | *(0.5 if graphically, 0 if only normalized results reported)* | Results are clearly reported in the context of the functional unit (1) |  |  |
|  |  | A contribution analysis is performed and clearly reported (1) | Hotspots are identified (1) |  |
|  | Phase 4 |  |  |  |
| Internal validity; Consistency |  | Conclusions are consistent with the goal and scope (1) | Conclusions are supported by the impact assessment results (1) |  |
| Internal validity |  | Results are contextualized through the use of sensitivity analysis (1) | Results are contextualized through the use of uncertainty analysis (1) |  |
| Bias |  | Limitations are adequately discussed (1) | The potential impact of omissions or assumptions on the study's outcomes are described (1) |  |
| Bias |  | The assessment has been critically appraised (i.e. peer review if journal article or independent, external critical review if report/thesis; 1) |  |  |
| Bias |  | Source(s) of funding and any potential conflict(s) of interest are disclosed (1) | Sources and potential conflicts are unlikely to be a source of bias (1) |  |
|  |  |  |  |  |

### Table 10: Topic 2. GRADE assessment per included outcome

| **Outcome** |  |  |  |  |  |  |  |  |  |  |  |  |
| --- | --- | --- | --- | --- | --- | --- | --- | --- | --- | --- | --- | --- |
| Climate change |  |  | **Missing according to critical appraisal** | |  |  |  |  |  |  |  |  |
| *Almutairi, 2022; Boberg, 2022; Davis, 2019; Donahue, 2020; Eckelman, 2012; Friedericy, 2022; Grimmond, 2012; Grimmond, 2021; Hicks, 2016; Hogan, 2022; Ibbotson, 2013; Kemble, 2022; Le, 2022; Leiden, 2020; McGain, 2010; McGain, 2012; McGain, 2017; McPherson, 2019; Morris, 2022; Rizan, 2021; Sanchez, 2020; Sherman, 2018; Sorensen, 2022; Unger, 2014; Vozzola, 2018; Vozzola, 2020* | | | Phase 1 (bias) | Consistency - FU not clearly defined and measurable, FU not justified and/or FU not consistent | | | | | |  |  |  |
|  |  |  | Phase 2 (indi) | Internal/external validity - Representativeness of data is not discussed and/or differences energy mix are not accounted for and/or potential significance of exclusions or assumptions is not addressed | | | | | | | | |
|  |  |  | Phase 3 (bias) | Transparency - Impact categories not documented transparently and/or missing information on characterization method and/or software | | | | | | | | |
|  |  |  | Phase 4 (bias) | Internal validity - Impacts not reported through sensitivity and/or uncertainty analysis | | | | | |  |  |  |
|  |  |  | Phase 4 (bias) | Bias - Limitations not adequately discussed and/or potential impact of omissions or assumptions on the study's outcomes are not described and/or study not critically appraised and/or no disclosed funding source/conflicts of interest and/or funding and conflicts of interest likely to be a source of bias | | | | | | | | |
|  |  |  | (indirectness) | Heterogeneity of interventions | |  |  |  |  |  |  |  |
|  |  |  |  |  |  |  |  |  |  |  |  |  |
|  |  |  |  |  |  |  |  |  |  |  |  |  |
| Waste |  |  | **Missing according to critical appraisal** | |  |  |  |  |  |  |  |  |
| *Boucheron, 2022; Grimmond, 2012; Grimmond, 2021; Hogan, 2022; Kemble, 2022; McPherson, 2019; Vozzola, 2018; Vozzola, 2020; Davis, 2018; McGain, 2017; Namburar, 2022* | | | Phase 1 (bias) | Transparency - Study's rationale and/or intended application and/or intended audience not clearly stated | | | | | | |  |  |
|  |  |  | Phase 1 (bias) | Consistency - FU not clearly defined and measurable, FU not justified and/or FU not consistent | | | | | |  |  |  |
|  |  |  | Phase 1 (bias) | Transparency, bias - System boundaries and/or life cycle stages not clearly stated and/or no appropriate justification of any omitted stages | | | | | | | | |
|  |  |  | Phase 2 (indi) | Internal/external validity - Representativeness of data is not discussed and/or differences energy mix are not accounted for and/or potential significance of exclusions or assumptions is not addressed | | | | | | | | |
|  |  |  | Phase 3 (bias) | Transparency - Impact categories not documented transparently and/or missing information on characterization method and/or software | | | | | | | | |
|  |  |  | Phase 4 (bias) | Internal validity - Impacts not reported through sensitivity and/or uncertainty analysis | | | | | |  |  |  |
|  |  |  | Phase 4 (bias) | Bias - Limitations not adequately discussed and/or potential impact of omissions or assumptions on the study's outcomes are not described and/or study not critically appraised and/or no disclosed funding source/conflicts of interest and/or funding and conflicts of interest likely to be a source of bias | | | | | | | | |
|  |  |  | (indirectness) | Heterogeneity of interventions | |  |  |  |  |  |  |  |
|  |  |  |  |  |  |  |  |  |  |  |  |  |
| Acidification |  |  | **Missing according to critical appraisal** | |  |  |  |  |  |  |  |  |
| *Almutairi, 2022; Boberg, 2022; Eckelman, 2012; Friedericy, 2022; Hicks, 2016; Ibbotson, 2013; Leiden, 2020; Morris, 2022; Rizan, 2021; Sanchez, 2020; Sherman, 2018; Sorensen, 2022; Unger, 2014* | | | Phase 2 (indi) | Internal/external validity - Representativeness of data is not discussed and/or differences energy mix are not accounted for and/or potential significance of exclusions or assumptions is not addressed | | | | | | | |  |
|  |  |  | Phase 4 (bias) | Internal validity - Impacts not reported through sensitivity and/or uncertainty analysis | | | | | |  |  |  |
|  |  |  | Phase 4 (bias) | Bias - Limitations not adequately discussed and/or potential impact of omissions or assumptions on the study's outcomes are not described and/or study not critically appraised and/or no disclosed funding source/conflicts of interest and/or funding and conflicts of interest likely to be a source of bias | | | | | | | | |
|  |  |  | (indirectness) | Heterogeneity of interventions | |  |  |  |  |  |  |  |
|  |  |  |  |  |  |  |  |  |  |  |  |  |
|  |  |  |  |  |  |  |  |  |  |  |  |  |
|  |  |  |  |  |  |  |  |  |  |  |  |  |
|  |  |  |  |  |  |  |  |  |  |  |  |  |
| Eutrophication |  |  | **Missing according to critical appraisal** | |  |  |  |  |  |  |  |  |
| *Almutairi, 2022; Boberg, 2022; Eckelman, 2012; Friedericy, 2022; Hicks, 2016; Ibbotson, 2013; Leiden, 2020; Morris, 2022; Rizan, 2021; Sanchez, 2020; Sherman, 2018; Unger, 2014* | | | Phase 1 (bias) | Transparency, bias - System boundaries and/or life cycle stages not clearly stated and/or no appropriate justification of any omitted stages | | | | | | | |  |
|  |  |  | Phase 2 (indi) | Internal/external validity - Representativeness of data is not discussed and/or differences energy mix are not accounted for and/or potential significance of exclusions or assumptions is not addressed | | | | | | | | |
|  |  |  | Phase 4 (bias) | Internal validity - Impacts not reported through sensitivity and/or uncertainty analysis | | | | | |  |  |  |
|  |  |  | Phase 4 (bias) | Bias - Limitations not adequately discussed and/or potential impact of omissions or assumptions on the study's outcomes are not described and/or study not critically appraised and/or no disclosed funding source/conflicts of interest and/or funding and conflicts of interest likely to be a source of bias | | | | | | | | |
|  |  |  | (indirectness) | Heterogeneity of interventions | |  |  |  |  |  |  |  |
|  |  |  |  |  |  |  |  |  |  |  |  |  |
|  |  |  |  |  |  |  |  |  |  |  |  |  |
|  |  |  |  |  |  |  |  |  |  |  |  |  |
| Human toxicity |  |  | **Missing according to critical appraisal** | |  |  |  |  |  |  |  |  |
| *Boberg, 2022; Eckelman, 2012; Friedericy, 2022; Hicks, 2016; Ibbotson, 2013; Leiden, 2020; Morris, 2022; Rizan, 2021; Sanchez, 2020; Sherman, 2018; Unger, 2014* | | | Phase 2 (indi) | Internal/external validity - Representativeness of data is not discussed and/or differences energy mix are not accounted for and/or potential significance of exclusions or assumptions is not addressed | | | | | | | |  |
|  |  |  | Phase 4 (bias) | Internal validity - Impacts not reported through sensitivity and/or uncertainty analysis | | | | | |  |  |  |
|  |  |  | Phase 4 (bias) | Bias - Limitations not adequately discussed and/or potential impact of omissions or assumptions on the study's outcomes are not described and/or study not critically appraised and/or no disclosed funding source/conflicts of interest and/or funding and conflicts of interest likely to be a source of bias | | | | | | | | |
|  |  |  | (indirectness) | Heterogeneity of interventions | |  |  |  |  |  |  |  |
|  |  |  |  |  |  |  |  |  |  |  |  |  |
|  |  |  |  |  |  |  |  |  |  |  |  |  |
|  |  |  |  |  |  |  |  |  |  |  |  |  |
|  |  |  |  |  |  |  |  |  |  |  |  |  |
| Ecotoxicity |  |  | **Missing according to critical appraisal** |  |  |  |  |  |  |  |  |  |
| *Boberg, 2022; Eckelman, 2012; Friedericy, 2022; Hicks, 2016; Ibbotson, 2013; Leiden, 2020; Morris, 2022; Rizan, 2021; Sanchez, 2020; Sherman, 2018; Unger, 2014* | | | Phase 2 (indi) | Internal/external validity - Representativeness of data is not discussed and/or differences energy mix are not accounted for and/or potential significance of exclusions or assumptions is not addressed | | | | | | | |  |
|  |  |  | Phase 4 (bias) | Internal validity - Impacts not reported through sensitivity and/or uncertainty analysis | | | | | |  |  |  |
|  |  |  | Phase 4 (bias) | Bias - Limitations not adequately discussed and/or potential impact of omissions or assumptions on the study's outcomes are not described and/or study not critically appraised and/or no disclosed funding source/conflicts of interest and/or funding and conflicts of interest likely to be a source of bias | | | | | | | | |
|  |  |  | (indirectness) | Heterogeneity of interventions | |  |  |  |  |  |  |  |
|  |  |  |  |  |  |  |  |  |  |  |  |  |
|  |  |  |  |  |  |  |  |  |  |  |  |  |
|  |  |  |  |  |  |  |  |  |  |  |  |  |
|  |  |  |  |  |  |  |  |  |  |  |  |  |
| Ozone depletion |  |  | **Missing according to critical appraisal** | |  |  |  |  |  |  |  |  |
| *Almutairi, 2022; Boberg, 2022; Eckelman, 2012; Hicks, 2016; Ibbotson, 2013; Leiden, 2020; Morris, 2022; Rizan, 2021; Sanchez, 2020; Sherman, 2018; Sorensen, 2022; Unger, 2014* | | | Phase 2 (indi) | Internal/external validity - Representativeness of data is not discussed and/or differences energy mix are not accounted for and/or potential significance of exclusions or assumptions is not addressed | | | | | | | |  |
|  |  |  | Phase 4 (bias) | Internal validity - Impacts not reported through sensitivity and/or uncertainty analysis | | | | | |  |  |  |
|  |  |  | Phase 4 (bias) | Bias - Limitations not adequately discussed and/or potential impact of omissions or assumptions on the study's outcomes are not described and/or study not critically appraised and/or no disclosed funding source/conflicts of interest and/or funding and conflicts of interest likely to be a source of bias | | | | | | | | |
|  |  |  | (indirectness) | Heterogeneity of interventions | |  |  |  |  |  |  |  |
|  |  |  |  |  |  |  |  |  |  |  |  |  |
|  |  |  |  |  |  |  |  |  |  |  |  |  |
|  |  |  |  |  |  |  |  |  |  |  |  |  |

| **GRADE** |  |  |  |  |  |  |  |  |  |
| --- | --- | --- | --- | --- | --- | --- | --- | --- | --- |
| Twenty-seven studies reported on the outcome climate change (Almutairi, 2022; Boberg, 2022; Davis, 2019; Donahue, 2020; Eckelman, 2012; Friedericy, 2022; Grimmond, 2012; Grimmond, 2021; Hicks, 2016; Hogan, 2022; Ibbotson, 2013; Kemble, 2022; Le, 2022; Leiden, 2020; McGain, 2010; McGain, 2012; McGain, 2017; McPherson, 2019; Morris, 2022; Rizan, 2021; Sanchez, 2020; Sherman, 2018; Sorensen, 2022; Unger, 2014; Vozzola, 2018; Vozzola, 2020). The level of evidence starts at grade high. The level of evidence was downgraded with 2 levels to low because of risk of bias (-1; FU not clearly defined and measurable, FU not justified and/or FU not consistent; Impact categories not documented transparently and/or missing information on characterization method and/or software; Impacts not reported through sensitivity and/or uncertainty analysis; Limitations not adequately discussed and/or potential impact of omissions or assumptions on the study's outcomes are not described and/or study not critically appraised and/or no disclosed funding source/conflicts of interest and/or funding and conflicts of interest likely to be a source of bias) and indirectness (-1; heterogeneity of interventions; Representativeness of data is not discussed and/or differences energy mix are not accounted for and/or potential significance of exclusions or assumptions is not addressed). | | | | | | | | |  |
|  |  |  |  |  |  |  |  |  |  |
|  |  |  |  |  |  |  |  |  |  |
|  |  |  |  |  |  |  |  |  |  |
|  |  |  |  |  |  |  |  |  |  |
|  |  |  |  |  |  |  |  |  |  |
|  |  |  |  |  |  |  |  |  |  |
|  |  |  |  |  |  |  |  |  |  |
| **GRADE** |  |  |  |  |  |  |  |  |  |
| Eleven studies reported on the outcome waste (Boucheron, 2022; Grimmond, 2012; Grimmond, 2021; Hogan, 2022; Kemble, 2022; McPherson, 2019; Vozzola, 2018; Vozzola, 2020; Davis, 2018; McGain, 2017; Namburar, 2022). As eight out of nine studies contain LCAs, the level of evidence starts at grade high. The level of evidence was downgraded with 2 levels to low because of risk of bias (-1; Study's rationale and/or intended application and/or intended audience not clearly stated; FU not clearly defined and measurable, FU not justified and/or FU not consistent; System boundaries and/or life cycle stages not clearly stated and/or no appropriate justification of any omitted stages; Impact categories not documented transparently and/or missing information on characterization method and/or software; Impacts not reported through sensitivity and/or uncertainty analysis; Limitations not adequately discussed and/or potential impact of omissions or assumptions on the study's outcomes are not described and/or study not critically appraised and/or no disclosed funding source/conflicts of interest and/or funding and conflicts of interest likely to be a source of bias) and indirectness (-1; heterogeneity of interventions; Representativeness of data is not discussed and/or differences energy mix are not accounted for and/or potential significance of exclusions or assumptions is not addressed). | | | | | | | | |  |
|  |  |  |  |  |  |  |  |  |  |
|  |  |  |  |  |  |  |  |  |  |
|  |  |  |  |  |  |  |  |  |  |
|  |  |  |  |  |  |  |  |  |  |
|  |  |  |  |  |  |  |  |  |  |
|  |  |  |  |  |  |  |  |  |  |
|  |  |  |  |  |  |  |  |  |  |
|  |  |  |  |  |  |  |  |  |  |
| **GRADE** |  |  |  |  |  |  |  |  |  |
| Thirteen studies reported on the outcome acidification (Almutairi, 2022; Boberg, 2022; Eckelman, 2012; Friedericy, 2022; Hicks, 2016; Ibbotson, 2013; Leiden, 2020; Morris, 2022; Rizan, 2021; Sanchez, 2020; Sherman, 2018; Sorensen, 2022; Unger, 2014). The level of evidence starts at grade high. The level of evidence was downgraded with 2 levels to low because of risk of bias (-1; Impacts not reported through sensitivity and/or uncertainty analysis; Limitations not adequately discussed and/or potential impact of omissions or assumptions on the study's outcomes are not described and/or study not critically appraised and/or no disclosed funding source/conflicts of interest and/or funding and conflicts of interest likely to be a source of bias) and indirectness (-1; Representativeness of data is not discussed and/or differences energy mix are not accounted for and/or potential significance of exclusions or assumptions is not addressed; heterogeneity of interventions). | | | | | | | | |  |
|  |  |  |  |  |  |  |  |  |  |
|  |  |  |  |  |  |  |  |  |  |
|  |  |  |  |  |  |  |  |  |  |
|  |  |  |  |  |  |  |  |  |  |
|  |  |  |  |  |  |  |  |  |  |
|  |  |  |  |  |  |  |  |  |  |
|  |  |  |  |  |  |  |  |  |  |
| **GRADE** |  |  |  |  |  |  |  |  |  |
| Twelve studies reported on the outcome eutrophication (Almutairi, 2022; Boberg, 2022; Eckelman, 2012; Friedericy, 2022; Hicks, 2016; Ibbotson, 2013; Leiden, 2020; Morris, 2022; Rizan, 2021; Sanchez, 2020; Sherman, 2018; Unger, 2014). The level of evidence starts at grade high. The level of evidence was downgraded with 2 levels to low because of risk of bias (-1; System boundaries and/or life cycle stages not clearly stated and/or no appropriate justification of any omitted stages; Impacts not reported through sensitivity and/or uncertainty analysis; Limitations not adequately discussed and/or potential impact of omissions or assumptions on the study's outcomes are not described and/or study not critically appraised and/or no disclosed funding source/conflicts of interest and/or funding and conflicts of interest likely to be a source of bias) and indirectness (-1; Representativeness of data is not discussed and/or differences energy mix are not accounted for and/or potential significance of exclusions or assumptions is not addressed; heterogeneity of interventions). | | | | | | | | |  |
|  |  |  |  |  |  |  |  |  |  |
|  |  |  |  |  |  |  |  |  |  |
|  |  |  |  |  |  |  |  |  |  |
|  |  |  |  |  |  |  |  |  |  |
|  |  |  |  |  |  |  |  |  |  |
|  |  |  |  |  |  |  |  |  |  |
|  |  |  |  |  |  |  |  |  |  |
| **GRADE** |  |  |  |  |  |  |  |  |  |
| Eleven studies reported on the human toxicity (Boberg, 2022; Eckelman, 2012; Friedericy, 2022; Hicks, 2016; Ibbotson, 2013; Leiden, 2020; Morris, 2022; Rizan, 2021; Sanchez, 2020; Sherman, 2018; Unger, 2014). The level of evidence starts at grade high. The level of evidence was downgraded with 2 levels to low because of risk of bias (-1; Impacts not reported through sensitivity and/or uncertainty analysis; Limitations not adequately discussed and/or potential impact of omissions or assumptions on the study's outcomes are not described and/or study not critically appraised and/or no disclosed funding source/conflicts of interest and/or funding and conflicts of interest likely to be a source of bias) and indirectness (-1; Representativeness of data is not discussed and/or differences energy mix are not accounted for and/or potential significance of exclusions or assumptions is not addressed; heterogeneity of interventions). | | | | | | | | |  |
|  |  |  |  |  |  |  |  |  |  |
|  |  |  |  |  |  |  |  |  |  |
|  |  |  |  |  |  |  |  |  |  |
|  |  |  |  |  |  |  |  |  |  |
|  |  |  |  |  |  |  |  |  |  |
|  |  |  |  |  |  |  |  |  |  |
|  |  |  |  |  |  |  |  |  |  |
| **GRADE** |  |  |  |  |  |  |  |  |  |
| Eleven studies reported on the human toxicity (Boberg, 2022; Eckelman, 2012; Friedericy, 2022; Hicks, 2016; Ibbotson, 2013; Leiden, 2020; Morris, 2022; Rizan, 2021; Sanchez, 2020; Sherman, 2018; Unger, 2014). The level of evidence starts at grade high. The level of evidence was downgraded with 2 levels to low because of risk of bias (-1; Impacts not reported through sensitivity and/or uncertainty analysis; Limitations not adequately discussed and/or potential impact of omissions or assumptions on the study's outcomes are not described and/or study not critically appraised and/or no disclosed funding source/conflicts of interest and/or funding and conflicts of interest likely to be a source of bias) and indirectness (-1; Representativeness of data is not discussed and/or differences energy mix are not accounted for and/or potential significance of exclusions or assumptions is not addressed; heterogeneity of interventions). | | | | | | | | |  |
|  |  |  |  |  |  |  |  |  |  |
|  |  |  |  |  |  |  |  |  |  |
|  |  |  |  |  |  |  |  |  |  |
|  |  |  |  |  |  |  |  |  |  |
|  |  |  |  |  |  |  |  |  |  |
|  |  |  |  |  |  |  |  |  |  |
|  |  |  |  |  |  |  |  |  |  |
| **GRADE** |  |  |  |  |  |  |  |  |  |
| Twelve studies reported on the outcome ozone depletion (Almutairi, 2022; Boberg, 2022; Eckelman, 2012; Hicks, 2016; Ibbotson, 2013; Leiden, 2020; Morris, 2022; Rizan, 2021; Sanchez, 2020; Sherman, 2018; Sorensen, 2022; Unger, 2014). The level of evidence starts at grade high. The level of evidence was downgraded with 2 levels to low because of risk of bias (-1; Impacts not reported through sensitivity and/or uncertainty analysis; Limitations not adequately discussed and/or potential impact of omissions or assumptions on the study's outcomes are not described and/or study not critically appraised and/or no disclosed funding source/conflicts of interest and/or funding and conflicts of interest likely to be a source of bias) and indirectness (-1; Representativeness of data is not discussed and/or differences energy mix are not accounted for and/or potential significance of exclusions or assumptions is not addressed; heterogeneity of interventions). | | | | | | | | |  |
|  |  |  |  |  |  |  |  |  |  |
|  |  |  |  |  |  |  |  |  |  |
|  |  |  |  |  |  |  |  |  |  |
|  |  |  |  |  |  |  |  |  |  |
|  |  |  |  |  |  |  |  |  |  |
|  |  |  |  |  |  |  |  |  |  |

### Table 11: Topic 4. Critical appraisal points per study

| **Study** | **Phase** | **Points given** |  |  |
| --- | --- | --- | --- | --- |
| **Hu et al. (2021)** | Phase 1 |  |  |  |
| Transparency |  | Study's rationale clearly stated (1) | Intended Application clearly stated (1) | Intended audience clearly stated (1) |
| Transparency |  | LCA method stated (1) |  |  |
| Consistency |  | FU defined and measurable (1) | FU justified (1) | FU consistent (1) |
| Transparency; Bias |  | System boundaries clearly stated (1) | Lifecycle stages clearly stated (1) | Appropriate justification of any omitted stages (1) |
| Internal Validity, Completeness | *Half mark if only for energy and vice versa* | System covers: production (1) of materials and energy | Use/reuse (1) of materials and energy | Disposal (1) of materials and energy |
|  | Phase 2 |  |  |  |
| Transparency, Internal Validity |  | Clear data collection (1) | Sources of data described (1) | Included data described (1) |
| Internal validity; External validity |  | Representativeness of the data is discussed (1) | Differences in electricity generating mix are accounted for (1) | Potential significance of exclusions or assumptions is addressed (1) |
| Transparency; Bias | *One mark if no allocation used* | Allocation if necessary described and justified (1) (no allocation) |  |  |
|  | Phase 3 |  |  |  |
| Transparency |  | Documented transparantly: Impact categories (1) | Characterization method (1) | Software used (1) |
| Consistency; Transparency | *(0.5 if graphically, 0 if only normalized results reported)* | Results are clearly reported in the context of the functional unit (1) |  |  |
|  |  | A contribution analysis is performed and clearly reported (1) | Hotspots are identified (1) |  |
|  | Phase 4 |  |  |  |
| Internal validity; Consistency |  | Conclusions are consistent with the goal and scope (1) | Conclusions are supported by the impact assessment results (1) |  |
| Internal validity |  | Results are contextualized through the use of sensitivity analysis (1) | Results are contextualized through the use of uncertainty analysis (1) |  |
| Bias |  | Limitations are adequately discussed (1) | The potential impact of omissions or assumptions on the study's outcomes are described (1) |  |
| Bias |  | The assessment has been critically appraised (i.e. peer review if journal article or independent, external critical review if report/thesis; 1) |  |  |
| Bias |  | Source(s) of funding and any potential conflict(s) of interest are disclosed (1) | Sources and potential conflicts are unlikely to be a source of bias (1) |  |
|  |  |  |  |  |
|  |  |  |  |  |
| **Study** | **Phase** | **Points given** |  |  |
| **McGain et al. (2021)** | Phase 1 |  |  |  |
| Transparency |  | Study's rationale clearly stated (1) | Intended Application clearly stated (1) | Intended audience clearly stated (1) |
| Transparency |  | LCA method stated (1) |  |  |
| Consistency |  | FU defined and measurable (1) | FU justified (1) | FU consistent (1) |
| Transparency; Bias |  | System boundaries clearly stated (1) | Lifecycle stages clearly stated (1) | Appropriate justification of any omitted stages (1) |
| Internal Validity, Completeness | *Half mark if only for energy and vice versa* | System covers: production (1) of materials and energy | Use/reuse (1) of materials and energy | Disposal (1) of materials and energy |
|  | Phase 2 |  |  |  |
| Transparency, Internal Validity |  | Clear data collection (1) | Sources of data described (1) | Included data described (1) |
| Internal validity; External validity |  | Representativeness of the data is discussed (1) | Differences in electricity generating mix are accounted for (1) | Potential significance of exclusions or assumptions is addressed (1) |
| Transparency; Bias | *One mark if no allocation used* | Allocation if necessary described and justified (1) (no allocation) |  |  |
|  | Phase 3 |  |  |  |
| Transparency |  | Documented transparantly: Impact categories (1) | Characterization method (1) | Software used (1) |
| Consistency; Transparency | *(0.5 if graphically, 0 if only normalized results reported)* | Results are clearly reported in the context of the functional unit (1) |  |  |
|  |  | A contribution analysis is performed and clearly reported (1) | Hotspots are identified (1) |  |
|  | Phase 4 |  |  |  |
| Internal validity; Consistency |  | Conclusions are consistent with the goal and scope (1) | Conclusions are supported by the impact assessment results (1) |  |
| Internal validity |  | Results are contextualized through the use of sensitivity analysis (1) | Results are contextualized through the use of uncertainty analysis (1) |  |
| Bias |  | Limitations are adequately discussed (1) | The potential impact of omissions or assumptions on the study's outcomes are described (1) |  |
| Bias |  | The assessment has been critically appraised (i.e. peer review if journal article or independent, external critical review if report/thesis; 1) |  |  |
| Bias |  | Source(s) of funding and any potential conflict(s) of interest are disclosed (1) | Sources and potential conflicts are unlikely to be a source of bias (1) |  |
|  |  |  |  |  |
|  |  |  |  |  |
| **Study** | **Phase** | **Points given** |  |  |
| **Sherman et al. (2012)** | Phase 1 |  |  |  |
| Transparency |  | Study's rationale clearly stated (1) | Intended Application clearly stated (1) | Intended audience clearly stated (1) |
| Transparency |  | LCA method stated (1) |  |  |
| Consistency |  | FU defined and measurable (1) | FU justified (1) | FU consistent (1) |
| Transparency; Bias |  | System boundaries clearly stated (1) | Lifecycle stages clearly stated (1) | Appropriate justification of any omitted stages (1) |
| Internal Validity, Completeness | *Half mark if only for energy and vice versa* | System covers: production (1) of materials and energy | Use/reuse (1) of materials and energy | Disposal (1) of materials and energy |
|  | Phase 2 |  |  |  |
| Transparency, Internal Validity |  | Clear data collection (1) | Sources of data described (1) | Included data described (1) |
| Internal validity; External validity |  | Representativeness of the data is discussed (1) | Differences in electricity generating mix are accounted for (1) | Potential significance of exclusions or assumptions is addressed (1) |
| Transparency; Bias | *One mark if no allocation used* | Allocation if necessary described and justified (1) (no allocation) |  |  |
|  | Phase 3 |  |  |  |
| Transparency |  | Documented transparantly: Impact categories (1) | Characterization method (1) | Software used (1) |
| Consistency; Transparency | *(0.5 if graphically, 0 if only normalized results reported)* | Results are clearly reported in the context of the functional unit (1) |  |  |
|  |  | A contribution analysis is performed and clearly reported (1) | Hotspots are identified (1) |  |
|  | Phase 4 |  |  |  |
| Internal validity; Consistency |  | Conclusions are consistent with the goal and scope (1) | Conclusions are supported by the impact assessment results (1) |  |
| Internal validity |  | Results are contextualized through the use of sensitivity analysis (1) | Results are contextualized through the use of uncertainty analysis (1) |  |
| Bias |  | Limitations are adequately discussed (1) | The potential impact of omissions or assumptions on the study's outcomes are described (1) |  |
| Bias |  | The assessment has been critically appraised (i.e. peer review if journal article or independent, external critical review if report/thesis; 1) |  |  |
| Bias |  | Source(s) of funding and any potential conflict(s) of interest are disclosed (1) | Sources and potential conflicts are unlikely to be a source of bias (1) |  |
|  |  |  |  |  |
|  |  |  |  |  |
| **Study** | **Phase** | **Points given** |  |  |
| **Thiel et al. (2018)** | Phase 1 |  |  |  |
| Transparency |  | Study's rationale clearly stated (1) | Intended Application clearly stated (1) | Intended audience clearly stated (1) |
| Transparency |  | LCA method stated (1) |  |  |
| Consistency |  | FU defined and measurable (1) | FU justified (1) | FU consistent (1) |
| Transparency; Bias |  | System boundaries clearly stated (1) | Lifecycle stages clearly stated (1) | Appropriate justification of any omitted stages (1) |
| Internal Validity, Completeness | *Half mark if only for energy and vice versa* | System covers: production (1) of materials and energy | Use/reuse (1) of materials and energy | Disposal (1) of materials and energy |
|  | Phase 2 |  |  |  |
| Transparency, Internal Validity |  | Clear data collection (1) | Sources of data described (1) | Included data described (1) |
| Internal validity; External validity |  | Representativeness of the data is discussed (1) | Differences in electricity generating mix are accounted for (1) | Potential significance of exclusions or assumptions is addressed (1) |
| Transparency; Bias | *One mark if no allocation used* | Allocation if necessary described and justified (1) (no allocation) |  |  |
|  | Phase 3 |  |  |  |
| Transparency |  | Documented transparantly: Impact categories (1) | Characterization method (1) | Software used (1) |
| Consistency; Transparency | *(0.5 if graphically, 0 if only normalized results reported)* | Results are clearly reported in the context of the functional unit (1) |  |  |
|  |  | A contribution analysis is performed and clearly reported (1) | Hotspots are identified (1) |  |
|  | Phase 4 |  |  |  |
| Internal validity; Consistency |  | Conclusions are consistent with the goal and scope (1) | Conclusions are supported by the impact assessment results (1) |  |
| Internal validity |  | Results are contextualized through the use of sensitivity analysis (1) | Results are contextualized through the use of uncertainty analysis (1) |  |
| Bias |  | Limitations are adequately discussed (1) | The potential impact of omissions or assumptions on the study's outcomes are described (1) |  |
| Bias |  | The assessment has been critically appraised (i.e. peer review if journal article or independent, external critical review if report/thesis; 1) |  |  |
| Bias |  | Source(s) of funding and any potential conflict(s) of interest are disclosed (1) | Sources and potential conflicts are unlikely to be a source of bias (1) |  |
|  |  |  |  |  |
|  |  |  |  |  |
| **Study** | **Phase** | **Points given** |  |  |
| **Wang et al. (2022)** | Phase 1 |  |  |  |
| Transparency |  | Study's rationale clearly stated (1) | Intended Application clearly stated (1) | Intended audience clearly stated (1) |
| Transparency |  | LCA method stated (1) |  |  |
| Consistency |  | FU defined and measurable (1) | FU justified (1) | FU consistent (1) |
| Transparency; Bias |  | System boundaries clearly stated (1) | Lifecycle stages clearly stated (1) | Appropriate justification of any omitted stages (1) |
| Internal Validity, Completeness | *Half mark if only for energy and vice versa* | System covers: production (1) of materials and energy | Use/reuse (1) of materials and energy | Disposal (1) of materials and energy |
|  | Phase 2 |  |  |  |
| Transparency, Internal Validity |  | Clear data collection (1) | Sources of data described (1) | Included data described (1) |
| Internal validity; External validity |  | Representativeness of the data is discussed (1) | Differences in electricity generating mix are accounted for (1) | Potential significance of exclusions or assumptions is addressed (1) |
| Transparency; Bias | *One mark if no allocation used* | Allocation if necessary described and justified (1) (no allocation) |  |  |
|  | Phase 3 |  |  |  |
| Transparency |  | Documented transparantly: Impact categories (1) | Characterization method (1) | Software used (1) |
| Consistency; Transparency | *(0.5 if graphically, 0 if only normalized results reported)* | Results are clearly reported in the context of the functional unit (1) |  |  |
|  |  | A contribution analysis is performed and clearly reported (1) | Hotspots are identified (1) |  |
|  | Phase 4 |  |  |  |
| Internal validity; Consistency |  | Conclusions are consistent with the goal and scope (1) | Conclusions are supported by the impact assessment results (1) |  |
| Internal validity |  | Results are contextualized through the use of sensitivity analysis (1) | Results are contextualized through the use of uncertainty analysis (1) |  |
| Bias |  | Limitations are adequately discussed (1) | The potential impact of omissions or assumptions on the study's outcomes are described (1) |  |
| Bias |  | The assessment has been critically appraised (i.e. peer review if journal article or independent, external critical review if report/thesis; 1) |  |  |
| Bias |  | Source(s) of funding and any potential conflict(s) of interest are disclosed (1) | Sources and potential conflicts are unlikely to be a source of bias (1) |  |
|  |  |  |  |  |

### Table 12: Topic 4. GRADE assessment per included outcome

| **Outcomes subquestion 4.1** | | |  |  |  |  |  |  |  |  |  |  |
| --- | --- | --- | --- | --- | --- | --- | --- | --- | --- | --- | --- | --- |
| Climate change | |  | **Missing according to critical appraisal** |  |  |  |  |  |  |  |  |  |
| *Sherman, 2012; Thiel, 2018* | | | Phase 2 (indi) | Internal/external validity - Representativeness of data is not discussed and/or differences energy mix are not accounted for and/or potential significance of exclusions or assumptions is not addressed | | | | | | | |  |
|  |  |  | Phase 4 (bias) | Internal validity - Impacts not reported through sensitivity and/or uncertainty analysis | | | | | |  |  |  |
|  |  |  | Phase 4 (bias) | Bias - Limitations not adequately discussed and/or potential impact of omissions or assumptions on the study's outcomes are not described and/or study not critically appraised and/or no disclosed funding source/conflicts of interest and/or funding and conflicts of interest likely to be a source of bias | | | | | | | | |
|  |  |  | (indirectness) | Heterogeneity of interventions | | |  |  |  |  |  |  |
|  |  |  |  |  |  |  |  |  |  |  |  |  |
|  |  |  |  |  |  |  |  |  |  |  |  |  |
|  |  |  |  |  |  |  |  |  |  |  |  |  |
|  |  |  |  |  |  |  |  |  |  |  |  |  |
| Other outcomes | |  | **Missing according to critical appraisal** | |  |  |  |  |  |  |  |  |
| *Waste, medicine in residue water, human toxicity, ozone depletion* | | | Waste, medicine in residue water, human toxicity, ozone depletion were not reported and could not be graded. | | | | | | |  |  |  |
|  |  |  |  |  |  |  |  |  |  |  |  |  |
|  |  |  |  |  |  |  |  |  |  |  |  |  |
|  |  |  |  |  |  |  |  |  |  |  |  |  |
|  |  |  |  |  |  |  |  |  |  |  |  |  |
|  |  |  |  |  |  |  |  |  |  |  |  |  |
|  |  |  |  |  |  |  |  |  |  |  |  |  |
|  |  |  |  |  |  |  |  |  |  |  |  |  |
|  |  |  |  |  |  |  |  |  |  |  |  |  |
| **Outcomes subquestion 4.2** |  |  |  |  |  |  |  |  |  |  |  |  |
| Climate change | |  | **Missing according to critical appraisal** | |  |  |  |  |  |  |  |  |
| *Hu, 2021* | | | Phase 1 (bias) | Internal validity/completeness - Missing system coverage of production and/or use/reuse and/or disposal of materials and/or energy | | | | | | | |  |
|  |  |  | Phase 2 (indi) | Internal/external validity - Representativeness of data is not discussed and/or differences energy mix are not accounted for and/or potential significance of exclusions or assumptions is not addressed | | | | | | | | |
|  |  |  | Phase 4 (bias) | Internal validity - Impacts not reported through sensitivity and/or uncertainty analysis | | | | | |  |  |  |
|  |  |  | Phase 4 (bias) | Bias - Limitations not adequately discussed and/or potential impact of omissions or assumptions on the study's outcomes are not described and/or study not critically appraised and/or no disclosed funding source/conflicts of interest and/or funding and conflicts of interest likely to be a source of bias | | | | | | | | |
|  |  |  | (indirectness) | One study |  |  |  |  |  |  |  |  |
|  |  |  |  |  |  |  |  |  |  |  |  |  |
|  |  |  |  |  |  |  |  |  |  |  |  |  |
|  |  |  |  |  |  |  |  |  |  |  |  |  |
| Other outcomes | |  | **Missing according to critical appraisal** | |  |  |  |  |  |  |  |  |
| *Waste, medicine in residue water, human toxicity, ozone depletion* | | | Waste, medicine in residue water, human toxicity, ozone depletion were not reported and could not be graded. | | | | | | |  |  |  |
|  |  |  |  |  |  |  |  |  |  |  |  |  |
|  |  |  |  |  |  |  |  |  |  |  |  |  |
|  |  |  |  |  |  |  |  |  |  |  |  |  |
|  |  |  |  |  |  |  |  |  |  |  |  |  |
|  |  |  |  |  |  |  |  |  |  |  |  |  |
|  |  |  |  |  |  |  |  |  |  |  |  |  |
|  |  |  |  |  |  |  |  |  |  |  |  |  |
|  |  |  |  |  |  |  |  |  |  |  |  |  |
| **Outcomes subquestion 4.3** | | |  |  |  |  |  |  |  |  |  |  |
| Climate change | |  | **Missing according to critical appraisal** | |  |  |  |  |  |  |  |  |
| *McGain, 2021; Wang, 2022* | | | Phase 1 (bias) | Consistency - FU not defined and measurable and/or FU not justified and/or FU not consistent | | | | | |  |  |  |
|  |  |  | Phase 1 (indi) | Internal Validity, Completeness - The system does not covers all stages: production (1), use/reuse (1) and disposal (1) of materials and energy | | | | | | | |  |
|  |  |  | Phase 2 (indi) | Transparency, internal validity - Data not clearly described, sources and included data not clearly described | | | | | |  |  |  |
|  |  |  | Phase 2 (indi) | Internal and external validity - Representativeness of data not discussed, differences in elektricity mix not accounted for, potential significance of ommitted stages not justified | | | | | | | | |
|  |  |  | Phase 3 (bias) | Transparency - Impact categories not documented transparently and/or characterization method not described and/or software used not described | | | | | | | | |
|  |  |  |  |  |  |  |  |  |  |  |  |  |
|  |  |  |  |  |  |  |  |  |  |  |  |  |
|  |  |  |  |  |  |  |  |  |  |  |  |  |
| Waste |  |  | **Missing according to critical appraisal** |  |  |  |  |  |  |  |  |  |
| *McGain, 2021* | | | Phase 1 (bias) | Consistency - FU not defined and measurable and/or FU not justified and/or FU not consistent | | | | | |  |  |  |
|  |  |  | Phase 3 (bias) | Transparency - Impact categories not documented transparently and/or characterization method not described and/or software used not described | | | | | | | | |
|  |  |  | (indirectness) | One study |  |  |  |  |  |  |  |  |
|  |  |  |  |  |  |  |  |  |  |  |  |  |
|  |  |  |  |  |  |  |  |  |  |  |  |  |
|  |  |  |  |  |  |  |  |  |  |  |  |  |
|  |  |  |  |  |  |  |  |  |  |  |  |  |
|  |  |  |  |  |  |  |  |  |  |  |  |  |
| Other outcomes | |  | **Missing according to critical appraisal** | |  |  |  |  |  |  |  |  |
| *Medicine in residue water, human toxicity, ozone depletion* | | | Medicine in residue water, human toxicity, ozone depletion were not reported and could not be graded. | | | | | | |  |  |  |
|  |  |  |  |  |  |  |  |  |  |  |  |  |
|  |  |  |  |  |  |  |  |  |  |  |  |  |
|  |  |  |  |  |  |  |  |  |  |  |  |  |
|  |  |  |  |  |  |  |  |  |  |  |  |  |
|  |  |  |  |  |  |  |  |  |  |  |  |  |
|  |  |  |  |  |  |  |  |  |  |  |  |  |
|  |  |  |  |  |  |  |  |  |  |  |  |  |

| **GRADE** |  |  |  |  |  |  |  |  |  |
| --- | --- | --- | --- | --- | --- | --- | --- | --- | --- |
| Two studies (Sherman, 2012; Thiel, 2018) reported on ‘climate change’. The level of evidence of this outcome measure was downgraded with 3 levels to **very low** due to risk of bias (-2; Impacts not reported through sensitivity and/or uncertainty analysis; Limitations not adequately discussed and/or potential impact of omissions or assumptions on the study's outcomes are not described and/or study not critically appraised and/or no disclosed funding source/conflicts of interest and/or funding and conflicts of interest likely to be a source of bias) and indirectness (-1; Representativeness of data is not discussed and/or differences energy mix are not accounted for and/or potential significance of exclusions or assumptions is not addressed; heterogeneity of interventions). | | | | | | | | |  |
|  |  |  |  |  |  |  |  |  |  |
|  |  |  |  |  |  |  |  |  |  |
|  |  |  |  |  |  |  |  |  |  |
|  |  |  |  |  |  |  |  |  |  |
|  |  |  |  |  |  |  |  |  |  |
|  |  |  |  |  |  |  |  |  |  |
|  |  |  |  |  |  |  |  |  |  |
| **GRADE** |  |  |  |  |  |  |  |  |  |
| Waste, medicine in residue water, human toxicity, ozone depletion were not reported and could not be graded. | | | | | | | | |  |
|  |  |  |  |  |  |  |  |  |  |
|  |  |  |  |  |  |  |  |  |  |
|  |  |  |  |  |  |  |  |  |  |
|  |  |  |  |  |  |  |  |  |  |
|  |  |  |  |  |  |  |  |  |  |
|  |  |  |  |  |  |  |  |  |  |
|  |  |  |  |  |  |  |  |  |  |
|  |  |  |  |  |  |  |  |  |  |
|  |  |  |  |  |  |  |  |  |  |
| **GRADE** |  |  |  |  |  |  |  |  |  |
| One study (Hu, 2021) reported on ‘climate change’. The level of evidence of this outcome measure was downgraded with 4 levels to **very low** due to risk of bias (-2; Missing system coverage of production and/or use/reuse and/or disposal of materials and/or energy; Impacts not reported through sensitivity and/or uncertainty analysis; Limitations not adequately discussed and/or potential impact of omissions or assumptions on the study's outcomes are not described and/or study not critically appraised and/or no disclosed funding source/conflicts of interest and/or funding and conflicts of interest likely to be a source of bias) and indirectness (-2; Representativeness of data is not discussed and/or differences energy mix are not accounted for and/or potential significance of exclusions or assumptions is not addressed; data were only collected from one study). | | | | | | | | |  |
|  |  |  |  |  |  |  |  |  |  |
|  |  |  |  |  |  |  |  |  |  |
|  |  |  |  |  |  |  |  |  |  |
|  |  |  |  |  |  |  |  |  |  |
|  |  |  |  |  |  |  |  |  |  |
|  |  |  |  |  |  |  |  |  |  |
|  |  |  |  |  |  |  |  |  |  |
| **GRADE** |  |  |  |  |  |  |  |  |  |
| Waste, medicine in residue water, human toxicity, ozone depletion were not reported and could not be graded. | | | | | | | | |  |
|  |  |  |  |  |  |  |  |  |  |
|  |  |  |  |  |  |  |  |  |  |
|  |  |  |  |  |  |  |  |  |  |
|  |  |  |  |  |  |  |  |  |  |
|  |  |  |  |  |  |  |  |  |  |
|  |  |  |  |  |  |  |  |  |  |
|  |  |  |  |  |  |  |  |  |  |
|  |  |  |  |  |  |  |  |  |  |
|  |  |  |  |  |  |  |  |  |  |
| **GRADE** |  |  |  |  |  |  |  |  |  |
| Two studies (McGain, 2021; Wang, 2022) reported on ‘climate change’. The level of evidence of this outcome measure was downgraded with 4 levels to **very low** due to risk of bias (-2; FU not defined and measurable and/or FU not justified and/or FU not consistent, Impact categories not documented transparently and/or characterization method not described and/or software used not described) and indirectness (-2; The system does not covers all stages: production (1), use/reuse (1) and disposal (1) of materials and energy, data not clearly described, sources and included data not clearly described, representativeness of data not discussed, differences in elektricity mix not accounted for, potential significance of ommitted stages not justified). | | | | | | | | |  |
|  |  |  |  |  |  |  |  |  |  |
|  |  |  |  |  |  |  |  |  |  |
|  |  |  |  |  |  |  |  |  |  |
|  |  |  |  |  |  |  |  |  |  |
|  |  |  |  |  |  |  |  |  |  |
|  |  |  |  |  |  |  |  |  |  |
|  |  |  |  |  |  |  |  |  |  |
| **GRADE** |  |  |  |  |  |  |  |  |  |
| One study (McGain, 2021) reported on ‘waste’. The level of evidence of this outcome measure was downgraded with 4 levels to **very low** due to risk of bias (-2; FU not defined and measurable and/or FU not justified and/or FU not consistent, Impact categories not documented transparently and/or characterization method not described and/or software used not described) and indirectness (-2; data were only collected from one study). | | | | | | | | |  |
|  |  |  |  |  |  |  |  |  |  |
|  |  |  |  |  |  |  |  |  |  |
|  |  |  |  |  |  |  |  |  |  |
|  |  |  |  |  |  |  |  |  |  |
|  |  |  |  |  |  |  |  |  |  |
|  |  |  |  |  |  |  |  |  |  |
|  |  |  |  |  |  |  |  |  |  |
| **GRADE** |  |  |  |  |  |  |  |  |  |
| Medicine in residue water, human toxicity, ozone depletion were not reported and could not be graded. | | | | | | | | |  |
|  |  |  |  |  |  |  |  |  |  |
|  |  |  |  |  |  |  |  |  |  |
|  |  |  |  |  |  |  |  |  |  |
|  |  |  |  |  |  |  |  |  |  |
|  |  |  |  |  |  |  |  |  |  |
|  |  |  |  |  |  |  |  |  |  |
|  |  |  |  |  |  |  |  |  |  |

### Table 13: Topic 5. Critical appraisal

Risk of bias table for interventions studies (cohort studies based on risk of bias tool by the CLARITY Group at McMaster University)

| **Author, year** | **Selection of participants  Was selection of exposed and non-exposed cohorts drawn from the same population?** | **Exposure  Can we be confident in the assessment of exposure?** | **Outcome of interest  Can we be confident that the outcome of interest was not present at start of study?** | **Confounding-assessment  Can we be confident in the assessment of confounding factors?** | **Confounding-analysis  Did the study match exposed and unexposed for all variables that are associated with the outcome of interest or did the statistical analysis adjust for these confounding variables?** | **Assessment of outcome  Can we be confident in the assessment of outcome?** | **Follow up  Was the follow up of cohorts adequate? In particular, was outcome data complete or imputed?** | **Co-interventions  Were co-interventions similar between groups?** | **Overall Risk of bias** |
| --- | --- | --- | --- | --- | --- | --- | --- | --- | --- |
| Alsved (2018) | Definitely no | Probably yes | Definitely yes | Definitely no | Definitely no | Probably yes | Probably yes | No information | Some concerns |
| Marsault (2021) | Definitely no | Probably no | Probably no | Definitely no | Definitely no | Probably yes | Probably yes | No information | High |

### Table 14: Topic 5. GRADE assessment per included outcome

| **Outcomes subquestion 5.1** | | | | |  | |  | |  | | | | |  |  |  |  |  |  |  |  |
| --- | --- | --- | --- | --- | --- | --- | --- | --- | --- | --- | --- | --- | --- | --- | --- | --- | --- | --- | --- | --- | --- |
| Climate change, energy use | | | | |  | | **Quality assessment** | |  | | | | |  |  |  |  |  |  |  |  |
| *No studies found* | | | | | | |  | |  | | | | |  |  |  |  |  |  |  |  |
|  |  |  |  |  |  |  |  | |  | | | | |  |  |  |  |  |  |  |  |
|  |  |  |  |  |  |  |  | |  | | | | |  |  |  |  |  |  |  |  |
|  |  |  |  |  |  |  |  | |  | | | | |  |  |  |  |  |  |  |  |
|  | |  | | |  | |  | |  | | | | |  |  |  |  |  |  |  |  |
| **Outcomes subquestion 5.2** | |  | | |  | |  | |  | | | | |  |  |  |  |  |  |  |  |
| Climate change | |  | | |  | | **Quality assessment** | |  | | | | |  |  |  |  |  |  |  |  |
|  | | | | | | | Climate change not reported and could not be graded. | | | | | | |  |  |  |  |  |  |  |  |
|  |  |  |  |  |  |  |  | |  | | | | |  |  |  |  |  |  |  |  |
|  |  |  |  |  |  |  |  | |  | | | | |  |  |  |  |  |  |  |  |
|  |  |  |  |  |  |  |  | |  | | | | |  |  |  |  |  |  |  |  |
|  | |  | | |  | |  | |  | | | | |  |  |  |  |  |  |  |  |
| Energy use | |  | | |  | | **Quality assessment** | |  | | | | |  |  |  |  |  |  |  |  |
| *Alsved, 2018; Marsault, 2021* | | | | | | | (bias) | |  | | | | |  |  |  |  |  |  |  |  |
|  |  |  |  |  |  |  | (indirectness) | |  | | | | |  |  |  |  |  |  |  |  |
|  |  |  |  |  |  |  | (imprecision) | |  | | | | |  |  |  |  |  |  |  |  |
|  |  |  |  |  |  |  |  | |  | | | | |  |  |  |  |  |  |  |  |
| **GRADE** |  | |  |  | |  |  |  | |  |  |  |  |  |  |  |  |  |  |  |  |
| No studies found on this subquestion, so no GRADE assessment. | | | | | | | | | | | |  |  |  |  |  |  |  |  |  |  |
|  |  |  |  |  |  |  |  |  |  |  |  |  |  |  |  |  |  |  |  |  |  |
|  |  |  |  |  |  |  |  |  |  |  |  |  |  |  |  |  |  |  |  |  |  |
|  |  |  |  |  |  |  |  |  |  |  |  |  |  |  |  |  |  |  |  |  |  |
|  |  | |  |  | |  |  |  | |  |  |  |  |  |  |  |  |  |  |  |  |
|  |  | |  |  | |  |  |  | |  |  |  |  |  |  |  |  |  |  |  |  |
| **GRADE** |  | |  |  | |  |  |  | |  |  |  |  |  |  |  |  |  |  |  |  |
| Climate change not reported and could not be graded. | | | | | | | | | | | |  |  |  |  |  |  |  |  |  |  |
|  |  |  |  |  |  |  |  |  |  |  |  |  |  |  |  |  |  |  |  |  |  |
|  |  |  |  |  |  |  |  |  |  |  |  |  |  |  |  |  |  |  |  |  |  |
|  |  |  |  |  |  |  |  |  |  |  |  |  |  |  |  |  |  |  |  |  |  |
|  |  | |  |  | |  |  |  | |  |  |  |  |  |  |  |  |  |  |  |  |
| **GRADE** |  | |  |  | |  |  |  | |  |  |  |  |  |  |  |  |  |  |  |  |
| The level of evidence regarding the outcome measure ‘energy use’ was downgraded to very low due to risk of bias (-1; lack of adequate adjustment for confounders) and imprecision (-1; lacking information on data distribution, use of dummy variables and surrogate endpoints). | | | | | | | | | | | |  |  |  |  |  |  |  |  |  |  |
|  |  |  |  |  |  |  |  |  |  |  |  |  |  |  |  |  |  |  |  |  |  |
|  |  |  |  |  |  |  |  |  |  |  |  |  |  |  |  |  |  |  |  |  |  |
|  |  |  |  |  |  |  |  |  |  |  |  |  |  |  |  |  |  |  |  |  |  |
